# Hyperactive neuronal networks facilitate tau spread in an Alzheimer’s disease mouse model

**DOI:** 10.1101/2024.12.01.625514

**Published:** 2024-12-03

**Authors:** Aaron J. Barbour, Keegan Hoag, Eli J. Cornblath, Abigail Chavez, Alfredo Lucas, Xiaofan Li, Sydney Zebrowitz, Chloe Hassman, Edward B. Lee, Kathryn A. Davis, Virginia M.Y. Lee, Delia M. Talos, Frances E. Jensen

**Affiliations:** 1 Department of Neurology, Perelman School of Medicine, University of Pennsylvania, Philadelphia, PA 19104; 2 Translational Neuropathology Research Laboratory, Perelman School of Medicine, University of Pennsylvania, Philadelphia, PA 19104; 3 Department of Pathology and Laboratory Medicine, Perelman School of Medicine, University of Pennsylvania, Philadelphia, PA 19104; 4 Institute on Aging, Perelman School of Medicine, University of Pennsylvania, Philadelphia, PA 19104; 5 Center for Neurodegenerative Disease Research, Perelman School of Medicine, University of Pennsylvania, Philadelphia, PA 19104

## Abstract

Pathological tau spreads throughout the brain along neuronal connections in Alzheimer’s disease (AD), but the mechanisms that underlie this process are poorly understood. Given the high incidence and deleterious consequences of epileptiform activity in AD, we hypothesized neuronal hyperactivity and seizures are key factors in tau spread. To examine these interactions, we created a novel mouse model involving the cross of targeted recombination in active populations (TRAP) mice and the 5 times familial AD (5XFAD; 5X-TRAP) model allowing for the permanent fluorescent labelling of neuronal activity. To establish a causal role of seizures in tau spread, we seeded mice with human AD brain-derived tau lysate and induced seizures with pentylenetetrazol (PTZ) kindling. Comprehensive brain mapping of tau pathology and neuronal activity revealed that basal hyperactivity in 5X-TRAP mice was associated with increased tau spread, which was exacerbated by seizure induction through activated networks and correlated with memory deficits. Computational modeling revealed that anterograde tau spread was elevated in 5X-TRAP mice and that regional neuronal activity was predictive of tau spread to that brain region. On a cellular level, we found that in both saline and PTZ-treated 5X-TRAP mice, hyperactive neurons disproportionately contributed to the spread of tau. Further, we found that Synaptogyrin-3, a synaptic vesicle protein that interacts with tau, was increased following PTZ kindling in 5X-TRAP mice, possibly indicative of a synaptic mechanism underlying seizure-exacerbated tau spread. Importantly, postmortem AD brain tissue from patients with a history of seizures showed increased tau pathology in patterns indicative of increased spread and increased Synaptogyrin-3 levels compared to those without seizures. Overall, our study identifies neuronal hyperactivity and seizures as key factors underlying the pathobiological and cognitive progression of AD. Therapies targeting these factors should be tested clinically to slow tau spread and AD progression.

## Introduction

The spread of the pathological tau protein is central to the progression of Alzheimer’s disease (AD), and proceeds along functional neuroanatomical connections (1–6), with pathological tau burden closely correlating with cognitive decline (7–10). Human AD imaging and postmortem studies demonstrate that tau pathology begins in the locus coeruleus, and spreads to the entorhinal cortex, followed by the limbic system and neocortex (10–12). In mice, intracerebral injection of human AD brain-derived tau lysate (AD-tau), tau seeding, results in misfolding and propagation of endogenous tau throughout the brain along neuronal connections (13), as seen in human AD. Tau spread is worsened by the presence of β–amyloid (Aβ) pathology in humans (1) and the five times familial AD (5XFAD) and other amyloidogenic rodent models (14, 15). Patterns of tau spread in AD and AD-tau seeded mice suggest trans-neuronal propagation of tau with computational models supporting a connectome-directed spread of tau pathology (16). Indeed, these models suggest that tau spreads in both retrograde and anterograde directions in seeded mice (17) and humans (1, 6, 18, 19). *In vitro* studies have shown that neuronal activity can induce synaptic tau release (20) and may promote anterograde tau spread. At the synapse, tau can bind Synaptogyrin-3 (SYNGR3) (21, 22), a presynaptic vesicular protein, which may be requisite for its release into the synaptic cleft, where external tau can bind lipoprotein receptor-related protein 1 (LRP1), and undergo postsynaptic internalization (23), thereby providing a potential mechanism of anterograde synaptic tau transmission. Understanding the factors that drive tau spread throughout the brain and the underlying mechanisms will yield novel therapeutic targets and strategies to slow disease progression.

Neuronal network hyperexcitability is prevalent in AD, particularly at early stages (24), with unprovoked seizures occurring in up to 22% of AD patients (25). We and others have found worsened AD pathology and cognitive outcomes in AD patients with a seizure history (25–30), suggestive of a bidirectional relationship between network hyperactivity and AD progression. Similarly, neuronal hyperactivity is found in 5XFAD mice, beginning at early, prodromal stages (26, 27) and seizure induction with pentylenetetrazol (PTZ) kindling exacerbates amyloid pathology, excitatory: inhibitory imbalance, and cognitive deficits (26, 27). Aβ-producing mouse models also show neuronal hyperactivity at the cellular level (26, 31), with a positive feedback loop occurring between neuronal hyperactivity and Aβ pathology (32, 33). While network to cellular level neuronal hyperexcitability are well-characterized phenomena in AD, the role of neuronal hyperactivity in tau spread is not well understood.

Recently developed genetic tools enable the permanent fluorescent (tdTomato, tdT) labelling and tracking of activated neuronal populations (Targeted Recombination in Active Population; TRAP) (34, 35). With these mice, we and others have demonstrated that neurons that underwent activity dependent tdT labelling during seizures have long-lasting hyperexcitability, synaptic alterations, and pathological effects (36–38), and are promising therapeutic targets. Here, we created a novel model by crossing TRAP mice with 5XFAD (5X-TRAP/WT-TRAP) to examine the interactions between tau spread and neuronal hyperactivity on a network and cellular level.

We hypothesized that neuronal hyperactivity would be associated with increased anterograde tau spread in 5X-TRAP mice and would be exacerbated by seizure induction. We additionally hypothesized that these changes in anterograde tau spread would be associated with dysregulation of synaptic tau interactors. To establish a causal role of seizures and neuronal hyperactivity in tau propagation, we seeded WT-TRAP and 5X-TRAP mice with AD-tau, induced seizures with PTZ kindling, and labelled all seizure activated neurons with tdT. With comprehensive brain mapping and computational analyses, we found that network hyperactivity was associated with increased anterograde tau spread in 5X-TRAP mice and was exacerbated by PTZ kindling through activated circuits. Memory deficits in PTZ kindled 5X-TRAP mice were highly correlated with tau spread and neuronal hyperactivity. Further, we found that hyperactive, tdT+ neurons drove increased tau spread in 5X-TRAP mice and seizure-induced increases in tau spread were associated with increased SYNGR3 providing a potential mechanism by which seizures may facilitate synaptic tau spread. To increase translational relevance, we examined tau pathology and levels of synaptic tau-interacting proteins in AD brain tissue and found increased indications of tau spread and elevated SYNGR3 in AD patients with a clinical seizure history compared to those without. Overall, these comprehensive studies demonstrate that hyperactive networks and neurons facilitate anterograde tau spread in AD and identify seizures and neuronal hyperexcitability as mitigatable and translatable targets to slow disease progression.

## Results

**Figure 1.**
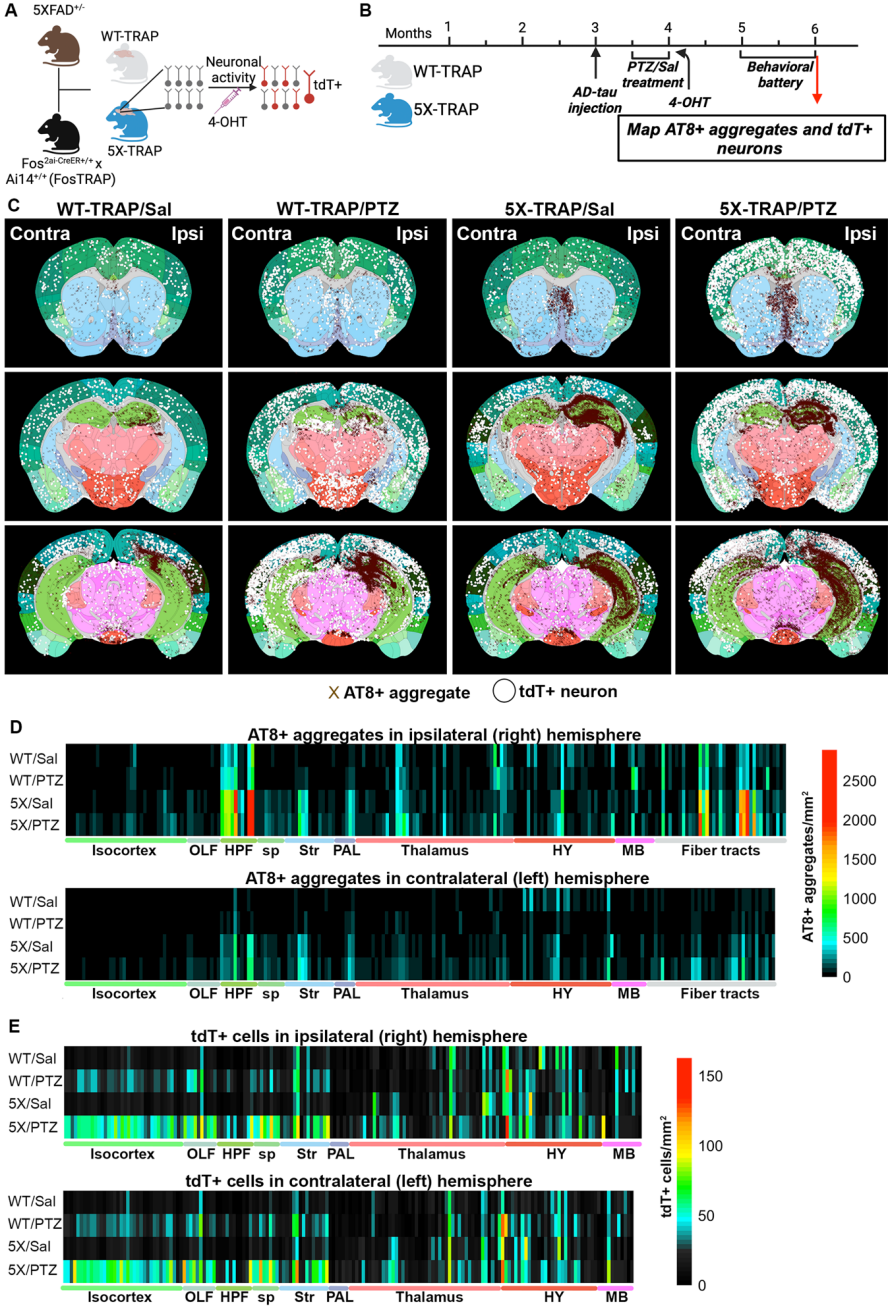
Brain mapping reveals increased tau spread in 5X-TRAP mice that is exacerbated by seizure induction and increased tdT+ labeling in PTZ and saline treated 5X-TRAP mice. (**A**) Schematic for the generation of, and activity-dependent labelling in WT-TRAP and 5X-TRAP mice. (**B**) Experimental timeline: WT-TRAP and 5X-TRAP mice underwent AD-tau seeding at three months of age, PTZ seizure kindling (or control, sal injection) at 3.5–4 months of age, and behavioral battery at 5–6 months of age. Brains sections underwent IHC, imaging, and AT8+ aggregate and tdT+ mapping. (**D**) Three representative sections from each experimental group displaying detected AT8+ tau aggregates (brown X) and tdT+ cells (white circles) mapped to the Allen Brain Atlas. (**D**) Heatmaps for each experimental group (rows) with each column displaying AT8+ aggregate densities in every sampled brain region in the hemispheres ipsilateral (right, top) and contralateral (left, bottom) to AD-tau injection site. (**E**) Heatmap with each cell displaying tdT+ cell count densities in every sampled brain region in the ipsilateral (right, top) and contralateral (left, bottom) hemispheres. The mean, SEM, sample size, and statistical results for AT8+ and tdT+ counts can be found in Tables S1–S4. Contra=contralateral, ipsi=ipsilateral, HPF=hippocampal formation, OLF=olfactory areas, sp=cortical subplate, Str=striatum, PAL=pallidum, HY=hypothalamus, MB=midbrain.

To study the interactions between AD pathology and neuronal activity, we developed a novel model by crossing 5XFAD mice with mice homozygous for Fos^2A-iCreER^ (TRAP2) and B6.Cg-*Gt(ROSA)26Sor*^tm14(CAG-tdTomato)Hze^/J (Ai14) (Fig 1A). The resulting 5X-TRAP and WT-TRAP mice allow for robust, permanent tdT expression in neurons that express the activity-dependent cFos protein during the 4–6-hour window after 4-hydroxytamoxifen (4-OHT) induced CreER expression (Fig 1A). The 5X-TRAP model thus allows for the examination of the interactions between brain-wide neuronal activity patterns and AD pathology.5X-TRAP and WT-TRAP mice underwent unilateral AD-tau injection into the right hippocampus and overlying cortex at 3 months of age. Two to three weeks following AD-tau seeding, PTZ kindling or control (saline, Sal) protocols were performed and seizure or basally-activated neurons were labelled with tdT. At 5–6 months of age, mice underwent behavioral testing and were euthanized at 6 months of age (3 months post AD-tau injection) (Fig 1B). Sectioned brain samples were then registered to the Allen Brain Atlas (ABA) and tau pathology, assessed by AT8 (phospho-tau Ser202, Thr205)+ aggregates, along with detected tdT+ cells, were mapped and quantified throughout the brain (Fig 1C, D, E, Table S1–S4). Statistics for brain mapping results can be found in Table S1–S4.

Mapping of AT8+ aggregates revealed that the 5XFAD genotype was overall associated with significantly increased tau spread in both the ipsilateral (right) and contralateral (left) hemispheres to AD-tau injection site, compared with WT-TRAP mice (Fig 1C, D, Table S1, S2). 5X-TRAP mice exhibited increased AT8+ aggregates in subcortical regions including the ipsilateral and contralateral hippocampus, basolateral amygdala (BLA), striatum, and ventral thalamic nuclei. In addition, we found significantly increased AT8+ aggregates in the contralateral isocortex overall and the ipsilateral prelimbic and retrosplenial cortex. Of note, there was also increased AT8+ aggregates in the fornix and corpus callosum of 5X-TRAP mice. Together, these data suggest that axonal transport of tau in 5X-TRAP mice results in enhanced intra- and cross-hemispheric spread of tau compared to WT-TRAP.

While 5X-TRAP mice showed increased tau spread at baseline, we also found that PTZ kindling exacerbated tau spread in 5X-TRAP mice. AT8+ aggregates were significantly increased in PTZ kindled 5X-TRAP mice compared to Sal or PTZ treated WT-TRAP in the isocortex (ipsilaterally in anterior cingulate, auditory, primary somatosensory, somatomotor areas, and posterior parietal association areas, and contralaterally in the anterior cingulate, somatomotor, gustatory, and visual areas), as well as the ipsilateral central amygdalar nucleus. We also found that compared to Sal treated 5X-TRAP, PTZ kindled 5X-TRAP mice had significantly increased AT8+ aggregates including in the ipsilateral somatosensory and rostrolateral visual cortex and dorsal thalamic nuclei, the contralateral isocortex overall, lateral thalamic nuclei, lateral septal complex, and in fiber tracts. These data suggest that PTZ induced seizures enhanced tau spread via axonal transport and through thalamic nuclei and cortical regions that have reciprocal connections and are strongly activated by PTZ (39, 40), including intralaminar nuclei of the dorsal thalamus and motor, parietal, and anterior cingulate cortices (41, 42). In contrast, PTZ kindling in WT-TRAP mice did not increase AT8+ aggregates, indicative of an interaction between seizures and Aβ pathology on tau spread.

In addition to tau pathology, we compared tdT+ counts throughout the brains of Sal and PTZ treated 5X-TRAP and WT-TRAP mice (Fig 1C, E, Tables S3, S4). As expected, PTZ kindled mice had increased tdT+ counts across both genotypes compared to Sal treated mice in both ipsilateral and contralateral hemispheres, overall, demonstrating increased activity-dependent neuronal labelling during seizures, consistent with our findings in a kainic acid seizure model (36). In addition, 5XFAD genotype was associated with increased tdT+ counts in the ipsilateral and contralateral isocortex and thalamus, regardless of kindling. Sal treated 5X-TRAP mice had increased tdT+ counts compared to Sal treated WT-TRAP in contralateral ventral and lateral thalamic nuclei, demonstrating basal hyperactivity in 5X-TRAP mice. PTZ kindled 5X-TRAP mice showed increased tdT+ counts compared to PTZ kindled WT-TRAP in across several ipsilateral and contralateral cortical areas, and in the ipsilateral and contralateral CA1, BLA, and thalamus, including the dorsal intralaminar nuclei, demonstrating worsened PTZ induced neuronal hyperactivity in 5X-TRAP mice. Together these data demonstrate basal hyperactivity in AD-tau seeded 5X-TRAP mice, which is exacerbated by PTZ kindling.

Overall, our mapping data demonstrate that regions with increased relative AT8+ aggregates were hyperactive (increased tdT+ counts) in both Sal and PTZ treated 5X-TRAP mice. Of note, PTZ kindled 5X-TRAP mice showed increased AT8+ aggregates in the intralaminar nuclei of the dorsal thalamus, somatomotor and anterior cingulate cortex, which are areas where we also found significantly increased levels of tdT+ counts compared to all other groups and has previously been shown to be activated by PTZ (39, 40). Sal treated 5X-TRAP mice also showed increased tau spread in areas with basal hyperactivity compared to Sal treated WT-TRAP. These data demonstrate that tau is spread through hyperactive networks in 5X-TRAP mice and enhanced by PTZ kindling.

To determine whether sex may influence tau spread and tdT labelling, we included sex as an independent variable in multiple linear regression for AT8+ aggregate and tdT+ counts (Table S5). We found that female sex was associated with worsened tau spread in the ipsilateral and contralateral hippocampi. Sex had a less pronounced effect on tdT+ counts, but it was found to be a predictor in the ipsilateral olfactory areas with elevations found in males. These results suggest that female sex is associated with increased tau spread, but these effects are not driven by neuronal hyperactivity.

### Memory deficits in PTZ kindled 5X-TRAP mice are correlated with thalamic and cortical tau spread and hippocampal hyperactivity

**Figure 2.**
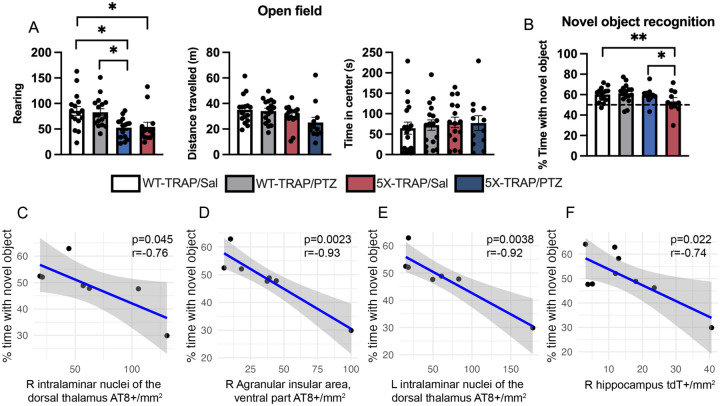
Tau spread and neuronal hyperactivity are associated with memory deficits in PTZ-kindled 5X-TRAP mice. Two-way ANOVA with genotype (WT-TRAP vs 5X-TRAP) and kindling (Sal vs PTZ) were performed for behavioral tests (**A**, **B**). (**A**) 5X-TRAP mice showed reduced rearing behavior (left; genotype effect: p<0.0001) and total distance travelled (center; genotype effect: p<0.05) without change in time spent in center (right). Sidak’s post hoc: *p<0.05; n=13–17/group. (**B**) PTZ-kindled 5X-TRAP mice had significantly worse novel object memory than WT-TRAP mice and saline-treated 5X-TRAP mice (interaction p<0.05). *, **=Sidak’s post hoc p<0.05, 0.01; n=10–16/group. Pearson’s correlation revealed AT8+ aggregate levels in the (**C**) ipsilateral (right, R) intralaminar nuclei of the dorsal thalamus, (**D**) ipsilateral agranular insular area, and (**E**) contralateral (left, L) intralaminar nuclei of the dorsal thalamus were significantly negatively associated with novel object memory in PTZ-kindled 5X-TRAP mice. (**F**) Pearson’s correlation revealed that tdT+ counts in the R hippocampus were significantly negatively associated with novel object memory in PTZ-kindled 5X-TRAP mice. Histograms display group mean ± SEM.

We have previously shown that PTZ kindling exacerbates memory deficits in 5XFAD mice (27). Here, we sought to determine whether PTZ would have similar effects in AD-tau seeded 5X-TRAP mice, and whether memory impairment would correlate with regional AT8+ aggregates or tdT+ counts. All Sal and PTZ treated WT-TRAP and 5X-TRAP mice underwent a behavioral battery at 5–6 months of age (2–3 months post AD-tau seeding) (Fig 1B), including open field assay, novel object recognition (NOR), and contextual fear conditioning (CFC). In the open field assay, we found decreased rearing behavior and total distance traveled in 5X-TRAP mice, overall, compared to WT-TRAP, suggestive of decreased exploratory behavior and motor activity (Fig 2A). We found similar time spent in the center of the arena across all groups indicating no differences in anxiety-like behavior (Fig 2A). Female sex was associated with decreased rearing behavior in the open field assay (Table S5). In the NOR paradigm, the % time spent with the novel object as compared to the familiar object was significantly decreased in PTZ kindled 5X-TRAP mice compared to Sal treated 5X-TRAP mice (Fig 2B). In contrast CFC testing at 1 day (recent memory) and 14 days (remote memory) following conditioning revealed no differences between groups in recent or remote recall (Fig S1). No effects of sex were found on CFC or NOR (Table S5). Overall, these results confirm prior reports of reduced locomotor activity in 5XFAD mice (43) and demonstrate that seizures significantly worsen NOR in AD-tau seeded 5X-TRAP mice.

To determine the associations between seizure activity and tau spread on cognition, we performed Pearson correlations for NOR performance and regional AT8+ aggregate and tdT+ counts in PTZ kindled 5X-TRAP mice. We found strong inverse correlations between performance on NOR and AT8+ aggregate levels in the ipsilateral intralaminar nuclei of the dorsal thalamus and agranular insular area, and in the contralateral intralaminar nuclei of the dorsal thalamus (Fig 2C–E), areas in which we found elevated tau spread in PTZ kindled 5X-TRAP mice (Fig 1C, Table S1, S2). In addition, we found that decreased NOR performance was significantly correlated with tdT+ counts in the ipsilateral hippocampus of PTZ kindled 5X-TRAP mice (Fig 2F). These results demonstrate that seizure-induced exacerbation of tau spread through thalamo-cortical circuits and hippocampal hyperactivity after PTZ administration are associated with worsened memory deficits.

### Computational modeling reveals that 5XFAD genotype is associated with increased anterograde tau spread

**Figure 3.**
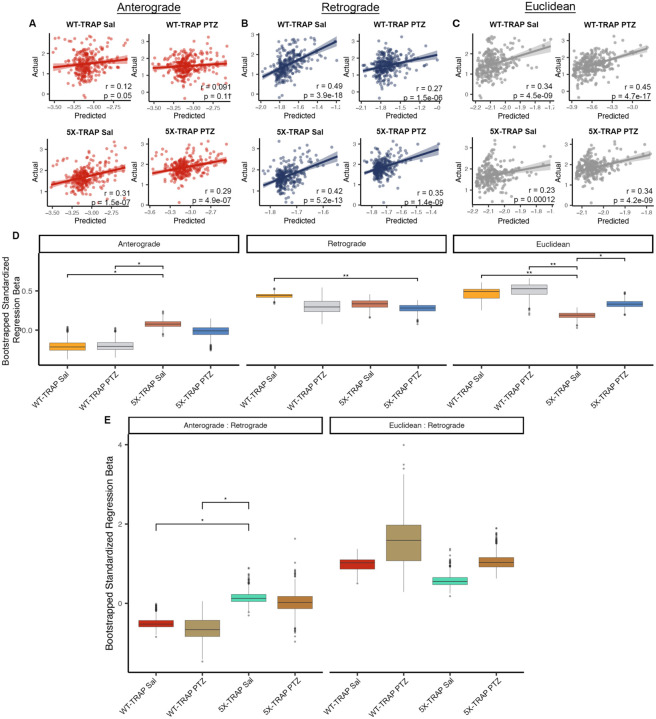
Computational modeling reveals an increased impact of anterograde tau spread in 5X-TRAP mice. Predictions of tau spread based on (**A**) anterograde, (**B**) retrograde, and (**C**) Euclidean models of spread plotted against our measured levels for each experimental group. Solid lines represent best fit and shaded regions are the 95% confidence interval. (**D**) For the Bidirectional Euclidean + tdT spread model architecture, we performed a bootstrap comparison of mean standardized regression beta weights between groups and adjusted for multiple comparisons. (**E**) For the Bidirectional Euclidean spread model architecture, we performed a bootstrap comparison of mean ratio of standardized anterograde to retrograde regression beta weights between groups and adjusted for multiple comparisons. p-values were adjusted for multiple comparisons in each panel using FDR correction: *p < 0.05, **p<0.01.

**Figure 4.**
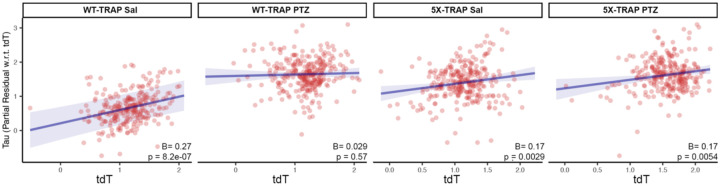
Regional tdT+ levels are predictive of tau spread. We modeled tau pathology as a function of anterograde spread, retrograde spread, Euclidean distance-based spread, tdT positive cell counts, and interactions between PTZ treatment and tdT positive cell counts. tdT levels were predictive of tau spread in all groups except PTZ treated WT-TRAP mice.

Given our data demonstrating increased tau spread through hyperactive networks in 5X-TRAP mice, we next determined whether 5XFAD genotype or seizures were associated with altered patterns of spread. Prior studies using computational modeling of tau pathological progression have demonstrated that tau spreads throughout the brain via the neuroanatomical connectome, predominantly in a retrograde direction yet with a small anterograde component (17). To determine if 5XFAD genotype and PTZ alter the patterns of tau spread, we generated linear diffusion models of tau spread from a hippocampal seed along a network defined by anterograde connectivity, retrograde connectivity, or Euclidean distance between brain areas derived from Oh and colleagues (44). We tested whether linear combinations of these different spread models would be more effective in predicting regional AT8+ aggregate counts from each experimental group (Fig 3, S2). Consistent with prior data (17), we found that retrograde models tended outperform to anterograde and Euclidean models (Figure 3A–C, Figure S2). Additionally, anterograde models produced a statistically significant fit with 5X-TRAP mice but not with WT-TRAP mice (Figure 4A). A model combining anterograde, retrograde, and Euclidean distance called “Bidirectional Euclidean” fit better than any individual model in all groups (Fig S2). Overall, these results were consistent with prior analysis of tau spread and suggest that anterograde connectivity may contribute more to spread in 5X-TRAP mice, while PTZ treated mice tended to have lower contributions of retrograde spread and higher contributions of Euclidean distance-based spread.

To further examine the relative importance of anterograde, retrograde, and Euclidean tau spread, we compared standardized regression beta weights from the “Bidirectional Euclidean” model (Fig S2) between groups (Fig 3D). We found that anterograde beta weights (Fig. 3D) and anterograde: retrograde beta weight ratios (Fig 3E) were increased in Sal treated 5X-TRAP mice compared to WT-TRAP. Beta weights were similar between PTZ and Sal treated 5X-TRAP mice (Fig 3D). Retrograde betas were lower in PTZ kindled 5X-TRAP mice compared to Sal treated WT-TRAP mice and Euclidean spread betas were lower in Sal treated 5X-TRAP compared to all other groups (Fig 3D), suggesting that anterograde connectivity contributes more to tau spread than retrograde or Euclidean based in 5X-TRAP mice. Overall, these computational analyses show that all groups fit with a pattern of connectome-based spread and that anterograde spread contributes to the elevations in AT8+ aggregates quantified in our brain mapping data in Sal and PTZ treated 5X-TRAP mice (Fig 1).

### Neuronal activity levels are predictive of tau spread

Given that elevated tau spread in 5X-TRAP mice tended to occur in hyperactive regions, we next determined whether neuronal activity levels were predictive of tau spread. To do so, we correlated tdT+ counts with AT8+ aggregates while accounting for predicted Bidirectional Euclidean spread. We found significant positive relationships between tdT+ counts and relative AT8+ aggregate levels in Sal treated WT-TRAP mice and Sal and PTZ treated 5X-TRAP mice, demonstrating that neuronal activity (tdT+ count) is predictive of AT8+ aggregates in these groups (Fig 4). tdT+ counts were not predictive of relative tau spread in PTZ treated WT-TRAP mice, since PTZ increased tdT counts without change in AT8+ aggregates in these mice (Fig 1, Table S1–S4). Together, these data suggest that neuronal hyperactivity contributes to increased tau aggregation found in 5X-TRAP mice.

### Hyperactive neurons are more likely to develop tau pathology than surrounding neurons

**Figure 5.**
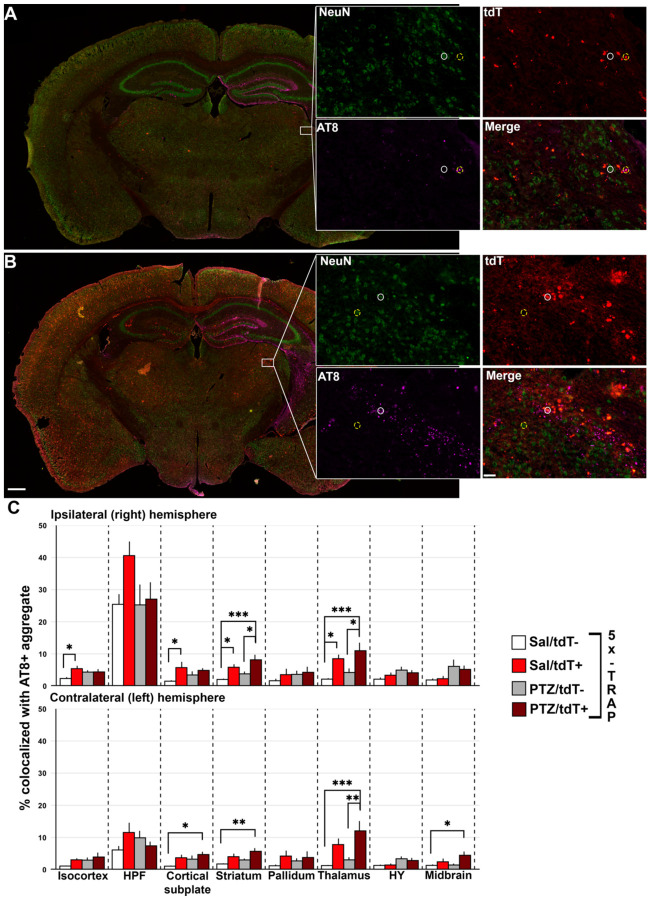
tdT+ neurons are more likely to colocalize with AT8+ aggregates in both saline and PTZ treated 5X-TRAP mice. Representative slide scanned coronal sections from (**A**) saline and (**B**) PTZ treated 5X-TRAP mice. Inset displays thalamic tdT+ (white circles) and tdT− (NeuN+) (yellow dashed circles) neurons colocalization with AT8+ tau aggregates. Scale bars = 500 μm, 20 μm (inset). (**C**) tdT+, tdT−, and colocalized AT8+ aggregates were mapped to the Allen Brain Atlas and % of detected tdT+/tdT− cells were quantified. Two-way ANOVA was performed with treatment (Sal vs PTZ) and cell type (tdT− vs tdT+) as independent variables. There was a significant increase in % tdT+ neurons colocalized with AT8+ aggregates than surrounding tdT− in Sal treated 5X-TRAP mice in the ipsilateral thalamus, striatum, and isocortex. There was a significant increase in % tdT+ neurons colocalized with AT8+ aggregates than surrounding tdT− cells in PTZ treated mice in the ipsilateral thalamus and striatum and contralateral thalamus. Tukey’s post hoc: *p<0.05, **p<0.01, ***p<0.001. Histograms display group mean ± SEM.

Given our results establishing that tau spreads through hyperactive networks in 5X-TRAP mice, we next determined whether hyperactive neurons drive pathological tau spread. We examined whether tdT+ neurons were more likely to develop somatic AT8+ aggregates compared to surrounding tdT− (NeuN+) neurons. Detected and mapped tdT+ and tdT− (NeuN+) neurons were colocalized with AT8+ aggregates across all sampled brain regions to calculate the percentage of tdT+ and tdT− neurons with somatic tau pathology. 5X-TRAP (Fig 5) and WT-TRAP (Fig S3) mice were examined separately by two-way ANOVA with kindling (PTZ vs Sal) and tdT labelling (tdT+ vs tdT-) as independent variables. Ipsilaterally, we found increased AT8+ aggregate colocalization in tdT+ neurons compared to tdT− in Sal treated 5X-TRAP mice in the isocortex, cortical subplate, striatum, and thalamus and increased AT8+ aggregate colocalization in tdT+ neurons compared to tdT− in PTZ kindled 5X-TRAP mice in the striatum and thalamus. In the contralateral hemisphere, we found that tdT+ neurons were more likely to colocalize with AT8+ aggregates than surrounding tdT− neurons in the thalamus of PTZ kindled 5X-TRAP mice (Fig 5). Given our results showing increased AT8+ aggregates and high tdT+ counts in the cortex of PTZ treated 5X-TRAP mice, we expected to see increased tdT+ colocalization with AT8+ aggregates in the cortex, but we found no differences in these mice. These results are likely due to increased neuronal death (Fig S4) in hyperactive cortical neurons carrying a high tau load after PTZ kindling. In contrast, WT-TRAP mice did not exhibit any significant differences in AT8+ aggregate colocalization due to kindling or tdT labelling (Fig S3). In summary, both Sal and PTZ treated 5X-TRAP mice exhibited increased AT8+ aggregate colocalization in tdT+ neurons compared to tdT− neurons in regions with increased overall AT8+ aggregates and tdT+ counts, demonstrating that tau disproportionately spreads through hyperactive neurons in these mice.

### Seizures dysregulate synaptic tau interactors in 5XFAD/5X-TRAP mice

**Figure 6.**
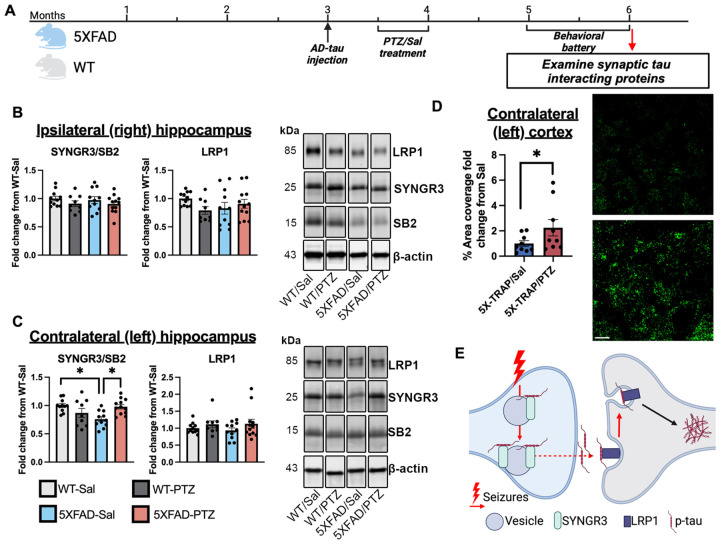
Dysregulation of SYNGR3 due to 5XFAD genotype and PTZ kindling. (**A**) Experimental timeline for WT and 5XFAD mice. SYNGR3 and LRP1 were examined by western blot in the hippocampi (**B**) ipsilateral (right) and (**C**) contralateral (left) to AD-tau injection. SYNGR3 was normalized to synaptobrevin-2 (SB2) to determine SYNGR3 levels relative to levels of synaptic vesicles and LRP1 was normalized to β-actin. Two-way ANOVA with genotype (WT vs 5XFAD) and kindling (Sal vs PTZ) were performed for western blot data. There was a reduction in SYNGR3/SB2 in the contralateral hippocampus of Sal treated 5XFAD mice compared to Sal treated WT and PTZ kindled 5XFAD without change in LRP1. Tukey’s post hoc: *p<0.05, **p<0.01; n=9–12/group. (**D**) IHC in the CA1 stratum radiatum contralateral (left) to AD-tau injection demonstrated increased SYNGR3 levels in PTZ kindled 5X-TRAP mice compared to Sal treated 5X-TRAP mice. T test compared genotypes: *p<0.05; n=9/group. Representative images show maximum intensity projections from the CA1 stratum radiatum of Sal treated (left) and PTZ kindled (right) 5X-TRAP mice. Scale bar = 10 μm. (**E**) Schematic for potential mechanism of synaptic tau spread by seizures in 5X-TRAP mice. Histograms display group mean ± SEM.

Our data suggest increased anterograde tau spread through hyperactive brain regions and neurons in 5X-TRAP mice. We hypothesized that these changes may be associated with dysregulation of synaptic tau interactors. A separate cohort of 5XFAD and WT mice underwent AD-tau seeding and PTZ kindling as described above, but without 4-OHT treatment and behavioral testing (Fig 6A). Brains were collected for western blot analysis of the presynaptic vesicular protein SYNGR3 and the endocytic LRP1, given their known roles in tau interactions at the synapse (21–23). SYNGR3 levels were normalized to synaptobrevin-2 (SB2) to control for potential changes in synapse/synaptic vesicle levels. We found that while there were no changes in the ipsilateral hippocampus (Fig 6B), in the contralateral hippocampus, SYNGR3/SB2 levels were significantly decreased in Sal treated 5XFAD mice compared to Sal treated WT and PTZ kindled 5XFAD mice (Fig 6C). No changes in LRP1 were found (Fig 6B, C). No significant effects of sex were found on SYNGR3/SB2 or LRP1 levels (Table S5). We confirmed an increase in SYNGR3 in the stratum radiatum of the contralateral CA1 in PTZ-kindled 5X-TRAP mice compared to Sal treated 5X-TRAP mice by IHC (Fig 6D). These data demonstrate that increased tau spread in PTZ kindled 5X-TRAP mice is associated increased SYNGR3 levels.

### Increased tau pathology and dysregulation of synaptic tau interactors in human postmortem AD brain is associated with a history of seizures.

**Figure 7.**
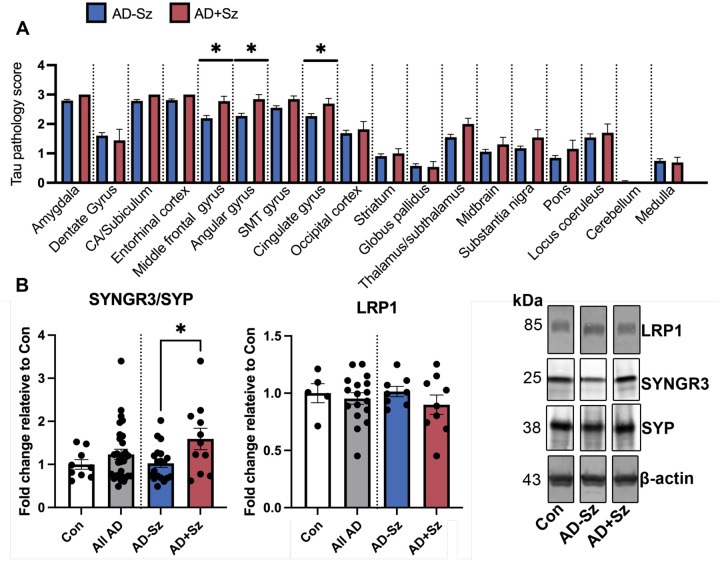
Worsened tau pathology and dysregulation of SYNGR3 in AD patients with a history of seizures. (**A**) Tau pathology severity ratings from postmortem analysis of AD-Sz and AD+Sz were compared by Mann-Whitney test: *p<0.05. AD-Sz n= 81–91/brain region, AD+Sz n=9–13/brain region. SMT gyrus= superior/middle temporal gyrus. (**B**) SYNGR3 and LRP1 were examined by western blot in control (con), AD-Sz, and AD+Sz temporal lobe cortex. SYNGR3 was normalized to synaptophysin (SYP) to determine SYNGR3 levels relative to synaptic vesicles and LRP1 was normalized to β-actin. Con to all AD and AD-Sz to AD+Sz comparisons were made by t tests. AD+Sz showed increased SYNGR3/SYP and decreased BIN1 compared to AD-Sz. Histograms display group mean ± SEM.

To determine whether seizures may worsen indications of tau spread in AD, we analyzed postmortem pathological ratings across 18 different brain regions from AD patients and stratified subjects based on the presence or absence of clinical seizure history. We found that AD patients with clinical seizure history (AD+Sz) exhibited more severe tau pathological ratings (phosphorylated tau (Ser396/404); PHF1 antibody) compared to AD patients without a clinical seizure history (AD-Sz). We found these alterations in regions affected at intermediate and late Braak stages including the cingulate gyrus, middle frontal gyrus, and angular gyrus (Fig 7A). We did not find significant differences in regions involved in earlier Braak stages including the locus coeruleus and entorhinal cortex (Fig 7A). We additionally performed ordinal linear regressions and found that no effect of sex on tau pathology (Table S6). These patterns indicate that a history of seizures may be associated with increased tau spread in AD.

Given the findings in the 5XFAD mouse model, we next determined whether there were alterations to the synaptic tau interacting proteins, SYNGR3 and LRP1, by western blot in the temporal lobe tissues of control, AD-Sz, and AD+Sz, where we have previously found increased phospho-tau in AD+Sz compared to the AD-Sz group (27). SYNGR3 levels were normalized to synaptophysin (SYP) to control for potential changes in synaptic/synaptic vesicle levels. We found that AD+Sz had significantly increased SYNGR3/SYP compared to AD-Sz (Fig 7B). In addition, female sex was associated with elevated SYNGR3/SYP level (Table S6). No changes in LRP1 were found (Fig 7B). These results are consistent with those found in PTZ kindled 5XFAD mice and demonstrate that worsened tau pathology in AD+Sz is associated with increased relative SYNGR3 levels.

## Discussion

Our comprehensive study demonstrates for the first time that neuronal hyperactivity drives increased anterograde pathological tau spread through activated brain networks and neurons in an AD model. Our data also highlight that tau spread and neuronal hyperactivity are highly correlated with worsened memory caused by seizures and provide a mechanism by which seizures may facilitate the synaptic spread of tau in 5X-TRAP mice. We found corroborating data in postmortem human AD brain tissue, suggestive of increased tau spread in patients with a history of seizures. Together, our data demonstrate hyperactive networks and neurons as therapeutic targets to slow AD progression.

TRAP models are widely used in basic research, and here we report the development of a new model involving the cross of these mice with a neurodegenerative model. With tamoxifen-inducible, neuronal activity (Fos) driven labelling, we used the 5X-TRAP mouse to examine the interactions between neuronal activity and AD pathology. We found that 5X-TRAP mice show basal increases in activity-dependent tdT labelling, corroborating prior reports of hyperexcitability in amyloidosis models (26, 45), which may underlie increased seizure susceptibility in 5XFAD mice (27). Our recent work demonstrated that tdT labelling during seizure induction allows for the long-term tracking of hyperactive neuronal populations (36). Thus, 5X-TRAP mice provided a robust model to test our hypothesis that neuronal hyperactivity is actively involved in disease progression.

Injection of pathological tau in the mouse brain recapitulates the connectome-directed spread seen in human AD (13, 17). To examine the interactions between Aβ pathology, neuronal hyperactivity, and tau spread, we performed AD-tau seeding followed by PTZ seizure kindling in 5X-TRAP and WT-TRAP mice and brain mapping. First, we found that Sal treated 5X-TRAP mice had significantly increased tau spread, relative to WT-TRAP mice, in subcortical and cortical regions and fiber tracts, which is consistent with prior reports of increased spread in both the 5XFAD model and amyloid precursor protein knock-in mouse (14, 15), and suggests trans-neuronal tau spread. Next, we observed that PTZ kindling caused further increases in tau spread in 5X-TRAP mice in several cortical regions, the thalamus, striatum, and fiber tracts. In both PTZ and saline-treated 5X-TRAP mice, regions with high tdT counts were associated with relative increases in tau pathology, including the thalamus and areas of the isocortex. PTZ induces thalamo-cortical seizure activity (39, 40), and our data show increased spread particularly in interconnected regions of the thalamus and cortex that were hyperactive and in fiber tracts. These data suggest that seizure induced hyperactivity drives axonal transport and trans-neuronal tau spread through activated networks. Tau levels in these regions were also associated with worsened cognitive performance in these mice, consistent with human studies linking cognitive decline and tau progression in AD (7–10), and further establishing a role of seizure-induced tau spread in overall disease progression.

PTZ kindling had little effect on tau spread measured by AT8+ aggregates in WT-TRAP mice, indicating that interactions between amyloid pathology and neuronal hyperactivity are involved in enhanced seeding effects. Notably, we have found that PTZ kindling also increases Aβ pathology in 5XFAD mice (27), which in turn can further increase and promote sustained hyperactivity, creating a positive feedback loop (33), which may result in enhanced tau seeding effects. Together, our mapping data suggest that Aβ and neuronal hyperactivity interactions promote the axonal transport and trans-neuronal spread of tau.

Of note, we found a significant effect of female sex to increase tau spread. While similar changes were not found in our human cohort, these cases were late-stage and our results in mice are consistent with tau positron emission tomography (PET) studies in patients with mild cognitive impairment (46). Our data do not suggest that sex effects on tau spread are due to sex-dependent changes in neuronal hyperactivity as we found little difference between males and females in tdT labeling. We have previously demonstrated that female 5XFAD mice had increased Aβ plaques and mTORC1 activation compared to males (27). Thus, increased tau spread in females found here may be due to increased neuritic plaque tau or a reduction in autophagy, caused by elevated mTOR activity (47), resulting in increased intracellular tau accumulation. Future studies should further investigate the mechanisms underlying sex and tau spread interactions.

To determine whether genotype or seizure induction significantly altered spread patterns, we utilized connectomic data made available from the Allen Institute (44). Consistent with prior results (17), models incorporating anterograde, retrograde, and Euclidean distance-based spread had better fit with measured AT8+ aggregates than any model individually. These data are indicative, overall, of connectome driven spread of tau, which is further supported by our findings of high AT8+ aggregate levels in fiber tracts, particularly in PTZ treated 5X-TRAP mice. Of note, measured AT8+ aggregates in PTZ treated mice tended have worse fit across all models, except Euclidean spread, compared to their Sal treated counterparts. Worsened overall fit with standardized connectome-based spread models and improved fit with models of Euclidean distance-based spread in PTZ treated mice may be indicative of altered anatomical and functional long-range connectivity and enhanced local connectivity caused by seizures (48–51). We also found that measured AT8+ aggregates in 5X-TRAP mice, but not WT-TRAP, fit with anterograde models of spread. We also found lower relative contribution of Euclidean and retrograde models to spread in Sal and PTZ treated 5X-TRAP mice, respectively. When taken with our mapping data, these results suggest that increased tau spread in 5X-TRAP mice is, at least in part, due to increased trans-neuronal anterograde spread.

We found that tdT levels were predictive of tau spread in all groups except PTZ treated WT-TRAP mice. The lack of an effect in PTZ treated WT-TRAP mice was expected since there was increased tdT+ counts due to seizure induction without change in tau spread. The relationship between neuronal activity level and predictivity of tau spread in Sal treated WT-TRAP mice suggest that tau travels between basally active networks in the absence of hyperactivity. Similarly, tau has been shown to spread through the default mode network in human patients, which that are more metabolically active at rest (1, 4, 6). Our results also indicate that regional neuronal activity levels are predictive of relative tau levels in 5X-TRAP mice and further indicate that neuronal hyperactivity may drive increased tau spread in 5X-TRAP mice.

Another novel aspect of our study was the ability to label and track hyperactive neurons to determine their contribution to tau spread. We found that tdT+ neurons were more likely to colocalize with AT8+ aggregates than the surrounding tdT− neurons in the thalamus and striatum of both PTZ and Sal treated 5X-TRAP mice. Importantly, these are regions in which there were increased overall AT8+ aggregates and tdT+ cells in 5X-TRAP mice compared to WT-TRAP and an exacerbation of AT8+ aggregates in PTZ kindled 5X-TRAP mice, indicating that these hyperactive tdT+ neurons drive enhanced tau spread. tdT labeling occurs soon after tau injection (Fig 2A), before extensive tau spread (15). When taken with our prior data demonstrating that tdT-TRAP labelled hyperactive neurons show sustained hyperexcitability (36), suggest that tau is propagated by these hyperactive populations. Indeed, temporal lobe epilepsy is associated with increases in tau kinases (52), and may be involved in the increased somatic tau levels found in tdT+ neurons.

While proof of synaptic tau transmission *in vivo* remains elusive, there is strong evidence indicating synaptic spread as a key mechanism underlying its trans-neuronal propagation (20, 53). Supportive of this mechanism, we found increases in the synaptic vesicular protein, SYNGR3, which binds tau presynaptically, in PTZ kindled 5X-TRAP mice compared to Sal treated 5X-TRAP mice and in AD brains with a history of seizures compared to those without. Future studies should examine whether these synaptic changes occur specifically in hyperactive neurons and if reduction of SYNGR3 slows tau spread.

To expand our investigations of the role of neuronal hyperactivity in tau pathology distribution into human AD, we performed a retrospective analysis of postmortem tau pathological ratings. We found changes consistent with increased tau spread in AD patients with seizure history compared to those without, consistent with a clinical study demonstrating that the development of seizures in AD is associated with increased CSF tau levels (54). While clinical examination of the relationship between seizures and tau localization in the brain is limited, a recent study from Lam and colleagues (55) found associations between seizure foci and spatial development of both tau and amyloid pathology, additionally corroborating our results that neuronal activity levels were predictive of tau spread to that region. Follow-up studies should be performed to monitor AD patients for epileptiform activity or other electrographic biomarkers and track tau progression by PET.

Our data support neuronal hyperexcitability as a target to slow AD progression, especially early in the disease. Indeed, the antiseizure medication (ASM), levetiracetam, slowed cognitive decline in mild cognitive impairment (56) and AD patients with epileptiform activity (57), but both studies used short term treatments (≤ 4 weeks) and did not examine pathological progression. Based on our results, studies with long term ASM therapy and serial tau PET scans would be warranted to link seizure control with modulation of tau spread. Other drugs targeting hyperexcitability may also hold promise. Indeed, we have found that modulating excitability via inhibition of mTORC1 activity with rapamycin reduced Aβ and cognitive deficits in PTZ kindled 5XFAD mice (27).

**Figure 8.**
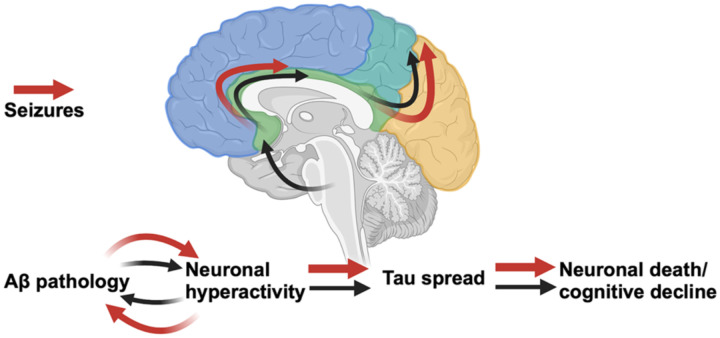
Schematic of proposed model for AD progression.

Together, these comprehensive studies indicate that in the presence of Aβ, neuronal hyperactivity facilitates anterograde tau spread through hyperactive networks and between hyperactive neurons with rates of tau spread associated with cognitive dysfunction. Thus, we contribute to a model of AD progression whereby amyloid-neuronal hyperactivity interactions facilitate the spread of tau, advancing disease progression and cognitive decline, a process which is exacerbated by seizures (Fig 8). Targeting neuronal hyperactivity, particularly at early, Aβ-predominant stages in AD, should be prioritized to slow overall disease progression.

## Methods and materials

### Mice.

All mouse procedures and protocols were approved by the University of Pennsylvania Institutional Animal Care and Use Committee (IACUC) Office of Animal Welfare. Fos^2A-iCreER^ (TRAP2) and B6.Cg-*Gt(ROSA)26Sor*^tm14(CAG-tdTomato)Hze^/J (Ai14) were obtained from Jackson Laboratory (stock 030323, 007914). TRAP2 and Ai14 mice were crossed for two generations, producing mice homozygous for Fos^2A-iCreER^ and B6.Cg-*Gt(ROSA)26Sor*^tm14(CAG-tdTomato)Hze^/J. 5XFAD on a mixed B6SJL background first generation (F1) generated from mice purchased from Jackson Laboratory (MMRRC strain #3840, Bar Harbor ME) were bred with mice homozygous for TRAP2/Ai14 to produce 5XFAD × TRAP (5X-TRAP) and littermate WT × TRAP (WT-TRAP) experimental mice that were used in all immunohistochemistry and behavioral experiments. For western blot experiments, we used male and female 5XFAD and littermate WT mice second generation (F2) from mice purchased from Jackson Laboratory (MMRRC strain #3840, Bar Harbor ME).

### AD-tau isolation.

AD-tau isolation was performed as previously established (13) from cortical tissue from patients confirmed to have AD by neuropathological diagnosis. Total protein concentrations were determined by BCA and tau, Aβ, and alpha synuclein concentrations were determined by ELISA: purified tau= 0.4–1.1 μg/μL, Aβ_42_ = 32–132 ng/mL, Aβ_40_ =3–46 ng/mL, and α synuclein= nondetectable – 0.54 μg/mL.

### Stereotaxic surgery.

AD-tau was injected into 3-month-old mice using previously established protocols (15). Briefly, mice were anesthetized with ketamine-xylazine-acepromazine and mounted in a stereotaxic apparatus. A hole was bore from 2.5 mm posterior from Bregma and 2.0 mm lateral (right) from midline. A Hamilton syringe was lowered 2.4 mm and 2.5 μL of 0.4 μg/μL (1 μg) AD-tau was injected into the hippocampus. The syringe was then retracted 1 mm (1.4 mm depth) and an additional 1 μg of AD-tau was injected into the cortex.

### PTZ kindling.

Two to three weeks following AD-tau injection, mice underwent PTZ kindling using previously established protocols (27, 58). Briefly, mice were injected (I.P.) every 48 hours for 15 days (8 injections) with 35 mg/kg PTZ (Sigma Aldrich, St Louis MO) or vehicle (0.9% saline, Sigma-Aldrich). Mice were monitored and video-recorded for one hour after PTZ administration and Racine scored for seizure severity. If 5XFAD/WT mice reached three consecutive days of stage 5 (tonic-clonic) seizures, they underwent one additional PTZ treatment and then removed from kindling.

### 4-OHT administration.

5X-TRAP and WT-TRAP mice were injected with 4-hydroxytamoxifen (50 mg/kg, I.P.) to induce permanent tdTomato expression in neurons that were activated (Fos-expressing) in the 4–6 hour window after administration. AD-tau seeded 5X-TRAP and WT-TRAP mice were injected with 4-OHT 30 min prior to on final PTZ or saline tdT expression in seizure activated (Fos+) neurons (36).

### Mouse behavior.

Behavioral experiments were carried out in the Neurobehavior Testing Core in the Translational Research Center at the University of Pennsylvania. Experimenters were blind to genotype and condition. Mice underwent a behavioral battery consisting of the open field assay, NOR, and CFC. Procedures took place from 5 – 6 months of age in all WT-TRAP and 5X-TRAP mice.

#### Open field assay.

Spontaneous locomotion and rearing activity were assessed in an open field arena (14” × 14” × 18”). The Photobeam Activity System (San Diego Instruments) was used to acquire data. The arena is fitted with a scaffold of IR emitters and detectors to collect peripheral, center and vertical (rearing) beam breaks. After a thirty-minute habituation to the testing room, a ten-minute trial began with a mouse placed in the center of the arena. All trials were recorded by high-definition camcorders. Digitally recorded trials were processed for automated analysis by ANYmaze software (Stoelting Co, Il) to obtain additional measures.

#### Novel object recognition.

The novel object recognition procedure consisted of habituation/pre-exposure, acquisition and recall trials. Before habituation, mice were handled daily for two minutes for three consecutive days. The habituation/pre-exposure phase consists of daily five-minute trials where the mouse is allowed to freely explore the empty NOR arena (approx. 1 square foot). During the acquisition trial, mice are returned to an arena now containing a pair of the same objects. The object pairs used are either glass bottles or metal bar (2”× 2” × five”) placed about three inches from the arena walls. Mice freely explore the object pairs for fifteen minutes. The arena and objects are cleaned with 70% EtOH between all trials. 24 hours after acquisition, mice returned to the arena, with one of the now-familiar objects replaced with a novel object (for example a bottle may be swapped with a metal bar or vice versa). During the recall trial, mice explore the two objects for 15 minutes. All trials are digitally recorded for automated off-line grading by ANYmaze software (Stoelting Co, Il). Time spent exploring (approaches and sniffing) and was determined as the primary dependent variable.

#### Contextual fear conditioning.

Mice were habituated to experimenter handling for three days prior to acquisition. For acquisition, mice were placed in a soundproof conditioning chamber (Med Associate, Fairfax, VT) for 4.5 minutes, during which time they received to unsignaled foot shocks (1 mA, 2s) at 1-minute intervals. For recall trials, mice were returned to the conditioning chamber for 5 minutes at 1 day (recent memory) and 14 days (remote memory) after acquisition, without any stimulation. Behavior was recorded during all trials and freezing behavior (lack of movement except for breathing) was assessed with FreezeScan software (Clever Systems, Reston, VA). Results from recall trials were analyzed relative to baseline freezing levels while the mice were in the recording chamber prior to foot shock during the acquisition trial to control for baseline differences in freezing behavior.

### Euthanasia.

Mice included for immunohistochemical analysis were anesthetized by I.P. injection of pentobarbital (50 mg/kg) (Sagent Pharmaceuticals, Schaumburg, IL) and underwent cardiac perfusion with ice cold, 4% paraformaldehyde. Mice used for western blots were instead perfused with cold PBS. The tissue was dissected and flash frozen.

### Immunohistochemistry.

Brains were incubated at 4°C for 24 h in 4% PFA, followed by sequential incubations in 10%, 20%, and 30% sucrose at 4°C. Samples were then cryoprotected in OCT Tissue Tek and sectioned at 20 um. Immunohistochemistry (IHC) was performed using standard, previously published protocols (27). Briefly, slices were permeabilized (0.02% triton-X100 in PBS) for 30 minutes, blocked for 1 hour (5% normal goat serum, 0.01% triton X-100, 1% bovine serum albumin), and incubated with primary antibody (see Table S7) in blocking solution overnight at 4°C and for one hour with secondary antibodies Alexafluor (Thermo Fisher Scientific, Waltham MA) at a concentration of 1:1000.

### Fluorojade-B.

Cryosectioned (20 μm) tissue was stained for fluorojade-B following manufacturer’s instructions (EMD Millipore, Burlington, MA). Briefly, slides were sequentially incubated in 80% ethanol for 5 min, 70% ethanol for 2 min, distilled water for 2 min, 0.06% KMnO4 for 15 min, distilled water for 2 min, 0.001% fluorojade-B solution for 20 min, and distilled water for 1 min (3 times). Images were acquired using a Nikon Eclipse 80i microscope and a digital Nikon DS-Fi2 camera (Micro Video Instruments, Avon MA) with a 20X objective. Fluorojade B+ cells were counted using Image J.

### Slide scanning and quantifications.

Fluorescent slide scanning for sectioned tissue was performed with a 3DHISTECH Lamina Scanner (Perkin Elmer, Waltham, MA) with a 20X objective. We used NeuroInfo software (MBF, Williston, VT) to register brain slices to the Allen Brain Atlas (ABA) and detect and map tau AT8+ aggregates and tdT+ and tdT−/NeuN+ neuronal populations. All AT8+ aggregates and tdT+ neurons were detected. Given that NeuN staining was inconsistent within samples, only tdT−/NeuN+ cells with sharp, defined edges were detected providing a representative sampling of tdT−/NeuN+ cells for each region. Data containing each detected object were exported from NeuroInfo and analyzed in R. Each object was summed through the ABA hierarchy (e. g. Somatosensory area and auditory area counts were added to the isocortex counts). Brain regions that contained imperfections (e.g. folds, hole in tissue) were excluded from analysis for that sample. Only brain regions with at least four samples per group were included for statistical analysis. Additionally, for cell type (tdT+/tdT−) colocalization with tau pathology, regions were excluded in samples that did not contain at least 8 tdT+ and 8 tdT− cells.

### Computational analysis.

As in prior work (59),we used linear diffusion models to predict the spatial distribution of tau pathology, ***x***(*t*), across *N* regions defined by the Allen Brain Atlas, using an *N* × *N* adjacency matrix ***A***, whose edge weights are the directed structural connectivity strength from region *i* to region *j*. Connectivity strength was defined by fluorescence intensity from retrograde viral tract tracing (44). We computed the estimated regional tau pathology $\hat{x}(t)$ using an equation of the form $\hat{x}(t)=e^{-cLt}x_{o},$ where *t* is 3 months post injection, ***x***_*o*_ is a vector with 1 unit of pathology in the hippocampal injection sites, ***L*** is the out-degree Laplacian of ***A***, and *c* is a free parameter obtained through model fitting. The hippocampal injection sites were the right dentate gyrus, CA1, CA3, and posterior parietal association area. We generated distributions of model fit, i.e. the spatial correlation between ***x***(*t*) and $\hat{x}(t)$, using bootstrapped samples of mice to compare models where ***A*** is defined by retrograde connectivity, anterograde connectivity, or interregional Euclidean distance. Finally, we used multivariate regression to construct a “Bidirectional Euclidean tdT” model which combined anterograde spread, retrograde spread, Euclidean distance-based spread, and tdT positive cell counts using the following form: $x(t)=\beta_{a}\hat{x}{\left(t\right)}_{a}+\beta_{r}\hat{x}{\left(t\right)}_{r}+\beta_{e}\hat{x}{\left(t\right)}_{e}+\beta_{T}T+\varepsilon$, where $\hat{x}{\left(t\right)}_{a}$ is predicted anterograde spread, $\hat{x}{\left(t\right)}_{r}$ is predicted retrograde spread, $\hat{x}{\left(t\right)}_{r}$ is spread based on Euclidean distance, *T* is a vector of tdT positive cell counts, and *ε* is an error term. Distributions of bootstrapped model fits or regression beta weights were compared using a two-tailed non-parametric test (60).

### Western blot.

Mouse hippocampi and human temporal lobe cortex were homogenized in lysis buffer (1.1% sucrose, 50 mM Tris HCl, 500 μM CaCl2, 1 mM NaHCO3, 1X protease inhibitor, 1mM phenylmethanesulfonyl fluoride, and 1X HALT^™^ (Thermo Fisher Scientific, Carlsbad, CA) on ice and centrifuged (4,100 g for 10 min at 4°C). Further centrifugation was performed (13,000 g for 10 min at 4°C) to isolate membrane fractions. Membrane fraction protein concentrations were calculated by Bradford Protein Assay (Bio-Rad, Hercules, CA). Western blots were performed using established protocols (27, 58). Gel electrophoresis with 4–20% SDS-PAGE gels (Bio-Rad) was performed to separate proteins based on molecular weight, followed by transfer to polyvinylidene difluoride membranes (EMD, Millipore, Burlington, MA). Membranes were incubated with primary antibodies overnight at 4°C and bands were imaged with an Odyssey Imaging System (LI-COR Biosciences, Lincoln, NE) and quantified with Image Lab software (Bio-Rad). Information for primary antibodies is listed in Table S7.

### Human Subjects.

All procedures and protocols were approved by the ethical standards of the Institutional Review Board (IRB) at the University of Pennsylvania. Subjects were diagnosed with AD based on clinical history, and neurological and neuropsychological assessment. Clinical diagnoses were confirmed post-mortem based on staging of Aβ_42_ and tau pathology. For assessment of tau pathology distribution in AD patients and control subjects, numeral ratings across 18 brain regions were retrieved from the Center for Neurodegenerative Disease Research (CNDR) database at the University of Pennsylvania. The frequency of tau pathology was scored by expert pathologists using a standard semi-quantitative scale: 0 (absent), 1 (sparse), 2 (moderate), and 3 (frequent). For western blot analysis, post-mortem AD and control superior/mid-temporal cortex frozen samples were acquired from CNDR repository. Clinical data from these patients are reported in Table S8.

### Statistics.

AT8+ and tdT+ mapping data were analyzed by 2-way ANOVA with genotype (WTTRAP vs 5X-TRAP) and kindling (PTZ vs Saline) as independent variables for each sampled brain region. Tukey’s post hoc identified group differences when main effects or interactions were found. A table containing results from every comparison made are included in Tables S1–S4. Behavior comparisons were made by 2-way ANOVA with genotype (WT-TRAP vs 5X-TRAP) and kindling (saline vs PTZ) as independent variables with Sidak’s post hoc to identify group differences when main effects or interactions were found. Correlations between regional AT8+/tdT+ counts and behavior were identified by Pearson correlation. For AT8+ aggregate colocalization experiments (Fig 3, S3), WT-TRAP and 5X-TRAP were analyzed separately by 2-way ANOVA with kindling (saline vs PTZ) and cell type (tdT+ vs tdT-) as independent variables for each sampled brain region with Tukey’s post hoc where main effects or interactions were found. Human pathological ratings were compared by Mann-Whitney test. For western blots, control to all AD and AD-Sz to AD+Sz comparisons were made by separate Student’s t-test. Males and females were included in all experiments. Sex was added as an independent variable for each dataset and analyzed by multiple or ordinal linear regressions. Results are included in Table S5 and S6.

## Supplementary Material

Supplement 1

Supplement 2

Supplement 3

Supplement 4

Supplement 5

## References

[1] Vogel J. W. , Spread of pathological tau proteins through communicating neurons in human Alzheimer’s disease. Nat Commun 11, 2612 (2020). 32457389 10.1038/s41467-020-15701-2 PMC7251068

[2] Schoonhoven D. N. , Tau protein spreads through functionally connected neurons in Alzheimer’s disease: a combined MEG/PET study. Brain 146, 4040–4054 (2023). 37279597 10.1093/brain/awad189 PMC10545627

[3] Zhou J., Gennatas E. D., Kramer J. H., Miller B. L., Seeley W. W., Predicting regional neurodegeneration from the healthy brain functional connectome. Neuron 73, 1216–1227 (2012). 22445348 10.1016/j.neuron.2012.03.004 PMC3361461

[4] Hoenig M. C. , Networks of tau distribution in Alzheimer’s disease. Brain 141, 568–581 (2018). 29315361 10.1093/brain/awx353

[5] Cope T. E. , Tau burden and the functional connectome in Alzheimer’s disease and progressive supranuclear palsy. Brain 141, 550–567 (2018). 29293892 10.1093/brain/awx347 PMC5837359

[6] Franzmeier N. , Functional connectivity associated with tau levels in ageing, Alzheimer’s, and small vessel disease. Brain 142, 1093–1107 (2019). 30770704 10.1093/brain/awz026 PMC6439332

[7] Bejanin A. , Tau pathology and neurodegeneration contribute to cognitive impairment in Alzheimer’s disease. Brain 140, 3286–3300 (2017). 29053874 10.1093/brain/awx243 PMC5841139

[8] Xia C. , Association of In Vivo [18F]AV-1451 Tau PET Imaging Results With Cortical Atrophy and Symptoms in Typical and Atypical Alzheimer Disease. JAMA Neurol 74, 427–436 (2017). 28241163 10.1001/jamaneurol.2016.5755 PMC5470368

[9] Arriagada P. V., Growdon J. H., Hedley-Whyte E. T., Hyman B. T., Neurofibrillary tangles but not senile plaques parallel duration and severity of Alzheimer’s disease. Neurology 42, 631–639 (1992). 1549228 10.1212/wnl.42.3.631

[10] Chen S. D. , Staging tau pathology with tau PET in Alzheimer’s disease: a longitudinal study. Transl Psychiatry 11, 483 (2021). 34537810 10.1038/s41398-021-01602-5 PMC8449785

[11] Braak H., Thal D. R., Ghebremedhin E., Del Tredici K., Stages of the pathologic process in Alzheimer disease: age categories from 1 to 100 years. J Neuropathol Exp Neurol 70, 960–969 (2011). 22002422 10.1097/NEN.0b013e318232a379

[12] Braak H., Alafuzoff I., Arzberger T., Kretzschmar H., Del Tredici K., Staging of Alzheimer disease-associated neurofibrillary pathology using paraffin sections and immunocytochemistry. Acta Neuropathol 112, 389–404 (2006). 16906426 10.1007/s00401-006-0127-z PMC3906709

[13] Guo J. L. , Unique pathological tau conformers from Alzheimer’s brains transmit tau pathology in nontransgenic mice. J Exp Med 213, 2635–2654 (2016). 27810929 10.1084/jem.20160833 PMC5110027

[14] Tok S. , Pathological and neurophysiological outcomes of seeding human-derived tau pathology in the APP-KI NL-G-F and NL-NL mouse models of Alzheimer’s Disease. Acta Neuropathol Commun 10, 92 (2022). 35739575 10.1186/s40478-022-01393-w PMC9219251

[15] He Z. , Amyloid-beta plaques enhance Alzheimer’s brain tau-seeded pathologies by facilitating neuritic plaque tau aggregation. Nat Med 24, 29–38 (2018). 29200205 10.1038/nm.4443 PMC5760353

[16] Vogel J. W. , Connectome-based modelling of neurodegenerative diseases: towards precision medicine and mechanistic insight. Nat Rev Neurosci 24, 620–639 (2023). 37620599 10.1038/s41583-023-00731-8

[17] Cornblath E. J. , Computational modeling of tau pathology spread reveals patterns of regional vulnerability and the impact of a genetic risk factor. Sci Adv 7, (2021). 10.1126/sciadv.abg6677 PMC8189700 34108219

[18] Seemiller J. , Indication of retrograde tau spreading along Braak stages and functional connectivity pathways. Eur J Nucl Med Mol Imaging 48, 2272–2282 (2021). 33462630 10.1007/s00259-020-05183-1 PMC8178161

[19] Franzmeier N. , Functional brain architecture is associated with the rate of tau accumulation in Alzheimer’s disease. Nat Commun 11, 347 (2020). 31953405 10.1038/s41467-019-14159-1 PMC6969065

[20] Wu J. W. , Neuronal activity enhances tau propagation and tau pathology in vivo. Nat Neurosci 19, 1085–1092 (2016). 27322420 10.1038/nn.4328 PMC4961585

[21] McInnes J. , Synaptogyrin-3 Mediates Presynaptic Dysfunction Induced by Tau. Neuron 97, 823–835 e828 (2018). 29398363 10.1016/j.neuron.2018.01.022

[22] Largo-Barrientos P. , Lowering Synaptogyrin-3 expression rescues Tau-induced memory defects and synaptic loss in the presence of microglial activation. Neuron 109, 767–777 e765 (2021). 33472038 10.1016/j.neuron.2020.12.016 PMC7927913

[23] Rauch J. N. , LRP1 is a master regulator of tau uptake and spread. Nature 580, 381–385 (2020). 32296178 10.1038/s41586-020-2156-5 PMC7687380

[24] Tok S., Ahnaou A., Drinkenburg W., Functional Neurophysiological Biomarkers of Early-Stage Alzheimer’s Disease: A Perspective of Network Hyperexcitability in Disease Progression. J Alzheimers Dis 88, 809–836 (2022). 34420957 10.3233/JAD-210397 PMC9484128

[25] Vossel K. A., Tartaglia M. C., Nygaard H. B., Zeman A. Z., Miller B. L., Epileptic activity in Alzheimer’s disease: causes and clinical relevance. Lancet Neurol 16, 311–322 (2017). 28327340 10.1016/S1474-4422(17)30044-3 PMC5973551

[26] Barbour A. J. , Seizures exacerbate excitatory: inhibitory imbalance in Alzheimer’s disease and 5XFAD mice. Brain, (2024). 10.1093/brain/awae126 PMC11146435 38662500

[27] Gourmaud S. , The role of mTORC1 activation in seizure-induced exacerbation of Alzheimer’s disease. Brain 145, 324–339 (2022). 34264340 10.1093/brain/awab268 PMC9126019

[28] Horvath A. A. , Subclinical epileptiform activity accelerates the progression of Alzheimer’s disease: A long-term EEG study. Clin Neurophysiol 132, 1982–1989 (2021). 34034963 10.1016/j.clinph.2021.03.050

[29] Baker J., Libretto T., Henley W., Zeman A., A Longitudinal Study of Epileptic Seizures in Alzheimer’s Disease. Front Neurol 10, 1266 (2019). 31866927 10.3389/fneur.2019.01266 PMC6904279

[30] Voglein J. , Seizures in Alzheimer’s disease are highly recurrent and associated with a poor disease course. J Neurol 267, 2941–2948 (2020). 32488295 10.1007/s00415-020-09937-7 PMC7501095

[31] Siskova Z. , Dendritic structural degeneration is functionally linked to cellular hyperexcitability in a mouse model of Alzheimer’s disease. Neuron 84, 1023–1033 (2014). 25456500 10.1016/j.neuron.2014.10.024

[32] Busche M. A. , Clusters of hyperactive neurons near amyloid plaques in a mouse model of Alzheimer’s disease. Science 321, 1686–1689 (2008). 18802001 10.1126/science.1162844

[33] Zott B. , A vicious cycle of beta amyloid-dependent neuronal hyperactivation. Science 365, 559–565 (2019). 31395777 10.1126/science.aay0198 PMC6690382

[34] Allen W. E. , Thirst-associated preoptic neurons encode an aversive motivational drive. Science 357, 1149–1155 (2017). 28912243 10.1126/science.aan6747 PMC5723384

[35] DeNardo L. A. , Temporal evolution of cortical ensembles promoting remote memory retrieval. Nat Neurosci 22, 460–469 (2019). 30692687 10.1038/s41593-018-0318-7 PMC6387639

[36] Xing B. , Reversible synaptic adaptations in a subpopulation of murine hippocampal neurons following early-life seizures. J Clin Invest, (2024). 10.1172/JCI175167 PMC10904056 38227384

[37] Naik A. A. , Mechanism of seizure-induced retrograde amnesia. Prog Neurobiol 200, 101984 (2021). 33388373 10.1016/j.pneurobio.2020.101984 PMC8026600

[38] Naik A. A., Brodovskaya A., Subedi S., Akram A., Kapur J., Extrahippocampal seizure and memory circuits overlap. eNeuro 9, (2022). 10.1523/ENEURO.0179-22.2022 PMC9319425 35853724

[39] Brevard M. E., Kulkarni P., King J. A., Ferris C. F., Imaging the neural substrates involved in the genesis of pentylenetetrazol-induced seizures. Epilepsia 47, 745–754 (2006). 16650141 10.1111/j.1528-1167.2006.00502.x

[40] Coulter D. A., Huguenard J. R., Prince D. A., Differential effects of petit mal anticonvulsants and convulsants on thalamic neurones: calcium current reduction. Br J Pharmacol 100, 800–806 (1990). 2169941 10.1111/j.1476-5381.1990.tb14095.x PMC1917607

[41] Sherman S. M., Thalamus plays a central role in ongoing cortical functioning. Nat Neurosci 19, 533–541 (2016). 27021938 10.1038/nn.4269

[42] Groenewegen H. J., Berendse H. W., The specificity of the ‘nonspecific’ midline and intralaminar thalamic nuclei. Trends Neurosci 17, 52–57 (1994). 7512768 10.1016/0166-2236(94)90074-4

[43] Smith S., Hopp S. C., The 5XFAD mouse model of Alzheimer’s disease displays age-dependent deficits in habituation to a novel environment. Aging Brain 3, 100078 (2023). 37333676 10.1016/j.nbas.2023.100078 PMC10275951

[44] Oh S. W. , A mesoscale connectome of the mouse brain. Nature 508, 207–214 (2014). 24695228 10.1038/nature13186 PMC5102064

[45] Korzhova V. , Long-term dynamics of aberrant neuronal activity in awake Alzheimer’s disease transgenic mice. Commun Biol 4, 1368 (2021). 34876653 10.1038/s42003-021-02884-7 PMC8651654

[46] Shokouhi S. , In vivo network models identify sex differences in the spread of tau pathology across the brain. Alzheimers Dement (Amst) 12, e12016 (2020). 32280740 10.1002/dad2.12016 PMC7144772

[47] Deleyto-Seldas N., Efeyan A., The mTOR-Autophagy Axis and the Control of Metabolism. Front Cell Dev Biol 9, 655731 (2021). 34277603 10.3389/fcell.2021.655731 PMC8281972

[48] Lin H. , Altered White Matter Structural Network in Frontal and Temporal Lobe Epilepsy: A Graph-Theoretical Study. Front Neurol 11, 561 (2020). 32625164 10.3389/fneur.2020.00561 PMC7311567

[49] Kreilkamp B. A. K. , Altered structural connectome in non-lesional newly diagnosed focal epilepsy: Relation to pharmacoresistance. Neuroimage Clin 29, 102564 (2021). 33508622 10.1016/j.nicl.2021.102564 PMC7841400

[50] Zhang Z. , Altered functional-structural coupling of large-scale brain networks in idiopathic generalized epilepsy. Brain 134, 2912–2928 (2011). 21975588 10.1093/brain/awr223

[51] Liang Y. , Altered Functional Connectivity after Epileptic Seizure Revealed by Scalp EEG. Neural Plast 2020, 8851415 (2020). 33299398 10.1155/2020/8851415 PMC7710419

[52] Gourmaud S. , Alzheimer-like amyloid and tau alterations associated with cognitive deficit in temporal lobe epilepsy. Brain 143, 191–209 (2020). 31834353 10.1093/brain/awz381 PMC6935754

[53] Colom-Cadena M. , Synaptic oligomeric tau in Alzheimer’s disease - A potential culprit in the spread of tau pathology through the brain. Neuron 111, 2170–2183 e2176 (2023). 37192625 10.1016/j.neuron.2023.04.020

[54] Tabuas-Pereira M. , Increased CSF tau is associated with a higher risk of seizures in patients with Alzheimer’s disease. Epilepsy Behav 98, 207–209 (2019). 31382178 10.1016/j.yebeh.2019.06.033

[55] Lam A. D. , Association of Seizure Foci and Location of Tau and Amyloid Deposition and Brain Atrophy in Patients With Alzheimer Disease and Seizures. Neurology 103, e209920 (2024). 39331846 10.1212/WNL.0000000000209920 PMC11441794

[56] Bakker A. , Reduction of hippocampal hyperactivity improves cognition in amnestic mild cognitive impairment. Neuron 74, 467–474 (2012). 22578498 10.1016/j.neuron.2012.03.023 PMC3351697

[57] Vossel K. , Effect of Levetiracetam on Cognition in Patients With Alzheimer Disease With and Without Epileptiform Activity: A Randomized Clinical Trial. JAMA Neurol 78, 1345–1354 (2021). 34570177 10.1001/jamaneurol.2021.3310 PMC8477304

[58] Barbour A. J. , Seizures exacerbate excitatory: inhibitory imbalance in Alzheimer’s disease and 5XFAD mice. Brain 147, 2169–2184 (2024). 38662500 10.1093/brain/awae126 PMC11146435

[59] Henderson M. X. , Spread of alpha-synuclein pathology through the brain connectome is modulated by selective vulnerability and predicted by network analysis. Nat Neurosci 22, 1248–1257 (2019). 31346295 10.1038/s41593-019-0457-5 PMC6662627

[60] Fox J.. (2002). Bootstrapping Regression Models Appendix to An R and S-PLUS Companion to Applied Regression.

