# Supplementary material for: Hyperactive neuronal networks enhance tau spread in an Alzheimer’s disease mouse model
Source: bioRxiv. 2025 Sep 18:2024.12.01.625514. Preprint. [Version 3] doi: 10.1101/2024.12.01.625514 (PMC11642807; doi:10.1101/2024.12.01.625514)
Supplement: Supplement 1 [file media-1.pdf]

Supplemental material

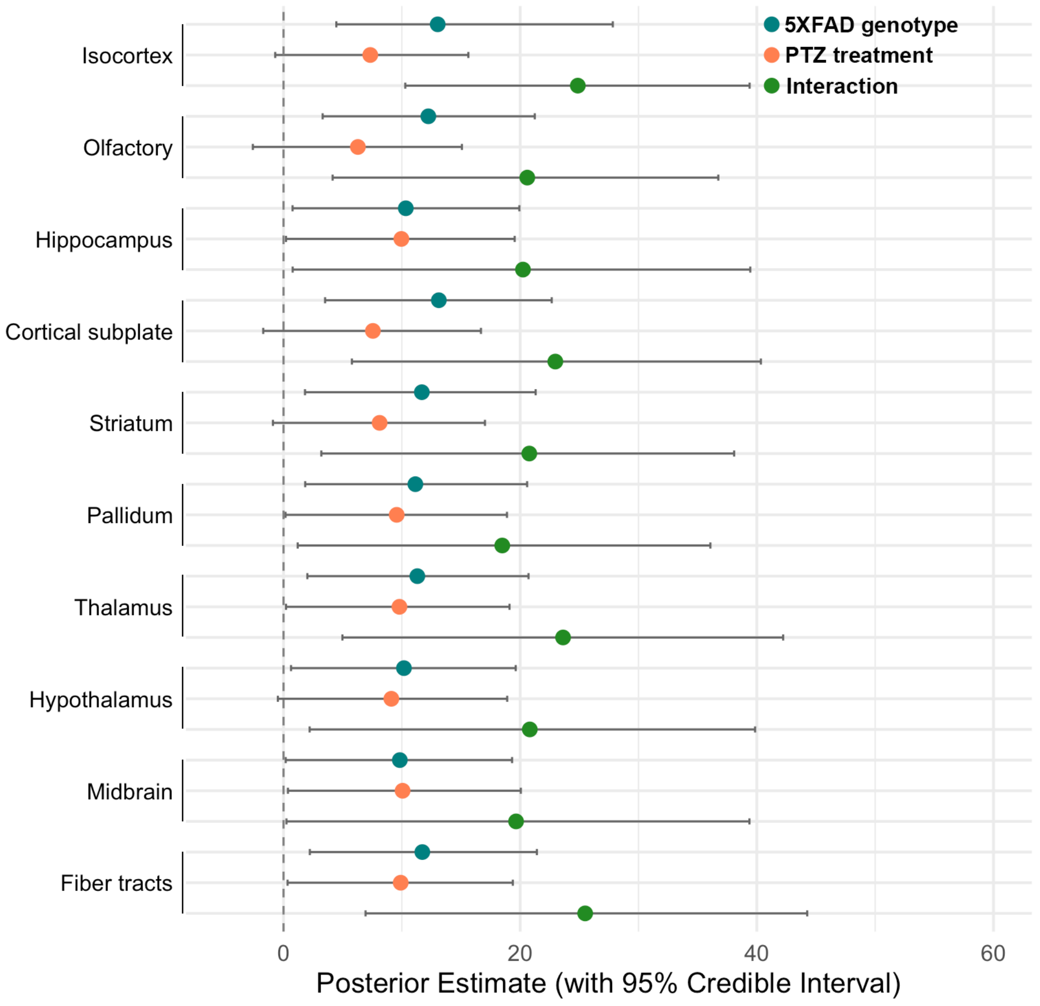

Figure S1. Hierarchical Bayesian models demonstrate interactions between PTZ treatment and 5XFAD genotype increase tau pathology in 10 major brain structures. Posterior means (points) and 95% credible intervals (bars) were estimated using Bayesian models for AT8+ levels including genotype, treatment, and their interactions as fixed effects, and animal and the region (nested within parent structure) as random effects. Each point represents the estimated effect on tau pathology (AT8+/mm^2) within the given brain region. Effects with 95% credible intervals that do not cross zero are considered to have credible

differences. Interactions between 5XFAD genotype and PTZ kindling credibly elevated AT8+ levels in all 10 major brain structures examined.

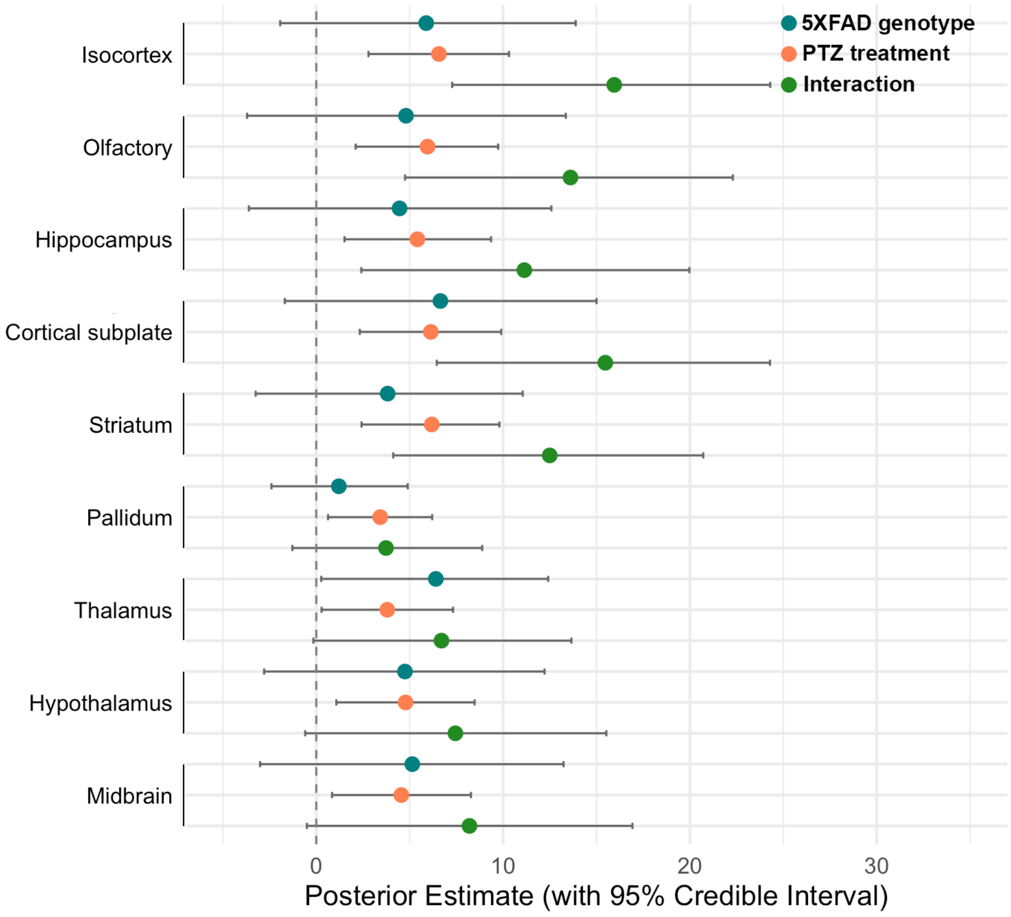

Figure S2. Hierarchical Bayesian models demonstrate that PTZ and its interactions with 5XFAD genotype increase neuronal activity in 9 major brain structures. Posterior means (points) and 95% credible intervals (bars) were estimated using Bayesian models for tdT+ counts including genotype, treatment, and their interactions as fixed effects, and animal and the region (nested within parent structure) as random effects. Each point represents the estimated effect on tdT+ counts (tdT+/mm^2) within the given brain region. Effects with 95% credible intervals that do not cross zero are considered to have credible differences. PTZ kindling credibly elevated tdT+ cells across all 10 parent brain regions and interacts with 5XFAD genotype to increase tdT+ cells in the isocortex, olfactory areas, hippocampus, cortical subplate, and striatum.

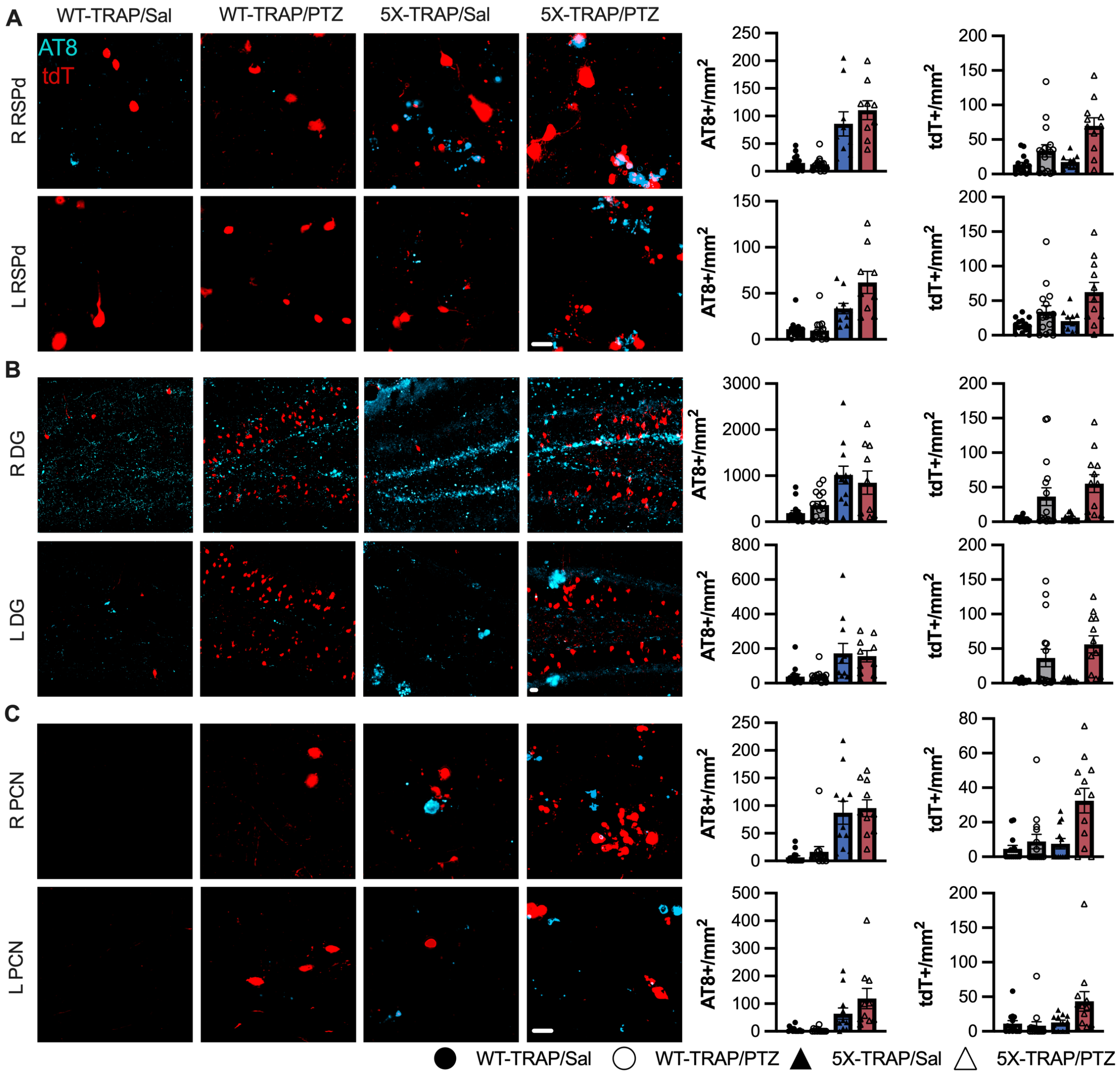

Figure S3. Representative staining and histograms from AT8 and tdT mapping.

Representative images and histograms from AT8 and tdTomato (tdT) brain mapping (Figure 1, Table S1, S2). AT8 IHC (cyan) and tdT (red) from all experimental groups in the ipsilateral (R) and contralateral (L) (to tau injection site) (**A**) dorsal retrosplenial cortex (RSPd), (**B**) dentate gyrus (DG), and (**C**) paracentral nucleus of the thalamus. N=9-16/group/brain region. Scale bars = 20 μm.

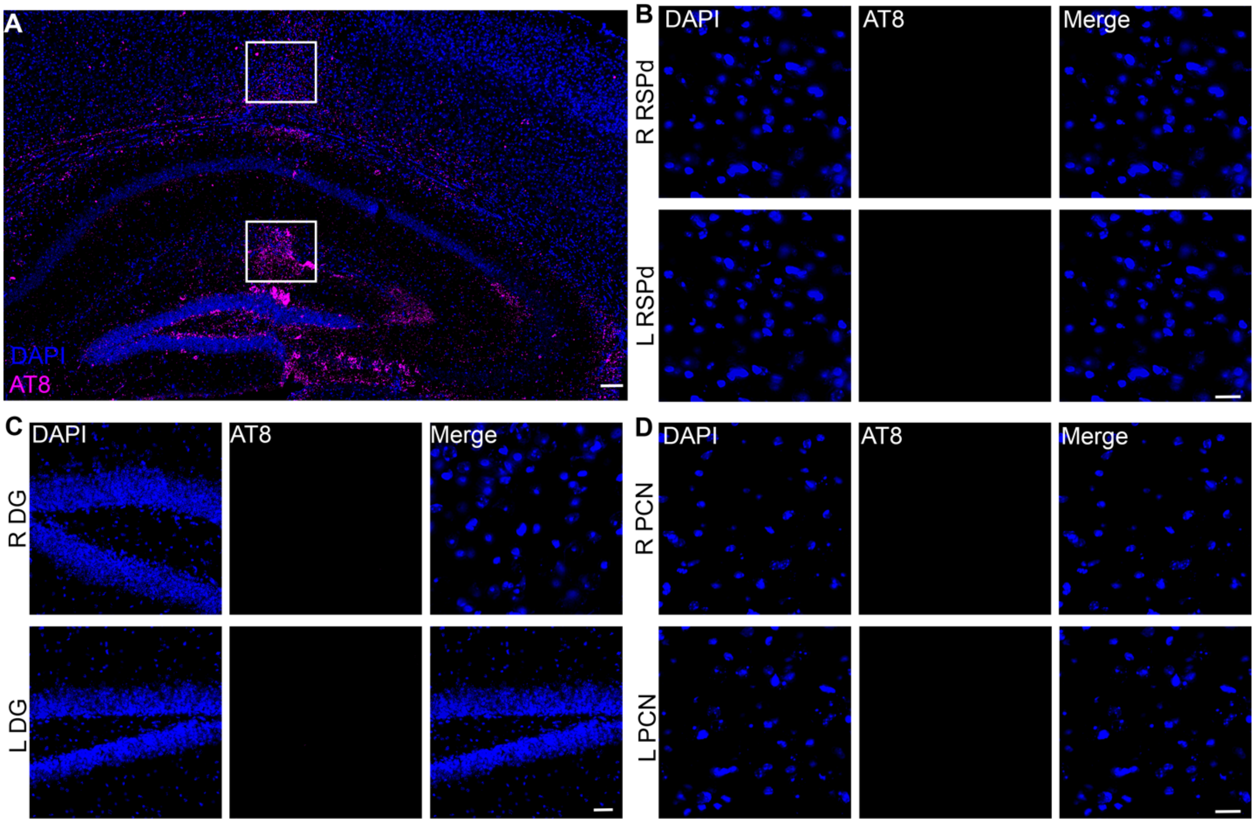

Figure S4. Validation of AD-tau injection site and AT8 specificity. (**A**) Representative AT8 (magenta) and DAPI (blue) image showing AD-tau injection sites (boxes) into the dorsal hippocampus and overlying cortex. Representative images of control sections omitting the primary antibody in the ipsilateral (R) and contralateral (L) (to AD-tau injection) (**B**) dorsal retrosplenial cortex (RSPd), (**C**) dentate gyrus (DG), and (**D**) paracentral nuclei of the thalamus from AD-tau seeded and slide scanned sections. Scale bars = 100 μm (**A**), 20 μm (**B, D**), 50 μm (**C**).

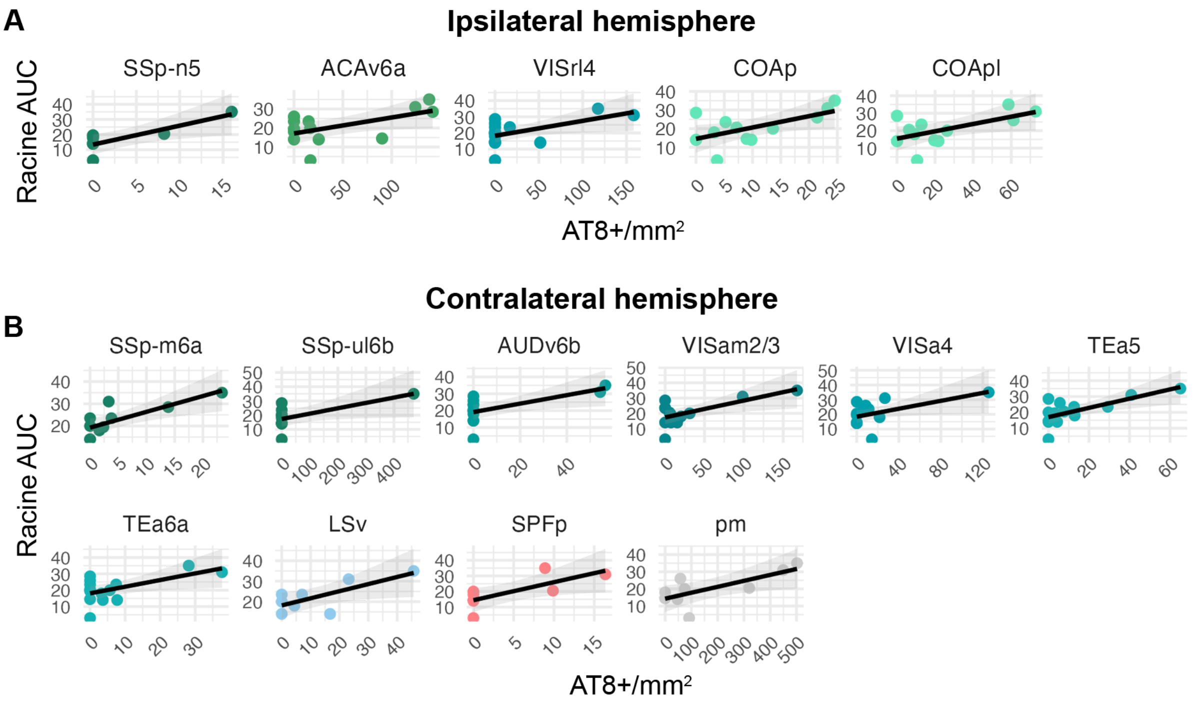

Figure S5. Seizure severity is correlated with tau spread in WT-TRAP mice. Pearson correlations were performed between area under the curve (AUC) for Racine score in the 15 min following the final PTZ administration and regional AT8+ immunoreactive aggregate levels in WT-TRAP mice. We found significant positive correlations in the (**A**) ipsilateral (to AD-tau injection) (right) isocortex: primary somatosensory area, nose, layer 5 (SSp-n5, r=0.82, p=0.019, n=6), anterior cingulate area, ventral part, layer 6a (ACAv6a, r=0.57, p=0.044, n=12), rostrolateral area, layer 4 (VISrl4, r=0.57, p=0.028, n=13), and cortical suplate: cortical amygdalar area posterior part (COAp, r=0.61, p=0.037, n=11). (**B**) Significant positive correlations were found in contralateral (left) isocortex: SSP, mouth, layer 6a (SSP-m6a, r=0.78, p=0.0076, n=9), SSp, upper limb, layer 6b (SSP-ul6b, r=0.64, p=0.048, n=9), ventral auditory area, layer 6b (AUDv6b, r=0.63, p=0.021, n=10), anteromedial visual area, layer 2/3 (VISam2/3, r=0.66, p=0.015, n=12), anterior area, layer 4 (VISa4, r=0.54, p=0.040, n=12), temporal association area, layer 5 (TEa5, r=0.69, p=0.0064, n=14) and 6a (TEa6a, r=0.58, p=0.030, n=14), striatum: lateral septal nucleus, ventral part (LSv, r=0.74, p=0.037, n=8), thalamus: subparafascicular nucleus, parvicellular part (SPFp, r=0.74, p=0.035, n=8), and fiber tracts:

primary mammillary tract (pm, r=0.71, p=0.032, n=9). Shaded areas display 95% confidence interval.

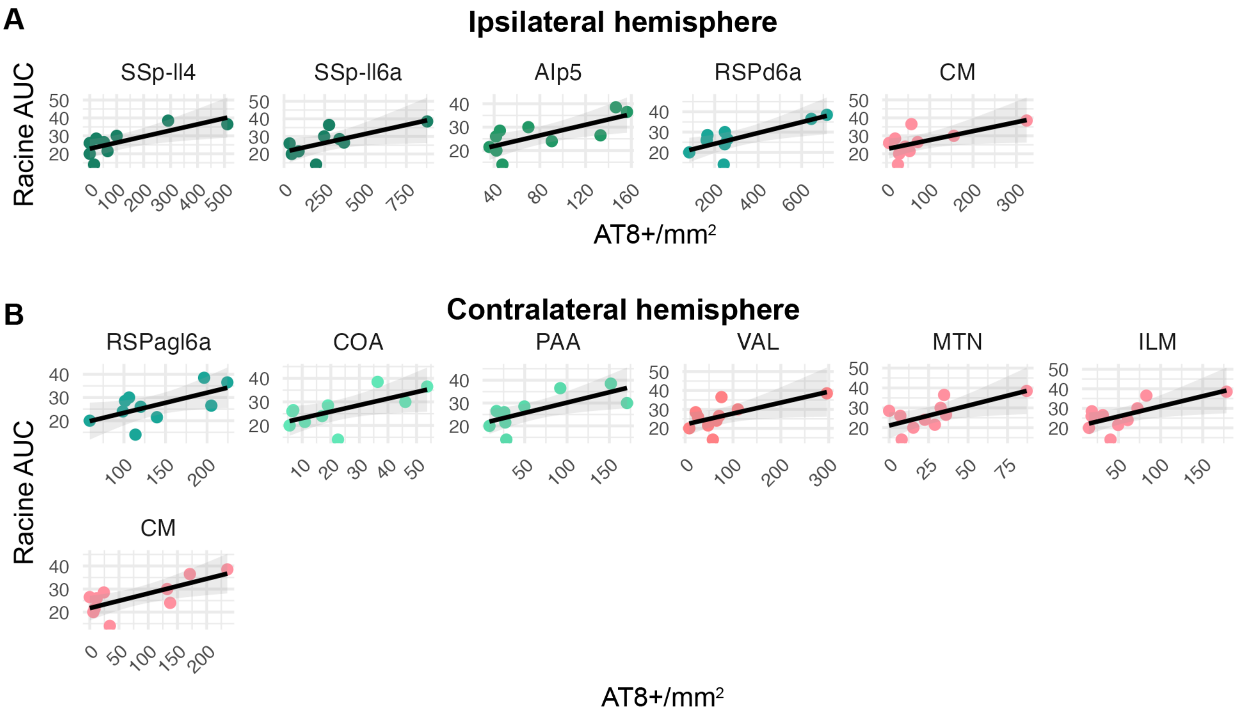

Figure S6. Seizure severity is correlated with tau spread in 5X-TRAP mice. Pearson correlations were performed between area under the curve (AUC) for Racine score in the 15 min following the final PTZ administration and regional AT8+ immunoreactive aggregate levels in 5X-TRAP mice. We found significant positive correlations in the (**A**) ipsilateral (to AD-tau injection) (right) isocortex: primary somatosensory area, lower limb layer 4 (SSp-ll4, r=0.76, p=0.041, n=9) and layer 6a (SSp-ll6a, r=0.68, p=0.046, n=9) agranular insular area, posterior part layer 5 (AIp5, r=0.73, p=0.015, n=11), and dorsal retrosplenial area layer 6a (RSPd6a, r=0.77, p=0.015, n=9), and the thalamus: central medial nucleus (CM, r=0.65, p=0.043, n=9). (**B**) Significant positive correlations were found in the contralateral (left) isocortex: retrosplenial area, lateral agranular part, layer 6a (RSPagl6a, r=0.64, p=0.047, n=10), olfactory areas: cortical amygdalar area (COA, r=0.65, p=0.043, n=9), piriform amygdalar area (PAA, r=0.72,

p=0.019, n=9), and thalamus: ventral anterior-lateral complex (VAL, r=0.64, p=0.045, n=10), midline group of the dorsal thalamus (MTN, r=0.67, p=0.035, n=10), intralaminar nuclei of the dorsal thalamus (r=0.69, p=0.029, n=10), and CM (r=0.73, p=0.014, n=9). Shaded areas display 95% confidence interval.

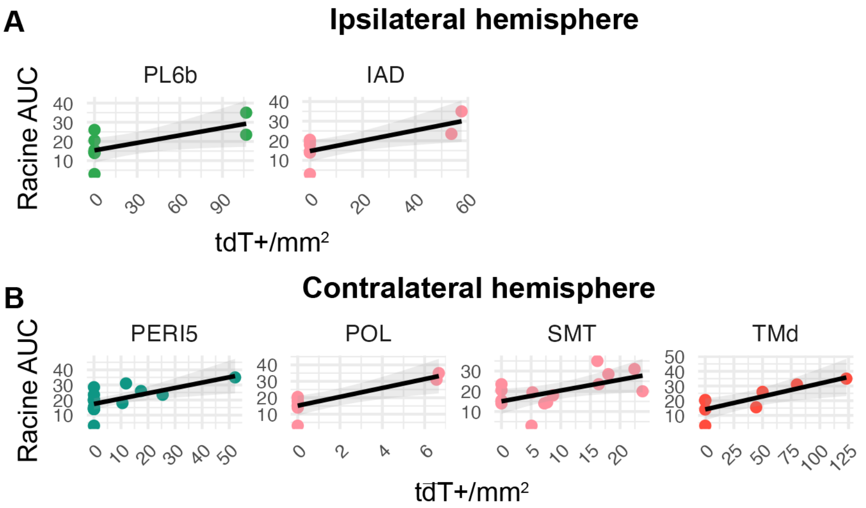

Figure S7. Seizure severity is correlated with activity dependent tdTomato labeling in WT-TRAP mice. Pearson correlations were performed between area under the curve (AUC) for Racine score in the 15 min following the final PTZ administration and regional tdT+ counts in WT-TRAP mice. (**A**) We found significant positive correlations in the ipsilateral (to AD-tau injection) (right) isocortex: prelimbic area layer 6b (PL6b,) and thalamus: interanterodorsal nucleus (IAD, r=0.75, p=0.020, n=8). (**B**) Significant positive correlations were found in the contralateral (left) isocortex: perirhinal area, layer 5 (PERI5, r=0.64, p=0.0075, n=13), thalamus: posterior limiting nucleus (POL, r=0.83, p=0.011, n=8), submedial nucleus (SMT, r=0.56, p=0.038, n=13) and hypothalamus: tuberomammillary nucleus, dorsal part (TMd, r=0.80, p=0.017, n=8). Shaded areas display 95% confidence interval.

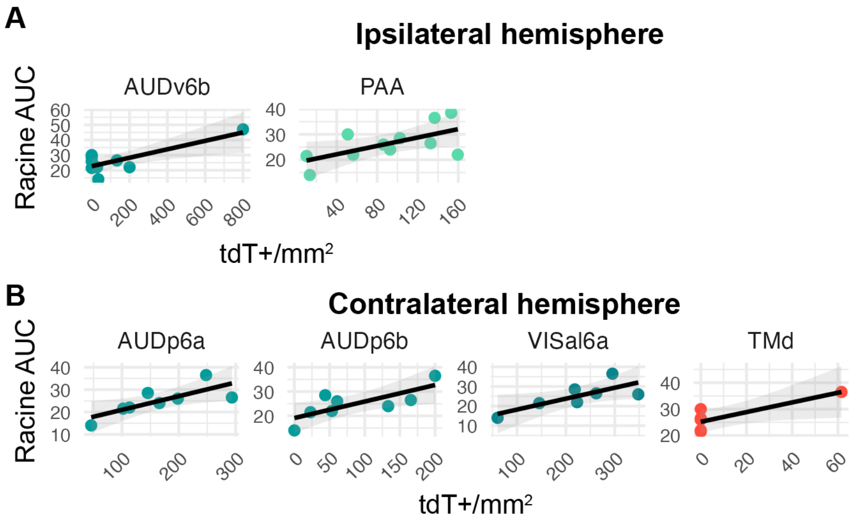

Figure S8. Seizure severity is correlated with activity dependent tdTomato labeling in 5X-TRAP mice. Pearson correlations were performed between area under the curve (AUC) for Racine score in the 15 min following the final PTZ administration and regional tdT+ counts in WT-TRAP mice. (**A**) We found significant positive correlations in the ipsilateral (to AD-tau injection) (right) isocortex: ventral auditory area, layer 6b (AUDv6b, r=0.80, p=0.0098, n=9), and olfactory areas: piriform amygdalar area (PAA, r=0.62, p=0.040, n=11). (**B**) Significant positive correlations were found in the contralateral (left) isocortex: primary auditory area layer 6a (AUDp6a, r=0.77, p=0.026, n=8) and 6b (AUDp6b, r=0.76, p=0.029, n=8), and anterolateral visual area layer 6b (VISal6a, r=0.77, p=0.44, n=7), and hypothalamus: tuberomammilary nucleus, dorsal part (TMd, r=0.83, p=0.42, n=6). Shaded areas display 95% confidence interval.

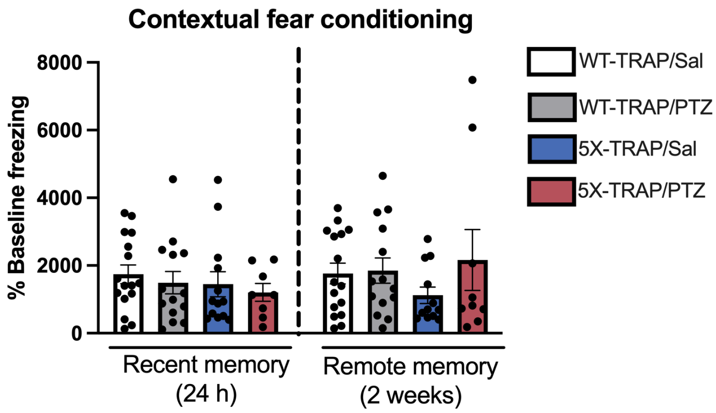

Figure S9. Results from contextual fear conditioning. Results are displayed as % change from baseline freezing levels to control for baseline differences in motor activity. Recent (1 day after conditioning) and remote (2 weeks after conditioning) recall were analyzed separately by 2-way ANOVA with genotype (WT-TRAP vs 5X-TRAP) and kindling (PTZ vs Sal) as independent variables. No significant effects were found. n=9-16/group. Histograms display group mean ± SEM.

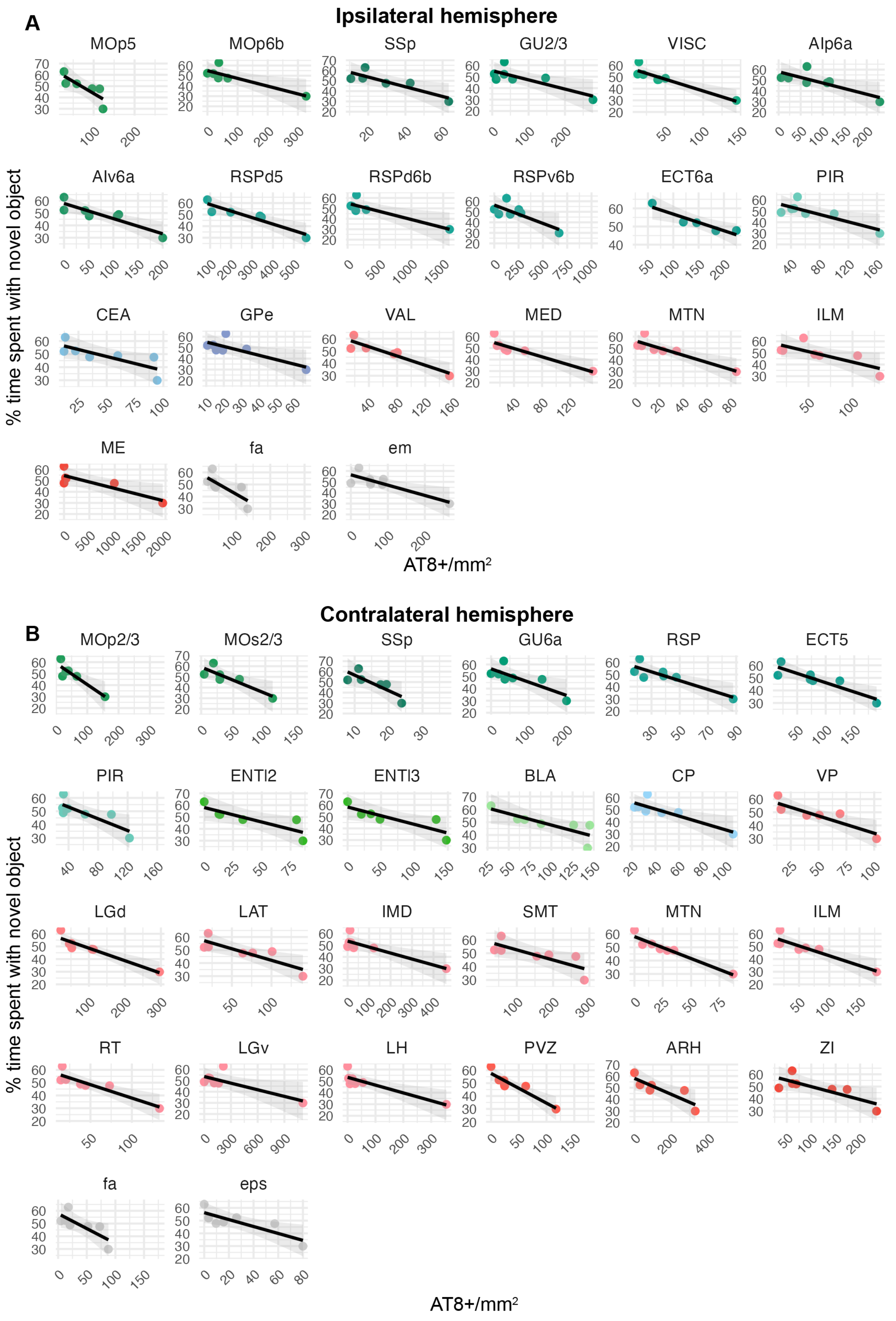
A
Ipsilateral hemisphere
MOp5
MOp6b
SSp
GU2/3
VISC
AIp6a
AIv6a
RSPd5
RSPd6b
RSPv6b
ECT6a
PIR
CEA
GPe
VAL
MED
MTN
ILM
ME
fa
em
% time spent with novel object
AT8+/mm²
B
Contralateral hemisphere
MOp2/3
MOs2/3
SSp
GU6a
RSP
ECT5
PIR
ENTl2
ENTl3
BLA
CP
VP
LGd
LAT
IMD
SMT
MTN
ILM
RT
LGv
LH
PVZ
ARH
ZI
fa
eps
% time spent with novel object
AT8+/mm²

Figure S10. Memory deficits are correlated with tau spread in 5X-TRAP mice. Pearson correlations were performed between % time spent with novel object during the novel object recognition (NOR) task and regional AT8+ levels in 5X-TRAP mice. (**A**) We found significant negative correlations in the ipsilateral (to AD-tau injection) (right) isocortex: primary motor area, layers 5 (MOp5, r=-0.82, p=0.044, n=6),and 6b (MOp6b, r=-0.86, p=0.027, n=6) primary somatosensory area, overall (SSp, r=-0.87, p=0.027, n=6), gustatory area, layer 2/3 (GU2/3, r=-0.81, p=0.029, n=7) visceral area, overall, (VISC, r=-0.93, p=0.0028, n=7), posterior and ventral agranular insular area, layer 6a (AIp6a, r=-0.79, p=0.031, n=7) (AIv6a, r=-0.90, p=0.0061, n=7), dorsal retrosplenial area, layers 5 and 6b (RSPd5, r=-0.93, p=0.0068, n=7) (RSPd6b, r=-0.87, p=0.022, n=7), ectorhinal area, layer 6a (ECT6a, r=-93, p=0.020, n=6), olfactory area: piriform area (PIR, r=-0.81, p=0.027, n=7), striatum: central amygdalar nucleus (CEA, r=-0.77, p=0.042, n=7), pallidum: external globus pallidus (GPe, r=-0.80, p=0.032, n=7), thalamus: ventral anterior lateral complex (VAL, r=-0.93, p=0.0030, n=7), medial group of the dorsal thalamus (MED, r=-0.91, p=0.0034, n=7), midline group of the dorsal thalamus (MTN, r=-0.90, p=0.0057, n=7), intralaminar nuclei of the dorsal thalamus (ILM, r=-0.76, p=0.045, n=7), hypothalamus: median eminence (ME, r=-0.86, p=0.026, n=6), and fiber tracts: corpus callosum anterior forceps (fa, r=-0.76, p=0047, n=7), external medullary lamina of the thalamus (em, r=-0.85, p=0.016, n=7). (**B**) Significant negative correlations were found in contralateral (left) isocortex: MOP2/3 (r=-0.91, p=0.012, n=6) secondary motor cortex layers 2/3 (MOs2/3, r=-0.88, p=0.022, n=6), SSp r=-0.82, p=0.047, n=6), GU6a (r=-0.81, p=0.028, n=6), RSP, overall (r=-0.88, p=0.0091, n=7)), ECT5 (r=-0.90, p=0.006, n=7), PIR (r=-0.82, p=0.023, n=7), entorhinal area, lateral part layer 2 and 4 (ENTl2, r=-0.83, p=0.039, n=6) (ENTl3, r=-0.85, p=0.034, n=6), cortical subplate: basolateral amygdala (BLA, r=-0.80, p=0.029, n=7), striatum: caudoputamen (CP, r=-0.85, p=0.013, n=7), thalamus: ventral posterior complex (VP, r=-0.88, p=0.0087, 6), dorsal lateral geniculate complex (LGd, r=-0.93, p=0.0030) lateral group of the dorsal thalamus (LAT, r=-0.85, p=0.015, n=6), intermediodorsal nucleus (IMD, r=-0.87, p=0.011, n=7), submedial nucleus (SMT, r=-0.89,

p=.037, n=7), MTN (r=-0.97, p=0.00050, n=7), ILM (r=-0.91, p=0.0038, n=7), reticular nucleus (RT, r=-0.91, p=0.0043, n=7), ventral lateral geniculate complex (LGv, r=-0.78, p=0.039, n=7), lateral habenula (LH, r=-0.88, p=0.0091, n=7), hypothalamus: periventricular zone (PVZ, r=-0.93, p=0.0024, n=7), arcuate hypothalamic nucleus (ARH, r=-0.86, p=0.029, n=6), zona incerta (ZI, r=-0.79, p=0.034, n=7), and fiber tracts: fa (r=-0.77, p=0.042, n=7), extrapyramidal fiber system (eps, r=-0.83, p=0.020, n=7). Shaded areas display 95% confidence interval.

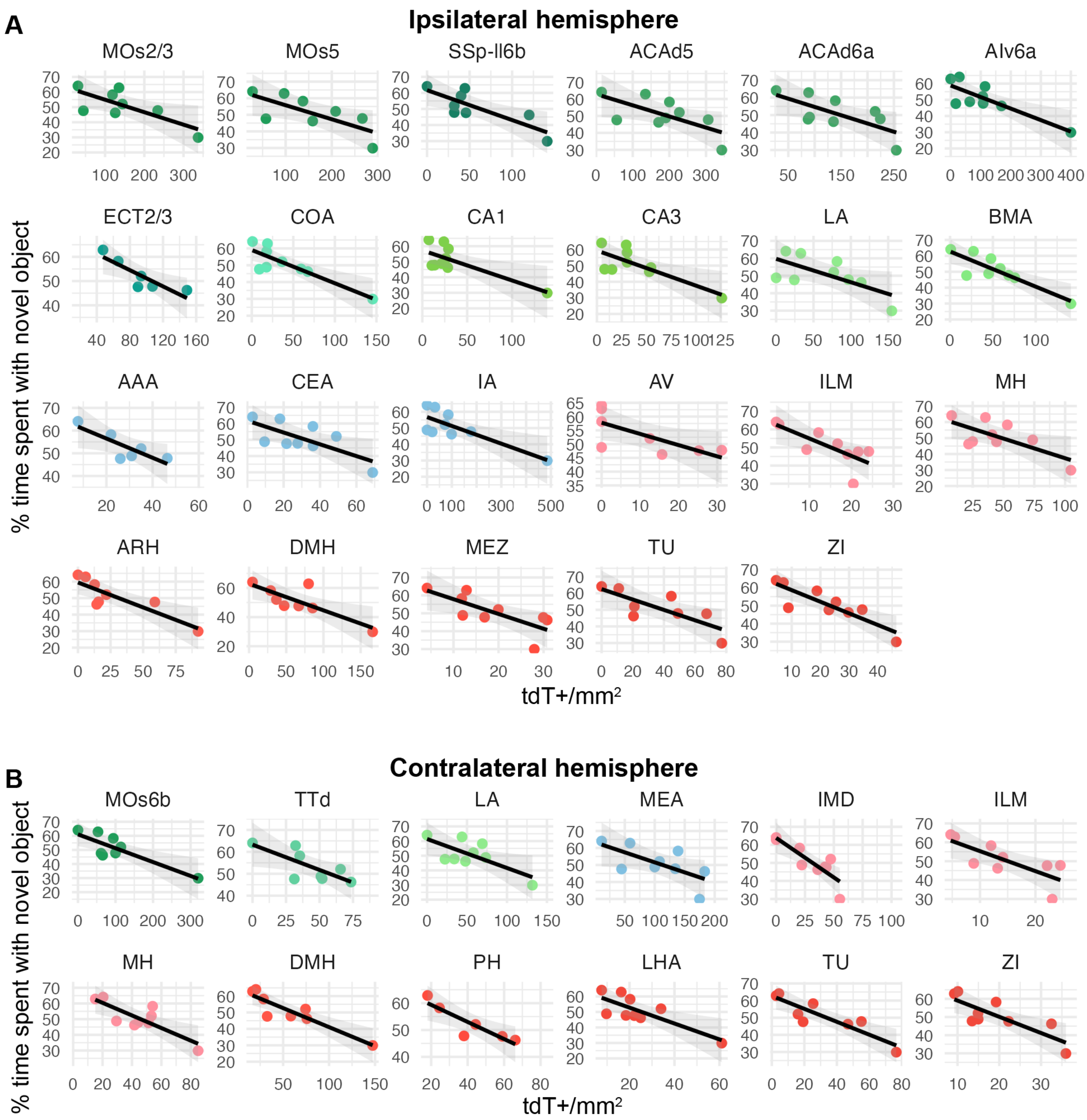

Figure S11. Memory deficits are correlated with activity dependent tdTomato labeling in 5X-TRAP mice. Pearson correlations were performed between % time spent with novel object during the novel object recognition (NOR) task and regional tdT+ counts in 5X-TRAP mice. We found significant negative correlations in the ipsilateral (to AD-tau injection) (right) (**A**) isocortex:

secondary motor cortex layers 2/3 (MOs2/3, r=-0.74, p=0.035, n=8) and 5 (MOs5, r=-0.72, p=0.043, n=8), primary somatosensory area, lower limb, layer 6b (SSp-ll6b, r=-0.81, p=0.015, n=8), dorsal anterior cingulate area layer 5 (ACAd5, r=-0.67, p=0.047, n=9) and 6a (ACAd6a, r=-0.69, p=0.037, n=9), ventral agranular insular area, layer 6a (AIv6a, r=-0.83, p=0.0057, n=9), ectorhinal area, layer 2/3 (ECT2/3, r=-0.87, p=0.023, n=6), cortical amygdalar area (COA, r=-0.86, p=0.0032, n=9), CA1 (r=-0.76, p=0.019, n=9), CA3 (r=-0.78, p=0.014, n=9), cortical subplate: lateral amygdala (LA, r=-0.67, p=0048, n=9), basomedial amygdala (BMA, r=-0.86, p=0.0027, n=9), striatum: anterior amygdalar area (AAA, r=-0.83, p=0.042, n=6), central amygdalar area (CEA, r=-0.71, p=0.031, n=9), intercalated amygdalar nucleus (IA, r=-0.80, p=0.0093, n=9), thalamus: anteroventral nucleus of the thalamus (AV, r=-0.71, p=0.048, n=8), intralaminar nuclei of the dorsal thalamus (ILM, r=-0.75, p=0.019, n=9), medial habenula (MH, r=-0.69, p=0.034, n=9), hypothalamus: arcuate nucleus (ARH, r=-0.86, p=0.0060, n=9), dorsal medial nucleus (DMH, r=-0.81, p=0.015, n=9), medial zone (MEZ, r=-0.75, p=0.021, n=9), tuberal nucleus (TU, r=-0.78, p=0.0022, n=8), zona incerta (ZI, r=-0.86, p=0.0027, n=9). (**B**) Significant negative correlations were found in the contralateral isocortex: MOs6b (r=-085, p=0.0079, n=8), olfactory area: dorsal taenia tecta (TTd, r=-0.74, p=0.036, n=8), cortical subplate: lateral amygdala (LA, r=-0.71, p=0.031, n=9), striatum: medial amygdalar nucleus (MEA, r=-0.67, p=0.050, n=9), thalamus: intermediodorsal nucleus (IMD, r=-0.85, p=0.0034, n=9), intralaminar nuclei of the dorsal thalamus (ILM, r=-0.76, p=0.17, n=9), MH (r=-0.81, p=0.0076, n=9), and hypothalamus: DMH (r=-0.91, p=0.0016, n=8), posterior hypothalamic nucleus (PH, r=-0.89, p=0.017, n=6), lateral hypothalamic area (LHA, r=-0.79, p=0.011, n=9), TU (r=-0.89, p=0.0026, n=8), ZI (r=-0.82, p=0.0073, n=9). Shaded areas display 95% confidence interval.

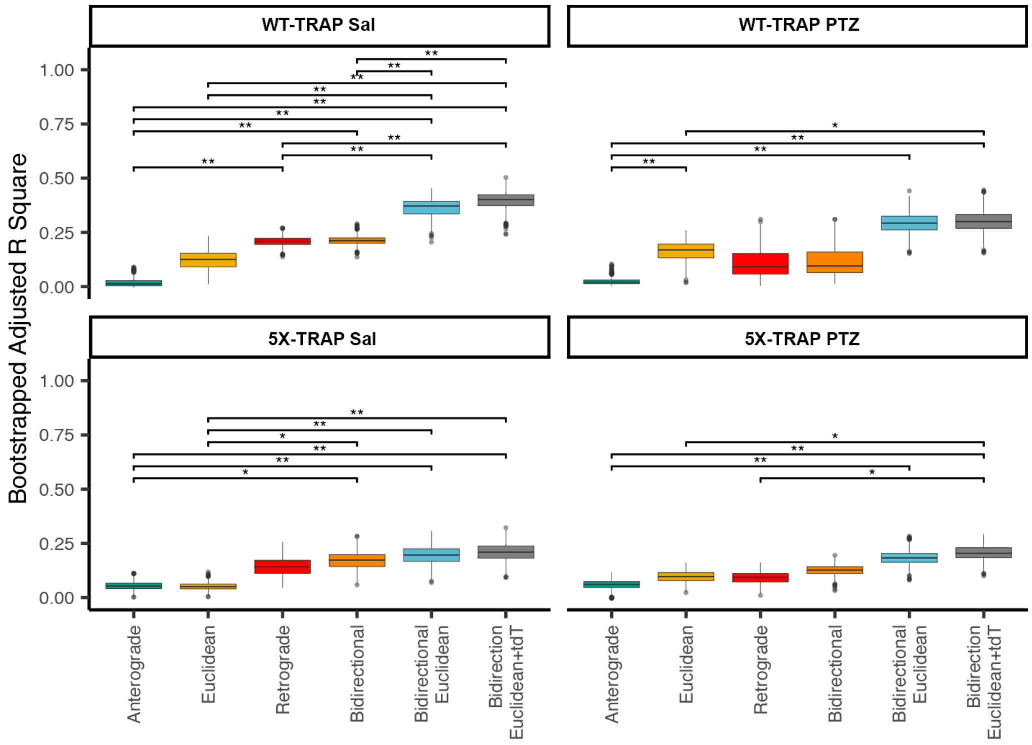

Figure S12. Between model comparisons of tau spread model fit for each experimental group. We fit spread models using a network defined by either anterograde connectivity, retrograde connectivity or Euclidean distance between regions. We also generated "bidirectional" models that combined anterograde and retrograde connections ("Bidirectional"), with the addition of Euclidean distance ("Bidirectional Euclidean") and a vector of tdT positive cell counts ("Bidirectional Euclidean + tdT"). We bootstrapped adjusted R square values for each of these model architectures to compare the model fit while accounting for the fact that increased model complexity will always improve training set performance. We compared bootstrapped adjusted R square models between groups for each model architecture. p-values were adjusted for multiple comparisons in each panel using FDR correction. *, $p<0.05$; **, $p<0.01$. Box plots boxes display the median, 25th and 75th percentiles, and whiskers extend to the data point that is at most 1.5 x interquartile range, and data beyond this point are plotted individually.

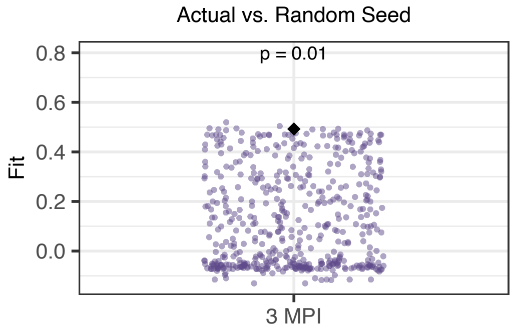

Figure S13. Null model demonstrating specificity of computational model to experimental seed site. We repeated the retrograde spread modeling with WT-TRAP mice using 500 random seed sites comprised of 4 seed sites with similar spatial clustering (mean interregional Euclidean distance) to the experimental seed site, which was the right dentate gyrus, CA1, CA3, and posterior parietal association area. Model fits when using random seeds are shown in light purple and model fit when using the experimental seed site is shown as a black diamond. When implementing the experimental seed site into the model in silico, the model performance in predicting regional AT8+ levels was better than 99% of random sites.

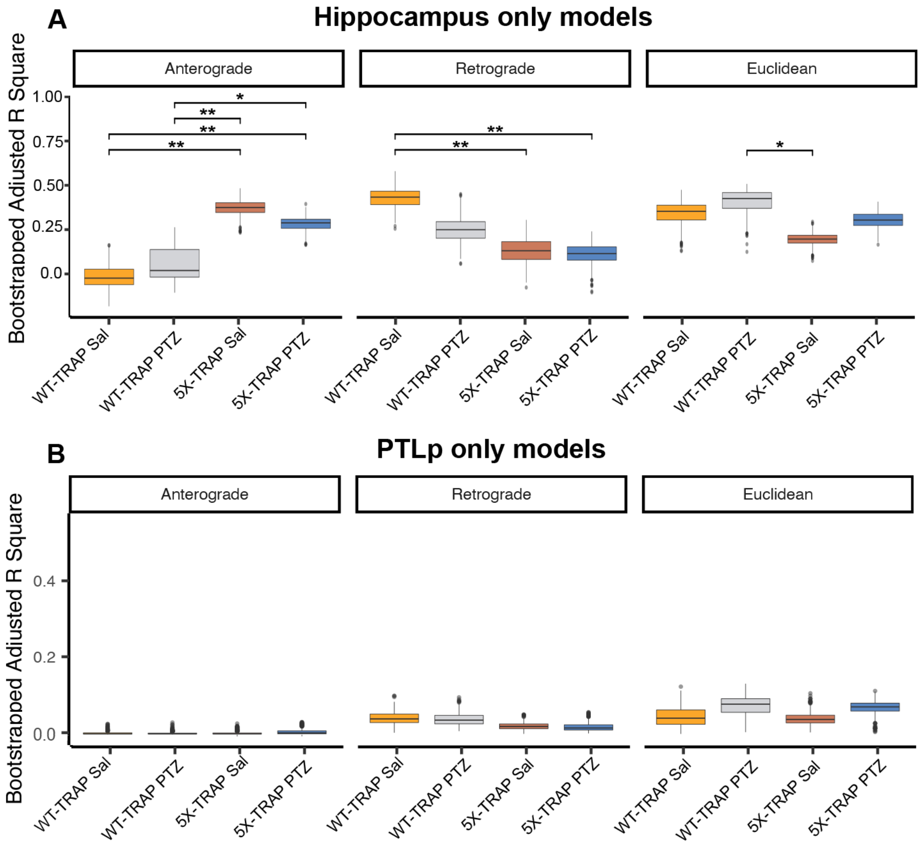

Figure S14. Between group comparisons of single site models of tau spread. (**A**) Bootstrapped adjusted R square values of predictions of tau spread based on anterograde, retrograde, and Euclidean models of spread models incorporating a single hippocampal seed site recapitulate results from the dual site models (Fig 4). (**B**) Bootstrapped adjusted R square values of predictions of tau spread based on anterograde, retrograde, and Euclidean models of spread models incorporating a single posterior parietal association cortex (PTLp) seed site perform poorly across all experimental groups. p-values were adjusted for multiple comparisons in each panel using FDR correction: *p<0.05, **p<0.01. Box plots boxes display the median, 25th and 75th percentiles, and whiskers extend to the data point that is at most 1.5 x interquartile range, and data beyond this point are plotted individually.

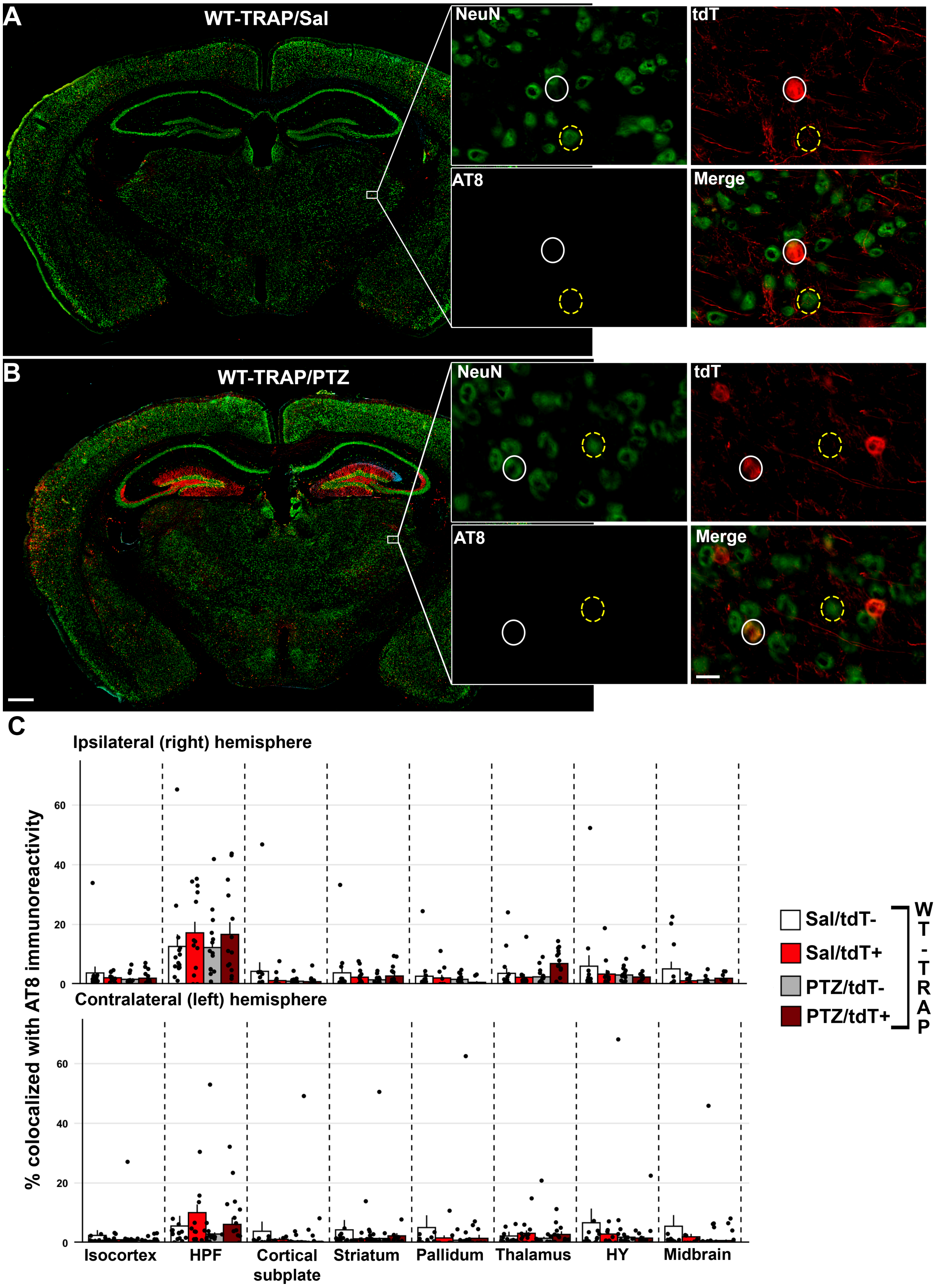

Figure S15. tdT+ and tdT- neurons colocalization with tau pathology in WT-TRAP mice.

Representative slide scanned coronal sections from (**A**) saline and (**B**) PTZ treated WT-TRAP

mice. Inset displays thalamic tdT+ (white circles) and tdT- (NeuN+) (yellow dashed circles) neurons colocalization with AT8+ immunoreactive aggregates. Scale bars = 500 μm, 20 μm (inset). (**C**) tdT+, tdT-, and colocalized AT8+ immunoreactive aggregates were mapped to the Allen Brain Atlas and % of detected tdT+/tdT- cells were quantified. Two-way ANOVA with treatment (Sal vs PTZ) and tdT labelling (tdT+ vs tdT-) as independent variables were performed. No significant differences were found, n=8-15/group/brain region. Histograms display group mean ± SEM.

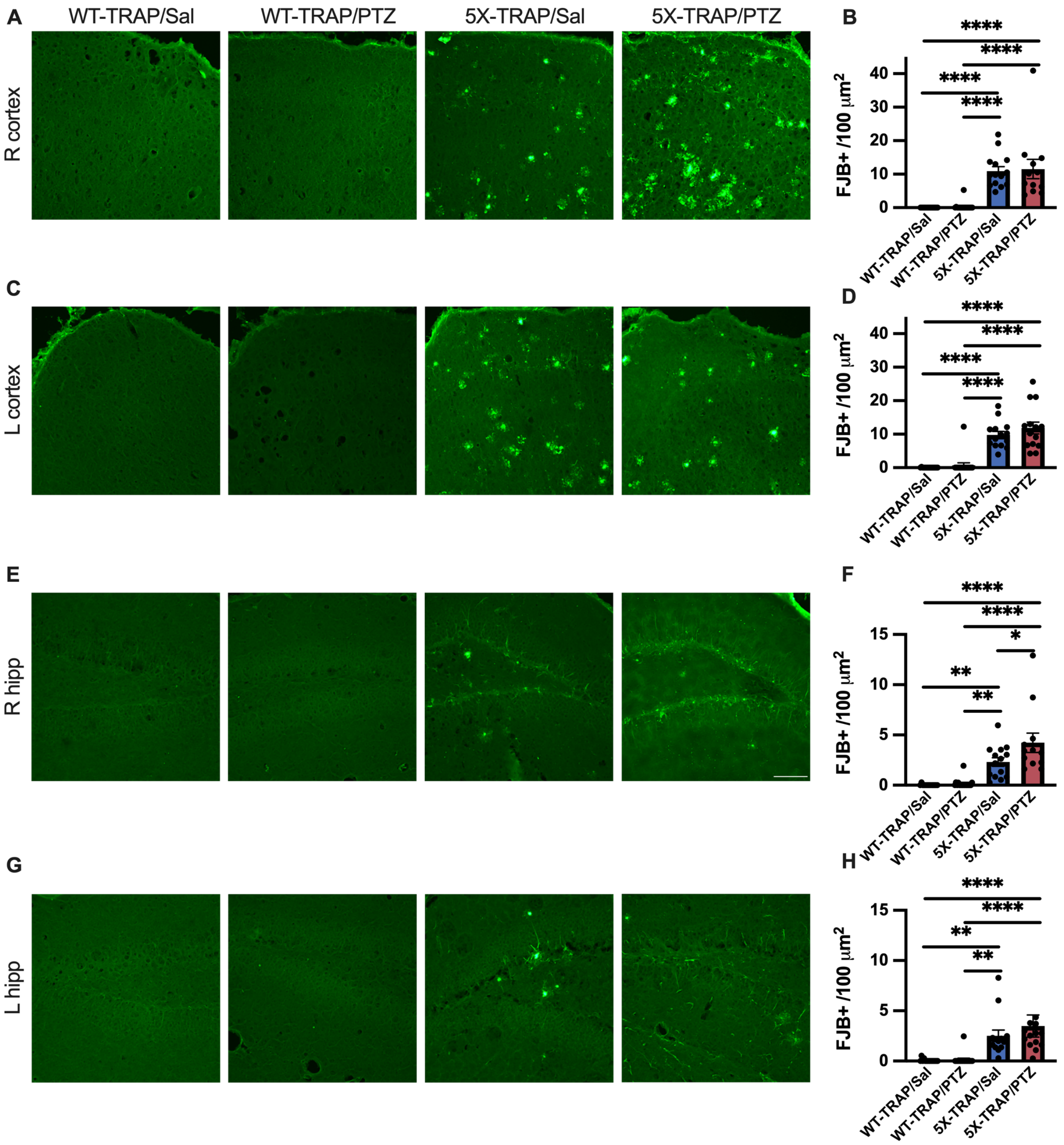

Figure S16. Neurodegeneration in AD-tau seeded 5X-TRAP mice is exacerbated by PTZ kindling. Neurodegeneration was assessed by Fluorojade-B (FJB) stain. Representative images of FJB stain from the (**A**) ipsilateral to injection site (right, R) cortex and (**C**) contralateral

(left, L) cortex, (**E**) ipsilateral hippocampus, and (**G**) contralateral hippocampus. Scale bar = 50 µm. FJB+ counts were compared by two-way ANOVA with genotype (WT-TRAP vs 5X-TRAP) and kindling (Sal vs PTZ) as independent variables. Increased FJB+ counts were found in Sal and PTZ treated 5X-TRAP mice compared to Sal and PTZ treated WT-TRAP mice in all sampled brain regions ((**B**) R cortex genotype: $F_{1,53}$=62.19, p<0.0001; (**D**) L cortex genotype: $F_{1,56}$=99.55; p<0.0001; (**F**) R hippocampus genotype: $F_{1,53}$=47.40, p<0.0001; (**H**) L hippocampus genotype: $F_{1,56}$=24.78, p<0.0001), and in PTZ kindled 5X-TRAP mice compared to Sal treated 5X-TRAP mice in the R hippocampus (kindling: $F_{1,53}$=4.94, p<0.05). Tukey's post hoc: *,**, ****=p<0.05, 0.01, 0.0001. n=13-17/group. Histograms display group mean ± SEM.

ID	Parent.ID	Region	WT.Sal	WT.Sal n	WT.PTZ	WT.PTZ n	5X.Sal	5X.Sal n	5X.PTZ	5X.PTZ n	Genotype effect	Treatment effect	Interaction	WT Sal vs WT PTZ	WT Sal vs WT 5XFAD	WT Sal vs 5XFAD PTZ	5XFAD Sal vs WT PTZ	WT PTZ VS 5XFAD PT	5XFAD Sal vs 5XFAD PTZ
R 315	R 695	R Isocortex	10.28 ± 2.23	15	14.04 ± 4.14	15	23.64 ± 4.35	10	35.01 ± 3.64	10	0.94 [-2.29, 4.17]	0.95 [-2.61, 4.41]	-1.88 [-6.54, 2.86]						
L 315	L 695	L Isocortex	4.57 ± 1.26	15	4.93 ± 1.28	15	11.53 ± 1.94	10	21.57 ± 4.37	10	2.53 [-0.78, 5.83]	2.03 [-1.55, 5.53]	-3.25 [-8.13, 1.45]						
R 500	R 315	R Somatomotor areas	3.75 ± 0.79	15	12.15 ± 7.23	15	18.43 ± 3.96	10	24.66 ± 5.73	10	2.21 [-0.98, 5.41]	1.52 [-2.05, 4.99]	-0.57 [-5.27, 4.02]						
L 500	L 315	L Somatomotor areas	3.27 ± 0.81	15	6.12 ± 2.22	15	17.63 ± 3.46	10	23.49 ± 6.20	10	4.20 [0.93, 7.53]*	1.61 [-1.96, 5.18]	-2.03 [-6.8, 2.69]	-1.04 [-3.97, 1.67]	-4.64 [-7.87, -1.57]*	-6.22 [-9.35, -3.02]*	3.60 [0.57, 6.8]*	-5.17 [-8.36, -2.03]*	-1.58 [-5.2, 1.77]
R 985	R 500	R Primary motor area	6.44 ± 1.29	13	14.88 ± 7.80	15	23.84 ± 5.28	10	35.46 ± 7.70	9	2.70 [-0.64, 6.1]	2.26 [-1.35, 5.93]	-2.27 [-7.15, 2.54]						
L 985	L 500	L Primary motor area	5.22 ± 1.33	15	7.08 ± 2.35	15	24.37 ± 5.65	10	37.62 ± 9.84	9	5.36 [2, 8.66]*	2.99 [-0.7, 6.62]	-3.94 [-8.75, 0.92]	-0.52 [-3.34, 2.27]	-3.87 [-7.05, -0.67]*	-6.87 [-9.99, -3.54]*	3.34 [0.24, 6.45]*	-6.34 [-9.45, -2.99]*	-2.97 [-6.44, 0.72]
R 993	R 500	R Secondary motor area	5.80 ± 1.65	11	23.38 ± 15.05	15	36.65 ± 7.58	10	53.69 ± 11.17	9	4.74 [1.38, 8.08]*	3.40 [-0.16, 7.02]	-1.73 [-6.62, 3.12]	-3.11 [-6.03, -0.14]*	-5.47 [-8.77, -2.18]*	-8.84 [-12.26, -5.55]*	2.34 [-0.81, 5.43]	-5.73 [-8.85, -2.36]*	-3.38 [-6.96, 0.03]
L 993	L 500	L Secondary motor area	4.74 ± 1.34	13	15.44 ± 7.16	13	31.73 ± 5.20	10	44.49 ± 10.09	9	5.41 [1.98, 8.82]*	3.13 [-0.41, 6.67]	-1.98 [-6.79, 2.9]	-2.62 [-5.63, 0.32]	-5.87 [-9.12, -2.72]*	-9.00 [-12.28, -5.76]*	3.27 [0.12, 6.51]*	-6.39 [-9.5, -2.89]*	-3.11 [-6.54, 0.39]
R 453	R 315	R Somatosensory areas	7.98 ± 2.04	15	10.58 ± 3.13	15	16.86 ± 2.61	10	33.54 ± 5.92	10	1.78 [-1.46, 4.97]	1.94 [-1.57, 5.46]	-2.90 [-7.58, 1.72]						
L 453	L 315	L Somatosensory areas	3.77 ± 1.03	15	4.88 ± 1.41	15	10.63 ± 1.88	10	17.38 ± 2.93	9	2.81 [-0.54, 6.15]	2.24 [-1.34, 5.89]	-3.24 [-8, 1.53]						
R 322	R 453	R Primary somatosensory area	13.09 ± 3.48	14	14.55 ± 4.78	15	22.31 ± 4.10	9	39.50 ± 7.03	9	1.40 [-1.94, 4.7]	1.82 [-1.87, 5.51]	-3.02 [-7.87, 1.85]						
L 322	L 453	L Primary somatosensory area	4.49 ± 1.24	15	5.16 ± 1.33	15	13.17 ± 2.48	10	21.91 ± 3.69	9	3.24 [-0.08, 6.63]	2.40 [-1.24, 5.98]	-3.56 [-8.25, 1.25]						
R 353	R 322	R Primary somatosensory area, nose	5.00 ± 1.42	10	2.96 ± 0.91	9	34.14 ± 17.04	8	130.19 ± 82.08	8	24.08 [20.26, 27.85]*	19.52 [15.64, 23.4]*	-20.80 [-26.16, -15.4]*	-0.18 [-3.65, 3.31]	-5.74 [-9.36, -2.12]*	-25.24 [-28.83, -21.61]*	5.54 [1.92, 9.3]*	-25.05 [-28.78, -21.45]*	-19.50 [-23.24, -15.64]*
L 353	L 322	L Primary somatosensory area, nose	2.53 ± 1.22	9	10.19 ± 6.34	7	14.55 ± 3.92	8	40.70 ± 22.49	7	11.25 [7.17, 15.3]*	10.38 [6.47, 14.28]*	-8.62 [-14.27, -2.97]*	-3.22 [-7.05, 0.36]	-5.05 [-8.69, -1.36]*	-15.43 [-19.21, -11.75]*	1.88 [-2.13, 5.47]	-12.22 [-16.11, -8.09]*	-10.35 [-14.1, -6.49]*
R 329	R 322	R Primary somatosensory area, barrel field	21.31 ± 6.52	13	25.20 ± 7.66	15	36.52 ± 7.51	10	60.61 ± 13.56	9	1.16 [-2.11, 4.43]	1.59 [-1.97, 5.21]	-2.71 [-7.41, 2.12]						
L 329	L 322	L Primary somatosensory area, barrel field	6.90 ± 2.48	14	6.63 ± 2.17	15	19.37 ± 3.84	10	34.91 ± 5.68	9	3.61 [0.31, 6.98]*	2.70 [-0.93, 6.36]	-4.06 [-8.94, 0.75]	-0.10 [-2.94, 2.88]	-1.98 [-5.26, 1.19]	-4.68 [-8.02, -1.45]*	1.89 [-1.25, 5.07]	-4.57 [-7.64, -1.1]*	-2.66 [-6.25, 0.81]
R 337	R 322	R Primary somatosensory area, lower limb	20.10 ± 9.07	13	14.48 ± 5.11	11	49.70 ± 10.78	10	96.53 ± 21.81	9	3.35 [-0.17, 6.85]	2.80 [-0.84, 6.39]	-4.17 [-9.06, 0.78]						
L 337	L 322	L Primary somatosensory area, lower limb	6.74 ± 3.03	13	3.01 ± 1.18	10	30.68 ± 5.73	10	58.81 ± 15.27	9	7.59 [3.94, 11.16]*	4.62 [0.99, 8.22]*	-6.30 [-11.32, -1.32]*	0.21 [-2.92, 3.46]	-3.72 [-7.21, -0.69]*	-8.33 [-11.55, -4.89]*	3.98 [0.53, 7.37]*	-8.58 [-11.88, -4.94]*	-4.60 [-8.1, -1.09]*
R 345	R 322	R Primary somatosensory area, mouth	7.15 ± 1.86	13	11.45 ± 4.21	13	17.03 ± 4.05	9	20.59 ± 5.08	9	0.74 [-2.69, 4.15]	1.00 [-2.64, 4.72]	-1.62 [-6.5, 3.49]						
L 345	L 322	L Primary somatosensory area, mouth	9.63 ± 3.91	13	6.09 ± 1.03	12	15.55 ± 4.92	10	18.33 ± 4.13	8	0.52 [-3.05, 4.22]	0.81 [-3.01, 4.56]	-2.12 [-7.21, 2.97]						
R 369	R 322	R Primary somatosensory area, upper limb	13.70 ± 4.35	14	11.60 ± 4.83	13	24.59 ± 4.76	10	67.75 ± 19.75	9	3.52 [0.12, 6.93]*	3.59 [-0.03, 7.17]	-5.00 [-9.85, -0.27]*	-0.06 [-2.99, 2.85]	-0.97 [-4.07, 2.25]	-4.52 [-7.7, -1.1]*	0.94 [-2.28, 4.17]	-4.46 [-7.78, -1.19]*	-3.57 [-6.91, 0.11]
L 369	L 322	L Primary somatosensory area, upper limb	9.66 ± 3.36	14	6.89 ± 2.87	13	20.72 ± 4.37	10	34.75 ± 8.80	9	2.32 [-1.11, 5.72]	1.92 [-1.68, 5.45]	-3.48 [-8.31, 1.43]						
R 361	R 322	R Primary somatosensory area, trunk	51.38 ± 26.74	14	37.77 ± 26.23	12	95.76 ± 30.65	10	145.56 ± 41.58	9	1.62 [-1.9, 5.07]	1.42 [-2.18, 5.04]	-3.00 [-7.83, 1.93]						
L 361	L 322	L Primary somatosensory area, trunk	6.44 ± 1.56	13	6.60 ± 2.40	13	35.13 ± 5.87	10	46.55 ± 11.90	9	5.66 [2.26, 9.07]*	2.22 [-1.42, 5.76]	-3.45 [-8.23, 1.51]	-0.23 [-3.33, 2.74]	-4.64 [-7.98, -1.42]*	-6.85 [-10.18, -3.52]*	4.41 [1.07, 7.51]*	-6.62 [-9.92, -3.24]*	-2.23 [-5.59, 1.43]
R 182305689	R 322	R Primary somatosensory area, unassigned	72.29 ± 39.72	14	8.27 ± 3.64	11	25.05 ± 5.66	10	83.67 ± 24.57	9	0.35 [-3.18, 3.9]	1.28 [-2.35, 4.93]	-3.28 [-8.28, 1.59]						
L 182305689	L 322	L Primary somatosensory area, unassigned	7.64 ± 4.28	14	3.45 ± 1.32	11	14.41 ± 2.64	10	41.83 ± 12.39	9	4.58 [1.09, 8.13]*	4.04 [0.43, 7.67]*	-5.95 [-10.94, -0.95]*	0.46 [-2.61, 3.52]	-1.07 [-4.38, 2]	-5.07 [-8.21, -1.79]*	1.53 [-1.87, 4.75]	-5.53 [-8.96, -2.2]*	-4.01 [-7.46, -0.49]*
R 378	R 453	R Supplemental somatosensory area	8.32 ± 2.89	11	18.36 ± 8.34	15	29.30 ± 4.90	9	60.38 ± 14.11	10	3.96 [0.66, 7.15]*	3.58 [-0.02, 7.21]	-3.84 [-8.76, 1.06]	-1.20 [-4.28, 1.81]	-2.59 [-6.01, 0.98]	-6.13 [-9.54, -2.85]*	1.39 [-1.75, 4.69]	-4.94 [-8.04, -1.8]*	-3.56 [-7.15, -0.07]*
L 378	L 453	L Supplemental somatosensory area	8.59 ± 2.35	14	12.11 ± 4.05	15	20.82 ± 3.37	10	50.86 ± 15.16	9	3.14 [-0.13, 6.46]	3.07 [-0.54, 6.7]	-3.98 [-8.85, 0.81]						
R 1057	R 315	R Gustatory areas	13.02 ± 3.89	15	11.76 ± 3.43	13	18.97 ± 5.00	10	25.50 ± 6.51	10	-0.09 [-3.47, 3.27]	0.34 [-3.16, 3.88]	-1.69 [-6.42, 2.98]						
L 1057	L 315	L Gustatory areas	6.25 ± 2.92	15	8.05 ± 3.73	13	14.53 ± 4.51	10	35.97 ± 14.13	10	2.99 [-0.32, 6.31]	3.28 [-0.29, 6.79]	-3.91 [-8.63, 0.88]						
R 677	R 315	R Visceral area	15.05 ± 5.31	13	29.68 ± 17.13	13	31.77 ± 9.44	9	44.54 ± 11.79	10	-0.20 [-3.53, 3.13]	0.70 [-2.9, 4.32]	-0.94 [-5.88, 3.87]						
L 677	L 315	L Visceral area	9.44 ± 2.88	14	10.52 ± 5.44	13	25.27 ± 3.71	10	35.68 ± 6.46	9	1.21 [-2.18, 4.63]	0.67 [-3.03, 4.34]	-1.81 [-6.66, 3.1]						
R 247	R 315	R Auditory areas	17.78 ± 5.75	14	8.29 ± 2.05	15	20.08 ± 2.64	10	39.43 ± 8.95	9	0.63 [-2.71, 4.01]	0.92 [-2.67, 4.54]	-2.68 [-7.51, 2.2]						
L 247	L 315	L Auditory areas	10.83 ± 2.92	14	6.04 ± 1.94	14	11.06 ± 2.13	10	54.02 ± 32.01	10	3.09 [-0.2, 6.29]	3.80 [0.26, 7.28]*	-5.24 [-9.94, -0.55]*	-0.01 [-2.87, 2.89]	-0.30 [-3.58, 2.77]	-4.06 [-7.34, -0.98]*	0.28 [-2.73, 3.58]	-4.06 [-7.19, -0.85]*	-3.77 [-7.3, -0.44]*
R 1011	R 247	R Dorsal auditory area	27.64 ± 11.66	12	10.47 ± 2.59	15	36.61 ± 4.73	10	56.42 ± 11.20	8	0.55 [-2.92, 3.93]	0.55 [-3.12, 4.22]	-2.37 [-7.31, 2.52]						
L 1011	L 247	L Dorsal auditory area	17.69 ± 8.89	12	8.52 ± 3.05	14	21.26 ± 3.62	10	83.71 ± 49.15	10	2.90 [-0.42, 6.2]	3.38 [-0.13, 6.91]	-4.93 [-9.84, -0.25]*	0.10 [-2.89, 3.11]	-0.39 [-3.73, 2.85]	-3.78 [-7, -0.46]*	0.53 [-2.71, 3.69]	-3.87 [-6.98, -0.7]*	-3.35 [-6.86, 0.01]
R 1002	R 247	R Primary auditory area	32.54 ± 13.40	13	18.16 ± 6.26	11	29.12 ± 6.48	10	63.99 ± 24.47	7	0.55 [-3.2, 4.22]	1.25 [-2.54, 5.04]	-2.77 [-7.79, 2.22]						
L 1002	L 247	L Primary auditory area	16.81 ± 4.93	13	8.79 ± 3.94	13	16.43 ± 4.11	10	74.83 ± 52.16	9	2.93 [-0.46, 6.32]	3.66 [0.07, 7.21]*	-5.15 [-10.02, -0.33]*	0.04 [-3.01, 3.12]	-0.25 [-3.43, 3]	-3.88 [-7.14, -0.62]*	0.26 [-2.97, 3.48]	-3.91 [-7.17, -0.6]*	-3.64 [-7.28, -0.31]*
L 1027	L 247	L Posterior auditory area	20.38 ± 6.63	7	3.38 ± 2.15	7	19.22 ± 10.50	8	33.38 ± 14.04	6									
R 1018	R 247	R Ventral auditory area	33.66 ± 9.68	14	13.65 ± 3.35	15	27.87 ± 4.88	10	51.41 ± 15.55	8	-0.01 [-3.46, 3.39]	0.50 [-3.17, 4.23]	-2.33 [-7.25, 2.56]						
L 1018	L 247	L Ventral auditory area	15.58 ± 5.15	14	10.66 ± 3.62	14	14.93 ± 3.20	10	89.32 ± 55.95	10	3.70 [0.45, 7.03]*	4.62 [1.12, 8.07]*	-5.92 [-10.63, -1.15]*	-0.16 [-3.14, 2.73]	-0.23 [-3.38, 2.93]	-4.82 [-7.93, -1.58]*	0.08 [-3.15, 3.16]	-4.66 [-7.87, -1.52]*	-4.60 [-8.12, -1.27]*
R 669	R 315	R Visual areas	54.72 ± 25.32	14	48.36 ± 17.02	15	57.83 ± 19.53	10	110.80 ± 29.95	9	0.04 [-3.3, 3.43]	0.82 [-2.79, 4.5]	-2.16 [-7.04, 2.66]						
L 669	L 315	L Visual areas	8.42 ± 2.63	14	3.54 ± 1.08	14	13.11 ± 3.21	10	37.91 ± 18.18	8	3.10 [-0.41, 6.53]	3.16 [-0.48, 6.79]	-4.75 [-9.66, 0.18]						
L 402	L 669	L Anterolateral visual area	15.34 ± 6.99	8	4.77 ± 1.90	7	16.94 ± 6.70	7	117.60 ± 92.92	7	6.16 [2.12, 10.25]*	6.24 [2.22, 10.31]*	-7.82 [-13.53, -2.22]*	0.14 [-3.66, 3.99]	-0.79 [-4.54, 3.07]	-6.99 [-10.93, -3.09]*	0.93 [-3.25, 4.73]	-7.08 [-11.05, -3.11]*	-6.19 [-10.31, -2.32]*
R 394	R 669	R Anteromedial visual area	44.33 ± 13.76	12	95.21 ± 36.19	14	103.91 ± 29.82	10	215.19 ± 64.92	9	1.65 [-1.68, 5.05]	2.36 [-1.25, 5.9]	-2.49 [-7.45, 2.42]						
L 394	L 669	L Anteromedial visual area	11.54 ± 5.55	13	8.21 ± 4.02	13	28.16 ± 5.57	10	53.97 ± 14.75	8	3.01 [-0.45, 6.47]	2.44 [-1.22, 6.07]	-3.78 [-8.69, 1.16]						
R 385	R 669	R Primary visual area	78.58 ± 24.18	11	164.43 ± 89.74	9	82.68 ± 36.28	9	230.76 ± 68.78	6	-0.70 [-4.75, 3.42]	1.55 [-2.58, 5.64]	-1.46 [-6.9, 3.99]						
L 385	L 669	L Primary visual area	14.06 ± 3.55	11	3.49 ± 1.28	10	13.67 ± 5.21	8	52.83 ± 27.42	8	2.52 [-1.11, 6.2]	3.23 [-0.63, 7.1]	-4.90 [-10.15, 0.24]						
R 533	R 669	R posteromedial visual area	237.86 ± 167.43	10	153.57 ± 107.41	7	81.10 ± 31.50	9	152.00 ± 30.36	6	-1.46 [-5.66, 2.76]	-0.03 [-3.98, 4]	-1.35 [-6.95, 4.12]						
L 533	L 669	L posteromedial visual area	26.40 ± 7.87	10	13.53 ± 7.23	9	18.53 ± 5.23	9	43.13 ± 10.06	7	-0.22 [-4.18, 3.74]	0.82 [-3.1, 4.74]	-2.24 [-7.59, 3.06]						
R 31	R 315	R Anterior cingulate area	8.29 ± 2.44	15	13.78 ± 5.10	15	32.48 ± 10.49	10	38.50 ± 12.30	10	1.87 [-1.45, 5.13]	0.57 [-3, 4.11]	-1.20 [-5.96, 3.41]						
L 31	L 315	L Anterior cingulate area	3.94 ± 0.80	15	11.65 ± 5.33	14	21.63 ± 5.61	10	34.37 ± 11.04	10	4.70 [1.39, 8]*	3.07 [-0.44, 6.6]	-2.46 [-7.27, 2.12]	-2.06 [-5, 0.66]	-4.71 [-7.85, -1.46]*	-7.75 [-10.91, -4.63]*	2.63 [-0.61, 5.79]	-5.68 [-8.92, -2.57]*	-3.04 [-6.51, 0.36]
R 39	R 31	R Anterior cingulate area, dorsal part	6.93 ± 1.86	13	17.59 ± 10.04	14	42.33 ± 9.84	10	54.62 ± 16.08	10	4.08 [0.73, 7.4]*	1.59 [-2.04, 5.14]	-1.15 [-6.05, 3.68]	-1.88 [-4.96, 1.06]	-5.39 [-8.6, -2.03]*	-6.95 [-10.12, -3.6]*	3.50 [0.35, 6.64]*	-5.07 [-8.41, -1.99]*	-1.57 [-4.99, 1.92]
L 39	L 31	L Anterior cingulate area, dorsal part	4.57 ± 1.05	15	20.00 ± 10.28	14	33.95 ± 8.46	10	41.62 ± 8.83	10	3.65 [0.33, 6.94]*	1.50 [-2.08, 5.07]	0.54 [-4.18, 5.35]	-3.51 [-6.38, -0.7]*	-6.63 [-9.96, -3.58]*	-8.11 [-11.33, -4.98]*	3.15 [0.03, 6.34]*	-4.63 [-7.71, -1.32]*	-1.49 [-4.83, 1.98]
R 48	R 31	R Anterior cingulate area, ventral part	21.21 ± 6.18	15	20.35 ± 6.30	14	57.09 ± 22.12	10	66.57 ± 21.94	10	0.94 [-2.37, 4.23]	0.28 [-3.28, 3.72]	-1.51 [-6.16, 3.13]						
L 48	L 31	L Anterior cingulate area, ventral part	7.54 ± 2.32	15	14.40 ± 5.09	13	32.99 ± 9.06	10	66.33 ± 26.42	10	5.69 [2.41, 9.02]*	4.25 [0.8, 7.72]*	-4.57 [-9.27, 0.2]	-1.14 [-4.07, 1.75]	-3.57 [-6.73, -0.42]*	-7.81 [-10.95, -4.72]*	2.42 [-0.79, 5.62]	-6.66 [-9.81, -3.44]*	-4.22 [-7.48, -0.69]*
R 95	R 315	R Agranular insular area	6.38 ± 2.48	14	12.03 ± 4.65	15	17.93 ± 3.66	9	20.94 ± 3.65	10	0.29 [-2.98, 3.55]	0.31 [-3.33, 3.87]	-0.72 [-5.54, 4.11]						
L 95	L 315	L Agranular insular area	5.38 ± 1.72	15	5.03 ± 0.89	15	14.86 ± 3.54	10	20.88 ± 4.34	10	1.84 [-1.39, 5.1]	0.95 [-2.52, 4.4]	-2.31 [-6.92, 2.3]						
R 104	R 95	R Agranular insular area, dorsal part	6.52 ± 2.24	11	15.32 ± 5.72	14	26.78 ± 9.26	9	28.42 ± 7.20	10	0.94 [-2.4, 4.21]	0.11 [-3.46, 3.72]	-0.29 [-5.2, 4.67]						
L 104	L 95	L Agranular insular area, dorsal part	6.25 ± 1.72	14	7.36 ± 2.40	15	24.94 ± 7.85	10	38.77 ± 12.65	9	3.91 [0.58, 7.27]*	2.05 [-1.54, 5.57]	-3.12 [-8.01, 1.64]	-0.39 [-3.31, 2.47]	-3.23 [-6.64, -0.17]*	-5.26 [-8.54, -1.94]*	2.84 [-0.24, 6.04]	-4.86 [-8.33, -1.88]*	-2.01 [-5.39, 1.58]
R 111	R 95	R Agranular insular area, posterior part	11.70 ± 4.74	12	26.16 ± 17.79	12	35.56 ± 10.45	9	33.17 ± 6.35	10	-0.50 [-3.88, 2.93]	-0.35 [-3.93, 3.32]	0.20 [-4.76, 5.06]						
L 111	L 95	L Agranular insular area, posterior part	11.55 ± 4.78	15	8.13 ± 2.55	13	24.42 ± 5.26	10	32.43 ± 6.24	10	0.94 [-2.34, 4.22]	0.53 [-3, 4.14]	-2.06 [-6.88, 2.71]						
R 119	R 95	R Agranular insular area, ventral part	11.71 ± 5.05	14	19.54 ± 10.15	14	26.73 ± 5.86	9	31.41 ± 8.69	10	-0.05 [-3.37, 3.24]	0.24 [-3.32, 3.87]	-0.92 [-5.71, 3.84]						
L 119	L 95	L Agranular insular area, ventral part	6.59 ± 1.79	14	5.92 ± 1.40	15	47.52 ± 24.82	10	40.86 ± 14.79	9	4.20 [0.79, 7.53]*	-1.19 [-4.8, 2.49]	-0.14 [-5.05, 4.72]	-0.12 [-2.9, 2.86]	-6.51 [-9.49, -3.22]*	-5.30 [-8.51, -1.99]*	6.37 [3.26, 9.51]*	-5.17 [-8.39, -1.85]*	1.21 [-2.27, 4.75]
R 254	R 315	R Retrosplenial area	22.27 ± 9.52	14	24.48 ± 11.17	15	98.79 ± 32.94	10	88.68 ± 13.82	9	1.78 [-1.57, 5.07]	-0.63 [-4.22, 2.95]	-0.55 [-5.37, 4.25]						
L 254	L 315	L Retrosplenial area	10.49 ± 4.30	14	11.16 ± 4.02	15	30.99 ± 6.29	10	42.24 ± 6.72	10	1.85 [-1.42, 5.18]	0.90 [-2.6, 4.44]	-2.13 [-6.84, 2.62]						
R 894	R 254	R Retrosplenial area, lateral agranular part	28.55 ± 7.17	14	28.25 ± 12.27	15	78.36 ± 13.86	10	111.40 ± 19.00	9	1.82 [-1.52, 5.12]	1.01 [-2.63, 4.58]	-2.33 [-7.06, 2.37]						
L 894	L 254	L Retrosplenial area, lateral agranular part	11.77 ± 3.81	13	6.90 ± 2.30	15	41.80 ± 8.89	10	56.81 ± 6.75	10	3.11 [-0.17, 6.38]	1.11 [-2.4, 4.61]	-2.81 [-7.57, 1.91]						
R 879	R 254	R Retrosplenial area, dorsal part	15.21 ± 3.59	15	12.53 ± 3.13	15	85.87 ± 21.90	9	110.52 ± 17.06	9	5.14 [1.83, 8.46]*	1.43 [-2.17, 5.16]	-2.89 [-7.71, 1.9]	0.03 [-2.76, 2.88]	-4.71 [-7.93, -1.36]*	-6.11 [-9.41, -2.91]*	4.72 [1.43, 7.89]*	-6.10 [-9.3, -2.94]*	-1.39 [-5.06, 2.1]
L 879	L 254	L Retrosplenial area, dorsal part	11.05 ± 2.70	14	9.64 ± 3.10	15	33.57 ± 5.62	10	61.98 ± 12.01	9	3.44 [0.1, 6.79]*	2.23 [-1.42, 5.85]	-3.65 [-8.39, 1.1]	-0.03 [-2.86, 2.85]	-2.25 [-5.46, 1]	-4.45 [-7.79, -1.24]*	2.21 [-0.89, 5.36]	-4.40 [-7.64, -1.18]*	-2.19[-5.71, -0.21]*
R 886	R 254	R Retrosplenial area, ventral part	47.86 ± 24.36	15	60.11 ± 29.98	15	236.46 ± 88.67	10	177.67 ± 34.74	10	1.34 [-1.97, 4.67]	-1.40 [-4.9, 2.2]	0.33 [-4.49, 5.04]						
L 886	L 254	L Retrosplenial area, ventral part	22.31 ± 11.30	14	27.87 ± 11.52	15	61.71 ± 14.92	10	76.84 ± 14.47	10	1.11 [-2.12, 4.33]	0.53 [-2.99, 4.02]	-1.60 [-6.35, 3.14]						
R 22	R 315	R Posterior parietal association areas	39.18 ± 12.52	15	50.76 ± 23.16	15	62.21 ± 23.09	10	158.60 ± 46.08	9	1.64 [-1.77, 5.05]	2.30 [-1.27, 5.99]	-3.29 [-8.11, 1.49]						
L 22	L 315	L Posterior parietal association areas	5.44 ± 1.80	14	3.93 ± 1.48	15	14.75 ± 2.94	10	45.64 ± 27.99	10	6.57 [3.4, 9.86]*	5.52 [2.01, 9.05]*	-7.08 [-11.8, -2.48]*	0.11 [-2.73, 2.96]	-1.93 [-5.2, 1.26]	-7.43 [-10.48, -4.24]*	2.04 [-1.01, 5.3]	-7.52 [-10.76, -4.6]*	-5.47 [-8.97, -2.13]*
R 312782546	R 22	R Anterior area	46.77 ± 13.33	15	92.78 ± 40.73	15	110.63 ± 45.89	10	266.65 ± 97.38	9	2.61 [-0.71, 5.95]	3.18 [-0.44, 6.76]	-3.49 [-8.3, 1.26]						
L 312782546	L 22	L Anterior area	6.81 ± 2.56	15	5.89 ± 1.98	15	23.24 ± 4.54	10	28.46 ± 6.20	9	2.82 [-0.5, 6.26]	1.22 [-2.44, 4.81]	-2.67 [-7.48, 2.11]						
R 417	R 22	R Rostrolateral visual area	50.15 ± 21.36	14	25.85 ± 10.34	15	48.28 ± 12.49	10	196.09 ± 73.10	9	2.29 [-1, 5.59]	2.78 [-0.82, 6.43]	-4.50 [-9.21, 0.23]						
L 417	L 22	L Rostrolateral visual area	7.56 ± 2.93	14	6.39 ± 2.96	14	20.30 ± 5.14	10	81.81 ± 52.96	8	9.00 [5.53, 12.47]*	8.35 [4.58, 12.02]*	-9.51 [-14.43, -4.65]*	-0.30 [-3.21, 2.58]	-1.95 [-5.14, 1.21]	-10.24 [-13.59, -6.83]*	1.64 [-1.59, 4.73]	-9.95 [-13.24, -6.52]*	-8.32 [-12.01, -4.79]*
R 541	R 315	R Temporal association areas	23.43 ± 6.28	13	19.91 ± 7.06	12	26.26 ± 4.11	10	34.78 ± 4.41	7	0.13 [-3.59, 3.84]	0.98 [-2.91, 4.87]	-2.21 [-7.3, 2.93]						
L 541	L 315	L Temporal association areas	12.21 ± 5.50	13	7.10 ± 2.46	15	19.34 ± 2.76	10	53.15 ± 21.86	10	2.67 [-0.6, 5.95]	2.61 [-0.82, 6.16]	-4.25 [-9.02, 0.41]						
R 922	R 315	R Perirhinal area	49.67 ± 29.68	14	28.17 ± 10.75	13	24.22 ± 8.63	10	81.15 ± 23.26	8	0.73 [-2.8, 4.27]	1.67 [-2.04, 5.36]	-3.42 [-8.31, 1.41]						
L 922	L 315	L Perirhinal area	11.83 ± 4.85	14	4.56 ± 1.70	15	22.29 ± 6.38	10	19.37 ± 4.45	10	0.17 [-3.08, 3.46]	-0.41 [-3.94, 3.18]	-1.41 [-6.13, 3.36]						
R 895	R 315	R Ectorhinal area	22.00 ± 8.20	14	19.99 ± 6.73	13	28.60 ± 4.77	10	51.25 ± 8.81	8	1.10 [-2.47, 4.66]	1.54 [-2.22, 5.3]	-2.97 [-7.92, 1.98]						
L 895	L 315	L Ectorhinal area	14.63 ± 8.02	14	3.28 ± 1.03	15	18.47 ± 5.31	10	30.13 ± 6.17	10	0.72 [-2.52, 3.92]	0.63 [-2.86, 4.16]	-2.65 [-7.32, 2.01]						
R 698	R 695	R Olfactory areas	18.34 ± 7.02	14	10.86 ± 1.78	14	23.93 ± 5.68	12	35.38 ± 5.97	10	-0.39 [-2.97, 2.16]	-0.07 [-2.49, 2.39]	0.13 [-3.65, 3.89]						
L 698	L 695	L Olfactory areas	13.76 ± 4.55	14	6.69 ± 0.99	14	15.84 ± 2.56	12	31.83 ± 5.50	10	-0.56 [-3.1, 2.03]	-0.19 [-2.6, 2.26]	0.8 [-3.02, 4.48]						
L 159	L 698	L Anterior olfactory nucleus	18.65 ± 8.79	8	8.35 ± 2.50	7	89.98 ± 22.41	6	138.62 ± 27.32	6	2.63 [-0.72, 5.96]	-0.46 [-3.59, 2.8]	3.57 [-1.27, 8.4]						
R 589	R 698	R Taenia tecta	9.34 ± 2.50	12	9.79 ± 2.38	9	62.86 ± 22.79	7	68.89 ± 34.04	8	4.16 [1.16, 7.14]*	-0.13 [-2.91, 2.62]	1.04 [-3.3, 5.36]	0.31 [-2.37, 2.98]	-4.95 [-7.94, -2.13]*	-6.75 [-9.47, -3.73]*	5.27 [2.33, 8.34]*	-7.06 [-10.06, -4.05]*	-1.78 [-4.86, 1.53]
L 589	L 698	L Taenia tecta	5.38 ± 1.42	12	5.09 ± 1.71	9	27.37 ± 7.68	8	64.79 ± 30.95	8	2.65 [-0.19, 5.48]	-0.25 [-3.03, 2.54]	7.3 [3.06, 11.48]*	0.44 [-2.29, 3.1]	-3.43 [-6.18, -0.64]*	-11.37 [-14.18, -8.49]*	3.87 [0.96, 6.93]*	-11.81 [-14.78, -8.71]*	-7.9 [-11.02, -4.85]*
R 597	R 589	R Taenia tecta, dorsal part	15.21 ± 4.11	12	25.67 ± 6.64	8	140.24 ± 59.50	7	137.62 ± 68.10	8	6.62 [3.64, 9.59]*	0.55 [-2.25, 3.38]	-0.47 [-4.85, 3.87]	-0.35 [-3.08, 2.42]	-7.44 [-10.26, -4.49]*	-8.38 [-11.15, -5.39]*	7.08 [4.05, 10.23]*	-8.02 [-11.01, -4.92]*	-0.96 [-4.28, 2.11]
L 597	L 589	L Taenia tecta, dorsal part	10.92 ± 2.91	12	11.59 ± 3.97	8	59.20 ± 16.77	8	129.11 ± 62.04	8	2.98 [0.04, 5.93]*	-0.08 [-2.98, 2.87]	6.58 [2.18, 10.99]*	0.27 [-2.5, 3.13]	-3.8 [-6.59, -0.93]*	-11.15 [-14.15, -8.35]*	4.05 [1.04, 7.15]*	-11.41 [-14.59, -8.46]*	-7.38 [-10.48, -4.12]*
R 814	R 698	R Dorsal peduncular area	20.10 ± 8.89	14	22.98 ± 6.95	8	54.90 ± 18.17	6	90.51 ± 31.69	8	1.1 [-1.97, 4.17]	0.15 [-2.61, 2.99]	1.05 [-3.39, 5.46]						
L 814	L 698	L Dorsal peduncular area	12.17 ± 2.80	13	29.03 ± 10.53	8	34.47 ± 13.42	6	103.29 ± 50.58	8	1.22 [-1.79, 4.3]	1.41 [-1.46, 4.2]	3.69 [-0.79, 8.21]						
R 961	R 698	R Piriform area	51.78 ± 23.45	10	36.69 ± 5.87	10	65.44 ± 19.47	7	78.16 ± 14.62	10	-0.07 [-3.14, 3.04]	0.01 [-2.79, 2.9]	-0.71 [-4.95, 3.57]						
L 961	L 698	L Piriform area	41.73 ± 11.14	12	36.06 ± 13.48	10	41.49 ± 7.31	8	66.44 ± 14.62	10	-0.33 [-3.19, 2.61]	0.19 [-2.58, 2.93]	-0.46 [-4.59, 3.66]						
R 631	R 698	R Cortical amygdalar area	20.26 ± 7.06	14	7.22 ± 1.45	11	19.74 ± 7.48	9	23.57 ± 3.80	10	-0.25 [-3.02, 2.51]	-0.27 [-2.81, 2.31]	-0.54 [-4.49, 3.38]						
L 631	L 698	L Cortical amygdalar area	9.08 ± 4.90	14	6.53 ± 1.94	12	9.45 ± 1.94	9	22.45 ± 5.46	10	0 [-2.73, 2.72]	-0.03 [-2.61, 2.46]	0.2 [-3.7, 4.08]						
R 639	R 631	R Cortical amygdalar area, anterior part	46.91 ± 19.68	14	24.76 ± 6.14	10	74.29 ± 32.14	9	45.71 ± 8.80	10	0.35 [-2.38, 3.07]	-0.12 [-2.79, 2.53]	-1.48 [-5.49, 2.45]						
L 639	L 631	L Cortical amygdalar area, anterior part	29.24 ± 16.45	14	12.46 ± 2.80	11	45.04 ± 9.23	9	86.95 ± 23.30	10	0.49 [-2.21, 3.29]	-0.29 [-2.86, 2.31]	0.47 [-3.57, 4.37]						
R 647	R 631	R Cortical amygdalar area, posterior part	29.81 ± 11.85	12	10.08 ± 2.54	12	22.09 ± 9.94	9	30.12 ± 7.01	8	-0.46 [-3.27, 2.32]	-0.35 [-2.91, 2.25]	-0.55 [-4.58, 3.56]						
L 647	L 631	L Cortical amygdalar area, posterior part	12.20 ± 6.80	13	8.41 ± 3.13	12	5.50 ± 1.52	8	26.65 ± 7.97	9	-0.53 [-3.34, 2.34]	-0.05 [-2.69, 2.52]	0.22 [-3.95, 4.3]						
R 655	R 647	R Cortical amygdalar area, posterior part, lateral zone	64.74 ± 25.38	13	24.92 ± 7.26	12	46.82 ± 17.49	9	69.03 ± 17.24	8	-0.51 [-3.29, 2.26]	-0.35 [-2.93, 2.22]	-0.5 [-4.52, 3.61]						
L 655	L 647	L Cortical amygdalar area, posterior part, lateral zone	22.05 ± 11.43	13	15.43 ± 7.18	12	13.23 ± 3.21	8	62.89 ± 19.48	9	-0.37 [-3.24, 2.53]	-0.03 [-2.54, 2.56]	0.71 [-3.4, 4.72]						
R 663	R 647	R Cortical amygdalar area, posterior part, medial zone	50.29 ± 22.20	11	14.53 ± 3.42	12	47.42 ± 26.24	8	49.42 ± 12.75	8	-0.3 [-3.19, 2.7]	-0.38 [-3.05, 2.3]	-0.7 [-4.87, 3.51]						
L 663	L 647	L Cortical amygdalar area, posterior part, medial zone	28.58 ± 19.10	11	17.71 ± 9.68	12	8.65 ± 3.66	8	38.29 ± 15.09	9	-0.62 [-3.61, 2.32]	-0.05 [-2.72, 2.54]	-0.48 [-4.62, 3.65]						
R 788	R 698	R Piriform-amygdalar area	50.03 ± 17.37	15	30.51 ± 8.44	11	54.78 ± 13.06	10	61.20 ± 12.39	10									
L 788	L 698	L Piriform-amygdalar area	24.69 ± 10.40	15	16.54 ± 6.42	14	33.19 ± 7.27	10	59.63 ± 18.51	10	0.07 [-2.6, 2.7]	-0.04 [-2.49, 2.39]	0.1 [-3.7, 3.93]						
R 1089	R 695	R Hippocampal formation	76.17 ± 21.82	15	113.86 ± 24.92	15	307.68 ± 54.66	12	288.09 ± 66.01	10	2.14 [-0.34, 4.63]	0.69 [-1.59, 2.99]	-1.51 [-5.09, 2.05]						
L 1089	L 695	L Hippocampal formation	13.31 ± 3.98	15	16.43 ± 3.07	15	57.00 ± 13.93	12	60.94 ± 10.31	10	2.38 [-0.05, 4.83]	0.43 [-1.85, 2.74]	-0.69 [-4.29, 2.9]						
R 1080	R 1089	R Hippocampal region	102.19 ± 30.45	15	169.93 ± 33.74	15	413.26 ± 72.46	12	388.38 ± 95.07	10	2.14 [-0.32, 4.59]	0.85 [-1.48, 3.16]	-1.65 [-5.18, 1.93]						
L 1080	L 1089	L Hippocampal region	17.95 ± 5.52	15	24.50 ± 4.58	15	76.30 ± 19.02	12	79.64 ± 14.85	10	2.35 [-0.08, 4.84]	0.57 [-1.68, 2.9]	-0.92 [-4.5, 2.58]						
R 375	R 1080	R Ammon's horn	141.91 ± 46.22	15	218.54 ± 45.48	15	489.25 ± 85.73	12	503.46 ± 115.49	10	1.54 [-0.89, 3.95]	0.72 [-1.52, 2.98]	-1.17 [-4.64, 2.34]						
L 375	L 1080	L Ammon's horn	22.27 ± 6.57	15	35.81 ± 7.65	15	97.57 ± 21.92	12	106.24 ± 19.19	10	2.49 [0.07, 4.93]*	0.82 [-1.51, 3.14]	-0.97 [-4.52, 2.57]	-0.65 [-2.89, 1.58]	-3.37 [-5.71, -0.95]*	-4.23 [-6.65, -1.6]*	2.68 [0.22, 5.04]*	-3.57 [-6.07, -0.99]*	-0.89 [-3.48, 1.83]
R 382	R 375	R Field CA1	254.69 ± 91.37	15	361.42 ± 72.10	15	856.87 ± 149.04	12	811.05 ± 164.63	10	1.47 [-0.98, 3.9]	0.62 [-1.65, 2.89]	-1.35 [-4.98, 2.24]						
L 382	L 375	L Field CA1	39.81 ± 13.45	15	74.95 ± 18.36	15	236.04 ± 46.87	12	232.86 ± 38.55	10	4.04 [1.64, 6.47]*	1.07 [-1.19, 3.36]	-1.71 [-5.3, 1.82]	-0.92 [-3.07, 1.4]	-4.91 [-7.35, -2.62]*	-5.31 [-7.84, -2.71]*	3.99 [1.73, 6.51]*	-4.39 [-7, -1.87]*	-0.39 [-3.03, 2.33]
R 423	R 375	R Field CA2	415.99 ± 119.59	15	508.83 ± 108.52	15	1121.75 ± 168.86	12	1077.28 ± 296.20	10	0.81 [-1.66, 3.29]	0.42 [-1.93, 2.74]	-1.08 [-4.64, 2.52]						
L 423	L 375	L Field CA2	42.46 ± 14.34	15	47.93 ± 13.61	15	148.84 ± 44.92	12	189.78 ± 56.18	9	1.61 [-0.89, 4.04]	0.34 [-2, 2.66]	-0.06 [-3.66, 3.57]						
R 463	R 375	R Field CA3	316.25 ± 107.59	14	547.25 ± 133.79	15	1116.28 ± 213.95	12	1130.61 ± 306.88	10	1.47 [-0.96, 3.95]	0.76 [-1.56, 3.07]	-1.28 [-4.87, 2.3]						
L 463	L 375	L Field CA3	49.49 ± 15.67	15	70.68 ± 14.52	15	158.04 ± 42.60	12	206.46 ± 41.21	9	1.3 [-1.14, 3.76]	0.63 [-1.67, 2.9]	-0.35 [-3.97, 3.26]						
R 726	R 1080	R Dentate gyrus	189.20 ± 55.75	15	362.60 ± 77.45	15	1016.85 ± 191.95	12	849.39 ± 251.92	10	3.48 [1.02, 5.93]*	1.1 [-1.2, 3.39]	-2.56 [-6.11, 0.98]	-0.94 [-3.2, 1.34]	-4.34 [-6.69, -1.97]*	-3.92 [-6.37, -1.28]*	3.4 [1.07, 5.84]*	-2.98 [-5.64, -0.52]*	0.43 [-2.2, 3.08]
L 726	L 1080	L Dentate gyrus	38.56 ± 13.38	15	39.91 ± 9.02	15	172.39 ± 57.24	11	156.31 ± 32.65	10	2.62 [0.14, 5.13]*	0.22 [-2.08, 2.52]	-1.25 [-4.89, 2.41]	-0.06 [-2.26, 2.23]	-3.49 [-5.79, -0.92]*	-3.49 [-6.03, -0.87]*	3.42 [1, 5.86]*	-3.43 [-5.99, -0.84]*	0 [-2.64, 2.83]
R 10703	R 726	R Dentate gyrus, molecular layer	367.17 ± 114.58	15	636.27 ± 152.08	15	1744.62 ± 320.17	12	1496.69 ± 417.39	10	2.86 [0.44, 5.37]*	0.93 [-1.33, 3.18]	-2.17 [-5.71, 1.37]	-0.78 [-3.02, 1.41]	-3.71 [-6.14, -1.36]*	-3.53 [-6.01, -0.89]*	2.96 [0.49, 5.24]*	-2.75 [-5.17, -0.13]*	0.22 [-2.46, 2.86]
L 10703	L 726	L Dentate gyrus, molecular layer	77.49 ± 25.82	15	66.72 ± 15.46	15	313.35 ± 96.93	11	271.15 ± 53.22	10	2.19 [-0.27, 4.68]	0.05 [-2.22, 2.31]	-1.2 [-4.8, 2.33]						
R 10704	R 726	R Dentate gyrus, polymorph layer	478.71 ± 137.74	15	1153.91 ± 259.60	15	2797.94 ± 545.16	12	2472.65 ± 823.91	10	3.96 [1.58, 6.45]*	1.62 [-0.68, 3.92]	-2.86 [-6.42, 0.69]	-1.46 [-3.72, 0.73]	-4.83 [-7.24, -2.46]*	-4.62 [-7.31, -2.11]*	3.36 [1.12, 5.82]*	-3.16 [-5.8, -0.69]*	0.2 [-2.59, 2.79]
L 10704	L 726	L Dentate gyrus, polymorph layer	98.11 ± 46.41	15	142.45 ± 34.99	15	332.78 ± 133.74	11	465.18 ± 110.73	10	1.53 [-0.95, 4.03]	0.63 [-1.67, 2.9]	0.12 [-3.51, 3.7]						
R 632	R 726	R Dentate gyrus, granule cell layer	373.29 ± 107.26	15	793.64 ± 159.74	15	2510.59 ± 551.19	12	1950.43 ± 627.18	10	4.84 [2.4, 7.28]*	1.32 [-0.94, 3.66]	-3.39 [-6.98, 0.1]	-1.16 [-3.36, 1.15]	-5.71 [-8.17, -3.41]*	-4.66 [-7.18, -2.07]*	4.54 [2.16, 6.91]*	-3.5 [-5.98, -0.97]*	1.04 [-1.7, 3.57]
L 632	L 726	L Dentate gyrus, granule cell layer	69.34 ± 25.97	15	91.38 ± 25.63	15	426.23 ± 160.88	11	374.11 ± 90.58	10	4.28 [1.82, 6.78]*	0.51 [-1.77, 2.8]	-1.86 [-5.43, 1.72]	-0.35 [-2.73, 1.77]	-5.15 [-7.56, -2.73]*	-4.83 [-7.38, -2.23]*	4.81 [2.3, 7.24]*	-4.47 [-7.07, -1.92]*	0.3 [-2.41, 2.96]
R 982	R 1080	R Fasciola cinerea	484.18 ± 230.22	13	692.89 ± 216.02	15	2064.50 ± 503.44	11	1579.94 ± 651.17	8	2.44 [-0.08, 4.99]	0.72 [-1.62, 3.07]	-2.14 [-5.88, 1.6]						
L 982	L 1080	L Fasciola cinerea	201.26 ± 60.95	13	235.22 ± 60.88	15	665.18 ± 283.96	11	658.64 ± 241.10	9	1.45 [-1.06, 3.95]	0.46 [-1.9, 2.82]	-0.94 [-4.57, 2.68]						
R 19	R 1080	R Induseum griseum	66.55 ± 20.23	15	78.42 ± 25.34	12	235.81 ± 80.92	12	159.08 ± 57.06	10	1.66 [-0.77, 4.11]	0.35 [-2.04, 2.71]	-2.07 [-5.69, 1.61]						
L 19	L 1080	L Induseum griseum	40.36 ± 16.37	15	88.73 ± 38.72	12	54.06 ± 14.72	12	142.48 ± 37.23	10	-0.55 [-3, 1.91]	1.36 [-0.99, 3.74]	0.27 [-3.43, 3.83]						
R 822	R 1089	R Retrohippocampal region	76.27 ± 31.20	14	71.30 ± 34.44	13	414.84 ± 184.98	12	306.12 ± 78.27	8	3.61 [1.17, 6.02]*	0.21 [-2.16, 2.62]	-2.21 [-5.87, 1.43]	-0.03 [-2.31, 2.36]	-4.49 [-6.81, -2.11]*	-3.52 [-6.3, -0.87]*	4.42 [1.97, 6.9]*	-3.47 [-6.25, -0.83]*	0.95 [-1.73, 3.8]
L 822	L 1089	L Retrohippocampal region	10.48 ± 3.52	14	8.80 ± 2.88	15	96.91 ± 53.50	12	68.76 ± 14.20	9	7.42 [4.97, 9.95]*	0.1 [-2.23, 2.41]	-3.41 [-7.08, 0.23]	0.06 [-2.16, 2.46]	-8.27 [-10.81, -5.92]*	-6.03 [-8.6, -3.36]*	8.34 [5.93, 10.72]*	-6.07 [-8.67, -3.53]*	2.25 [-0.61, 4.89]
R 909	R 822	R Entorhinal area	33.81 ± 14.20	13	23.78 ± 7.10	12	39.52 ± 23.14	10	50.79 ± 14.55	8									
L 909	L 822	L Entorhinal area	7.12 ± 3.36	14	5.27 ± 2.28	15	10.23 ± 3.05	10	16.21 ± 4.33	9	0.25 [-2.3, 2.81]	0 [-2.37, 2.34]	-0.42 [-4.1, 3.32]						
R 918	R 909	R Entorhinal area, lateral part	52.33 ± 21.96	13	38.34 ± 12.27	12	60.87 ± 35.61	10	78.32 ± 22.42	8	0 [-2.59, 2.57]	-0.02 [-2.48, 2.45]	-0.85 [-4.67, 2.98]						
L 918	L 909	L Entorhinal area, lateral part	11.03 ± 5.20	14	7.66 ± 3.52	15	15.78 ± 4.71	10	25.05 ± 6.70	9	0.24 [-2.29, 2.84]	-0.05 [-2.36, 2.29]	-0.4 [-4.08, 3.28]						
R 502	R 822	R Subiculum	457.85 ± 173.38	12	463.21 ± 212.07	10	2475.96 ± 853.31	10	2339.07 ± 608.71	7	3.55 [0.97, 6.15]*	0.19 [-2.4, 2.72]	-0.72 [-4.71, 3.25]	-0.04 [-2.48, 2.54]	-4.42 [-7, -1.95]*	-4.92 [-7.79, -2.06]*	4.38 [1.72, 7.02]*	-4.89 [-7.94, -2.11]*	-0.49 [-3.49, 2.46]
L 502	L 822	L Subiculum	105.07 ± 47.75	12	49.79 ± 29.39	11	546.74 ± 158.41	10	651.54 ± 87.13	7	3.33 [0.7, 5.93]*	-0.26 [-2.79, 2.23]	1.02 [-2.96, 4.91]	0.42 [-2.13, 2.79]	-4.2 [-6.78, -1.7]*	-5.98 [-8.84, -3.04]*	4.62 [2.06, 7.27]*	-6.42 [-9.25, -3.33]*	-1.79 [-4.72, 1.22]
R 484682470	R 822	R Prosubiculum	1001.71 ± 472.42	10	806.08 ± 417.98	9	2890.09 ± 912.18	10	2521.16 ± 673.63	7	1.15 [-1.5, 3.9]	0.06 [-2.63, 2.82]	-0.66 [-4.67, 3.36]						
L 484682470	L 822	L Prosubiculum	25.39 ± 11.06	10	86.19 ± 58.03	10	527.25 ± 149.10	10	669.50 ± 104.13	7	18.72 [16, 21.45]*	2.39 [-0.34, 5.06]	2.97 [-1.01, 6.95]	-2.24 [-4.81, 0.44]	-19.59 [-22.19, -16.86]*	-25.98 [-29.05, -23.12]*	17.36 [14.69, 20.04]*	-23.74 [-26.69, -20.9]*	-6.38 [-9.28, -3.37]*
R 703	R 688	R Cortical subplate	11.67 ± 5.27	15	7.44 ± 2.25	15	33.72 ± 6.72	12	44.85 ± 5.86	10	1.78 [0.03, 3.62]*	-0.14 [-1.8, 1.53]	-0.03 [-2.62, 2.57]	0.21 [-1.42, 1.91]	-2.36 [-4.17, -0.64]*	-3.33 [-5.3, -1.42]*	2.59 [0.8, 4.36]*	-3.54 [-5.56, -1.69]*	-0.97 [-2.88, 1.11]
L 703	L 688	L Cortical subplate	9.13 ± 6.04	15	5.66 ± 1.49	15	23.45 ± 3.00	12	40.15 ± 6.94	10	1.46 [-0.28, 3.22]	-0.17 [-1.81, 1.49]	0.87 [-1.76, 3.43]						
R 583	R 703	R Claustrum	31.89 ± 7.27	15	17.22 ± 5.61	14	93.93 ± 30.71	12	69.55 ± 13.71	10	1.84 [0.09, 3.61]*	-0.16 [-1.84, 1.48]	-1.73 [-4.29, 0.89]	0.23 [-1.43, 1.89]	-2.43 [-4.13, -0.65]*	-1.68 [-3.69, 0.14]	2.66 [0.86, 4.4]*	-1.91 [-3.85, 0]	0.76 [-1.32, 2.67]
L 583	L 703	L Claustrum	7.28 ± 3.41	15	24.59 ± 11.25	14	71.94 ± 18.02	12	74.03 ± 21.49	10	8.78 [7.02, 10.53]*	2.68 [1.01, 4.37]*	-3.52 [-6.1, -0.89]*	-2.61 [-4.31, -0.97]*	-9.36 [-11.08, -7.64]*	-9.64 [-11.67, -7.81]*	6.75 [4.96, 8.52]*	-7.05 [-9.03, -5.12]*	-0.3 [-2.28, 1.69]
R 942	R 703	R Endopiriform nucleus	8.90 ± 1.87	13	8.70 ± 1.80	13	31.80 ± 8.11	12	46.38 ± 8.63	10	1.98 [0.15, 3.8]*	-0.17 [-1.89, 1.56]	0.67 [-1.95, 3.34]	0.23 [-1.48, 1.97]	-2.58 [-4.33, -0.76]*	-4.2 [-6.18, -2.29]*	2.8 [1, 4.69]*	-4.45 [-6.41, -2.5]*	-1.65 [-3.69, 0.33]
L 942	L 703	L Endopiriform nucleus	9.26 ± 5.30	14	12.28 ± 5.18	15	23.68 ± 4.72	12	36.81 ± 6.88	10	1.5 [-0.3, 3.31]	0.58 [-1.06, 2.3]	-0.3 [-2.89, 2.3]						
R 952	R 942	R Endopiriform nucleus, dorsal part	18.61 ± 5.22	13	21.35 ± 6.25	14	59.39 ± 13.09	12	79.66 ± 18.40	10	1.59 [-0.18, 3.43]	-0.16 [-1.87, 1.58]	0.11 [-2.52, 2.69]						
L 952	L 942	L Endopiriform nucleus, dorsal part	16.66 ± 8.93	14	21.67 ± 5.76	15	38.13 ± 6.83	12	60.91 ± 13.99	10	1.23 [-0.59, 3.06]	0.55 [-1.17, 2.25]	-0.32 [-2.95, 2.35]						
R 966	R 942	R Endopiriform nucleus, ventral part	16.14 ± 3.39	13	35.54 ± 15.88	14	70.02 ± 22.92	12	114.22 ± 17.44	10	2.74 [0.91, 4.6]*	0.89 [-0.86, 2.62]	0.72 [-1.93, 3.36]	-0.82 [-2.64, 0.82]	-3.32 [-5.15, -1.5]*	-6.09 [-8.06, -4.13]*	2.51 [0.72, 4.29]*	-5.26 [-7.16, -3.28]*	-2.75 [-4.81, -0.78]*
L 966	L 942	L Endopiriform nucleus, ventral part	21.66 ± 13.40	14	28.32 ± 20.09	15	63.03 ± 14.33	12	95.95 ± 16.36	10	1.85 [0.09, 3.62]*	0.56 [-1.08, 2.22]	-0.17 [-2.72, 2.34]	-0.49 [-2.09, 1.15]	-2.44 [-4.19, -0.69]*	-3.96 [-5.95, -2.04]*	1.94 [0.17, 3.71]*	-3.46 [-5.44, -1.58]*	-1.53 [-3.53, 0.46]
R 131	R 703	R Lateral amygdalar nucleus	35.39 ± 15.44	13	20.01 ± 8.78	15	70.01 ± 14.09	12	56.87 ± 10.71	10	0.99 [-0.78, 2.81]	-0.11 [-1.8, 1.59]	-1.38 [-4.01, 1.22]						
L 131	L 703	L Lateral amygdalar nucleus	17.70 ± 9.49	14	15.93 ± 5.01	15	46.27 ± 14.90	12	54.01 ± 12.70	10	1.58 [-0.21, 3.38]	0.19 [-1.51, 1.85]	-0.87 [-3.5, 1.7]						
R 295	R 703	R Basolateral amygdalar nucleus	17.99 ± 11.30	15	5.41 ± 1.01	13	71.25 ± 11.46	10	98.08 ± 12.73	10	3.16 [1.36, 4.97]*	-0.34 [-2, 1.33]	0.4 [-2.19, 3.06]	0.41 [-1.25, 2.09]	-3.76 [-5.48, -1.89]*	-4.96 [-6.77, -2.89]*	4.16 [2.25, 6.02]*	-5.37 [-7.31, -3.38]*	-1.2 [-3.25, 0.86]
L 295	L 703	L Basolateral amygdalar nucleus	14.02 ± 10.08	14	5.39 ± 1.44	13	67.14 ± 5.24	10	90.75 ± 12.86	10	4.09 [2.25, 5.94]*	-0.35 [-2.06, 1.35]	0.54 [-2.14, 3.17]	0.43 [-1.33, 2.08]	-4.66 [-6.53, -2.91]*	-6.01 [-7.98, -4.01]*	5.1 [3.14, 6.91]*	-6.42 [-8.4, -4.43]*	-1.34 [-3.39, 0.8]
R 303	R 295	R Basolateral amygdalar nucleus, anterior part	27.41 ± 12.59	15	10.53 ± 3.20	12	182.11 ± 29.04	10	232.34 ± 33.97	10	5.86 [4.06, 7.72]*	-0.23 [-2.01, 1.53]	0.61 [-2.17, 3.31]	0.3 [-1.44, 2.06]	-6.45 [-8.27, -4.63]*	-7.98 [-9.95, -6.06]*	6.75 [4.9, 8.65]*	-8.27 [-10.2, -6.25]*	-1.53 [-3.64, 0.51]
L 303	L 295	L Basolateral amygdalar nucleus, anterior part	27.70 ± 17.97	14	15.37 ± 4.75	13	161.03 ± 13.94	10	230.26 ± 35.55	10	5.11 [3.26, 6.99]*	-0.18 [-1.89, 1.51]	1.19 [-1.51, 3.82]	0.25 [-1.37, 2]	-5.7 [-7.49, -3.8]*	-7.85 [-9.79, -5.87]*	5.94 [4.03, 7.8]*	-8.1 [-10.17, -6.16]*	-2.16 [-4.22, -0.04]*
R 311	R 295	R Basolateral amygdalar nucleus, posterior part	33.62 ± 26.51	14	11.39 ± 3.17	12	116.90 ± 37.54	10	184.86 ± 17.21	8	2.76 [0.9, 4.62]*	-0.25 [-2, 1.47]	0.85 [-1.87, 3.62]	0.3 [-1.43, 2.05]	-3.37 [-5.22, -1.55]*	-5.09 [-7.2, -3.1]*	3.68 [1.83, 5.61]*	-5.42 [-7.52, -3.33]*	-1.73 [-3.87, 0.4]
L 311	L 295	L Basolateral amygdalar nucleus, posterior part	17.26 ± 9.77	13	8.51 ± 3.80	13	103.56 ± 22.26	9	161.51 ± 32.43	9	5.26 [3.36, 7.17]*	-0.16 [-1.87, 1.56]	2.02 [-0.69, 4.73]	0.24 [-1.49, 1.89]	-5.86 [-7.64, -3.89]*	-8.85 [-10.81, -6.85]*	6.08 [4.15, 7.97]*	-9.08 [-11.09, -7.15]*	-3 [-5.14, -0.9]*
R 451	R 295	R Basolateral amygdalar nucleus, ventral part	71.38 ± 58.75	14	8.95 ± 3.05	11	93.49 ± 13.87	9	137.15 ± 29.84	8	0.6 [-1.29, 2.45]	-0.44 [-2.21, 1.31]	-0.39 [-3.08, 2.39]						
L 451	L 295	L Basolateral amygdalar nucleus, ventral part	51.17 ± 47.51	12	4.39 ± 2.48	12	74.52 ± 19.59	8	114.95 ± 23.46	9	0.79 [-1.17, 2.76]	-0.5 [-2.29, 1.28]	-0.23 [-3.06, 2.55]						
R 319	R 703	R Basomedial amygdalar nucleus	16.79 ± 8.23	14	5.79 ± 1.38	13	58.64 ± 12.08	10	55.45 ± 10.92	10	2.62 [0.76, 4.45]*	-0.14 [-1.87, 1.56]	-1.39 [-4.06, 1.27]	0.2 [-1.49, 1.89]	-3.2 [-5.07, -1.42]*	-2.83 [-4.69, -0.73]*	3.41 [1.55, 5.34]*	-3.05 [-5.03, -1.11]*	0.38 [-1.73, 2.41]
L 319	L 703	L Basomedial amygdalar nucleus	17.86 ± 13.73	15	4.98 ± 1.81	13	30.35 ± 6.79	11	61.05 ± 13.40	10	0.95 [-0.82, 2.75]	-0.48 [-2.17, 1.23]	0.71 [-1.87, 3.31]						

R 327	R 319	R Basomedial amygdalar nucleus, anterior part	28.67 ± 9.27	14	15.31 ± 5.53	12	80.67 ± 27.17	10	68.09 ± 11.54	10	1.94 [0.1, 3.75]*	0.08 [-1.67, 1.81]	-1.84 [-4.45, 0.86]	-0.01 [-1.73, 1.66]	-2.53 [-4.35, -0.76]*	-1.91 [-3.78, 0.09]	2.52 [0.62, 4.37]*	-1.89 [-3.96, 0.03]	0.63 [-1.49, 2.63]
L 327	L 319	L Basomedial amygdalar nucleus, anterior part	23.09 ± 16.70	13	7.29 ± 2.46	11	71.46 ± 22.20	11	86.26 ± 21.42	10	2.35 [0.59, 4.15]*	-0.31 [-2.07, 1.43]	-0.55 [-3.19, 2.08]	0.37 [-1.36, 2.08]	-2.95 [-4.7, -1.2]*	-3.23 [-5.1, -1.23]*	3.31 [1.54, 5.25]*	-3.59 [-5.59, -1.62]*	-0.28 [-2.25, 1.75]
R 334	R 319	R Basomedial amygdalar nucleus, posterior part	38.57 ± 27.60	13	8.97 ± 1.99	11	163.37 ± 31.18	9	158.56 ± 38.99	8	3.3 [1.41, 5.24]*	-0.2 [-1.97, 1.57]	-1.1 [-3.92, 1.68]	0.27 [-1.48, 2.08]	-3.88 [-5.77, -2.03]*	-3.73 [-5.69, -1.6]*	4.16 [2.14, 6.05]*	-3.99 [-6.03, -1.86]*	0.16 [-1.98, 2.37]
L 334	L 319	L Basomedial amygdalar nucleus, posterior part	50.46 ± 39.91	14	8.44 ± 3.82	13	56.75 ± 12.04	9	160.46 ± 34.00	9	0.15 [-1.71, 2.05]	-0.52 [-2.22, 1.21]	1.25 [-1.48, 3.98]						
R 780	R 703	R Posterior amygdalar nucleus	29.70 ± 16.76	12	29.28 ± 11.34	12	73.91 ± 27.78	10	124.88 ± 27.95	8	1.17 [-0.71, 3.03]	0.48 [-1.31, 2.24]	0.48 [-2.25, 3.26]						
L 780	L 703	L Posterior amygdalar nucleus	32.66 ± 22.10	12	17.03 ± 9.75	13	22.03 ± 10.62	10	53.02 ± 16.70	9									
R 477	R 623	R Striatum	11.03 ± 2.39	15	10.88 ± 1.47	15	36.84 ± 5.41	12	40.83 ± 3.92	10	1.58 [-2.06, 5.12]	0.05 [-3.3, 3.41]	-0.88 [-6.1, 4.55]						
L 477	L 623	L Striatum	9.90 ± 3.18	15	13.25 ± 2.64	15	25.06 ± 3.17	12	32.84 ± 2.44	10	0.76 [-2.85, 4.36]	0.42 [-2.99, 3.8]	-0.79 [-6.18, 4.53]						
R 485	R 477	R Striatum dorsal region	11.71 ± 1.98	15	11.62 ± 1.82	15	30.11 ± 5.23	12	28.73 ± 5.48	10	0.79 [-2.69, 4.34]	0.06 [-3.28, 3.46]	-1.32 [-6.66, 3.88]						
L 485	L 477	L Striatum dorsal region	9.98 ± 1.91	15	20.59 ± 6.41	15	18.39 ± 3.30	12	23.70 ± 5.47	10	0.07 [-3.53, 3.67]	1.14 [-2.25, 4.51]	-1.76 [-6.98, 3.61]						
R 672	R 485	R Caudoputamen	25.08 ± 3.86	14	24.89 ± 3.49	14	60.22 ± 10.46	12	63.83 ± 9.96	9	0.52 [-3.09, 4.18]	0.08 [-3.49, 3.55]	-0.94 [-6.42, 4.52]						
L 672	L 485	L Caudoputamen	23.02 ± 3.71	13	41.18 ± 12.82	15	36.77 ± 6.59	12	52.66 ± 10.72	9	-0.1 [-3.75, 3.52]	0.94 [-2.5, 4.4]	-1.25 [-6.6, 4.02]						
R 493	R 477	R Striatum ventral region	18.55 ± 4.39	15	16.00 ± 2.36	14	58.79 ± 6.41	12	62.97 ± 9.01	10	1.39 [-2.19, 4.9]	-0.04 [-3.5, 3.41]	-0.89 [-6.14, 4.33]						
L 493	L 477	L Striatum ventral region	17.36 ± 4.85	15	18.10 ± 3.50	14	60.48 ± 6.20	12	67.55 ± 13.23	10	1.7 [-1.81, 5.23]	0.14 [-3.28, 3.58]	-0.89 [-6.24, 4.34]						
R 56	R 493	R Nucleus accumbens	28.64 ± 6.26	12	22.27 ± 1.91	12	149.21 ± 19.73	12	179.24 ± 16.54	8	3.54 [-0.18, 7.28]	-0.01 [-3.67, 3.77]	-0.25 [-5.96, 5.51]						
L 56	L 493	L Nucleus accumbens	21.39 ± 3.87	13	17.66 ± 2.92	13	147.04 ± 18.04	12	159.72 ± 16.88	8	5.16 [1.49, 8.74]*	0.04 [-3.55, 3.64]	-0.78 [-6.38, 4.78]	0.05 [-3.3, 3.7]	-6.03 [-9.54, -2.46]*	-6.57 [-10.53, -2.46]*	6.03 [2.46, 9.46]*	-6.58 [-10.63, -2.67]*	-0.55 [-4.5, 3.57]
R 754	R 493	R Olfactory tubercle	72.10 ± 19.61	13	61.79 ± 10.43	11	82.80 ± 11.83	9	91.05 ± 14.31	9	-0.67 [-4.55, 3.22]	-0.06 [-3.74, 3.62]	-0.71 [-6.42, 5.06]						
L 754	L 493	L Olfactory tubercle	73.85 ± 23.46	13	68.94 ± 12.81	12	87.05 ± 10.73	12	78.94 ± 6.82	8									
R 275	R 477	R Lateral septal complex	13.31 ± 2.76	14	27.97 ± 8.03	12	217.10 ± 33.91	11	242.29 ± 31.38	10	14.58 [10.88, 18.19]*	1.35 [-2.21, 5.02]	-0.6 [-6.07, 4.94]	-1.28 [-4.9, 2.17]	-15.42 [-18.94, -11.82]*	-17.45 [-21.22, -13.82]*	14.15 [10.41, 17.89]*	-16.16 [-20.06, -12.43]*	-2.04 [-5.96, 1.91]
L 275	L 477	L Lateral septal complex	6.44 ± 1.83	14	11.89 ± 2.78	12	124.10 ± 14.85	11	171.78 ± 17.69	10	17.5 [13.72, 21.14]*	1.08 [-2.49, 4.75]	5.16 [-0.29, 10.61]	-1 [-4.47, 2.55]	-18.38 [-21.78, -14.55]*	-25.89 [-29.58, -22.19]*	17.33 [13.45, 20.96]*	-24.88 [-28.71, -21.18]*	-7.56 [-11.36, -3.54]*
R 242	R 275	R Lateral septal nucleus	18.94 ± 3.90	14	44.23 ± 14.98	12	302.46 ± 44.31	11	358.21 ± 51.01	10	14.22 [10.58, 17.88]*	1.58 [-2.03, 5.1]	0.2 [-5.2, 5.65]	-1.51 [-4.98, 1.99]	-15.04 [-18.68, -11.56]*	-18.12 [-21.78, -14.42]*	13.55 [9.84, 17.3]*	-16.65 [-20.27, -12.71]*	-3.08 [-7, 0.8]
L 242	L 275	L Lateral septal nucleus	7.92 ± 2.28	14	14.16 ± 3.65	12	177.79 ± 19.45	11	259.15 ± 28.15	10	20.69 [16.97, 24.39]*	1.04 [-2.57, 4.72]	8.12 [2.56, 13.63]*	-0.97 [-4.42, 2.61]	-21.53 [-25.1, -17.86]*	-31.97 [-35.62, -28.2]*	20.56 [16.93, 24.26]*	-31.05 [-34.73, -27.15]*	-10.44 [-14.21, -6.5]*
R 258	R 242	R Lateral septal nucleus, rostral (rostroventral) part	39.71 ± 7.94	14	78.51 ± 33.06	11	682.80 ± 93.04	11	785.07 ± 123.41	10	15.42 [11.7, 19.05]*	1.3 [-2.37, 4.98]	0.15 [-5.38, 5.63]	-1.2 [-4.9, 2.23]	-16.26 [-19.74, -12.56]*	-19 [-22.69, -15.35]*	15.05 [11.2, 18.96]*	-17.77 [-21.52, -13.84]*	-2.74 [-6.58, 1.18]
L 258	L 242	L Lateral septal nucleus, rostral (rostroventral) part	16.28 ± 4.63	14	31.78 ± 7.94	11	388.50 ± 46.88	11	540.04 ± 55.75	10	22.14 [18.49, 25.82]*	1.27 [-2.37, 4.93]	6.89 [1.5, 12.33]*	-1.19 [-4.67, 2.39]	-22.96 [-26.51, -19.44]*	-32.44 [-36.14, -28.68]*	21.77 [18.02, 25.51]*	-31.23 [-34.98, -27.22]*	-9.47 [-13.39, -5.72]*
R 266	R 242	R Lateral septal nucleus, ventral part	25.33 ± 12.20	11	86.68 ± 44.01	6	331.69 ± 77.68	10	492.62 ± 98.12	8	11.24 [7.18, 15.34]*	2.63 [-1.94, 7.26]	2.67 [-3.82, 9.14]	-2.54 [-7.08, 1.95]	-12.08 [-16.22, -8.18]*	-18.68 [-22.89, -14.61]*	9.51 [4.88, 14.08]*	-16.11 [-20.72, -11.09]*	-6.62 [-10.78, -2.3]*
L 266	L 242	L Lateral septal nucleus, ventral part	7.76 ± 6.51	10	12.13 ± 5.67	8	285.96 ± 35.68	11	438.18 ± 100.78	8	35.16 [31.15, 39.14]*	0.87 [-3.44, 5.12]	17.31 [11.06, 23.33]*	-0.78 [-5.11, 3.27]	-36 [-39.81, -32.09]*	-55.44 [-59.63, -51.16]*	35.23 [31.03, 39.36]*	-54.7 [-59.03, -50.13]*	-19.46 [-23.76, -15.5]*
R 333	R 275	R Septohippocampal nucleus	52.76 ± 15.81	14	149.02 ± 46.55	7	477.08 ± 131.21	9	374.90 ± 137.25	6	7.2 [3.33, 11.11]*	2.19 [-1.99, 6.37]	-5.05 [-11.44, 1.35]	-2.1 [-6.14, 2.04]	-8.03 [-11.83, -4.19]*	-6.49 [-10.64, -1.89]*	5.92 [1.32, 10.2]*	-4.34 [-9.24, 0.61]	1.57 [-3.23, 6.14]
L 333	L 275	L Septohippocampal nucleus	22.66 ± 11.46	13	132.15 ± 49.38	7	339.67 ± 116.13	9	357.09 ± 141.35	6	13.18 [9.3, 17.12]*	5.22 [0.97, 9.52]*	-5.42 [-11.84, 0.98]	-5.16 [-9.19, -0.83]*	-14 [-17.78, -10.16]*	-15.12 [-19.41, -10.65]*	8.87 [4.44, 13.26]*	-9.94 [-14.87, -5.19]*	-1.08 [-5.79, 3.53]
R 278	R 477	R Striatum-like amygdalar nuclei	19.13 ± 8.08	15	15.29 ± 5.72	14	27.18 ± 5.21	12	32.90 ± 6.42	10	-0.36 [-3.94, 3.24]	-0.12 [-3.55, 3.35]	-0.73 [-6.07, 4.6]						
L 278	L 477	L Striatum-like amygdalar nuclei	21.37 ± 12.64	15	8.97 ± 2.41	14	16.01 ± 3.23	12	29.88 ± 7.32	10	-1.01 [-4.64, 2.59]	-0.49 [-3.98, 2.98]	-0.02 [-5.4, 5.22]						
R 23	R 278	R Anterior amygdalar area	45.65 ± 30.06	13	10.06 ± 4.43	9	50.15 ± 21.99	10	80.85 ± 28.71	6	-0.27 [-4.13, 3.52]	-0.39 [-4.39, 3.52]	-0.74 [-6.81, 5.5]						
R 536	R 278	R Central amygdalar nucleus	13.55 ± 4.78	15	8.79 ± 3.61	14	28.15 ± 8.17	11	48.53 ± 11.11	10	0.12 [-3.61, 3.75]	-0.27 [-3.74, 3.21]	0.8 [-4.48, 6.12]						
L 536	L 278	L Central amygdalar nucleus	25.99 ± 17.15	14	8.86 ± 3.22	13	23.94 ± 6.53	12	39.82 ± 10.38	10	-0.85 [-4.44, 2.76]	-0.57 [-4.1, 2.89]	0.01 [-5.3, 5.38]						
R 544	R 536	R Central amygdalar nucleus, capsular part	20.64 ± 4.01	14	19.51 ± 7.53	14	87.67 ± 27.05	10	179.49 ± 43.15	10	2.24 [-1.51, 6.05]	0.08 [-3.38, 3.54]	3.48 [-2.04, 8.91]						
L 544	L 536	L Central amygdalar nucleus, capsular part	29.55 ± 9.83	13	26.91 ± 8.25	13	78.56 ± 9.38	12	121.58 ± 29.46	10	0.94 [-2.79, 4.67]	0.09 [-3.56, 3.74]	0.2 [-5.25, 5.53]						
R 551	R 536	R Central amygdalar nucleus, lateral part	34.65 ± 9.90	14	17.59 ± 7.38	14	57.95 ± 25.49	10	82.58 ± 29.32	10	-0.35 [-4.12, 3.36]	-0.34 [-3.81, 3.17]	0.21 [-5.29, 5.71]						
L 551	L 536	L Central amygdalar nucleus, lateral part	49.60 ± 29.61	13	15.11 ± 7.72	13	37.64 ± 12.09	12	56.78 ± 22.88	10	-0.95 [-4.63, 2.71]	-0.51 [-4.14, 3.16]	-0.28 [-5.85, 5.27]						
R 559	R 536	R Central amygdalar nucleus, medial part	28.99 ± 13.37	15	17.74 ± 7.59	13	45.41 ± 11.56	11	67.77 ± 16.11	10	-0.4 [-4.08, 3.25]	-0.27 [-3.8, 3.25]	0.09 [-5.32, 5.67]						
L 559	L 536	L Central amygdalar nucleus, medial part	61.36 ± 45.46	14	14.59 ± 6.72	13	38.71 ± 18.03	12	74.22 ± 21.46	10	-1.15 [-4.69, 2.53]	-0.65 [-4.15, 2.91]	0.06 [-5.38, 5.37]						
R 1105	R 278	R Intercalated amygdalar nucleus	24.56 ± 11.42	15	27.85 ± 15.48	13	77.50 ± 24.56	12	118.48 ± 58.08	10	1.38 [-2.21, 5]	0.25 [-3.28, 3.79]	0.26 [-5.25, 5.72]						
L 1105	L 278	L Intercalated amygdalar nucleus	9.47 ± 4.97	15	15.42 ± 7.54	13	48.35 ± 18.71	12	145.47 ± 51.42	10	3.35 [-0.2, 6.86]	0.75 [-2.74, 4.2]	8.35 [2.98, 13.71]*	-0.67 [-4.11, 2.62]	-4.17 [-7.6, -0.73]*	-14.59 [-18.14, -10.92]*	3.51 [-0.21, 6.91]	-13.93 [-17.51, -10.04]*	-10.4 [-14.07, -6.41]*
R 403	R 278	R Medial amygdalar nucleus	61.02 ± 30.40	15	42.56 ± 15.57	14	70.43 ± 12.83	12	62.93 ± 13.26	10	-0.62 [-4.21, 3.05]	-0.22 [-3.63, 3.2]	-1.06 [-6.36, 4.26]						
L 403	L 278	L Medial amygdalar nucleus	54.00 ± 35.75	15	23.55 ± 6.66	14	26.18 ± 4.56	12	45.30 ± 8.65	10	-1.28 [-4.83, 2.28]	-0.47 [-3.88, 2.87]	-0.34 [-5.78, 4.95]						
R 803	R 623	R Pallidum	10.95 ± 3.33	15	13.69 ± 3.79	14	24.96 ± 8.77	11	31.76 ± 6.76	10	1.37 [-0.02, 2.77]	0.02 [-1.3, 1.33]	-0.23 [-2.28, 1.78]						
L 803	L 623	L Pallidum	7.46 ± 2.51	15	12.27 ± 4.28	14	16.37 ± 4.73	12	25.01 ± 4.72	10	1.4 [0, 2.78]	0.44 [-0.89, 1.77]	-0.21 [-2.27, 1.87]	-0.55 [-1.88, 0.83]	-1.71 [-3.14, -0.33]*	-2.83 [-4.41, -1.19]*	1.15 [-0.35, 2.63]	-2.27 [-3.91, -0.69]*	-1.12 [-2.89, 0.43]
R 818	R 803	R Pallidum, dorsal region	8.80 ± 4.46	15	10.34 ± 6.62	13	3.51 ± 0.84	12	11.72 ± 2.12	10									
L 818	L 803	L Pallidum, dorsal region	5.72 ± 3.18	15	8.29 ± 6.27	13	3.81 ± 0.92	12	9.21 ± 1.79	10	-0.14 [-1.52, 1.24]	0.37 [-0.99, 1.71]	-0.34 [-2.4, 1.67]						
R 1022	R 818	R Globus pallidus, external segment	21.06 ± 11.51	15	25.02 ± 17.10	13	7.78 ± 1.60	11	24.15 ± 5.38	10	-0.53 [-1.96, 0.86]	0.1 [-1.25, 1.44]	-0.15 [-2.21, 1.89]						
L 1022	L 818	L Globus pallidus, external segment	16.80 ± 11.74	14	19.60 ± 16.92	13	6.70 ± 1.68	12	18.06 ± 4.68	10	-0.39 [-1.8, 1.01]	0.09 [-1.27, 1.45]	-0.35 [-2.38, 1.73]						
R 1031	R 818	R Globus pallidus, internal segment	16.00 ± 5.51	14	19.15 ± 11.89	12	7.70 ± 2.40	10	21.75 ± 4.34	10	-0.37 [-1.76, 1.06]	0.12 [-1.27, 1.49]	-0.06 [-2.15, 2.04]						
L 1031	L 818	L Globus pallidus, internal segment	10.01 ± 2.81	13	11.39 ± 5.68	13	7.65 ± 2.56	12	16.85 ± 6.38	9	0.05 [-1.37, 1.45]	0.14 [-1.22, 1.5]	0.02 [-2.04, 2.1]						
R 835	R 803	R Pallidum, ventral region	14.28 ± 4.00	15	8.45 ± 2.10	14	21.11 ± 5.53	12	27.03 ± 5.61	10	0.67 [-0.73, 2.06]	-0.63 [-2, 0.69]	0.13 [-1.91, 2.19]						
L 835	L 803	L Pallidum, ventral region	11.95 ± 3.31	15	8.17 ± 1.88	14	18.32 ± 4.56	12	29.25 ± 5.98	10	0.73 [-0.65, 2.1]	-0.53 [-1.86, 0.81]	0.53 [-1.5, 2.54]						
R 342	R 835	R Substantia innominata	29.16 ± 8.25	15	16.54 ± 4.23	14	40.64 ± 11.01	12	58.74 ± 9.68	9	0.58 [-0.76, 1.98]	-0.65 [-2, 0.66]	0.43 [-1.61, 2.44]						
L 342	L 835	L Substantia innominata	24.62 ± 6.84	15	16.99 ± 3.89	13	36.13 ± 9.26	12	60.74 ± 8.49	9	0.66 [-0.73, 2.06]	-0.57 [-1.93, 0.74]	0.73 [-1.33, 2.85]						
R 826	R 803	R Pallidum, medial region	32.88 ± 8.04	14	54.80 ± 10.86	11	129.94 ± 45.83	12	140.21 ± 38.18	9	3.14 [1.76, 4.55]*	0.82 [-0.58, 2.2]	-1.49 [-3.6, 0.58]	-0.93 [-2.29, 0.51]	-3.44 [-4.82, -2.02]*	-3.67 [-5.35, -2.08]*	2.52 [0.96, 4.01]*	-2.74 [-4.38, -1.06]*	-0.22 [-1.87, 1.42]
L 826	L 803	L Pallidum, medial region	23.24 ± 7.98	14	50.37 ± 15.84	10	75.50 ± 24.77	12	97.42 ± 24.45	9	2.45 [1.07, 3.86]*	1.31 [-0.13, 2.74]	-1.34 [-3.46, 0.77]	-1.42 [-2.84, 0.02]	-2.75 [-4.27, -1.42]*	-3.6 [-5.29, -1.95]*	1.35 [-0.27, 2.88]	-2.19 [-3.89, -0.45]*	-0.85 [-2.53, 0.81]
R 904	R 826	R Medial septal complex	49.32 ± 12.06	14	82.91 ± 16.02	11	175.64 ± 58.33	12	188.96 ± 52.37	9	2.77 [1.36, 4.18]*	0.85 [-0.55, 2.27]	-1.56 [-3.65, 0.59]	-0.96 [-2.41, 0.46]	-3.08 [-4.47, -1.62]*	-3.25 [-4.85, -1.58]*	2.11 [0.54, 3.64]*	-2.29 [-3.92, -0.54]*	-0.18 [-1.85, 1.46]
L 904	L 826	L Medial septal complex	34.86 ± 11.97	14	75.92 ± 23.68	10	107.54 ± 35.01	12	142.33 ± 35.54	9	2.28 [0.9, 3.69]*	1.32 [-0.08, 2.72]	-1.3 [-3.45, 0.76]	-1.43 [-2.8, 0.03]	-2.59 [-4.01, -1.19]*	-3.5 [-5.1, -1.83]*	1.16 [-0.48, 2.66]	-2.06 [-3.73, -0.39]*	-0.91 [-2.53, 0.81]
R 596	R 904	R Diagonal band nucleus	100.10 ± 24.59	14	165.86 ± 34.16	11	314.18 ± 108.74	12	306.71 ± 102.76	9	2.34 [0.95, 3.77]*	0.82 [-0.59, 2.2]	-1.87 [-3.99, 0.27]	-0.93 [-2.33, 0.5]	-2.64 [-4.1, -1.25]*	-2.48 [-4.17, -0.89]*	1.71 [0.19, 3.29]*	-1.54 [-3.26, 0.09]	0.16 [-1.55, 1.81]
L 596	L 904	L Diagonal band nucleus	69.79 ± 24.71	14	150.60 ± 47.34	10	208.20 ± 67.34	12	250.01 ± 65.77	9	2.18 [0.8, 3.57]*	1.3 [-0.08, 2.71]	-1.68 [-3.79, 0.41]	-1.41 [-2.8, 0.05]	-2.49 [-3.88, -1.09]*	-3 [-4.59, -1.32]*	1.08 [-0.47, 2.59]	-1.58 [-3.27, 0.12]	-0.51 [-2.24, 1.1]
R 549	R 1129	R Thalamus	20.10 ± 11.44	15	18.26 ± 6.07	15	25.35 ± 3.49	12	43.46 ± 10.29	10	-0.33 [-4.08, 3.46]	-0.03 [-3.52, 3.58]	-0.23 [-5.81, 5.33]						
L 549	L 1129	L Thalamus	5.43 ± 1.80	14	6.69 ± 2.00	15	14.90 ± 2.66	12	25.71 ± 5.24	10	1.19 [-2.68, 5.01]	0.31 [-3.25, 3.84]	0.53 [-4.98, 6.21]						
R 864	R 549	R Thalamus, sensory-motor cortex related	17.48 ± 11.66	15	14.61 ± 4.45	15	25.15 ± 4.45	12	42.37 ± 10.76	10	-0.12 [-3.92, 3.73]	-0.09 [-3.63, 3.44]	-0.07 [-5.73, 5.61]						
L 864	L 549	L Thalamus, sensory-motor cortex related	2.79 ± 0.92	14	4.57 ± 1.40	15	15.30 ± 3.16	12	26.39 ± 5.49	10	3.9 [0.09, 7.69]*	0.72 [-2.84, 4.34]	2.14 [-3.34, 7.68]	-0.67 [-4.18, 2.84]	-4.73 [-8.47, -0.94]*	-9 [-13.07, -5.13]*	4.05 [0.34, 7.79]*	-8.32 [-12.39, -4.58]*	-4.27 [-8.42, -0.33]*
R 637	R 864	R Ventral group of the dorsal thalamus	15.51 ± 10.86	15	11.38 ± 6.16	15	23.34 ± 3.89	12	34.53 ± 6.14	10	-0.1 [-3.87, 3.67]	-0.23 [-3.77, 3.26]	-0.2 [-5.71, 5.44]						
L 637	L 864	L Ventral group of the dorsal thalamus	3.22 ± 1.12	14	4.05 ± 1.49	15	18.55 ± 3.68	12	31.14 ± 6.94	10	4.21 [0.42, 8.07]*	0.34 [-3.13, 4.02]	2.44 [-3.13, 8.01]	-0.29 [-3.9, 3.14]	-5.04 [-9.05, -1.55]*	-9.25 [-13.39, -5.25]*	4.75 [1.23, 8.55]*	-8.96 [-12.94, -4.91]*	-4.19 [-8.41, -0.06]*
R 629	R 637	R Ventral anterior-lateral complex of the thalamus	9.12 ± 3.13	15	17.55 ± 12.40	13	65.36 ± 18.51	12	69.37 ± 14.29	10	5.59 [1.76, 9.31]*	0.93 [-2.69, 4.61]	-1.65 [-7.25, 3.95]	-0.86 [-4.6, 2.51]	-6.4 [-10.03, -2.57]*	-7.11 [-11.25, -3.32]*	5.53 [1.58, 9.16]*	-6.2 [-10.2, -2.19]*	-0.71 [-4.72, 3.49]
L 629	L 637	L Ventral anterior-lateral complex of the thalamus	5.87 ± 2.40	14	13.22 ± 6.49	13	39.66 ± 9.69	12	78.12 ± 25.98	10	5.19 [1.27, 9.01]*	1.29 [-2.5, 5.04]	4.1 [-1.59, 9.9]	-1.24 [-4.99, 2.38]	-6.02 [-9.79, -2.18]*	-12.8 [-16.85, -8.86]*	4.78 [1.03, 8.59]*	-11.56 [-15.72, -7.64]*	-6.82 [-10.9, -2.57]*
R 685	R 637	R Ventral medial nucleus of the thalamus	9.14 ± 3.17	15	37.64 ± 21.93	14	53.02 ± 8.69	12	77.09 ± 12.81	10	4.22 [0.38, 7.95]*	3.12 [-0.41, 6.59]	-1.66 [-7.13, 3.98]	-3.06 [-6.54, 0.39]	-5.07 [-8.78, -1.34]*	-7.9 [-11.96, -4.08]*	1.95 [-1.82, 5.88]	-4.85 [-8.77, -0.85]*	-2.88 [-7.22, 1.17]
L 685	L 637	L Ventral medial nucleus of the thalamus	9.89 ± 5.38	14	12.47 ± 5.40	14	41.80 ± 8.97	12	69.33 ± 20.04	10	2.68 [-1.14, 6.49]	0.29 [-3.38, 3.93]	1.34 [-4.23, 6.96]						
R 709	R 637	R Ventral posterior complex of the thalamus	31.99 ± 24.77	15	16.48 ± 8.81	15	31.36 ± 5.32	12	47.66 ± 10.18	10	-0.63 [-4.33, 3.07]	-0.46 [-3.97, 3.07]	-0.2 [-5.74, 5.34]						
L 709	L 637	L Ventral posterior complex of the thalamus	4.75 ± 1.64	13	4.26 ± 2.01	15	25.97 ± 5.19	12	43.42 ± 9.24	10	3.99 [0.19, 7.88]*	0.05 [-3.56, 3.68]	2.45 [-3.13, 8.09]	-0.01 [-3.56, 3.5]	-4.83 [-8.68, -1.14]*	-8.72 [-12.77, -4.66]*	4.82 [1.03, 8.36]*	-8.74 [-12.71, -4.88]*	-3.91 [-7.93, 0.29]
R 718	R 709	R Ventral posterolateral nucleus of the thalamus	128.35 ± 93.72	15	48.32 ± 25.65	15	56.55 ± 8.03	12	85.67 ± 13.99	10	-1.13 [-4.89, 2.62]	-0.56 [-4.1, 2.98]	-0.39 [-5.89, 5.18]						
L 718	L 709	L Ventral posterolateral nucleus of the thalamus	16.85 ± 7.04	13	10.88 ± 4.50	15	49.65 ± 10.89	12	98.11 ± 28.94	10	1.45 [-2.43, 5.25]	-0.19 [-3.76, 3.47]	1.92 [-3.7, 7.58]						
R 725	R 709	R Ventral posterolateral nucleus of the thalamus, parvicellular part	38.35 ± 24.97	11	6.53 ± 2.14	12	135.09 ± 57.56	9	88.12 ± 19.81	7	2.59 [-1.79, 6.88]	-0.22 [-4.29, 3.79]	-1.79 [-8.07, 4.47]						
L 725	L 709	L Ventral posterolateral nucleus of the thalamus, parvicellular part	4.87 ± 3.79	11	3.51 ± 1.92	11	57.63 ± 16.45	8	63.30 ± 21.19	7	10.25 [5.9, 14.58]*	-0.08 [-4.13, 3.86]	0.93 [-5.44, 7.41]	0.13 [-3.75, 4.13]	-11.06 [-15.38, -6.84]*	-13.34 [-17.93, -8.76]*	11.19 [6.82, 15.43]*	-13.46 [-18.28, -8.92]*	-2.26 [-6.98, 2.67]
R 733	R 709	R Ventral posteromedial nucleus of the thalamus	25.91 ± 20.76	14	26.36 ± 12.92	15	64.74 ± 13.91	12	107.63 ± 28.63	10	0.96 [-2.88, 4.87]	0.14 [-3.47, 3.8]	0.38 [-5.25, 6]						
L 733	L 709	L Ventral posteromedial nucleus of the thalamus	5.90 ± 2.79	13	7.65 ± 4.50	15	52.26 ± 9.56	12	86.59 ± 19.14	10	7.34 [3.48, 11.19]*	0.44 [-3.12, 4.1]	4.25 [-1.32, 9.76]	-0.39 [-3.94, 3.22]	-8.16 [-12.19, -4.57]*	-14.24 [-18.37, -10.31]*	7.78 [4.12, 11.5]*	-13.86 [-17.86, -10.03]*	-6.13 [-10.39, -2.25]*
R 741	R 709	R Ventral posteromedial nucleus of the thalamus, parvicellular part	6.20 ± 4.23	9	4.38 ± 1.70	11	62.10 ± 16.54	12	52.47 ± 14.93	7	8.06 [3.85, 12.26]*	-0.27 [-4.43, 3.98]	-1.84 [-8.13, 4.38]	0.31 [-3.83, 4.51]	-8.89 [-13.01, -4.79]*	-8.23 [-12.7, -3.25]*	9.19 [5.22, 13.17]*	-8.53 [-12.96, -4.03]*	0.69 [-3.81, 5.25]
L 741	L 709	L Ventral posteromedial nucleus of the thalamus, parvicellular part	2.07 ± 1.07	8	3.56 ± 2.66	13	58.91 ± 23.08	10	54.65 ± 17.29	7	26.43 [21.93, 30.86]*	0.18 [-4.16, 4.45]	-2.75 [-9.24, 3.64]	-0.13 [-4.24, 4.22]	-27.26 [-31.61, -22.77]*	-26.08 [-31.23, -21.59]*	27.13 [22.99, 30.99]*	-25.99 [-30.36, -21.57]*	1.16 [-3.36, 5.92]
R 563807435	R 637	R Posterior triangular thalamic nucleus	69.49 ± 46.29	9	11.19 ± 7.21	9	32.61 ± 13.28	9	96.76 ± 21.06	7	-0.97 [-5.5, 3.5]	-0.73 [-5.16, 3.7]	2 [-4.67, 8.62]						
L 563807435	L 637	L Posterior triangular thalamic nucleus	4.36 ± 2.35	9	6.77 ± 2.87	7	47.28 ± 16.60	9	58.52 ± 17.80	7	9.43 [5.05, 13.94]*	1.5 [-3.12, 6.22]	1.55 [-5.17, 8.24]	-1.45 [-6, 3.23]	-10.24 [-14.55, -5.87]*	-14.7 [-19.48, -10.07]*	8.81 [4.12, 13.55]*	-13.25 [-18.28, -8.26]*	-4.44 [-9.3, -0.02]*
R 406	R 864	R Subparafascicular nucleus	44.16 ± 28.24	11	6.49 ± 5.38	13	29.21 ± 9.23	10	52.60 ± 8.26	8	-0.85 [-5.01, 3.4]	-0.84 [-4.78, 3.11]	1.32 [-4.7, 7.39]						
L 406	L 864	L Subparafascicular nucleus	8.44 ± 5.00	11	5.69 ± 2.85	12	24.15 ± 9.92	10	32.98 ± 7.14	8	1.38 [-2.85, 5.54]	-0.08 [-4.04, 3.83]	1.07 [-5.16, 7.14]						
R 414	R 406	R Subparafascicular nucleus, magnocellular part	25.76 ± 18.91	6	1.32 ± 1.32	8	56.26 ± 19.56	7	58.22 ± 11.25	6	0.86 [-4.22, 5.91]	-0.8 [-5.77, 4.13]	-0.2 [-7.33, 6.97]						
R 422	R 406	R Subparafascicular nucleus, parvicellular part	107.27 ± 68.83	9	19.60 ± 15.39	9	52.50 ± 25.20	7	118.87 ± 29.15	7	-1.02 [-5.73, 3.62]	-0.72 [-5.12, 3.68]	1.76 [-4.99, 8.49]						
L 422	L 406	L Subparafascicular nucleus, parvicellular part	19.72 ± 13.16	9	4.39 ± 2.28	8	65.84 ± 29.07	8	94.85 ± 29.39	7	1.82 [-2.68, 6.2]	-0.76 [-5.2, 3.72]	2.79 [-3.75, 9.42]						
R 1008	R 864	R Geniculate group, dorsal thalamus	97.46 ± 64.51	14	65.02 ± 23.10	15	94.50 ± 24.48	12	169.97 ± 83.25	9	-0.55 [-4.44, 3.28]	-0.21 [-3.86, 3.37]	0.85 [-4.79, 6.56]						
L 1008	L 864	L Geniculate group, dorsal thalamus	7.04 ± 2.94	14	19.72 ± 11.41	15	33.54 ± 7.36	12	65.35 ± 17.46	10	3.21 [-0.6, 7.12]	1.91 [-1.68, 5.46]	1.47 [-4.02, 6.98]						
R 475	R 1008	R Medial geniculate complex	6.88 ± 4.39	8	19.54 ± 9.22	8	32.87 ± 15.76	8	74.48 ± 24.96	7	2.94 [-1.59, 7.55]	1.38 [-3.19, 5.9]	4.93 [-1.81, 11.66]						
L 475	L 1008	L Medial geniculate complex	7.45 ± 2.53	8	13.83 ± 7.30	8	29.40 ± 12.34	9	52.94 ± 13.33	7	2.08 [-2.45, 6.58]	1.37 [-3.18, 5.95]	2.25 [-4.41, 8.87]						
L 1072	L 475	L Medial geniculate complex, dorsal part	12.41 ± 5.03	7	91.06 ± 54.05	6	40.99 ± 20.17	8	95.63 ± 17.73	7	2.12 [-2.74, 6.85]	6.76 [1.7, 11.84]*	-2.57 [-9.54, 4.6]	-6.7 [-11.83, -1.83]*	-2.96 [-7.65, 1.85]	-8.56 [-13.31, -3.49]*	-3.77 [-8.86, 1.14]	-1.8 [-7.16, 3.25]	-5.59 [-10.43, -0.71]*
L 1079	L 475	L Medial geniculate complex, ventral part	30.94 ± 12.78	6	31.94 ± 19.23	6	29.31 ± 18.12	8	90.43 ± 36.02	6	-0.13 [-5.03, 4.88]	0.52 [-4.73, 5.85]	0.92 [-6.32, 8.27]						
R 1088	R 475	R Medial geniculate complex, medial part	15.67 ± 15.67	8	12.74 ± 5.66	8	69.02 ± 34.05	8	91.69 ± 37.99	7	2.59 [-2.04, 7.2]	-0.65 [-5.26, 3.89]	2.34 [-4.23, 9.04]						
L 1088	L 475	L Medial geniculate complex, medial part	6.25 ± 2.52	8	10.63 ± 5.64	8	68.13 ± 26.35	9	120.21 ± 33.45	7	9.02 [4.48, 13.61]*	1.2 [-3.35, 5.79]	7.59 [0.9, 14.35]*	-1.13 [-5.63, 3.45]	-9.83 [-14.43, -5.4]*	-20.04 [-24.76, -15.05]*	8.73 [4.14, 13.14]*	-18.89 [-23.58, -13.93]*	-10.18 [-14.95, -5.43]*
R 170	R 1008	R Dorsal part of the lateral geniculate complex	150.07 ± 96.41	14	113.69 ± 38.51	15	169.40 ± 39.77	12	278.64 ± 123.91	9	-0.36 [-4.17, 3.4]	-0.1 [-3.68, 3.46]	0.69 [-4.92, 6.36]						
L 170	L 1008	L Dorsal part of the lateral geniculate complex	9.60 ± 4.67	14	31.23 ± 17.32	15	58.96 ± 10.55	12	104.54 ± 26.58	10	4.59 [0.82, 8.39]*	2.36 [-1.24, 5.94]	1.23 [-4.38, 6.81]	-2.31 [-5.86, 1.23]	-5.41 [-9.07, -1.6]*	-10.42 [-14.53, -6.6]*	3.11 [-0.73, 6.61]	-8.14 [-12, -4.14]*	-5.02 [-9.13, -0.92]*
R 496345664	R 170	R Dorsal part of the lateral geniculate complex, shell	388.57 ± 233.96	13	405.49 ± 168.04	14	432.40 ± 119.55	12	523.24 ± 263.57	9	-0.38 [-4.27, 3.53]	0.12 [-3.6, 3.84]	-0.04 [-5.91, 5.58]						
L 496345664	L 170	L Dorsal part of the lateral geniculate complex, shell	42.96 ± 22.57	14	139.70 ± 109.01	14	113.37 ± 33.45	11	171.97 ± 43.09	9	1.04 [-2.82, 4.96]	2.86 [-0.79, 6.53]	-1.57 [-7.36, 4.14]						
R 496345668	R 170	R Dorsal part of the lateral geniculate complex, core	374.58 ± 226.87	14	184.68 ± 62.13	15	373.06 ± 113.51	12	513.57 ± 212.84	9	-0.51 [-4.35, 3.22]	-0.4 [-4.04, 3.2]	0.61 [-5.09, 6.3]						
L 496345668	L 170	L Dorsal part of the lateral geniculate complex, core	12.38 ± 5.65	14	62.29 ± 31.82	15	116.80 ± 21.13	12	208.32 ± 49.98	10	7.91 [4.11, 11.78]*	4.15 [0.62, 7.69]*	2.06 [-3.46, 7.68]	-4.11 [-7.74, -0.76]*	-8.7 [-12.54, -5.02]*	-16.37 [-20.27, -12.33]*	4.62 [0.96, 8.43]*	-12.24 [-16.17, -8.29]*	-7.61 [-11.73, -3.52]*
R 496345672	R 170	R Dorsal part of the lateral geniculate complex, ipsilateral zone	99.05 ± 65.13	12	255.88 ± 138.88	14	200.82 ± 74.45	12	805.96 ± 391.03	9	0.58 [-3.38, 4.58]	1.67 [-2.13, 5.4]	4.3 [-1.4, 10.21]						
L 496345672	L 170	L Dorsal part of the lateral geniculate complex, ipsilateral zone	17.71 ± 10.20	12	13.18 ± 7.24	15	128.28 ± 46.07	10	176.01 ± 64.80	9	5.81 [1.54, 9.95]*	-0.04 [-3.76, 3.79]	2.61 [-3.3, 8.51]	0.09 [-3.67, 3.78]	-6.65 [-10.78, -2.55]*	-10.62 [-14.99, -6.51]*	6.7 [2.88, 10.73]*	-10.7 [-14.78, -6.53]*	-3.99 [-8.44, 0.36]
R 856	R 549	R Thalamus, polymodal association cortex related	30.76 ± 16.73	15	29.17 ± 10.87	15	37.05 ± 4.79	12	64.38 ± 15.46	10	-0.39 [-4.17, 3.32]	0 [-3.56, 3.58]	-0.27 [-5.84, 5.3]						
L 856	L 549	L Thalamus, polymodal association cortex related	9.44 ± 3.11	15	11.07 ± 3.28	15	21.20 ± 3.68	12	37.45 ± 8.22	10	0.64 [-3.08, 4.46]	0.23 [-3.34, 3.8]	0.36 [-5.27, 5.86]						
R 138	R 856	R Lateral group of the dorsal thalamus	31.62 ± 14.89	14	38.92 ± 13.83	15	59.08 ± 8.95	12	118.55 ± 34.82	10	0.36 [-3.52, 4.2]	0.35 [-3.31, 3.98]	0.36 [-5.13, 5.93]						
L 138	L 856	L Lateral group of the dorsal thalamus	11.30 ± 5.98	14	17.34 ± 7.24	15	31.05 ± 4.71	12	64.58 ± 12.36	10	1.23 [-2.61, 5.03]	0.67 [-2.95, 4.26]	1.12 [-4.47, 6.84]						
R 218	R 138	R Lateral posterior nucleus of the thalamus	143.56 ± 81.82	14	166.37 ± 63.85	15	185.02 ± 37.15	12	441.34 ± 163.92	10	-0.23 [-3.96, 3.48]	0.28 [-3.25, 3.81]	0.34 [-5.18, 5.87]						
L 218	L 138	L Lateral posterior nucleus of the thalamus	51.91 ± 33.73	14	67.20 ± 29.40	15	61.87 ± 13.06	12	147.33 ± 29.46	10	-0.35 [-4.18, 3.41]	0.4 [-3.15, 3.99]	0.08 [-5.54, 5.64]						
R 1020	R 138	R Posterior complex of the thalamus	16.51 ± 6.34	14	8.77 ± 4.29	15	69.99 ± 12.18	12	102.37 ± 20.01	10	2.71 [-1.15, 6.52]	-0.36 [-3.96, 3.22]	1.15 [-4.45, 6.71]						
L 1020	L 138	L Posterior complex of the thalamus	7.07 ± 2.46	14	8.70 ± 3.78	15	50.76 ± 7.97	12	106.25 ± 25.05	10	5.66 [1.82, 9.46]*	0.35 [-3.17, 3.9]	6.35 [0.79, 11.92]*	-0.31 [-3.89, 3.1]	-6.47 [-10.21, -2.68]*	-14.61 [-18.48, -10.53]*	6.2 [2.3, 9.66]*	-14.31 [-18.33, -10.49]*	-8.12 [-12.31, -4.02]*
R 1029	R 138	R Posterior limiting nucleus of the thalamus	96.89 ± 78.90	10	77.22 ± 47.96	9	87.13 ± 42.16	10	224.90 ± 61.40	8	-0.53 [-4.78, 3.63]	-0.1 [-4.29, 4.2]	1.44 [-4.86, 7.85]						
L 1029	L 138	L Posterior limiting nucleus of the thalamus	23.63 ± 12.52	11	13.06 ± 7.59	7	86.03 ± 20.70	10	111.88 ± 30.34	7	2.21 [-2.04, 6.39]	0.43 [-4, 4.81]	1.11 [-5.41, 7.77]						
R 560581551	R 138	R Ethmoid nucleus of the thalamus	9.78 ± 5.55	13	11.67 ± 4.13	15	97.74 ± 30.46	12	75.05 ± 18.10	10	8.5 [4.62, 12.35]*	0.35 [-3.25, 4.06]	-3.83 [-9.39, 1.77]	-0.29 [-3.96, 3.18]	-9.31 [-13.15, -5.58]*	-7.25 [-11.36, -3.28]*	9.03 [5.33, 12.69]*	-6.96 [-10.9, -2.96]*	2.09 [-2.03, 6.17]
L 560581551	L 138	L Ethmoid nucleus of the thalamus	4.91 ± 2.08	13	5.56 ± 3.49	15	87.72 ± 22.92	12	108.25 ± 24.31	10	16.36 [12.41, 20.28]*	0.28 [-3.36, 3.95]	2.75 [-2.86, 8.3]	-0.22 [-3.78, 3.4]	-17.17 [-21.01, -13.36]*	-21.61 [-25.83, -17.71]*	16.96 [13.24, 20.77]*	-21.38 [-25.39, -17.51]*	-4.42 [-8.55, -0.33]*
R 239	R 856	R Anterior group of the dorsal thalamus	70.40 ± 36.10	15	61.45 ± 23.79	13	77.17 ± 12.97	12	131.21 ± 37.69	10	-0.48 [-4.23, 3.26]	-0.13 [-3.78, 3.48]	-0.26 [-5.88, 5.37]						
L 239	L 856	L Anterior group of the dorsal thalamus	11.54 ± 3.57	15	31.14 ± 12.66	13	40.59 ± 8.80	12	54.07 ± 11.32	10	1.92 [-1.81, 5.64]	1.69 [-2, 5.31]	-1.68 [-7.27, 3.87]						
R 255	R 239	R Anteroventral nucleus of thalamus	30.60 ± 13.55	13	41.79 ± 26.63	9	121.32 ± 72.85	12	57.63 ± 20.20	8	2.42 [-1.51, 6.26]	0.27 [-3.75, 4.3]	-2.03 [-8.11, 4.02]						
L 255	L 239	L Anteroventral nucleus of thalamus	10.43 ± 3.49	12	52.75 ± 47.70	9	40.02 ± 14.51	12	57.69 ± 23.59	8	2.15 [-1.96, 6.18]	3.78 [-0.49, 7.95]	-1.9 [-8.21, 4.38]						
R 127	R 239	R Anteromedial nucleus	4.99 ± 1.51	13	16.19 ± 13.89	8	19.12 ± 7.66	12	18.18 ± 6.40	8	2.05 [-1.87, 5.94]	2.23 [-2.02, 6.41]	-2.08 [-8.14, 3.99]						
L 127	L 239	L Anteromedial nucleus	5.54 ± 2.21	13	26.15 ± 23.18	9	12.91 ± 4.37	12	11.98 ± 4.64	8	0.54 [-3.3, 4.41]	3.37 [-0.66, 7.4]	-3.23 [-9.19, 2.78]						
R 1096	R 127	R Anteromedial nucleus, dorsal part	9.49 ± 4.06	13	31.83 ± 26.10	8	34.95 ± 12.18	12	37.11 ± 15.84	8	1.93 [-1.97, 5.78]	2.36 [-1.85, 6.66]	-1.81 [-7.93, 4.47]						
L 1096	L 127	L Anteromedial nucleus, dorsal part	14.59 ± 6.50	13	52.74 ± 48.72	9	28.19 ± 11.40	12	28.46 ± 10.75	8	0.17 [-3.67, 4.02]	2.28 [-1.75, 6.33]	-1.95 [-8.08, 4.07]						
R 1104	R 127	R Anteromedial nucleus, ventral part	10.44 ± 3.94	13	34.16 ± 32.01	8	45.82 ± 23.27	12	37.44 ± 13.30	8	2.62 [-1.25, 6.44]	2.26 [-1.99, 6.43]	-2.72 [-8.81, 3.39]						
L 1104	L 127	L Anteromedial nucleus, ventral part	5.50 ± 2.81	13	51.87 ± 42.97	9	20.30 ± 10.20	12	21.32 ± 10.55	7	1.93 [-1.95, 5.78]	8.09 [4.05, 12.04]*	-7.1 [-13.17, -0.9]*	-8.02 [-11.86, -3.92]*	-2.77 [-6.46, 1.11]	-5.15 [-9.39, -0.63]*	-5.31 [-9.31, -0.96]*	2.93 [-1.72, 7.56]	-2.4 [-6.73, 2.16]
R 64	R 239	R Anterodorsal nucleus	119.85 ± 60.23	12	176.82 ± 109.31	9	202.86 ± 94.83	11	161.16 ± 49.59	8	-0.01 [-4.08, 4.02]	0.2 [-4.01, 4.36]	-0.23 [-6.48, 6.04]						
L 64	L 239	L Anterodorsal nucleus	81.92 ± 36.74	11	100.42 ± 77.65	9	127.10 ± 64.34	10	110.63 ± 66.99	7	-0.01 [-4.16, 4.2]	0 [-4.33, 4.21]	0.11 [-6.32, 6.44]						
R 1120	R 239	R Interanteromedial nucleus of the thalamus	3.17 ± 3.17	12	32.88 ± 27.73	8	8.08 ± 3.75	11	40.26 ± 21.36	7	0.63 [-3.35, 4.7]	8.89 [4.61, 13.14]*	1.79 [-4.54, 8.26]	-8.83 [-13.05, -4.65]*	-1.45 [-5.32, 2.65]	-13.53 [-18.04, -9.21]*	-7.4 [-11.48, -2.89]*	-4.7 [-9.34, 0.17]	-12.06 [-16.7, -7.59]*
L 1120	L 239	L Interanteromedial nucleus of the thalamus	6.10 ± 3.21	11	54.43 ± 51.37	8	12.43 ± 4.82	11	39.37 ± 15.72	7	0.18 [-3.83, 4.21]	7.49 [3.15, 11.83]*	-2.55 [-8.85, 3.8]	-7.44 [-11.57, -2.98]*	-1.01 [-4.86, 3.1]	-7.38 [-11.76, -2.75]*	-6.4 [-10.8, -2.25]*	0.08 [-4.8, 4.8]	-6.35 [-11, -1.91]*
R 1113	R 239	R Interanterodorsal nucleus of the thalamus	14.57 ± 7.21	12	45.64 ± 40.34	9	49.80 ± 15.76	12	71.45 ± 24.84	8	1.71 [-2.17, 5.66]	1.85 [-2.3, 5.97]	-0.04 [-6.13, 5.96]						
L 1113	L 239	L Interanterodorsal nucleus of the thalamus	5.32 ± 2.42	12	52.34 ± 49.21	9	28.93 ± 11.22	12	35.77 ± 14.05	7	3.73 [-0.22, 7.68]	8.57 [4.41, 12.69]*	-6.49 [-12.73, -0.2]*	-8.51 [-12.66, -4.53]*	-4.55 [-8.41, -0.57]*	-8.05 [-12.27, -3.44]*	-3.98 [-8.04, 0.19]	0.47 [-4.14, 5.04]	-3.48 [-7.84, 1.08]
R 155	R 239	R Lateral dorsal nucleus of thalamus	244.15 ± 128.66	15	148.97 ± 51.10	13	253.47 ± 43.50	12	424.32 ± 152.63	10	-0.57 [-4.28, 3.14]	-0.42 [-4.01, 3.21]	-0.01 [-5.57, 5.55]						
L 155	L 239	L Lateral dorsal nucleus of thalamus	24.13 ± 8.21	15	48.65 ± 18.96	13	130.27 ± 33.97	12	151.08 ± 32.49	10	3.78 [-0.02, 7.52]	1 [-2.62, 4.7]	-1.28 [-6.9, 4.46]						
R 444	R 856	R Medial group of the dorsal thalamus	11.02 ± 5.45	15	14.62 ± 7.00	14	32.40 ± 9.11	12	45.19 ± 13.31	10	1.37 [-2.37, 5.15]	0.33 [-3.19, 3.93]	-0.35 [-5.85, 5.27]						
L 444	L 856	L Medial group of the dorsal thalamus	11.57 ± 5.44	15	8.70 ± 4.65	14	26.24 ± 6.51	12	52.67 ± 12.71	10	0.7 [-3.1, 4.38]	-0.24 [-3.8, 3.34]	1.37 [-4.16, 6.91]						
R 59	R 444	R Intermediodorsal nucleus of the thalamus	13.77 ± 10.14	14	12.77 ± 6.75	14	34.49 ± 12.01	12	68.80 ± 37.54	10	0.99 [-2.82, 4.86]	-0.01 [-3.73, 3.7]	1.32 [-4.29, 6.95]						
L 59	L 444	L Intermediodorsal nucleus of the thalamus	14.24 ± 4.84	14	17.17 ± 9.43	14	40.24 ± 20.12	12	75.28 ± 44.24	10	1.3 [-2.51, 5.1]	0.28 [-3.41, 3.91]	1.04 [-4.42, 6.59]						
R 362	R 444	R Mediodorsal nucleus of thalamus	17.50 ± 7.24	15	37.72 ± 17.02	14	68.41 ± 23.15	12	88.23 ± 31.48	10	2.3 [-1.47, 6.11]	1.15 [-2.43, 4.74]	-1.16 [-6.81, 4.38]						
L 362	L 444	L Mediodorsal nucleus of thalamus	26.59 ± 12.87	15	20.76 ± 13.15	13	55.36 ± 12.84	12	103.54 ± 27.18	10	0.47 [-3.27, 4.23]	-0.24 [-3.87, 3.44]	0.88 [-4.59, 6.51]						
R 366	R 444	R Submedial nucleus of the thalamus	24.80 ± 17.01	14	15.06 ± 8.70	13	70.19 ± 20.68	11	139.98 ± 36.44	9	1.35 [-2.57, 5.31]	-0.32 [-4.02, 3.34]	2.1 [-3.64, 7.95]						
L 366	L 444	L Submedial nucleus of the thalamus	11.08 ± 4.89	14	10.99 ± 3.13	13	51.37 ± 14.37	11	159.86 ± 34.10	10	3.6 [-0.36, 7.51]	0.07 [-3.6, 3.74]	8.09 [2.37, 13.9]*	-0.03 [-3.54, 3.72]	-4.41 [-8.38, -0.53]*	-14.01 [-17.99, -10.02]*	4.41 [0.54, 8.3]*	-14 [-17.77, -9.66]*	-9.56 [-13.9, -5.55]*
R 1077	R 444	R Perireunensis nucleus	61.28 ± 32.99	15	65.79 ± 36.80	13	33.97 ± 11.08	12	39.51 ± 12.06	10	-1.05 [-4.81, 2.74]	0.06 [-3.59, 3.81]	-1.11 [-6.75, 4.57]						
L 1077	L 444	L Perireunensis nucleus	33.14 ± 16.12	15	24.84 ± 9.80	13	21.60 ± 10.12	12	68.25 ± 35.99	10	-0.94 [-4.63, 2.82]	-0.24 [-3.97, 3.36]	0.5 [-4.97, 5.98]						
R 571	R 856	R Midline group of the dorsal thalamus	29.85 ± 14.81	15	26.67 ± 10.35	15	30.75 ± 7.40	12	31.47 ± 8.98	10	-0.59 [-4.32, 3.24]	-0.09 [-3.63, 3.51]	-1.06 [-6.63, 4.39]						
L 571	L 856	L Midline group of the dorsal thalamus	23.42 ± 14.89	15	20.03 ± 8.12	15	16.76 ± 4.35	12	27.25 ± 7.77	10									
R 149	R 571	R Paraventricular nucleus of the thalamus	92.30 ± 54.15	15	81.41 ± 36.71	15	63.02 ± 27.99	12	59.50 ± 27.61	10	-0.9 [-4.75, 3.01]	-0.06 [-3.6, 3.51]	-1.14 [-6.77, 4.31]						
L 149	L 571	L Paraventricular nucleus of the thalamus	95.98 ± 67.14	15	50.63 ± 21.28	15	28.83 ± 13.40	12	75.55 ± 27.74	10	-1.29 [-5.05, 2.5]	-0.42 [-3.99, 3.08]	-0.23 [-5.78, 5.34]						
R 181	R 571	R Nucleus of reuniens	44.35 ± 17.17	15	36.03 ± 14.26	13	52.47 ± 16.04	12	77.39 ± 33.57	10	-0.43 [-4.19, 3.38]	-0.2 [-3.84, 3.44]	-0.39 [-6.04, 5.27]						
L 181	L 571	L Nucleus of reuniens	25.61 ± 11.13	15	33.55 ± 14.45	13	34.01 ± 9.60	12	47.80 ± 11.97	10	-0.28 [-4.07, 3.51]	0.32 [-3.38, 3.89]	-0.92 [-6.44, 4.74]						
R 560581559	R 571	R Xiphoid thalamic nucleus	47.39 ± 15.76	15	59.45 ± 22.53	13	74.23 ± 24.04	12	103.66 ± 26.07	10	-0.04 [-3.8, 3.75]	0.24 [-3.46, 3.85]	-0.77 [-6.42, 4.94]						
L 560581559	L 571	L Xiphoid thalamic nucleus	22.47 ± 10.35	15	48.35 ± 20.48	13	40.36 ± 25.74	12	43.45 ± 12.85	10	0.18 [-3.56, 3.92]	1.13 [-2.46, 4.75]	-2.16 [-7.66, 3.45]						
R 51	R 856	R Intralaminar nuclei of the dorsal thalamus	8.53 ± 3.34	15	12.83 ± 5.35	15	35.21 ± 5.39	12	64.34 ± 12.04	10	2.56 [-1.25, 6.36]	0.55 [-3, 4.12]	1.67 [-3.92, 7.27]						
L 51	L 856	L Intralaminar nuclei of the dorsal thalamus	6.70 ± 2.76	15	6.42 ± 1.77	15	32.21 ± 5.03	12	56.75 ± 15.41	10	3.22 [-0.52, 7.07]	0.02 [-3.5, 3.61]	2.47 [-3.06, 7.89]						
R 189	R 51	R Rhomboid nucleus	9.13 ± 3.94	15	30.63 ± 23.71	13	66.87 ± 19.53	12	154.40 ± 54.69	10	5.74 [1.96, 9.54]*	2.35 [-1.29, 6.02]	6.07 [0.4, 11.66]*	-2.3 [-5.92, 1.31]	-6.55 [-10.26, -2.76]*	-16.42 [-20.08, -12.11]*	4.24 [0.51, 8.08]*	-14.08 [-18.22, -10.11]*	-9.87 [-14.02, -5.74]*
L 189	L 51	L Rhomboid nucleus	14.29 ± 5.63	15	15.07 ± 7.73	13	83.37 ± 42.10	12	145.38 ± 46.47	10	4.26 [0.55, 7.98]*	0.03 [-3.59, 3.59]	3.14 [-2.37, 8.72]	0.03 [-3.39, 3.7]	-5.07 [-8.77, -1.41]*	-9.69 [-13.59, -5.71]*	5.08 [1.31, 8.94]*	-9.68 [-13.78, -5.76]*	-4.59 [-8.68, -0.43]*
R 599	R 51	R Central medial nucleus of the thalamus	5.54 ± 1.87	15	23.87 ± 10.81	14	41.27 ± 11.24	12	78.72 ± 30.24	10	5.85 [2.12, 9.67]*	3.3 [-0.31, 6.96]	2.29 [-3.38, 7.88]	-3.25 [-6.74, 0.29]	-6.69 [-10.36, -2.99]*	-13.67 [-17.6, -9.73]*	3.42 [-0.34, 7.04]	-10.44 [-14.26, -6.24]*	-7.03 [-11.16, -2.99]*
L 599	L 51	L Central medial nucleus of the thalamus	7.23 ± 3.19	15	21.54 ± 12.23	14	33.04 ± 10.89	12	76.12 ± 26.92	10	2.99 [-0.82, 6.64]	2.02 [-1.55, 5.54]	2.78 [-2.63, 8.25]						
R 907	R 51	R Paracentral nucleus	6.53 ± 2.92	14	16.57 ± 9.33	13	87.34 ± 20.90	11	95.51 ± 15.27	10	11.93 [8.09, 15.85]*	1.63 [-2.07, 5.33]	-1.62 [-7.47, 4.06]	-1.59 [-5.22, 2.06]	-12.76 [-16.65, -9.03]*	-14.17 [-18.07, -10.06]*	11.16 [7.57, 15.15]*	-12.61 [-16.51, -8.4]*	-1.41 [-5.63, 2.83]
L 907	L 51	L Paracentral nucleus	5.75 ± 2.52	14	3.91 ± 2.03	13	63.98 ± 20.37	12	118.79 ± 36.69	10	9.61 [5.85, 13.42]*	-0.22 [-3.88, 3.41]	8.6 [2.87, 14.21]*	0.27 [-3.36, 3.79]	-10.46 [-14.28, -6.76]*	-20.23 [-24.21, -16.22]*	10.71 [6.9, 14.44]*	-20.51 [-24.5, -16.52]*	-9.8 [-14, -5.64]*
R 575	R 51	R Central lateral nucleus of the thalamus	42.14 ± 22.93	14	53.59 ± 20.49	14	46.98 ± 12.85	12	144.27 ± 27.95	10	-0.42 [-4.18, 3.41]	0.39 [-3.17, 4.06]	0.75 [-4.84, 6.27]						
L 575	L 51	L Central lateral nucleus of the thalamus	30.41 ± 16.11	14	20.61 ± 7.83	14	48.24 ± 8.63	12	100.20 ± 33.71	10	0.07 [-3.72, 3.92]	-0.2 [-3.81, 3.52]	0.76 [-4.82, 6.35]						
R 930	R 51	R Parafascicular nucleus	9.96 ± 3.89	11	13.73 ± 8.31	10	90.52 ± 13.59	10	186.55 ± 35.66	7	8.13 [3.97, 12.31]*	0.85 [-3.26, 4.84]	8.04 [1.75, 14.34]*	-0.81 [-4.84, 3.15]	-8.94 [-13.06, -4.85]*	-19.27 [-23.71, -14.53]*	8.13 [3.89, 12.33]*	-18.46 [-23.06, -13.83]*	-10.3 [-15.1, -5.79]*
L 930	L 51	L Parafascicular nucleus	11.84 ± 5.51	10	3.78 ± 1.21	10	88.11 ± 14.82	8	116.19 ± 30.95	7	6.6 [2.16, 11]*	-0.91 [-5.13, 3.29]	2.44 [-4.01, 8.94]	0.94 [-3.28, 5.01]	-7.46 [-11.89, -3.18]*	-10.37 [-14.88, -5.89]*	8.39 [4, 12.74]*	-11.38 [-15.81, -6.48]*	-2.95 [-7.66, 1.94]

R 262	R 856	R Reticular nucleus of the thalamus	147.28 ± 98.89	15	150.34 ± 69.44	14	67.19 ± 9.27	12	88.68 ± 12.28	10	-1.14 [-4.95, 2.63]	0.03 [-3.51, 3.65]	-1.03 [-6.64, 4.55]						
L 262	L 856	L Reticular nucleus of the thalamus	38.38 ± 20.08	15	20.33 ± 9.57	14	35.30 ± 9.20	12	54.39 ± 11.66	10	-0.67 [-4.46, 3.11]	-0.48 [-4.07, 3.12]	-0.19 [-5.79, 5.51]						
R 1014	R 856	R Geniculate group, ventral thalamus	204.26 ± 149.45	13	131.01 ± 85.05	15	131.50 ± 42.87	12	147.24 ± 56.70	9	-0.83 [-4.66, 3.09]	-0.17 [-3.82, 3.43]	0.09 [-5.72, 5.79]						
L 1014	L 856	L Geniculate group, ventral thalamus	24.28 ± 10.71	14	61.35 ± 45.68	15	37.65 ± 9.63	12	97.66 ± 51.54	10	-0.01 [-3.83, 3.83]	1.65 [-2.05, 5.13]	-0.32 [-5.84, 5.34]						
R 27	R 1014	R Intergeniculate leaflet of the lateral geniculate complex	699.24 ± 651.07	9	337.85 ± 170.18	12	152.71 ± 67.90	10	634.83 ± 297.17	8	-1.16 [-5.55, 3.19]	-0.49 [-4.68, 3.68]	1.12 [-5.14, 7.3]						
L 27	L 1014	L Intergeniculate leaflet of the lateral geniculate complex	162.58 ± 132.15	12	37.19 ± 24.92	10	87.57 ± 46.12	10	66.59 ± 19.88	8	-0.96 [-5.07, 3.25]	0.06 [-4.02, 4.08]	0.16 [-6, 6.37]						
R 178	R 1014	R Ventral part of the lateral geniculate complex	422.46 ± 314.26	13	247.26 ± 178.69	15	264.47 ± 87.31	12	272.06 ± 109.51	9	-0.84 [-4.69, 3.07]	-0.25 [-3.81, 3.43]	0.11 [-5.44, 5.76]						
L 178	L 1014	L Ventral part of the lateral geniculate complex	48.55 ± 23.98	14	122.35 ± 91.96	15	78.23 ± 19.60	12	202.64 ± 102.79	10	0.07 [-3.75, 3.83]	1.63 [-1.94, 5.24]	-0.2 [-5.79, 5.4]						
L 321	L 1014	L Subgeniculate nucleus	85.30 ± 55.75	8	16.92 ± 9.46	10	39.26 ± 24.73	9	61.06 ± 28.13	8	-0.98 [-5.57, 3.58]	-0.48 [-4.83, 3.86]	0.85 [-5.41, 7.31]						
R 958	R 856	R Epithalamus	49.64 ± 21.98	15	69.94 ± 33.29	15	46.02 ± 15.49	12	76.87 ± 16.73	10	-0.68 [-4.31, 2.98]	0.48 [-3.03, 4.07]	-1 [-6.55, 4.59]						
L 958	L 856	L Epithalamus	25.39 ± 6.95	15	17.04 ± 4.36	15	36.30 ± 15.33	12	67.75 ± 30.83	10	-0.16 [-3.91, 3.65]	-0.28 [-3.78, 3.27]	0.36 [-5.17, 5.83]						
R 483	R 958	R Medial habenula	92.77 ± 51.22	15	129.91 ± 50.93	15	113.12 ± 37.60	12	170.17 ± 41.54	10	-0.36 [-4.14, 3.42]	0.45 [-3.03, 4.06]	-1.01 [-6.5, 4.44]						
L 483	L 958	L Medial habenula	50.41 ± 13.56	15	36.77 ± 10.69	15	122.13 ± 55.55	12	204.29 ± 98.23	10	0.83 [-2.93, 4.59]	-0.25 [-3.73, 3.35]	0.7 [-4.92, 6.22]						
R 186	R 958	R Lateral habenula	99.49 ± 41.89	15	187.00 ± 133.69	14	68.20 ± 31.88	12	135.10 ± 34.78	10	-0.91 [-4.65, 2.87]	0.93 [-2.59, 4.55]	-1.42 [-7.03, 4.1]						
L 186	L 958	L Lateral habenula	47.05 ± 18.22	15	26.02 ± 7.44	14	17.33 ± 4.61	12	70.46 ± 35.80	10	-1.24 [-4.98, 2.5]	-0.39 [-3.97, 3.24]	0.38 [-5.03, 5.89]						
R 1097	R 1129	R Hypothalamus	28.16 ± 12.18	14	25.05 ± 5.54	14	32.63 ± 8.05	12	44.52 ± 8.01	10									
L 1097	L 1129	L Hypothalamus	25.06 ± 14.00	14	11.80 ± 2.92	13	18.49 ± 4.27	12	29.52 ± 4.80	10	-0.77 [-2.52, 1.05]	-0.32 [-2.13, 1.42]	0.05 [-2.58, 2.72]						
R 157	R 1097	R Periventricular zone	72.81 ± 35.11	14	27.51 ± 8.36	14	27.19 ± 13.82	12	76.25 ± 22.27	10	-1.13 [-2.88, 0.64]	-0.48 [-2.21, 1.21]	0.44 [-2.22, 3.06]						
L 157	L 1097	L Periventricular zone	66.69 ± 39.27	15	25.96 ± 7.32	14	19.00 ± 6.70	12	55.92 ± 17.27	10	-1.25 [-3.03, 0.52]	-0.5 [-2.25, 1.24]	0.35 [-2.29, 3]						
R 390	R 157	R Supraoptic nucleus	47.81 ± 16.96	10	35.43 ± 21.53	7	97.29 ± 36.14	11	44.67 ± 33.88	6	0.11 [-1.81, 2.02]	-0.33 [-2.45, 1.75]	-1.49 [-4.61, 1.64]						
R 38	R 157	R Paraventricular hypothalamic nucleus	35.09 ± 15.33	13	30.70 ± 15.32	8	23.50 ± 5.32	12	44.11 ± 12.91	8	-0.81 [-2.61, 0.99]	0.31 [-1.66, 2.29]	-0.18 [-3.08, 2.66]						
L 38	L 157	L Paraventricular hypothalamic nucleus	41.95 ± 17.23	13	25.10 ± 18.09	8	18.40 ± 5.21	12	46.33 ± 13.36	8	-1.04 [-2.84, 0.79]	0.04 [-1.93, 2.04]	0.15 [-2.71, 2.99]						
R 118	R 157	R Periventricular hypothalamic nucleus, intermediate part	38.99 ± 21.20	13	22.20 ± 8.93	14	10.03 ± 2.78	12	41.25 ± 18.36	10	-1.18 [-2.97, 0.65]	-0.22 [-1.99, 1.53]	0.31 [-2.33, 2.96]						
L 118	L 157	L Periventricular hypothalamic nucleus, intermediate part	43.35 ± 23.12	13	20.81 ± 8.37	14	7.00 ± 2.03	12	32.33 ± 10.36	10	-1.26 [-3.07, 0.58]	-0.31 [-2.06, 1.46]	0.17 [-2.5, 2.8]						
R 223	R 157	R Arcuate hypothalamic nucleus	302.37 ± 132.61	13	85.80 ± 26.59	13	251.83 ± 207.61	11	279.64 ± 84.26	9	-0.57 [-2.44, 1.25]	-0.46 [-2.23, 1.31]	-0.21 [-2.87, 2.51]						
L 223	L 157	L Arcuate hypothalamic nucleus	318.68 ± 171.74	13	92.06 ± 29.42	14	189.43 ± 124.48	10	193.24 ± 56.31	9	-0.84 [-2.71, 1.03]	-0.52 [-2.3, 1.25]	-0.22 [-2.98, 2.54]						
R 141	R 1097	R Periventricular region	63.80 ± 32.39	15	44.58 ± 24.72	15	23.24 ± 6.33	12	40.48 ± 12.00	10	-1.16 [-2.87, 0.6]	-0.21 [-1.89, 1.5]	-0.25 [-2.85, 2.36]						
L 141	L 1097	L Periventricular region	86.06 ± 40.49	14	30.77 ± 14.28	15	15.53 ± 6.08	12	35.96 ± 8.50	10	-1.32 [-3.1, 0.43]	-0.53 [-2.26, 1.14]	0.05 [-2.55, 2.7]						
R 830	R 141	R Dorsomedial nucleus of the hypothalamus	98.32 ± 60.62	12	57.27 ± 21.03	13	23.46 ± 4.79	11	41.48 ± 13.08	9	-1.12 [-2.97, 0.77]	-0.21 [-2.03, 1.64]	-0.38 [-3.15, 2.4]						
L 830	L 141	L Dorsomedial nucleus of the hypothalamus	70.98 ± 53.60	12	37.20 ± 20.76	13	14.88 ± 4.13	10	30.42 ± 11.27	9	-1.15 [-3.06, 0.76]	-0.27 [-2.09, 1.56]	-0.29 [-3.09, 2.51]						
R 126	R 141	R Periventricular hypothalamic nucleus, posterior part	312.33 ± 199.14	9	148.59 ± 112.61	9	97.40 ± 49.60	6	147.55 ± 75.43	8	-1.22 [-3.48, 1]	-0.08 [-2.14, 1.97]	-0.12 [-3.23, 3.03]						
L 126	L 141	L Periventricular hypothalamic nucleus, posterior part	378.70 ± 196.82	9	152.59 ± 98.81	9	115.81 ± 59.39	6	131.20 ± 44.61	8	-1.25 [-3.5, 1.01]	-0.16 [-2.23, 1.86]	-0.14 [-3.25, 3.03]						
R 347	R 141	R Subparaventricular zone	33.91 ± 23.07	13	26.96 ± 14.59	8	10.00 ± 3.96	12	28.46 ± 12.82	8	-1.16 [-2.95, 0.66]	0.25 [-1.7, 2.25]	-0.18 [-3.05, 2.68]						
L 347	L 141	L Subparaventricular zone	28.36 ± 21.05	13	20.04 ± 8.81	8	18.88 ± 6.59	11	39.34 ± 21.22	7	-0.84 [-2.67, 0.99]	0.17 [-1.81, 2.14]	0.36 [-2.59, 3.36]						
R 467	R 1097	R Hypothalamic medial zone	40.12 ± 18.14	15	36.63 ± 9.43	15	50.24 ± 17.65	12	50.79 ± 8.62	10	-0.26 [-2.02, 1.56]	0.02 [-1.67, 1.73]	-0.73 [-3.37, 1.9]						
L 467	L 1097	L Hypothalamic medial zone	35.50 ± 22.22	15	20.25 ± 5.68	15	23.48 ± 8.93	12	34.73 ± 6.82	10	-0.87 [-2.63, 0.91]	-0.35 [-2.06, 1.35]	-0.04 [-2.69, 2.58]						
R 88	R 467	R Anterior hypothalamic nucleus	32.01 ± 13.11	15	27.06 ± 8.29	11	46.07 ± 10.36	12	46.34 ± 17.25	8	-0.08 [-1.82, 1.72]	0.02 [-1.8, 1.86]	-0.48 [-3.22, 2.28]						
L 88	L 467	L Anterior hypothalamic nucleus	16.06 ± 6.92	14	28.22 ± 13.25	11	20.19 ± 4.94	12	25.68 ± 7.62	9	-0.21 [-1.99, 1.6]	0.89 [-0.92, 2.76]	-1.19 [-3.94, 1.53]						
R 331	R 467	R Mammillary body	72.82 ± 26.42	9	73.02 ± 18.50	12	101.54 ± 52.67	10	107.91 ± 27.22	7	0.16 [-1.86, 2.16]	0.27 [-1.68, 2.24]	-0.69 [-3.65, 2.28]						
L 331	L 467	L Mammillary body	61.08 ± 36.25	10	22.45 ± 5.47	11	53.35 ± 28.51	10	79.23 ± 23.24	8	-0.37 [-2.33, 1.61]	-0.22 [-2.17, 1.69]	-0.04 [-2.97, 2.85]						
R 210	R 331	R Lateral mammillary nucleus	108.63 ± 23.21	6	69.04 ± 40.90	7	46.56 ± 27.81	7	116.18 ± 20.62	7	-0.6 [-2.93, 1.77]	0.43 [-1.96, 2.8]	-0.12 [-3.46, 3.21]						
L 210	L 331	L Lateral mammillary nucleus	175.64 ± 131.22	7	32.86 ± 17.39	8	65.32 ± 49.93	7	58.14 ± 16.55	8	-0.85 [-3.11, 1.41]	-0.26 [-2.46, 1.92]	-0.3 [-3.5, 2.84]						
R 491	R 331	R Medial mammillary nucleus	57.56 ± 15.88	7	25.01 ± 10.45	9	105.50 ± 70.20	6	147.44 ± 30.48	7	0.55 [-1.85, 2.92]	0.05 [-2.15, 2.19]	0.41 [-2.84, 3.68]						
L 491	L 331	L Medial mammillary nucleus	39.65 ± 16.02	7	12.27 ± 9.40	8	81.71 ± 46.24	6	100.44 ± 13.74	7	0.78 [-1.61, 3.16]	-0.13 [-2.34, 2.11]	0.34 [-3, 3.69]						
R 732	R 491	R Medial mammillary nucleus, median part	80.71 ± 41.99	7	67.75 ± 43.24	8	186.65 ± 131.35	6	169.94 ± 63.25	7	1.04 [-1.31, 3.41]	0.42 [-1.86, 2.65]	-0.89 [-4.22, 2.47]						
L 732	L 491	L Medial mammillary nucleus, median part	80.55 ± 42.06	7	7.02 ± 7.02	8	142.80 ± 86.57	6	100.13 ± 33.49	7	0.49 [-1.79, 2.81]	-0.34 [-2.56, 1.85]	-0.45 [-3.7, 2.81]						
R 606826647	R 491	R Medial mammillary nucleus, lateral part	154.69 ± 40.16	7	77.25 ± 40.46	9	201.72 ± 127.50	6	257.59 ± 71.80	7	0.01 [-2.33, 2.37]	0.11 [-2.05, 2.32]	-0.01 [-3.33, 3.21]						
L 606826647	L 491	L Medial mammillary nucleus, lateral part	75.70 ± 30.84	7	18.15 ± 11.53	8	112.24 ± 63.78	6	177.88 ± 22.02	7	0.19 [-2.15, 2.54]	-0.19 [-2.43, 2.05]	0.8 [-2.5, 4.15]						
R 606826651	R 491	R Medial mammillary nucleus, medial part	94.27 ± 42.52	7	26.23 ± 21.29	9	215.63 ± 140.85	6	414.54 ± 102.92	6	1 [-1.33, 3.33]	-0.1 [-2.29, 2.04]	2.2 [-1.1, 5.55]						
L 606826651	L 491	L Medial mammillary nucleus, medial part	79.06 ± 36.07	7	47.19 ± 36.38	8	219.77 ± 131.63	6	309.25 ± 61.29	6	1.5 [-0.84, 3.81]	0.17 [-2.02, 2.35]	0.96 [-2.39, 4.25]						
R 525	R 331	R Supramammillary nucleus	428.91 ± 196.29	8	406.35 ± 130.90	9	807.88 ± 424.70	8	588.50 ± 212.44	7	0.64 [-1.53, 2.77]	0.44 [-1.7, 2.58]	-1.37 [-4.45, 1.77]						
L 525	L 331	L Supramammillary nucleus	397.32 ± 248.17	9	133.36 ± 35.24	9	386.22 ± 229.90	9	344.77 ± 184.63	8	-0.32 [-2.34, 1.72]	-0.22 [-2.23, 1.84]	-0.5 [-3.44, 2.5]						
R 557	R 331	R Tuberomammillary nucleus	35.13 ± 24.15	8	68.58 ± 25.03	11	18.54 ± 6.66	8	13.85 ± 2.31	6	-0.77 [-2.9, 1.41]	1.28 [-0.77, 3.28]	-1.7 [-4.79, 1.4]						
L 557	L 331	L Tuberomammillary nucleus	48.60 ± 40.88	10	44.08 ± 22.29	9	7.85 ± 3.50	8	17.46 ± 8.24	7	-1.18 [-3.23, 0.89]	0.31 [-1.7, 2.3]	-0.64 [-3.69, 2.42]						
R 1	R 557	R Tuberomammillary nucleus, ventral part	28.87 ± 14.55	7	134.14 ± 62.53	9	40.66 ± 15.37	8	32.81 ± 5.69	6	-0.43 [-2.65, 1.78]	3.45 [1.25, 5.62]*	-3.98 [-7.19, -0.74]*	-3.35 [-5.45, -1.3]*	-0.17 [-2.25, 2]	-0.44 [-2.75, 1.86]	-3.18 [-5.21, -1.25]*	2.92 [0.66, 5.07]*	-0.27 [-2.5, 1.96]
L 1	L 557	L Tuberomammillary nucleus, ventral part	103.48 ± 84.74	10	25.98 ± 19.07	7	12.90 ± 4.04	8	19.98 ± 16.55	7	-1.2 [-3.28, 0.82]	-0.21 [-2.37, 1.91]	-0.25 [-3.31, 2.87]						
L 980	L 467	L Dorsal premammillary nucleus	213.87 ± 180.59	10	61.05 ± 34.13	8	23.91 ± 12.21	7	70.39 ± 15.60	8	-1.33 [-3.45, 0.81]	-0.23 [-2.27, 1.86]	-0.01 [-3.09, 3.08]						
R 1004	R 467	R Ventral premammillary nucleus	266.08 ± 241.83	8	120.94 ± 50.97	10	40.36 ± 13.60	8	76.88 ± 33.43	7	-1.15 [-3.29, 1.03]	-0.27 [-2.35, 1.81]	-0.09 [-3.17, 2.98]						
L 1004	L 467	L Ventral premammillary nucleus	245.89 ± 184.72	10	68.42 ± 26.38	9	23.91 ± 9.66	9	74.02 ± 20.23	8	-1.17 [-3.18, 0.82]	-0.31 [-2.3, 1.74]	-0.14 [-3.15, 2.83]						
R 63	R 467	R Paraventricular hypothalamic nucleus, descending division	62.09 ± 32.35	13	55.39 ± 26.26	11	18.38 ± 8.33	10	48.67 ± 17.75	9	-1.17 [-3.01, 0.71]	0.14 [-1.71, 1.98]	-0.27 [-3.03, 2.48]						
L 63	L 467	L Paraventricular hypothalamic nucleus, descending division	33.70 ± 21.58	12	16.24 ± 8.64	11	28.75 ± 14.78	10	21.69 ± 12.73	8	-0.72 [-2.59, 1.18]	-0.37 [-2.24, 1.53]	-0.35 [-3.24, 2.52]						
R 693	R 467	R Ventromedial hypothalamic nucleus	73.58 ± 35.94	14	26.33 ± 13.13	12	69.23 ± 42.28	11	55.33 ± 11.99	9	-0.55 [-2.37, 1.29]	-0.39 [-2.2, 1.4]	-0.51 [-3.25, 2.24]						
L 693	L 467	L Ventromedial hypothalamic nucleus	64.66 ± 32.39	14	31.33 ± 15.55	12	33.32 ± 23.51	10	21.61 ± 5.55	9	-0.93 [-2.76, 0.97]	-0.26 [-2.03, 1.52]	-0.68 [-3.46, 2.09]						
R 946	R 467	R Posterior hypothalamic nucleus	126.67 ± 72.67	14	106.33 ± 57.44	14	97.26 ± 32.76	10	92.86 ± 29.33	8	-0.68 [-2.54, 1.16]	-0.06 [-1.76, 1.65]	-0.65 [-3.43, 2.13]						
L 946	L 467	L Posterior hypothalamic nucleus	109.07 ± 75.76	14	51.35 ± 22.42	14	42.09 ± 11.66	10	52.51 ± 9.50	8	-1.04 [-2.93, 0.82]	-0.42 [-2.15, 1.32]	-0.17 [-2.91, 2.57]						
R 290	R 1097	R Hypothalamic lateral zone	23.87 ± 8.50	15	23.20 ± 5.92	15	44.06 ± 8.71	12	61.49 ± 12.83	10	0.32 [-1.46, 2.07]	0.06 [-1.64, 1.75]	-0.05 [-2.68, 2.54]						
L 290	L 1097	L Hypothalamic lateral zone	16.48 ± 8.91	15	11.98 ± 2.79	15	28.02 ± 4.98	12	40.68 ± 7.00	10	0.17 [-1.63, 1.94]	-0.19 [-1.91, 1.49]	0.25 [-2.38, 2.88]						
R 194	R 290	R Lateral hypothalamic area	50.26 ± 16.54	15	69.22 ± 13.30	15	93.28 ± 21.54	10	105.23 ± 24.49	10	0.28 [-1.52, 2.11]	0.47 [-1.23, 2.19]	-0.88 [-3.55, 1.79]						
L 194	L 290	L Lateral hypothalamic area	37.01 ± 21.70	15	40.30 ± 9.14	15	44.74 ± 11.43	11	61.46 ± 17.65	10	-0.39 [-2.18, 1.41]	0.16 [-1.53, 1.84]	-0.36 [-2.96, 2.31]						
R 364	R 290	R Parasubthalamic nucleus	58.49 ± 36.83	12	34.61 ± 11.23	12	54.60 ± 16.58	10	110.18 ± 46.03	8	-0.42 [-2.32, 1.49]	-0.22 [-2.05, 1.62]	0.48 [-2.39, 3.32]						
L 364	L 290	L Parasubthalamic nucleus	66.15 ± 49.11	12	15.26 ± 4.77	12	37.78 ± 14.03	10	51.80 ± 21.80	8	-0.75 [-2.66, 1.14]	-0.38 [-2.22, 1.43]	-0.08 [-2.89, 2.73]						
R 576073704	R 290	R Perifornical nucleus	35.07 ± 10.31	15	41.01 ± 12.34	13	125.40 ± 37.00	12	75.59 ± 21.68	9	2.06 [0.31, 3.8]*	0.33 [-1.43, 2.08]	-2.43 [-5.11, 0.23]	-0.25 [-1.89, 1.35]	-2.67 [-4.32, -1.01]*	-1.37 [-3.15, 0.49]	2.42 [0.71, 4.12]*	-1.12 [-3, 0.73]	1.32 [-0.59, 3.15]
L 576073704	L 290	L Perifornical nucleus	22.84 ± 10.08	15	13.52 ± 5.12	13	41.68 ± 11.09	12	42.27 ± 10.53	8	0.3 [-1.46, 2]	-0.24 [-2.01, 1.5]	-0.31 [-2.97, 2.47]						
R 470	R 290	R Subthalamic nucleus	3.38 ± 2.00	12	7.28 ± 2.38	12	51.64 ± 13.72	12	94.00 ± 27.40	8	13.89 [12.08, 15.73]*	1.39 [-0.49, 3.24]	10.49 [7.71, 13.23]*	-1.3 [-3.06, 0.4]	-14.5 [-16.21, -12.76]*	-27.16 [-29.03, -25.19]*	13.2 [11.38, 14.9]*	-25.86 [-27.88, -24.01]*	-12.67 [-14.55, -10.7]*
L 470	L 290	L Subthalamic nucleus	27.44 ± 24.44	10	8.09 ± 3.51	15	44.92 ± 10.07	11	88.79 ± 27.16	9	0.13 [-1.81, 2.07]	-0.59 [-2.43, 1.25]	1.45 [-1.31, 4.23]						
R 614	R 290	R Tuberal nucleus	78.43 ± 41.56	14	39.17 ± 15.23	13	48.96 ± 18.53	10	86.11 ± 21.49	9	-0.81 [-2.66, 1.04]	-0.27 [-2.05, 1.48]	-0.02 [-2.71, 2.75]						
L 614	L 290	L Tuberal nucleus	78.77 ± 50.12	14	24.59 ± 9.62	13	32.32 ± 8.79	10	41.42 ± 7.67	9	-1.04 [-2.91, 0.86]	-0.47 [-2.24, 1.31]	-0.18 [-2.94, 2.55]						
R 797	R 290	R Zona incerta	35.59 ± 12.19	15	43.43 ± 17.94	15	110.49 ± 20.98	12	142.45 ± 33.21	10	1.58 [-0.23, 3.38]	0.3 [-1.41, 2.01]	-0.12 [-2.7, 2.49]						
L 797	L 290	L Zona incerta	17.43 ± 6.44	15	13.00 ± 3.81	15	82.26 ± 12.95	12	119.90 ± 24.67	10	3.18 [1.41, 4.98]*	-0.17 [-1.89, 1.53]	1.62 [-1, 4.25]	0.25 [-1.3, 1.86]	-3.79 [-5.44, -2.12]*	-6.03 [-7.75, -4.29]*	4.05 [2.34, 5.69]*	-6.29 [-8.05, -4.54]*	-2.25 [-4.06, -0.36]*
R 804	R 797	R Fields of Forel	21.56 ± 14.47	10	6.13 ± 2.17	10	116.28 ± 27.14	8	159.17 ± 40.25	8	4.03 [1.98, 6.08]*	-0.27 [-2.25, 1.66]	1.7 [-1.25, 4.59]	0.35 [-1.42, 2.25]	-4.64 [-6.67, -2.76]*	-6.86 [-8.81, -4.89]*	5 [3.03, 7]*	-7.23 [-9.16, -5.23]*	-2.22 [-4.28, -0.15]*
L 804	L 797	L Fields of Forel	14.47 ± 9.86	10	6.76 ± 3.09	9	80.49 ± 22.78	9	147.48 ± 30.36	8	4.3 [2.33, 6.31]*	-0.11 [-2.11, 1.89]	4.09 [1.11, 7.09]*	0.21 [-1.69, 2.05]	-4.9 [-6.82, -3.04]*	-9.69 [-11.66, -7.63]*	5.11 [3.1, 7.03]*	-9.87 [-11.91, -7.86]*	-4.78 [-6.79, -2.7]*
R 10671	R 1097	R Median eminence	370.20 ± 175.50	12	123.49 ± 60.37	13	86.38 ± 37.80	9	527.81 ± 229.09	9	-1.13 [-3.06, 0.81]	-0.5 [-2.32, 1.35]	0.89 [-1.91, 3.69]						
L 10671	L 1097	L Median eminence	388.51 ± 217.62	12	165.39 ± 117.44	13	166.01 ± 141.29	10	633.30 ± 276.05	9	-0.91 [-2.82, 0.95]	-0.39 [-2.21, 1.41]	0.78 [-1.93, 3.55]						
R 313	R 343	R Midbrain	46.07 ± 33.32	13	14.19 ± 5.33	12	26.34 ± 7.51	12	70.78 ± 26.74	8	-1.17 [-6.25, 3.98]	-0.24 [-5.42, 4.92]	-0.44 [-8.39, 7.35]						
L 313	L 343	L Midbrain	17.42 ± 9.95	13	15.86 ± 10.31	14	19.87 ± 5.91	12	41.98 ± 10.21	9	-0.61 [-5.79, 4.66]	0.04 [-4.99, 5.15]	-0.42 [-8.19, 7.3]						
R 339	R 313	R Midbrain, sensory related	7.20 ± 4.90	11	18.73 ± 6.44	10	55.32 ± 31.88	10	17.40 ± 7.05	7	6.05 [0.33, 11.8]*	1.74 [-3.98, 7.35]	-8.49 [-17.07, 0.04]	-1.71 [-7.26, 3.89]	-6.98 [-12.24, -1]*	-1.92 [-8.18, 4.33]	5.28 [-0.45, 11.23]	-0.27 [-6.74, 5.8]	4.95 [-1.25, 11.54]
L 339	L 313	L Midbrain, sensory related	8.74 ± 7.90	11	8.88 ± 3.23	10	119.58 ± 79.23	10	15.52 ± 5.10	7	11.99 [6.2, 17.72]*	0.19 [-5.62, 5.86]	-13.5 [-22.14, -4.77]*	-0.17 [-5.77, 5.64]	-12.91 [-18.61, -7.33]*	-1.32 [-7.46, 4.8]	12.77 [7.02, 18.41]*	-1.18 [-7.34, 5.15]	11.57 [5.19, 17.89]*
R 599626923	R 339	R Subcommissural organ	51.95 ± 51.95	11	0.00 ± 0.00	8	130.94 ± 62.43	10	0.00 ± 0.00	7	0.88 [-4.8, 6.49]	-0.42 [-6.43, 5.53]	-3.54 [-12.18, 5.03]						
L 599626923	L 339	L Subcommissural organ	23.27 ± 23.27	11	0.00 ± 0.00	8	473.17 ± 332.90	10	31.32 ± 23.01	7	18.66 [12.92, 24.39]*	-0.42 [-6.43, 5.5]	-19.97 [-28.77, -11.26]*	0.49 [-5.57, 6.24]	-19.54 [-25.34, -14.09]*	-0.94 [-7.21, 5.23]	20.06 [13.81, 25.99]*	-1.37 [-7.93, 5.29]	18.66 [12.36, 24.87]*
R 323	R 313	R Midbrain, motor related	49.73 ± 43.16	13	20.43 ± 8.81	13	26.14 ± 6.98	12	74.73 ± 31.40	8									
L 323	L 313	L Midbrain, motor related	16.78 ± 10.94	13	3.79 ± 1.17	14	14.07 ± 4.83	12	23.96 ± 5.13	8	-0.9 [-6.1, 4.22]	-0.67 [-5.64, 4.36]	-0.39 [-8.19, 7.55]						
L 381	L 323	L Substantia nigra, reticular part	29.35 ± 27.22	6	3.91 ± 2.69	7	10.73 ± 4.94	7	22.81 ± 10.68	7	-1.22 [-8.37, 5.95]	-0.58 [-7.58, 6.57]	-0.72 [-10.7, 9.41]						
R 128	R 323	R Midbrain reticular nucleus	18.86 ± 14.81	8	13.13 ± 7.73	9	74.69 ± 34.09	9	97.43 ± 16.67	6	1.8 [-4.49, 8.09]	-0.61 [-6.88, 5.72]	0.66 [-8.74, 10.02]						
L 128	L 323	L Midbrain reticular nucleus	12.61 ± 7.67	8	6.14 ± 2.73	9	107.45 ± 49.14	9	115.01 ± 28.23	7	6.43 [0.21, 12.75]*	-0.75 [-6.98, 5.5]	0.02 [-9.05, 8.88]	0.79 [-5.39, 6.89]	-7.32 [-13.7, -1.44]*	-8.34 [-14.93, -1.87]*	8.13 [2.12, 13.97]*	-9.1 [-15.73, -2.87]*	-0.99 [-7.34, 5.51]
R 795	R 323	R Periaqueductal gray	60.32 ± 44.36	13	19.42 ± 10.33	13	25.90 ± 6.13	12	34.38 ± 6.72	8	-1.34 [-6.53, 3.96]	-0.55 [-5.7, 4.67]	-0.92 [-8.75, 6.94]						
L 795	L 323	L Periaqueductal gray	32.38 ± 17.10	13	9.55 ± 3.64	13	15.82 ± 6.06	12	24.34 ± 4.73	8	-1.25 [-6.43, 4.05]	-0.59 [-5.79, 4.57]	-0.83 [-8.75, 7.23]						
R 50	R 795	R Precommissural nucleus	129.02 ± 101.46	10	54.64 ± 41.27	12	49.27 ± 14.22	12	44.49 ± 16.18	8	-1.2 [-6.81, 4.35]	-0.22 [-5.77, 5.23]	-1.46 [-9.66, 6.88]						
L 50	L 795	L Precommissural nucleus	49.97 ± 29.18	10	12.55 ± 6.94	12	25.40 ± 14.83	12	109.77 ± 88.66	8	-1.11 [-6.73, 4.54]	-0.45 [-6.11, 5.18]	0.52 [-7.71, 8.76]						
R 1100	R 323	R Pretectal region	107.69 ± 100.48	11	388.61 ± 349.69	11	53.19 ± 18.65	11	266.43 ± 193.53	8	-1.19 [-6.82, 4.35]	2.8 [-2.61, 8.36]	-2.46 [-10.57, 5.76]						
L 1100	L 323	L Pretectal region	28.89 ± 21.88	12	4.88 ± 1.61	10	22.25 ± 6.76	10	37.75 ± 8.15	7	-0.92 [-6.48, 4.68]	-0.4 [-5.88, 5.08]	-0.52 [-9.07, 8.03]						
R 215	R 1100	R Anterior pretectal nucleus	235.65 ± 224.06	11	753.95 ± 700.98	11	109.58 ± 37.15	11	559.69 ± 385.94	8	-1.24 [-6.85, 4.26]	2.38 [-3.1, 7.94]	-2.08 [-10.35, 6.15]						
L 215	L 1100	L Anterior pretectal nucleus	54.45 ± 44.47	12	9.32 ± 3.19	10	54.50 ± 17.16	10	86.13 ± 19.55	7	-0.71 [-6.33, 4.85]	-0.4 [-5.92, 5.13]	-0.45 [-9.02, 8.18]						
R 628	R 1100	R Nucleus of the optic tract	37.68 ± 19.92	8	531.48 ± 325.14	7	242.80 ± 128.48	8	48.75 ± 27.27	7	3.95 [-2.45, 10.48]	13.29 [6.64, 20.02]*	-19.37 [-28.83, -9.78]*	-13.23 [-19.88, -6.63]*	-4.85 [-11.5, 1.35]	-0.49 [-7.21, 5.94]	-8.4 [-15.02, -1.61]*	12.72 [5.58, 19.3]*	4.37 [-2.31, 11.02]
L 628	L 1100	L Nucleus of the optic tract	24.98 ± 9.95	8	8.54 ± 6.16	7	27.67 ± 12.32	9	44.98 ± 22.42	7	-1.07 [-7.48, 5.18]	-0.42 [-7.12, 6.27]	-0.11 [-9.39, 9.54]						
R 634	R 1100	R Nucleus of the posterior commissure	169.70 ± 152.90	9	17.55 ± 5.16	7	25.81 ± 16.08	8	50.26 ± 17.86	7	-1.91 [-8.2, 4.36]	-0.26 [-6.83, 6.33]	-0.51 [-9.93, 8.99]						
L 634	L 1100	L Nucleus of the posterior commissure	83.32 ± 56.59	9	10.91 ± 5.09	7	27.19 ± 10.37	8	46.40 ± 16.32	7	-1.75 [-8.07, 4.7]	-0.23 [-6.72, 6.25]	-0.46 [-9.77, 8.88]						
R 706	R 1100	R Olivary pretectal nucleus	169.57 ± 123.91	8	67.76 ± 30.35	7	52.99 ± 24.74	7	175.73 ± 133.90	7	-2.08 [-8.76, 4.56]	0.05 [-6.55, 6.49]	0.11 [-9.5, 9.73]						
L 706	L 1100	L Olivary pretectal nucleus	51.72 ± 46.14	6	21.61 ± 13.07	7	23.63 ± 9.81	6	42.37 ± 21.85	7	-2.59 [-10.03, 4.84]	-0.35 [-7.51, 6.76]	0.34 [-9.97, 10.44]						
L 1061	L 1100	L Posterior pretectal nucleus	10.80 ± 4.26	8	6.82 ± 3.66	7	22.34 ± 11.49	8	41.74 ± 22.01	7	-0.33 [-6.57, 5.99]	-0.17 [-6.71, 6.42]	0.93 [-8.39, 10.27]						
R 1009	R 997	R fiber tracts	49.43 ± 11.92	15	44.88 ± 8.45	15	143.96 ± 21.77	12	179.27 ± 25.61	10	1.7 [-0.33, 3.66]	-0.03 [-1.91, 1.8]	0.15 [-2.71, 3.12]						
L 1009	L 997	L fiber tracts	12.93 ± 4.34	15	11.90 ± 2.84	15	24.10 ± 5.18	12	52.17 ± 9.51	10	0.65 [-1.32, 2.59]	-0.01 [-1.9, 1.86]	1.6 [-1.31, 4.5]						
R 967	R 1009	R cranial nerves	26.62 ± 11.38	15	25.26 ± 7.47	15	17.63 ± 6.33	12	57.56 ± 17.62	10	-0.54 [-2.51, 1.41]	0.02 [-1.86, 1.9]	0.88 [-2.02, 3.83]						
L 967	L 1009	L cranial nerves	18.92 ± 8.80	15	8.96 ± 3.61	15	13.68 ± 4.13	12	86.71 ± 39.32	10	-0.49 [-2.46, 1.49]	-0.45 [-2.25, 1.41]	3.73 [0.8, 6.58]*	0.54 [-1.16, 2.26]	0.1 [-1.83, 1.91]	-3.82 [-5.76, -1.91]*	0.44 [-1.44, 2.31]	-4.36 [-6.35, -2.47]*	-3.91 [-6.04, -1.89]*
R 840	R 967	R olfactory nerve	24.71 ± 15.28	15	10.31 ± 3.73	15	6.65 ± 1.18	12	26.08 ± 12.27	10	-0.94 [-2.93, 1]	-0.5 [-2.36, 1.36]	0.7 [-2.22, 3.65]						
L 840	L 967	L olfactory nerve	11.90 ± 4.32	15	9.64 ± 2.28	15	7.23 ± 2.47	12	25.95 ± 11.60	10	-0.6 [-2.59, 1.39]	-0.11 [-2.03, 1.77]	1.1 [-1.86, 4.03]						
R 21	R 840	R lateral olfactory tract, general	52.27 ± 36.18	15	18.84 ± 7.78	15	7.62 ± 2.19	12	29.99 ± 18.63	10	-1.05 [-3.01, 0.9]	-0.55 [-2.43, 1.33]	0.38 [-2.6, 3.28]						
L 21	L 840	L lateral olfactory tract, general	18.61 ± 7.10	15	16.48 ± 6.18	15	7.58 ± 3.95	12	13.55 ± 6.23	10									
R 665	R 21	R lateral olfactory tract, body	104.53 ± 72.36	15	37.68 ± 15.56	15	15.23 ± 4.37	12	59.97 ± 37.26	10	-1.07 [-3.03, 0.89]	-0.57 [-2.46, 1.28]	0.43 [-2.57, 3.37]						
L 665	L 21	L lateral olfactory tract, body	37.23 ± 14.20	15	32.97 ± 12.36	15	15.15 ± 7.91	12	27.09 ± 12.47	10	-0.82 [-2.81, 1.17]	-0.05 [-1.9, 1.81]	-0.2 [-3.07, 2.71]						
R 900	R 840	R anterior commissure, olfactory limb	16.92 ± 8.35	15	13.80 ± 4.48	14	17.62 ± 3.17	12	52.60 ± 19.85	9	-0.17 [-2.13, 1.79]	-0.09 [-1.95, 1.81]	1.57 [-1.37, 4.55]						
L 900	L 840	L anterior commissure, olfactory limb	21.04 ± 8.80	15	19.06 ± 8.53	14	22.75 ± 8.82	12	118.79 ± 56.55	9	-0.13 [-2.11, 1.83]	0 [-1.96, 1.91]	3.98 [1.01, 6.97]*	0.08 [-1.72, 1.86]	-0.26 [-2.12, 1.55]	-4.88 [-6.9, -2.86]*	0.36 [-1.57, 2.19]	-4.96 [-7.06, -2.92]*	-4.62 [-6.8, -2.64]*
R 848	R 967	R optic nerve	64.81 ± 30.68	15	80.74 ± 25.95	15	39.40 ± 17.31	12	137.09 ± 48.33	10	-0.6 [-2.57, 1.36]	0.31 [-1.52, 2.13]	0.62 [-2.31, 3.54]						
L 848	L 967	L optic nerve	47.68 ± 26.63	15	21.23 ± 9.93	15	29.74 ± 10.32	12	203.66 ± 96.87	10	-0.59 [-2.58, 1.36]	-0.49 [-2.35, 1.34]	3.54 [0.62, 6.5]*	0.56 [-1.08, 2.35]	0.17 [-1.59, 2.13]	-3.5 [-5.5, -1.56]*	0.38 [-1.58, 2.2]	-4.09 [-6.06, -2.09]*	-3.69 [-5.75, -1.61]*
R 916	R 848	R brachium of the superior colliculus	617.44 ± 558.13	10	652.16 ± 284.02	10	336.43 ± 103.58	10	641.74 ± 297.80	8	-0.44 [-2.63, 1.72]	0.46 [-1.75, 2.64]	-0.36 [-3.55, 2.93]						
L 916	L 848	L brachium of the superior colliculus	66.80 ± 43.16	11	62.85 ± 31.42	9	125.08 ± 82.93	10	154.96 ± 58.14	7	0.8 [-1.39, 2.92]	0.33 [-1.91, 2.55]	-0.13 [-3.49, 3.17]						
R 125	R 848	R optic tract	138.35 ± 66.54	15	131.67 ± 52.32	15	54.57 ± 26.46	12	282.88 ± 116.67	10	-0.81 [-2.76, 1.14]	0.02 [-1.82, 1.9]	1.03 [-1.91, 3.9]						
L 125	L 848	L optic tract	112.59 ± 64.93	15	48.15 ± 29.04	15	61.62 ± 30.12	12	525.56 ± 297.51	10	-0.65 [-2.71, 1.33]	-0.49 [-2.42, 1.4]	4.03 [1.14, 7.03]*	0.57 [-1.15, 2.4]	0.24 [-1.64, 2.16]	-3.91 [-5.88, -1.92]*	0.32 [-1.56, 2.21]	-4.48 [-6.48, -2.56]*	-4.16 [-6.26, -2.12]*
R 832	R 967	R oculomotor nerve	36.05 ± 24.45	10	8.85 ± 5.08	9	10.21 ± 8.43	9	18.24 ± 9.27	7	-0.77 [-3.02, 1.47]	-0.41 [-2.64, 1.83]	0.48 [-2.88, 3.8]						
L 832	L 967	L oculomotor nerve	12.44 ± 11.02	9	8.38 ± 6.47	9	3.78 ± 3.78	9	17.77 ± 15.56	7	-0.74 [-3.03, 1.53]	0.05 [-2.24, 2.33]	0.91 [-2.44, 4.33]						
R 158	R 832	R posterior commissure	72.11 ± 48.91	10	13.61 ± 10.77	7	20.42 ± 16.85	9	35.67 ± 19.29	7	-0.78 [-3.06, 1.41]	-0.33 [-2.69, 2.05]	0.39 [-3.17, 3.83]						
L 158	L 832	L posterior commissure	24.88 ± 22.05	9	4.62 ± 3.10	7	7.57 ± 7.57	9	35.54 ± 31.11	7	-0.75 [-3.07, 1.57]	-0.33 [-2.75, 2.12]	1.31 [-2.15, 4.88]						
R 792	R 967	R dorsal roots	6.76 ± 3.21	10	2.84 ± 1.45	11	20.62 ± 7.65	10	28.57 ± 9.56	8	2.23 [0.07, 4.41]*	-0.24 [-2.36, 1.92]	0.86 [-2.34, 4.02]	0.32 [-1.71, 2.38]	-2.63 [-4.69, -0.54]*	-3.89 [-6.17, -1.68]*	2.95 [0.81, 4.99]*	-4.21 [-6.44, -2.11]*	-1.25 [-3.48, 1.09]
L 792	L 967	L dorsal roots	4.94 ± 2.79	10	1.68 ± 0.83	11	13.44 ± 5.01	10	24.10 ± 7.17	8	1.91 [-0.31, 4.09]	-0.38 [-2.56, 1.78]	1.97 [-1.25, 5.17]						
R 932	R 792	R cervicothalamic tract	10.15 ± 4.81	10	4.27 ± 2.17	11	30.94 ± 11.48	10	42.86 ± 14.34	8	2.23 [0.04, 4.4]*	-0.24 [-2.42, 1.95]	0.86 [-2.39, 4.08]	0.32 [-1.81, 2.32]	-2.64 [-4.7, -0.57]*	-3.9 [-6.09, -1.59]*	2.96 [0.89, 5.05]*	-4.22 [-6.43, -1.99]*	-1.27 [-3.55, 0.98]
L 932	L 792	L cervicothalamic tract	7.42 ± 4.18	10	2.51 ± 1.24	11	20.15 ± 7.52	10	36.15 ± 10.76	8	1.91 [-0.26, 4.09]	-0.37 [-2.48, 1.74]	1.97 [-1.26, 5.16]						
R 697	R 932	R medial lemniscus	20.29 ± 9.63	10	8.53 ± 4.34	11	61.87 ± 22.96	10	85.71 ± 28.68	8	2.23 [0.05, 4.39]*	-0.26 [-2.36, 1.91]	0.86 [-2.33, 4.12]	0.34 [-1.77, 2.3]	-2.64 [-4.81, -0.65]*	-3.89 [-6.19, -1.74]*	2.96 [0.92, 4.95]*	-4.21 [-6.38, -2.01]*	-1.25 [-3.51, 0.91]
L 697	L 932	L medial lemniscus	14.83 ± 8.37	10	5.03 ± 2.48	11	40.31 ± 15.03	10	72.29 ± 21.52	8	1.89 [-0.3, 4.08]	-0.39 [-2.51, 1.78]	1.99 [-1.28, 5.26]						
R 484682512	R 1009	R supra-callosal cerebral white matter	184.43 ± 69.10	15	170.87 ± 80.90	15	319.85 ± 75.99	12	557.55 ± 87.73	10	0.52 [-1.44, 2.5]	0 [-1.88, 1.83]	0.71 [-2.23, 3.62]						
L 484682512	L 1009	L supra-callosal cerebral white matter	27.23 ± 12.53	15	38.47 ± 12.02	15	32.88 ± 9.56	12	129.40 ± 65.28	10	-0.01 [-2.03, 1.97]	0.47 [-1.42, 2.36]	2.48 [-0.47, 5.42]						
R 983	R 1009	R lateral forebrain bundle system	63.46 ± 15.03	15	55.80 ± 13.68	15	141.84 ± 21.70	12	179.96 ± 22.64	10	1.04 [-0.94, 3.07]	-0.03 [-1.94, 1.84]	0.04 [-2.85, 2.98]						
L 983	L 1009	L lateral forebrain bundle system	13.41 ± 5.08	15	12.36 ± 2.96	15	19.98 ± 3.62	12	51.01 ± 12.53	10	0.29 [-1.69, 2.28]	0 [-1.88, 1.86]	1.71 [-1.27, 4.7]						
R 776	R 983	R corpus callosum	106.41 ± 25.89	15	109.07 ± 30.58	15	289.64 ± 47.01	12	365.30 ± 49.34	10	1.52 [-0.44, 3.44]	0.1 [-1.75, 1.95]	0.02 [-2.96, 2.95]						
L 776	L 983	L corpus callosum	21.15 ± 7.45	15	26.97 ± 7.25	15	43.56 ± 7.93	12	98.42 ± 24.90	10	0.84 [-1.14, 2.81]	0.33 [-1.56, 2.18]	1.68 [-1.24, 4.61]						
R 956	R 776	R corpus callosum, anterior forceps	16.65 ± 4.03	15	22.87 ± 6.44	15	34.18 ± 8.74	12	88.50 ± 27.18	10	0.84 [-1.13, 2.82]	0.45 [-1.44, 2.33]	2.23 [-0.73, 5.16]						
L 956	L 776	L corpus callosum, anterior forceps	15.06 ± 6.73	15	14.81 ± 5.89	15	25.98 ± 4.83	12	54.57 ± 16.23	10	0.52 [-1.46, 2.48]	0.05 [-1.78, 1.9]	1.25 [-1.64, 4.17]						
R 579	R 956	R external capsule	33.99 ± 8.40	15	32.65 ± 8.09	15	84.35 ± 19.36	12	200.01 ± 53.34	10	1.29 [-0.68, 3.24]	0.05 [-1.8, 1.88]	2.75 [-0.16, 5.63]						
L 579	L 956	L external capsule	31.70 ± 14.00	15	17.61 ± 3.97	15	58.10 ± 9.37	12	89.02 ± 18.35	10	0.63 [-1.32, 2.56]	-0.37 [-2.23, 1.48]	0.76 [-2.16, 3.66]						
R 964	R 776	R corpus callosum, extreme capsule	31.57 ± 12.45	15	34.44 ± 15.81	15	109.01 ± 37.37	12	135.05 ± 39.98	10	2.25 [0.28, 4.21]*	0.17 [-1.69, 2.05]	0.06 [-2.84, 2.94]	-0.09 [-1.85, 1.66]	-2.64 [-4.45, -0.78]*	-3.51 [-5.49, -1.56]*	2.56 [0.69, 4.37]*	-3.44 [-5.39, -1.49]*	-0.89 [-2.92, 1.2]
L 964	L 776	L corpus callosum, extreme capsule	14.66 ± 9.68	15	15.82 ± 6.94	15	51.23 ± 15.66	12	59.83 ± 16.44	10	2.3 [0.35, 4.24]*	0.16 [-1.71, 2.02]	-0.16 [-3.08, 2.74]	-0.09 [-1.81, 1.68]	-2.7 [-4.5, -0.85]*	-3.34 [-5.28, -1.34]*	2.62 [0.73, 4.42]*	-3.25 [-5.25, -1.36]*	-0.64 [-2.72, 1.45]
R 1108	R 776	R genu of corpus callosum	25.53 ± 11.81	14	35.87 ± 19.28	11	34.70 ± 12.04	11	63.60 ± 20.90	8	0.3 [-1.75, 2.33]	0.58 [-1.43, 2.57]	-0.04 [-3.2, 3.04]						
L 1108	L 776	L genu of corpus callosum	18.60 ± 11.16	14	27.92 ± 10.22	10	30.33 ± 13.05	10	92.22 ± 47.55	9	0.55 [-1.52, 2.63]	0.76 [-1.31, 2.81]	1.89 [-1.24, 4.99]						
R 971	R 776	R corpus callosum, posterior forceps	705.24 ± 147.11	11	522.06 ± 188.66	12	1441.98 ± 443.04	10	1858.05 ± 399.63	9	0.99 [-1.14, 3.06]	0.04 [-2.01, 2.08]	0.07 [-3.05, 3.2]						
L 971	L 776	L corpus callosum, posterior forceps	87.24 ± 36.94	12	70.89 ± 24.71	10	138.38 ± 35.45	10	372.80 ± 125.50	8	0.47 [-1.68, 2.55]	0.18 [-1.97, 2.31]	2.13 [-1.05, 5.31]						
R 484682516	R 776	R corpus callosum, body	189.01 ± 49.52	15	195.44 ± 54.88	13	570.95 ± 115.50	12	802.53 ± 201.16	10	1.8 [-0.13, 3.74]	0.13 [-1.77, 2.04]	0.52 [-2.41, 3.46]						
L 484682516	L 776	L corpus callosum, body	46.99 ± 16.65	15	68.39 ± 31.10	13	80.13 ± 22.82	12	304.41 ± 125.95	10	0.49 [-1.52, 2.43]	0.56 [-1.37, 2.51]	3.62 [0.65, 6.58]*	-0.48 [-2.36, 1.24]	-0.91 [-2.72, 0.95]	-5.71 [-7.61, -3.67]*	0.41 [-1.47, 2.33]	-5.24 [-7.27, -3.2]*	-4.81 [-6.9, -2.82]*
R 986	R 776	R corpus callosum, splenium	644.85 ± 189.46	13	513.17 ± 173.47	15	1340.09 ± 249.82	12	1382.18 ± 338.32	9	0.97 [-1, 2.96]	-0.03 [-1.92, 1.88]	-0.5 [-3.54, 2.4]						
L 986	L 776	L corpus callosum, splenium	86.26 ± 33.95	13	94.78 ± 35.24	15	207.78 ± 49.78	12	356.71 ± 118.77	10	1.3 [-0.7, 3.33]	0.27 [-1.7, 2.21]	0.86 [-2.04, 3.79]						
R 784	R 983	R corticospinal tract	41.70 ± 19.71	15	13.93 ± 4.73	15	22.53 ± 7.83	12	58.88 ± 16.61	10	-0.67 [-2.63, 1.32]	-0.61 [-2.51, 1.26]	0.88 [-2.04, 3.81]						
L 784	L 983	L corticospinal tract	11.90 ± 6.49	15	5.10 ± 1.65	15	6.88 ± 1.87	12	36.20 ± 12.74	10	-0.63 [-2.59, 1.26]	-0.49 [-2.35, 1.38]	2.36 [-0.51, 5.25]						
R 6	R 784	R internal capsule	77.42 ± 37.50	15	16.14 ± 5.50	14	23.52 ± 4.81	12	84.33 ± 21.52	10	-0.9 [-2.87, 1.05]	-0.68 [-2.56, 1.22]	0.88 [-2.1, 3.83]						
L 6	L 784	L internal capsule	20.14 ± 9.44	15	9.56 ± 3.17	15	14.12 ± 3.55	12	76.21 ± 33.40	10	-0.51 [-2.5, 1.43]	-0.44 [-2.34, 1.44]	2.95 [0.04, 5.91]*	0.52 [-1.32, 2.22]	0.1 [-1.78, 1.99]	-3.05 [-5.02, -1.06]*	0.41 [-1.5, 2.26]	-3.56 [-5.56, -1.65]*	-3.15 [-5.22, -1.04]*
R 924	R 784	R cerebal peduncle	107.17 ± 101.58	7	69.17 ± 31.55	9	173.06 ± 83.99	8	286.44 ± 132.42	7	0.51 [-1.97, 2.96]	-0.24 [-2.67, 2.26]	0.88 [-2.77, 4.47]						
L 924	L 784	L cerebal peduncle	60.91 ± 52.75	9	29.21 ± 24.12	9	11.75 ± 7.14	9	66.50 ± 15.26	7	-0.6 [-2.83, 1.68]	-0.13 [-2.34, 2.15]	0.62 [-2.73, 4.02]						

R 896	R 983	R thalamus related	152.34 ± 50.45	15	92.01 ± 20.61	15	212.24 ± 41.23	12	238.98 ± 44.53	10	0.18 [-1.76, 2.12]	-0.33 [-2.21, 1.55]	-0.08 [-3.02, 2.81]						
L 896	L 983	L thalamus related	25.07 ± 11.40	15	13.88 ± 3.67	15	17.98 ± 3.75	12	55.80 ± 16.35	10	-0.48 [-2.44, 1.45]	-0.36 [-2.22, 1.51]	1.28 [-1.61, 4.22]						
R 1092	R 896	R external medullary lamina of the thalamus	125.34 ± 102.16	15	76.67 ± 37.57	14	58.85 ± 15.71	12	72.31 ± 23.55	10	-0.74 [-2.71, 1.21]	-0.3 [-2.22, 1.6]	-0.17 [-3.12, 2.8]						
L 1092	L 896	L external medullary lamina of the thalamus	28.89 ± 14.01	15	21.00 ± 15.18	13	26.46 ± 5.41	12	55.76 ± 14.99	10	-0.28 [-2.22, 1.68]	-0.15 [-2.06, 1.76]	0.57 [-2.34, 3.52]						
R 484682520	R 896	R optic radiation	357.52 ± 111.50	15	208.58 ± 51.65	15	523.66 ± 101.82	12	609.47 ± 112.86	9	0.26 [-1.71, 2.2]	-0.35 [-2.23, 1.56]	0 [-3.05, 2.95]						
L 484682520	L 896	L optic radiation	58.17 ± 27.36	14	31.72 ± 8.64	15	36.20 ± 9.82	12	128.42 ± 40.85	10	-0.52 [-2.54, 1.44]	-0.31 [-2.19, 1.57]	1.3 [-1.65, 4.24]						
R 484682524	R 896	R auditory radiation	131.47 ± 79.58	14	109.44 ± 66.44	15	123.15 ± 55.00	12	174.76 ± 45.51	8	-0.21 [-2.2, 1.76]	-0.03 [-1.91, 1.9]	-0.08 [-3.1, 2.99]						
L 484682524	L 896	L auditory radiation	44.18 ± 23.30	14	11.58 ± 5.39	15	37.57 ± 8.32	12	66.87 ± 16.29	10	-0.29 [-2.27, 1.72]	-0.6 [-2.52, 1.24]	0.68 [-2.26, 3.63]						
R 1000	R 1009	R extrapyramidal fiber systems	5.06 ± 2.92	15	9.62 ± 4.37	15	26.24 ± 5.83	12	25.07 ± 7.52	10	3.98 [2, 5.92]*	0.97 [-0.92, 2.82]	-1.78 [-4.7, 1.12]	-0.9 [-2.7, 0.82]	-4.38 [-6.29, -2.6]*	-4.2 [-6.14, -2.24]*	3.48 [1.69, 5.44]*	-3.31 [-5.36, -1.4]*	0.2 [-1.83, 2.25]
L 1000	L 1009	L extrapyramidal fiber systems	7.66 ± 4.21	15	8.01 ± 2.78	15	26.57 ± 6.63	12	28.58 ± 8.46	10	2.26 [0.26, 4.21]*	0.12 [-1.76, 2.01]	-0.45 [-3.4, 2.51]	-0.04 [-1.77, 1.71]	-2.67 [-4.5, -0.77]*	-2.96 [-4.89, -0.96]*	2.63 [0.61, 4.34]*	-2.93 [-4.82, -0.88]*	-0.3 [-2.32, 1.84]
R 760	R 1000	R cerebral nuclei related	7.60 ± 4.38	15	14.72 ± 6.82	14	39.36 ± 8.75	12	38.60 ± 11.06	10	3.99 [1.98, 5.91]*	1.04 [-0.84, 2.94]	-1.74 [-4.64, 1.16]	-0.97 [-2.76, 0.77]	-4.39 [-6.27, -2.55]*	-4.31 [-6.26, -2.31]*	3.41 [1.55, 5.29]*	-3.37 [-5.37, -1.41]*	0.06 [-2.01, 2.07]
L 760	L 1000	L cerebral nuclei related	11.49 ± 6.32	15	14.17 ± 4.95	15	39.86 ± 9.95	12	43.81 ± 12.49	10	2.27 [0.21, 4.26]*	0.31 [-1.59, 2.16]	-0.56 [-3.47, 2.35]	-0.23 [-2.01, 1.55]	-2.69 [-4.59, -0.77]*	-3.08 [-5.01, -1.11]*	2.44 [0.62, 4.3]*	-2.83 [-4.76, -0.91]*	-0.39 [-2.5, 1.7]
R 102	R 760	R nigrostriatal tract	15.19 ± 8.77	15	29.43 ± 13.65	14	78.72 ± 17.50	12	77.21 ± 22.12	10	3.98 [2.02, 5.91]*	1.02 [-0.87, 2.87]	-1.71 [-4.63, 1.2]	-0.94 [-2.6, 0.9]	-4.38 [-6.18, -2.54]*	-4.33 [-6.34, -2.37]*	3.45 [1.56, 5.26]*	-3.39 [-5.41, -1.41]*	0.06 [-2.03, 2.06]
L 102	L 760	L nigrostriatal tract	22.97 ± 12.64	15	28.33 ± 9.91	15	79.72 ± 19.90	12	87.62 ± 24.98	10	2.27 [0.33, 4.22]*	0.31 [-1.52, 2.15]	-0.56 [-3.49, 2.29]	-0.23 [-1.98, 1.48]	-2.67 [-4.59, -0.88]*	-3.04 [-5.01, -1.11]*	2.45 [0.56, 4.3]*	-2.83 [-4.79, -0.89]*	-0.39 [-2.47, 1.59]
R 991	R 1009	R medial forebrain bundle system	62.75 ± 17.51	15	66.32 ± 12.53	15	287.98 ± 55.23	12	330.40 ± 70.81	10	3.39 [1.4, 5.36]*	0.13 [-1.74, 2]	-0.04 [-2.99, 2.91]	-0.05 [-1.77, 1.68]	-3.79 [-5.61, -1.9]*	-4.51 [-6.43, -2.48]*	3.75 [1.95, 5.63]*	-4.46 [-6.46, -2.55]*	-0.71 [-2.82, 1.32]
L 991	L 1009	L medial forebrain bundle system	15.44 ± 4.66	15	19.01 ± 5.58	15	46.67 ± 11.15	12	78.22 ± 14.20	10	1.81 [-0.13, 3.77]	0.3 [-1.56, 2.18]	1.15 [-1.77, 4]						
R 768	R 991	R cerebrum related	92.93 ± 25.51	15	99.39 ± 19.63	15	434.75 ± 83.75	12	496.60 ± 108.00	10	3.46 [1.47, 5.4]*	0.14 [-1.72, 2.03]	-0.05 [-3, 2.87]	-0.06 [-1.78, 1.7]	-3.86 [-5.69, -1.99]*	-4.6 [-6.54, -2.62]*	3.8 [1.99, 5.65]*	-4.53 [-6.37, -2.49]*	-0.72 [-2.89, 1.29]
L 768	L 991	L cerebrum related	21.26 ± 6.17	15	27.71 ± 8.87	15	67.15 ± 16.18	12	114.04 ± 20.53	10	1.95 [-0.05, 3.9]	0.39 [-1.52, 2.28]	1.23 [-1.7, 4.12]						
R 884	R 768	R amygdalar capsule	32.40 ± 17.76	15	25.53 ± 10.91	15	60.16 ± 14.57	12	104.35 ± 18.80	10	0.65 [-1.28, 2.56]	-0.14 [-2.01, 1.71]	0.92 [-1.98, 3.86]						
L 884	L 768	L amygdalar capsule	22.97 ± 16.16	15	17.95 ± 7.37	15	50.42 ± 8.88	12	52.42 ± 14.74	10	0.99 [-0.99, 2.95]	-0.14 [-2.01, 1.73]	-0.37 [-3.36, 2.5]						
R 940	R 768	R cingulum bundle	141.13 ± 37.11	15	180.21 ± 86.67	15	273.44 ± 61.72	12	453.27 ± 71.35	10	0.75 [-1.2, 2.66]	0.36 [-1.47, 2.21]	0.31 [-2.63, 3.27]						
L 940	L 768	L cingulum bundle	44.69 ± 17.01	15	32.57 ± 11.61	15	89.45 ± 35.80	12	192.93 ± 73.04	10	0.78 [-1.2, 2.79]	-0.21 [-2.1, 1.65]	1.95 [-0.98, 4.91]						
R 1099	R 768	R fornix system	152.91 ± 47.98	15	160.32 ± 29.76	15	757.87 ± 147.25	12	848.15 ± 188.38	10	3.75 [1.82, 5.72]*	0.13 [-1.71, 1.99]	-0.13 [-3.01, 2.78]	-0.06 [-1.84, 1.64]	-4.16 [-6.03, -2.33]*	-4.8 [-6.73, -2.8]*	4.11 [2.27, 5.99]*	-4.75 [-6.61, -2.7]*	-0.65 [-2.76, 1.29]
L 1099	L 768	L fornix system	29.15 ± 8.47	15	44.57 ± 15.10	15	108.91 ± 26.25	12	178.56 ± 33.94	10	2.53 [0.55, 4.49]*	0.6 [-1.3, 2.48]	1.19 [-1.75, 4.13]	-0.52 [-2.27, 1.23]	-2.93 [-4.79, -1.11]*	-5.36 [-7.35, -3.4]*	2.41 [0.5, 4.25]*	-4.84 [-6.81, -2.88]*	-2.43 [-4.47, -0.29]*
R 466	R 1099	R alveus	326.61 ± 95.12	15	424.47 ± 62.45	15	1571.15 ± 216.91	12	1842.55 ± 369.51	10	3.62 [1.63, 5.54]*	0.38 [-1.49, 2.25]	-0.16 [-3.03, 2.81]	-0.31 [-2.03, 1.42]	-4.04 [-5.83, -2.19]*	-4.88 [-6.85, -2.91]*	3.7 [1.89, 5.54]*	-4.58 [-6.56, -2.59]*	-0.86 [-2.93, 1.16]
L 466	L 1099	L alveus	42.32 ± 7.57	15	99.47 ± 27.97	15	225.63 ± 41.40	12	391.64 ± 71.87	10	4.11 [2.1, 6.07]*	1.42 [-0.46, 3.33]	1.93 [-0.98, 4.89]	-1.33 [-3.08, 0.43]	-4.51 [-6.33, -2.58]*	-8.51 [-10.45, -6.56]*	3.18 [1.28, 5.02]*	-7.15 [-8.99, -5.11]*	-4 [-6.1, -1.96]*
R 603	R 1099	R fimbria	356.68 ± 142.39	15	363.15 ± 89.01	15	2032.74 ± 475.59	12	2165.30 ± 620.41	10	4.5 [2.53, 6.44]*	0.09 [-1.78, 1.98]	-0.32 [-3.21, 2.59]	-0.02 [-1.77, 1.72]	-4.91 [-6.71, -3.05]*	-5.31 [-7.22, -3.29]*	4.89 [2.99, 6.64]*	-5.29 [-7.16, -3.31]*	-0.41 [-2.4, 1.69]
L 603	L 1099	L fimbria	73.25 ± 26.47	15	99.33 ± 59.27	15	228.80 ± 70.79	12	357.27 ± 95.99	10	1.89 [-0.01, 3.82]	0.42 [-1.43, 2.3]	0.76 [-2.19, 3.68]						
R 737	R 1099	R postcommissural fornix	24.29 ± 11.52	15	18.10 ± 5.48	14	143.57 ± 51.11	12	118.40 ± 26.93	10	4.68 [2.7, 6.59]*	-0.19 [-2.1, 1.7]	-1.42 [-4.35, 1.47]	0.27 [-1.48, 2.06]	-5.08 [-6.88, -3.21]*	-4.11 [-6.11, -2.13]*	5.35 [3.53, 7.34]*	-4.38 [-6.41, -2.4]*	0.98 [-1.17, 2.99]
L 737	L 1099	L postcommissural fornix	12.99 ± 7.05	15	14.58 ± 5.18	14	32.46 ± 6.00	12	42.60 ± 8.47	10	1.31 [-0.71, 3.27]	0.22 [-1.67, 2.09]	-0.03 [-2.95, 2.89]						
R 436	R 737	R columns of the fornix	48.58 ± 23.04	15	36.19 ± 10.95	14	287.14 ± 102.22	12	236.79 ± 53.87	10	4.71 [2.74, 6.61]*	-0.17 [-2, 1.77]	-1.46 [-4.37, 1.51]	0.25 [-1.55, 1.98]	-5.12 [-6.89, -3.15]*	-4.12 [-6.2, -2.27]*	5.35 [3.41, 7.12]*	-4.36 [-6.44, -2.46]*	0.99 [-1.07, 3.02]
L 436	L 737	L columns of the fornix	25.98 ± 14.09	15	29.16 ± 10.37	14	64.92 ± 11.99	12	86.63 ± 16.91	10	1.29 [-0.65, 3.23]	0.21 [-1.69, 2.1]	0.04 [-2.9, 2.94]						
R 618	R 1099	R hippocampal commissures	182.91 ± 42.44	15	162.04 ± 51.79	15	502.90 ± 123.86	12	830.63 ± 103.58	10	1.53 [-0.45, 3.52]	-0.05 [-1.96, 1.84]	1.26 [-1.64, 4.16]						
L 618	L 1099	L hippocampal commissures	46.11 ± 16.09	15	59.70 ± 15.35	15	195.81 ± 48.76	12	339.58 ± 111.27	10	3.04 [1.12, 4.97]*	0.37 [-1.46, 2.21]	2.15 [-0.75, 5.08]	-0.3 [-2.08, 1.41]	-3.45 [-5.24, -1.56]*	-6.6 [-8.53, -4.6]*	3.15 [1.37, 5.02]*	-6.31 [-8.16, -4.27]*	-3.16 [-5.16, -1.08]*
R 443	R 618	R dorsal hippocampal commissure	359.91 ± 85.84	15	325.07 ± 103.37	15	1009.13 ± 246.88	12	1661.26 ± 207.17	10	1.59 [-0.41, 3.55]	-0.03 [-1.93, 1.83]	1.27 [-1.64, 4.17]						
L 443	L 618	L dorsal hippocampal commissure	92.15 ± 32.21	15	118.47 ± 30.95	15	391.90 ± 97.45	12	679.15 ± 222.55	10	3.06 [1.09, 5.03]*	0.37 [-1.48, 2.22]	2.16 [-0.73, 5.09]	-0.28 [-2.07, 1.41]	-3.46 [-5.27, -1.58]*	-6.62 [-8.59, -4.78]*	3.16 [1.32, 5.01]*	-6.34 [-8.33, -4.42]*	-3.18 [-5.13, -1.09]*
R 301	R 768	R stria terminalis	68.75 ± 21.35	15	50.88 ± 20.47	15	284.35 ± 162.65	12	300.84 ± 130.15	10	2.92 [0.95, 4.84]*	-0.19 [-2.07, 1.7]	-0.17 [-3.13, 2.71]	0.28 [-1.52, 1.99]	-3.33 [-5.21, -1.53]*	-3.61 [-5.64, -1.66]*	3.6 [1.67, 5.39]*	-3.86 [-5.8, -1.85]*	-0.28 [-2.33, 1.81]
L 301	L 768	L stria terminalis	107.60 ± 58.08	15	21.81 ± 14.12	15	61.58 ± 29.67	12	120.46 ± 36.42	10	-0.64 [-2.62, 1.36]	-0.74 [-2.63, 1.16]	0.69 [-2.24, 3.58]						
R 484682528	R 301	R commissural branch of stria terminalis	11.08 ± 8.12	14	37.22 ± 21.98	12	59.80 ± 20.95	12	76.82 ± 36.39	10	4.26 [2.3, 6.21]*	2.52 [0.53, 4.5]*	-1.58 [-4.56, 1.41]	-2.45 [-4.29, -0.55]*	-4.66 [-6.55, -2.81]*	-6.24 [-8.26, -4.3]*	2.21 [0.2, 4.13]*	-3.8 [-5.84, -1.65]*	-1.59 [-3.56, 0.56]
L 484682528	L 301	L commissural branch of stria terminalis	219.71 ± 202.36	14	5.67 ± 4.01	13	67.28 ± 28.84	12	106.33 ± 39.64	9	-0.85 [-2.85, 1.18]	-0.81 [-2.8, 1.15]	0.41 [-2.66, 3.42]						
R 824	R 991	R hypothalamus related	33.22 ± 15.61	15	27.79 ± 6.72	15	43.15 ± 11.09	12	82.10 ± 28.26	10	0.09 [-1.88, 2.02]	-0.09 [-2, 1.77]	0.68 [-2.32, 3.63]						
L 824	L 991	L hypothalamus related	19.37 ± 8.94	15	14.95 ± 3.21	14	31.66 ± 7.10	12	50.29 ± 18.98	10	0.4 [-1.59, 2.4]	-0.21 [-2.13, 1.67]	0.61 [-2.35, 3.52]						
R 54	R 824	R medial forebrain bundle	160.47 ± 81.63	8	43.51 ± 20.58	10	235.56 ± 124.46	9	573.40 ± 276.38	7	0.36 [-1.93, 2.7]	-0.39 [-2.67, 1.89]	2.29 [-1.04, 5.65]						
L 54	L 824	L medial forebrain bundle	87.09 ± 59.44	9	107.97 ± 69.07	10	144.28 ± 43.15	8	84.58 ± 25.11	8	0.74 [-1.6, 2.98]	0.54 [-1.7, 2.79]	-1.68 [-5.01, 1.69]						
R 349	R 824	R supraoptic commissures	99.82 ± 47.49	14	84.73 ± 63.76	12	28.64 ± 17.10	12	51.04 ± 21.70	10	-0.84 [-2.84, 1.15]	0.01 [-1.98, 1.99]	-0.39 [-3.37, 2.63]						
L 349	L 824	L supraoptic commissures	50.45 ± 36.05	14	28.14 ± 15.77	12	33.28 ± 22.92	12	8.01 ± 5.34	10	-0.49 [-2.47, 1.48]	-0.29 [-2.21, 1.68]	-0.8 [-3.81, 2.15]						
R 46	R 824	R mammillary related	20.62 ± 8.13	14	63.65 ± 19.68	15	33.87 ± 11.53	12	52.08 ± 11.87	10	0.51 [-1.46, 2.48]	2.24 [0.33, 4.13]*	-1.95 [-4.89, 0.96]	-2.17 [-3.96, -0.41]*	-0.92 [-2.74, 1.05]	-1.84 [-3.91, 0.06]	-1.25 [-3.13, 0.57]	0.32 [-1.63, 2.33]	-0.93 [-3.07, 1.02]
L 46	L 824	L mammillary related	18.49 ± 10.50	14	15.97 ± 4.82	15	41.00 ± 9.79	12	46.52 ± 15.87	10	1.08 [-0.87, 3.05]	0.01 [-1.88, 1.85]	-0.31 [-3.24, 2.54]						
R 753	R 46	R principal mammillary tract	166.83 ± 88.13	9	580.09 ± 166.03	9	164.50 ± 96.77	9	246.45 ± 83.95	7	0.16 [-2.14, 2.46]	2.83 [0.55, 5.13]*	-2.79 [-6.16, 0.65]	-2.75 [-4.94, -0.56]*	-0.55 [-2.79, 1.63]	-1.23 [-3.55, 1.15]	-2.2 [-4.41, 0.03]	1.52 [-0.81, 3.91]	-0.67 [-2.98, 1.75]
L 753	L 46	L principal mammillary tract	74.67 ± 36.86	10	171.35 ± 65.76	9	258.27 ± 94.48	9	278.72 ± 136.47	8	2.63 [0.4, 4.85]*	1.67 [-0.55, 3.89]	-1.95 [-5.23, 1.34]	-1.59 [-3.7, 0.51]	-3.05 [-5.27, -1.02]*	-3.39 [-5.68, -1.19]*	1.44 [-0.7, 3.65]	-1.79 [-4.1, 0.46]	-0.34 [-2.71, 1.8]
R 690	R 46	R mammillothalamic tract	18.60 ± 7.41	14	55.72 ± 36.17	14	45.87 ± 13.52	12	77.29 ± 19.33	10	1.33 [-0.64, 3.27]	2.15 [0.26, 4.04]*	-1.06 [-3.99, 1.85]	-2.06 [-3.9, -0.34]*	-1.74 [-3.59, 0.11]	-3.47 [-5.42, -1.46]*	-0.35 [-2.25, 1.49]	-1.39 [-3.33, 0.6]	-1.74 [-3.74, 0.35]
L 690	L 46	L mammillothalamic tract	31.97 ± 21.49	14	7.91 ± 3.26	14	45.85 ± 13.98	12	62.31 ± 14.13	10	0.3 [-1.71, 2.28]	-0.59 [-2.5, 1.32]	0.51 [-2.43, 3.51]						
R 681	R 46	R mammillotegmental tract	58.53 ± 34.35	9	23.20 ± 19.90	10	142.86 ± 95.98	8	32.43 ± 14.68	6	1.57 [-0.76, 3.9]	-0.32 [-2.58, 1.93]	-1.76 [-5.29, 1.74]						
L 681	L 46	L mammillotegmental tract	72.63 ± 43.47	9	81.62 ± 38.40	10	37.08 ± 24.78	9	158.73 ± 99.79	7	-0.31 [-2.6, 1.98]	0.43 [-1.84, 2.64]	0.84 [-2.56, 4.17]						
R 1083	R 824	R epithalamus related	59.71 ± 32.08	15	27.21 ± 10.86	15	70.30 ± 22.40	12	120.70 ± 50.88	10	-0.03 [-1.98, 1.88]	-0.46 [-2.35, 1.46]	0.73 [-2.14, 3.62]						
L 1083	L 824	L epithalamus related	30.32 ± 14.68	15	16.47 ± 4.22	15	44.81 ± 15.84	12	83.95 ± 39.89	10	0.28 [-1.67, 2.2]	-0.38 [-2.22, 1.45]	1.08 [-1.83, 4.05]						
R 802	R 1083	R stria medullaris	167.95 ± 105.45	15	83.99 ± 49.61	13	222.00 ± 77.80	12	394.23 ± 155.43	10	0.12 [-1.83, 2.08]	-0.37 [-2.27, 1.53]	0.81 [-2.2, 3.74]						
L 802	L 1083	L stria medullaris	90.06 ± 63.96	15	52.67 ± 18.18	13	130.89 ± 53.44	12	272.04 ± 149.28	10	0.24 [-1.74, 2.16]	-0.3 [-2.24, 1.62]	1.28 [-1.71, 4.26]						
R 595	R 1083	R fasciculus retroflexus	48.10 ± 27.68	14	21.16 ± 11.59	15	63.72 ± 27.60	12	34.49 ± 14.44	10	0.21 [-1.81, 2.19]	-0.4 [-2.3, 1.48]	-0.81 [-3.73, 2.14]						
L 595	L 1083	L fasciculus retroflexus	24.38 ± 17.63	14	11.13 ± 3.92	15	29.97 ± 14.24	12	37.38 ± 13.66	10	0.08 [-1.94, 2.08]	-0.41 [-2.3, 1.49]	0.13 [-2.85, 3.03]						
R 611	R 1083	R habenular commissure	103.13 ± 58.96	13	67.05 ± 34.43	15	108.52 ± 30.46	11	241.58 ± 147.82	8	-0.11 [-2.16, 1.93]	-0.19 [-2.14, 1.71]	0.9 [-2.17, 3.97]						
L 611	L 1083	L habenular commissure	61.83 ± 42.92	13	40.92 ± 23.96	15	208.57 ± 126.02	11	91.46 ± 38.69	9	2.25 [0.23, 4.31]*	-0.15 [-2.05, 1.75]	-2.32 [-5.34, 0.69]	0.24 [-1.56, 2.01]	-2.65 [-4.67, -0.77]*	-0.82 [-2.8, 1.29]	2.88 [0.99, 4.75]*	-1.05 [-3.03, 1.01]	1.83 [-0.3, 3.95]

Table S1. **Results from parent and subregion level AT8+ immunoreactive aggregate mapping.** Mean AT8+ immunoreactive aggregates/mm^2 +/- standard error of the mean and sample size for each experimental group (n) for each sampled brain region. Parent ID indicates the hierarchical "parent" area for each region. Results from Bayesian statistical models (genotype, treatment, and brain region as fixed effects, animal as random effect) show the estimated relative change (normalized to regional WT-Sal) and 95% credible intervals (CI) in AT8+ levels due to interactions between brain region and 5XFAD genotype (genotype effect), PTZ treatment (treatment effect), and 5XFAD genotype and PTZ (Interaction). Estimated marginal means and 95% CI were used for group contrasts at each brain region, where credible genotype, treatment, or interaction effects were found. Separate models were performed for the isocortex, olfactory areas, hippocampal formation, cortical subplate, striatum, pallidum, thalamus, hypothalamus, midbrain, and fiber tracts. *=results are considered credible when the 95% CI does not cross 0.

ID	Parent	Region	WT.Sal	n	WT.PTZ	n	5X.Sal	n	5X.PTZ	n	Genotype effect	Treatment effect	Interaction	WT Sal vs WT PTZ	WT Sal vs 5XFAD Sal	WT Sal vs 5XFAD PTZ	5XFAD Sal vs WT PTZ	WT PTZ VS 5XFAD PT	5XFAD Sal vs 5XFAD PTZ
R 315	R 695	R Isocortex	5.04 ± 1.12	15	14.57 ± 3.57	16	6.16 ± 0.89	11	26.77 ± 4.49	12	-0.02 [-2.03, 2.02]	1.72 [-0.16, 3.61]	2.18 [-0.62, 4.9]						
L 315	L 695	L Isocortex	4.95 ± 0.88	15	14.91 ± 3.41	16	6.65 ± 0.97	11	27.62 ± 5.00	12	0.11 [-1.86, 2.15]	1.84 [0.01, 3.69]*	2.19 [-0.62, 4.94]	-1.97 [-3.99, -0.06]*	-0.43 [-2.51, 1.66]	-4.83 [-7.02, -2.74]*	-1.57 [-3.69, 0.6]	-2.84 [-5, -0.67]*	-4.42 [-6.73, -2.14]*
R 500	R 315	R Somatomotor areas	3.71 ± 1.00	15	18.48 ± 4.95	16	6.04 ± 0.87	11	37.68 ± 7.56	12	0.38 [-1.6, 2.37]	3.81 [1.95, 5.66]*	4.51 [1.71, 7.28]*	-3.95 [-5.92, -1.99]*	-0.69 [-2.74, 1.38]	-9.39 [-11.51, -7.23]*	-3.26 [-5.38, -1.08]*	-5.45 [-7.52, -3.25]*	-8.71 [-11.03, -6.44]*
L 500	L 315	L Somatomotor areas	3.69 ± 0.69	15	17.85 ± 4.81	16	6.42 ± 0.95	11	33.58 ± 7.00	12	0.5 [-1.51, 2.52]	3.67 [1.84, 5.54]*	3.49 [0.67, 6.29]*	-3.8 [-5.71, -1.8]*	-0.8 [-2.84, 1.33]	-8.35 [-10.58, -6.28]*	-2.99 [-5.02, -0.7]*	-4.55 [-6.63, -2.43]*	-7.54 [-9.81, -5.19]*
R 985	R 500	R Primary motor area	5.74 ± 1.30	13	28.86 ± 8.51	16	8.09 ± 1.35	11	63.27 ± 11.88	11	0.23 [-1.86, 2.28]	3.93 [2.04, 5.87]*	5.47 [2.66, 8.28]*	-4.07 [-6.02, -2]*	-0.54 [-2.62, 1.67]	-10.33 [-12.52, -8.08]*	-3.52 [-5.6, -1.31]*	-6.25 [-8.41, -4.09]*	-9.77 [-12.14, -7.48]*
L 985	L 500	L Primary motor area	4.95 ± 0.85	15	27.45 ± 7.77	16	7.95 ± 1.28	11	56.97 ± 10.37	11	0.37 [-1.61, 2.41]	4.38 [2.52, 6.3]*	5.31 [2.45, 8.09]*	-4.52 [-6.59, -2.6]*	-0.69 [-2.72, 1.46]	-10.75 [-12.96, -8.55]*	-3.84 [-6, -1.71]*	-6.23 [-8.36, -4.07]*	-10.08 [-12.37, -7.79]*
R 993	R 500	R Secondary motor area	8.40 ± 2.76	11	27.21 ± 6.25	16	11.40 ± 1.65	11	62.35 ± 11.15	11	0.14 [-1.92, 2.28]	2.11 [0.19, 4.07]*	3.73 [0.87, 6.54]*	-2.24 [-4.37, -0.28]*	-0.45 [-2.63, 1.64]	-6.67 [-8.92, -4.37]*	-1.8 [-3.95, 0.38]	-4.44 [-6.69, -2.36]*	-6.22 [-8.51, -3.87]*
L 993	L 500	L Secondary motor area	7.66 ± 1.62	13	27.83 ± 7.77	14	13.26 ± 2.13	11	54.46 ± 11.00	11	0.48 [-1.56, 2.55]	2.64 [0.71, 4.58]*	2.53 [-0.32, 5.38]	-2.78 [-4.81, -0.74]*	-0.79 [-2.97, 1.3]	-6.35 [-8.49, -4.06]*	-1.99 [-4.19, 0.2]	-3.56 [-5.76, -1.33]*	-5.55 [-7.93, -3.23]*
R 453	R 315	R Somatosensory areas	5.73 ± 1.41	15	20.09 ± 5.36	16	7.81 ± 1.09	11	33.35 ± 6.03	12	0.13 [-1.86, 2.19]	2.34 [0.49, 4.19]*	1.91 [-0.88, 4.64]	-2.46 [-4.42, -0.51]*	-0.44 [-2.53, 1.66]	-5.07 [-7.22, -2.85]*	-2.04 [-4.22, 0.1]	-2.6 [-4.75, -0.49]*	-4.63 [-6.87, -2.22]*
L 453	L 315	L Somatosensory areas	5.48 ± 1.07	15	19.32 ± 4.66	16	7.83 ± 1.04	11	34.68 ± 7.16	11	0.19 [-1.84, 2.25]	2.36 [0.48, 4.27]*	2.32 [-0.53, 5.15]	-2.51 [-4.48, -0.52]*	-0.5 [-2.62, 1.62]	-5.56 [-7.78, -3.4]*	-2.01 [-4.15, 0.14]	-3.07 [-5.21, -0.92]*	-5.07 [-7.37, -2.66]*
R 322	R 453	R Primary somatosensory area	8.57 ± 1.99	14	26.65 ± 7.19	16	10.42 ± 1.58	10	44.78 ± 8.12	11	-0.06 [-2.09, 2.07]	1.96 [0.12, 3.84]*	1.88 [-1.01, 4.7]	-2.1 [-4.13, -0.18]*	-0.25 [-2.41, 1.88]	-4.46 [-6.61, -2.23]*	-1.84 [-3.98, 0.4]	-2.36 [-4.54, -0.24]*	-4.21 [-6.6, -1.89]*
L 322	L 453	L Primary somatosensory area	7.20 ± 1.39	15	25.53 ± 6.13	16	10.42 ± 1.37	11	45.48 ± 8.94	11	0.21 [-1.8, 2.24]	2.39 [0.55, 4.26]*	2.27 [-0.54, 5.03]	-2.52 [-4.57, -0.63]*	-0.53 [-2.63, 1.57]	-5.57 [-7.81, -3.48]*	-2.01 [-4.12, 0.15]	-3.04 [-5.27, -0.94]*	-5.06 [-7.3, -2.68]*
R 353	R 322	R Primary somatosensory area, nose	12.22 ± 3.12	10	28.02 ± 11.77	10	11.46 ± 3.12	8	46.69 ± 13.05	11	-0.17 [-2.39, 2.04]	1.02 [-1.08, 3.1]	1.57 [-1.44, 4.6]						
L 353	L 322	L Primary somatosensory area, nose	12.72 ± 2.94	9	15.42 ± 8.08	8	12.32 ± 3.30	9	115.37 ± 76.99	10	0.04 [-2.14, 2.27]	0.7 [-1.51, 2.9]	7.43 [4.31, 10.55]*	-0.85 [-3.08, 1.54]	-0.34 [-2.69, 1.91]	-8.87 [-11.11, -6.45]*	-0.5 [-2.81, 1.95]	-8.02 [-10.38, -5.67]*	-8.51 [-10.89, -6.05]*
R 329	R 322	R Primary somatosensory area, barrel fiel	15.56 ± 3.64	14	45.92 ± 12.98	16	19.13 ± 2.63	11	70.87 ± 13.14	11	-0.01 [-2.05, 2.06]	1.79 [-0.1, 3.71]	1.31 [-1.47, 4.07]						
L 329	L 322	L Primary somatosensory area, barrel fiel	14.96 ± 2.83	14	42.41 ± 9.72	16	18.73 ± 2.21	11	70.64 ± 14.03	11	0 [-2.02, 2.02]	1.67 [-0.17, 3.57]	1.59 [-1.26, 4.38]						
R 337	R 322	R Primary somatosensory area, lower limb	13.30 ± 4.49	13	47.05 ± 18.71	12	14.62 ± 2.37	11	67.35 ± 13.58	11	-0.14 [-2.16, 1.89]	2.63 [0.67, 4.61]*	1.12 [-1.74, 3.97]	-2.76 [-4.79, -0.72]*	-0.18 [-2.34, 1.94]	-4.32 [-6.57, -2.12]*	-2.6 [-4.8, -0.36]*	-1.54 [-3.76, 0.67]	-4.13 [-6.38, -1.76]*
L 337	L 322	L Primary somatosensory area, lower limb	12.63 ± 2.71	13	35.55 ± 11.17	11	16.21 ± 2.84	11	68.79 ± 14.96	11	0.04 [-1.99, 2.14]	1.68 [-0.31, 3.7]	2.27 [-0.67, 5.12]						
R 345	R 322	R Primary somatosensory area, mouth	7.18 ± 1.45	13	33.31 ± 9.18	14	11.11 ± 1.94	10	66.08 ± 11.69	11	0.27 [-1.77, 2.36]	3.24 [1.3, 5.22]*	4.25 [1.4, 7.08]*	-3.38 [-5.4, -1.33]*	-0.58 [-2.71, 1.57]	-8.46 [-10.73, -6.36]*	-2.8 [-4.99, -0.58]*	-5.07 [-7.28, -2.93]*	-7.86 [-10.16, -5.5]*
L 345	L 322	L Primary somatosensory area, mouth	6.53 ± 1.21	13	36.43 ± 11.13	13	9.95 ± 1.64	11	71.73 ± 14.97	10	0.3 [-1.75, 2.41]	3.99 [2.1, 5.95]*	5.2 [2.29, 8.02]*	-4.11 [-6.24, -2.16]*	-0.61 [-2.7, 1.51]	-10.18 [-12.33, -7.89]*	-3.54 [-5.71, -1.27]*	-6.04 [-8.29, -3.86]*	-9.57 [-12.01, -7.29]*
R 369	R 322	R Primary somatosensory area, upper limb	9.66 ± 2.71	14	37.98 ± 12.85	14	13.75 ± 3.06	11	65.58 ± 12.62	11	0.18 [-1.81, 2.21]	2.92 [1.04, 4.89]*	2.22 [-0.63, 5.07]	-3.05 [-5.18, -1.13]*	-0.49 [-2.61, 1.61]	-6.01 [-8.22, -3.75]*	-2.56 [-4.76, -0.42]*	-2.95 [-5.25, -0.86]*	-5.52 [-7.85, -3.2]*
L 369	L 322	L Primary somatosensory area, upper limb	8.48 ± 2.17	14	35.45 ± 12.31	14	13.05 ± 2.34	11	62.32 ± 12.52	11	0.3 [-1.73, 2.34]	3.19 [1.31, 5.14]*	2.41 [-0.47, 5.17]	-3.33 [-5.28, -1.3]*	-0.61 [-2.72, 1.51]	-6.59 [-8.81, -4.41]*	-2.72 [-4.84, -0.49]*	-3.27 [-5.45, -1.05]*	-5.98 [-8.4, -3.72]*
R 361	R 322	R Primary somatosensory area, trunk	14.14 ± 3.24	14	45.63 ± 15.40	13	17.15 ± 2.49	11	71.97 ± 14.77	11	-0.03 [-2.04, 2.02]	2.04 [0.12, 4.01]*	1.63 [-1.28, 4.44]	-2.16 [-4.23, -0.19]*	-0.26 [-2.46, 1.74]	-4.33 [-6.57, -2.15]*	-1.9 [-4.07, 0.34]	-2.17 [-4.48, -0.05]*	-4.05 [-6.33, -1.66]*
L 361	L 322	L Primary somatosensory area, trunk	10.41 ± 2.03	13	41.51 ± 12.37	14	18.44 ± 3.96	11	72.25 ± 15.06	11	0.53 [-1.53, 2.6]	2.99 [1.05, 4.97]*	1.96 [-0.93, 4.81]	-3.12 [-5.14, -1.04]*	-0.86 [-2.88, 1.34]	-6.17 [-8.47, -4.08]*	-2.28 [-4.41, -0.04]*	-3.05 [-5.29, -0.94]*	-5.34 [-7.75, -3.11]*
R 182305	R 322	R Primary somatosensory area, unassigne	14.46 ± 4.12	14	45.79 ± 22.24	12	17.82 ± 1.97	11	67.34 ± 15.21	11	0 [-2.05, 2.06]	2.26 [0.29, 4.23]*	0.94 [-1.96, 3.76]	-2.41 [-4.46, -0.36]*	-0.3 [-2.29, 1.92]	-3.88 [-6.08, -1.7]*	-2.09 [-4.23, 0.17]	-1.5 [-3.68, 0.7]	-3.58 [-5.8, -1.14]*
L 182305	L 322	L Primary somatosensory area, unassigne	14.27 ± 4.33	14	39.66 ± 14.25	12	18.93 ± 2.60	11	66.71 ± 16.11	11	0.09 [-1.95, 2.16]	1.54 [-0.43, 3.52]	1.59 [-1.32, 4.48]						
R 378	R 453	R Supplemental somatosensory area	9.47 ± 2.42	12	32.43 ± 8.57	16	12.91 ± 1.91	10	59.62 ± 10.69	12	0.07 [-1.98, 2.21]	2.28 [0.39, 4.25]*	2.53 [-0.4, 5.31]	-2.41 [-4.46, -0.37]*	-0.38 [-2.54, 1.79]	-5.57 [-7.77, -3.35]*	-2.04 [-4.25, 0.15]	-3.15 [-5.3, -0.99]*	-5.2 [-7.46, -2.76]*
L 378	L 453	L Supplemental somatosensory area	9.73 ± 1.87	14	32.44 ± 8.20	16	12.62 ± 1.78	11	67.50 ± 13.81	10	0.06 [-1.98, 2.16]	2.17 [0.33, 4.05]*	2.83 [-0.02, 5.62]	-2.31 [-4.3, -0.33]*	-0.36 [-2.56, 1.72]	-5.75 [-8.06, -3.61]*	-1.95 [-4.12, 0.26]	-3.44 [-5.68, -1.27]*	-5.39 [-7.76, -3.03]*
R 1057	R 315	R Gustatory areas	7.49 ± 2.30	15	24.01 ± 5.81	14	5.31 ± 1.04	11	53.26 ± 9.53	12	-0.52 [-2.5, 1.47]	1.8 [-0.04, 3.69]	4.41 [1.56, 7.15]*	-1.92 [-3.88, 0.07]	0.22 [-1.86, 2.26]	-6.37 [-8.53, -4.21]*	-2.15 [-4.29, 0.07]	-4.45 [-6.59, -2.32]*	-6.59 [-8.85, -4.23]*
L 1057	L 315	L Gustatory areas	7.49 ± 1.86	15	29.04 ± 8.91	14	4.95 ± 1.10	11	61.45 ± 12.76	12	-0.58 [-2.58, 1.44]	2.51 [0.65, 4.4]*	4.83 [2.04, 7.64]*	-2.64 [-4.69, -0.71]*	0.27 [-1.81, 2.41]	-7.46 [-9.66, -5.32]*	-2.93 [-5.12, -0.76]*	-4.81 [-6.91, -2.62]*	-7.73 [-10.02, -5.44]*
R 677	R 315	R Visceral area	4.79 ± 0.99	14	20.61 ± 6.73	14	6.58 ± 1.11	10	38.16 ± 9.44	12	0.06 [-1.98, 2.13]	3.29 [1.39, 5.26]*	3.17 [0.31, 6.03]*	-3.43 [-5.52, -1.46]*	-0.36 [-2.59, 1.68]	-7.2 [-9.39, -5.02]*	-3.07 [-5.3, -0.83]*	-3.77 [-6.03, -1.7]*	-6.86 [-9.11, -4.39]*
L 677	L 315	L Visceral area	5.66 ± 1.40	14	25.20 ± 7.86	14	6.18 ± 1.26	11	54.27 ± 10.63	10	-0.15 [-2.16, 1.93]	3.45 [1.55, 5.37]*	4.41 [1.52, 7.24]*	-3.59 [-5.68, -1.64]*	-0.16 [-2.3, 1.88]	-8.41 [-10.61, -6.19]*	-3.43 [-5.57, -1.21]*	-4.82 [-7.07, -2.67]*	-8.24 [-10.6, -5.91]*
R 247	R 315	R Auditory areas	9.40 ± 1.98	14	20.44 ± 5.19	16	11.01 ± 1.91	11	39.70 ± 7.94	11	0.01 [-1.98, 2.05]	1.09 [-0.77, 3.02]	1.71 [-1.1, 4.46]						
L 247	L 315	L Auditory areas	8.55 ± 1.86	14	22.16 ± 5.06	15	12.46 ± 2.16	11	49.35 ± 11.63	12	0.3 [-1.76, 2.35]	1.38 [-0.53, 3.31]	2.74 [-0.11, 5.54]						
R 1011	R 247	R Dorsal auditory area	16.71 ± 4.34	12	32.89 ± 9.09	16	17.00 ± 3.10	11	62.03 ± 13.74	10	-0.15 [-2.2, 1.93]	0.89 [-1.04, 2.85]	1.91 [-1.02, 4.79]						
L 1011	L 247	L Dorsal auditory area	11.87 ± 3.46	12	38.01 ± 9.52	15	17.05 ± 2.61	11	77.59 ± 17.50	12	0.27 [-1.81, 2.38]	1.97 [0.04, 3.94]*	2.93 [0.01, 5.73]*	-2.11 [-4.21, -0.15]*	-0.58 [-2.76, 1.51]	-5.86 [-8.16, -3.69]*	-1.53 [-3.8, 0.6]	-3.75 [-5.87, -1.54]*	-5.27 [-7.59, -2.95]*
R 1002	R 247	R Primary auditory area	16.72 ± 2.80	13	35.50 ± 9.00	12	18.33 ± 3.79	11	56.58 ± 20.14	9	-0.06 [-2.1, 2.03]	1 [-0.93, 3.01]	1.44 [-1.49, 4.31]						
L 1002	L 247	L Primary auditory area	14.94 ± 3.26	13	36.62 ± 7.95	14	22.03 ± 4.04	11	82.65 ± 22.99	11	0.34 [-1.7, 2.42]	1.07 [-0.85, 3.03]	2.68 [-0.17, 5.51]						
R 1027	R 247	R Posterior auditory area	14.02 ± 4.30	6	30.85 ± 16.99	7	13.43 ± 4.18	10	95.27 ± 21.89	6	-0.33 [-2.67, 2.08]	0.27 [-2.15, 2.68]	3.92 [0.52, 7.28]*	-0.42 [-2.87, 2.18]	0.01 [-2.48, 2.39]	-4.55 [-7.27, -1.9]*	-0.43 [-2.88, 1.88]	-4.15 [-6.91, -1.7]*	-4.56 [-7.16, -2.06]*
L 1027	L 247	L Posterior auditory area	18.86 ± 4.26	7	39.66 ± 16.56	7	17.83 ± 7.22	8	86.33 ± 28.91	7	-0.05 [-2.37, 2.32]	-1.16 [-3.49, 1.26]	3.67 [0.27, 6.99]*	1.03 [-1.52, 3.4]	-0.27 [-2.56, 2.31]	-3.14 [-5.67, -0.59]*	1.27 [-1.06, 3.86]	-4.19 [-6.68, -1.62]*	-2.89 [-5.44, -0.3]*
R 1018	R 247	R Ventral auditory area	11.28 ± 3.13	14	28.60 ± 7.11	16	15.39 ± 2.52	11	50.72 ± 13.45	10	0.2 [-1.87, 2.22]	1.44 [-0.45, 3.36]	1.79 [-0.98, 4.64]						
L 1018	L 247	L Ventral auditory area	11.47 ± 2.65	14	32.13 ± 7.77	15	16.43 ± 3.09	11	63.53 ± 15.22	12	0.26 [-1.73, 2.36]	1.56 [-0.32, 3.48]	2.33 [-0.48, 5.07]						
R 669	R 315	R Visual areas	15.86 ± 3.60	14	19.17 ± 5.72	16	13.57 ± 3.11	11	44.94 ± 8.20	11	-0.32 [-2.36, 1.74]	0.11 [-1.76, 1.98]	1.63 [-1.27, 4.44]						
L 669	L 315	L Visual areas	15.15 ± 3.23	14	23.35 ± 6.16	15	17.20 ± 3.77	11	51.66 ± 10.33	10	-0.04 [-2.06, 2]	0.3 [-1.57, 2.23]	1.59 [-1.32, 4.4]						
R 402	R 669	R Anterolateral visual area	23.40 ± 8.47	7	37.45 ± 13.13	8	23.00 ± 7.31	9	87.20 ± 17.94	6	-0.2 [-2.51, 2.13]	0 [-2.29, 2.33]	1.16 [-2.2, 4.45]						
L 402	L 669	L Anterolateral visual area	24.28 ± 6.23	8	43.41 ± 12.87	8	20.47 ± 9.24	8	103.15 ± 21.45	8	-0.15 [-2.42, 2.14]	-0.89 [-3.15, 1.37]	3.47 [0.24, 6.65]*	0.75 [-1.63, 3.12]	-0.16 [-2.57, 2.19]	-3.14 [-5.56, -0.66]*	0.9 [-1.51, 3.39]	-3.87 [-6.29, -1.49]*	-2.99 [-5.42, -0.36]*
R 394	R 669	R Anteromedial visual area	16.65 ± 5.36	12	30.98 ± 8.55	15	17.60 ± 2.26	11	65.10 ± 10.90	11	-0.09 [-2.15, 1.99]	0.69 [-1.23, 2.6]	1.91 [-0.96, 4.73]						
L 394	L 669	L Anteromedial visual area	15.82 ± 3.33	13	41.29 ± 10.53	14	24.33 ± 5.35	11	64.18 ± 13.72	10	0.39 [-1.64, 2.49]	1.6 [-0.3, 3.55]	0.53 [-2.39, 3.37]						
R 385	R 669	R Primary visual area	34.16 ± 8.77	11	47.47 ± 14.41	10	25.30 ± 8.76	9	88.51 ± 17.72	7	-0.16 [-2.35, 2.06]	-0.67 [-2.75, 1.43]	1.01 [-2.14, 4.16]						
L 385	L 669	L Primary visual area	41.30 ± 7.94	11	48.38 ± 14.51	11	34.11 ± 9.21	9	87.50 ± 19.41	9	-0.26 [-2.38, 1.89]	-0.55 [-2.56, 1.48]	1.62 [-1.37, 4.56]						
R 533	R 669	R posteromedial visual area	21.31 ± 6.70	10	47.17 ± 14.83	8	15.39 ± 4.15	10	60.61 ± 11.41	7	-0.24 [-2.39, 1.93]	-0.51 [-2.67, 1.69]	1.2 [-1.97, 4.33]						
L 533	L 669	L posteromedial visual area	16.93 ± 3.76	10	36.99 ± 11.74	10	22.56 ± 6.05	10	62.11 ± 13.03	9	0.36 [-1.77, 2.53]	0.14 [-1.93, 2.27]	1.86 [-1.18, 4.85]						
R 31	R 315	R Anterior cingulate area	9.34 ± 2.83	15	20.34 ± 3.87	16	11.85 ± 2.07	11	41.89 ± 5.69	12	0.04 [-1.96, 2.07]	1.02 [-0.82, 2.9]	1.99 [-0.77, 4.76]						
L 31	L 315	L Anterior cingulate area	8.45 ± 1.34	15	20.36 ± 3.87	15	12.22 ± 2.01	11	38.11 ± 5.63	12	0.2 [-1.75, 2.2]	1.55 [-0.3, 3.42]	1.32 [-1.47, 4.07]						
R 39	R 31	R Anterior cingulate area, dorsal part	15.65 ± 4.83	13	32.65 ± 7.35	15	19.02 ± 3.47	11	68.64 ± 10.38	12	0.04 [-1.99, 2.11]	1.08 [-0.82, 3.03]	1.88 [-0.99, 4.69]						
L 39	L 31	L Anterior cingulate area, dorsal part	13.99 ± 2.40	15	34.05 ± 7.14	15	18.60 ± 3.57	11	58.46 ± 8.97	12	0.09 [-1.92, 2.09]	1.57 [-0.33, 3.48]	1.07 [-1.72, 3.84]						
R 48	R 31	R Anterior cingulate area, ventral part	14.80 ± 4.61	15	30.02 ± 5.04	15	17.81 ± 3.18	11	61.48 ± 8.25	12	-0.02 [-2.01, 2.02]	0.82 [-1.02, 2.72]	1.92 [-0.89, 4.69]						
L 48	L 31	L Anterior cingulate area, ventral part	11.41 ± 2.17	15	28.69 ± 4.83	14	19.41 ± 3.04	11	59.06 ± 8.76	12	0.46 [-1.55, 2.5]	1.62 [-0.26, 3.58]	1.65 [-1.19, 4.43]						
R 972	R 315	R Prelimbic area	6.52 ± 2.51	10	33.54 ± 11.10	10	23.03 ± 8.75	6	49.99 ± 12.92	8	2.34 [0, 4.7]	3.64 [1.56, 5.75]*	-0.39 [-3.6, 2.78]	-3.77 [-5.91, -1.51]*	-2.65 [-5.1, -0.25]*	-6.29 [-8.66, -3.9]*	-1.12 [-3.57, 1.29]	-2.51 [-4.92, -0.21]*	-3.63 [-6.18, -0.94]*
L 972	L 315	L Prelimbic area	15.87 ± 2.35	9	34.74 ± 14.44	8	15.72 ± 8.68	6	48.82 ± 17.46	7	-0.35 [-2.72, 2.02]	0.26 [-2, 2.51]	0.36 [-2.94, 3.69]						
R 95	R 315	R Agranular insular area	4.11 ± 0.80	14	11.83 ± 3.38	16	4.27 ± 0.61	10	23.24 ± 3.91	12	-0.2 [-2.27, 1.89]	1.78 [-0.1, 3.73]	2.7 [-0.15, 5.51]						
L 95	L 315	L Agranular insular area	3.64 ± 0.52	15	12.75 ± 3.56	16	4.46 ± 0.81	11	26.90 ± 4.31	11	-0.01 [-2, 1.98]	2.34 [0.51, 4.19]*	3.23 [0.41, 5.98]*	-2.47 [-4.42, -0.49]*	-0.29 [-2.41, 1.73]	-6.25 [-8.51, -4.11]*	-2.18 [-4.29, 0.01]	-3.77 [-5.84, -1.49]*	-5.96 [-8.24, -3.57]*
R 104	R 95	R Agranular insular area, dorsal part	6.94 ± 1.59	11	20.72 ± 5.96	15	6.04 ± 0.97	10	38.58 ± 7.27	12	-0.38 [-2.48, 1.74]	1.69 [-0.26, 3.68]	2.86 [-0.03, 5.72]						
L 104	L 95	L Agranular insular area, dorsal part	5.99 ± 0.62	14	19.66 ± 5.90	16	6.27 ± 1.28	11	43.61 ± 7.90	10	-0.13 [-2.14, 1.95]	2.19 [0.32, 4.11]*	3.36 [0.45, 6.17]*	-2.31 [-4.26, -0.31]*	-0.17 [-2.3, 1.86]	-6.1 [-8.42, -3.92]*	-2.16 [-4.24, 0.05]	-3.79 [-5.97, -1.64]*	-5.94 [-8.16, -3.43]*
R 111	R 95	R Agranular insular area, posterior part	5.02 ± 1.12	13	13.42 ± 4.28	13	5.43 ± 1.20	10	30.83 ± 5.64	12	-0.14 [-2.2, 1.94]	1.68 [-0.27, 3.64]	3.25 [0.36, 6.09]*	-1.81 [-3.86, 0.24]	-0.17 [-2.27, 2.04]	-5.48 [-7.72, -3.33]*	-1.65 [-3.81, 0.68]	-3.66 [-5.89, -1.56]*	-5.31 [-7.66, -2.97]*
L 111	L 95	L Agranular insular area, posterior part	3.64 ± 0.65	15	13.43 ± 3.77	14	5.34 ± 1.00	11	35.13 ± 5.96	11	0.24 [-1.71, 2.3]	2.7 [0.83, 4.62]*	4.88 [2, 7.69]*	-2.82 [-4.86, -0.92]*	-0.54 [-2.65, 1.5]	-8.51 [-10.5, -6.15]*	-2.28 [-4.56, -0.11]*	-5.67 [-7.72, -3.42]*	-7.97 [-10.26, -5.58]*
R 119	R 95	R Agranular insular area, ventral part	9.07 ± 1.89	14	22.11 ± 5.87	15	7.12 ± 1.78	10	53.62 ± 14.97	12	-0.46 [-2.48, 1.58]	1.17 [-0.71, 3.06]	3.84 [1.02, 6.62]*	-1.3 [-3.27, 0.7]	0.15 [-2.08, 2.2]	-5.24 [-7.36, -3.04]*	-1.45 [-3.63, 0.81]	-3.95 [-6.04, -1.71]*	-5.38 [-7.62, -2.91]*
L 119	L 95	L Agranular insular area, ventral part	9.33 ± 1.29	14	21.88 ± 6.38	16	9.48 ± 2.44	11	49.42 ± 7.51	10	-0.15 [-2.19, 1.9]	1.26 [-0.59, 3.15]	2.32 [-0.59, 5.12]						
R 254	R 315	R Retrosplenial area	8.07 ± 2.09	14	18.72 ± 4.35	16	8.69 ± 1.53	11	33.62 ± 6.24	11	-0.16 [-2.17, 1.87]	1.15 [-0.69, 3.06]	2.03 [-0.78, 4.81]						
L 254	L 315	L Retrosplenial area	8.94 ± 1.54	14	18.96 ± 4.67	16	10.39 ± 2.21	11	31.38 ± 6.32	12	0 [-2.04, 2.04]	1.02 [-0.87, 2.88]	1.12 [-1.67, 3.93]						
R 894	R 254	R Retrosplenial area, lateral agranular part	14.96 ± 3.04	14	34.34 ± 9.33	16	16.52 ± 2.28	11	61.34 ± 10.97	11	-0.1 [-2.11, 1.97]	1.18 [-0.69, 3.09]	1.94 [-0.93, 4.68]						
L 894	L 254	L Retrosplenial area, lateral agranular part	15.69 ± 2.53	13	36.33 ± 8.78	16	17.56 ± 2.99	11	58.68 ± 10.51	12	-0.01 [-2.05, 2.1]	1.26 [-0.56, 3.18]	1.15 [-1.64, 3.95]						
R 879	R 254	R Retrosplenial area, dorsal part	13.43 ± 3.44	15	33.68 ± 8.98	16	17.39 ± 3.20	10	69.85 ± 11.82	11	-0.04 [-2.04, 2.08]	1.34 [-0.5, 3.22]	2.18 [-0.67, 4.99]						
L 879	L 254	L Retrosplenial area, dorsal part	14.89 ± 2.66	14	34.03 ± 8.70	16	20.55 ± 4.50	11	62.34 ± 14.08	11	0.22 [-1.81, 2.25]	1.2 [-0.67, 3.11]	1.13 [-1.74, 3.9]						
R 886	R 254	R Retrosplenial area, ventral part	11.35 ± 2.91	15	23.47 ± 5.05	16	10.53 ± 2.29	11	40.57 ± 7.16	12	-0.33 [-2.35, 1.7]	0.89 [-0.94, 2.74]	1.57 [-1.25, 4.28]						
L 886	L 254	L Retrosplenial area, ventral part	12.76 ± 2.38	14	22.66 ± 5.51	16	12.31 ± 3.00	11	36.93 ± 7.36	12	-0.22 [-2.29, 1.81]	0.67 [-1.19, 2.61]	1.06 [-1.75, 3.9]						
R 22	R 315	R Posterior parietal association areas	11.53 ± 2.78	15	27.38 ± 7.29	16	14.09 ± 2.28	11	48.52 ± 7.76	11	-0.02 [-2.01, 2.04]	1.21 [-0.64, 3.08]	1.54 [-1.22, 4.27]						
L 22	L 315	L Posterior parietal association areas	13.49 ± 2.82	14	30.90 ± 7.12	16	18.35 ± 3.07	11	49.98 ± 9.58	12	0.18 [-1.84, 2.22]	1.19 [-0.67, 3.08]	0.96 [-1.89, 3.72]						
R 312782	R 22	R Anterior area	14.76 ± 3.79	15	39.69 ± 11.04	16	17.06 ± 3.32	11	65.65 ± 11.15	11	-0.09 [-2.04, 1.95]	1.52 [-0.33, 3.4]	1.54 [-1.31, 4.31]						
L 312782	L 22	L Anterior area	17.74 ± 4.47	15	47.39 ± 11.73	16	24.59 ± 5.71	11	70.61 ± 15.47	11	0.14 [-1.83, 2.19]	1.51 [-0.31, 3.41]	0.87 [-1.97, 3.66]						
R 417	R 22	R Rostrolateral visual area	21.82 ± 5.89	14	47.23 ± 11.78	16	24.51 ± 5.11	11	85.05 ± 13.90	11	-0.05 [-2.09, 1.99]	1.06 [-0.79, 2.93]	1.47 [-1.38, 4.22]						
L 417	L 22	L Rostrolateral visual area	20.64 ± 5.79	14	47.69 ± 9.88	15	33.77 ± 6.07	11	98.49 ± 16.90	10	0.48 [-1.55, 2.53]	1.09 [-0.77, 3.02]	1.65 [-1.22, 4.42]						
R 541	R 315	R Temporal association areas	8.46 ± 2.76	13	20.62 ± 6.01	13	8.58 ± 1.88	11	44.76 ± 12.66	9	-0.15 [-2.17, 1.97]	1.45 [-0.49, 3.46]	2.94 [-0.02, 5.88]						
L 541	L 315	L Temporal association areas	8.36 ± 1.77	13	20.77 ± 5.81	16	12.16 ± 2.09	11	45.41 ± 10.25	12	0.29 [-1.74, 2.37]	1.39 [-0.52, 3.33]	2.38 [-0.45, 5.19]						
R 922	R 315	R Perirhinal area	3.17 ± 1.00	14	8.07 ± 2.25	14	4.22 ± 1.46	11	26.00 ± 4.61	9	0.16 [-1.86, 2.21]	1.7 [-0.16, 3.64]	4.43 [1.54, 7.29]*	-1.82 [-3.93, 0.1]	-0.47 [-2.64, 1.54]	-6.97 [-9.28, -4.78]*	-1.35 [-3.49, 0.9]	-5.14 [-7.43, -2.91]*	-6.5 [-8.99, -4.25]*
L 922	L 315	L Perirhinal area	1.61 ± 0.68	14	8.17 ± 2.23	16	3.21 ± 1.16	11	16.66 ± 6.16	12	0.82 [-1.16, 2.9]	3.98 [2.12, 5.86]*	4.19 [1.41, 6.94]*	-4.11 [-6.15, -2.19]*	-1.12 [-3.2, 1.04]	-9.68 [-11.88, -7.51]*	-3 [-5.08, -0.77]*	-5.56 [-7.68, -3.41]*	-8.55 [-10.92, -6.31]*
R 895	R 315	R Ectorhinal area	7.15 ± 1.98	14	15.48 ± 4.02	14	6.91 ± 2.11	11	36.78 ± 8.29	9	-0.2 [-2.25, 1.87]	1.31 [-0.61, 3.23]	2.1 [-0.79, 4.97]						
L 895	L 315	L Ectorhinal area	6.68 ± 1.54	14	17.96 ± 5.05	16	7.18 ± 1.77	11	30.29 ± 6.83	12	-0.1 [-2.08, 1.96]	1.59 [-0.25, 3.51]	1.67 [-1.17, 4.41]						
R 698	R 695	R Olfactory areas	7.98 ± 2.04	14	17.33 ± 5.03	15	7.27 ± 1.29	12	38.37 ± 7.38	12	-0.42 [-2.48, 1.64]	0.9 [-1.05, 2.87]	2.68 [-0.28, 5.62]						
L 698	L 695	L Olfactory areas	7.85 ± 1.38	14	14.38 ± 3.93	15	7.86 ± 1.39	12	33.13 ± 5.98	12	-0.33 [-2.44, 1.77]	0.57 [-1.45, 2.62]	2.34 [-0.66, 5.27]						
L 159	L 698	L Anterior olfactory nucleus	14.53 ± 3.78	8	21.33 ± 6.60	7	13.43 ± 4.80	6	132.21 ± 58.22	6	-0.34 [-2.83, 2.22]	0.04 [-2.4, 2.44]	6.93 [3.31, 10.59]*	-0.25 [-2.63, 1.96]	0 [-2.33, 2.48]	-7.43 [-9.82, -5.06]*	-0.25 [-2.63, 2.26]	-7.19 [-9.63, -4.8]*	-7.43 [-9.97, -4.91]*
R 589	R 698	R Taenia tecta	8.64 ± 1.99	12	24.39 ± 9.70	10	7.23 ± 2.12	8	25.80 ± 4.75	10	-0.53 [-2.83, 1.71]	1.46 [-0.73, 3.64]	0.41 [-2.82, 3.65]						
L 589	L 698	L Taenia tecta	7.57 ± 1.68	12	17.36 ± 6.61	10	6.25 ± 0.92	9	23.18 ± 3.90	10	-0.56 [-2.75, 1.63]	0.93 [-1.21, 3.1]	1.06 [-2.07, 4.21]						
R 597	R 589	R Taenia tecta, dorsal part	17.39 ± 3.87	12	54.08 ± 20.90	9	14.53 ± 4.23	8	51.25 ± 9.82	10	-0.53 [-2.76, 1.73]	1.62 [-0.56, 3.82]	0.23 [-2.96, 3.43]						
L 597	L 589	L Taenia tecta, dorsal part	15.30 ± 3.43	12	39.39 ± 13.96	9	11.96 ± 1.85	9	46.45 ± 8.09	10	-0.6 [-2.78, 1.61]	1.08 [-1.1, 3.27]	0.92 [-2.24, 4.09]						
R 814	R 698	R Dorsal peduncular area	18.58 ± 6.56	14	49.87 ± 17.89	9	20.14 ± 4.32	8	64.39 ± 11.32	10	-0.38 [-2.59, 1.8]	1.15 [-0.99, 3.31]	1.01 [-2.2, 4.21]						
L 814	L 698	L Dorsal peduncular area	16.60 ± 4.15	13	50.30 ± 17.35	9	27.19 ± 9.69	8	69.07 ± 10.31	10	0.21 [-2.06, 2.44]	1.53 [-0.64, 3.75]	0.8 [-2.5, 4.07]						
R 961	R 698	R Piriform area	21.15 ± 5.31	10	65.41 ± 18.77	10	21.92 ± 3.36	8	94.77 ± 17.95	12	-0.39 [-2.66, 1.92]	1.28 [-0.94, 3.53]	2.03 [-1.11, 5.25]						
L 961	L 698	L Piriform area	20.18 ± 2.77	12	32.56 ± 12.33	11	22.35 ± 3.81	9	95.32 ± 15.51	11	-0.12 [-2.32, 2.1]	1.02 [-1.1, 3.17]	2.06 [-1.07, 5.27]						
R 619	R 698	R Nucleus of the lateral olfactory tract	15.24 ± 5.00	13	28.54 ± 11.13	6	12.29 ± 3.44	10	30.33 ± 8.99	6	-0.41 [-2.58, 1.72]	-0.19 [-2.5, 2.22]	1.02 [-2.45, 4.5]						
L 619	L 698	L Nucleus of the lateral olfactory tract	15.47 ± 6.13	10	40.34 ± 22.24	7	23.31 ± 8.47	8	41.71 ± 12.07	6	0.21 [-2.14, 2.55]	0.74 [-1.63, 3.16]	-1.37 [-4.85, 2.2]						
R 260	R 619	R Nucleus of the lateral olfactory tract, mo	51.39 ± 23.28	13	44.57 ± 23.43	6	20.90 ± 7.25	9	41.71 ± 21.73	6	-0.83 [-2.99, 1.35]	-1.18 [-3.58, 1.18]	1.26 [-2.28, 4.67]						
L 260	L 619	L Nucleus of the lateral olfactory tract, mo	36.09 ± 14.87	10	31.02 ± 14.72	6	28.21 ± 10.92	8	43.82 ± 21.87	6	-0.54 [-2.86, 1.79]	-1.42 [-3.86, 1.01]	0.05 [-3.51, 3.61]						
R 268	R 619	R Nucleus of the lateral olfactory tract, py	27.31 ± 10.44	13	78.51 ± 32.74	6	25.07 ± 8.84	10	73.88 ± 24.95	6	-0.3 [-2.43, 1.83]	0.81 [-1.56, 3.17]	0.62 [-2.73, 4.07]						
L 268	L 619	L Nucleus of the lateral olfactory tract, py	34.58 ± 14.31	10	110.60 ± 62.91	7	61.90 ± 21.29	8	104.47 ± 25.15	6	0.49 [-1.84, 2.83]	1.33 [-1.03, 3.65]	-1.92 [-5.43, 1.62]						
R 1139	R 619	R Nucleus of the lateral olfactory tract, lay	19.95 ± 6.97	13	40.97 ± 20.19	6	19.55 ± 7.76	10	57.88 ± 24.48	6	-0.23 [-2.38, 1.89]	0.01 [-2.33, 2.37]	1.55 [-1.86, 5.01]						
L 1139	L 619	L Nucleus of the lateral olfactory tract, lay	23.78 ± 11.81	10	114.79 ± 88.86	7	43.27 ± 18.66	8	206.06 ± 173.41	6	0.51 [-1.78, 2.85]	2.95 [0.58, 5.35]*	2.07 [-1.42, 5.58]	-3.17 [-5.31, -0.96]*	-0.84 [-3.01, 1.31]	-6.36 [-8.77, -4.11]*	-2.31 [-4.55, 0.04]	-3.18 [-5.49, -0.67]*	-5.51 [-7.91, -3.03]*
R 631	R 698	R Cortical amygdalar area	11.47 ± 2.67	14	15.75 ± 3.61	11	8.81 ± 1.51	10	37.68 ± 11.97	12	-0.47 [-2.58, 1.64]	-0.22 [-2.27, 1.85]	2.36 [-0.64, 5.34]						
L 631	L 698	L Cortical amygdalar area	11.12 ± 2.75	14	20.61 ± 6.10	13	8.48 ± 2.27	10	26.91 ± 5.97	12	-0.39 [-2.51, 1.73]	0.5 [-1.5, 2.5]	0.73 [-2.28, 3.74]						
R 639	R 631	R Cortical amygdalar area, anterior part	49.04 ± 10.48	15	75.23 ± 15.14	10	48.66 ± 8.13	10	112.09 ± 23.51	12	-0.27 [-2.35, 1.86]	0.01 [-2.08, 2.12]	0.9 [-2.12, 3.97]						
L 639	L 631	L Cortical amygdalar area, anterior part	48.15 ± 10.58	14	91.56 ± 17.99	12	40.61 ± 9.81	10	89.02 ± 15.67	12	-0.3 [-2.43, 1.81]	0.7 [-1.33, 2.75]	-0.14 [-3.17, 2.92]						
R 647	R 631	R Cortical amygdalar area, posterior part	7.52 ± 2.84	13	11.28 ± 3.23	12	4.93 ± 1.70	10	37.48 ± 11.79	9	-0.51 [-2.69, 1.67]	0.55 [-1.49, 2.63]	3.44 [0.33, 6.49]*	-0.75 [-2.59, 1.14]	0.17 [-1.79, 2.25]	-4.29 [-6.34, -2.3]*	-0.93 [-2.96, 1.09]	-3.53 [-5.53, -1.48]*	-4.45 [-6.6, -2.24]*
L 647	L 631	L Cortical amygdalar area, posterior part	5.78 ± 1.43	13	14.89 ± 3.43	13	4.45 ± 1.18	9	29.35 ± 7.48	11	-0.26 [-2.43, 1.89]	1.71 [-0.33, 3.73]	2.26 [-0.71, 5.33]						
R 655	R 647	R Cortical amygdalar area, posterior part,	15.03 ± 5.08	13	19.09 ± 5.67	12	10.28 ± 4.09	10	74.75 ± 21.75	9	-0.47 [-2.6, 1.73]	0.33 [-1.72, 2.42]	3.62 [0.51, 6.79]*	-0.54 [-2.41, 1.37]	0.13 [-1.8, 2.1]	-4.27 [-6.25, -2.21]*	-0.67 [-2.64, 1.36]	-3.75 [-5.76, -1.7]*	-4.43 [-6.6, -2.3]*
L 655	L 647	L Cortical amygdalar area, posterior part,	10.20 ± 3.12	13	25.85 ± 8.75	13	10.61 ± 3.31	9	58.92 ± 14.25	11	0 [-2.21, 2.22]	1.66 [-0.38, 3.71]	2.76 [-0.32, 5.88]						
R 663	R 647	R Cortical amygdalar area, posterior part,	17.98 ± 8.08	11	26.92 ± 9.35	12	10.40 ± 3.13	9	65.03 ± 27.72	8	-0.51 [-2.78, 1.68]	0.57 [-1.57, 2.71]	2.48 [-0.73, 5.69]						
L 663	L 647	L Cortical amygdalar area, posterior part,	13.45 ± 4.01	11	32.59 ± 8.45	13	7.02 ± 1.80	9	53.96 ± 14.78	9	-0.5 [-2.77, 1.71]	1.56 [-0.54, 3.62]	1.72 [-1.41, 4.93]						
R 788	R 698	R Piriform-amygdalar area	13.50 ± 2.82	15	35.75 ± 12.88	11	15.95 ± 2.13	10	90.37 ± 15.91	11	-0.09 [-2.17, 2.05]	0.94 [-1.13, 3.02]	3.97 [0.9, 7.07]*	-1.17 [-2.98, 0.73]	-0.24 [-2.2, 1.68]	-5.64 [-7.49, -3.68]*	-0.91 [-2.9, 1.13]	-4.49 [-6.48, -2.51]*	-5.39 [-7.49, -3.32]*
L 788	L 698	L Piriform-amygdalar area	12.94 ± 2.71	15	38.14 ± 15.13	15	16.46 ± 4.25	11	78.13 ± 12.67	11	-0.07 [-2.21, 1.99]	1.7 [-0.3, 3.69]	2.53 [-0.43, 5.52]						
R 1089	R 695	R Hippocampal formation	1.21 ± 0.27	15	7.66 ± 2.32	16	1.81 ± 0.39	12	13.40 ± 3.20	12	-0.78 [-7.77, 6.27]	-0.68 [-7.44, 6.23]	-0.98 [-9.23, 7.08]						
L 1089	L 695	L Hippocampal formation	0.98 ± 0.17	15	7.42 ± 2.28	16	1.38 ± 0.25	12	11.56 ± 2.31	12	-0.89 [-7.78, 6.07]	-0.03 [-6.69, 6.68]	-0.95 [-9.08, 7.04]						
R 1080	R 1089	R Hippocampal region	1.53 ± 0.34	15	10.13 ± 3.15	16	2.27 ± 0.48	12	17.46 ± 4.33	12	-0.8 [-7.64, 6.12]	-0.51 [-7.19, 6.26]	-0.91 [-8.87, 7.13]						
L 1080	L 1089	L Hippocampal region	1.23 ± 0.20	15	10.25 ± 3.16	16	1.80 ± 0.33	12	15.48 ± 3.19	12	-0.9 [-8.01, 6.23]	0.34 [-6.63, 7.17]	-0.85 [-8.96, 7.34]						
R 375	R 1080	R Ammon's horn	1.67 ± 0.39	15	7.60 ± 2.04	16	2.43 ± 0.45	12	15.87 ± 4.25	12	-0.75 [-7.59, 6.13]	-1.56 [-8.35, 5.25]	-1.17 [-9.27, 6.9]						
L 375	L 1080	L Ammon's horn	1.28 ± 0.25	15	8.00 ± 2.30	16	2.23 ± 0.41	12	11.54 ± 2.26	12	-1.07 [-8.15, 5.95]	-1.05 [-7.91, 5.84]	-1.71 [-9.78, 6.33]						
R 382	R 375	R Field CA1	2.50 ± 0.55	15	10.49 ± 2.91	16	3.79 ± 0.83	12	31.40 ± 10.41	12	-0.12 [-7.09, 6.8]	-1.23 [-7.98, 5.54]	-0.17 [-8.15, 7.88]						
L 382	L 375	L Field CA1	1.69 ± 0.43	15	11.19 ± 3.70	16	3.82 ± 1.02	12	24.27 ± 4.65	12	0.01 [-7.02, 7.07]	-0.19 [-7.06, 6.6]	-0.11 [-8.1, 7.86]						
R 423	R 375	R Field CA2	1.17 ± 0.59	15	11.48 ± 4.05	16	2.39 ± 1.18	12	25.80 ± 6.74	12	0.66 [-6.26, 7.59]	2.18 [-4.48, 8.86]	2.01 [-5.97, 9.86]						
L 423	L 375	L Field CA2	2.06 ± 0.68	15	15.77 ± 5.94	16	2.98 ± 1.21	12	20.51 ± 6.74	11	-1.03 [-7.95, 5.89]	-0.14 [-6.74, 6.4]	-1.24 [-9.36, 6.94]						
R 463	R 375	R Field CA3	4.80 ± 1.33	14	21.51 ± 6.03	16	6.21 ± 1.04	12	35.27 ± 9.84	12	-1.13 [-8.1, 5.92]	-1.89 [-8.74, 4.9]	-1.87 [-10.08, 6.3]						
L 463	L 375	L Field CA3	3.57 ± 0.76	15	21.43 ± 6.24	16	5.17 ± 0.74	12	22.80 ± 5.27	11	-1.31 [-8.24, 5.52]	-1.28 [-8.07, 5.47]	-2.11 [-10.26, 5.89]						
R 726	R 1080	R Dentate gyrus	4.04 ± 0.87	15	36.43 ± 12.86	16	6.23 ± 1.55	12	55.24 ± 12.89	12	-0.75 [-7.6, 6.23]	0.82 [-5.94, 7.49]	-0.68 [-8.71, 7.43]						
L 726	L 1080	L Dentate gyrus	3.51 ± 0.60	15	36.54 ± 12.74	16	4.13 ± 0.90	11	56.09 ± 12.35	12	-0.75 [-7.96, 6.39]	1.71 [-5.06, 8.53]	-0.05 [-8.31, 8.03]						
R 10703	R 726	R Dentate gyrus, molecular layer	5.73 ± 1.19	15	42.80 ± 13.80	16	9.46 ± 2.88	12	66.77 ± 14.13	12	-0.83 [-7.74, 6.08]	-0.14 [-6.88, 6.55]	-1.05 [-9.22, 7.12]						
L 10703	L 726	L Dentate gyrus, molecular layer	5.54 ± 1.14	15	40.57 ± 14.01	16	5.66 ± 1.54	11	78.01 ± 19.84	12	-0.59 [-7.54, 6.37]	0.28 [-6.53, 7.07]	-0.09 [-8.07, 7.83]						
R 10704	R 726	R Dentate gyrus, polymorph layer	8.36 ± 2.79	15	120.11 ± 50.37	16	23.96 ± 9.26	12	157.82 ± 43.21	12	13.67 [-22.98, 50.16]	05.86 [71.57, 140.05]	10.1 [-41.49, 62.26]	-13.77 [-26.42, -0.71]*	-1.91 [-16.12, 11.84]	-19.22 [-33.62, -5.54]*	-11.92 [-25.82, 1.87]	-5.44 [-19.06, 8.48]	-17.37 [-31.96, -1.95]*
L 10704	L 726	L Dentate gyrus, polymorph layer	7.13 ± 2.96	15	145.22 ± 50.71	16	7.85 ± 2.64	11	115.20 ± 29.64	12	-0.91 [-38.11, 36.06]	32.29 [98.53, 166.68]	-42.94 [-95.53, 9.68]	-19.62 [-32.65, -6.47]*	-0.05 [-13.77, 15.14]	-16.49 [-30.24, -2.19]*	-19.59 [-34.26, -5.71]*	3.09 [-10.98, 16.95]	-16.46 [-31.75, -1.27]*
R 632	R 726	R Dentate gyrus, granule cell layer	13.80 ± 3.95	15	131.62 ± 48.34	16	16.46 ± 3.96	12	193.94 ± 48.17	12	0.71 [-35.42, 36.8]	11.84 [77.61, 146.09]	47.89 [-3.95, 98.47]	-9 [-21.46, 4.29]	-0.27 [-14.53, 13.33]	-14.57 [-28.22, -0.14]*	-8.71 [-22.5, 5.08]	-5.54 [-18.99, 8.4]	-14.26 [-29.05, 0.7]
L 632	L 726	L Dentate gyrus, granule cell layer	10.85 ± 2.02	15	131.25 ± 48.03	16	14.92 ± 3.23	11	198.30 ± 45.79	12	2.2 [-34.91, 39.45]	114.4 [79.59, 148.99]	51.11 [-1.02, 103.75]	-11.71 [-24.74, 1.54]	-0.31 [-15.14, 13.3]	-18.68 [-32.63, -4.29]*	-11.14 [-25.5, 3.05]	-6.99 [-20.86, 6.46]	-18.22 [-33.77, -3.1]*
R 982	R 1080	R Fasciola cinerea	7.75 ± 4.41	13	27.78 ± 10.36	16	7.65 ± 5.56	12	28.14 ± 12.89	10	-1.53 [-8.59, 5.6]	-2.57 [-9.38, 4.13]	-2.49 [-10.58, 5.55]						
L 982	L 1080	L Fasciola cinerea	8.20 ± 6.37	13	15.61 ± 6.04	16	6.97 ± 3.79	12	22.34 ± 9.92	11	-1.44 [-8.4, 5.55]	-3.32 [-10.11, 3.46]	-2.58 [-10.8, 5.6]						
R 19	R 1080	R Induseum griseum	0.46 ± 0.46	15	14.41 ± 8.49	13	7.99 ± 4.24	12	50.33 ± 11.89	12	16.79 [9.67, 23.9]*	21.25 [14.29, 28.23]*	24.63 [16.5, 32.65]*	-27.11 [-34.99, -19.38]*	-18.54 [-26.82, -10.35]*	-75.66 [-86.51, -64.81]*	-8.55 [-19.76, 1.99]	-48.58 [-59.1, -38.96]*	-57.15 [-67.86, -47.56]*
L 19	L 1080	L Induseum griseum	0.51 ± 0.51	15	16.37 ± 12.13	13	11.87 ± 9.26	12	54.85 ± 19.84	12	18.51 [11.57, 25.54]*	20.54 [13.6, 27.53]*	23.08 [15.04, 31.11]*	-26.39 [-34.41, -18.61]*	-20.23 [-27.97, -12.15]*	-75.1 [-86.11, -64.61]*	-6.21 [-16.78, 4.74]	-48.68 [-58.91, -38.93]*	-54.88 [-64.84, -44.57]*
R 822	R 1089	R Retrohippocampal region	2.34 ± 0.69	14	5.58 ± 2.14	14	2.92 ± 1.07	12	14.98 ± 3.14	9	-1 [-7.96, 6.08]	-2.45 [-9.4, 4.58]	-1.7 [-9.93, 6.5]						
L 822	L 1089	L Retrohippocampal region	1.78 ± 0.60	14	4.99 ± 1.86	16	1.74 ± 0.50	12	9.33 ± 2.84	11	-1.19 [-8.14, 5.9]	-2.68 [-9.51, 4.07]	-2.07 [-10.22, 6.12]						
R 909	R 822	R Entorhinal area	2.66 ± 0.88	14	5.33 ± 1.68	13	2.10 ± 0.77	11	18.22 ± 4.64	9	-0.92 [-8.01, 6.19]	-2.52 [-9.7, 4.48]	-1.4 [-9.46, 6.82]						
L 909	L 822	L Entorhinal area	2.08 ± 0.69	14	5.68 ± 1.80	16	1.95 ± 0.59	11	11.93 ± 3.69	11	-1.19 [-8.21, 5.88]	-2.64 [-9.43, 4.17]	-1.87 [-10.09, 6.17]						
R 918	R 909	R Entorhinal area, lateral part	4.11 ± 1.36	14	8.27 ± 2.61	13	3.24 ± 1.18	11	28.09 ± 7.15	9	-0.96 [-8.1, 6.19]	-2.52 [-9.45, 4.47]	-1.42 [-9.65, 6.82]						
L 918	L 909	L Entorhinal area, lateral part	3.21 ± 1.07	14	9.29 ± 3.23	16	3.01 ± 0.91	11	18.42 ± 5.69	11	-1.24 [-8.28, 5.67]	-2.56 [-9.5, 4.34]	-1.91 [-10.01, 6.15]						
R 502	R 822	R Subiculum	7.24 ± 2.88	12	21.06 ± 9.08	11	20.49 ± 10.80	10	31.91 ± 6.98	7	-0.62 [-7.96, 6.68]	-2.82 [-10.04, 4.51]	-1.99 [-10.37, 6.47]						
L 502	L 822	L Subiculum	7.28 ± 2.58	12	19.18 ± 12.82	12	6.74 ± 2.08	10	24.45 ± 8.55	7	-1.09 [-8.49, 6.29]	-3.28 [-10.39, 3.98]	-1.86 [-10.22, 6.39]						
R 484682	R 822	R Prosubiculum	1.51 ± 1.14	10	19.98 ± 8.23	10	22.49 ± 6.78	10	53.19 ± 13.12	7	6.14 [-1.18, 13.4]	2.87 [-4.57, 10.18]	3.33 [-5.03, 11.68]						
L 484682	L 822	L Prosubiculum	4.00 ± 2.67	10	14.14 ± 7.89	11	5.97 ± 2.93	10	31.01 ± 13.21	7	-0.58 [-7.84, 6.67]	-2.01 [-9.24, 5.26]	-1.02 [-9.28, 7.39]						
R 703	R 688	R Cortical subplate	5.74 ± 1.15	15	12.77 ± 3.41	16	6.13 ± 1.12	12	29.73 ± 5.83	12	-0.24 [-1.94, 1.48]	1.12 [-0.5, 2.77]	2.79 [0.37, 5.23]*	-1.24 [-3.26, 0.72]	-0.07 [-2.11, 1.95]	-4.55 [-6.59, -2.3]*	-1.16 [-3.31, 0.94]	-3.29 [-5.4, -1.17]*	-4.46 [-6.66, -2.19]*
L 703	L 688	L Cortical subplate	4.78 ± 0.76	15	12.95 ± 3.38	16	6.81 ± 1.22	12	28.76 ± 5.36	12	0.12 [-1.56, 1.81]	1.6 [-0.03, 3.21]	2.78 [0.31, 5.16]*	-1.74 [-3.62, 0.24]	-0.44 [-2.43, 1.61]	-5.37 [-7.57, -3.29]*	-1.27 [-3.47, 0.73]	-3.64 [-5.85, -1.65]*	-4.92 [-7.2, -2.77]*
R 583	R 703	R Claustrum	18.66 ± 5.42	15	41.93 ± 14.47	15	31.45 ± 8.47	12	106.07 ± 15.90	12	0.38 [-1.31, 2.06]	1.14 [-0.5, 2.78]	2.64 [0.13, 5.12]*	-1.26 [-3.28, 0.71]	-0.7 [-2.77, 1.28]	-5.03 [-7.26, -3]*	-0.56 [-2.68, 1.52]	-3.76 [-5.99, -1.73]*	-4.32 [-6.6, -2.13]*
L 583	L 703	L Claustrum	18.06 ± 4.12	15	41.60 ± 13.14	15	27.92 ± 4.68	12	108.11 ± 19.85	12	0.24 [-1.45, 1.95]	1.2 [-0.46, 2.85]	3.04 [0.61, 5.47]*	-1.33 [-3.37, 0.57]	-0.56 [-2.54, 1.52]	-5.33 [-7.45, -3.16]*	-0.77 [-2.89, 1.29]	-4 [-6.1, -1.9]*	-4.76 [-6.93, -2.49]*
R 942	R 703	R Endopiriform nucleus	6.55 ± 1.50	13	16.32 ± 4.66	14	7.14 ± 1.39	12	28.77 ± 5.41	12	-0.2 [-1.95, 1.5]	1.16 [-0.51, 2.86]	1.94 [-0.52, 4.45]						
L 942	L 703	L Endopiriform nucleus	5.07 ± 0.83	14	13.44 ± 3.77	16	8.24 ± 1.53	12	34.08 ± 6.71	11	0.36 [-1.35, 2.05]	1.58 [-0.08, 3.19]	2.93 [0.46, 5.36]*	-1.72 [-3.67, 0.3]	-0.68 [-2.73, 1.35]	-5.74 [-7.91, -3.53]*	-1.04 [-3.18, 1]	-4.02 [-6.18, -1.9]*	-5.06 [-7.34, -2.86]*
R 952	R 942	R Endopiriform nucleus, dorsal part	13.00 ± 3.30	13	36.72 ± 10.53	15	12.48 ± 2.04	12	55.72 ± 9.41	12	-0.33 [-2.04, 1.39]	1.65 [0, 3.31]	1.48 [-0.98, 3.92]	-1.77 [-3.77, 0.26]	0.02 [-1.98, 2.12]	-3.66 [-5.84, -1.52]*	-1.78 [-3.89, 0.31]	-1.88 [-4.03, 0.21]	-3.66 [-5.88, -1.49]*
L 952	L 942	L Endopiriform nucleus, dorsal part	10.88 ± 1.85	14	27.75 ± 7.25	16	16.92 ± 3.05	12	62.27 ± 12.59	12	0.29 [-1.46, 2.03]	1.48 [-0.22, 3.19]	2.48 [0, 4.95]						
R 966	R 942	R Endopiriform nucleus, ventral part	13.07 ± 3.56	13	19.91 ± 5.96	15	17.62 ± 4.84	12	60.30 ± 13.57	12	0.06 [-1.69, 1.79]	0.36 [-1.27, 2.05]	2.69 [0.25, 5.11]*	-0.5 [-2.45, 1.55]	-0.39 [-2.54, 1.59]	-3.98 [-6.26, -1.89]*	-0.11 [-2.31, 1.9]	-3.5 [-5.66, -1.44]*	-3.61 [-5.83, -1.35]*
L 966	L 942	L Endopiriform nucleus, ventral part	8.68 ± 1.81	14	25.82 ± 8.54	16	15.78 ± 3.45	12	63.55 ± 15.57	12	0.56 [-1.16, 2.24]	1.91 [0.24, 3.54]*	3.39 [0.94, 5.78]*	-2.04 [-4.05, -0.1]*	-0.87 [-2.91, 1.18]	-6.72 [-8.96, -4.59]*	-1.16 [-3.34, 0.9]	-4.69 [-6.8, -2.53]*	-5.83 [-8.1, -3.63]*

R 131	R 703	R Lateral amygdalar nucleus	11.85 ± 3.78	14	33.91 ± 9.13	15	11.11 ± 2.27	12	69.13 ± 15.59	12	-0.3 [-2.03, 1.44]	1.75 [0.1, 3.38]*	2.94 [0.48, 5.42]*	-1.88 [-3.83, 0.17]	-0.03 [-2.12, 1.93]	-5.26 [-7.37, -3.13]*	-1.86 [-3.99, 0.29]	-3.38 [-5.45, -1.21]*	-5.24 [-7.43, -2.96]*
L 131	L 703	L Lateral amygdalar nucleus	6.60 ± 1.94	14	37.24 ± 10.12	16	12.61 ± 3.04	12	56.53 ± 13.12	12	0.67 [-1.04, 2.38]	4.6 [2.93, 6.24]*	1.85 [-0.59, 4.34]	-4.75 [-6.68, -2.72]*	-0.99 [-3.08, 0.98]	-7.99 [-10.26, -5.93]*	-3.75 [-5.84, -1.61]*	-3.25 [-5.38, -1.16]*	-6.99 [-9.28, -4.79]*
R 295	R 703	R Basolateral amygdalar nucleus	7.80 ± 1.80	15	19.23 ± 5.51	14	9.71 ± 2.01	9	44.45 ± 9.72	12	-0.08 [-1.89, 1.68]	1.12 [-0.57, 2.76]	3.14 [0.69, 5.68]*	-1.26 [-3.16, 0.78]	-0.24 [-2.38, 1.82]	-5.06 [-7.23, -2.93]*	-1.01 [-3.3, 1.1]	-3.8 [-6.01, -1.75]*	-4.81 [-7.16, -2.59]*
L 295	L 703	L Basolateral amygdalar nucleus	6.04 ± 1.23	14	19.49 ± 5.65	14	9.68 ± 2.38	10	48.97 ± 8.65	11	0.41 [-1.35, 2.18]	1.84 [0.19, 3.53]*	4.01 [1.48, 6.51]*	-1.98 [-3.99, -0.02]*	-0.74 [-2.72, 1.43]	-7.13 [-9.32, -4.96]*	-1.23 [-3.43, 0.93]	-5.15 [-7.31, -3.07]*	-6.4 [-8.71, -4.1]*
R 303	R 295	R Basolateral amygdalar nucleus, anterior	23.11 ± 5.94	15	41.34 ± 10.69	13	31.69 ± 6.81	9	111.23 ± 25.99	12	0.05 [-1.7, 1.85]	0.88 [-0.81, 2.54]	2.37 [-0.16, 4.88]						
L 303	L 295	L Basolateral amygdalar nucleus, anterior p	16.63 ± 3.38	14	48.80 ± 11.50	14	28.29 ± 7.46	10	132.88 ± 22.42	11	0.51 [-1.23, 2.23]	1.55 [-0.12, 3.23]	4.08 [1.55, 6.58]*	-1.7 [-3.66, 0.32]	-0.84 [-2.85, 1.33]	-7 [-9.17, -4.84]*	-0.84 [-3.01, 1.31]	-5.33 [-7.61, -3.3]*	-6.17 [-8.48, -3.89]*
R 311	R 295	R Basolateral amygdalar nucleus, posterior	9.43 ± 3.52	14	27.76 ± 10.45	13	8.08 ± 2.77	9	83.37 ± 22.15	9	-0.4 [-2.22, 1.4]	2.19 [0.5, 3.87]*	5.28 [2.71, 7.88]*	-2.32 [-4.33, -0.27]*	0.08 [-2.08, 2.21]	-7.94 [-10.21, -5.78]*	-2.41 [-4.63, -0.15]*	-5.61 [-7.92, -3.47]*	-8.01 [-10.48, -5.75]*
L 311	L 295	L Basolateral amygdalar nucleus, posterior	8.28 ± 2.63	13	33.23 ± 14.68	14	8.01 ± 2.91	10	69.34 ± 14.70	10	-0.15 [-1.95, 1.59]	2.71 [1.05, 4.36]*	4.01 [1.5, 6.52]*	-2.84 [-4.83, -0.85]*	-0.17 [-2.24, 1.95]	-7.43 [-9.67, -5.16]*	-2.67 [-4.75, -0.41]*	-4.58 [-6.72, -2.34]*	-7.26 [-9.57, -4.98]*
R 451	R 295	R Basolateral amygdalar nucleus, ventral p	10.31 ± 3.25	14	14.17 ± 4.06	12	10.49 ± 2.65	9	64.97 ± 12.25	10	-0.25 [-2.05, 1.54]	1.13 [-0.54, 2.81]	4.09 [1.53, 6.63]*	-1.27 [-3.24, 0.81]	-0.07 [-2.25, 2.02]	-5.85 [-8.02, -3.61]*	-1.19 [-3.36, 1.04]	-4.58 [-6.78, -2.38]*	-5.77 [-8.09, -3.49]*
L 451	L 295	L Basolateral amygdalar nucleus, ventral p	7.05 ± 2.08	12	16.31 ± 4.96	13	12.63 ± 5.91	10	66.23 ± 17.60	10	0.69 [-1.12, 2.51]	1.46 [-0.29, 3.17]	5.47 [2.87, 8.08]*	-1.59 [-3.57, 0.52]	-1.01 [-3.12, 1.14]	-8.49 [-10.71, -6.26]*	-0.59 [-2.74, 1.56]	-6.89 [-9.12, -4.77]*	-7.48 [-9.79, -5.2]*
R 319	R 703	R Basomedial amygdalar nucleus	10.85 ± 2.29	14	20.10 ± 4.75	14	10.71 ± 3.12	9	51.90 ± 11.72	12	-0.19 [-1.96, 1.56]	0.6 [-1.06, 2.26]	2.85 [0.37, 5.35]*	-0.72 [-2.74, 1.32]	-0.14 [-2.26, 2.01]	-4.14 [-6.28, -1.97]*	-0.59 [-2.83, 1.57]	-3.4 [-5.48, -1.18]*	-4 [-6.23, -1.65]*
L 319	L 703	L Basomedial amygdalar nucleus	11.38 ± 2.37	15	23.07 ± 7.24	14	12.68 ± 3.10	11	50.19 ± 9.85	12	-0.18 [-1.92, 1.53]	1.28 [-0.41, 2.95]	1.81 [-0.7, 4.32]						
R 327	R 319	R Basomedial amygdalar nucleus, anterior	29.35 ± 5.44	14	55.53 ± 13.90	13	28.59 ± 7.83	9	119.13 ± 24.17	12	-0.2 [-2, 1.59]	1.08 [-0.62, 2.75]	1.67 [-0.88, 4.16]						
L 327	L 319	L Basomedial amygdalar nucleus, anterior	29.25 ± 6.31	15	65.09 ± 22.99	12	37.64 ± 11.02	11	111.96 ± 22.29	12	-0.01 [-1.77, 1.68]	2.04 [0.36, 3.72]*	0.29 [-2.13, 2.83]	-2.18 [-4.26, -0.17]*	-0.31 [-2.41, 1.72]	-3.19 [-5.38, -1.08]*	-1.86 [-4.01, 0.33]	-1.01 [-3.2, 1.08]	-2.87 [-5.12, -0.69]*
R 334	R 319	R Basomedial amygdalar nucleus, posterio	12.33 ± 5.17	13	20.80 ± 8.41	12	13.47 ± 4.90	9	68.79 ± 18.46	9	0 [-1.83, 1.82]	0.97 [-0.77, 2.7]	2.84 [0.22, 5.43]*	-1.1 [-3.19, 0.88]	-0.32 [-2.54, 1.74]	-4.67 [-6.92, -2.45]*	-0.78 [-3.01, 1.43]	-3.58 [-5.82, -1.41]*	-4.35 [-6.58, -1.94]*
L 334	L 319	L Basomedial amygdalar nucleus, posterior	12.23 ± 3.28	14	32.57 ± 14.32	14	10.42 ± 3.11	10	71.97 ± 15.41	11	-0.34 [-2.08, 1.46]	1.98 [0.31, 3.63]*	2.81 [0.25, 5.3]*	-2.12 [-4.13, -0.12]*	0.01 [-2.11, 2.1]	-5.33 [-7.42, -3]*	-2.13 [-4.27, 0.07]	-3.2 [-5.41, -1.14]*	-5.34 [-7.59, -3]*
R 780	R 703	R Posterior amygdalar nucleus	11.44 ± 3.52	12	30.84 ± 10.69	12	9.94 ± 2.57	11	73.68 ± 28.57	8	-0.36 [-2.11, 1.4]	1.85 [0.1, 3.58]*	3.48 [0.87, 6.07]*	-1.98 [-3.99, 0.16]	0.04 [-2.11, 2.09]	-5.84 [-8.12, -3.51]*	-2.01 [-4.22, 0.12]	-3.87 [-6.03, -1.57]*	-5.87 [-8.12, -3.51]*
L 780	L 703	L Posterior amygdalar nucleus	9.90 ± 3.56	12	19.16 ± 6.28	14	11.00 ± 4.82	11	46.09 ± 17.83	9	-0.11 [-1.87, 1.63]	1.23 [-0.47, 2.95]	1.98 [-0.56, 4.51]						
R 477	R 623	R Striatum	3.90 ± 0.79	15	8.32 ± 1.57	16	3.98 ± 0.56	12	12.29 ± 2.05	12	-0.32 [-1.99, 1.33]	0.98 [-0.55, 2.54]	0.8 [-1.54, 3.1]						
L 477	L 623	L Striatum	3.45 ± 0.60	15	8.44 ± 1.57	16	4.53 ± 0.72	12	13.19 ± 1.87	11	-0.02 [-1.68, 1.64]	1.3 [-0.24, 2.89]	0.58 [-1.74, 2.91]						
R 485	R 477	R Striatum dorsal region	2.65 ± 0.50	15	8.68 ± 2.08	16	3.28 ± 0.49	12	10.09 ± 1.79	12	-0.09 [-1.76, 1.53]	2.13 [0.57, 3.7]*	0.07 [-2.2, 2.32]	-2.29 [-3.7, -0.82]*	-0.24 [-1.77, 1.32]	-2.95 [-4.52, -1.31]*	-2.06 [-3.7, -0.5]*	-0.66 [-2.26, 0.9]	-2.71 [-4.31, -0.96]*
L 485	L 477	L Striatum dorsal region	2.24 ± 0.42	15	7.53 ± 1.59	16	3.05 ± 0.49	12	9.56 ± 1.97	12	0.01 [-1.57, 1.67]	2.21 [0.7, 3.81]*	0.35 [-1.96, 2.6]	-2.38 [-3.87, -0.96]*	-0.34 [-1.91, 1.2]	-3.4 [-5.05, -1.88]*	-2.03 [-3.64, -0.5]*	-1.04 [-2.59, 0.56]	-3.06 [-4.73, -1.4]*
R 672	R 485	R Caudoputamen	5.68 ± 1.00	14	18.53 ± 4.27	15	6.55 ± 0.98	12	22.02 ± 3.37	11	-0.12 [-1.77, 1.58]	2.09 [0.48, 3.71]*	0.17 [-2.22, 2.43]	-2.25 [-3.79, -0.79]*	-0.22 [-1.83, 1.32]	-2.98 [-4.63, -1.37]*	-2.03 [-3.57, -0.41]*	-0.73 [-2.28, 0.9]	-2.76 [-4.39, -1.04]*
L 672	L 485	L Caudoputamen	5.17 ± 0.82	13	15.06 ± 3.18	16	6.09 ± 0.98	12	22.94 ± 3.61	10	-0.06 [-1.73, 1.6]	1.87 [0.33, 3.45]*	0.62 [-1.75, 2.93]	-2.03 [-3.54, -0.55]*	-0.28 [-1.87, 1.27]	-3.26 [-4.98, -1.6]*	-1.74 [-3.36, -0.19]*	-1.24 [-2.9, 0.34]	-2.98 [-4.73, -1.28]*
R 493	R 477	R Striatum ventral region	5.72 ± 1.23	15	7.32 ± 1.39	15	4.60 ± 0.70	12	14.77 ± 3.25	12	-0.54 [-2.19, 1.11]	0.29 [-1.26, 1.87]	1.13 [-1.18, 3.41]						
L 493	L 477	L Striatum ventral region	4.46 ± 0.85	15	8.57 ± 1.50	15	6.01 ± 1.05	12	14.42 ± 2.91	12	0.01 [-1.65, 1.66]	0.93 [-0.65, 2.53]	0.6 [-1.7, 2.87]						
R 56	R 493	R Nucleus accumbens	9.77 ± 1.91	12	16.98 ± 3.92	13	9.70 ± 1.49	12	36.39 ± 8.05	10	-0.21 [-1.91, 1.5]	0.79 [-0.86, 2.47]	1.74 [-0.71, 4.12]						
L 56	L 493	L Nucleus accumbens	6.96 ± 1.19	13	17.07 ± 3.95	14	11.88 ± 2.70	12	37.05 ± 8.24	10	0.41 [-1.27, 2.11]	1.38 [-0.24, 3.02]	2.03 [-0.37, 4.39]						
R 754	R 493	R Olfactory tubercle	17.84 ± 5.20	13	16.84 ± 2.66	12	8.86 ± 1.94	10	27.80 ± 7.05	11	-0.64 [-2.4, 1.11]	-0.18 [-1.82, 1.48]	0.71 [-1.73, 3.13]						
L 754	L 493	L Olfactory tubercle	13.76 ± 3.44	13	18.63 ± 3.87	13	11.84 ± 2.05	12	22.29 ± 3.61	9	-0.37 [-2.01, 1.31]	0.52 [-1.1, 2.17]	-0.28 [-2.68, 2.1]						
R 275	R 477	R Lateral septal complex	12.94 ± 3.68	14	21.87 ± 5.28	13	13.45 ± 1.87	12	28.37 ± 5.05	12	-0.3 [-1.91, 1.36]	0.46 [-1.14, 2.07]	0.33 [-1.99, 2.57]						
L 275	L 477	L Lateral septal complex	11.84 ± 2.38	14	24.52 ± 5.16	13	11.39 ± 1.63	12	27.39 ± 6.30	12	-0.36 [-2.04, 1.3]	0.86 [-0.74, 2.47]	0.13 [-2.26, 2.47]						
R 242	R 275	R Lateral septal nucleus	19.97 ± 5.55	14	33.33 ± 8.11	13	20.45 ± 2.67	12	44.13 ± 8.68	12	-0.31 [-1.98, 1.36]	0.45 [-1.12, 2.06]	0.38 [-1.91, 2.71]						
L 242	L 275	L Lateral septal nucleus	18.88 ± 3.84	14	38.14 ± 7.65	13	17.90 ± 2.61	12	43.11 ± 10.51	12	-0.4 [-2.03, 1.29]	0.78 [-0.83, 2.44]	0.2 [-2.18, 2.55]						
R 258	R 242	R Lateral septal nucleus, rostral (rostrover	35.85 ± 9.38	14	68.00 ± 16.64	12	38.69 ± 5.30	12	79.48 ± 18.22	12	-0.24 [-1.93, 1.44]	0.52 [-1.12, 2.17]	0.24 [-2.16, 2.61]						
L 258	L 242	L Lateral septal nucleus, rostral (rostroven	35.22 ± 7.40	14	75.02 ± 16.20	12	33.98 ± 4.59	12	78.72 ± 20.94	12	-0.38 [-2.06, 1.29]	0.74 [-0.88, 2.37]	0.17 [-2.18, 2.54]						
R 266	R 242	R Lateral septal nucleus, ventral part	68.77 ± 28.74	11	75.81 ± 26.88	7	63.34 ± 11.90	10	116.88 ± 24.77	10	-0.38 [-2.14, 1.42]	0.17 [-1.62, 2.07]	0.32 [-2.3, 2.9]						
L 266	L 242	L Lateral septal nucleus, ventral part	46.13 ± 15.73	10	79.99 ± 24.55	9	52.91 ± 19.22	11	139.14 ± 31.81	9	-0.27 [-2.03, 1.48]	0.6 [-1.21, 2.4]	1.04 [-1.45, 3.56]						
R 333	R 275	R Septohippocampal nucleus	13.96 ± 6.89	14	17.06 ± 8.99	8	19.58 ± 12.76	10	6.21 ± 4.56	7	-0.01 [-1.73, 1.68]	0.75 [-1.03, 2.55]	-1.73 [-4.31, 0.81]						
L 333	L 275	L Septohippocampal nucleus	3.28 ± 2.62	13	22.64 ± 10.95	8	2.73 ± 1.84	10	19.55 ± 11.11	7	-0.58 [-2.29, 1.15]	6.42 [4.65, 8.2]*	-1.31 [-3.93, 1.29]	-6.58 [-8.26, -4.89]*	0.25 [-1.38, 1.83]	-5.36 [-7.17, -3.59]*	-6.83 [-8.56, -5.02]*	1.21 [-0.7, 3.07]	-5.62 [-7.48, -3.74]*
R 278	R 477	R Striatum-like amygdalar nuclei	9.22 ± 2.09	15	16.64 ± 3.09	15	9.69 ± 2.11	12	35.13 ± 7.03	12	-0.29 [-1.94, 1.35]	0.84 [-0.72, 2.42]	1.55 [-0.78, 3.81]						
L 278	L 477	L Striatum-like amygdalar nuclei	8.81 ± 1.63	15	20.04 ± 4.80	15	12.96 ± 2.61	12	35.56 ± 5.61	12	0.13 [-1.53, 1.79]	1.32 [-0.24, 2.91]	0.89 [-1.44, 3.22]						
R 23	R 278	R Anterior amygdalar area	12.99 ± 4.25	13	18.16 ± 6.36	9	13.37 ± 4.13	9	32.16 ± 5.54	8	-0.31 [-2.07, 1.47]	0.42 [-1.29, 2.17]	0.99 [-1.57, 3.48]						
R 536	R 278	R Central amygdalar nucleus	6.43 ± 1.26	15	15.87 ± 3.50	15	9.12 ± 2.33	11	27.61 ± 5.56	12	0.02 [-1.61, 1.65]	1.51 [-0.07, 3.11]	1.07 [-1.22, 3.34]						
L 536	L 278	L Central amygdalar nucleus	6.81 ± 1.33	14	16.85 ± 4.25	14	10.95 ± 2.52	12	33.78 ± 5.93	12	0.32 [-1.33, 1.96]	1.75 [0.19, 3.34]*	1.24 [-1.09, 3.56]	-1.91 [-3.44, -0.45]*	-0.67 [-2.26, 0.85]	-4.14 [-5.76, -2.56]*	-1.25 [-2.86, 0.3]	-2.24 [-3.85, -0.64]*	-3.5 [-5.17, -1.86]*
R 544	R 536	R Central amygdalar nucleus, capsular par	9.29 ± 2.31	14	40.44 ± 11.74	15	23.77 ± 8.71	11	74.55 ± 14.22	12	1.22 [-0.43, 2.87]	3.46 [1.86, 5.05]*	1.72 [-0.57, 4.02]	-3.62 [-5.07, -2.15]*	-1.56 [-3.1, 0.02]	-7.23 [-8.92, -5.67]*	-2.07 [-3.59, -0.42]*	-3.6 [-5.29, -2.11]*	-5.68 [-7.38, -4]*
L 544	L 536	L Central amygdalar nucleus, capsular part	12.53 ± 2.94	13	34.52 ± 7.58	14	34.44 ± 12.31	12	84.46 ± 17.21	12	1.53 [-0.16, 3.2]	2.09 [0.47, 3.75]*	1.54 [-0.81, 3.91]	-2.25 [-3.8, -0.72]*	-1.86 [-3.41, -0.23]*	-6 [-7.62, -4.39]*	-0.39 [-1.96, 1.22]	-3.74 [-5.39, -2.19]*	-4.14 [-5.77, -2.49]*
R 551	R 536	R Central amygdalar nucleus, lateral part	13.69 ± 4.08	14	40.88 ± 7.38	15	14.10 ± 3.96	11	73.62 ± 17.83	12	-0.31 [-2, 1.35]	2.09 [0.52, 3.7]*	1.97 [-0.37, 4.28]	-2.26 [-3.69, -0.73]*	-0.02 [-1.61, 1.54]	-4.58 [-6.22, -2.99]*	-2.22 [-3.92, -0.7]*	-2.33 [-3.88, -0.74]*	-4.56 [-6.22, -2.86]*
L 551	L 536	L Central amygdalar nucleus, lateral part	11.88 ± 3.31	13	39.91 ± 10.47	14	19.32 ± 4.71	12	83.61 ± 18.71	12	0.41 [-1.26, 2.1]	2.71 [1.15, 4.32]*	2.34 [0.04, 4.64]*	-2.87 [-4.39, -1.36]*	-0.76 [-2.26, 0.93]	-6.3 [-7.99, -4.69]*	-2.12 [-3.7, -0.49]*	-3.42 [-5.04, -1.82]*	-5.56 [-7.29, -3.93]*
R 559	R 536	R Central amygdalar nucleus, medial part	14.63 ± 2.87	15	21.14 ± 5.89	14	18.21 ± 4.40	11	42.00 ± 8.89	12	-0.16 [-1.81, 1.54]	0.66 [-0.91, 2.27]	0.67 [-1.69, 3.01]						
L 559	L 536	L Central amygdalar nucleus, medial part	15.76 ± 3.68	14	31.16 ± 9.31	14	18.71 ± 4.73	12	54.77 ± 7.78	12	-0.11 [-1.76, 1.57]	1.25 [-0.35, 2.84]	0.69 [-1.67, 3.01]						
R 1105	R 278	R Intercalated amygdalar nucleus	21.10 ± 5.77	15	36.40 ± 12.31	14	30.68 ± 8.46	12	90.37 ± 39.04	12	0.13 [-1.51, 1.78]	0.96 [-0.64, 2.56]	1.5 [-0.83, 3.81]						
L 1105	L 278	L Intercalated amygdalar nucleus	17.67 ± 4.93	15	42.19 ± 10.44	14	33.67 ± 5.81	12	78.20 ± 16.76	12	0.57 [-1.05, 2.22]	1.37 [-0.19, 2.97]	0.78 [-1.53, 3.09]						
R 403	R 278	R Medial amygdalar nucleus	29.77 ± 7.14	15	47.16 ± 9.39	15	27.44 ± 6.10	12	106.46 ± 19.59	12	-0.41 [-2.07, 1.26]	0.63 [-0.9, 2.24]	1.66 [-0.65, 3.97]						
L 403	L 278	L Medial amygdalar nucleus	28.63 ± 5.64	15	60.02 ± 16.42	15	40.35 ± 9.28	12	106.53 ± 18.44	12	0.07 [-1.59, 1.77]	1.13 [-0.42, 2.75]	0.82 [-1.5, 3.12]						
R 803	R 623	R Pallidum	2.37 ± 0.54	15	3.96 ± 1.08	15	2.62 ± 0.57	11	5.47 ± 1.05	12	-1.77 [-32.51, 29.11]	-6.65 [-35.95, 23.3]	-11.62 [-54.89, 32.46]						
L 803	L 623	L Pallidum	2.22 ± 0.41	15	2.88 ± 0.54	15	2.72 ± 0.34	12	6.70 ± 1.54	12	-1.81 [-31.65, 28.8]	-7.16 [-36.59, 22.15]	-10.7 [-53.96, 32.61]						
R 818	R 803	R Pallidum, dorsal region	1.85 ± 0.48	15	2.67 ± 0.77	14	2.02 ± 0.56	12	4.63 ± 0.80	12	-1.55 [-31.59, 28.18]	-6.77 [-36.37, 22.64]	-11.44 [-55.05, 31.2]						
L 818	L 803	L Pallidum, dorsal region	2.10 ± 0.44	15	2.49 ± 0.57	14	1.88 ± 0.43	12	4.10 ± 0.94	12	-1.95 [-31.25, 27.95]	-7.33 [-36.64, 22.07]	-11.2 [-54.68, 31.63]						
R 1022	R 818	R Globus pallidus, external segment	4.07 ± 1.02	15	6.94 ± 1.94	14	5.64 ± 1.41	11	13.23 ± 2.57	12	-1.45 [-31.71, 28.85]	-6.74 [-37.13, 22.75]	-11.17 [-55.41, 32.86]						
L 1022	L 818	L Globus pallidus, external segment	4.04 ± 0.95	14	7.01 ± 1.65	14	4.53 ± 1.53	12	9.86 ± 2.12	12	-1.72 [-31.81, 29.07]	-6.84 [-36.92, 23.37]	-11.53 [-56.58, 32.63]						
R 1031	R 818	R Globus pallidus, internal segment	3.73 ± 2.32	14	3.12 ± 1.18	13	1.47 ± 0.71	11	2.20 ± 1.09	12	-2.44 [-34.61, 28.37]	-7.8 [-38.53, 23.45]	-11.78 [-57.64, 33]						
L 1031	L 818	L Globus pallidus, internal segment	5.93 ± 1.57	13	1.71 ± 0.74	14	2.90 ± 1.18	12	4.29 ± 1.57	11	-2.05 [-33.41, 29.36]	-7.85 [-38.57, 23.2]	-11.64 [-55.54, 33.5]						
R 835	R 803	R Pallidum, ventral region	3.73 ± 0.90	15	5.28 ± 1.46	15	3.04 ± 0.84	12	9.73 ± 3.03	12	-1.93 [-31.56, 27.58]	-7.11 [-36.41, 22.54]	-10.85 [-53.3, 32.37]						
L 835	L 803	L Pallidum, ventral region	3.82 ± 0.86	15	5.14 ± 1.59	15	4.55 ± 0.79	12	11.38 ± 3.20	12	-1.47 [-31.77, 28.96]	-7.13 [-36.82, 22.51]	-10.76 [-53.47, 32.92]						
R 342	R 835	R Substantia innominata	7.29 ± 1.90	15	10.69 ± 2.93	15	6.66 ± 1.77	11	21.87 ± 6.41	11	-2.13 [-33.51, 28.97]	-7.09 [-36.66, 22.42]	-10.56 [-54.62, 33.34]						
L 342	L 835	L Substantia innominata	7.75 ± 1.78	15	10.80 ± 3.38	14	9.40 ± 1.57	12	25.70 ± 7.04	11	-1.63 [-31.83, 28.49]	-6.89 [-36.98, 23.52]	-10.47 [-53.47, 33.46]						
R 826	R 803	R Pallidum, medial region	4.45 ± 1.87	14	7.85 ± 2.83	12	4.13 ± 1.32	11	5.12 ± 1.25	11	-1.79 [-32.99, 29.74]	-6.65 [-37.92, 25.18]	-12.87 [-58.57, 33.68]						
L 826	L 803	L Pallidum, medial region	2.59 ± 1.01	14	3.62 ± 1.07	11	6.12 ± 2.18	11	7.46 ± 1.69	11	-0.39 [-31.39, 31.49]	-7.17 [-39.29, 25.09]	-12.29 [-58.62, 33.08]						
R 904	R 826	R Medial septal complex	6.67 ± 2.81	14	10.27 ± 4.10	12	5.84 ± 1.95	11	5.71 ± 1.39	11	-1.54 [-32.4, 29.8]	-6.72 [-39.16, 25.2]	-13.24 [-58.47, 32.4]						
L 904	L 826	L Medial septal complex	3.89 ± 1.52	14	5.43 ± 1.60	11	7.90 ± 3.26	11	10.40 ± 2.17	11	-1.01 [-32.02, 30.27]	-7.25 [-39.9, 24.99]	-11.75 [-57.31, 35.31]						
R 564	R 904	R Medial septal nucleus	0.00 ± 0.00	9	9.07 ± 2.48	6	2.85 ± 1.46	8	22.38 ± 7.90	8	26.79 [-10.89, 63.91]	83.21 [41.39, 125.66]	92.36 [36.15, 149.33]*	90.65 [-131.96, -50.11]	-28.73 [-66.17, 7.9]	224.16 [-262.44, -186.92	-62.25 [-102.59, -19.03]*	-133.6 [-174.89, -91.15]*	-195.42 [-233.17, -154.74]*
L 564	L 904	L Medial septal nucleus	0.00 ± 0.00	8	12.46 ± 5.86	6	7.26 ± 3.70	8	26.15 ± 12.72	8	70.97 [31.84, 110.18]*	117 [73.64, 159.78]*	52.02 [-6.47, 109.94]	124.47 [-167.21, -83.36	-72.81 [-111.67, -34.35]*	261.94 [-299.99, -222.68	-51.79 [-95.44, -11.74]*	137.17 [-178.18, -94.54]	-188.95 [-228.38, -151.22]*
R 596	R 904	R Diagonal band nucleus	14.21 ± 5.68	14	20.28 ± 8.27	12	12.99 ± 4.01	11	7.69 ± 2.65	11	-2.03 [-33.64, 29.22]	-7.17 [-39.19, 23.79]	-12.88 [-57.79, 33.44]						
L 596	L 904	L Diagonal band nucleus	8.83 ± 3.37	14	9.98 ± 3.28	11	15.12 ± 6.62	11	20.50 ± 4.05	11	-1.07 [-33.07, 29.94]	-7.35 [-39.53, 24.95]	-11.91 [-56.8, 34.36]						
R 549	R 1129	R Thalamus	2.84 ± 0.73	15	3.20 ± 0.60	16	4.49 ± 1.05	12	7.52 ± 1.26	12	-0.19 [-2.76, 2.37]	0.51 [-2.04, 2.97]	-0.2 [-3.84, 3.48]						
L 549	L 1129	L Thalamus	2.67 ± 0.55	14	3.03 ± 0.56	16	5.07 ± 1.15	12	7.56 ± 1.14	12	0.27 [-2.31, 2.82]	0.69 [-1.84, 3.21]	-0.52 [-4.18, 3.02]						
R 864	R 549	R Thalamus, sensory-motor cortex related	2.26 ± 0.51	15	2.96 ± 0.75	16	5.72 ± 1.42	12	9.68 ± 1.91	12	0.66 [-1.93, 3.24]	0.46 [-2.11, 3]	-0.17 [-3.8, 3.46]						
L 864	L 549	L Thalamus, sensory-motor cortex related	2.40 ± 0.60	14	2.80 ± 0.59	16	6.25 ± 1.52	12	9.21 ± 1.51	12	1.05 [-1.47, 3.64]	1.15 [-1.38, 3.69]	-1.03 [-4.69, 2.52]						
R 637	R 864	R Ventral group of the dorsal thalamus	2.48 ± 0.55	15	3.19 ± 0.72	16	6.52 ± 1.52	12	13.07 ± 2.64	12	0.61 [-1.94, 3.13]	0.2 [-2.27, 2.7]	0.28 [-3.35, 3.8]						
L 637	L 864	L Ventral group of the dorsal thalamus	2.47 ± 0.49	14	3.26 ± 0.62	16	7.26 ± 1.86	12	11.95 ± 2.05	12	1.33 [-1.23, 3.95]	1.54 [-1.04, 4.03]	-1.29 [-4.95, 2.37]						
R 629	R 637	R Ventral anterior-lateral complex of the th	2.80 ± 0.81	15	2.11 ± 0.87	14	11.25 ± 2.71	12	30.56 ± 7.90	12	1.93 [-0.6, 4.52]	-1.06 [-3.61, 1.48]	1.71 [-2.02, 5.27]						
L 629	L 637	L Ventral anterior-lateral complex of the th	3.02 ± 1.04	14	4.92 ± 1.32	14	11.96 ± 2.48	12	26.00 ± 8.50	12	3.44 [0.88, 6.06]*	1.73 [-0.86, 4.27]	-1.48 [-5.26, 2.05]	-1.56 [-3.8, 0.56]	-4.38 [-6.56, -2.09]*	-5.12 [-7.53, -2.97]*	2.79 [0.51, 5.13]*	-3.58 [-5.83, -1.25]*	-0.76 [-3.2, 1.68]
R 685	R 637	R Ventral medial nucleus of the thalamus	6.40 ± 2.88	15	6.00 ± 1.37	15	9.16 ± 3.45	12	13.05 ± 3.01	12	1.17 [-1.36, 3.7]	1.58 [-0.91, 4.08]	-1.32 [-4.95, 2.22]						
L 685	L 637	L Ventral medial nucleus of the thalamus	5.75 ± 2.19	14	5.87 ± 1.38	15	14.30 ± 5.40	12	12.47 ± 2.51	12	1.95 [-0.63, 4.53]	1.75 [-0.81, 4.3]	-2.93 [-6.63, 0.68]						
R 709	R 637	R Ventral posterior complex of the thalamu	3.80 ± 0.75	15	5.26 ± 1.23	16	10.82 ± 2.53	12	21.24 ± 4.27	12	0.7 [-1.83, 3.27]	0.02 [-2.48, 2.55]	0.47 [-3.18, 4.05]						
L 709	L 637	L Ventral posterior complex of the thalamu	4.24 ± 0.78	13	4.95 ± 1.07	16	11.39 ± 3.00	12	19.35 ± 3.19	12	1.12 [-1.47, 3.76]	1.23 [-1.34, 3.84]	-0.93 [-4.63, 2.67]						
R 718	R 709	R Ventral posterolateral nucleus of the tha	2.83 ± 0.57	15	5.78 ± 1.58	16	13.50 ± 3.51	12	28.98 ± 6.36	12	0.82 [-1.68, 3.41]	-0.16 [-2.67, 2.33]	1.7 [-2.03, 5.32]						
L 718	L 709	L Ventral posterolateral nucleus of the thal	4.68 ± 1.51	13	6.86 ± 1.64	16	12.00 ± 2.17	12	29.05 ± 4.85	12	1.71 [-0.9, 4.36]	1.21 [-1.37, 3.73]	-0.12 [-3.79, 3.47]						
R 725	R 709	R Ventral posterolateral nucleus of the tha	0.00 ± 0.00	11	3.93 ± 1.98	13	5.49 ± 2.80	8	31.11 ± 11.81	7	3.9 [1.08, 6.77]*	3.8 [1.09, 6.43]*	-0.15 [-4.22, 3.9]	-3.65 [-5.98, -1.39]*	-4.81 [-7.42, -2.29]*	-8.97 [-11.59, -6.27]*	1.17 [-1.32, 3.69]	-5.34 [-7.9, -2.72]*	-4.14 [-7.07, -1.32]*
L 725	L 709	L Ventral posterolateral nucleus of the thal	1.22 ± 1.22	11	5.75 ± 3.38	12	23.15 ± 9.71	10	10.19 ± 4.66	8	4.73 [1.96, 7.54]*	2.12 [-0.59, 4.8]	-4.27 [-8.25, -0.28]*	-1.97 [-4.41, 0.38]	-5.68 [-8.12, -3.08]*	-4.01 [-6.68, -1.43]*	3.7 [1.26, 6.13]*	-2.06 [-4.58, 0.46]	1.65 [-1.14, 4.22]
R 733	R 709	R Ventral posteromedial nucleus of the tha	12.83 ± 2.68	14	14.15 ± 3.52	16	26.58 ± 6.03	12	52.29 ± 11.45	12	0.37 [-2.23, 3.01]	-0.22 [-2.8, 2.34]	0.65 [-3.01, 4.2]						
L 733	L 709	L Ventral posteromedial nucleus of the tha	11.35 ± 1.98	13	11.51 ± 2.65	16	30.21 ± 9.60	12	46.32 ± 7.79	12	1.04 [-1.48, 3.68]	1.07 [-1.5, 3.54]	-0.81 [-4.49, 2.77]						
R 741	R 709	R Ventral posteromedial nucleus of the tha	1.81 ± 1.81	9	4.52 ± 2.25	12	7.90 ± 3.37	10	13.43 ± 4.18	9	3.8 [1, 6.59]*	2.71 [-0.09, 5.52]	-2.51 [-6.51, 1.41]	-2.57 [-4.91, 0]	-4.72 [-7.29, -2.25]*	-5.46 [-7.97, -2.68]*	2.16 [-0.22, 4.62]	-2.89 [-5.43, -0.45]*	-0.7 [-3.28, 1.94]
L 741	L 709	L Ventral posteromedial nucleus of the tha	6.37 ± 2.90	8	5.80 ± 2.60	14	8.67 ± 4.39	10	10.89 ± 3.67	9	-0.66 [-3.54, 2.18]	0.4 [-2.41, 3.16]	1.51 [-2.43, 5.45]						
R 56380	R 637	R Posterior triangular thalamic nucleus	11.86 ± 8.67	9	2.38 ± 1.74	10	12.81 ± 4.87	10	22.75 ± 8.19	7	0.55 [-2.29, 3.37]	-2.01 [-4.9, 0.86]	3.18 [-1, 7.31]						
L 56380	L 637	L Posterior triangular thalamic nucleus	4.14 ± 1.71	9	11.10 ± 5.69	8	10.23 ± 5.49	10	19.08 ± 9.12	7	-0.72 [-3.56, 2.14]	0.48 [-2.52, 3.45]	1.26 [-2.91, 5.35]						
R 406	R 864	R Subparafascicular nucleus	12.59 ± 4.03	11	5.51 ± 2.41	14	28.21 ± 9.99	11	18.92 ± 3.47	8	0.95 [-1.77, 3.64]	-1.98 [-4.67, 0.7]	1.75 [-2.19, 5.55]						
L 406	L 864	L Subparafascicular nucleus	11.22 ± 3.51	11	10.21 ± 3.64	13	8.89 ± 3.13	11	14.14 ± 2.09	8	-0.21 [-2.89, 2.47]	-0.37 [-3.03, 2.31]	1.16 [-2.77, 4.91]						
R 422	R 406	R Subparafascicular nucleus, parvicellular	21.07 ± 9.39	9	8.42 ± 4.36	10	35.70 ± 19.07	9	18.48 ± 9.30	7	-0.06 [-2.95, 2.87]	-2.07 [-4.98, 0.87]	0.76 [-3.52, 4.99]						
L 422	L 406	L Subparafascicular nucleus, parvicellular p	17.85 ± 9.94	9	10.97 ± 5.16	9	9.39 ± 4.65	8	25.45 ± 4.49	7	-0.36 [-3.24, 2.58]	0.38 [-2.51, 3.31]	2.27 [-1.95, 6.44]						
R 1008	R 864	R Geniculate group, dorsal thalamus	5.05 ± 1.82	14	5.70 ± 1.92	16	11.58 ± 3.38	12	14.85 ± 3.57	11	1.59 [-0.97, 4.24]	0.61 [-1.99, 3.14]	-1.46 [-5.23, 2.2]						
L 1008	L 864	L Geniculate group, dorsal thalamus	6.10 ± 3.08	14	4.43 ± 1.32	16	13.85 ± 3.28	12	16.86 ± 3.23	12	1.73 [-0.89, 4.28]	0.55 [-2, 3.01]	-0.79 [-4.37, 2.82]						
R 475	R 1008	R Medial geniculate complex	1.34 ± 0.64	8	0.96 ± 0.64	9	4.29 ± 1.54	10	15.94 ± 4.58	7	1.15 [-1.69, 4.01]	-1.51 [-4.52, 1.45]	4.78 [0.58, 8.9]*	1.66 [-0.98, 4.31]	-2.06 [-4.71, 0.45]	-5.87 [-8.65, -3.04]*	3.73 [1.15, 6.27]*	-7.52 [-10.31, -4.77]*	-3.79 [-6.48, -0.97]*
L 475	L 1008	L Medial geniculate complex	5.35 ± 3.32	8	2.34 ± 0.88	9	6.03 ± 2.30	10	17.27 ± 4.69	7	0.15 [-2.77, 3.03]	0.3 [-2.7, 3.29]	2.79 [-1.47, 6.99]						
R 1072	R 475	R Medial geniculate complex, dorsal part	0.00 ± 0.00	6	4.26 ± 4.26	8	10.30 ± 7.29	9	41.68 ± 13.32	6	2.36 [-0.71, 5.42]	1.65 [-1.52, 4.79]	7.11 [2.71, 11.46]*	-1.49 [-4.27, 1.47]	-3.27 [-6.24, -0.66]*	-12.58 [-15.59, -9.48]*	1.8 [-1.01, 4.3]	-11.09 [-13.99, -8.1]*	-9.28 [-12.17, -6.37]*
L 1072	L 475	L Medial geniculate complex, dorsal part	14.98 ± 12.66	7	3.88 ± 2.67	7	11.77 ± 8.44	8	14.52 ± 6.65	7	-0.4 [-3.42, 2.61]	-0.7 [-3.87, 2.47]	1.73 [-2.66, 6.02]						
R 1079	R 475	R Medial geniculate complex, ventral part	0.00 ± 0.00	6	4.51 ± 3.18	7	7.70 ± 3.49	9	36.96 ± 15.52	6	4.79 [1.66, 7.97]*	3.03 [-0.3, 6.25]	1.04 [-3.53, 5.53]	-2.88 [-5.79, 0.13]	-5.72 [-8.69, -2.97]*	-10.3 [-13.43, -7.24]*	2.84 [0.11, 5.57]*	-7.43 [-10.55, -4.43]*	-4.58 [-7.38, -1.64]*
L 1079	L 475	L Medial geniculate complex, ventral part	21.61 ± 12.70	6	7.03 ± 5.93	7	11.78 ± 6.94	8	40.79 ± 16.25	7	-1.43 [-4.54, 1.67]	-2.43 [-5.65, 0.76]	4.09 [-0.29, 8.44]						
R 1088	R 475	R Medial geniculate complex, medial part	4.76 ± 2.02	8	0.00 ± 0.00	9	9.48 ± 3.92	10	27.83 ± 6.34	7	0.76 [-2.06, 3.63]	-3.37 [-6.25, -0.42]*	5.43 [1.23, 9.49]*	3.54 [1, 6.28]*	-1.68 [-4.17, 1.05]	-4.27 [-7.16, -1.57]*	5.21 [2.57, 7.73]*	-7.79 [-10.51, -5.11]*	-2.59 [-5.28, 0.2]
L 1088	L 475	L Medial geniculate complex, medial part	2.93 ± 2.03	8	4.02 ± 1.93	9	7.21 ± 3.52	10	36.22 ± 7.04	7	1.2 [-1.63, 4.13]	1.58 [-1.31, 4.53]	3.37 [-0.82, 7.5]						
R 170	R 1008	R Dorsal part of the lateral geniculate com	8.40 ± 3.21	14	9.79 ± 3.20	16	21.62 ± 6.26	12	27.31 ± 6.97	11	2.15 [-0.43, 4.8]	1.08 [-1.43, 3.62]	-2.03 [-5.86, 1.6]						
L 170	L 1008	L Dorsal part of the lateral geniculate com	9.03 ± 4.67	14	8.05 ± 2.63	16	26.75 ± 6.24	12	30.01 ± 6.23	12	2.39 [-0.18, 4.98]	1 [-1.53, 3.51]	-1.43 [-5.06, 2.16]						
R 496345	R 170	R Dorsal part of the lateral geniculate com	7.16 ± 4.58	13	23.59 ± 8.53	15	31.79 ± 11.54	12	35.23 ± 10.70	11	1.96 [-0.68, 4.6]	3.3 [0.64, 5.84]*	-1.92 [-5.63, 1.77]	-3.14 [-5.33, -0.95]*	-2.9 [-5.16, -0.56]*	-4.79 [-7.08, -2.33]*	-0.24 [-2.48, 2.03]	-1.64 [-4.01, 0.6]	-1.9 [-4.38, 0.51]
L 496345	L 170	L Dorsal part of the lateral geniculate com	17.45 ± 13.38	14	15.57 ± 6.56	15	37.03 ± 14.93	11	58.73 ± 19.99	11	1.28 [-1.39, 3.86]	1.12 [-1.53, 3.66]	0.08 [-3.63, 3.83]						
R 496345	R 170	R Dorsal part of the lateral geniculate com	21.04 ± 8.53	14	18.07 ± 5.92	16	48.53 ± 18.07	12	60.55 ± 15.11	11	1.99 [-0.61, 4.65]	0.89 [-1.61, 3.41]	-1.83 [-5.56, 1.86]						
L 496345	L 170	L Dorsal part of the lateral geniculate com	19.42 ± 8.71	14	18.44 ± 5.48	16	58.75 ± 15.40	12	68.44 ± 13.65	12	2.32 [-0.25, 4.94]	0.7 [-1.8, 3.23]	-1.08 [-4.71, 2.42]						
R 496345	R 170	R Dorsal part of the lateral geniculate com	16.65 ± 8.15	12	21.86 ± 8.26	15	42.81 ± 14.00	12	64.98 ± 24.98	11	1.42 [-1.19, 4.02]	1.7 [-0.95, 4.3]	-0.88 [-4.61, 2.74]						
L 496345	L 170	L Dorsal part of the lateral geniculate com	18.23 ± 9.86	12	11.84 ± 5.57	16	51.56 ± 15.07	11	48.48 ± 13.97	11	2.08 [-0.62, 4.73]	0 [-2.58, 2.53]	-0.42 [-4.21, 3.18]						
R 856	R 549	R Thalamus, polymodal association cortex	4.33 ± 1.14	15	4.67 ± 0.83	16	5.83 ± 1.34	12	9.64 ± 1.52	12	-0.37 [-2.9, 2.23]	0.47 [-2.04, 2.94]	-0.15 [-3.73, 3.46]						
L 856	L 549	L Thalamus, polymodal association cortex	3.82 ± 0.75	15	4.49 ± 0.83	16	6.55 ± 1.42	12	10.01 ± 1.55	12	-0.03 [-2.59, 2.57]	0.55 [-1.99, 3.08]	-0.36 [-3.99, 3.26]						
R 138	R 856	R Lateral group of the dorsal thalamus	1.91 ± 0.44	14	2.70 ± 0.72	16	6.32 ± 1.77	12	15.60 ± 2.89	12	1.01 [-1.53, 3.64]	0.18 [-2.37, 2.69]	0.66 [-3.04, 4.33]						
L 138	L 856	L Lateral group of the dorsal thalamus	1.53 ± 0.36	14	2.62 ± 0.68	16	6.35 ± 1.46	12	14.76 ± 2.26	12	2.1 [-0.49, 4.68]	1.43 [-1.13, 3.97]	-0.72 [-4.36, 2.87]						
R 218	R 138	R Lateral posterior nucleus of the thalamu	4.77 ± 1.37	14	5.76 ± 1.77	16	12.85 ± 3.02	12	22.53 ± 4.21	12	1.34 [-1.28, 3.88]	0.54 [-2.04, 3.07]	-0.25 [-3.98, 3.36]						
L 218	L 138	L Lateral posterior nucleus of the thalamu	3.64 ± 0.88	14	7.45 ± 2.19	16	14.30 ± 3.64	12	24.49 ± 3.91	12	1.96 [-0.58, 4.54]	0.86 [-1.71, 3.39]	-0.38 [-4.13, 3.15]						
R 1020	R 138	R Posterior complex of the thalamus	2.33 ± 0.69	14	5.69 ± 2.87	16	13.38 ± 4.98	12	40.32 ± 8.78	12	2.49 [-0.1, 5.12]	1.31 [-1.26, 3.82]	-0.28 [-3.94, 3.37]						
L 1020	L 138	L Posterior complex of the thalamus	1.71 ± 0.68	14	3.71 ± 1.03	16	10.88 ± 1.92	12	37.87 ± 6.97	12	3.14 [0.55, 5.72]*	2.04 [-0.53, 4.56]	-0.59 [-4.22, 3.04]	-1.89 [-3.97, 0.27]	-4.08 [-6.3, -1.79]*	-6.02 [-8.33, -3.68]*	2.19 [0.05, 4.52]*	-4.15 [-6.35, -1.86]*	-1.97 [-4.34, 0.51]
R 1029	R 138	R Posterior limiting nucleus of the thalamu	20.44 ± 8.29	10	7.34 ± 3.74	10	12.48 ± 5.15	10	14.38 ± 5.68	8	-0.89 [-3.6, 1.88]	-1.78 [-4.64, 1]	1.86 [-2.18, 5.74]						
L 1029	L 138	L Posterior limiting nucleus of the thalamu	4.61 ± 2.37	11	1.65 ± 1.08	8	16.04 ± 7.65	10	20.63 ± 9.37	7	0.87 [-1.88, 3.64]	-1.14 [-4.06, 1.73]	1.98 [-2.22, 6.03]						
R 325	R 138	R Suprageniculate nucleus	7.16 ± 4.64	6	0.00 ± 0.00	7	0.84 ± 0.84	9	27.18 ± 15.73	6	-2.39 [-5.51, 0.71]	-3.01 [-6.22, 0.21]	5.57 [1.15, 9.98]*	3.16 [0.2, 6.01]*	1.45 [-1.44, 4.22]	-1.63 [-4.68, 1.45]	1.7 [-1.13, 4.43]	-4.79 [-7.8, -1.86]*	-3.08 [-6.07, -0.23]*
L 325	L 138	L Suprageniculate nucleus	11.73 ± 7.45	7	2.90 ± 2.90	6	7.00 ± 5.67	8	12.08 ± 5.03	7	-1.49 [-4.54, 1.58]	-2.32 [-5.58, 0.91]	3.56 [-0.95, 7.97]						
R 560581	R 138	R Ethmoid nucleus of the thalamus	5.04 ± 2.59	13	4.60 ± 1.93	16	8.95 ± 3.43	12	11.13 ± 4.30	12	0.33 [-2.22, 2.95]	0.31 [-2.25, 2.84]	0.04 [-3.61, 3.61]						
L 560581	L 138	L Ethmoid nucleus of the thalamus	4.24 ± 1.68	13	3.64 ± 1.81	16	13.11 ± 7.06	12	15.87 ± 5.39	12	0.38 [-2.22, 3.05]	-0.2 [-2.82, 2.39]	1.41 [-2.35, 5.05]						
R 239	R 856	R Anterior group of the dorsal thalamus	4.14 ± 1.26	15	4.15 ± 1.08	14	4.71 ± 1.02	12	8.40 ± 1.27	12	-0.18 [-2.71, 2.37]	0.19 [-2.35, 2.7]	0.11 [-3.56, 3.71]						
L 239	L 856	L Anterior group of the dorsal thalamus	3.48 ± 0.86	15	2.84 ± 0.52	14	6.35 ± 1.43	12	9.19 ± 1.57	12	1.35 [-1.2, 3.89]	1.22 [-1.38, 3.76]	-1.08 [-4.74, 2.52]						
R 255	R 239	R Anteroventral nucleus of thalamus	11.55 ± 3.89	13	8.49 ± 4.41	10	7.41 ± 2.22	12	10.15 ± 3.53	10	-0.65 [-3.24, 1.93]	-0.59 [-3.35, 2.08]	0.11 [-3.72, 3.84]						
L 255	L 239	L Anteroventral nucleus of thalamus	18.61 ± 6.69	12	17.17 ± 11.25	10	5.72 ± 1.52	12	14.95 ± 9.01	10	-1.31 [-3.91, 1.34]	0.25 [-2.54, 2.97]	-0.48 [-4.42, 3.39]						
R 127	R 239	R Anteromedial nucleus	3.31 ± 1.75	13	2.19 ± 0.82	9	3.75 ± 1.11	12	2.57 ± 1.34	10	0.3 [-2.3, 2.95]	0.22 [-2.51, 2.97]	-2.97 [-6.84, 0.83]						
L 127	L 239	L Anteromedial nucleus	2.65 ± 0.96	13	1.23 ± 0.57	10	2.47 ± 0.79	12	4.01 ± 1.73	10	-1.81 [-4.41, 0.84]	-1.64 [-4.38, 1.11]	2.73 [-1.16, 6.56]						
R 1096	R 127	R Anteromedial nucleus, dorsal part	6.83 ± 4.66	13	4.41 ± 2.37	9	4.20 ± 1.70	12	5.42 ± 3.10	10	-1.1 [-3.75, 1.48]	-0.14 [-2.94, 2.65]	-0.25 [-4.17, 3.51]						
L 1096	L 127	L Anteromedial nucleus, dorsal part	7.06 ± 2.75	13	2.18 ± 1.16	10	6.29 ± 2.66	12	7.36 ± 3.96	10	-2.46 [-5.08, 0.19]	-2.45 [-5.17, 0.24]	2.6 [-1.27, 6.33]						
R 1104	R 127	R Anteromedial nucleus, ventral part	6.14 ± 2.36	13	4.16 ± 2.65	9	12.98 ± 4.40	12	4.68 ± 2.41	10	1.27 [-1.36, 3.9]	-1.23 [-4.03, 1.52]	-2.52 [-6.44, 1.34]						
L 1104	L 127	L Anteromedial nucleus, ventral part	2.77 ± 1.31	13	2.81 ± 1.57	10	2.56 ± 1.35	12	10.62 ± 5.43	9	-1.48 [-4.11, 1.17]	-0.04 [-2.82, 2.63]	2.51 [-1.4, 6.33]						
R 64	R 239	R Anterodorsal nucleus	15.70 ± 8.13	12	11.60 ± 2.75	10	5.27 ± 2.17	11	11.75 ± 5.85	10	-1.59 [-4.35, 1.11]	0.91 [-1.89, 3.68]	-1.69 [-5.57, 2.13]						
L 64	L 239	L Anterodorsal nucleus	11.58 ± 3.18	11	4.07 ± 2.52	10	4.66 ± 1.87	11	13.38 ± 4.30	9	-2.67 [-5.36, 0.05]	-2.58 [-5.42, 0.18]	4.34 [0.36, 8.26]*	2.73 [0.24, 5.17]*	1.74 [-0.57, 4.28]	-0.54 [-3.06, 1.96]	0.98 [-1.37, 3.53]	-3.27 [-5.89, -0.74]*	-2.28 [-4.76, 0.37]
R 1120	R 239	R Interanteromedial nucleus of the thalamu	29.66 ± 14.46	12	18.62 ± 5.17	9	30.89 ± 24.71	10	16.69 ± 9.48	9	-1.98 [-4.68, 0.68]	1.35 [-1.44, 4.12]	-1.21 [-5.18, 2.69]						
L 1120	L 239	L Interanteromedial nucleus of the thalamu	19.96 ± 8.03	11	14.30 ± 7.61	9	29.34 ± 16.90	10	31.85 ± 9.77	9	-1.53 [-4.27, 1.19]	-0.4 [-3.2, 2.38]	1.72 [-2.25, 5.62]						
R 1113	R 239	R Interanterodorsal nucleus of the thalamu	37.85 ± 12.13	12	12.36 ± 8.18	9	23.33 ± 6.38	12	34.43 ± 14.13	10	-1.26 [-3.84, 1.38]	-3.64 [-6.46, -0.83]*	2.76 [-1.11, 6.63]	3.79 [1.34, 6.25]*	0.34 [-1.99, 2.69]	0.69 [-1.84, 3.13]	3.47 [1.01, 5.96]*	-3.1 [-5.66, -0.49]*	0.36 [-2.08, 2.89]
L 1113	L 239	L Interanterodorsal nucleus of the thalamu	26.86 ± 7.53	12	10.11 ± 4.04	10	44.01 ± 13.80	12	23.73 ± 9.49	9	-1.02 [-3.64, 1.58]	-2.26 [-4.96, 0.42]	1.08 [-2.82, 4.93]						
R 155	R 239	R Lateral dorsal nucleus of thalamus	3.97 ± 1.01	15	10.51 ± 4.64	14	11.37 ± 3.04	12	23.01 ± 3.56	12	0.78 [-1.76, 3.36]	0.52 [-2.06, 3.08]	0.5 [-3.22, 4.06]						
L 155	L 239	L Lateral dorsal nucleus of thalamus	3.83 ± 1.44	15	5.77 ± 1.45	14	16.65 ± 4.23	12	22.12 ± 4.55	12	2.08 [-0.49, 4.64]	1.31 [-1.24, 3.83]	-0.66 [-4.38, 3.02]						
R 444	R 856	R Medial group of the dorsal thalamus	5.91 ± 1.46	15	6.57 ± 1.47	15	8.56 ± 2.26	12	12.70 ± 2.41	12	0.02 [-2.54, 2.6]	0.59 [-1.97, 3.07]	-0.38 [-4.02, 3.22]						
L 444	L 856	L Medial group of the dorsal thalamus	5.96 ± 1.29	15	6.53 ± 1.22	15	11.16 ± 2.74	12	13.02 ± 2.40	12	0.02 [-2.53, 2.6]	0.64 [-1.87, 3.11]	-0.77 [-4.41, 2.78]						
R 59	R 444	R Intermediodorsal nucleus of the thalamu	24.04 ± 6.07	14	31.81 ± 6.36	15	40.41 ± 8.20	12	31.86 ± 7.55	12	0.94 [-1.72, 3.54]	0.83 [-1.72, 3.38]	-1.81 [-5.42, 1.83]						
L 59	L 444	L Intermediodorsal nucleus of the thalamu	17.53 ± 4.95	14	28.35 ± 7.05	15	46.41 ± 14.84	12	35.06 ± 8.03	12	1.05 [-1.52, 3.74]	2.13 [-0.49, 4.67]	-2.43 [-6.13, 1.2]						
R 362	R 444	R Mediodorsal nucleus of thalamus	10.70 ± 2.83	15	8.92 ± 2.11	15	14.33 ± 4.81	12	21.80 ± 4.63	12	-0.28 [-2.81, 2.24]	0.23 [-2.35, 2.79]	0.55 [-3.18, 4.12]						
L 362	L 444	L Mediodorsal nucleus of thalamus	12.01 ± 2.64	15	8.94 ± 1.51	14	21.58 ± 5.95	12	23.56 ± 4.89	12	-0.09 [-2.64, 2.48]	0.42 [-2.18, 2.9]	-0.55 [-4.22, 3.03]						
R 366	R 444	R Submedial nucleus of the thalamus	2.94 ± 0.79	14	6.51 ± 1.53	14	17.93 ± 5.81	12	37.27 ± 8.24	11	0.33 [-2.28, 2.93]	1.51 [-1.05, 4.07]	2.11 [-1.61, 5.77]						
L 366	L 444	L Submedial nucleus of the thalamus	4.73 ± 1.47	14	9.34 ± 2.28	14	16.37 ± 5.22	11	33.61 ± 8.17	12	1.7 [-0.94, 4.32]	1.93 [-0.67, 4.47]	-1.55 [-5.26, 2.14]						
R 1077	R 444	R Perireunensis nucleus	30.30 ± 9.24	15	14.38 ± 4.29	14	15.03 ± 5.20	12	28.50 ± 5.11	12	-1.64 [-4.18, 0.94]	-0.05 [-2.66, 2.48]	1.82 [-1.88, 5.46]						
L 1077	L 444	L Perireunensis nucleus	33.24 ± 13.90	15	21.16 ± 6.68	14	20.68 ± 7.71	12	23.80 ± 8.54	12	-1.46 [-4.07, 1.07]	0.51 [-2.11, 3.08]	-0.22 [-3.85, 3.4]						
R 571	R 856	R Midline group of the dorsal thalamus	20.60 ± 5.93	15	19.61 ± 5.68	16	18.55 ± 3.12	12	28.07 ± 4.26	12	-0.68 [-3.2, 1.88]	-0.46 [-2.98, 2]	0.7 [-3, 4.25]						
L 571	L 856	L Midline group of the dorsal thalamus	16.71 ± 3.47	15	20.96 ± 5.75	16	22.16 ± 3.66	12	29.80 ± 5.67	12	0.38 [-2.15, 2.97]	0.12 [-2.44, 2.67]	-0.15 [-3.82, 3.49]						
R 149	R 571	R Paraventricular nucleus of the thalamus	80.87 ± 18.53	15	72.73 ± 21.61	16	81.97 ± 14.86	12	98.93 ± 16.90	12	-0.49 [-3.05, 2.04]	-0.64 [-3.13, 1.86]	0.19 [-3.38, 3.75]						

L 149	L 571	L Paraventricular nucleus of the thalamus	70.48 ± 15.30	15	83.54 ± 23.49	16	104.84 ± 15.81	12	103.12 ± 20.20	12	0.47 [-2.05, 3.01]	-0.31 [-2.81, 2.16]	-0.11 [-3.7, 3.45]						
R 15	R 571	R Parataenial nucleus	64.81 ± 26.99	10	35.41 ± 22.71	6	48.57 ± 17.58	7	24.53 ± 18.02	6	-0.76 [-3.72, 2.24]	-1.43 [-4.52, 1.62]	-1.4 [-5.88, 2.98]						
L 15	L 571	L Parataenial nucleus	32.56 ± 9.97	10	43.41 ± 28.05	7	24.20 ± 12.26	8	34.10 ± 22.72	7	-1.94 [-4.81, 0.97]	0.17 [-2.86, 3.18]	-0.61 [-5.03, 3.57]						
R 181	R 571	R Nucleus of reuniens	14.67 ± 6.28	15	20.38 ± 5.75	14	12.19 ± 3.48	12	29.37 ± 7.72	12	-0.46 [-2.99, 2.14]	0.41 [-2.13, 2.98]	0.47 [-3.3, 4.03]						
L 181	L 571	L Nucleus of reuniens	12.58 ± 3.94	15	17.13 ± 4.44	14	17.01 ± 5.00	12	27.06 ± 9.05	12	0.64 [-1.87, 3.25]	0.4 [-2.14, 2.95]	-0.26 [-3.95, 3.39]						
R 560581	R 571	R Xiphoid thalamic nucleus	27.49 ± 7.45	15	31.08 ± 7.51	14	22.39 ± 6.77	12	47.78 ± 12.73	12	-0.86 [-3.43, 1.68]	1.3 [-1.25, 3.86]	-0.47 [-4.12, 3.16]						
L 560581	L 571	L Xiphoid thalamic nucleus	18.05 ± 5.14	15	44.65 ± 14.15	14	28.33 ± 8.83	12	50.01 ± 14.14	12	-0.52 [-3.04, 2.08]	1.79 [-0.73, 4.27]	-1.17 [-4.83, 2.44]						
R 51	R 856	R Intralaminar nuclei of the dorsal thalamu	7.28 ± 1.84	15	6.51 ± 1.45	16	8.27 ± 2.06	12	14.10 ± 2.70	12	0.13 [-2.41, 2.7]	0.47 [-2.08, 3]	-0.31 [-4.02, 3.21]						
L 51	L 856	L Intralaminar nuclei of the dorsal thalamu	6.72 ± 1.51	15	6.54 ± 2.17	16	9.63 ± 2.55	12	14.24 ± 2.25	12	0.18 [-2.35, 2.75]	-0.59 [-3.07, 1.9]	0.82 [-2.9, 4.42]						
R 189	R 51	R Rhomboid nucleus	17.21 ± 5.97	15	21.71 ± 8.29	14	22.79 ± 8.31	12	40.50 ± 13.81	12	-0.01 [-2.59, 2.59]	0.68 [-1.92, 3.21]	-0.09 [-3.72, 3.48]						
L 189	L 51	L Rhomboid nucleus	15.23 ± 4.47	15	14.24 ± 8.74	14	28.18 ± 9.49	12	40.04 ± 10.79	12	0 [-2.53, 2.58]	-1.22 [-3.77, 1.28]	1.75 [-1.97, 5.37]						
R 599	R 51	R Central medial nucleus of the thalamus	32.59 ± 7.54	15	27.98 ± 6.71	15	32.01 ± 10.18	12	41.33 ± 10.64	12	-0.14 [-2.69, 2.42]	0.22 [-2.43, 2.72]	-0.83 [-4.48, 2.79]						
L 599	L 51	L Central medial nucleus of the thalamus	26.49 ± 7.30	15	24.98 ± 8.35	15	40.46 ± 14.42	12	29.16 ± 5.45	12	0.22 [-2.35, 2.8]	-0.45 [-3, 2.08]	0.42 [-3.27, 4.01]						
R 907	R 51	R Paracentral nucleus	4.62 ± 2.02	14	8.84 ± 4.18	14	7.58 ± 2.96	12	32.53 ± 7.10	12	-0.55 [-3.15, 2.03]	0.82 [-1.76, 3.38]	2.81 [-0.88, 6.47]						
L 907	L 51	L Paracentral nucleus	11.24 ± 4.29	14	8.11 ± 5.68	14	12.83 ± 3.18	12	43.34 ± 14.09	12	0.06 [-2.46, 2.68]	-1.08 [-3.7, 1.53]	3.63 [0.02, 7.25]*	1.23 [-0.97, 3.39]	-0.97 [-3.26, 1.38]	-4.06 [-6.35, -1.71]*	2.22 [-0.00, 4.52]	-5.29 [-7.63, -3.07]*	-3.06 [-5.41, -0.59]*
R 575	R 51	R Central lateral nucleus of the thalamus	10.24 ± 4.95	14	14.48 ± 4.75	15	8.33 ± 1.53	12	21.85 ± 4.31	12	1.32 [-1.25, 3.92]	1.36 [-1.23, 3.92]	0.08 [-3.59, 3.66]						
L 575	L 51	L Central lateral nucleus of the thalamus	8.98 ± 2.14	14	15.37 ± 5.82	15	12.86 ± 3.90	12	23.07 ± 5.08	12	-0.42 [-3.01, 2.19]	-0.11 [-2.63, 2.45]	1.97 [-1.69, 5.54]						
R 930	R 51	R Parafascicular nucleus	6.97 ± 3.47	11	5.36 ± 2.22	11	19.65 ± 6.76	10	15.05 ± 6.17	7	3.05 [0.29, 5.84]*	1.86 [-0.93, 4.53]	-3.93 [-7.97, -0.06]*	-1.7 [-4.07, 0.74]	-3.99 [-6.47, -1.55]*	-2.43 [-5.07, 0.29]	2.27 [-0.12, 4.85]	-0.72 [-3.29, 2.02]	1.54 [-1.34, 4.21]
L 930	L 51	L Parafascicular nucleus	7.84 ± 3.03	10	5.04 ± 1.86	11	15.59 ± 5.87	9	21.45 ± 6.41	7	2.19 [-0.64, 5.02]	-0.1 [-2.88, 2.66]	-1.03 [-5.17, 3.04]						
R 262	R 856	R Reticular nucleus of the thalamus	2.46 ± 0.56	15	7.98 ± 1.89	15	10.40 ± 3.75	12	24.72 ± 5.19	12	0.44 [-2.12, 3.01]	0.77 [-1.76, 3.29]	0.46 [-3.25, 4]						
L 262	L 856	L Reticular nucleus of the thalamus	2.86 ± 0.88	15	6.17 ± 1.88	15	12.59 ± 4.08	12	24.32 ± 4.81	12	1.7 [-0.86, 4.27]	1.27 [-1.28, 3.77]	-0.9 [-4.57, 2.62]						
R 1014	R 856	R Geniculate group, ventral thalamus	22.45 ± 9.08	13	18.61 ± 4.93	16	29.20 ± 7.39	12	21.30 ± 6.72	11	0.96 [-1.62, 3.65]	0.36 [-2.22, 2.85]	-1.94 [-5.62, 1.69]						
L 1014	L 856	L Geniculate group, ventral thalamus	17.71 ± 4.99	14	15.31 ± 4.82	16	24.37 ± 7.00	12	27.64 ± 7.49	12	1.18 [-1.39, 3.71]	0.04 [-2.56, 2.59]	-1.15 [-4.87, 2.51]						
R 27	R 1014	R Intergeniculate leaflet of the lateral genic	60.84 ± 24.16	9	43.19 ± 15.11	13	109.07 ± 30.72	10	61.17 ± 23.29	8	1.32 [-1.6, 4.12]	0.05 [-2.8, 2.89]	-1.68 [-5.68, 2.31]						
L 27	L 1014	L Intergeniculate leaflet of the lateral genic	45.24 ± 18.11	12	43.99 ± 17.84	11	85.14 ± 26.39	10	63.76 ± 28.60	8	2.37 [-0.33, 5.1]	0.44 [-2.3, 3.16]	-3.13 [-7.12, 0.82]						
R 178	R 1014	R Ventral part of the lateral geniculate con	47.75 ± 20.66	13	40.05 ± 11.19	16	57.04 ± 15.39	12	45.07 ± 14.45	11	0.82 [-1.72, 3.41]	0.37 [-2.21, 2.9]	-1.8 [-5.49, 1.84]						
L 178	L 1014	L Ventral part of the lateral geniculate com	37.14 ± 10.87	14	34.44 ± 10.85	16	49.97 ± 14.77	12	59.16 ± 15.34	12	1.11 [-1.49, 3.74]	0.08 [-2.46, 2.65]	-1.09 [-4.88, 2.52]						
L 321	L 1014	L Subgeniculate nucleus	0.00 ± 0.00	8	2.17 ± 2.17	11	3.08 ± 3.08	10	0.00 ± 0.00	7	0.51 [-2.38, 3.43]	0.93 [-1.97, 3.82]	-2.91 [-7.15, 1.15]						
R 958	R 856	R Epithalamus	8.47 ± 1.63	15	14.20 ± 3.32	16	7.78 ± 3.47	12	14.43 ± 2.40	12	-2.27 [-4.81, 0.3]	0.49 [-1.98, 2.99]	1.46 [-2.17, 4.98]						
L 958	L 856	L Epithalamus	6.10 ± 1.58	15	13.65 ± 3.24	16	7.64 ± 1.84	12	12.98 ± 1.92	12	-0.28 [-2.81, 2.34]	1.18 [-1.28, 3.66]	-0.71 [-4.38, 2.83]						
R 483	R 958	R Medial habenula	21.01 ± 5.46	15	36.92 ± 9.78	16	13.66 ± 4.30	12	41.79 ± 8.02	12	-1.94 [-4.54, 0.6]	1.4 [-1.15, 3.91]	1.21 [-2.42, 4.8]						
L 483	L 958	L Medial habenula	15.09 ± 5.40	15	37.64 ± 9.04	16	18.42 ± 4.55	12	39.76 ± 6.31	12	1.23 [-1.27, 3.78]	2.57 [0.05, 5.08]*	-1.91 [-5.57, 1.64]	-2.42 [-4.56, -0.34]*	-2.16 [-4.4, -0.01]*	-3.32 [-5.54, -1.03]*	-0.25 [-2.51, 1.97]	-0.92 [-3.12, 1.29]	-1.18 [-3.56, 1.18]
R 186	R 958	R Lateral habenula	11.89 ± 3.02	15	17.42 ± 5.83	15	17.73 ± 11.75	12	15.47 ± 4.94	12	-1.6 [-4.21, 0.92]	0.29 [-2.3, 2.8]	1.21 [-2.42, 4.85]						
L 186	L 958	L Lateral habenula	8.92 ± 2.50	15	12.63 ± 4.44	15	11.94 ± 3.48	12	12.31 ± 2.77	12	-0.13 [-2.67, 2.48]	0.33 [-2.22, 2.84]	-0.54 [-4.26, 3.03]						
R 1097	R 1129	R Hypothalamus	9.67 ± 2.14	14	10.14 ± 2.00	15	11.38 ± 2.35	12	13.66 ± 1.86	12	-0.11 [-2.66, 2.42]	-0.07 [-2.44, 2.33]	-0.19 [-3.68, 3.24]						
L 1097	L 1129	L Hypothalamus	8.57 ± 1.57	14	11.14 ± 2.28	14	11.64 ± 2.17	12	13.68 ± 1.95	12	0.08 [-2.5, 2.61]	0.13 [-2.29, 2.57]	-0.39 [-3.9, 3.1]						
R 157	R 1097	R Periventricular zone	16.58 ± 5.26	14	21.85 ± 6.78	15	23.54 ± 5.66	12	31.46 ± 5.42	12	0.14 [-2.4, 2.67]	0.24 [-2.14, 2.64]	-0.25 [-3.68, 3.17]						
L 157	L 1097	L Periventricular zone	14.32 ± 3.24	15	27.32 ± 7.97	15	26.68 ± 5.89	12	29.95 ± 5.43	12	0.53 [-1.98, 3.05]	0.78 [-1.58, 3.17]	-1.03 [-4.5, 2.42]						
R 390	R 157	R Supraoptic nucleus	83.65 ± 36.42	10	72.34 ± 21.83	7	79.37 ± 36.24	10	17.92 ± 9.22	8	-0.15 [-2.89, 2.61]	-0.44 [-3.35, 2.42]	-0.74 [-4.74, 3.27]						
L 390	L 157	L Supraoptic nucleus	39.56 ± 19.55	9	73.99 ± 35.32	6	69.59 ± 32.01	9	24.75 ± 11.90	7	0.68 [-2.16, 3.57]	0.43 [-2.64, 3.48]	-2.1 [-6.34, 2.06]						
R 38	R 157	R Paraventricular hypothalamic nucleus	61.56 ± 22.81	13	140.29 ± 32.59	9	73.92 ± 19.24	11	162.74 ± 30.03	10	-0.04 [-2.65, 2.54]	0.99 [-1.66, 3.63]	-0.06 [-3.73, 3.66]						
L 38	L 157	L Paraventricular hypothalamic nucleus	64.58 ± 16.26	13	146.01 ± 32.11	9	114.74 ± 26.80	11	143.40 ± 20.36	10	0.54 [-2.03, 3.1]	0.98 [-1.67, 3.59]	-1.05 [-4.73, 2.68]						
R 30	R 157	R Periventricular hypothalamic nucleus, an	9.16 ± 4.68	8	93.78 ± 54.91	6	58.19 ± 28.05	7	67.79 ± 21.76	8	5.29 [2.23, 8.29]*	8.72 [5.63, 11.8]*	-8.41 [-12.6, -4.15]*	-8.9 [-11.6, -6.09]*	-5.62 [-8.26, -2.96]*	-6.49 [-9.06, -3.93]*	-3.31 [-6.26, -0.53]*	2.42 [-0.17, 5.38]	-0.88 [-3.53, 1.77]
L 30	L 157	L Periventricular hypothalamic nucleus, ant	6.15 ± 6.12	8	123.40 ± 50.10	6	32.05 ± 11.41	7	74.64 ± 24.67	8	4.15 [1.12, 7.15]*	18.56 [15.51, 21.62]*	-12.37 [-16.62, -8.12]*	-18.73 [-21.57, -16.07]*	-4.49 [-7.2, -1.92]*	-11.23 [-13.84, -8.69]*	-14.27 [-17.12, -11.4]*	7.51 [4.71, 10.23]*	-6.75 [-9.42, -4.11]*
R 118	R 157	R Periventricular hypothalamic nucleus, int	16.91 ± 8.68	13	24.28 ± 8.43	15	16.23 ± 6.61	12	37.83 ± 10.30	12	-0.27 [-2.83, 2.32]	0.41 [-2, 2.85]	0.38 [-3.08, 3.85]						
L 118	L 157	L Periventricular hypothalamic nucleus, inte	13.61 ± 6.53	13	30.71 ± 12.87	15	21.70 ± 8.08	12	40.78 ± 12.21	12	0.38 [-2.22, 2.9]	1.25 [-1.18, 3.69]	-0.33 [-3.86, 3.08]						
R 223	R 157	R Arcuate hypothalamic nucleus	30.70 ± 9.90	13	29.02 ± 11.45	14	31.36 ± 11.41	11	30.42 ± 8.68	11	-0.2 [-2.78, 2.41]	-0.01 [-2.49, 2.48]	-0.49 [-4.05, 3.05]						
L 223	L 157	L Arcuate hypothalamic nucleus	27.88 ± 10.08	13	48.79 ± 15.14	15	20.26 ± 3.18	11	28.59 ± 7.21	11	-0.48 [-3.1, 2.09]	0.74 [-1.73, 3.19]	-0.9 [-4.43, 2.61]						
R 141	R 1097	R Periventricular region	13.47 ± 2.79	15	17.95 ± 3.13	16	23.72 ± 4.85	12	28.61 ± 4.24	12	0.44 [-2.05, 2.9]	0.15 [-2.24, 2.56]	-0.27 [-3.67, 3.23]						
L 141	L 1097	L Periventricular region	12.68 ± 2.64	14	15.90 ± 4.74	16	23.57 ± 7.24	12	26.53 ± 4.53	12	0.58 [-1.98, 3.09]	0.11 [-2.29, 2.53]	-0.37 [-3.82, 3.08]						
R 830	R 141	R Dorsomedial nucleus of the hypothalamu	38.31 ± 9.00	12	39.45 ± 11.08	14	50.79 ± 11.53	11	69.68 ± 14.14	11	0.07 [-2.58, 2.66]	0.02 [-2.48, 2.5]	0.01 [-3.59, 3.64]						
L 830	L 141	L Dorsomedial nucleus of the hypothalamu	26.31 ± 9.22	12	34.14 ± 12.81	14	57.21 ± 17.63	11	58.78 ± 11.62	11	0.93 [-1.7, 3.55]	0.29 [-2.19, 2.76]	-0.69 [-4.25, 2.83]						
R 126	R 141	R Periventricular hypothalamic nucleus, po	8.07 ± 3.99	9	19.93 ± 7.10	10	32.22 ± 9.79	7	27.37 ± 11.16	8	2.65 [-0.31, 5.64]	1.04 [-1.7, 3.77]	-1.95 [-5.95, 2.03]						
L 126	L 141	L Periventricular hypothalamic nucleus, pos	9.65 ± 3.33	9	7.91 ± 2.83	10	27.75 ± 10.73	7	30.91 ± 9.94	8	1.52 [-1.45, 4.5]	-0.62 [-3.4, 2.15]	0.64 [-3.42, 4.62]						
R 347	R 141	R Subparaventricular zone	18.00 ± 7.27	13	59.50 ± 18.50	9	51.13 ± 15.84	11	85.62 ± 22.13	10	1.61 [-0.97, 4.21]	2.02 [-0.63, 4.71]	-0.62 [-4.28, 3.05]						
L 347	L 141	L Subparaventricular zone	28.63 ± 12.54	13	61.05 ± 18.12	9	61.93 ± 25.98	10	67.29 ± 24.74	9	0.83 [-1.82, 3.48]	0.86 [-1.86, 3.52]	-1.07 [-4.89, 2.69]						
R 286	R 141	R Suprachiasmatic nucleus	37.05 ± 12.49	10	39.65 ± 12.95	6	132.14 ± 79.88	8	51.64 ± 37.11	7	2.52 [-0.34, 5.34]	-0.37 [-3.33, 2.57]	-2.58 [-6.81, 1.59]						
L 286	L 141	L Suprachiasmatic nucleus	27.10 ± 11.76	9	32.98 ± 16.34	6	127.07 ± 66.33	8	56.97 ± 40.23	7	3.62 [0.71, 6.54]*	-0.23 [-3.26, 2.81]	-3.13 [-7.35, 1.11]	0.03 [-2.66, 2.7]	-3.93 [-6.44, -1.45]*	-1.15 [-3.72, 1.48]	3.98 [1.28, 6.78]*	-1.19 [-4.09, 1.53]	2.78 [0.21, 5.6]*
R 467	R 1097	R Hypothalamic medial zone	16.01 ± 3.87	15	15.95 ± 3.57	16	16.12 ± 3.26	12	17.82 ± 2.60	12	-0.33 [-2.83, 2.17]	-0.19 [-2.53, 2.18]	-0.19 [-3.58, 3.25]						
L 467	L 1097	L Hypothalamic medial zone	13.95 ± 3.08	15	18.37 ± 4.04	16	15.77 ± 3.19	12	19.27 ± 2.85	12	-0.2 [-2.74, 2.3]	0.14 [-2.21, 2.54]	-0.37 [-3.77, 3.1]						
R 88	R 467	R Anterior hypothalamic nucleus	26.22 ± 5.25	15	42.47 ± 11.33	12	41.77 ± 14.50	12	41.18 ± 6.93	10	0.27 [-2.24, 2.78]	0.42 [-2.02, 2.91]	-0.91 [-4.48, 2.62]						
L 88	L 467	L Anterior hypothalamic nucleus	29.34 ± 6.99	14	47.55 ± 12.63	12	44.18 ± 11.01	12	37.89 ± 8.89	11	0.23 [-2.31, 2.73]	0.48 [-2.02, 2.96]	-1.09 [-4.59, 2.45]						
R 331	R 467	R Mammillary body	11.81 ± 4.85	9	7.57 ± 2.55	13	7.98 ± 2.58	11	9.47 ± 1.37	7	-0.5 [-3.34, 2.27]	-0.64 [-3.36, 2.05]	0.31 [-3.56, 4.25]						
L 331	L 467	L Mammillary body	7.43 ± 1.73	10	7.76 ± 2.41	12	5.51 ± 1.33	11	11.43 ± 1.79	8	-0.33 [-3.05, 2.36]	-0.22 [-2.86, 2.42]	0.51 [-3.27, 4.29]						
R 210	R 331	R Lateral mammillary nucleus	1.98 ± 1.98	6	23.78 ± 21.68	8	11.70 ± 6.28	9	10.12 ± 7.19	7	4.56 [1.53, 7.64]*	10.48 [7.37, 13.57]*	-11.59 [-15.81, -7.37]*	-10.66 [-13.47, -7.95]*	-4.88 [-7.58, -2.2]*	-4.33 [-7.25, -1.56]*	-5.77 [-8.28, -3.21]*	6.32 [3.63, 8.98]*	0.55 [-2.06, 3.16]
L 210	L 331	L Lateral mammillary nucleus	10.63 ± 10.63	7	11.75 ± 6.45	9	10.91 ± 9.01	8	19.10 ± 5.25	8	-0.3 [-3.31, 2.69]	-0.34 [-3.27, 2.57]	0.7 [-3.34, 4.81]						
R 491	R 331	R Medial mammillary nucleus	29.37 ± 22.66	7	12.32 ± 7.14	10	4.77 ± 1.92	8	10.85 ± 2.79	7	-1.16 [-4.23, 1.84]	-1.06 [-3.99, 1.8]	0.88 [-3.27, 5.06]						
L 491	L 331	L Medial mammillary nucleus	6.62 ± 2.72	7	10.61 ± 4.53	9	4.45 ± 1.32	8	12.54 ± 2.57	7	-0.65 [-3.7, 2.35]	0.15 [-2.78, 3.03]	0.7 [-3.46, 4.81]						
R 732	R 491	R Medial mammillary nucleus, median part	91.09 ± 69.77	7	11.61 ± 6.72	9	5.98 ± 4.30	7	15.38 ± 4.99	7	-1.2 [-4.31, 1.88]	-1.33 [-4.33, 1.6]	1.02 [-3.21, 5.2]						
L 732	L 491	L Medial mammillary nucleus, median part	24.04 ± 9.30	7	11.33 ± 5.68	9	11.22 ± 7.87	7	17.87 ± 9.93	7	-0.82 [-3.9, 2.24]	-0.99 [-3.9, 1.94]	0.83 [-3.37, 5.06]						
R 606826	R 491	R Medial mammillary nucleus, lateral part	52.94 ± 45.72	7	28.16 ± 15.74	10	11.67 ± 3.63	8	13.29 ± 5.65	7	-1.1 [-4.12, 1.91]	-0.94 [-3.8, 1.92]	0.59 [-3.51, 4.67]						
L 606826	L 491	L Medial mammillary nucleus, lateral part	9.39 ± 5.74	7	32.56 ± 13.80	9	5.34 ± 2.24	8	21.18 ± 7.23	7	-0.76 [-3.8, 2.25]	2.02 [-0.92, 4.95]	-0.69 [-4.91, 3.52]						
R 606826	R 491	R Medial mammillary nucleus, medial part	10.02 ± 6.87	7	6.95 ± 6.12	10	4.93 ± 3.23	8	35.54 ± 11.13	6	-0.82 [-3.8, 2.19]	-0.78 [-3.64, 2.11]	3.38 [-0.83, 7.62]						
L 606826	L 491	L Medial mammillary nucleus, medial part	10.68 ± 4.36	7	11.94 ± 9.02	9	2.83 ± 2.11	8	50.26 ± 6.94	6	-1.05 [-4.07, 1.99]	-0.33 [-3.26, 2.6]	4.3 [0.12, 8.49]*	0.13 [-2.47, 2.69]	0.74 [-1.85, 3.42]	-3.81 [-6.63, -0.93]*	-0.59 [-3.19, 1.95]	-3.94 [-6.61, -1.2]*	-4.53 [-7.37, -1.8]*
R 525	R 331	R Supramammillary nucleus	29.33 ± 10.20	8	23.01 ± 7.44	10	34.60 ± 12.25	10	41.62 ± 6.09	7	0 [-2.87, 2.85]	-0.59 [-3.49, 2.2]	0.41 [-3.58, 4.47]						
L 525	L 331	L Supramammillary nucleus	31.64 ± 9.01	9	25.51 ± 8.41	10	30.39 ± 9.72	10	56.99 ± 17.11	8	-0.23 [-3.08, 2.57]	-0.6 [-3.37, 2.16]	0.96 [-2.94, 4.87]						
R 557	R 331	R Tuberomammillary nucleus	8.77 ± 3.86	8	13.58 ± 4.28	12	15.27 ± 5.85	9	12.36 ± 4.08	6	0.54 [-2.28, 3.42]	0.17 [-2.56, 2.91]	-1.03 [-5.16, 2.91]						
L 557	L 331	L Tuberomammillary nucleus	6.55 ± 2.91	10	8.52 ± 3.29	10	7.24 ± 2.01	9	3.66 ± 3.05	7	-0.01 [-2.84, 2.76]	-0.21 [-2.91, 2.51]	-0.86 [-4.78, 3.09]						
L 1126	L 557	L Tuberomammillary nucleus, dorsal part	19.79 ± 14.23	8	37.19 ± 16.34	8	31.94 ± 11.87	6	10.26 ± 10.26	6	0.07 [-3.08, 3.22]	0.14 [-2.76, 3.09]	-1.6 [-5.92, 2.77]						
R 1	R 557	R Tuberomammillary nucleus, ventral part	17.91 ± 8.58	7	23.32 ± 9.49	10	16.18 ± 7.19	9	30.73 ± 12.62	6	-0.22 [-3.2, 2.78]	0 [-2.97, 2.93]	0.28 [-3.87, 4.47]						
L 1	L 557	L Tuberomammillary nucleus, ventral part	11.93 ± 4.90	10	8.34 ± 5.76	8	8.79 ± 2.96	9	1.77 ± 1.77	7	-0.37 [-3.17, 2.37]	-0.4 [-3.2, 2.38]	-0.72 [-4.76, 3.27]						
R 980	R 467	R Dorsal premammillary nucleus	64.21 ± 26.94	7	33.02 ± 13.58	8	24.54 ± 7.59	7	34.63 ± 10.17	6	-0.85 [-3.97, 2.22]	-1.12 [-4.11, 1.91]	0.89 [-3.43, 5.15]						
L 980	L 467	L Dorsal premammillary nucleus	43.19 ± 13.06	10	34.97 ± 16.02	9	26.05 ± 12.81	7	30.63 ± 5.05	8	-0.44 [-3.4, 2.52]	-0.64 [-3.4, 2.12]	0.25 [-3.82, 4.26]						
R 1004	R 467	R Ventral premammillary nucleus	28.38 ± 12.38	8	32.63 ± 10.52	11	22.40 ± 8.21	9	39.38 ± 12.17	7	-0.41 [-3.34, 2.48]	-0.37 [-3.14, 2.4]	0.41 [-3.54, 4.41]						
L 1004	L 467	L Ventral premammillary nucleus	26.98 ± 11.20	10	53.74 ± 19.02	10	32.81 ± 8.63	10	34.54 ± 9.46	8	0.2 [-2.55, 2.89]	0.5 [-2.16, 3.22]	-0.98 [-4.81, 2.87]						
R 63	R 467	R Paraventricular hypothalamic nucleus, de	68.25 ± 16.55	13	40.81 ± 7.64	12	78.80 ± 19.75	9	63.70 ± 8.70	11	-0.19 [-2.9, 2.45]	-0.48 [-2.98, 2.04]	-0.16 [-3.78, 3.54]						
L 63	L 467	L Paraventricular hypothalamic nucleus, de	58.38 ± 9.90	12	63.71 ± 13.47	12	110.43 ± 30.13	9	51.19 ± 11.03	10	0.62 [-2.12, 3.32]	0.1 [-2.45, 2.63]	-1.44 [-5.13, 2.29]						
R 693	R 467	R Ventromedial hypothalamic nucleus	17.70 ± 3.93	14	23.87 ± 6.12	13	17.78 ± 7.02	12	45.46 ± 8.38	11	-0.27 [-2.81, 2.28]	0.6 [-1.87, 3.07]	0.51 [-3.02, 4.03]						
L 693	L 467	L Ventromedial hypothalamic nucleus	20.90 ± 4.19	14	28.53 ± 9.54	13	16.73 ± 4.27	11	48.49 ± 14.74	11	-0.47 [-3.05, 2.12]	0.61 [-1.87, 3.11]	0.46 [-3.09, 4.02]						
R 946	R 467	R Posterior hypothalamic nucleus	57.59 ± 15.87	14	47.73 ± 12.78	15	66.80 ± 15.09	11	45.16 ± 8.84	9	0.03 [-2.58, 2.59]	-0.25 [-2.66, 2.19]	-0.77 [-4.32, 2.8]						
L 946	L 467	L Posterior hypothalamic nucleus	48.44 ± 13.08	14	49.74 ± 11.82	15	62.74 ± 17.50	11	43.28 ± 7.54	9	0.18 [-2.42, 2.73]	-0.07 [-2.44, 2.37]	-1 [-4.55, 2.54]						
R 290	R 1097	R Hypothalamic lateral zone	8.60 ± 1.98	15	9.30 ± 2.01	16	9.94 ± 2.26	12	13.04 ± 1.91	12	-0.17 [-2.66, 2.31]	-0.1 [-2.48, 2.27]	-0.04 [-3.47, 3.38]						
L 290	L 1097	L Hypothalamic lateral zone	6.93 ± 1.31	15	9.35 ± 1.86	16	10.27 ± 1.86	12	12.79 ± 1.97	12	0.16 [-2.36, 2.64]	0.16 [-2.18, 2.5]	-0.29 [-3.71, 3.12]						
R 194	R 290	R Lateral hypothalamic area	19.30 ± 4.49	15	24.86 ± 6.70	16	23.45 ± 5.60	10	23.41 ± 3.79	12	-0.24 [-2.81, 2.3]	0.11 [-2.24, 2.48]	-0.47 [-3.9, 3.06]						
L 194	L 290	L Lateral hypothalamic area	16.10 ± 3.25	15	19.93 ± 5.00	16	25.93 ± 5.40	11	25.47 ± 5.20	12	0.25 [-2.27, 2.79]	0.06 [-2.34, 2.44]	-0.56 [-4.07, 2.91]						
R 364	R 290	R Parasubthalamic nucleus	31.29 ± 8.07	12	51.81 ± 12.95	13	40.23 ± 12.24	11	85.00 ± 15.14	8	0.12 [-2.5, 2.71]	0.41 [-2.12, 2.9]	0.54 [-3.13, 4.28]						
L 364	L 290	L Parasubthalamic nucleus	26.34 ± 10.25	12	55.39 ± 19.23	13	26.40 ± 6.64	11	63.91 ± 12.08	9	-0.11 [-2.73, 2.45]	0.9 [-1.62, 3.41]	-0.13 [-3.77, 3.57]						
R 576073	R 290	R Perifornical nucleus	24.78 ± 5.39	15	23.21 ± 6.17	14	35.65 ± 12.36	12	31.24 ± 7.87	11	0.12 [-2.41, 2.61]	-0.12 [-2.56, 2.29]	-0.62 [-4.1, 2.92]						
L 576073	L 290	L Perifornical nucleus	12.82 ± 3.93	15	32.87 ± 12.64	14	32.68 ± 7.02	12	47.44 ± 9.73	10	1.23 [-1.3, 3.73]	1.5 [-0.94, 3.96]	-0.82 [-4.41, 2.75]						
L 173	L 290	L Retrochiasmatic area	5.61 ± 3.08	12	16.88 ± 12.58	7	9.31 ± 3.49	8	12.81 ± 8.15	7	0.28 [-2.51, 3.04]	1.34 [-1.47, 4.16]	-1.41 [-5.47, 2.64]						
R 470	R 290	R Subthalamic nucleus	5.86 ± 2.30	12	6.59 ± 3.03	13	11.35 ± 6.63	11	43.86 ± 23.55	9	0.68 [-1.99, 3.31]	0.05 [-2.5, 2.6]	4.97 [1.29, 8.61]*	-0.23 [-2.36, 1.84]	-0.99 [-3.19, 1.21]	-6.58 [-8.91, -4.3]*	0.76 [-1.41, 2.85]	-6.35 [-8.51, -3.98]*	-5.57 [-7.94, -3.2]*
L 470	L 290	L Subthalamic nucleus	9.24 ± 3.82	10	6.24 ± 2.37	16	7.13 ± 2.98	11	24.28 ± 11.33	11	-0.48 [-3.17, 2.2]	-0.44 [-2.96, 2.1]	1.83 [-1.76, 5.44]						
R 614	R 290	R Tuberal nucleus	17.77 ± 5.06	14	16.74 ± 3.76	14	18.02 ± 4.43	11	36.96 ± 7.86	11	-0.26 [-2.8, 2.3]	-0.06 [-2.49, 2.36]	0.66 [-2.86, 4.21]						
L 614	L 290	L Tuberal nucleus	9.68 ± 2.13	14	29.43 ± 8.89	14	24.78 ± 5.34	11	29.23 ± 6.80	11	1.29 [-1.26, 3.83]	2.04 [-0.37, 4.47]	-2.03 [-5.56, 1.5]						
R 797	R 290	R Zona incerta	14.20 ± 3.56	15	14.92 ± 4.15	16	17.70 ± 3.68	12	21.95 ± 3.63	12	-0.08 [-2.59, 2.42]	-0.13 [-2.49, 2.24]	-0.06 [-3.52, 3.36]						
L 797	L 290	L Zona incerta	12.57 ± 2.36	15	10.50 ± 3.03	16	16.06 ± 3.43	12	19.38 ± 2.99	12	-0.05 [-2.59, 2.46]	-0.35 [-2.71, 2.02]	0.13 [-3.29, 3.54]						
R 804	R 797	R Fields of Forel	19.98 ± 9.38	10	18.41 ± 8.89	11	18.24 ± 8.34	9	24.85 ± 7.61	8	-0.12 [-2.92, 2.62]	-0.25 [-2.92, 2.39]	0.06 [-3.72, 3.9]						
L 804	L 797	L Fields of Forel	9.00 ± 3.30	10	21.36 ± 9.59	10	19.94 ± 6.87	10	18.68 ± 7.87	8	1.2 [-1.57, 3.97]	1.08 [-1.63, 3.79]	-1.76 [-5.67, 2.14]						
R 10671	R 1097	R Median eminence	27.35 ± 15.86	12	11.59 ± 5.45	14	10.28 ± 6.11	10	18.75 ± 8.98	10	-0.69 [-3.36, 1.98]	-0.55 [-3.05, 1.96]	0.09 [-3.51, 3.76]						
L 10671	L 1097	L Median eminence	13.07 ± 13.07	12	8.60 ± 4.74	14	7.11 ± 5.83	11	18.56 ± 11.18	10	-0.68 [-3.29, 1.9]	-0.32 [-2.84, 2.16]	0.58 [-3.04, 4.21]						
R 313	R 343	R Midbrain	13.92 ± 3.61	13	10.76 ± 4.06	13	10.85 ± 3.13	12	11.33 ± 2.57	10	-3.02 [-133.71, 127.62]	3.11 [-131.31, 122.12	-1.17 [-193.86, 189.46]						
L 313	L 343	L Midbrain	10.46 ± 2.52	13	11.28 ± 3.78	15	12.52 ± 3.99	12	11.91 ± 3.27	11									
R 339	R 313	R Midbrain, sensory related	1.56 ± 1.05	11	16.73 ± 8.28	11	18.99 ± 15.77	10	28.05 ± 6.58	7	9.6 [-134.33, 153.42]	8.39 [-133.47, 149.22	-7.81 [-216.13, 203.07]						
L 339	L 313	L Midbrain, sensory related	13.92 ± 8.73	11	10.29 ± 5.43	11	4.57 ± 2.91	10	19.71 ± 4.02	7	-3.3 [-144.11, 142.05]	2.39 [-141.85, 136.59	-1.36 [-217.22, 211.33]						
L 302	L 339	L Superior colliculus, sensory related	3.62 ± 2.45	6	25.89 ± 12.65	7	9.45 ± 6.26	7	34.78 ± 4.42	6	1.9 [-180.58, 182.77]	6.19 [-177.05, 189.46	-6.14 [-265.95, 249.74]						
R 599626	R 339	R Subcommissural organ	0.00 ± 0.00	11	19.75 ± 19.75	9	32.00 ± 32.00	10	112.82 ± 61.17	7	18.61 [174.82, 464.98]	95.42 [46.97, 342.41	607.22 [389.49, 829.49]*	197.24 [-345.02, -49.76	-320.09 [-464.47, -174.62]*	128.13 [-1293.09, -974.8	123.11 [-30.06, 272.43]	31.26 [-1096.35, -764.77	-807.95 [-973.31, -651.92]*
L 599626	L 339	L Subcommissural organ	25.25 ± 17.73	11	0.00 ± 0.00	9	6.40 ± 6.40	10	8.47 ± 8.47	7	-2.31 [-145.36, 139.38]	2.42 [-149.44, 145.37	-3.86 [-222.75, 212.66]						
R 323	R 313	R Midbrain, motor related	12.80 ± 3.77	13	9.93 ± 3.72	14	11.31 ± 3.46	12	11.02 ± 2.26	10	-3.24 [-133.21, 126.52]	2.91 [-127.47, 122.15	-2.34 [-189.68, 186.19]						
L 323	L 313	L Midbrain, motor related	8.10 ± 1.78	13	8.19 ± 3.03	15	17.16 ± 7.01	12	12.37 ± 4.02	10	-0.65 [-131.04, 130.83]	1.67 [-128.17, 120.96	-3.86 [-189.88, 181.71]						
L 381	L 323	L Substantia nigra, reticular part	0.00 ± 0.00	6	2.46 ± 2.08	8	1.32 ± 1.32	8	3.87 ± 1.78	7	11.4 [-169.66, 189.43]	2.58 [-153.55, 201.62	-2.79 [-253.49, 244.06]						
R 128	R 323	R Midbrain reticular nucleus	11.21 ± 3.94	8	9.30 ± 3.98	10	12.88 ± 5.13	10	19.43 ± 3.14	6	-0.83 [-159.87, 159.47]	-1.9 [-158.52, 155.5]	-3.54 [-229.93, 227.06]						
L 128	L 323	L Midbrain reticular nucleus	8.21 ± 2.11	8	6.40 ± 2.12	10	9.82 ± 4.01	10	18.74 ± 4.60	7	-1.82 [-159.5, 151.42]	1.51 [-156.21, 155.85	-1.96 [-223.72, 221.27]						
R 795	R 323	R Periaqueductal gray	37.03 ± 14.58	13	24.15 ± 7.42	14	24.91 ± 6.73	12	20.97 ± 4.74	10	-3.38 [-132.66, 125.7]	2.73 [-128.72, 123.12	-0.87 [-188.96, 189.11]						
L 795	L 323	L Periaqueductal gray	21.49 ± 4.62	13	20.37 ± 7.09	14	35.34 ± 11.26	12	21.77 ± 6.05	10	-2.17 [-134.17, 132.26]	-1.58 [-131.74, 126]	-3.46 [-198.28, 187.84]						
R 50	R 795	R Precommissural nucleus	82.12 ± 31.21	10	54.36 ± 15.87	13	66.33 ± 14.97	12	42.45 ± 11.48	10	-2.19 [-139.61, 142.02]	-1.9 [-141.98, 136.23	-3.72 [-199.54, 194.99]						
L 50	L 795	L Precommissural nucleus	50.80 ± 12.39	10	42.81 ± 15.30	13	64.84 ± 20.75	12	99.93 ± 36.10	10	-1.01 [-140.23, 141.13]	0.79 [-136.39, 134.57	-4.37 [-205.26, 193.6]						
R 1100	R 323	R Pretectal region	9.03 ± 2.68	11	15.24 ± 7.46	12	10.46 ± 3.16	11	10.98 ± 2.12	8	-1.46 [-145.99, 139.12]	-0.45 [-136.85, 138.3	-3.84 [-212.29, 202.82]						
L 1100	L 323	L Pretectal region	8.00 ± 1.87	12	15.56 ± 6.90	11	10.30 ± 4.35	10	13.15 ± 2.48	7	-1.37 [-144.02, 139.39]	0.32 [-136.35, 138.69	-4.38 [-218.24, 206.69]						
R 215	R 1100	R Anterior pretectal nucleus	12.71 ± 3.87	11	25.42 ± 14.17	12	20.25 ± 6.15	11	21.81 ± 5.23	8	-1.31 [-138.32, 136.47]	-0.8 [-135.94, 136.54	-3.72 [-212.91, 202.12]						
L 215	L 1100	L Anterior pretectal nucleus	11.74 ± 3.08	12	24.09 ± 13.07	11	19.79 ± 6.68	10	26.81 ± 6.84	7	-0.53 [-143.89, 140.24]	-1.4 [-139.46, 137.53	-3.83 [-213.6, 206.28]						
R 628	R 1100	R Nucleus of the optic tract	15.98 ± 6.28	8	11.08 ± 5.65	8	17.33 ± 6.37	8	21.68 ± 7.47	7	-1.43 [-166.75, 162.93]	1.83 [-165.76, 162.76	-2.87 [-243.92, 229.87]						
L 628	L 1100	L Nucleus of the optic tract	16.47 ± 4.81	8	23.91 ± 8.96	8	10.44 ± 6.98	9	24.39 ± 3.43	7	-2.52 [-160.21, 153.26]	2.11 [-166.22, 160.13	-1.28 [-229.31, 232.48]						
R 634	R 1100	R Nucleus of the posterior commissure	38.89 ± 15.69	9	57.23 ± 35.39	8	16.90 ± 8.36	8	22.61 ± 8.16	7	-4.43 [-165.82, 150.9]	2.66 [-162.31, 155.79	-0.75 [-230.15, 229.57]						
L 634	L 1100	L Nucleus of the posterior commissure	24.99 ± 10.14	9	52.78 ± 33.71	8	26.19 ± 12.23	8	25.68 ± 7.98	7	-2.58 [-163.75, 157.49]	-0.3 [-162.88, 158.72	-4.69 [-238.86, 231.37]						
R 706	R 1100	R Olivary pretectal nucleus	16.68 ± 7.28	8	10.62 ± 4.76	8	13.39 ± 5.28	7	22.15 ± 8.52	7	-0.19 [-165.9, 169.17]	1.26 [-165.03, 162.98	-4.52 [-245.06, 233.62]						
L 706	L 1100	L Olivary pretectal nucleus	18.43 ± 9.41	6	8.94 ± 4.73	8	3.63 ± 2.33	6	25.77 ± 7.78	7	-3.41 [-191.55, 188.85]	2.73 [-179.72, 171.22	-0.77 [-258.33, 246.73]						
R 1061	R 1100	R Posterior pretectal nucleus	33.81 ± 19.98	6	48.73 ± 28.74	7	42.69 ± 20.45	7	18.11 ± 8.25	6	-1.85 [-183.98, 180.57]	-2.25 [-185.4, 183.14	-3.53 [-255.14, 254.51]						
L 1061	L 1100	L Posterior pretectal nucleus	36.68 ± 15.04	8	30.34 ± 15.06	8	23.00 ± 17.77	9	22.33 ± 8.36	7	-2.79 [-163.26, 160.02]	3.18 [-169.48, 162.15	-2.56 [-235.03, 229.04]						
R 549009	R 1100	R Retroparafascicular nucleus	10.16 ± 10.16	6	1.40 ± 1.40	8	14.28 ± 8.01	6	14.69 ± 8.61	6	-2.13 [-187.46, 191.56]	-2.98 [-178.5, 174.73	-2.29 [-255.61, 254.15]						

Table S2. **Results from parent and subregion level tdT+ mapping.** Mean tdT+ cells/mm^2 +/- standard error of the mean and sample size for each experimental group (n) for each sampled brain region. Parent ID indicates the hierarchical "parent" area for each region. Results from Bayesian statistical models (genotype, treatment, and brain region as fixed effects, animal as random effect) show the estimated relative change (normalized to regional WT-Sal) and 95% credible intervals (CI) in tdT+ cells due to interactions between brain region and 5XFAD genotype (genotype effect), PTZ treatment (treatment effect), and 5XFAD genotype and PTZ treatment (Interaction). Estimated marginal means and 95% CIs were used for group contrasts at each brain region, where credible genotype, treatment, or interaction effects were found. Separate models were performed for the isocortex, olfactory areas, hippocampal formation, cortical subplate, striatum, pallidum, thalamus, hypothalamus, and midbrain. *=Results are considered credible when the 95% CI does not cross 0.

Brain region	Dependent variable	Independent variable					
		Sex		Genotype		Kindling	
		t statistic (DF) (M)	p value	t statistic (WT)	p value	t statistic (Sal)	p value
R isocortex	**AT8+/mm^2**	-0.743(46)	0.46	-4.58(46)	<0.0001	-1.838(46)	0.073
R olfactory areas		-0.3(48)	0.77	-2.55(48)	0.014	-0.143(48)	0.89
R hippocampus		-2.52(48)	0.015	-5.014(48)	<0.0001	-0.355(48)	0.72
R cortical subplate		-1.162(48)	0.25	-5.533(48)	<0.0001	-0.437(48)	0.66
R striatum		-1.582(48)	0.12	-8.122(48)	<0.0001	-0.49(48)	0.63
R thalamus		-1.43(48)	0.16	-1.55(48)	0.13	-0.766(48)	0.48
R hypothalamus		-0.456(46)	0.65	-1.187(46)	0.24	-0.381(46)	0.71
R midbrain		-1.948(41)	0.0583	-0.395(41)	0.7	0.144(41)	0.88
R fiber tracts		-2.249(48)	0.016	-6.894(48)	<0.0001	-0.783(48)	0.44
L isocortex		-1.558(46)	0.13	-5.103(46)	<0.0001	-4.089(46)	0.073
L olfactory areas		-0.194(46)	0.85	-3.213(46)	0.0024	-0.757(46)	0.453
L hippocampus		-3.002 (48)	0.0043	-5.559(48)	<0.0001	-0.468(48)	0.64
L cortical subplate		-0.28(48)	0.79	-4.773(48)	<0.0001	-1.026(48)	0.3
L striatum		0.353(48)	0.73	-5.746(48)	<0.0001	-1.756(48)	0.085
L Thalamus		-0.463(47)	0.65	-4.681(47)	<0.0001	-1.825(47)	0.074
L hypothalamus		-0.0781(45)	0.94	-0.52(45)	0.6	0.271(45)	0.79
L midbrain		-1.464(44)	0.15	-1.171(44)	0.25	-0.781(44)	0.44
L fiber tracts		-1.285(48)	0.2	-4.275(48)	<0.0001	-2.012(48)	0.05
R isocortex	**tdT+/mm^2**	1.844(50)	0.071	-2.573(50)	0.013	-4.872(50)	<0.0001
R olfactory areas		2.164(49)	0.035	-2.585(49)	0.013	-4.296(49)	<0.0001
R hippocampus		1.091(48)	0.2803	-1.809(48)	0.076	-4.476(48)	<0.0001
R cortical subplate		1.824(51)	0.074	-2.883(51)	0.0057	-4.29(51)	<0.0001
R striatum		1.937(51)	0.058	-1.877(51)	0.066	-4.644(51)	<0.0001
R thalamus		0.118(51)	0.91	-3.235(51)	0.0021	-1.676(51)	0.01
R hypothalamus		1.306(49)	0.17	-1.576(49)	0.12	-0.712(49)	0.48
R midbrain		0.156(44)	0.88	0.336(44)	0.74	0.391(44)	0.7
L isocortex		1.61(50)	0.11	-2.66(50)	0.011	-4.848(50)	<0.0001
L olfactory areas		1.762(49)	0.084	-2.802(49)	0.0073	-4.103(49)	0.00015
L hippocampus		0.887(51)	0.38	-1.504(51)	0.14	-4.844(51)	<0.0001
L cortical subplate		1.555(51)	0.13	-3.057(51)	0.0035	-4.519(51)	<0.0001
L striatum		1.142(50)	0.16	-2.455(50)	0.018	-5.148(50)	<0.0001
L Thalamus		-0.0743(50)	0.94	-3.939(50)	0.00025	-1.519(50)	0.14
L hypothalamus		1.753(48)	0.086	-1.805(48)	0.077	-1.326(48)	0.19
L midbrain		0.322(47)	0.75	-0.453(47)	0.65	-0.099(47)	0.92
	**Seizure severity**	0.428(27)	0.67	-2.59(27)	0.015	NA	NA
	**NOR**	-0.138(52)	0.89	1.925(52)	0.06	1.142(52)	0.26
	**OF distance**	-1.24(56)	0.22	2.425(56)	0.019	1.03(56)	0.31
	**OF center time**	-0.181(56)	0.86	-0.408(56)	0.68	0.0131(56)	0.99
	**OF rearing**	3.165(56)	0.0025	4.585(56)	<0.0001	-0.143(56)	0.89
	**CFC recent**	-0.948(56)	0.35	-1.309(56)	0.2	-1.36(56)	0.18
	**CFC remote**	-0.441(55)	0.66	-0.887(55)	0.38	-1.469(55)	0.15
R cortex	**Fluorojade-B**	-1.282(54)	0.21	-7.895(54)	<0.0001	-0.162(54)	0.87
R hippocampus		-1.303(54)	0.198	-6.412(54)	<0.0001	-1.741(54)	0.087
L cortex		-1.092(55)	0.389	-9.865(55)	<0.0001	-0.869(55)	0.29
L hippocampus		-1.654(55)	0.1	-4.957(55)	<0.0001	-0.857(55)	0.39

Table S3. Multiple linear regressions for sex effects in mouse studies. Multiple linear regression was performed including sex, genotype, and kindling as independent variables for AT8+ levels and tdTomato (tdT)+ levels, and behavioral results. We found that female sex (reference variable) was associated with increased AT8+ levels in the hippocampus ipsilateral (right, R) and contralateral (left, L) to AD-tau injection compared to male mice and that male sex was associated with increased tdT labelling in the R olfactory areas. Females displayed less rearing behavior in the open field assay.

Dependent variable	Independent variable					
	Cohort		Genotype		Kindling	
	F (DF)	p value	F (DF)	p value	F (DF)	p value
**NOR**	1.206 (11, 39)	0.32	1.307 (1, 39)	0.26	1.613 (1, 39)	0.21
**CFC recent**	1.232 (11, 43)	0.30	1.422 (1, 43)	0.24	1.286 (1, 43)	0.26
**CFC remote**	1.114 (11, 42)	0.38	0.993 (1, 42)	0.32	1.745 (1, 42)	0.19

Table S4. Multiple linear regressions for cohort effects in memory studies. Multiple linear regression was performed including cohort, genotype, and kindling as independent variables for performance in novel object recognition (NOR) and CFC. No significant cohort effects were found.

Brain Region	Dependent variable	Independent variable			
		Sex		Seizure history	
		z value (M)	p value	z value (+Sz)	p value
Amygdala	Tau pathology score	0.845	0.4	NA	NA
Dentate gyrus		-0.41	0.68	-0.59	0.56
CA/Subiculum		-1.58	0.12	NA	NA
Entorhinal cortex		NA	NA	NA	NA
Middle frontal gyrus		1.07	0.28	1.75	0.08
Angular gyrus		1.05	0.29	NA	NA
SMT gyrus		1.22	0.22	1.13	0.26
Cingulate gyrus		1.58	0.11	1.6	0.11
Occipital cortex		0.32	0.75	0.27	0.79
Striatum		0.53	0.59	0.92	0.36
Globus pallidus		-0.99	0.32	0.035	0.97
Thalamus		-0.22	0.82	0.98	0.33
Midbrain		0.72	0.47	0.22	0.83
Substantia nigra		3.03	0.0024	1.4	0.16
Pons		1.5	0.13	0.22	0.82
Locus Coeruleus		2.09	0.036	0.45	0.65
Medulla		0.56	0.57	-1.29	0.2
Cerebellum		-0.45	0.65	NA	NA

Table S5. Statistical analyses for sex effects in human studies. Ordinal linear regressions for regional tau pathology scores with seizure history and sex as independent variables. NA indicates that there were not enough levels of the dependent variable for comparison. Male sex was associated with worsened tau scores in the substantia nigra and locus coeruleus.

Antibody	Ref (Distributor)	Isotype	Size (kDa)
NeuN	ABN78 (EMD Millipore)	Rabbit polyclonal IgG	48/42
phospho-tau AT8 [Ser202; Thr205]	MN1020 (Millipore)	Mouse monoclonal IgG1κ	40-80

Table S6. Primary antibody information.

ID	Seizure history	Age at death (years)	PMI	Braak stage	Sex	Age of onset (years)	Brain weight (grams)	Ventricular enlargement	Brain atrophy
AD-Sz1	Absent	84	24	6	Female	78	1204	Mild	None/Normal
AD-Sz2	Absent	90+	19	6	Male	76	1309	Severe	Mild
AD-Sz3	Absent	78	6	6	Male	70	1190	Severe	Severe
AD-Sz4	Absent	70	5	6	Male	61	1256	Moderate	Moderate
AD-Sz5	Absent	90+	84	6	Female	70	908	Moderate	Moderate
AD-Sz6	Absent	79	17	6	Male	72	1345	Moderate	Mild
AD-Sz7	Absent	90+	10	6	Male	82	1366	None/Normal	Mild
AD-Sz8	Absent	88	20		Female	76	1094	Mild	Moderate
AD-Sz9	Absent	90+	5	3	Female	89	1123	Mild	Mild
AD-Sz10	Absent	87	19	6	Male	73	1198	Mild	Mild
AD-Sz11	Absent	86	10	6	Male	72	1278	Moderate	Mild
AD-Sz12	Absent	62	8	6	Female	56	988	None/Normal	Mild
AD-Sz13	Absent	90+	17		Female	83	1124	None/Normal	Mild
AD-Sz14	Absent	79	4	6	Female	69	909	Mild	Moderate
AD-Sz15	Absent	83	4	6	Male	69	1155	Severe	Severe
AD-Sz16	Absent	85	9	5	Female	76	1014	Moderate	Moderate
AD-Sz17	Absent	84	21	3	Male	72	1265	Mild	Mild
AD-Sz18	Absent	44	44	6	Male	42	1510	None/Normal	None/Normal
AD-Sz19	Absent	85	14	5	Female	78	1138	None/Normal	Moderate
AD-Sz20	Absent	63	16	6	Male	54	1243	None/Normal	Mild
AD-Sz21	Absent	74	4	6	Female	62	989	Moderate	Moderate
AD-Sz22	Absent	90+	48	3	Male	88	1438	Moderate	Moderate
AD-Sz23	Absent	84	21.5	6	Female	69	1151	Mild	Mild
AD-Sz24	Absent	70	6		Female	57	978	Severe	Severe
AD-Sz25	Absent	71	4	6	Male	59	1330	Moderate	Moderate
AD-Sz26	Absent	63	5		Female	53	1137	None/Normal	None/Normal
AD-Sz27	Absent	84	18	5	Male	77	1281	Moderate	None/Normal
AD-Sz28	Absent	85	20		Female	80	1026	Moderate	Moderate
AD-Sz29	Absent	78	18	6	Male	68	1100	None/Normal	Moderate
AD-Sz30	Absent	65	8.5	6	Female	55	997	Severe	Severe
AD-Sz31	Absent	84	18.5	6	Male	68	1185	Moderate	Severe
AD-Sz32	Absent	90+	24	6	Female	86	938	Mild	Mild
AD-Sz33	Absent	90+	12	6	Female	79	1269	Mild	Mild
AD-Sz34	Absent	74	6	6	Female	64	1149	Mild	Mild
AD-Sz35	Absent	70	16	6	Female	51	845	Severe	Severe
AD-Sz36	Absent	64	16.5	6	Male	55	1320	None/Normal	None/Normal
AD-Sz37	Absent	84	7	6	Male	69	1309	Moderate	Mild
AD-Sz38	Absent	78	6.5	6	Male	64	926	Severe	Severe
AD-Sz39	Absent	65	17	6	Male	59	960	Moderate	Severe

AD-Sz40	Absent	64	20	3	Female	59	1098	Moderate	Severe
AD-Sz41	Absent	72	8	6	Male	63	1167	Moderate	Moderate
AD-Sz42	Absent	68	7	6	Male	54	1058	Moderate	Moderate
AD-Sz43	Absent	88	15	5	Female	78	1145	Mild	None/Normal
AD-Sz44	Absent	89	12	6	Female	72	1080	Severe	Moderate
AD-Sz45	Absent	90+	12	6	Female	76	1283	Moderate	Moderate
AD-Sz46	Absent	89	5		Female	76	1060	Severe	Severe
AD-Sz47	Absent	85	12	6	Female	70	1069	Moderate	Mild
AD-Sz48	Absent	90+	11	6	Male	82	1459	Moderate	Moderate
AD-Sz49	Absent	90+	4	6	Male	84	1105	Moderate	Moderate
AD-Sz50	Absent	75	18.5	6	Male	61	1210	Severe	Mild
AD-Sz51	Absent	84	5		Female	74	1053	Severe	Severe
AD-Sz52	Absent	81	18	6	Male	73	1427	Moderate	Mild
AD-Sz53	Absent	83	4.5	6	Male	66	1013	Moderate	Severe
AD-Sz54	Absent	84	11	6	Female	78	1043	Moderate	Moderate
AD-Sz55	Absent	90+	20	5	Male	83	1237	Moderate	Moderate
AD-Sz56	Absent	81	26	6	Female	65	1260	Moderate	Mild
AD-Sz57	Absent	64	14	6	Female	53	1099	Mild	Moderate
AD-Sz58	Absent	61	18	6	Male	53	1203	Severe	Severe
AD-Sz59	Absent	86	6.5	6	Male	75	1147	Severe	Moderate
AD-Sz60	Absent	71	4.5	6	Male	62	1234	Mild	Moderate
AD-Sz61	Absent	85	4	6	Female	66	969	Severe	Severe
AD-Sz62	Absent	82	7	6	Female	71	1113	Moderate	Moderate
AD-Sz63	Absent	90+	18	4	Female	75	1012	Moderate	Mild
AD-Sz64	Absent	86	6	5	Female	76	889	Mild	Moderate
AD-Sz65	Absent	90+	9	3	Male	86	1232	Mild	Moderate
AD-Sz66	Absent	79	20	6	Male	72	1147	Moderate	Mild
AD-Sz67	Absent	85	16	5	Male	65	1404	Severe	Moderate
AD-Sz68	Absent	83	11		Female	66	845	Severe	Severe
AD-Sz69	Absent	75	18	6	Female	65	1207	Moderate	Severe
AD-Sz70	Absent	70	4		Male	56	1201	Severe	Severe
AD-Sz71	Absent	74	8	6	Male	59	1173	Moderate	Mild
AD-Sz72	Absent	90+	10	6	Female	83	1214	Mild	Mild
AD-Sz73	Absent	85	4	6	Female	61	967	Severe	Severe
AD-Sz74	Absent	88	27	5	Female	76	1205	Moderate	Mild
AD-Sz75	Absent	63	27		Female	51	999	Severe	Moderate
AD-Sz76	Absent	83	18	6	Male	73	1336	Moderate	Mild
AD-Sz77	Absent	89	18	6	Female	74	1208	Moderate	Severe
AD-Sz78	Absent	88	7		Female	72	1016	Severe	Severe
AD-Sz79	Absent	88	17	4	Male	79	1177	Mild	Mild
AD-Sz80	Absent	85	20	N/A	Female	79	1202	Moderate	Moderate

AD-Sz81	Absent	70	3.5	6	Female	52	1068	Moderate	Moderate
AD-Sz82	Absent	86	21	6	Female		1128	Mild	Moderate
AD-Sz83	Absent	75	19	6	Male	65	1193	Moderate	Moderate
AD-Sz84	Absent	73	22	6	Female	63	1022	Moderate	Moderate
AD-Sz85	Absent	73	20	5	Male	66	1319	Mild	Mild
AD-Sz86	Absent	77	17	6	Female	70	1102	Moderate	Mild
AD-Sz87	Absent	79	11	5	Female	68	1049	Moderate	Moderate
AD-Sz88	Absent	68	20	6	Male		1134	Mild	Mild
AD-Sz89	Absent	72	24	6	Male	68	1176	Moderate	Moderate
AD-Sz90	Absent	55	37	6	Female		868		
AD-Sz91	Absent	75	5	6	Male	64	1235	Severe	Mild
AD-Sz92	Absent	53	14	6	Female		1146	None/Normal	Moderate
AD-Sz93	Absent	75	12	6	Female	70	1140	Mild	Moderate
AD-Sz94	Absent	72	17	5	Male	63	1388	Mild	Mild
AD-Sz95	Absent	57	9	6	Male	52	1331	Mild	Mild
AD+Sz1	Yes	83	7	6	Female	59	783	Moderate	Severe
AD+Sz2	Yes	83	11	6	Male	71	1077	Severe	Severe
AD+Sz3	Yes	81	5	6	Female	71	847	Severe	Moderate
AD+Sz4	Yes	77	3	6	Female	71	1010	Mild	Moderate
AD+Sz5	Yes	76	45	6	Female	66	1139	Mild	Mild
AD+Sz6	Yes	65	11	6	Female	55	1125	Moderate	Moderate
AD+Sz7	Yes	80	12	6	Female	67	1063	Moderate	Moderate
AD+Sz8	Yes	68	12	6	Female	56	942	Severe	Severe
AD+Sz9	Yes	89	3	6	Male	77	1034	Mild	Mild
AD+Sz10	Yes	71	23	6	Male	65	1206	Mild	Moderate
AD+Sz11	Yes	68	9	6	Female	60	1000	Mild	Moderate

Table S7. Clinical information for human subjects. PMI=postmortem interval.

AT8/mm^2 ~ Genotype * Treatment + (1 + Genotype * Treatment | Parent.ID/Region.ID) + (1 | Ms.ID)			
**Major brain region**	**Rhat**	**Bulk ESS Range**	**Tail ESS Range**
Isocortex	<1.01	1993 - 18922	1738 - 10904
Olfactory areas	<1.01	2712 - 16265	2930 - 11270
Hippocampus	<1.01	1007 - 18811	898 - 11036
Cortical subplate	<1.01	1220 - 19825	3209 - 11026
Striatum	<1.01	837 - 14707	1240 - 11230
Pallidum	<1.01	919 - 16733	1256 - 11205
Thalamus	<1.01	1172 - 14301	2007 - 10439
Hypothalamus	<1.01	1568 - 14125	2111 - 10665
Midbrain	<1.01	2141 - 19508	2029 - 11177
Fiber tracts	<1.01	944 - 21268	1268 - 10952
**AT8/mm^2 (WT/Sal norm) ~ Genotype * Treatment * region + (1 | Ms.ID)**			
**Major brain region**	**Rhat**	**Bulk ESS Range**	**Tail ESS Range**
Isocortex	<1.01	1579 - 15452	3983 - 9145
Olfactory areas	<1.01	3187 - 15853	5676 - 9886
Hippocampus	<1.01	3083 - 18569	5592 - 11169
Cortical subplate	<1.01	3166 - 13096	5307 - 10225
Striatum	<1.01	3341 - 16352	6174 - 10907
Pallidum	<1.01	2840 - 11702	5383 - 9888
Thalamus	<1.01	1259 - 12518	2607 - 8169
Hypothalamus	<1.01	1193 - 12079	2220 - 8767
Midbrain	<1.01	3511 - 19084	6321 - 10395
Fiber tracts	<1.01	1098 - 12365	2501 - 7641
**tdT/mm^2 ~ Genotype * Treatment + (1 + Genotype * Treatment | Parent.ID/Region.ID) + (1 | Ms.ID)**			
**Major brain region**	**Rhat**	**Bulk ESS Range**	**Tail ESS Range**
Isocortex	<1.01	568 - 19395	1473 - 11109
Olfactory areas	<1.01	2115 - 15291	2880 - 11167
Hippocampus	<1.01	1291 - 19055	2063 - 11061
Cortical subplate	<1.01	1299 - 20982	2855 - 11230
Striatum	<1.01	2685 - 20878	3321 - 11448
Pallidum	<1.01	2705 - 20670	4453 - 12086
Thalamus	<1.01	1983 - 21508	2432 - 11444
Hypothalamus	<1.01	1673 - 27406	2893 - 17714
Midbrain	<1.01	1756 - 25477	3024 - 11631
**tdT/mm^2 (WT/Sal norm) ~ Genotype * Treatment * region + (1 | Ms.ID)**			
**Major brain region**	**Rhat**	**Bulk ESS Range**	**Tail ESS Range**
Isocortex	<1.01	404 - 13523	622 - 8643
Olfactory areas	<1.01	1355 - 9235	2334 - 8571
Hippocampus	<1.01	4702 - 36338	7764 - 13882
Cortical subplate	<1.01	2779 - 12546	4884 - 8253
Striatum	<1.01	1532 - 8901	2498 - 7379
Pallidum	<1.01	3526 - 23707	6144 - 10865
Thalamus	<1.01	349 - 13533	968 - 11308
Hypothalamus	<1.01	1783 - 22733	3947 - 17378
Midbrain	<1.01	4344 - 22070	5982 - 10918

Table S8. Bayesian model diagnostics. Diagnostics from Bayesian models incorporating parent region/subregion as random effects, or each brain region independently as a fixed effect and genotype and treatment as fixed effects. Ms.ID = subject. Rhat of less than 1.01 indicates

strong Markov chain Monte Carlo chain convergence. Bulk effective sample size (ESS) >400 and tail ESS >400 indicate reliable estimates of median and tails of the posterior distribution, respectively.
