# Supplementary material for: Hyperactive neuronal networks facilitate tau spread in an Alzheimer’s disease mouse model
Source: bioRxiv. 2024 Dec 3:2024.12.01.625514. Preprint. [Version 2] doi: 10.1101/2024.12.01.625514 (PMC11642807; doi:10.1101/2024.12.01.625514)
Supplement: Supplementary file 1 [file media-1.pdf]

ID	Parent ID	Region	WT.Sal	n	WT.PTZ	n	5X.Sal	n	5X.PTZ	n	Genotype F	Genotype p	Treatment F	Treatment p	Interaction F	Interaction p	p1	p2	p3	p4	p5	p6
8		Basic cell groups and regions	10.15 ± 2.79	15	13.47 ± 2.05	15	29.28 ± 5.12	12	33.61 ± 5.83	10	25.9	0.00006	0.568	0.455	0.0171	0.897		**	***	*	**	
567	8	Cerebrum	11.10 ± 2.70	15	16.62 ± 2.92	15	39.50 ± 6.91	12	42.17 ± 7.70	10	29.8	1.69E-06	0.405	0.528	0.0823	0.775		***	***	**	**	
688	567	Cerebral cortex	14.51 ± 3.57	15	21.73 ± 3.97	15	54.29 ± 9.68	12	54.21 ± 10.47	10	28.4	2.58E-06	0.141	0.709	0.286	0.595		***	**	**	*	
695	688	Cortical plate	18.21 ± 4.41	15	27.58 ± 5.10	15	73.52 ± 14.09	12	67.47 ± 13.34	10	27.1	4.05E-06	0.00669	0.935	0.696	0.408		***	**	**	*	
315	695	Isocortex	10.28 ± 2.23	15	14.04 ± 4.14	15	23.64 ± 4.35	10	35.01 ± 3.64	10	21.6	0.0000284	3.53	0.0665	1.06	0.308			***		**	
500	315	Somatomotor areas	3.75 ± 0.79	15	12.15 ± 7.23	15	18.43 ± 3.96	10	24.66 ± 5.73	10	6.58	0.0136	2.11	0.154	0.0417	0.839			*			
985	500	Primary motor area	6.44 ± 1.29	13	14.88 ± 7.80	15	23.84 ± 5.28	10	35.46 ± 7.70	9	8.82	0.00486	1.88	0.177	0.0623	0.804			*			
993	500	Secondary motor area	5.80 ± 1.65	11	23.38 ± 15.05	15	36.65 ± 7.58	10	53.69 ± 11.17	9	6.71	0.0132	1.51	0.227	0.000516	0.982			*			
453	315	Somatosensory areas	7.98 ± 2.04	15	10.58 ± 3.13	15	16.86 ± 2.61	10	33.54 ± 5.92	10	20.9	0.0000366	5.81	0.02	4.09	0.0489			****		***	*
322	453	Primary somatosensory area	13.09 ± 3.48	14	14.55 ± 4.78	15	22.31 ± 4.10	9	39.50 ± 7.03	9	12	0.00123	2.23	0.143	2.51	0.12			**		**	
353	322	Primary somatosensory area, nose	5.00 ± 1.42	10	2.96 ± 0.91	9	34.14 ± 17.04	8	130.19 ± 82.08	8	4.05	0.0529	1.39	0.248	1.64	0.209						
329	322	Primary somatosensory area, barrel field	21.31 ± 6.52	13	25.20 ± 7.66	13	36.52 ± 7.51	10	60.61 ± 13.56	9	8.31	0.00613	1.51	0.226	1.33	0.256			*		*	
337	322	Primary somatosensory area, lower limb	20.10 ± 9.07	13	14.48 ± 5.11	11	49.70 ± 10.78	10	96.53 ± 21.81	9	20	0.0000645	2.35	0.134	4.69	0.0365			***		***	
345	322	Primary somatosensory area, mouth	7.15 ± 1.86	13	11.45 ± 4.21	13	17.03 ± 4.05	9	20.59 ± 5.08	9	6.07	0.0182	1.11	0.299	0.00913	0.924						
369	322	Primary somatosensory area, upper limb	13.70 ± 4.35	14	11.60 ± 4.83	13	24.59 ± 4.76	10	67.75 ± 19.75	9	13.3	0.000735	3.46	0.0701	6.43	0.015			***		***	*
361	322	Primary somatosensory area, trunk	51.38 ± 26.74	14	37.77 ± 26.23	12	95.76 ± 30.65	10	145.56 ± 41.58	9	5.75	0.0212	0.212	0.648	1.05	0.312						
182000000	322	Primary somatosensory area, unassigned	72.29 ± 39.72	14	8.27 ± 3.64	11	25.05 ± 5.66	10	83.67 ± 24.57	9	0.106	0.747	0.141	0.709	4.81	0.0342						
378	453	Supplemental somatosensory area	8.32 ± 2.89	11	18.36 ± 8.34	15	29.30 ± 4.90	9	60.38 ± 14.11	10	13.5	0.000685	3.93	0.0543	1.4	0.244			**		**	
1060	315	Gustatory areas	13.02 ± 3.89	15	11.76 ± 3.43	13	18.97 ± 5.00	10	25.50 ± 6.51	10	4.38	0.0422	0.259	0.613	0.701	0.407						
677	315	Visceral area	15.05 ± 5.31	13	29.68 ± 17.13	13	31.77 ± 9.44	9	44.54 ± 11.79	10	1.62	0.211	1.36	0.251	0.00557	0.941						
247	315	Auditory areas	17.78 ± 5.75	14	8.29 ± 2.05	15	20.08 ± 2.64	10	39.43 ± 8.95	9	10.3	0.00251	0.0599	0.808	7.88	0.00741			*		***	
1010	247	Dorsal auditory area	27.64 ± 11.66	12	10.47 ± 2.59	15	36.61 ± 4.73	10	56.42 ± 11.20	8	11.3	0.0017	0.451	0.506	5.27	0.0269					**	
1000	247	Primary auditory area	32.54 ± 13.40	13	18.16 ± 6.26	11	29.12 ± 6.48	10	63.99 ± 24.47	7	1.9	0.176	0.148	0.703	3.57	0.0668						
1030	247	Posterior auditory area	49.94 ± 26.62	6	12.42 ± 8.14	6	26.91 ± 10.67	8	72.00 ± 36.25	5	0.426	0.521	0.0172	0.897	3.89	0.0617						
1020	247	Ventral auditory area	33.66 ± 9.68	14	13.65 ± 3.35	15	27.87 ± 4.88	10	51.41 ± 15.55	8	2.95	0.0929	0.287	0.595	6.4	0.0152					*	
669	315	Visual areas	54.72 ± 25.32	14	48.36 ± 17.02	15	57.83 ± 19.53	10	110.80 ± 29.95	9	1.9	0.175	0.474	0.495	1.6	0.212						
402	669	Anterolateral visual area	53.82 ± 16.93	7	6.30 ± 2.84	7	32.97 ± 15.75	8	81.84 ± 37.60	5	1.29	0.268	0.0681	0.796	6.42	0.0186						
394	669	Anteromedial visual area	44.33 ± 13.76	12	95.21 ± 36.19	14	103.91 ± 29.82	10	215.19 ± 64.92	9	5.59	0.0228	3.57	0.066	0.632	0.431			*			
385	669	Primary visual area	78.58 ± 24.18	11	164.43 ± 89.74	9	82.68 ± 36.28	9	230.76 ± 68.78	6	0.276	0.603	3.69	0.0641	0.284	0.598						
533	669	posteromedial visual area	237.86 ± 167.43	10	153.57 ± 107.41	7	81.10 ± 31.50	9	152.00 ± 30.36	6	0.632	0.433	0.00805	0.929	0.417	0.524						
31	315	Anterior cingulate area	8.29 ± 2.44	15	13.78 ± 5.10	15	32.48 ± 10.49	10	38.50 ± 12.30	10	10.9	0.00184	0.619	0.436	0.00128	0.972			*			
39	31	Anterior cingulate area, dorsal part	6.93 ± 1.86	13	17.59 ± 10.04	14	42.33 ± 9.84	10	54.62 ± 16.08	10	12.7	0.000896	1.14	0.292	0.00637	0.937			*			
48	31	Anterior cingulate area, ventral part	21.21 ± 6.18	15	20.35 ± 6.30	14	57.09 ± 22.12	10	66.57 ± 21.94	10	8.71	0.00501	0.0877	0.768	0.139	0.711						
972	315	Prelimbic area	10.49 ± 4.07	10	18.19 ± 10.94	9	68.71 ± 32.24	4	48.29 ± 13.11	7	10.4	0.00336	0.117	0.735	1.14	0.295		*				
95	315	Agranular insular area	6.38 ± 2.48	14	12.03 ± 4.65	15	17.93 ± 3.66	9	20.94 ± 3.65	10	6.68	0.0131	1.49	0.229	0.113	0.739						
104	95	Agranular insular area, dorsal part	6.52 ± 2.24	11	15.32 ± 5.72	14	26.78 ± 9.26	9	28.42 ± 7.20	10	6.68	0.0135	0.665	0.42	0.316	0.577						
111	95	Agranular insular area, posterior part	11.70 ± 4.74	12	26.16 ± 17.79	12	35.56 ± 10.45	9	33.17 ± 6.35	10	1.69	0.201	0.411	0.525	0.521	0.475						
119	95	Agranular insular area, ventral part	11.71 ± 5.05	14	19.54 ± 10.15	14	26.73 ± 5.86	9	31.41 ± 8.69	10	2.67	0.11	0.736	0.396	0.0368	0.849						
254	315	Retrosplenial area	22.27 ± 9.52	14	24.48 ± 11.17	15	98.79 ± 32.94	10	88.68 ± 13.82	9	16.3	0.000217	0.108	0.745	0.124	0.726		*		*		
894	254	Retrosplenial area, lateral agranular part	28.55 ± 7.17	14	28.25 ± 12.27	15	78.36 ± 13.86	10	111.40 ± 19.00	9	26.2	6.49E-06	0.643	0.427	1.66	0.204		*	***	*	***	
879	254	Retrosplenial area, dorsal part	15.21 ± 3.59	15	12.53 ± 3.13	15	85.87 ± 21.90	9	110.52 ± 17.06	9	58.4	1.32E-09	0.502	0.482	1.53	0.222		***	****	***	****	
886	254	Retrosplenial area, ventral part	47.86 ± 24.36	15	60.11 ± 29.98	15	236.46 ± 88.67	10	177.67 ± 34.74	10	11.5	0.00147	0.133	0.717	0.617	0.436		*		*		
22	315	Posterior parietal association areas	39.18 ± 12.52	15	50.76 ± 23.16	15	62.21 ± 23.09	10	158.60 ± 46.08	9	6.2	0.0165	2.92	0.0946	2.71	0.107			*		*	
313000000	22	Anterior area	46.77 ± 13.33	15	92.78 ± 40.73	15	110.63 ± 45.89	10	266.65 ± 97.38	9	5.7	0.0212	3.21	0.0797	1.26	0.268			*			
417	22	Rostrolateral visual area	50.15 ± 21.36	14	25.85 ± 10.34	15	48.28 ± 12.49	10	196.09 ± 73.10	9	6.83	0.0123	1.7	0.199	7.4	0.0093			*		**	*
541	315	Temporal association areas	23.43 ± 6.28	13	19.91 ± 7.06	12	26.26 ± 4.11	10	34.78 ± 4.41	7	1.67	0.204	0.014	0.906	0.906	0.347						
922	315	Perirhinal area	49.67 ± 29.68	14	28.17 ± 10.75	13	24.22 ± 8.63	10	81.15 ± 23.26	8	0.229	0.635	0.184	0.67	3.14	0.084						
895	315	Ectorhinal area	22.00 ± 8.20	14	19.99 ± 6.73	13	28.60 ± 4.77	10	51.25 ± 8.81	8	5.53	0.0236	0.913	0.345	2.6	0.115					*	
698	695	Olfactory areas	18.34 ± 7.02	14	10.86 ± 1.78	14	23.93 ± 5.68	12	35.38 ± 5.97	10	7.06	0.0108	0.000777	0.978	2.97	0.0915					*	
159	698	Anterior olfactory nucleus	17.12 ± 5.41	8	19.64 ± 2.87	5	138.93 ± 49.51	6	133.42 ± 41.54	6	14.2	0.00112	0.156	0.697	0.0162	0.9		*				
589	698	Taenia tecta	9.34 ± 2.50	12	9.79 ± 2.38	9	62.86 ± 22.79	7	68.89 ± 34.04	8	9.96	0.00347	0.239	0.629	0.0245	0.877						
597	589	Taenia tecta, dorsal part	15.21 ± 4.11	12	25.67 ± 6.64	8	140.24 ± 59.50	7	137.62 ± 68.10	8	8.92	0.00547	0.27	0.607	0.0269	0.871						
814	698	Dorsal peduncular area	20.10 ± 8.89	14	22.98 ± 6.95	8	54.90 ± 18.17	6	90.51 ± 31.69	8	8.12	0.00759	2.35	0.135	0.843	0.365			*			
961	698	Piriform area	51.78 ± 23.45	10	36.69 ± 5.87	10	65.44 ± 19.47	7	78.16 ± 14.62	10	2.87	0.0999	1.37E-06	0.999	0.657	0.423						
619	698	Nucleus of the lateral olfactory tract	21.37 ± 7.57	13	6.88 ± 2.66	6	44.55 ± 18.56	11	58.94 ± 24.67	4	4.57	0.0408	0.0719	0.79	0.788	0.382						
260	619	Nucleus of the lateral olfactory tract, molecular layer	62.16 ± 36.47	13	13.94 ± 8.84	6	67.22 ± 34.14	10	37.24 ± 9.90	4	0.0815	0.777	1.08	0.307	0.0514	0.822						
268	619	Nucleus of the lateral olfactory tract, pyramidal layer	31.82 ± 9.56	13	14.51 ± 5.72	6	119.31 ± 47.51	11	181.98 ± 84.97	4	8.74	0.00602	0.0555	0.815	0.937	0.341						
1140	619	Nucleus of the lateral olfactory tract, layer 3	37.43 ± 10.38	13	8.60 ± 8.60	6	57.87 ± 26.94	11	97.09 ± 38.37	4	3.44	0.0735	0.0143	0.906	2.04	0.164						
631	698	Cortical amygdalar area	20.26 ± 7.06	14	7.22 ± 1.45	11	19.74 ± 7.48	9	23.57 ± 3.80	10	1.75	0.193	0.766	0.387	2.09	0.157						
639	631	Cortical amygdalar area, anterior part	46.91 ± 19.68	14	24.76 ± 6.14	10	74.29 ± 32.14	9	45.71 ± 8.80	10	1.56	0.22	1.35	0.253	0.0272	0.87						
647	631	Cortical amygdalar area, posterior part	29.81 ± 11.85	12	10.08 ± 2.54	12	22.09 ± 9.94	9	30.12 ± 7.01	8	0.405	0.528	0.916	0.345	2.42	0.128						
655	647	Cortical amygdalar area, posterior part, lateral zone	64.74 ± 25.38	13	24.92 ± 7.26	12	46.82 ± 17.49	9	69.03 ± 17.24	8	0.349	0.558	0.601	0.443	2.53	0.12						
663	647	Cortical amygdalar area, posterior part, medial zone	50.29 ± 22.20	11	14.53 ± 3.42	12	47.42 ± 26.24	8	49.42 ± 12.75	8	0.853	0.362	1.4	0.245	1.14	0.294						
788	698	Piriform-amygdalar area	50.03 ± 17.37	15	30.51 ± 8.44	11	54.78 ± 13.06	10	61.20 ± 12.39	10	1.33	0.255	0.221	0.64	0.788	0.38						
1090	695	Hippocampal formation	76.17 ± 21.82	15	113.86 ± 24.92	15	307.68 ± 54.66	12	288.09 ± 66.01	10	25	8.13E-06	0.0124	0.912	0.489	0.488		***	**	**	*	
1080	1090	Hippocampal region	102.19 ± 30.45	15	169.93 ± 33.74	15	413.26 ± 72.46	12	388.38 ± 95.07	10	22.4	0.0000201	0.0914	0.764	0.67	0.417		**	**	*		
375	1080	Ammon's horn	141.91 ± 46.22	15	218.54 ± 45.48	15	489.25 ± 85.73	12	503.46 ± 115.49	10	20.1	0.0000456	0.268	0.607	0.193	0.662		**	**	*	*	
382	375	Field CA1	254.69 ± 91.37	15	361.42 ± 72.10	15	856.87 ± 149.04	12	811.05 ± 164.63	10	20.8	0.0000356	0.0272	0.87	0.429	0.515		**	**	*	*	
423	375	Field CA2	415.99 ± 119.59	15	508.83 ± 108.52	15	1121.75 ± 168.86	12	1077.28 ± 296.20	10	14.6	0.000388	0.00156	0.969	0.167	0.685		*	*			
463	375	Field CA3	316.25 ± 107.59	14	547.25 ± 133.79	15	1116.28 ± 213.95	12	1130.61 ± 306.88	10	13.9	0.000516	0.266	0.609	0.337	0.564		*	*			
726	1080	Dentate gyrus	189.20 ± 55.75	15	362.60 ± 77.45	15	1016.85 ± 191.95	12	849.39 ± 251.92	10	21.6	0.0000264	0.0000037	0.998	1.41	0.241		***	*	**		
10700	726	Dentate gyrus, molecular layer	367.17 ± 114.58	15	636.27 ± 152.08	15	1744.62 ± 320.17	12	1496.69 ± 417.39	10	21.2	0.0000308	0.0000194	0.997	1.1	0.299		**	*	*		
10700	726	Dentate gyrus, polymorph layer	478.71 ± 137.74	15	1153.91 ± 259.60	15	2797.94 ± 545.16	12	2472.65 ± 823.91	10	17.5	0.000123	0.155	0.695	1.28	0.263		**	*	*		
632	726	Dentate gyrus, granule cell layer	373.29 ± 107.26	15	793.64 ± 159.74	15	2510.59 ± 551.19	12	1950.43 ± 627.18	10	20.4	0.0000404	0.0334	0.856	1.75	0.192		***	*	**		
982	1080	Fasciola cinerea	484.18 ± 230.22	13	692.89 ± 216.02	15	2064.50 ± 503.44	11	1579.94 ± 651.17	8	10.9	0.00195	0.311	0.58	0.825	0.369		*		*		
19	1080	Induseum griseum	66.55 ± 20.23	15	78.42 ± 25.34	12	235.81 ± 80.92	12	159.08 ± 57.06	10	6.88	0.0119	0.293	0.591	0.798	0.376						
822	1090	Retrohippocampal region	76.27 ± 31.20	14	71.30 ± 34.44	13	414.84 ± 184.98	12	306.12 ± 78.27	8	8.1	0.00676	0.493	0.486	0.249	0.621						
909	822	Entorhinal area	33.81 ± 14.20	13	23.78 ± 7.10	12	39.52 ± 23.14	10	50.79 ± 14.55	8	0.996	0.324	0.0124	0.912	0.468	0.498						
918	909	Entorhinal area, lateral part	52.33 ± 21.96	13	38.34 ± 12.27	12	60.87 ± 35.61	10	78.32 ± 22.42	8	0.907	0.347	0.00515	0.943	0.421	0.52						
502	822	Subiculum	457.85 ± 173.38	12	463.21 ± 212.07	10	2475.96 ± 853.31	10	2339.07 ± 608.71	7	14.4	0.000563	0.0733	0.788	0.0187	0.892		*		*		
485000000	822	Prosubiculum	1001.71 ± 472.42	10	806.08 ± 417.98	9	2890.09 ± 912.18	10	2521.16 ± 673.63	7	7.42	0.0104	0.341	0.563	0.0167	0.898						
703	688	Cortical subplate	11.67 ± 5.27	15	7.44 ± 2.25	15	33.72 ± 6.72	12	44.85 ± 5.86	10	32.8	6.5E-07	0.034	0.855	2.25	0.14		*	***	**	****	

583	703	Claustrum	31.89 ± 7.27	15	17.22 ± 5.61	14	93.93 ± 30.71	12	69.55 ± 13.71	10	12.3	0.00102	1.58	0.215	0.0872	0.769	*		**		
942	703	Endopiriform nucleus	8.90 ± 1.87	13	8.70 ± 1.80	13	31.80 ± 8.11	12	46.38 ± 8.63	10	28.5	3.11E-06	0.865	0.357	1.73	0.195	*	***	*	***	
952	942	Endopiriform nucleus, dorsal part	18.61 ± 5.22	13	21.35 ± 6.25	14	59.39 ± 13.09	12	79.66 ± 18.40	10	20.5	0.0000443	0.475	0.494	0.647	0.425	*	**		**	
966	942	Endopiriform nucleus, ventral part	16.14 ± 3.39	13	35.54 ± 15.88	14	70.02 ± 22.92	12	114.22 ± 17.44	10	16.3	0.000206	2.64	0.111	0.578	0.451		***		**	
131	703	Lateral amygdalar nucleus	35.39 ± 15.44	13	20.01 ± 8.78	15	70.01 ± 14.09	12	56.87 ± 10.71	10	7.99	0.00693	1.9	0.174	0.0078	0.93			*		
295	703	Basolateral amygdalar nucleus	17.99 ± 11.30	15	5.41 ± 1.01	13	71.25 ± 11.46	10	98.08 ± 12.73	10	50.3	8.34E-09	0.406	0.527	3.73	0.06	**	****	***	****	
303	295	Basolateral amygdalar nucleus, anterior part	27.41 ± 12.59	15	10.53 ± 3.20	12	182.11 ± 29.04	10	232.34 ± 33.97	10	80.1	2.26E-11	1.14	0.291	2.58	0.115	***	****	****	****	
311	295	Basolateral amygdalar nucleus, posterior part	33.62 ± 26.51	14	11.39 ± 3.17	12	116.90 ± 37.54	10	184.86 ± 17.21	8	24	0.0000164	0.248	0.621	3.14	0.0839		***	*	***	
451	295	Basolateral amygdalar nucleus, ventral part	71.38 ± 58.75	14	8.95 ± 3.05	11	93.49 ± 13.87	9	137.15 ± 29.84	8	2.77	0.104	0.167	0.685	1.55	0.221					
319	703	Basomedial amygdalar nucleus	16.79 ± 8.23	14	5.79 ± 1.38	13	58.64 ± 12.08	10	55.45 ± 10.92	10	28.3	3.57E-06	0.649	0.425	0.206	0.652	**	*	***	**	
327	319	Basomedial amygdalar nucleus, anterior part	28.67 ± 9.27	14	15.31 ± 5.53	12	80.67 ± 27.17	10	68.09 ± 11.54	10	13.3	0.000739	0.599	0.443	0.000733	0.979			*		
334	319	Basomedial amygdalar nucleus, posterior part	38.57 ± 27.60	13	8.97 ± 1.99	11	163.37 ± 31.18	9	158.56 ± 38.99	8	24.9	0.0000144	0.429	0.516	0.205	0.653	**	*	**	**	
780	703	Posterior amygdalar nucleus	29.70 ± 16.76	12	29.28 ± 11.34	12	73.91 ± 27.78	10	124.88 ± 27.95	8	10.9	0.00214	0.748	0.393	1.53	0.223		*		*	
623	567	Cerebral nuclei	8.20 ± 1.83	15	8.23 ± 1.20	15	26.33 ± 4.29	12	29.58 ± 2.91	10	55.9	1.37E-09	0.0393	0.844	0.374	0.544	***	****	****	****	
477	623	Striatum	11.03 ± 2.39	15	10.88 ± 1.47	15	36.84 ± 5.41	12	40.83 ± 3.92	10	67.9	9.59E-11	0.0121	0.913	0.375	0.543	****	****	****	****	
485	477	Striatum dorsal region	11.71 ± 1.98	15	11.62 ± 1.82	15	30.11 ± 5.23	12	28.73 ± 5.48	10	24.5	9.41E-06	0.162	0.689	0.0324	0.858	**	*	**	*	
672	485	Caudoputamen	25.08 ± 3.86	14	24.89 ± 3.49	14	60.22 ± 10.46	12	63.83 ± 9.96	9	27.6	0.000004	0.0284	0.867	0.0728	0.788	**	**	**	**	
493	477	Striatum ventral region	18.55 ± 4.39	15	16.00 ± 2.36	14	58.79 ± 6.41	12	62.97 ± 9.01	10	62.1	3.84E-10	0.0249	0.875	0.371	0.545	***	****	****	****	
56	493	Nucleus accumbens	28.64 ± 6.26	12	22.27 ± 1.91	12	149.21 ± 19.73	12	179.24 ± 16.54	8	112	3.83E-13	0.0903	0.765	1.95	0.17	****	****	****	****	
998	493	Fundus of striatum	16.48 ± 7.86	9	21.59 ± 17.09	6	49.69 ± 23.87	7	74.43 ± 24.33	5	5.32	0.0304	0.653	0.427	0.291	0.595					
754	493	Olfactory tubercle	72.10 ± 19.61	13	61.79 ± 10.43	11	82.80 ± 11.83	9	91.05 ± 14.31	9	1.56	0.22	0.00947	0.923	0.347	0.559					
275	477	Lateral septal complex	13.31 ± 2.76	14	27.97 ± 8.03	12	217.10 ± 33.91	11	242.29 ± 31.38	10	96.7	1.43E-12	1.12	0.295	0.0613	0.806	****	****	****	****	
242	275	Lateral septal nucleus	18.94 ± 3.90	14	44.23 ± 14.98	12	302.46 ± 44.31	11	358.21 ± 51.01	10	91.8	3.11E-12	1.95	0.17	0.239	0.627	****	****	****	****	
258	242	Lateral septal nucleus, rostral (rostroventral) part	39.71 ± 7.94	13	78.51 ± 33.06	11	682.80 ± 93.04	11	785.07 ± 123.41	10	88.1	7.13E-12	1.66	0.204	0.195	0.661	****	****	****	****	
266	242	Lateral septal nucleus, ventral part	25.33 ± 12.20	11	86.68 ± 44.01	6	331.69 ± 77.68	10	492.62 ± 98.12	8	27.8	9.92E-06	4.89	0.0345	0.551	0.464	**	****		**	
333	275	Septohippocampal nucleus	52.76 ± 15.81	14	149.02 ± 46.55	7	477.08 ± 131.21	9	374.90 ± 137.25	6	17.7	0.000198	0.163	0.689	1.32	0.259	**				
278	477	Striatum-like amygdalar nuclei	19.13 ± 8.08	15	15.29 ± 5.72	14	27.18 ± 5.21	12	32.90 ± 6.42	10	3.41	0.0711	0.000143	0.991	0.497	0.484					
23	278	Anterior amygdalar area	45.65 ± 30.06	13	10.06 ± 4.43	9	50.15 ± 21.99	10	80.85 ± 28.71	6	1.37	0.25	0.125	0.726	1.56	0.22					
292	278	Bed nucleus of the accessory olfactory tract	26.54 ± 12.73	5	10.93 ± 5.45	8	10.53 ± 10.53	4	0.00 ± 0.00	4	2.34	0.145	2.07	0.169	0.0855	0.774					
536	278	Central amygdalar nucleus	13.55 ± 4.78	15	8.79 ± 3.61	14	28.15 ± 8.17	11	48.53 ± 11.11	10	15.5	0.000273	0.709	0.404	3.45	0.0696		**		**	
544	536	Central amygdalar nucleus, capsular part	20.64 ± 4.01	14	19.51 ± 7.53	14	87.67 ± 27.05	10	179.49 ± 43.15	10	26.8	5.34E-06	3.03	0.0889	4.5	0.0396		****		****	*
551	536	Central amygdalar nucleus, lateral part	34.65 ± 9.90	14	17.59 ± 7.38	14	57.95 ± 25.49	10	82.58 ± 29.32	10	6.12	0.0173	0.000313	0.986	1.36	0.249					
559	536	Central amygdalar nucleus, medial part	28.99 ± 13.37	15	17.74 ± 7.59	13	45.41 ± 11.56	11	67.77 ± 16.11	10	6.64	0.0133	0.0821	0.776	1.8	0.187				*	
1100	278	Intercalated amygdalar nucleus	24.56 ± 11.42	15	27.85 ± 15.48	13	77.50 ± 24.56	12	118.48 ± 58.08	10	6.24	0.0161	0.468	0.497	0.446	0.507					
403	278	Medial amygdalar nucleus	61.02 ± 30.40	15	42.56 ± 15.57	14	70.43 ± 12.83	12	62.93 ± 13.26	10	0.45	0.506	0.434	0.513	0.0637	0.802					
803	623	Pallidum	10.95 ± 3.33	15	13.69 ± 3.79	14	24.96 ± 8.77	11	31.76 ± 6.76	10	8.14	0.00647	0.618	0.436	0.131	0.719					
818	803	Pallidum, dorsal region	8.80 ± 4.46	15	10.34 ± 6.62	13	3.51 ± 0.84	12	11.72 ± 2.12	10	0.245	0.623	1	0.322	0.542	0.465					
1020	818	Globus pallidus, external segment	21.06 ± 11.51	15	25.02 ± 17.10	13	7.78 ± 1.60	11	24.15 ± 5.38	10	0.391	0.535	0.609	0.439	0.271	0.605					
1030	818	Globus pallidus, internal segment	16.00 ± 5.51	14	19.15 ± 11.89	12	7.70 ± 2.40	10	21.75 ± 4.34	10	0.165	0.687	1.11	0.298	0.532	0.47					
835	803	Pallidum, ventral region	14.28 ± 4.00	15	8.45 ± 2.10	14	21.11 ± 5.53	12	27.03 ± 5.61	10	8.07	0.00664	0.0682	0.795	1.83	0.183				*	
342	835	Substantia innominata	29.16 ± 8.25	15	16.54 ± 4.23	14	40.64 ± 11.01	12	58.74 ± 9.68	9	8.9	0.00455	0.0207	0.886	3.24	0.0785				**	
826	803	Pallidum, medial region	32.88 ± 8.04	14	54.80 ± 10.86	11	129.94 ± 45.83	12	140.21 ± 38.18	9	9.97	0.00295	0.282	0.598	0.0393	0.844					
904	826	Medial septal complex	49.32 ± 12.06	14	82.91 ± 16.02	11	175.64 ± 58.33	12	188.96 ± 52.37	9	9.44	0.00372	0.359	0.553	0.069	0.794					
564	904	Medial septal nucleus	64.33 ± 26.78	9	157.35 ± 64.59	5	470.78 ± 163.36	8	524.95 ± 154.37	7	10.4	0.00353	0.902	0.351	0.0248	0.876		*			
596	904	Diagonal band nucleus	100.10 ± 24.59	14	165.86 ± 34.16	11	314.18 ± 108.74	12	306.71 ± 102.76	9	6.22	0.0166	0.171	0.682	0.247	0.622					
343	8	Brain stem	14.97 ± 7.01	15	12.35 ± 3.10	15	17.63 ± 3.00	12	29.13 ± 6.10	10	3.15	0.0824	0.312	0.579	1.79	0.187					
1130	343	Interbrain	17.40 ± 7.71	15	15.78 ± 4.21	15	21.59 ± 3.43	12	34.09 ± 7.20	10	3.21	0.0795	0.41	0.525	1.35	0.251					
549	1130	Thalamus	20.10 ± 11.44	15	18.26 ± 6.07	15	25.35 ± 3.49	12	43.46 ± 10.29	10	2.84	0.0986	0.469	0.497	1.3	0.26					
864	549	Thalamus, sensory-motor cortex related	17.48 ± 11.66	15	14.61 ± 4.45	15	25.15 ± 4.45	12	42.37 ± 10.76	10	3.93	0.0532	0.318	0.576	1.34	0.253					
637	864	Ventral group of the dorsal thalamus	15.51 ± 10.86	15	11.38 ± 6.16	15	23.34 ± 3.89	12	34.53 ± 6.14	10	3.63	0.0628	0.0446	0.834	0.933	0.339					
629	637	Ventral anterior-lateral complex of the thalamus	9.12 ± 3.13	15	17.55 ± 12.40	13	65.36 ± 18.51	12	69.37 ± 14.29	10	18.6	0.0000851	0.227	0.636	0.0306	0.862	*	**	*	*	
685	637	Ventral medial nucleus of the thalamus	9.14 ± 3.17	15	37.64 ± 21.93	14	53.02 ± 8.69	12	77.09 ± 12.81	10	8.98	0.00434	3.38	0.0724	0.0251	0.875		**			
709	637	Ventral posterior complex of the thalamus	31.99 ± 24.77	15	16.48 ± 8.81	15	31.36 ± 5.32	12	47.66 ± 10.18	10	0.801	0.375	0.0299	0.863	0.969	0.33					
718	709	Ventral posterolateral nucleus of the thalamus	128.35 ± 93.72	15	48.32 ± 25.65	15	56.55 ± 8.03	12	85.67 ± 13.99	10	0.122	0.728	0.342	0.562	0.899	0.348					
725	709	Ventral posterolateral nucleus of the thalamus, parvicellular part	38.35 ± 24.97	11	6.53 ± 2.14	12	135.09 ± 57.56	9	88.12 ± 19.81	7	8.12	0.00728	2.16	0.151	0.0579	0.811			*		
733	709	Ventral posteromedial nucleus of the thalamus	25.91 ± 20.76	14	26.36 ± 12.92	15	64.74 ± 13.91	12	107.63 ± 28.63	10	9.67	0.00318	0.637	0.429	1.24	0.272		*		*	
741	709	Ventral posteromedial nucleus of the thalamus, parvicellular part	6.20 ± 4.23	9	4.38 ± 1.70	11	62.10 ± 16.54	12	52.47 ± 14.93	7	19.2	0.000102	1.63	0.21	0.106	0.747	**		**	*	
564000000	637	Posterior triangular thalamic nucleus	69.49 ± 46.29	9	11.19 ± 7.21	9	32.61 ± 13.28	9	96.76 ± 21.06	7	0.549	0.465	0.00789	0.93	4.99	0.0331					
406	864	Subparafascicular nucleus	44.16 ± 28.24	11	6.49 ± 5.38	13	29.21 ± 9.23	10	52.60 ± 8.26	8	0.809	0.374	0.649	0.426	3.48	0.07					
414	406	Subparafascicular nucleus, magnocellular part	25.76 ± 18.91	6	1.32 ± 1.32	8	56.26 ± 19.56	7	58.22 ± 11.25	6	9.7	0.00488	1.37	0.254	0.868	0.361			*	*	
422	406	Subparafascicular nucleus, parvicellular part	107.27 ± 68.83	9	19.60 ± 15.39	9	52.50 ± 25.20	7	118.87 ± 29.15	7	0.26	0.614	0.219	0.643	3.11	0.0887					
1010	864	Geniculate group, dorsal thalamus	97.46 ± 64.51	14	65.02 ± 23.10	15	94.50 ± 24.48	12	169.97 ± 83.25	9	0.858	0.359	0.0272	0.87	1.11	0.297					
475	1010	Medial geniculate complex	6.88 ± 4.39	8	19.54 ± 9.22	8	32.87 ± 15.76	8	74.48 ± 24.96	7	7.26	0.012	2.92	0.0992	0.951	0.338		*			
1070	475	Medial geniculate complex, dorsal part	29.77 ± 17.79	6	116.39 ± 52.72	7	56.57 ± 31.42	8	402.41 ± 205.49	5	2.9	0.103	5.03	0.0354	2.27	0.146		*			*
1080	475	Medial geniculate complex, ventral part	10.96 ± 5.26	6	56.92 ± 34.16	6	68.73 ± 30.51	8	98.62 ± 27.82	5	3.21	0.0878	1.27	0.272	0.0796	0.781					
1090	475	Medial geniculate complex, medial part	15.67 ± 15.67	8	12.74 ± 5.66	8	69.02 ± 34.05	8	91.69 ± 37.99	7	6.39	0.0176	0.0776	0.783	0.242	0.626					
170	1010	Dorsal part of the lateral geniculate complex	150.07 ± 96.41	14	113.69 ± 38.51	15	169.40 ± 39.77	12	278.64 ± 123.91	9	1.27	0.265	0.0474	0.829	0.884	0.352					
496000000	170	Dorsal part of the lateral geniculate complex, shell	388.57 ± 233.96	13	405.49 ± 168.04	14	432.40 ± 119.55	12	523.24 ± 263.57	9	0.154	0.697	0.0451	0.833	0.0341	0.854					
496000000	170	Dorsal part of the lateral geniculate complex, core	374.58 ± 226.87	14	184.68 ± 62.13	15	373.06 ± 113.51	12	513.57 ± 212.84	9	0.851	0.361	0.165	0.687	0.997	0.323					
496000000	170	Dorsal part of the lateral geniculate complex, ipsilateral zone	99.05 ± 65.13	12	255.88 ± 138.88	14	200.82 ± 74.45	12	805.96 ± 391.03	9	3.11	0.0848	3.3	0.0765	1.57	0.218					
856	549	Thalamus, polymodal association cortex related	30.76 ± 16.73	15	29.17 ± 10.87	15	37.05 ± 4.79	12	64.38 ± 15.46	10	2.25	0.14	0.542	0.465	1.17	0.284					
138	856	Lateral group of the dorsal thalamus	31.62 ± 14.89	14	38.92 ± 13.83	15	59.08 ± 8.95	12	118.55 ± 34.82	10	8.09	0.00659	2.1	0.154	1.99	0.165		*		*	
218	138	Lateral posterior nucleus of the thalamus	143.56 ± 81.82	14	166.37 ± 63.85	15	185.02 ± 37.15	12	441.34 ± 163.92	10	2.97	0.0915	1.66	0.205	1.71	0.198					
1020	138	Posterior complex of the thalamus	16.51 ± 6.34	14	8.77 ± 4.29	15	69.99 ± 12.18	12	102.37 ± 20.01	10	46.4	1.56E-08	0.225	0.637	3.52	0.0668	**	****	***	****	
1030	138	Posterior limiting nucleus of the thalamus	96.89 ± 78.90	10	77.22 ± 47.96	9	87.13 ± 42.16	10	224.90 ± 61.40	8	1.08	0.306	0.827	0.37	1.71	0.2					
325	138	Suprageniculate nucleus	29.14 ± 17.95	6	68.95 ± 28.83	6	50.65 ± 30.22	8	235.01 ± 158.59	5	1.61	0.219	2.33	0.142	1.13	0.299					
561000000	138	Ethmoid nucleus of the thalamus	9.78 ± 5.55	13	11.67 ± 4.13	15	97.74 ± 30.46	12	75.05 ± 18.10	10	20.2	0.0000462	0.805	0.374	0.531	0.47	**		**		
239	856	Anterior group of the dorsal thalamus	70.40 ± 36.10	15	61.45 ± 23.79	13	77.17 ± 12.97	12	131.21 ± 37.69	10	1.41	0.242	0.377	0.542	1.09	0.302					
255	239	Anteroventral nucleus of thalamus	30.60 ± 13.55	13	41.79 ± 26.63	8	121.32 ± 72.85	12	57.63 ± 20.20	8	1.81	0.186	0.297	0.589	0.669	0.418					
127	239	Anteromedial nucleus	4.99 ± 1.51	13	16.19 ± 13.89	8	19.12 ± 7.66	12	18.18 ± 6.40	8	1.57	0.217	0.499	0.484	0.627	0.434					
1100	127	Anteromedial nucleus, dorsal part	9.49 ± 4.06	13	31.83 ± 26.10	8	34.95 ± 12.18	12	37.11 ± 15.84	8	1.57	0.217	0.79	0.38	0.494	0.487					
1100	127	Anteromedial nucleus, ventral part	10.44 ± 3.94	13	34.16 ± 32.01	8	45.82 ± 23.27	12	37.44 ± 13.30	8	1.4	0.244	0.18	0.674	0.658	0.422					
64	239	Anterodorsal nucleus	119.85 ± 60.23	12	176.82 ± 109.31	9	202.86 ± 94.83	11	161.16 ± 49.59	8	0.246	0.623	0.014	0.906	0.346	0.56					
1120	239	Interanteromedial nucleus of the thalamus	3.17 ± 3.17	12	32.88 ± 27.73	8	8.08 ± 3.75	11	40.26 ± 21.36	7	0.171	0.682	4.5	0.0413	0.0072	0.933					
1110	239	Interanterodorsal nucleus of the thalamus	14.57 ± 7.21	12	45.64 ± 40.34	9	49.80 ± 15.76	8	71.45 ± 24.84	8	1.94	0.172	1.26	0.269	0.0427	0.838					

155	239	Lateral dorsal nucleus of thalamus	244.15 ± 128.66	15	148.97 ± 51.10	13	253.47 ± 43.50	12	424.32 ± 152.63	10	1.57	0.217	0.0381	0.846	1.6	0.212				
444	856	Medial group of the dorsal thalamus	11.02 ± 5.45	15	14.62 ± 7.00	14	32.40 ± 9.11	12	45.19 ± 13.31	10	9.21	0.00391	0.664	0.419	0.294	0.59		*			
59	444	Intermediodorsal nucleus of the thalamus	13.77 ± 10.14	14	12.77 ± 6.75	14	34.49 ± 12.01	12	68.80 ± 37.54	10	4.59	0.0374	0.542	0.465	1.02	0.319					
362	444	Mediodorsal nucleus of thalamus	17.50 ± 7.24	15	37.72 ± 17.02	14	68.41 ± 23.15	12	88.23 ± 31.48	10	6.76	0.0124	0.927	0.341	0.000103	0.992					
366	444	Submedial nucleus of the thalamus	24.80 ± 17.01	14	15.06 ± 8.70	13	70.19 ± 20.68	11	139.98 ± 36.44	9	16.1	0.000234	1.11	0.298	3.75	0.0595		**		***	
1080	444	Perireunensis nucleus	61.28 ± 32.99	15	65.79 ± 36.80	13	33.97 ± 11.08	12	39.51 ± 12.06	10	0.87	0.356	0.0332	0.856	0.000325	0.986					
571	856	Midline group of the dorsal thalamus	29.85 ± 14.81	15	26.67 ± 10.35	15	30.75 ± 7.40	12	31.47 ± 8.98	10	0.0552	0.815	0.0207	0.886	0.0279	0.868					
149	571	Paraventricular nucleus of the thalamus	92.30 ± 54.15	15	81.41 ± 36.71	15	63.02 ± 27.99	12	59.50 ± 27.61	10	0.378	0.542	0.0257	0.873	0.00769	0.93					
15	571	Parataenial nucleus	12.58 ± 5.33	10	1.65 ± 1.65	6	23.42 ± 9.77	7	17.34 ± 13.58	4	2.7	0.114	1.36	0.256	0.0926	0.764					
181	571	Nucleus of reuniens	44.35 ± 17.17	15	36.03 ± 14.26	13	52.47 ± 16.04	12	77.39 ± 33.57	10	1.35	0.251	0.0914	0.764	0.679	0.414					
561000000	571	Xiphoid thalamic nucleus	47.39 ± 15.76	15	59.45 ± 22.53	13	74.23 ± 24.04	12	103.66 ± 26.07	10	2.56	0.117	0.796	0.377	0.158	0.693					
51	856	Intralaminar nuclei of the dorsal thalamus	8.53 ± 3.34	15	12.83 ± 5.35	15	35.21 ± 5.39	12	64.34 ± 12.04	10	35.6	2.82E-07	4.2	0.0459	3.7	0.0604	*	****		****	*
189	51	Rhomboid nucleus	9.13 ± 3.94	15	30.63 ± 23.71	13	66.87 ± 19.53	12	154.40 ± 54.69	10	10.7	0.00204	3.43	0.0704	1.49	0.228		**		*	
599	51	Central medial nucleus of the thalamus	5.54 ± 1.87	15	23.87 ± 10.81	14	41.27 ± 11.24	12	78.72 ± 30.24	10	9.68	0.00316	3.16	0.0819	0.442	0.509		**			
907	51	Paracentral nucleus	6.53 ± 2.92	14	16.57 ± 9.33	13	87.34 ± 20.90	11	95.51 ± 15.27	10	39.4	1.31E-07	0.485	0.49	0.00542	0.942	***	****	**	***	
575	51	Central lateral nucleus of the thalamus	42.14 ± 22.93	14	53.59 ± 20.49	14	46.98 ± 12.85	12	144.27 ± 27.95	10	4.45	0.0405	4.8	0.0336	3.93	0.0534		*		*	*
930	51	Parafascicular nucleus	9.96 ± 3.89	11	13.73 ± 8.31	10	90.52 ± 13.59	10	186.55 ± 35.66	7	60	5.24E-09	5.49	0.0252	8.57	0.00607	**	****	**	****	**
561000000	51	Posterior intralaminar thalamic nucleus	68.20 ± 53.92	4	35.16 ± 35.16	4	94.92 ± 77.19	5	85.52 ± 49.01	5	0.426	0.525	0.115	0.739	0.04	0.844					
262	856	Reticular nucleus of the thalamus	147.28 ± 98.89	15	150.34 ± 69.44	14	67.19 ± 9.27	12	88.68 ± 12.28	10	1.02	0.319	0.034	0.855	0.0168	0.897					
1010	856	Geniculate group, ventral thalamus	204.26 ± 149.45	15	131.01 ± 85.05	13	131.50 ± 42.87	12	147.24 ± 56.70	9	0.0911	0.764	0.105	0.748	0.192	0.663					
27	1010	Intergeniculate leaflet of the lateral geniculate complex	699.24 ± 651.07	9	337.85 ± 170.18	12	152.71 ± 67.90	10	634.83 ± 297.17	8	0.125	0.726	0.0174	0.896	1.5	0.23					
564000000	1010	Intermediate geniculate nucleus	0.00 ± 0.00	4	625.34 ± 587.56	6	66.51 ± 31.89	5	114.98 ± 83.88	4	0.36	0.558	1	0.333	0.551	0.47					
178	1010	Ventral part of the lateral geniculate complex	422.46 ± 314.26	13	247.26 ± 178.69	15	264.47 ± 87.31	12	272.06 ± 109.51	9	0.113	0.738	0.186	0.668	0.185	0.669					
321	1010	Subgeniculate nucleus	218.90 ± 79.81	7	258.83 ± 212.47	10	244.00 ± 176.92	4	160.52 ± 80.50	6	0.0692	0.795	0.00112	0.974	0.108	0.745					
958	856	Epithalamus	49.64 ± 21.98	15	69.94 ± 33.29	15	46.02 ± 15.49	12	76.87 ± 16.73	10	0.00297	0.957	0.971	0.329	0.0431	0.837					
483	958	Medial habenula	92.77 ± 51.22	15	129.91 ± 50.93	15	113.12 ± 37.60	12	170.17 ± 41.54	10	0.375	0.543	0.847	0.362	0.0418	0.839					
186	958	Lateral habenula	99.49 ± 41.89	15	187.00 ± 133.69	14	68.20 ± 31.88	12	135.10 ± 34.78	10	0.254	0.616	0.984	0.326	0.0161	0.9					
1100	1130	Hypothalamus	28.16 ± 12.18	14	25.05 ± 5.54	14	32.63 ± 8.05	12	44.52 ± 8.01	10	1.62	0.21	0.106	0.746	0.676	0.415					
157	1100	Periventricular zone	72.81 ± 35.11	14	27.51 ± 8.36	14	27.19 ± 13.82	12	76.25 ± 22.27	10	0.00132	0.971	0.029	0.866	4.09	0.049					
390	157	Supraoptic nucleus	47.81 ± 16.96	10	35.43 ± 21.53	7	97.29 ± 36.14	11	44.67 ± 33.88	6	1.32	0.259	1.25	0.273	0.434	0.515					
38	157	Paraventricular hypothalamic nucleus	35.09 ± 15.33	13	30.70 ± 15.32	8	23.50 ± 5.32	12	44.11 ± 12.91	8	0.0201	0.888	0.356	0.554	0.896	0.35					
30	157	Periventricular hypothalamic nucleus, anterior part	9.79 ± 6.67	8	63.64 ± 54.52	6	1.74 ± 1.74	7	35.25 ± 27.81	5	0.367	0.551	2.6	0.121	0.134	0.718					
118	157	Periventricular hypothalamic nucleus, intermediate part	38.99 ± 21.20	13	22.20 ± 8.93	14	10.03 ± 2.78	12	41.25 ± 18.36	10	0.156	0.695	0.121	0.73	2.7	0.108					
223	157	Arcuate hypothalamic nucleus	302.37 ± 132.61	13	85.80 ± 26.59	13	251.83 ± 207.61	11	279.64 ± 84.26	9	0.245	0.623	0.774	0.384	0.87	0.356					
141	1100	Periventricular region	63.80 ± 32.39	15	44.58 ± 24.72	15	23.24 ± 6.33	12	40.48 ± 12.00	10	0.899	0.348	0.0136	0.908	0.549	0.462					
830	141	Dorsomedial nucleus of the hypothalamus	98.32 ± 60.62	12	57.27 ± 21.03	13	23.46 ± 4.79	11	41.48 ± 13.08	9	1.72	0.197	0.111	0.74	0.694	0.41					
126	141	Periventricular hypothalamic nucleus, posterior part	312.33 ± 199.14	9	148.59 ± 112.61	9	97.40 ± 49.60	6	147.55 ± 75.43	8	0.52	0.477	0.328	0.571	0.599	0.445					
347	141	Subparaventricular zone	33.91 ± 23.07	13	26.96 ± 14.59	8	10.00 ± 3.96	12	28.46 ± 12.82	8	0.697	0.409	0.0947	0.76	0.548	0.464					
286	141	Suprachiasmatic nucleus	40.87 ± 23.44	10	38.91 ± 22.75	6	12.61 ± 8.36	9	71.73 ± 60.84	4	0.0929	0.763	0.863	0.362	1.3	0.265					
467	1100	Hypothalamic medial zone	40.12 ± 18.14	15	36.63 ± 9.43	15	50.24 ± 17.65	12	50.79 ± 8.62	10	0.649	0.424	0.025	0.875	0.0183	0.893					
88	467	Anterior hypothalamic nucleus	32.01 ± 13.11	15	27.06 ± 8.29	11	46.07 ± 10.36	12	46.34 ± 17.25	8	1.67	0.203	0.0597	0.808	0.042	0.839					
331	467	Mammillary body	72.82 ± 26.42	9	73.02 ± 18.50	12	101.54 ± 52.67	10	107.91 ± 27.22	7	0.831	0.368	0.00365	0.952	0.00785	0.93					
210	331	Lateral mammillary nucleus	108.63 ± 23.21	6	69.04 ± 40.90	7	46.56 ± 27.81	7	116.18 ± 20.62	7	0.0316	0.861	0.343	0.564	3.37	0.0794					
491	331	Medial mammillary nucleus	57.56 ± 15.88	7	25.01 ± 10.45	9	105.50 ± 70.20	6	147.44 ± 30.48	7	6.89	0.0146	0.00108	0.974	1.2	0.284					
732	491	Medial mammillary nucleus, median part	80.71 ± 41.99	7	67.75 ± 43.24	8	186.65 ± 131.35	6	169.94 ± 63.25	7	2.09	0.161	0.0388	0.845	0.00068	0.979					
607000000	491	Medial mammillary nucleus, lateral part	154.69 ± 40.16	7	77.25 ± 40.46	9	201.72 ± 127.50	6	257.59 ± 71.80	7	2.91	0.1	0.0826	0.776	0.886	0.356					
607000000	491	Medial mammillary nucleus, medial part	94.27 ± 42.52	7	26.23 ± 21.29	9	215.63 ± 140.85	6	414.54 ± 102.92	6	11	0.00287	0.16	0.693	2.85	0.104		*		**	
525	331	Supramammillary nucleus	428.91 ± 196.29	8	406.35 ± 130.90	9	807.88 ± 424.70	8	588.50 ± 212.44	7	1.14	0.294	0.255	0.618	0.14	0.711					
557	331	Tuberomammillary nucleus	35.13 ± 24.15	8	68.58 ± 25.03	11	18.54 ± 6.66	8	13.85 ± 2.31	6	2.74	0.108	1.15	0.292	0.797	0.379					
1130	557	Tuberomammillary nucleus, dorsal part	50.00 ± 50.00	8	76.47 ± 39.37	7	23.65 ± 11.58	5	0.00 ± 0.00	5	1.56	0.225	0.0142	0.906	0.38	0.544					
1	557	Tuberomammillary nucleus, ventral part	28.87 ± 14.55	7	134.14 ± 62.53	9	40.66 ± 15.37	8	32.81 ± 5.69	6	1.2	0.284	2.19	0.151	2	0.169					
980	467	Dorsal premammillary nucleus	283.84 ± 262.85	7	78.71 ± 29.34	7	27.32 ± 19.61	5	83.80 ± 12.85	6	0.62	0.44	0.408	0.53	0.747	0.397					
1000	467	Ventral premammillary nucleus	266.08 ± 241.83	8	120.94 ± 50.97	10	40.36 ± 13.60	8	76.88 ± 33.43	7	1.18	0.287	0.17	0.683	0.545	0.466					
63	467	Paraventricular hypothalamic nucleus, descending division	62.09 ± 32.35	13	55.39 ± 26.26	11	18.38 ± 8.33	10	48.67 ± 17.75	9	1.08	0.305	0.134	0.716	0.525	0.473					
693	467	Ventromedial hypothalamic nucleus	73.58 ± 35.94	14	26.33 ± 13.13	12	69.23 ± 42.28	11	55.33 ± 11.99	9	0.12	0.731	1.12	0.297	0.282	0.599					
946	467	Posterior hypothalamic nucleus	126.67 ± 72.67	14	106.33 ± 57.44	14	97.26 ± 32.76	10	92.86 ± 29.33	8	0.13	0.72	0.0476	0.828	0.017	0.897					
290	1100	Hypothalamic lateral zone	23.87 ± 8.50	15	23.20 ± 5.92	15	44.06 ± 8.71	12	61.49 ± 12.83	10	10.6	0.00206	0.423	0.519	1.05	0.311		*		*	
194	290	Lateral hypothalamic area	50.26 ± 16.54	15	69.22 ± 13.30	15	93.28 ± 21.54	10	105.23 ± 24.49	10	4.53	0.0387	0.788	0.379	0.0356	0.851					
226	290	Lateral preoptic area	77.90 ± 34.93	6	43.94 ± 31.64	6	117.74 ± 43.78	6	207.08 ± 137.80	4	2.47	0.133	0.0361	0.851	1.04	0.322					
364	290	Parasubthalamic nucleus	58.49 ± 36.83	12	34.61 ± 11.23	12	54.60 ± 16.58	10	110.18 ± 46.03	8	1.27	0.266	0.0774	0.782	1.81	0.187					
576000000	290	Perifornical nucleus	35.07 ± 10.31	15	41.01 ± 12.34	13	125.40 ± 37.00	12	75.59 ± 21.68	9	8.9	0.00459	0.843	0.364	1.59	0.214	*		*		
173	290	Retrochiasmatic area	54.11 ± 33.29	13	3.40 ± 3.40	5	29.64 ± 7.59	10	48.12 ± 24.37	6	0.00489	0.945	0.28	0.601	1.35	0.255					
470	290	Subthalamic nucleus	3.38 ± 2.00	12	7.28 ± 2.38	12	51.64 ± 13.72	12	94.00 ± 27.40	8	27.1	6.19E-06	1.34	0.254	2.31	0.136	*	***		***	
614	290	Tuberal nucleus	78.43 ± 41.56	14	39.17 ± 15.23	13	48.96 ± 18.53	10	86.11 ± 21.49	9	0.0548	0.816	0.0703	0.792	1.65	0.207					
797	290	Zona incerta	35.59 ± 12.19	15	43.43 ± 17.94	15	110.49 ± 20.98	12	142.45 ± 33.21	10	17.7	0.000113	0.488	0.488	0.344	0.56		**		**	
804	797	Fields of Forel	21.56 ± 14.47	10	6.13 ± 2.17	10	116.28 ± 27.14	8	159.17 ± 40.25	8	28.9	6.73E-06	0.209	0.65	1.6	0.215	*	**	**	***	
10700	1100	Median eminence	370.20 ± 175.50	12	123.49 ± 60.37	13	86.38 ± 37.80	9	527.81 ± 229.09	9	0.209	0.65	0.08	0.779	5.67	0.0222					
313	343	Midbrain	46.07 ± 33.32	13	14.19 ± 5.33	12	26.34 ± 7.51	12	70.78 ± 26.74	8	0.365	0.549	0.0001	0.992	2.89	0.0969					
339	313	Midbrain, sensory related	7.20 ± 4.90	11	18.73 ± 6.44	10	55.32 ± 31.88	10	17.40 ± 7.05	7	2.21	0.146	0.455	0.505	1.92	0.175					
600000000	339	Subcommissural organ	51.95 ± 51.95	11	0.00 ± 0.00	8	130.94 ± 62.43	10	0.00 ± 0.00	7	0.943	0.339	3.47	0.0718	0.672	0.418					
323	313	Midbrain, motor related	49.73 ± 43.16	13	20.43 ± 8.81	13	26.14 ± 6.98	12	74.73 ± 31.40	8	0.153	0.698	0.00966	0.922	1.93	0.172					
381	323	Substantia nigra, reticular part	35.68 ± 32.20	5	17.16 ± 14.10	8	14.96 ± 6.77	8	23.90 ± 7.25	7	0.143	0.709	0.0314	0.861	0.833	0.37					
749	323	Ventral tegmental area	20.19 ± 16.67	4	12.90 ± 12.90	4	32.28 ± 24.04	7	52.31 ± 8.76	4	1.3	0.272	0.0508	0.825	0.411	0.531					
128	323	Midbrain reticular nucleus	18.86 ± 14.81	8	13.13 ± 7.73	9	74.69 ± 34.09	9	97.43 ± 16.67	6	9.59	0.00441	0.00505	0.944	0.407	0.529					
795	323	Periaqueductal gray	60.32 ± 44.36	13	19.42 ± 10.33	13	25.90 ± 6.13	12	34.38 ± 6.72	8	0.225	0.638	0.505	0.481	0.854	0.361					
50	795	Precommissural nucleus	129.02 ± 101.46	10	54.64 ± 41.27	12	49.27 ± 14.22	12	44.49 ± 16.18	8	0.727	0.399	0.407	0.527	0.394	0.534					
1100	323	Pretectal region	107.69 ± 100.48	11	388.61 ± 349.69	11	53.19 ± 18.65	11	266.43 ± 193.53	8	0.164	0.688	1.49	0.23	0.0255	0.874					
215	1100	Anterior pretectal nucleus	235.65 ± 224.06	11	753.95 ± 700.98	11	109.58 ± 37.15	11	559.69 ± 385.94	8	0.136	0.714	1.38	0.247	0.00635	0.937					
628	1100	Nucleus of the optic tract	37.68 ± 19.92	8	531.48 ± 325.14	7	242.80 ± 128.48	8	48.75 ± 27.27	7	0.483	0.493	0.805	0.378	4.24	0.0497					
634	1100	Nucleus of the posterior commissure	169.70 ± 152.90	9	17.55 ± 5.16	7	25.81 ± 16.08	8	50.26 ± 17.86	7	0.499	0.486	0.561	0.46	0.942	0.34					
706	1100	Olivary pretectal nucleus	169.57 ± 123.91	8	67.76 ± 30.35	7	52.99 ± 24.74	7	175.73 ± 133.90	7	0.00678	0.935	0.00467	0.946	1.36	0.255					
1060	1100	Posterior pretectal nucleus	7.54 ± 3.83	6	22.58 ± 11.42	6	34.37 ± 15.43	8	62.39 ± 18.84	5	5.54	0.0284	1.65	0.212	0.217	0.646					
549000000	1100	Retroparafascicular nucleus	0.00 ± 0.00	6	1.60 ± 1.60	7	134.75 ± 41.64	5	54.99 ± 17.19	6	22.4	0.000127	3.35	0.0823	4.49	0.0469	***		***		*
348	313	Midbrain, behavioral state related	11.44 ± 10.18	5	16.54 ± 16.05	6	20.37 ± 15.32	7	5.30 ± 3.28	4	0.000146	0.99	0.105	0.75	0.487	0.494					
374	348	Substantia nigra, compact part	22.87 ± 20.36	5	0.00 ± 0.00	4	40.83 ± 30.63	7	14.99 ± 12.70	4	0.445	0.514	1.03	0.324	0.00337	0.954					

967	1010	cranial nerves	26.62 ± 11.38	15	25.26 ± 7.47	15	17.63 ± 6.33	12	57.56 ± 17.62	10	0.942	0.337	2.09	0.155	3.58	0.0644					
1010	997	fiber tracts	49.43 ± 11.92	15	44.88 ± 8.45	15	143.96 ± 21.77	12	179.27 ± 25.61	10	47	1.23E-08	0.194	0.662	1.45	0.235	***	****	***	****	
21	840	lateral olfactory tract, general	52.27 ± 36.18	15	18.84 ± 7.78	15	7.62 ± 2.19	12	29.99 ± 18.63	10	0.632	0.431	0.162	0.689	1.48	0.23					
665	21	lateral olfactory tract, body	104.53 ± 72.36	15	37.68 ± 15.56	15	15.23 ± 4.37	12	59.97 ± 37.26	10	0.632	0.431	0.162	0.689	1.48	0.23					
840	967	olfactory nerve	24.71 ± 15.28	15	10.31 ± 3.73	15	6.65 ± 1.18	12	26.08 ± 12.27	10	0.0383	0.846	0.0000466	0.995	2.64	0.111					
848	967	optic nerve	64.81 ± 30.68	15	80.74 ± 25.95	15	39.40 ± 17.31	12	137.09 ± 48.33	10	0.183	0.671	2.62	0.112	1.72	0.196					
900	840	anterior commissure, olfactory limb	16.92 ± 8.35	15	13.80 ± 4.48	14	17.62 ± 3.17	12	52.60 ± 19.85	9	3.73	0.0597	1.65	0.206	4.18	0.0466				*	
117	848	optic chiasm	12.96 ± 8.15	11	3.88 ± 3.88	8	0.00 ± 0.00	10	23.29 ± 18.43	5	0.016	0.9	0.336	0.567	4.08	0.0525					
125	848	optic tract	138.35 ± 66.54	15	131.67 ± 52.32	15	54.57 ± 26.46	12	282.88 ± 116.67	10	0.163	0.688	1.83	0.182	2.97	0.0915					
158	832	posterior commissure	72.11 ± 48.91	10	13.61 ± 10.77	7	20.42 ± 16.85	9	35.67 ± 19.29	7	0.386	0.539	0.482	0.493	1.24	0.275					
832	967	oculomotor nerve	36.05 ± 24.45	10	8.85 ± 5.08	9	10.21 ± 8.43	9	18.24 ± 9.27	7	0.405	0.529	0.493	0.488	1.28	0.266					
916	848	brachium of the superior colliculus	617.44 ± 558.13	10	652.16 ± 284.02	10	336.43 ± 103.58	10	641.74 ± 297.80	8	0.184	0.671	0.227	0.637	0.142	0.709					
792	967	dorsal roots	6.76 ± 3.21	10	2.84 ± 1.45	11	20.62 ± 7.65	10	28.57 ± 9.56	8	11.2	0.00198	9.38E-06	0.998	1.03	0.318				*	
932	792	cervicothalamic tract	10.15 ± 4.81	10	4.27 ± 2.17	11	30.94 ± 11.48	10	42.86 ± 14.34	8	11.2	0.00198	9.38E-06	0.998	1.03	0.318				*	
697	932	medial lemniscus	20.29 ± 9.63	10	8.53 ± 4.34	11	61.87 ± 22.96	10	85.71 ± 28.68	8	11.2	0.00198	9.38E-06	0.998	1.03	0.318				*	
579	956	external capsule	33.99 ± 8.40	15	32.65 ± 8.09	15	84.35 ± 19.36	12	200.01 ± 53.34	10	20.4	0.0000415	3.48	0.0681	6.21	0.0162		****		****	*
776	983	corpus callosum	106.41 ± 25.89	15	109.07 ± 30.58	15	289.64 ± 47.01	12	365.30 ± 49.34	10	34	4.52E-07	0.413	0.523	0.953	0.334	**	***	**	***	
956	776	corpus callosum, anterior forceps	16.65 ± 4.03	15	22.87 ± 6.44	15	34.18 ± 8.74	12	88.50 ± 27.18	10	11.1	0.00164	4.28	0.044	3.95	0.0525		***		**	*
983	1010	lateral forebrain bundle system	63.46 ± 15.03	15	55.80 ± 13.68	15	141.84 ± 21.70	12	179.96 ± 22.64	10	31.2	1.07E-06	0.164	0.687	1.63	0.208	*	***	**	***	
485000000	1010	supra-callosal cerebral white matter	184.43 ± 69.10	15	170.87 ± 80.90	15	319.85 ± 75.99	12	557.55 ± 87.73	10	10.3	0.0024	1.06	0.307	2.5	0.121		*		**	
6	784	internal capsule	77.42 ± 37.50	15	16.14 ± 5.50	14	23.52 ± 4.81	12	84.33 ± 21.52	10	0.0161	0.9	0.137	0.713	6.31	0.0155					
784	983	corticospinal tract	41.70 ± 19.71	15	13.93 ± 4.73	15	22.53 ± 7.83	12	58.88 ± 16.61	10	0.64	0.428	0.00841	0.927	5.23	0.0267					
924	784	cerebal peduncle	107.17 ± 101.58	7	69.17 ± 31.55	9	173.06 ± 83.99	8	286.44 ± 132.42	7	2.61	0.118	0.0613	0.806	0.725	0.402					
964	776	corpus callosum, extreme capsule	31.57 ± 12.45	15	34.44 ± 15.81	15	109.01 ± 37.37	12	135.05 ± 39.98	10	11.5	0.0014	0.114	0.737	0.197	0.659		*			
971	776	corpus callosum, posterior forceps	705.24 ± 147.11	11	522.06 ± 188.66	12	1441.98 ± 443.04	10	1858.05 ± 399.63	9	11.6	0.00155	0.0164	0.899	0.977	0.329				*	
986	776	corpus callosum, splenium	644.85 ± 189.46	13	513.17 ± 173.47	15	1340.09 ± 249.82	12	1382.18 ± 338.32	9	11.4	0.00154	0.378	0.542	0.142	0.708			*		
1110	776	genu of corpus callosum	25.53 ± 11.81	14	35.87 ± 19.28	11	34.70 ± 12.04	11	63.60 ± 20.90	8	1.19	0.282	1.31	0.259	0.342	0.562					
485000000	776	corpus callosum, body	189.01 ± 49.52	15	195.44 ± 54.88	13	570.95 ± 115.50	12	802.53 ± 201.16	10	20.9	0.0000366	0.906	0.346	1.12	0.296		**		**	
896	983	thalamus related	152.34 ± 50.45	15	92.01 ± 20.61	15	212.24 ± 41.23	12	238.98 ± 44.53	10	6.06	0.0175	0.481	0.491	1.12	0.295					
1090	896	external medullary lamina of the thalamus	125.34 ± 102.16	15	76.67 ± 37.57	14	58.85 ± 15.71	12	72.31 ± 23.55	10	0.326	0.571	0.103	0.749	0.223	0.639					
102	760	nigrostriatal tract	15.19 ± 8.77	15	29.43 ± 13.65	14	78.72 ± 17.50	12	77.21 ± 22.12	10	13.8	0.000543	0.155	0.696	0.27	0.606	*	*			
760	1000	cerebral nuclei related	7.60 ± 4.38	15	14.72 ± 6.82	14	39.36 ± 8.75	12	38.60 ± 11.06	10	13.8	0.000543	0.155	0.696	0.27	0.606	*	*			
1000	1010	extrapyramidal fiber systems	5.06 ± 2.92	15	9.62 ± 4.37	15	26.24 ± 5.83	12	25.07 ± 7.52	10	13.5	0.000611	0.0708	0.791	0.323	0.572	*	*			
485000000	896	optic radiation	357.52 ± 111.50	15	208.58 ± 51.65	15	523.66 ± 101.82	12	609.47 ± 112.86	9	7.92	0.00711	0.574	0.452	1.45	0.235				*	
485000000	896	auditory radiation	131.47 ± 79.58	14	109.44 ± 66.44	15	123.15 ± 55.00	12	174.76 ± 45.51	8	0.122	0.728	0.00435	0.948	0.272	0.605					
768	991	cerebrum related	92.93 ± 25.51	15	99.39 ± 19.63	15	434.75 ± 83.75	12	496.60 ± 108.00	10	37.6	1.6E-07	0.0511	0.822	0.212	0.647	***	***	**	***	
884	768	amygdalar capsule	32.40 ± 17.76	15	25.53 ± 10.91	15	60.16 ± 14.57	12	104.35 ± 18.80	10	10.9	0.00185	0.626	0.433	2.62	0.112		*		**	
908	768	anterior commissure, temporal limb	7.19 ± 5.16	9	0.86 ± 0.86	6	31.99 ± 11.08	6	51.10 ± 32.91	5	6.52	0.0181	0.222	0.642	0.809	0.378					
940	768	cingulum bundle	141.13 ± 37.11	15	180.21 ± 86.67	15	273.44 ± 61.72	12	453.27 ± 71.35	10	8.51	0.00535	1.77	0.19	1.06	0.308		*		*	
991	1010	medial forebrain bundle system	62.75 ± 17.51	15	66.32 ± 12.53	15	287.98 ± 55.23	12	330.40 ± 70.81	10	37.9	1.43E-07	0.0542	0.817	0.24	0.626	***	***	***	***	
1100	768	fornix system	152.91 ± 47.98	15	160.32 ± 29.76	15	757.87 ± 147.25	12	848.15 ± 188.38	10	37.5	1.61E-07	0.0173	0.896	0.155	0.696	***	***	***	***	
436	737	columns of the fornix	48.58 ± 23.04	15	36.19 ± 10.95	14	287.14 ± 102.22	12	236.79 ± 53.87	10	16.2	0.00021	0.408	0.526	0.119	0.732	*		*		
466	1100	alveus	326.61 ± 95.12	15	424.47 ± 62.45	15	1571.15 ± 216.91	12	1842.55 ± 369.51	10	50.4	5.13E-09	0.368	0.547	0.215	0.645	***	****	***	****	
530	1100	dorsal fornix	286.79 ± 168.54	6	284.58 ± 220.13	4	1646.90 ± 934.02	4	1486.60 ± 931.92	4	4.58	0.0505	0.00836	0.928	0.0171	0.898					
603	1100	fimbria	356.68 ± 142.39	15	363.15 ± 89.01	15	2032.74 ± 475.59	12	2165.30 ± 620.41	10	26	5.72E-06	0.00274	0.958	0.0342	0.854	**	**	**	**	
618	1100	hippocampal commissures	182.91 ± 42.44	15	162.04 ± 51.79	15	502.90 ± 123.86	12	830.63 ± 103.58	10	36.4	2.24E-07	1.73	0.195	4.68	0.0354	*	****	*	****	*
737	1100	postcommissural fornix	24.29 ± 11.52	15	18.10 ± 5.48	14	143.57 ± 51.11	12	118.40 ± 26.93	10	16.2	0.00021	0.408	0.526	0.119	0.732	*		*		
54	824	medial forebrain bundle	160.47 ± 81.63	8	43.51 ± 20.58	10	235.56 ± 124.46	9	573.40 ± 276.38	7	4.74	0.0375	0.204	0.655	2.74	0.109					
301	768	stria terminalis	68.75 ± 21.35	15	50.88 ± 20.47	15	284.35 ± 162.65	12	300.84 ± 130.15	10	6.29	0.0156	0.0225	0.881	0.0344	0.854					
443	618	dorsal hippocampal commissure	359.91 ± 85.84	15	325.07 ± 103.37	15	1009.13 ± 246.88	12	1661.26 ± 207.17	10	36.8	1.99E-07	1.77	0.19	4.56	0.0379	*	****	*	****	*
824	991	hypothalamus related	33.22 ± 15.61	15	27.79 ± 6.72	15	43.15 ± 11.09	12	82.10 ± 28.26	10	3.91	0.0536	0.591	0.446	2.01	0.163					
485000000	301	commissural branch of stria terminalis	11.08 ± 8.12	14	37.22 ± 21.98	12	59.80 ± 20.95	12	76.82 ± 36.39	10	4.11	0.0486	0.972	0.33	0.0428	0.837					
349	824	supraoptic commissures	99.82 ± 47.49	14	84.73 ± 63.76	12	28.64 ± 17.10	12	51.04 ± 21.70	10	1.49	0.229	0.00305	0.956	0.178	0.675					
46	824	mammillary related	20.62 ± 8.13	14	63.65 ± 19.68	15	33.87 ± 11.53	12	52.08 ± 11.87	10	0.00802	0.929	5.05	0.0293	0.729	0.398					
681	46	mammillotegmental tract	58.53 ± 34.35	9	23.20 ± 19.90	10	142.86 ± 95.98	8	32.43 ± 14.68	6	0.867	0.36	1.9	0.178	0.506	0.483					
690	46	mammillothalamic tract	18.60 ± 7.41	14	55.72 ± 36.17	14	45.87 ± 13.52	12	77.29 ± 19.33	10	1.12	0.296	2.11	0.153	0.015	0.903					
753	46	principal mammillary tract	166.83 ± 88.13	9	580.09 ± 166.03	9	164.50 ± 96.77	9	246.45 ± 83.95	7	1.77	0.193	5.21	0.0297	1.97	0.171					
1080	824	epithalamus related	59.71 ± 32.08	15	27.21 ± 10.86	15	70.30 ± 22.40	12	120.70 ± 50.88	10	2.8	0.101	0.0000426	0.995	1.93	0.171					
595	1080	fasciculus retroflexus	48.10 ± 27.68	14	21.16 ± 11.59	15	63.72 ± 27.60	12	34.49 ± 14.44	10	0.43	0.515	1.73	0.194	0.00268	0.959					
611	1080	habenular commissure	103.13 ± 58.96	13	67.05 ± 34.43	13	108.52 ± 30.46	11	241.58 ± 147.82	8	1.62	0.211	0.117	0.734	1.62	0.21					
802	1080	stria medullaris	167.95 ± 105.45	15	83.99 ± 49.61	13	222.00 ± 77.80	12	394.23 ± 155.43	10	2.93	0.0936	0.0726	0.789	1.62	0.209					
