# Supplementary material for: Hyperactive neuronal networks facilitate tau spread in an Alzheimer’s disease mouse model
Source: bioRxiv. 2024 Dec 3:2024.12.01.625514. Preprint. [Version 2] doi: 10.1101/2024.12.01.625514 (PMC11642807; doi:10.1101/2024.12.01.625514)
Supplement: Supplementary file 2 [file media-2.pdf]

| ID | Parent ID | Region | WT.Sal | n | WT.PTZ | n | 5X.Sal | n | 5X.PTZ | n | Genotype F | Genotype p | Treatment F | Treatment p | Interaction F | Interaction p | p1 | p2 | p3 | p4 | p5 | p6 |
|---|---|---|---|---|---|---|---|---|---|---|---|---|---|---|---|---|---|---|---|---|---|---|
| 8 |  | Basic cell groups and regions | 4.19 ± 1.44 | 15 | 4.22 ± 0.65 | 15 | 10.04 ± 1.81 | 12 | 14.81 ± 1.85 | 10 | 31.6 | 9.32E-07 | 1.37 | 0.247 | 2.7 | 0.107 |  | * | **** | * | **** |  |
| 567 | 8 | Cerebrum | 4.25 ± 1.24 | 15 | 4.79 ± 0.71 | 15 | 12.25 ± 2.16 | 12 | 17.21 ± 2.12 | 10 | 42.9 | 3.62E-08 | 1.65 | 0.205 | 2.05 | 0.159 |  | ** | **** | ** | **** |  |
| 688 | 567 | Cerebral cortex | 4.60 ± 1.32 | 15 | 5.09 ± 0.91 | 15 | 14.25 ± 2.87 | 12 | 20.12 ± 2.81 | 10 | 38.7 | 1.16E-07 | 1.3 | 0.261 | 1.88 | 0.177 |  | ** | **** | ** | **** |  |
| 695 | 688 | Cortical plate | 5.42 ± 1.38 | 15 | 6.28 ± 1.16 | 15 | 18.42 ± 4.07 | 12 | 23.84 ± 3.42 | 10 | 35.6 | 2.79E-07 | 0.705 | 0.405 | 0.803 | 0.375 |  | ** | **** | ** | *** |  |
| 315 | 695 | Isocortex | 4.57 ± 1.26 | 15 | 4.93 ± 1.28 | 15 | 11.53 ± 1.94 | 10 | 21.57 ± 4.37 | 10 | 28.2 | 0.0000031 | 3.77 | 0.0584 | 4.74 | 0.0346 |  |  | **** |  | **** | * |
| 500 | 315 | Somatomotor areas | 3.27 ± 0.81 | 15 | 6.12 ± 2.22 | 15 | 17.63 ± 3.46 | 10 | 23.49 ± 6.20 | 10 | 24.3 | 0.0000111 | 1.66 | 0.205 | 0.218 | 0.643 |  | * | *** |  | ** |  |
| 985 | 500 | Primary motor area | 5.22 ± 1.33 | 15 | 7.08 ± 2.35 | 15 | 24.37 ± 5.65 | 10 | 37.62 ± 9.84 | 9 | 28 | 3.52E-06 | 1.55 | 0.22 | 1.49 | 0.229 |  | * | **** |  | *** |  |
| 993 | 500 | Secondary motor area | 4.74 ± 1.34 | 13 | 15.44 ± 7.16 | 13 | 31.73 ± 5.20 | 10 | 44.49 ± 10.09 | 9 | 19.5 | 0.0000708 | 3.01 | 0.0903 | 0.0264 | 0.872 |  | * | *** |  | * |  |
| 453 | 315 | Somatosensory areas | 3.77 ± 1.03 | 15 | 4.88 ± 1.41 | 15 | 10.63 ± 1.88 | 10 | 17.38 ± 2.93 | 9 | 30.5 | 1.59E-06 | 3.26 | 0.0775 | 2.63 | 0.112 |  | * | **** |  | **** |  |
| 322 | 453 | Primary somatosensory area | 4.49 ± 1.24 | 15 | 5.16 ± 1.33 | 15 | 13.17 ± 2.48 | 10 | 21.91 ± 3.69 | 9 | 37.1 | 2.28E-07 | 2.98 | 0.0909 | 3.82 | 0.0569 |  | * | **** | * | **** | * |
| 353 | 322 | Primary somatosensory area, nose | 2.53 ± 1.22 | 9 | 10.19 ± 6.34 | 7 | 14.55 ± 3.92 | 8 | 40.70 ± 22.49 | 7 | 3.63 | 0.0674 | 2.58 | 0.12 | 0.74 | 0.397 |  |  |  |  |  |  |
| 329 | 322 | Primary somatosensory area, barrel field | 6.90 ± 2.48 | 14 | 6.63 ± 2.17 | 15 | 19.37 ± 3.84 | 10 | 34.91 ± 5.68 | 9 | 35.8 | 3.54E-07 | 2.42 | 0.127 | 5.47 | 0.0239 |  |  | **** | * | **** | * |
| 337 | 322 | Primary somatosensory area, lower limb | 6.74 ± 3.03 | 13 | 3.01 ± 1.18 | 10 | 30.68 ± 5.73 | 10 | 58.81 ± 15.27 | 9 | 27.4 | 0.0000063 | 2.81 | 0.102 | 4.67 | 0.037 |  |  | **** |  | **** |  |
| 345 | 322 | Primary somatosensory area, mouth | 9.63 ± 3.91 | 13 | 6.09 ± 1.03 | 12 | 15.55 ± 4.92 | 10 | 18.33 ± 4.13 | 8 | 5.62 | 0.0228 | 0.109 | 0.743 | 0.713 | 0.404 |  |  |  |  |  |  |
| 369 | 322 | Primary somatosensory area, upper limb | 9.66 ± 3.36 | 14 | 6.89 ± 2.87 | 13 | 20.72 ± 4.37 | 10 | 34.75 ± 8.80 | 9 | 16 | 0.00025 | 0.731 | 0.397 | 3.1 | 0.0856 |  |  | ** |  | ** |  |
| 361 | 322 | Primary somatosensory area, trunk | 6.44 ± 1.56 | 13 | 6.60 ± 2.40 | 13 | 35.13 ± 5.87 | 10 | 46.55 ± 11.90 | 9 | 36 | 4.32E-07 | 0.514 | 0.478 | 0.978 | 0.329 |  | ** | **** | ** | **** |  |
| 182305689 | 322 | Primary somatosensory area, unassigned | 7.64 ± 4.28 | 14 | 3.45 ± 1.32 | 11 | 14.41 ± 2.64 | 10 | 41.83 ± 12.39 | 9 | 12.9 | 0.000905 | 3.02 | 0.0897 | 7.05 | 0.0113 |  |  | ** |  | *** | * |
| 378 | 453 | Supplemental somatosensory area | 8.59 ± 2.35 | 14 | 12.11 ± 4.05 | 15 | 20.82 ± 3.37 | 10 | 50.86 ± 15.16 | 9 | 14.6 | 0.000416 | 4.02 | 0.0511 | 4.03 | 0.051 |  |  | *** |  | *** | * |
| 1057 | 315 | Gustatory areas | 6.25 ± 2.92 | 15 | 8.05 ± 3.73 | 13 | 14.53 ± 4.51 | 10 | 35.97 ± 14.13 | 10 | 6.84 | 0.0122 | 2.51 | 0.121 | 2.08 | 0.157 |  |  | * |  | * |  |
| 677 | 315 | Visceral area | 9.44 ± 2.88 | 14 | 10.52 ± 5.44 | 13 | 25.27 ± 3.71 | 10 | 35.68 ± 6.46 | 9 | 18.6 | 0.0000969 | 1.06 | 0.31 | 0.981 | 0.328 |  |  | ** |  | ** |  |
| 247 | 315 | Auditory areas | 10.83 ± 2.92 | 14 | 6.04 ± 1.94 | 14 | 11.06 ± 2.13 | 10 | 54.02 ± 32.01 | 10 | 3.14 | 0.0831 | 1.27 | 0.266 | 3.08 | 0.086 |  |  |  |  |  |  |
| 1011 | 247 | Dorsal auditory area | 17.69 ± 8.89 | 12 | 8.52 ± 3.05 | 14 | 21.26 ± 3.62 | 10 | 83.71 ± 49.15 | 10 | 3.38 | 0.0729 | 0.881 | 0.353 | 2.63 | 0.112 |  |  |  |  |  |  |
| 1002 | 247 | Primary auditory area | 16.81 ± 4.93 | 13 | 8.79 ± 3.94 | 13 | 16.43 ± 4.11 | 10 | 74.83 ± 52.16 | 9 | 2.24 | 0.143 | 0.831 | 0.367 | 2.43 | 0.126 |  |  |  |  |  |  |
| 1027 | 247 | Posterior auditory area | 20.38 ± 6.63 | 12 | 3.38 ± 2.15 | 7 | 19.22 ± 10.50 | 8 | 33.38 ± 14.04 | 6 | 2.09 | 0.161 | 0.0759 | 0.785 | 2.86 | 0.104 |  |  |  |  |  |  |
| 1018 | 247 | Ventral auditory area | 15.58 ± 5.15 | 14 | 10.66 ± 3.62 | 14 | 14.93 ± 3.20 | 10 | 89.32 ± 55.95 | 10 | 2.69 | 0.108 | 1.44 | 0.236 | 2.78 | 0.102 |  |  |  |  |  |  |
| 669 | 315 | Visual areas | 8.42 ± 2.63 | 14 | 3.54 ± 1.08 | 14 | 13.11 ± 3.21 | 10 | 37.91 ± 18.18 | 8 | 7.53 | 0.00889 | 0.74 | 0.394 | 4.81 | 0.0339 |  |  | * |  | ** |  |
| 402 | 669 | Anterolateral visual area | 15.34 ± 6.99 | 8 | 4.77 ± 1.90 | 7 | 16.94 ± 6.70 | 7 | 117.60 ± 92.92 | 7 | 1.51 | 0.23 | 1 | 0.326 | 1.52 | 0.229 |  |  |  |  |  |  |
| 394 | 669 | Anteromedial visual area | 11.54 ± 5.55 | 13 | 8.21 ± 4.02 | 13 | 28.16 ± 5.57 | 10 | 53.97 ± 14.75 | 8 | 17.4 | 0.000159 | 0.932 | 0.34 | 4.02 | 0.0517 |  |  | ** |  | *** |  |
| 385 | 669 | Primary visual area | 14.06 ± 3.55 | 11 | 3.49 ± 1.28 | 10 | 13.67 ± 5.21 | 8 | 52.83 ± 27.42 | 8 | 3.81 | 0.0595 | 0.897 | 0.35 | 4.1 | 0.0511 |  |  |  |  | * |  |
| 533 | 669 | posteromedial visual area | 26.40 ± 7.87 | 10 | 13.53 ± 7.23 | 9 | 18.53 ± 5.23 | 9 | 43.13 ± 10.06 | 7 | 1.44 | 0.239 | 0.251 | 0.62 | 6.01 | 0.02 |  |  |  |  |  |  |
| 31 | 315 | Anterior cingulate area | 3.94 ± 0.80 | 15 | 11.65 ± 5.33 | 14 | 21.63 ± 5.61 | 10 | 34.37 ± 11.04 | 10 | 11.3 | 0.00157 | 2.94 | 0.0931 | 0.176 | 0.676 |  |  | ** |  |  |  |
| 39 | 31 | Anterior cingulate area, dorsal part | 4.57 ± 1.05 | 15 | 20.00 ± 10.28 | 14 | 33.95 ± 8.46 | 10 | 41.62 ± 8.83 | 10 | 10.6 | 0.00219 | 2.69 | 0.108 | 0.243 | 0.624 |  |  | ** |  |  |  |
| 48 | 31 | Anterior cingulate area, ventral part | 7.54 ± 2.32 | 15 | 14.40 ± 5.09 | 13 | 32.99 ± 9.06 | 10 | 66.33 ± 26.42 | 10 | 9.98 | 0.00286 | 2.59 | 0.114 | 1.19 | 0.281 |  |  | ** |  | * |  |
| 972 | 315 | Prelimbic area | 6.42 ± 3.36 | 9 | 28.49 ± 20.82 | 7 | 13.35 ± 6.68 | 4 | 12.59 ± 4.28 | 6 | 0.189 | 0.668 | 1.13 | 0.299 | 0.851 | 0.366 |  |  |  |  |  |  |
| 95 | 315 | Agranular insular area | 5.38 ± 1.72 | 15 | 5.03 ± 0.89 | 15 | 14.86 ± 3.54 | 10 | 20.88 ± 4.34 | 10 | 24.5 | 0.0000104 | 0.768 | 0.386 | 1.55 | 0.22 |  |  | *** | * | *** |  |
| 104 | 95 | Agranular insular area, dorsal part | 6.25 ± 1.72 | 14 | 7.36 ± 2.40 | 14 | 24.94 ± 7.85 | 10 | 38.77 ± 12.65 | 9 | 16.7 | 0.000185 | 0.731 | 0.397 | 1.08 | 0.304 |  |  | ** |  | ** |  |
| 111 | 95 | Agranular insular area, posterior part | 11.55 ± 4.78 | 15 | 8.13 ± 2.55 | 13 | 24.42 ± 5.26 | 10 | 32.43 ± 6.24 | 10 | 14.7 | 0.000394 | 0.178 | 0.675 | 1.42 | 0.24 |  |  | * |  | ** |  |
| 119 | 95 | Agranular insular area, ventral part | 6.59 ± 1.79 | 14 | 5.92 ± 1.40 | 15 | 47.52 ± 24.82 | 10 | 40.86 ± 14.79 | 9 | 10.1 | 0.00273 | 0.156 | 0.695 | 0.0629 | 0.803 |  |  |  |  |  |  |
| 254 | 315 | Retrosplenial area | 10.49 ± 4.30 | 14 | 11.16 ± 4.02 | 15 | 30.99 ± 6.29 | 10 | 42.24 ± 6.72 | 10 | 24.7 | 0.0000101 | 0.795 | 0.377 | 1.03 | 0.315 |  | * | *** | * | *** |  |
| 894 | 254 | Retrosplenial area, lateral agranular part | 11.77 ± 3.81 | 13 | 6.90 ± 2.30 | 15 | 41.80 ± 8.89 | 10 | 56.81 ± 6.75 | 10 | 56.9 | 1.82E-09 | 0.15 | 0.701 | 3.47 | 0.0692 |  | ** | **** | *** | **** |  |
| 879 | 254 | Retrosplenial area, dorsal part | 11.05 ± 2.70 | 14 | 9.64 ± 3.10 | 15 | 33.57 ± 5.62 | 10 | 61.98 ± 12.01 | 9 | 42 | 6.63E-08 | 2.48 | 0.122 | 6.76 | 0.0127 |  | * | **** | * | **** | * |
| 886 | 254 | Retrosplenial area, ventral part | 22.31 ± 11.30 | 14 | 27.87 ± 11.52 | 15 | 61.71 ± 14.92 | 10 | 76.84 ± 14.47 | 10 | 11.6 | 0.0014 | 0.467 | 0.498 | 0.136 | 0.714 |  |  | * |  | * |  |
| 22 | 315 | Posterior parietal association areas | 5.44 ± 1.80 | 14 | 3.93 ± 1.48 | 15 | 14.75 ± 2.94 | 10 | 45.64 ± 27.99 | 10 | 4.88 | 0.0323 | 0.972 | 0.33 | 1.93 | 0.171 |  |  |  |  |  |  |
| 312782546 | 22 | Anterior area | 6.81 ± 2.56 | 15 | 5.89 ± 1.98 | 15 | 23.24 ± 4.54 | 10 | 28.46 ± 6.20 | 9 | 28.9 | 2.64E-06 | 0.0762 | 0.784 | 0.723 | 0.4 |  | * | *** | ** | *** |  |
| 417 | 22 | Rostrolateral visual area | 7.56 ± 2.93 | 14 | 6.39 ± 2.96 | 14 | 20.30 ± 5.14 | 10 | 81.81 ± 52.96 | 8 | 4.97 | 0.0313 | 1.3 | 0.26 | 2.76 | 0.104 |  |  | * |  | * |  |
| 541 | 315 | Temporal association areas | 12.21 ± 5.50 | 13 | 7.10 ± 2.46 | 15 | 19.34 ± 2.76 | 10 | 53.15 ± 21.86 | 10 | 7.62 | 0.00839 | 1.1 | 0.299 | 3.91 | 0.0544 |  |  | * |  | ** |  |
| 922 | 315 | Perirhinal area | 11.83 ± 4.85 | 14 | 4.56 ± 1.70 | 15 | 22.29 ± 6.38 | 10 | 19.37 ± 4.45 | 10 | 8.27 | 0.00614 | 1.73 | 0.195 | 0.243 | 0.625 |  |  |  | * |  |  |
| 895 | 315 | Ectorhinal area | 14.63 ± 8.02 | 14 | 3.28 ± 1.03 | 15 | 18.47 ± 5.31 | 10 | 30.13 ± 6.17 | 10 | 7.15 | 0.0104 | 0.15 | 0.701 | 3.93 | 0.0535 |  |  |  |  | ** |  |
| 698 | 695 | Olfactory areas | 13.76 ± 4.55 | 14 | 6.69 ± 0.99 | 14 | 15.84 ± 2.56 | 12 | 31.83 ± 5.50 | 10 | 12.7 | 0.000859 | 0.454 | 0.504 | 9.95 | 0.00284 |  |  | ** |  | *** | * |
| 159 | 698 | Anterior olfactory nucleus | 18.65 ± 8.79 | 8 | 8.35 ± 2.50 | 7 | 89.98 ± 22.41 | 6 | 138.62 ± 27.32 | 6 | 36.4 | 3.78E-06 | 1.37 | 0.254 | 3.16 | 0.0887 |  | * | *** | * | **** |  |
| 589 | 698 | Taenia tecta | 5.38 ± 1.42 | 12 | 5.09 ± 1.71 | 9 | 27.37 ± 7.68 | 8 | 64.79 ± 30.95 | 8 | 8.17 | 0.00734 | 1.91 | 0.177 | 1.84 | 0.184 |  |  | * |  | * |  |
| 597 | 589 | Taenia tecta, dorsal part | 10.92 ± 2.91 | 12 | 11.59 ± 3.97 | 8 | 59.20 ± 16.77 | 8 | 129.11 ± 62.04 | 8 | 7.68 | 0.00923 | 1.96 | 0.171 | 1.43 | 0.24 |  |  | * |  | * |  |
| 814 | 698 | Dorsal peduncular area | 12.17 ± 2.80 | 13 | 29.03 ± 10.53 | 8 | 34.47 ± 13.42 | 6 | 103.29 ± 50.58 | 8 | 3.7 | 0.0637 | 3.8 | 0.0603 | 1.09 | 0.305 |  |  | * |  |  |  |
| 961 | 698 | Piriform area | 41.73 ± 11.14 | 12 | 36.06 ± 13.48 | 10 | 41.49 ± 7.31 | 8 | 66.44 ± 14.62 | 10 | 1.52 | 0.226 | 0.605 | 0.442 | 1.5 | 0.228 |  |  |  |  |  |  |
| 619 | 698 | Nucleus of the lateral olfactory tract | 7.21 ± 3.85 | 10 | 6.46 ± 2.37 | 7 | 40.43 ± 9.33 | 9 | 92.54 ± 39.01 | 4 | 19.4 | 0.000163 | 1.52 | 0.228 | 4.61 | 0.0413 |  |  | *** |  | ** |  |
| 260 | 619 | Nucleus of the lateral olfactory tract, molecular layer | 15.30 ± 9.63 | 10 | 34.25 ± 11.87 | 6 | 32.44 ± 9.47 | 9 | 136.40 ± 63.07 | 4 | 5.59 | 0.0261 | 6.56 | 0.0169 | 4.31 | 0.0483 |  |  | ** |  | * | * |
| 268 | 619 | Nucleus of the lateral olfactory tract, pyramidal layer | 9.87 ± 7.72 | 10 | 3.77 ± 1.90 | 7 | 128.48 ± 28.05 | 9 | 237.25 ± 123.61 | 4 | 19.4 | 0.00016 | 0.377 | 0.544 | 2.31 | 0.14 |  |  | ** |  | ** |  |
| 1139 | 619 | Nucleus of the lateral olfactory tract, layer 3 | 15.98 ± 9.65 | 10 | 7.03 ± 7.03 | 7 | 45.53 ± 20.81 | 9 | 175.03 ± 120.11 | 4 | 5.36 | 0.0288 | 1.23 | 0.278 | 3.84 | 0.0608 |  |  | * |  | * |  |
| 631 | 698 | Cortical amygdalar area | 9.08 ± 4.90 | 14 | 6.53 ± 1.94 | 12 | 9.45 ± 1.94 | 9 | 22.45 ± 5.46 | 10 | 3.78 | 0.0587 | 1.21 | 0.277 | 3.46 | 0.07 |  |  |  |  | * |  |
| 639 | 631 | Cortical amygdalar area, anterior part | 29.24 ± 16.45 | 14 | 12.46 ± 2.80 | 11 | 45.04 ± 9.23 | 9 | 86.95 ± 23.30 | 10 | 8.07 | 0.00704 | 0.654 | 0.423 | 3.51 | 0.0683 |  |  | * |  | ** |  |
| 647 | 631 | Cortical amygdalar area, posterior part | 12.20 ± 6.80 | 13 | 8.41 ± 3.13 | 12 | 5.50 ± 1.52 | 8 | 26.65 ± 7.97 | 9 | 1.05 | 0.313 | 1.31 | 0.26 | 4.51 | 0.0402 |  |  |  |  |  |  |
| 655 | 647 | Cortical amygdalar area, posterior part, lateral zone | 22.05 ± 11.43 | 13 | 15.43 ± 7.18 | 12 | 13.23 ± 3.21 | 8 | 62.89 ± 19.48 | 9 | 2.74 | 0.106 | 2.11 | 0.154 | 5.51 | 0.0242 |  |  |  |  | * |  |
| 663 | 647 | Cortical amygdalar area, posterior part, medial zone | 28.58 ± 19.10 | 11 | 17.71 ± 9.68 | 12 | 8.65 ± 3.66 | 8 | 38.29 ± 15.09 | 9 | 0.00974 | 0.922 | 0.207 | 0.652 | 2.07 | 0.159 |  |  |  |  |  |  |
| 788 | 698 | Piriform-amygdalar area | 24.69 ± 10.40 | 15 | 16.54 ± 6.42 | 14 | 33.19 ± 7.27 | 10 | 59.63 ± 18.51 | 10 | 5.28 | 0.0262 | 0.343 | 0.561 | 2.42 | 0.127 |  |  |  |  | * |  |
| 1089 | 695 | Hippocampal formation | 13.31 ± 3.98 | 15 | 16.43 ± 3.07 | 15 | 57.00 ± 13.93 | 12 | 60.94 ± 10.31 | 10 | 29.2 | 2.03E-06 | 0.0351 | 0.852 | 0.00251 | 0.96 |  | ** | ** | ** | ** |  |
| 1080 | 1089 | Hippocampal region | 17.95 ± 5.52 | 15 | 24.50 ± 4.58 | 15 | 76.30 ± 19.02 | 12 | 79.64 ± 14.85 | 10 | 25 | 7.95E-06 | 0.0567 | 0.813 | 0.0199 | 0.888 |  | ** | ** | ** | ** |  |
| 375 | 1080 | Ammon's horn | 22.27 ± 6.57 | 15 | 35.81 ± 7.65 | 15 | 97.57 ± 21.92 | 12 | 106.24 ± 19.19 | 10 | 27.5 | 3.53E-06 | 0.359 | 0.552 | 0.0304 | 0.862 |  | ** | *** | * | ** |  |
| 382 | 375 | Field CA1 | 39.81 ± 13.45 | 15 | 74.95 ± 18.36 | 15 | 236.04 ± 46.87 | 12 | 232.86 ± 38.55 | 10 | 36.2 | 2.36E-07 | 0.144 | 0.706 | 0.418 | 0.521 |  | **** | *** | ** | ** |  |
| 423 | 375 | Field CA2 | 42.46 ± 14.34 | 15 | 47.93 ± 13.61 | 15 | 148.84 ± 44.92 | 12 | 189.78 ± 56.18 | 9 | 15.1 | 0.00032 | 0.134 | 0.716 | 0.313 | 0.578 |  |  | * |  | * |  |
| 463 | 375 | Field CA3 | 49.49 ± 15.67 | 15 | 70.68 ± 14.52 | 15 | 158.04 ± 42.60 | 12 | 206.46 ± 41.21 | 9 | 18.5 | 0.0000857 | 0.743 | 0.393 | 0.232 | 0.632 |  | * | ** |  | * |  |
| 726 | 1080 | Dentate gyrus | 38.56 ± 13.38 | 15 | 39.91 ± 9.02 | 15 | 172.39 ± 57.24 | 11 | 156.31 ± 32.65 | 10 | 17.8 | 0.000109 | 0.089 | 0.767 | 0.0861 | 0.77 |  | * | * | * | * |  |
| 10703 | 726 | Dentate gyrus, molecular layer | 77.49 ± 25.82 | 15 | 66.72 ± 15.46 | 15 | 313.35 ± 96.93 | 11 | 271.15 ± 53.22 | 10 | 19.1 | 0.0000682 | 0.336 | 0.565 | 0.0968 | 0.757 |  | ** | * | ** | * |  |
| 10704 | 726 | Dentate gyrus, polymorph layer | 98.11 ± 46.41 | 15 | 142.45 ± 34.99 | 15 | 332.78 ± 133.74 | 11 | 465.18 ± 110.73 | 10 | 11.8 | 0.00124 | 0.871 | 0.355 | 0.297 | 0.588 |  |  | * |  | * |  |
| 632 | 726 | Dentate gyrus, granule cell layer | 69.34 ± 25.97 | 15 | 91.38 ± 25.63 | 15 | 426.23 ± 160.88 | 11 | 374.11 ± 90.58 | 10 | 15.5 | 0.000276 | 0.039 | 0.844 | 0.206 | 0.652 |  | * |  | * |  |  |
| 982 | 1080 | Fasciola cinerea | 201.26 ± 60.95 | 13 | 235.22 ± 60.88 | 13 | 665.18 ± 283.96 | 11 | 658.64 ± 241.10 | 9 | 6.98 | 0.0114 | 0.0145 | 0.905 | 0.0145 | 0.905 |  |  |  |  |  |  |
| 19 | 1080 | Induseum griseum | 40.36 ± 16.37 | 15 | 88.73 ± 38.72 | 12 | 54.06 ± 14.72 | 12 | 142.48 ± 37.23 | 10 | 1.35 | 0.252 | 5.96 | 0.0186 | 0.531 | 0.47 |  |  |  |  |  |  |
| 822 | 1089 | Retrohippocampal region | 10.48 ± 3.52 | 14 | 8.80 ± 2.88 | 15 | 96.91 ± 53.50 | 12 | 68.76 ± 14.20 | 9 | 7.7 | 0.00794 | 0.527 | 0.471 | 0.245 | 0.623 |  |  |  |  |  |  |
| 909 | 822 | Entorhinal area | 7.12 ± 3.36 | 14 | 5.27 ± 2.28 | 15 | 10.23 ± 3.05 | 10 | 16.21 ± 4.33 | 9 | 4.57 | 0.0382 | 0.0907 | 0.765 | 1.45 | 0.235 |  |  |  |  |  |  |
| 918 | 909 | Entorhinal area, lateral part | 11.03 ± 5.20 | 14 | 7.66 ± 3.52 | 15 | 15.78 ± 4.71 | 10 | 25.05 ± 6.70 | 9 | 4.74 | 0.0348 | 0.0572 | 0.812 | 1.58 | 0.216 |  |  |  |  |  |  |
| 1037 | 822 | Postsubiculum | 96.62 ± 59.14 | 5 | 96.21 ± 38.00 | 6 | 45.48 ± 45.48 | 4 | 176.16 ± 55.22 | 5 | 0.177 | 0.68 | 1.37 | 0.259 | 1.69 | 0.212 |  |  |  |  |  |  |
| 502 | 822 | Subiculum | 105.07 ± 47.75 | 12 | 49.79 ± 29.39 | 11 | 546.74 ± 158.41 | 10 | 651.54 ± 87.13 | 7 | 30.9 | 2.71E-06 | 0.0582 | 0.811 | 0.744 | 0.394 |  | ** | ** | ** | *** |  |
| 484682470 | 822 | Prosubiculum | 25.39 ± 11.06 | 10 | 86.19 ± 58.03 | 10 | 527.25 ± 149.10 | 10 | 669.50 ± 104.13 | 7 | 31.5 | 3.01E-06 | 0.274 | 0.604 | 0.178 | 0.675 |  | ** | *** | ** | ** |  |

| | | | | | | | | | | | | | | | | | | | | | |
|---|---|---|---|---|---|---|---|---|---|---|---|---|---|---|---|---|---|---|---|---|---|
| 703 | 688 | Cortical subplate | 9.13 ± 6.04 | 15 | 5.66 ± 1.49 | 15 | 23.45 ± 3.00 | 12 | 40.15 ± 6.94 | 10 | 25.4 | 6.98E-06 | 0.717 | 0.401 | 4.52 | 0.0386 | | *** | * | **** | |
| 583 | 703 | Claustrum | 7.28 ± 3.41 | 15 | 24.59 ± 11.25 | 14 | 71.94 ± 18.02 | 12 | 74.03 ± 21.49 | 10 | 17.7 | 0.000115 | 0.457 | 0.502 | 0.308 | 0.581 | ** | ** | | | |
| 942 | 703 | Endopiriform nucleus | 9.26 ± 5.30 | 14 | 12.28 ± 5.18 | 15 | 23.68 ± 4.72 | 12 | 36.81 ± 6.88 | 10 | 12.1 | 0.00108 | 1.28 | 0.265 | 0.832 | 0.366 | | ** | | * | |
| 952 | 942 | Endopiriform nucleus, dorsal part | 16.66 ± 8.93 | 14 | 21.67 ± 5.76 | 15 | 38.13 ± 6.83 | 12 | 60.91 ± 13.99 | 10 | 11.7 | 0.00132 | 1.54 | 0.22 | 1.02 | 0.318 | | ** | | * | |
| 966 | 942 | Endopiriform nucleus, ventral part | 21.66 ± 13.40 | 14 | 28.32 ± 20.09 | 15 | 63.03 ± 14.33 | 12 | 95.95 ± 16.36 | 10 | 10.1 | 0.00258 | 0.759 | 0.388 | 0.598 | 0.443 | | * | | * | |
| 131 | 703 | Lateral amygdalar nucleus | 17.70 ± 9.49 | 14 | 15.93 ± 5.01 | 15 | 46.27 ± 14.90 | 12 | 54.01 ± 12.70 | 10 | 9.83 | 0.00296 | 0.000696 | 0.979 | 0.202 | 0.655 | | | | | |
| 295 | 703 | Basolateral amygdalar nucleus | 14.02 ± 10.08 | 14 | 5.39 ± 1.44 | 13 | 67.14 ± 5.24 | 10 | 90.75 ± 12.86 | 10 | 64.9 | 3.91E-10 | 0.563 | 0.457 | 3.55 | 0.0664 | *** | **** | **** | **** | |
| 303 | 295 | Basolateral amygdalar nucleus, anterior part | 27.70 ± 17.97 | 14 | 15.37 ± 4.75 | 13 | 161.03 ± 13.94 | 10 | 230.26 ± 35.55 | 10 | 76.8 | 4.05E-11 | 1.7 | 0.199 | 4.24 | 0.0455 | *** | **** | **** | **** | |
| 311 | 295 | Basolateral amygdalar nucleus, posterior part | 17.26 ± 9.77 | 13 | 8.51 ± 3.80 | 13 | 103.56 ± 22.26 | 9 | 161.51 ± 32.43 | 9 | 47.4 | 2.73E-08 | 1.18 | 0.285 | 3.68 | 0.0622 | ** | **** | ** | **** | |
| 451 | 295 | Basolateral amygdalar nucleus, ventral part | 51.17 ± 47.51 | 12 | 4.39 ± 2.48 | 12 | 74.52 ± 19.59 | 8 | 114.95 ± 23.46 | 9 | 4.79 | 0.035 | 0.0804 | 0.778 | 1.94 | 0.172 | | | | | |
| 319 | 703 | Basomedial amygdalar nucleus | 17.86 ± 13.73 | 15 | 4.98 ± 1.81 | 13 | 30.35 ± 6.79 | 11 | 61.05 ± 13.40 | 10 | 9.6 | 0.00335 | 0.345 | 0.56 | 4.17 | 0.0471 | | * | | ** | |
| 327 | 319 | Basomedial amygdalar nucleus, anterior part | 23.09 ± 16.70 | 15 | 7.29 ± 2.46 | 11 | 71.46 ± 22.20 | 11 | 86.26 ± 21.42 | 10 | 12.5 | 0.00101 | 0.00583 | 0.94 | 0.747 | 0.392 | | | | * | |
| 334 | 319 | Basomedial amygdalar nucleus, posterior part | 50.46 ± 39.91 | 14 | 8.44 ± 3.82 | 13 | 56.75 ± 12.04 | 9 | 160.46 ± 34.00 | 9 | 6.99 | 0.0115 | 0.375 | 0.544 | 6.09 | 0.0178 | | | | ** | |
| 780 | 703 | Posterior amygdalar nucleus | 32.66 ± 22.10 | 12 | 17.03 ± 9.75 | 13 | 22.03 ± 10.62 | 10 | 53.02 ± 16.70 | 9 | 0.603 | 0.442 | 0.0618 | 0.805 | 2.14 | 0.152 | | | | | |
| 623 | 567 | Cerebral nuclei | 7.16 ± 2.31 | 15 | 9.86 ± 2.01 | 15 | 17.77 ± 2.51 | 12 | 23.66 ± 1.91 | 10 | 28.6 | 2.48E-06 | 2.45 | 0.124 | 0.494 | 0.486 | ** | **** | | *** | |
| 477 | 623 | Striatum | 9.90 ± 3.18 | 15 | 13.25 ± 2.64 | 15 | 25.06 ± 3.17 | 12 | 32.84 ± 2.44 | 10 | 33.2 | 5.72E-07 | 2.27 | 0.139 | 0.545 | 0.464 | ** | **** | * | *** | |
| 485 | 477 | Striatum dorsal region | 9.98 ± 1.91 | 15 | 20.59 ± 6.41 | 15 | 18.39 ± 3.30 | 12 | 23.70 ± 5.47 | 10 | 1.56 | 0.217 | 3.04 | 0.0878 | 0.315 | 0.577 | | | | | |
| 672 | 485 | Caudoputamen | 23.02 ± 3.71 | 13 | 41.18 ± 12.82 | 15 | 36.77 ± 6.59 | 12 | 52.66 ± 10.72 | 9 | 1.69 | 0.2 | 2.74 | 0.105 | 0.0135 | 0.908 | | | | | |
| 493 | 477 | Striatum ventral region | 17.36 ± 4.85 | 15 | 18.10 ± 3.50 | 14 | 60.48 ± 6.20 | 12 | 67.55 ± 13.23 | 10 | 44.5 | 2.57E-08 | 0.101 | 0.752 | 0.209 | 0.65 | *** | **** | *** | **** | |
| 56 | 493 | Nucleus accumbens | 21.39 ± 3.87 | 13 | 17.66 ± 2.92 | 13 | 147.04 ± 18.04 | 12 | 159.72 ± 16.88 | 8 | 133 | 1.4E-14 | 0.754 | 0.39 | 0.499 | 0.484 | **** | **** | **** | **** | |
| 998 | 493 | Fundus of striatum | 7.26 ± 4.76 | 10 | 18.65 ± 10.39 | 4 | 76.94 ± 40.21 | 7 | 131.20 ± 68.92 | 4 | 7.02 | 0.015 | 1.35 | 0.258 | 0.405 | 0.531 | | | | | |
| 754 | 493 | Olfactory tubercle | 73.85 ± 23.46 | 13 | 68.94 ± 12.81 | 12 | 87.05 ± 10.73 | 12 | 78.94 ± 6.82 | 8 | 0.503 | 0.482 | 0.191 | 0.665 | 0.00904 | 0.925 | | | | | |
| 275 | 477 | Lateral septal complex | 6.44 ± 1.83 | 14 | 11.89 ± 2.78 | 12 | 124.10 ± 14.85 | 11 | 171.78 ± 17.69 | 10 | 175 | 9.72E-17 | 6.46 | 0.0147 | 4.1 | 0.049 | **** | **** | **** | **** | * |
| 242 | 275 | Lateral septal nucleus | 7.92 ± 2.28 | 14 | 14.16 ± 3.65 | 12 | 177.79 ± 19.45 | 11 | 259.15 ± 28.15 | 10 | 182 | 4.84E-17 | 7.99 | 0.0071 | 6.07 | 0.0178 | **** | **** | **** | **** | ** |
| 258 | 242 | Lateral septal nucleus, rostral (rostroventral) part | 16.28 ± 4.63 | 14 | 31.78 ± 7.94 | 11 | 388.50 ± 46.88 | 11 | 540.04 ± 55.75 | 10 | 170 | 2.35E-16 | 7.91 | 0.00743 | 4.13 | 0.0484 | **** | **** | **** | **** | * |
| 266 | 242 | Lateral septal nucleus, ventral part | 7.76 ± 6.51 | 10 | 12.13 ± 5.67 | 8 | 285.96 ± 35.68 | 11 | 438.18 ± 100.78 | 8 | 50 | 4.31E-08 | 2.15 | 0.152 | 2.29 | 0.14 | *** | **** | ** | **** | |
| 333 | 275 | Septohippocampal nucleus | 22.66 ± 11.46 | 13 | 132.15 ± 49.38 | 7 | 339.67 ± 116.13 | 9 | 357.09 ± 141.35 | 6 | 12.5 | 0.00132 | 1.05 | 0.314 | 0.312 | 0.581 | * | * | | | |
| 278 | 477 | Striatum-like amygdalar nuclei | 21.37 ± 12.64 | 15 | 8.97 ± 2.41 | 14 | 16.01 ± 3.23 | 12 | 29.88 ± 7.32 | 10 | 0.685 | 0.412 | 0.0249 | 0.875 | 2.47 | 0.122 | | | | | |
| 23 | 278 | Anterior amygdalar area | 12.19 ± 5.99 | 10 | 2.78 ± 1.74 | 5 | 72.61 ± 35.08 | 8 | 152.71 ± 95.78 | 4 | 6.85 | 0.0154 | 0.698 | 0.412 | 1.5 | 0.233 | | | | | |
| 292 | 278 | Bed nucleus of the accessory olfactory tract | 18.87 ± 12.80 | 12 | 18.05 ± 12.24 | 8 | 12.70 ± 12.70 | 6 | 16.30 ± 10.96 | 4 | 0.0925 | 0.763 | 0.00224 | 0.963 | 0.0223 | 0.882 | | | | | |
| 536 | 278 | Central amygdalar nucleus | 25.99 ± 17.15 | 14 | 8.86 ± 3.22 | 13 | 23.94 ± 6.53 | 12 | 39.82 ± 10.38 | 10 | 1.38 | 0.247 | 0.0578 | 0.811 | 2.1 | 0.154 | | | | | |
| 544 | 536 | Central amygdalar nucleus, capsular part | 29.55 ± 9.83 | 13 | 26.91 ± 8.25 | 13 | 78.56 ± 9.38 | 12 | 121.58 ± 29.46 | 10 | 22.7 | 0.0000211 | 1.03 | 0.316 | 2.36 | 0.132 | | *** | | *** | |
| 551 | 536 | Central amygdalar nucleus, lateral part | 49.60 ± 29.61 | 13 | 15.11 ± 7.72 | 13 | 37.64 ± 12.09 | 12 | 56.78 ± 22.88 | 10 | 0.45 | 0.506 | 0.28 | 0.599 | 1.77 | 0.191 | | | | | |
| 559 | 536 | Central amygdalar nucleus, medial part | 61.36 ± 45.46 | 14 | 14.59 ± 6.72 | 13 | 38.71 ± 18.03 | 12 | 74.22 ± 21.46 | 10 | 0.289 | 0.594 | 0.127 | 0.723 | 1.97 | 0.168 | | | | | |
| 1105 | 278 | Intercalated amygdalar nucleus | 9.47 ± 4.97 | 15 | 15.42 ± 7.54 | 13 | 48.35 ± 18.71 | 12 | 145.47 ± 51.42 | 10 | 12.4 | 0.000991 | 3.92 | 0.0538 | 3.92 | 0.0537 | | *** | | ** | * |
| 403 | 278 | Medial amygdalar nucleus | 54.00 ± 35.75 | 15 | 23.55 ± 6.66 | 14 | 26.18 ± 4.56 | 12 | 45.30 ± 8.65 | 10 | 0.0452 | 0.832 | 0.17 | 0.682 | 1.26 | 0.268 | | | | | |
| 803 | 623 | Pallidum | 7.46 ± 2.51 | 15 | 12.27 ± 4.28 | 14 | 16.37 ± 4.73 | 12 | 25.01 ± 4.72 | 10 | 6.99 | 0.0111 | 2.36 | 0.132 | 0.223 | 0.639 | | * | | | |
| 818 | 803 | Pallidum, dorsal region | 5.72 ± 3.18 | 15 | 8.29 ± 6.27 | 13 | 3.81 ± 0.92 | 12 | 9.21 ± 1.79 | 10 | 0.0243 | 0.877 | 0.962 | 0.332 | 0.13 | 0.72 | | | | | |
| 1022 | 818 | Globus pallidus, external segment | 16.80 ± 11.74 | 14 | 19.60 ± 16.92 | 13 | 6.70 ± 1.68 | 12 | 18.06 ± 4.68 | 10 | 0.282 | 0.598 | 0.352 | 0.556 | 0.138 | 0.712 | | | | | |
| 1031 | 818 | Globus pallidus, internal segment | 10.01 ± 2.81 | 13 | 11.39 ± 5.68 | 13 | 7.65 ± 2.56 | 12 | 16.85 ± 6.38 | 9 | 0.0761 | 0.784 | 1.12 | 0.295 | 0.752 | 0.391 | | | | | |
| 835 | 803 | Pallidum, ventral region | 11.95 ± 3.31 | 15 | 8.17 ± 1.88 | 14 | 18.32 ± 4.56 | 12 | 29.25 ± 5.98 | 10 | 11.6 | 0.00139 | 0.316 | 0.577 | 3.56 | 0.0654 | | * | | ** | |
| 342 | 835 | Substantia innominata | 24.62 ± 6.84 | 15 | 16.99 ± 3.89 | 13 | 36.13 ± 9.26 | 12 | 60.74 ± 8.49 | 9 | 12.5 | 0.00095 | 0.504 | 0.481 | 4.82 | 0.0333 | | ** | | ** | |
| 826 | 803 | Pallidum, medial region | 23.24 ± 7.98 | 14 | 50.37 ± 15.84 | 10 | 75.50 ± 24.77 | 12 | 97.42 ± 24.45 | 9 | 7.27 | 0.0101 | 1.82 | 0.185 | 0.0192 | 0.89 | | * | | | |
| 904 | 826 | Medial septal complex | 34.86 ± 11.97 | 14 | 75.92 ± 23.68 | 10 | 107.54 ± 35.01 | 12 | 142.33 ± 35.54 | 9 | 6.84 | 0.0124 | 2.08 | 0.157 | 0.0134 | 0.909 | | * | | | |
| 564 | 904 | Medial septal nucleus | 51.87 ± 20.39 | 8 | 90.42 ± 37.37 | 5 | 276.63 ± 102.96 | 8 | 401.22 ± 113.78 | 7 | 9.52 | 0.00507 | 1.6 | 0.218 | 0.252 | 0.62 | | * | | | |
| 596 | 904 | Diagonal band nucleus | 69.79 ± 24.71 | 14 | 150.60 ± 47.34 | 10 | 208.20 ± 67.34 | 12 | 250.01 ± 65.77 | 9 | 5.58 | 0.023 | 1.51 | 0.226 | 0.139 | 0.711 | | | | | |
| 343 | 8 | Brain stem | 7.13 ± 3.16 | 15 | 5.37 ± 1.24 | 15 | 10.61 ± 2.09 | 12 | 17.83 ± 2.91 | 10 | 9.68 | 0.00314 | 0.465 | 0.499 | 3.24 | 0.078 | | * | | ** | |
| 1129 | 343 | Interbrain | 8.58 ± 3.91 | 15 | 6.89 ± 1.61 | 15 | 12.53 ± 2.30 | 12 | 20.93 ± 3.59 | 10 | 8.25 | 0.00606 | 0.523 | 0.473 | 2.75 | 0.104 | | * | | * | |
| 549 | 1129 | Thalamus | 5.43 ± 1.80 | 14 | 6.69 ± 2.00 | 15 | 14.90 ± 2.66 | 12 | 25.71 ± 5.24 | 10 | 24 | 0.0000118 | 2.51 | 0.12 | 2.76 | 0.103 | | **** | | *** | |
| 864 | 549 | Thalamus, sensory-motor cortex related | 2.79 ± 0.92 | 14 | 4.57 ± 1.40 | 15 | 15.30 ± 3.16 | 12 | 26.39 ± 5.49 | 10 | 36.8 | 2.13E-07 | 2.93 | 0.0937 | 2.76 | 0.103 | * | **** | * | **** | |
| 637 | 864 | Ventral group of the dorsal thalamus | 3.22 ± 1.12 | 14 | 4.05 ± 1.49 | 15 | 18.55 ± 3.68 | 12 | 31.14 ± 6.94 | 10 | 37.9 | 1.57E-07 | 1.85 | 0.18 | 2.96 | 0.0917 | * | **** | * | **** | |
| 629 | 637 | Ventral anterior-lateral complex of the thalamus | 5.87 ± 2.40 | 14 | 13.22 ± 6.49 | 13 | 39.66 ± 9.69 | 12 | 78.12 ± 25.98 | 10 | 15.8 | 0.000257 | 2.71 | 0.106 | 1.63 | 0.209 | | *** | | ** | |
| 685 | 637 | Ventral medial nucleus of the thalamus | 9.89 ± 5.38 | 14 | 12.47 ± 5.40 | 14 | 41.80 ± 8.97 | 12 | 69.33 ± 20.04 | 10 | 18.9 | 0.0000762 | 1.33 | 0.254 | 1.53 | 0.222 | | ** | | ** | |
| 709 | 637 | Ventral posterior complex of the thalamus | 4.75 ± 1.64 | 13 | 4.26 ± 2.01 | 15 | 25.97 ± 5.19 | 12 | 43.42 ± 9.24 | 10 | 40.5 | 8.13E-08 | 1.14 | 0.291 | 3.62 | 0.0633 | * | **** | ** | **** | |
| 718 | 709 | Ventral posterolateral nucleus of the thalamus | 16.85 ± 7.04 | 13 | 10.88 ± 4.50 | 15 | 49.65 ± 10.89 | 12 | 98.11 ± 28.94 | 10 | 19.3 | 0.0000642 | 0.969 | 0.33 | 4.05 | 0.0501 | | *** | | *** | |
| 725 | 709 | Ventral posterolateral nucleus of the thalamus, parvicellular part | 4.87 ± 3.79 | 11 | 3.51 ± 1.92 | 11 | 57.63 ± 16.45 | 8 | 63.30 ± 21.19 | 7 | 25.7 | 0.0000149 | 0.000919 | 0.976 | 0.101 | 0.753 | ** | ** | ** | ** | |
| 733 | 709 | Ventral posteromedial nucleus of the thalamus | 5.90 ± 2.79 | 13 | 7.65 ± 4.50 | 15 | 52.26 ± 9.56 | 12 | 86.59 ± 19.14 | 10 | 43.3 | 3.88E-08 | 1.4 | 0.243 | 2.96 | 0.0922 | ** | **** | ** | **** | |
| 741 | 709 | Ventral posteromedial nucleus of the thalamus, parvicellular part | 2.07 ± 1.07 | 8 | 3.56 ± 2.66 | 13 | 58.91 ± 23.08 | 10 | 54.65 ± 17.29 | 7 | 14.4 | 0.000576 | 0.778 | 0.384 | 0.041 | 0.841 | * | | * | | |
| 563807435 | 637 | Posterior triangular thalamic nucleus | 4.36 ± 2.35 | 9 | 6.77 ± 2.87 | 7 | 47.28 ± 16.60 | 9 | 58.56 ± 17.80 | 7 | 14.5 | 0.00071 | 0.303 | 0.586 | 0.127 | 0.725 | | * | | * | |
| 406 | 864 | Subparafascicular nucleus | 8.44 ± 5.00 | 11 | 5.69 ± 2.85 | 12 | 24.15 ± 9.92 | 10 | 32.98 ± 7.14 | 8 | 10.8 | 0.00224 | 0.0115 | 0.915 | 0.8 | 0.377 | | | | * | |
| 414 | 406 | Subparafascicular nucleus, magnocellular part | 14.59 ± 9.07 | 6 | 18.08 ± 9.23 | 7 | 14.61 ± 5.80 | 6 | 16.05 ± 10.52 | 5 | 0.0125 | 0.912 | 0.0897 | 0.768 | 0.0133 | 0.909 | | | | | |
| 422 | 406 | Subparafascicular nucleus, parvicellular part | 19.72 ± 13.16 | 9 | 4.39 ± 2.28 | 8 | 65.84 ± 29.07 | 8 | 94.85 ± 29.39 | 7 | 10.4 | 0.00325 | 0.0622 | 0.805 | 1.13 | 0.296 | | | | * | |
| 1008 | 864 | Geniculate group, dorsal thalamus | 7.04 ± 2.94 | 14 | 19.72 ± 11.41 | 15 | 33.54 ± 7.36 | 12 | 65.35 ± 17.46 | 10 | 11.8 | 0.00127 | 3.3 | 0.0757 | 0.843 | 0.363 | | ** | | * | |
| 475 | 1008 | Medial geniculate complex | 7.45 ± 2.53 | 8 | 13.83 ± 7.30 | 8 | 29.40 ± 12.34 | 9 | 52.94 ± 13.33 | 7 | 9.22 | 0.00514 | 1.73 | 0.199 | 0.751 | 0.393 | | * | | | |
| 1072 | 475 | Medial geniculate complex, dorsal part | 12.41 ± 5.03 | 7 | 91.06 ± 54.05 | 6 | 40.99 ± 20.17 | 8 | 95.63 ± 17.73 | 7 | 0.408 | 0.529 | 5.82 | 0.0239 | 0.192 | 0.665 | | | | | |
| 1079 | 475 | Medial geniculate complex, ventral part | 30.94 ± 12.78 | 6 | 31.94 ± 19.23 | 6 | 29.31 ± 18.12 | 8 | 90.43 ± 36.02 | 6 | 1.35 | 0.258 | 1.88 | 0.184 | 1.74 | 0.201 | | | | | |
| 1088 | 475 | Medial geniculate complex, medial part | 6.25 ± 2.52 | 8 | 10.63 ± 5.64 | 8 | 68.13 ± 26.35 | 9 | 120.21 ± 33.45 | 7 | 16 | 0.000425 | 1.17 | 0.289 | 1.28 | 0.268 | | ** | | ** | |
| 170 | 1008 | Dorsal part of the lateral geniculate complex | 9.60 ± 4.67 | 14 | 31.23 ± 17.32 | 15 | 58.96 ± 10.55 | 12 | 104.54 ± 26.58 | 10 | 14.9 | 0.000338 | 3.27 | 0.0769 | 0.578 | 0.451 | | *** | | * | |
| 496345664 | 170 | Dorsal part of the lateral geniculate complex, shell | 42.96 ± 22.57 | 14 | 139.70 ± 109.01 | 14 | 113.37 ± 33.45 | 11 | 171.97 ± 43.09 | 9 | 0.561 | 0.458 | 1.29 | 0.262 | 0.0739 | 0.787 | | | | | |
| 496345668 | 170 | Dorsal part of the lateral geniculate complex, core | 12.38 ± 5.65 | 14 | 62.29 ± 31.82 | 15 | 116.80 ± 21.13 | 12 | 208.32 ± 49.98 | 10 | 18.1 | 0.0001 | 4.31 | 0.0433 | 0.504 | 0.481 | | *** | | ** | |
| 496345672 | 170 | Dorsal part of the lateral geniculate complex, ipsilateral zone | 17.71 ± 10.20 | 12 | 13.18 ± 7.24 | 15 | 128.28 ± 46.07 | 10 | 176.01 ± 64.80 | 9 | 16.9 | 0.00018 | 0.0366 | 0.849 | 0.613 | 0.438 | | * | | ** | |
| 856 | 549 | Thalamus, polymodal association cortex related | 9.44 ± 3.11 | 15 | 11.07 ± 3.28 | 15 | 21.20 ± 3.68 | 12 | 37.45 ± 8.22 | 10 | 17.4 | 0.000124 | 2.48 | 0.122 | 2.66 | 0.109 | | *** | | ** | |
| 138 | 856 | Lateral group of the dorsal thalamus | 11.30 ± 5.98 | 14 | 17.34 ± 7.24 | 15 | 31.05 ± 4.71 | 12 | 64.58 ± 12.36 | 10 | 18.6 | 0.0000813 | 4.38 | 0.0417 | 3.23 | 0.0786 | | *** | | *** | * |
| 218 | 138 | Lateral posterior nucleus of the thalamus | 51.91 ± 33.73 | 14 | 67.20 ± 29.40 | 15 | 61.87 ± 13.06 | 12 | 147.33 ± 29.46 | 10 | 2.29 | 0.137 | 2.24 | 0.141 | 1.47 | 0.231 | | | | | |
| 1020 | 138 | Posterior complex of the thalamus | 7.07 ± 2.46 | 14 | 8.70 ± 3.78 | 15 | 50.76 ± 7.97 | 12 | 106.25 ± 25.05 | 10 | 41.2 | 6.3E-08 | 3.64 | 0.0624 | 6.15 | 0.0168 | * | **** | * | **** | ** |
| 1029 | 138 | Posterior limiting nucleus of the thalamus | 23.63 ± 12.52 | 11 | 13.06 ± 7.59 | 7 | 86.03 ± 20.70 | 10 | 111.88 ± 30.34 | 7 | 16.6 | 0.000299 | 0.224 | 0.639 | 0.89 | 0.353 | | * | | * | |
| 325 | 138 | Suprageniculate nucleus | 5.91 ± 5.91 | 7 | 43.26 ± 12.35 | 5 | 10.54 ± 7.76 | 8 | 62.27 ± 27.29 | 7 | 0.466 | 0.501 | 8.27 | 0.00854 | 0.199 | 0.66 | | | | | |
| 560581551 | 138 | Ethmoid nucleus of the thalamus | 4.91 ± 2.08 | 13 | 5.56 ± 3.49 | 15 | 87.72 ± 22.92 | 12 | 108.25 ± 24.31 | 10 | 38.3 | 1.49E-07 | 0.0179 | 0.894 | 0.442 | 0.51 | ** | *** | ** | **** | |
| 239 | 856 | Anterior group of the dorsal thalamus | 11.54 ± 3.57 | 15 | 31.14 ± 12.66 | 13 | 40.59 ± 8.80 | 12 | 54.07 ± 11.32 | 10 | 7.89 | 0.00727 | 3.21 | 0.0799 | 0.107 | 0.746 | | * | | | |
| 255 | 239 | Anteroventral nucleus of thalamus | 10.43 ± 3.49 | 12 | 52.75 ± 47.70 | 9 | 40.02 ± 14.51 | 12 | 57.69 ± 23.59 | 8 | 0.635 | 0.431 | 1.46 | 0.234 | 0.249 | 0.621 | | | | | |
| 127 | 239 | Anteromedial nucleus | 5.54 ± 2.21 | 13 | 26.15 ± 23.18 | 9 | 12.91 ± 4.37 | 12 | 11.98 ± 4.64 | 8 | 0.0165 | 0.899 | 0.964 | 0.332 | 1.03 | 0.316 | | | | | |
| 1096 | 127 | Anteromedial nucleus, dorsal part | 14.59 ± 6.50 | 13 | 52.74 ± 48.72 | 9 | 28.19 ± 11.40 | 12 | 28.46 ± 10.75 | 8 | 0.00584 | 0.939 | 0.78 | 0.383 | 0.684 | 0.413 | | | | | |
| 1104 | 127 | Anteromedial nucleus, ventral part | 5.50 ± 2.81 | 13 | 51.87 ± 42.97 | 9 | 20.30 ± 10.20 | 12 | 21.32 ± 10.55 | 8 | 0.0186 | 0.892 | 1.59 | 0.216 | 1.2 | 0.28 | | | | | |
| 64 | 239 | Anterodorsal nucleus | 81.92 ± 36.74 | 11 | 100.42 ± 77.65 | 9 | 127.10 ± 64.34 | 10 | 110.63 ± 66.99 | 7 | 0.241 | 0.627 | 0.000554 | 0.981 | 0.0793 | 0.78 | | | | | |
| 1120 | 239 | Interanteromedial nucleus of the thalamus | 6.10 ± 3.21 | 11 | 54.43 ± 51.37 | 8 | 12.43 ± 4.82 | 11 | 39.37 ± 15.72 | 7 | 0.0101 | 0.921 | 2.64 | 0.113 | 0.207 | 0.652 | | | | | |

| | | | | | | | | | | | | | | | | | | | | |
|---|---|---|---|---|---|---|---|---|---|---|---|---|---|---|---|---|---|---|---|---|
| 1113 | 239 | Interanterodorsal nucleus of the thalamus | 5.32 ± 2.42 | 12 | 52.34 ± 49.21 | 9 | 28.93 ± 11.22 | 12 | 35.77 ± 14.05 | 7 | 0.106 | 0.747 | 1.35 | 0.253 | 0.691 | 0.411 | | | | |
| 155 | 239 | Lateral dorsal nucleus of thalamus | 24.13 ± 8.21 | 15 | 48.65 ± 18.96 | 13 | 130.27 ± 33.97 | 12 | 151.08 ± 32.49 | 10 | 19.5 | 0.0000614 | 0.861 | 0.358 | 0.00614 | 0.938 | ** | ** | | * | |
| 444 | 856 | Medial group of the dorsal thalamus | 11.57 ± 5.44 | 15 | 8.70 ± 4.65 | 14 | 26.24 ± 6.51 | 12 | 52.67 ± 12.71 | 10 | 15.6 | 0.000262 | 1.57 | 0.216 | 4.15 | 0.0473 | | ** | | *** | |
| 59 | 444 | Intermediodorsal nucleus of the thalamus | 14.24 ± 4.84 | 14 | 17.17 ± 9.43 | 14 | 40.24 ± 20.12 | 12 | 75.28 ± 44.24 | 10 | 3.85 | 0.0559 | 0.526 | 0.472 | 0.581 | 0.45 | | | | | |
| 362 | 444 | Mediodorsal nucleus of thalamus | 26.59 ± 12.87 | 15 | 20.76 ± 13.15 | 13 | 55.36 ± 12.84 | 12 | 103.54 ± 27.18 | 10 | 10.8 | 0.00192 | 1.15 | 0.289 | 2.74 | 0.105 | | ** | | ** | |
| 366 | 444 | Submedial nucleus of the thalamus | 11.08 ± 4.89 | 14 | 10.99 ± 3.13 | 13 | 51.37 ± 14.37 | 11 | 159.86 ± 34.10 | 10 | 33.2 | 7.54E-07 | 8.71 | 0.00506 | 11.5 | 0.00149 | | **** | | **** | *** |
| 1077 | 444 | Perireunensis nucleus | 33.14 ± 16.12 | 15 | 24.84 ± 9.80 | 13 | 21.60 ± 10.12 | 12 | 68.25 ± 35.99 | 10 | 0.531 | 0.47 | 0.706 | 0.405 | 2.13 | 0.151 | | | | | |
| 571 | 856 | Midline group of the dorsal thalamus | 23.42 ± 14.89 | 15 | 20.03 ± 8.12 | 15 | 16.76 ± 4.35 | 12 | 27.25 ± 7.77 | 10 | 7.25E-05 | 0.993 | 0.0552 | 0.815 | 0.428 | 0.516 | | | | | |
| 149 | 571 | Paraventricular nucleus of the thalamus | 95.98 ± 67.14 | 15 | 50.63 ± 21.28 | 15 | 28.83 ± 13.40 | 12 | 75.55 ± 27.74 | 10 | 0.297 | 0.588 | 0.0168 | 0.897 | 1.13 | 0.293 | | | | | |
| 15 | 571 | Parataenial nucleus | 2.32 ± 1.56 | 10 | 138.34 ± 129.38 | 7 | 2.84 ± 1.87 | 8 | 52.29 ± 28.73 | 5 | 0.303 | 0.586 | 2.6 | 0.119 | 0.477 | 0.496 | | | | | |
| 181 | 571 | Nucleus of reuniens | 25.61 ± 11.13 | 15 | 33.55 ± 14.45 | 13 | 34.01 ± 9.60 | 12 | 47.80 ± 11.97 | 10 | 0.833 | 0.366 | 0.739 | 0.394 | 0.0575 | 0.812 | | | | | |
| 560581559 | 571 | Xiphoid thalamic nucleus | 22.47 ± 10.35 | 15 | 48.35 ± 20.48 | 13 | 40.36 ± 25.74 | 12 | 43.45 ± 12.85 | 10 | 0.165 | 0.687 | 0.749 | 0.391 | 0.384 | 0.538 | | | | | |
| 51 | 856 | Intralaminar nuclei of the dorsal thalamus | 6.70 ± 2.76 | 15 | 6.42 ± 1.77 | 15 | 32.21 ± 5.03 | 12 | 56.75 ± 15.41 | 10 | 31.3 | 1.03E-06 | 1.67 | 0.202 | 3.46 | 0.0688 | * | **** | * | **** | |
| 189 | 51 | Rhomboid nucleus | 14.29 ± 5.63 | 15 | 15.07 ± 7.73 | 13 | 83.37 ± 42.10 | 12 | 145.38 ± 46.47 | 10 | 12 | 0.00114 | 0.919 | 0.343 | 1.19 | 0.282 | | * | | * | |
| 599 | 51 | Central medial nucleus of the thalamus | 7.23 ± 3.19 | 15 | 21.54 ± 12.23 | 14 | 33.04 ± 10.89 | 12 | 76.12 ± 26.92 | 10 | 8.21 | 0.00622 | 3.55 | 0.0659 | 1.1 | 0.3 | | ** | | * | |
| 907 | 51 | Paracentral nucleus | 5.75 ± 2.52 | 14 | 3.91 ± 2.03 | 13 | 63.98 ± 20.37 | 12 | 118.79 ± 36.69 | 10 | 22 | 0.0000257 | 1.39 | 0.244 | 2.45 | 0.124 | | *** | | *** | |
| 575 | 51 | Central lateral nucleus of the thalamus | 30.41 ± 16.11 | 14 | 20.61 ± 7.83 | 14 | 48.24 ± 8.63 | 12 | 100.20 ± 33.71 | 10 | 7.37 | 0.00931 | 0.771 | 0.384 | 3.15 | 0.0823 | | * | | * | |
| 930 | 51 | Parafascicular nucleus | 11.84 ± 5.51 | 10 | 3.78 ± 1.21 | 10 | 88.11 ± 14.82 | 8 | 116.19 ± 30.95 | 7 | 42 | 3.13E-07 | 0.0919 | 0.764 | 1.56 | 0.221 | ** | *** | ** | **** | |
| 262 | 856 | Reticular nucleus of the thalamus | 38.38 ± 20.08 | 15 | 20.33 ± 9.57 | 14 | 35.30 ± 9.20 | 12 | 54.39 ± 11.66 | 10 | 0.956 | 0.333 | 0.0296 | 0.864 | 1.62 | 0.21 | | | | | |
| 1014 | 856 | Geniculate group, ventral thalamus | 24.28 ± 10.71 | 14 | 61.35 ± 45.68 | 15 | 37.65 ± 9.63 | 12 | 97.66 ± 51.54 | 10 | 0.491 | 0.487 | 1.74 | 0.194 | 0.108 | 0.743 | | | | | |
| 27 | 1014 | Intergeniculate leaflet of the lateral geniculate complex | 162.58 ± 132.15 | 12 | 37.19 ± 24.92 | 10 | 87.57 ± 46.12 | 10 | 66.59 ± 19.88 | 8 | 0.11 | 0.743 | 0.847 | 0.364 | 0.373 | 0.545 | | | | | |
| 563807439 | 1014 | Intermediate geniculate nucleus | 36.54 ± 21.76 | 6 | 57.87 ± 43.27 | 6 | 65.13 ± 42.31 | 4 | 26.08 ± 15.99 | 4 | 0.00206 | 0.964 | 0.0067 | 0.936 | 0.737 | 0.403 | | | | | |
| 178 | 1014 | Ventral part of the lateral geniculate complex | 48.55 ± 23.98 | 14 | 122.35 ± 91.96 | 15 | 78.23 ± 19.60 | 12 | 202.64 ± 102.79 | 10 | 0.594 | 0.445 | 1.77 | 0.19 | 0.13 | 0.72 | | | | | |
| 321 | 1014 | Subgeniculate nucleus | 85.30 ± 55.75 | 8 | 16.92 ± 9.46 | 10 | 39.26 ± 24.73 | 9 | 61.06 ± 28.13 | 8 | 1.92E-05 | 0.997 | 0.596 | 0.446 | 2.02 | 0.165 | | | | | |
| 958 | 856 | Epithalamus | 25.39 ± 6.95 | 15 | 17.04 ± 4.36 | 15 | 36.30 ± 15.33 | 12 | 67.75 ± 30.83 | 10 | 4.12 | 0.0479 | 0.239 | 0.627 | 1.84 | 0.182 | | | | | |
| 483 | 958 | Medial habenula | 50.41 ± 13.56 | 15 | 36.77 ± 10.69 | 15 | 122.13 ± 55.55 | 12 | 204.29 ± 98.23 | 10 | 6.23 | 0.0161 | 0.215 | 0.645 | 1.04 | 0.313 | | | | | |
| 186 | 958 | Lateral habenula | 47.05 ± 18.22 | 15 | 26.02 ± 7.44 | 14 | 17.33 ± 4.61 | 12 | 70.46 ± 35.80 | 10 | 0.071 | 0.791 | 0.345 | 0.56 | 4.06 | 0.0496 | | | | | |
| 1097 | 1129 | Hypothalamus | 25.06 ± 14.00 | 14 | 11.80 ± 2.92 | 13 | 18.49 ± 4.27 | 12 | 29.52 ± 4.80 | 10 | 0.299 | 0.587 | 0.0843 | 0.773 | 1.93 | 0.172 | | | | | |
| 157 | 1097 | Periventricular zone | 66.69 ± 39.27 | 15 | 25.96 ± 7.32 | 14 | 19.00 ± 6.70 | 12 | 55.92 ± 17.27 | 10 | 0.21 | 0.649 | 0.0811 | 0.777 | 2.41 | 0.128 | | | | | |
| 390 | 157 | Supraoptic nucleus | 34.50 ± 19.28 | 9 | 52.06 ± 22.96 | 6 | 30.90 ± 17.20 | 9 | 46.21 ± 32.79 | 4 | 0.0414 | 0.84 | 0.582 | 0.453 | 0.00246 | 0.961 | | | | | |
| 38 | 157 | Paraventricular hypothalamic nucleus | 41.95 ± 17.23 | 13 | 25.10 ± 18.09 | 8 | 18.40 ± 5.21 | 12 | 46.33 ± 13.36 | 8 | 0.178 | 0.676 | 0.119 | 0.732 | 2.31 | 0.137 | | | | | |
| 30 | 157 | Periventricular hypothalamic nucleus, anterior part | 24.98 ± 22.64 | 8 | 28.91 ± 16.08 | 6 | 14.70 ± 10.56 | 7 | 47.16 ± 36.62 | 5 | 0.00653 | 0.936 | 0.595 | 0.449 | 0.415 | 0.526 | | | | | |
| 118 | 157 | Periventricular hypothalamic nucleus, intermediate part | 43.35 ± 23.12 | 13 | 20.81 ± 8.37 | 14 | 7.00 ± 2.03 | 12 | 32.33 ± 10.36 | 10 | 0.894 | 0.35 | 0.000448 | 0.983 | 2.92 | 0.0941 | | | | | |
| 223 | 157 | Arcuate hypothalamic nucleus | 318.68 ± 171.74 | 13 | 92.06 ± 29.42 | 14 | 189.43 ± 124.48 | 10 | 193.24 ± 56.31 | 9 | 0.0184 | 0.893 | 1.3 | 0.261 | 0.972 | 0.33 | | | | | |
| 141 | 1097 | Periventricular region | 86.06 ± 40.49 | 14 | 30.77 ± 14.28 | 15 | 15.53 ± 6.08 | 12 | 35.96 ± 8.50 | 10 | 1.93 | 0.172 | 0.725 | 0.399 | 2.38 | 0.13 | | | | | |
| 830 | 141 | Dorsomedial nucleus of the hypothalamus | 70.98 ± 53.60 | 12 | 37.20 ± 20.76 | 13 | 14.88 ± 4.13 | 10 | 30.42 ± 11.27 | 9 | 0.95 | 0.336 | 0.118 | 0.733 | 0.573 | 0.453 | | | | | |
| 126 | 141 | Periventricular hypothalamic nucleus, posterior part | 378.70 ± 196.82 | 9 | 152.59 ± 98.81 | 9 | 115.81 ± 59.39 | 6 | 131.20 ± 44.61 | 8 | 1.04 | 0.317 | 1.03 | 0.318 | 0.857 | 0.363 | | | | | |
| 347 | 141 | Subparaventricular zone | 28.36 ± 21.05 | 13 | 20.04 ± 8.81 | 8 | 18.88 ± 6.59 | 11 | 39.34 ± 21.22 | 7 | 0.00905 | 0.925 | 0.0843 | 0.773 | 0.685 | 0.413 | | | | | |
| 286 | 141 | Suprachiasmatic nucleus | 105.32 ± 77.86 | 9 | 27.97 ± 10.65 | 6 | 25.06 ± 13.84 | 8 | 9.76 ± 9.76 | 4 | 1.13 | 0.299 | 0.704 | 0.41 | 0.3 | 0.589 | | | | | |
| 467 | 1097 | Hypothalamic medial zone | 35.50 ± 22.22 | 15 | 20.25 ± 5.68 | 15 | 23.48 ± 8.93 | 12 | 34.73 ± 6.82 | 10 | 0.00137 | 0.971 | 0.0844 | 0.773 | 0.85 | 0.361 | | | | | |
| 88 | 467 | Anterior hypothalamic nucleus | 16.06 ± 6.92 | 14 | 28.22 ± 13.25 | 11 | 20.19 ± 4.94 | 12 | 25.68 ± 7.62 | 9 | 0.0205 | 0.887 | 1.11 | 0.298 | 0.148 | 0.703 | | | | | |
| 331 | 467 | Mammillary body | 61.08 ± 36.25 | 10 | 22.45 ± 5.47 | 11 | 53.35 ± 28.51 | 10 | 79.23 ± 23.24 | 8 | 0.813 | 0.373 | 0.179 | 0.675 | 1.56 | 0.22 | | | | | |
| 210 | 331 | Lateral mammillary nucleus | 175.64 ± 131.22 | 7 | 32.86 ± 17.39 | 8 | 65.32 ± 49.93 | 7 | 58.14 ± 16.55 | 8 | 0.327 | 0.572 | 1.27 | 0.27 | 1.04 | 0.318 | | | | | |
| 491 | 331 | Medial mammillary nucleus | 39.65 ± 16.02 | 7 | 12.27 ± 9.40 | 8 | 81.71 ± 46.24 | 6 | 100.44 ± 13.74 | 7 | 8.49 | 0.00761 | 0.0608 | 0.807 | 1.01 | 0.326 | | | | * | |
| 732 | 491 | Medial mammillary nucleus, median part | 80.55 ± 42.06 | 7 | 7.02 ± 7.02 | 8 | 142.80 ± 86.57 | 6 | 100.13 ± 33.49 | 7 | 2.97 | 0.0975 | 1.66 | 0.21 | 0.113 | 0.739 | | | | | |
| 606826647 | 491 | Medial mammillary nucleus, lateral part | 75.70 ± 30.84 | 7 | 18.15 ± 11.53 | 8 | 112.24 ± 63.78 | 6 | 177.88 ± 22.02 | 7 | 9.27 | 0.00559 | 0.000014 | 0.997 | 3.32 | 0.0808 | | | | ** | |
| 606826651 | 491 | Medial mammillary nucleus, medial part | 79.06 ± 36.07 | 7 | 47.19 ± 36.38 | 8 | 219.77 ± 131.63 | 6 | 309.25 ± 61.29 | 6 | 8.31 | 0.00841 | 0.0489 | 0.827 | 0.74 | 0.399 | | | | | |
| 525 | 331 | Supramammillary nucleus | 397.32 ± 248.17 | 9 | 133.36 ± 35.24 | 9 | 386.22 ± 229.90 | 9 | 344.77 ± 184.63 | 8 | 0.248 | 0.622 | 0.669 | 0.42 | 0.327 | 0.572 | | | | | |
| 557 | 331 | Tuberomammillary nucleus | 48.60 ± 40.88 | 10 | 44.08 ± 22.29 | 9 | 7.85 ± 3.50 | 8 | 17.46 ± 8.24 | 7 | 1.54 | 0.224 | 0.00504 | 0.944 | 0.0659 | 0.799 | | | | | |
| 1126 | 557 | Tuberomammillary nucleus, dorsal part | 6.04 ± 4.24 | 8 | 92.49 ± 56.63 | 7 | 30.36 ± 26.27 | 5 | 28.52 ± 18.42 | 6 | 0.38 | 0.544 | 2.03 | 0.169 | 1.69 | 0.207 | | | | | |
| 1 | 557 | Tuberomammillary nucleus, ventral part | 103.48 ± 84.74 | 10 | 25.98 ± 19.07 | 7 | 12.90 ± 4.04 | 8 | 19.98 ± 16.55 | 7 | 0.938 | 0.341 | 0.53 | 0.473 | 0.582 | 0.452 | | | | | |
| 980 | 467 | Dorsal premammillary nucleus | 213.87 ± 180.59 | 10 | 61.05 ± 34.13 | 8 | 23.91 ± 12.21 | 7 | 70.39 ± 15.60 | 8 | 0.656 | 0.424 | 0.387 | 0.539 | 0.774 | 0.386 | | | | | |
| 1004 | 467 | Ventral premammillary nucleus | 245.89 ± 184.72 | 10 | 68.42 ± 26.38 | 9 | 23.91 ± 9.66 | 9 | 74.02 ± 20.23 | 8 | 1.2 | 0.282 | 0.442 | 0.511 | 1.18 | 0.286 | | | | | |
| 63 | 467 | Paraventricular hypothalamic nucleus, descending division | 33.70 ± 21.58 | 12 | 16.24 ± 8.64 | 11 | 28.75 ± 14.78 | 10 | 21.69 ± 12.73 | 8 | 0.000118 | 0.991 | 0.639 | 0.429 | 0.102 | 0.751 | | | | | |
| 693 | 467 | Ventromedial hypothalamic nucleus | 64.66 ± 32.39 | 14 | 31.33 ± 15.55 | 12 | 33.32 ± 23.51 | 10 | 21.61 ± 5.55 | 9 | 0.737 | 0.396 | 0.998 | 0.324 | 0.191 | 0.665 | | | | | |
| 946 | 467 | Posterior hypothalamic nucleus | 109.07 ± 75.76 | 14 | 51.35 ± 22.42 | 14 | 42.09 ± 11.66 | 10 | 52.51 ± 9.50 | 8 | 0.493 | 0.486 | 0.361 | 0.551 | 0.459 | 0.502 | | | | | |
| 290 | 1097 | Hypothalamic lateral zone | 16.48 ± 8.91 | 15 | 11.98 ± 2.79 | 15 | 28.02 ± 4.98 | 12 | 40.68 ± 7.00 | 10 | 9.05 | 0.00418 | 0.0822 | 0.776 | 1.72 | 0.197 | | | | * | |
| 194 | 290 | Lateral hypothalamic area | 37.01 ± 21.70 | 15 | 40.30 ± 9.14 | 15 | 44.74 ± 11.43 | 11 | 61.46 ± 17.65 | 10 | 0.743 | 0.393 | 0.271 | 0.605 | 0.165 | 0.687 | | | | | |
| 226 | 290 | Lateral preoptic area | 16.14 ± 10.21 | 4 | 34.40 ± 15.38 | 5 | 100.61 ± 86.90 | 6 | 139.46 ± 47.87 | 5 | 2.54 | 0.131 | 0.116 | 0.738 | 0.0298 | 0.865 | | | | | |
| 364 | 290 | Parasubthalamic nucleus | 66.15 ± 49.11 | 12 | 15.26 ± 4.77 | 12 | 37.78 ± 14.03 | 10 | 51.80 ± 21.80 | 8 | 0.00433 | 0.948 | 0.595 | 0.445 | 1.12 | 0.297 | | | | | |
| 576073704 | 290 | Perifornical nucleus | 22.84 ± 10.08 | 15 | 13.52 ± 5.12 | 13 | 41.68 ± 11.09 | 12 | 42.27 ± 10.53 | 8 | 5.68 | 0.0216 | 0.494 | 0.486 | 0.256 | 0.615 | | | | | |
| 173 | 290 | Retrochiasmatic area | 72.94 ± 40.54 | 12 | 9.17 ± 5.43 | 6 | 19.12 ± 5.10 | 9 | 24.00 ± 11.50 | 5 | 0.894 | 0.353 | 1.05 | 0.315 | 1.06 | 0.313 | | | | | |
| 470 | 290 | Subthalamic nucleus | 27.44 ± 24.44 | 10 | 8.09 ± 3.51 | 15 | 44.92 ± 10.07 | 11 | 88.79 ± 27.16 | 9 | 9.31 | 0.00398 | 0.0118 | 0.914 | 3.69 | 0.0618 | | | | ** | |
| 614 | 290 | Tuberal nucleus | 78.77 ± 50.12 | 14 | 24.59 ± 9.62 | 13 | 32.32 ± 8.79 | 10 | 41.42 ± 7.67 | 9 | 0.257 | 0.615 | 0.78 | 0.382 | 0.969 | 0.33 | | | | | |
| 797 | 290 | Zona incerta | 17.43 ± 6.44 | 15 | 13.00 ± 3.81 | 15 | 82.26 ± 12.95 | 12 | 119.90 ± 24.67 | 10 | 49.3 | 6.89E-09 | 0.636 | 0.429 | 3.03 | 0.0883 | ** | **** | *** | **** | |
| 804 | 797 | Fields of Forel | 14.47 ± 9.86 | 10 | 6.76 ± 3.09 | 9 | 80.49 ± 22.78 | 9 | 147.48 ± 30.36 | 8 | 29.9 | 5.05E-06 | 2.16 | 0.151 | 4.06 | 0.0525 | | **** | * | **** | |
| 10671 | 1097 | Median eminence | 388.51 ± 217.62 | 12 | 165.39 ± 117.44 | 13 | 166.01 ± 141.29 | 10 | 633.30 ± 276.05 | 9 | 0.385 | 0.539 | 0.135 | 0.715 | 3.27 | 0.078 | | | | | |
| 313 | 343 | Midbrain | 17.42 ± 9.95 | 13 | 15.86 ± 10.31 | 14 | 19.87 ± 5.91 | 12 | 41.98 ± 10.21 | 9 | 2.01 | 0.163 | 0.63 | 0.432 | 1.53 | 0.222 | | | | | |
| 339 | 313 | Midbrain, sensory related | 8.74 ± 7.90 | 11 | 8.88 ± 3.23 | 10 | 119.58 ± 79.23 | 10 | 15.52 ± 5.10 | 7 | 2.34 | 0.135 | 1.39 | 0.247 | 1.48 | 0.232 | | | | | |
| 302 | 339 | Superior colliculus, sensory related | 23.83 ± 21.28 | 6 | 20.46 ± 5.99 | 7 | 37.38 ± 35.61 | 6 | 32.96 ± 7.16 | 5 | 0.371 | 0.549 | 0.054 | 0.819 | 0.000593 | 0.981 | | | | | |
| 851 | 302 | Superior colliculus, optic layer | 34.78 ± 34.78 | 4 | 14.80 ± 11.23 | 7 | 91.21 ± 83.67 | 6 | 103.11 ± 38.32 | 5 | 2.02 | 0.173 | 0.11 | 0.744 | 0.093 | 0.764 | | | | | |
| 834 | 302 | Superior colliculus, zonal layer | 131.27 ± 116.17 | 5 | 100.49 ± 29.69 | 7 | 0.99 ± 0.99 | 6 | 74.03 ± 21.32 | 5 | 1.99 | 0.174 | 0.295 | 0.593 | 0.913 | 0.351 | | | | | |
| 599626923 | 339 | Subcommissural organ | 23.27 ± 23.27 | 11 | 0.00 ± 0.00 | 8 | 473.17 ± 332.90 | 10 | 31.32 ± 23.01 | 7 | 2.17 | 0.15 | 1.38 | 0.248 | 1.22 | 0.278 | | | | | |
| 323 | 313 | Midbrain, motor related | 16.78 ± 10.94 | 13 | 3.79 ± 1.17 | 14 | 14.07 ± 4.83 | 12 | 23.96 ± 5.13 | 8 | 1.19 | 0.282 | 0.406 | 0.527 | 2.69 | 0.108 | | | | | |
| 381 | 323 | Substantia nigra, reticular part | 29.35 ± 27.22 | 6 | 3.91 ± 2.69 | 7 | 10.73 ± 4.94 | 7 | 22.81 ± 10.68 | 7 | 0.00432 | 0.948 | 0.194 | 0.664 | 1.92 | 0.179 | | | | | |
| 128 | 323 | Midbrain reticular nucleus | 12.61 ± 7.67 | 8 | 6.14 ± 2.73 | 9 | 107.45 ± 49.14 | 9 | 115.01 ± 28.23 | 7 | 11.6 | 0.00196 | 0.0928 | 0.763 | 0.0552 | 0.816 | | | | | |
| 795 | 323 | Periaqueductal gray | 32.38 ± 17.10 | 13 | 9.55 ± 3.64 | 13 | 15.82 ± 6.06 | 12 | 24.34 ± 4.73 | 8 | 0.0633 | 0.803 | 0.762 | 0.388 | 2.12 | 0.153 | | | | | |
| 50 | 795 | Precommissural nucleus | 49.97 ± 29.18 | 10 | 12.55 ± 6.94 | 12 | 25.40 ± 14.83 | 12 | 109.77 ± 88.66 | 8 | 0.742 | 0.394 | 0.159 | 0.692 | 2.61 | 0.115 | | | | | |
| 1100 | 323 | Pretectal region | 28.89 ± 21.88 | 12 | 4.88 ± 1.61 | 10 | 22.25 ± 6.76 | 10 | 37.75 ± 8.15 | 7 | 0.511 | 0.479 | 0.265 | 0.61 | 1.82 | 0.186 | | | | | |
| 215 | 1100 | Anterior pretectal nucleus | 54.45 ± 44.47 | 12 | 9.32 ± 3.19 | 10 | 54.50 ± 17.16 | 10 | 86.13 ± 19.55 | 7 | 1.2 | 0.28 | 0.202 | 0.656 | 1.59 | 0.216 | | | | | |
| 628 | 1100 | Nucleus of the optic tract | 24.98 ± 6.79 | 8 | 8.54 ± 6.16 | 7 | 27.67 ± 12.32 | 9 | 44.98 ± 22.42 | 7 | 1.72 | 0.201 | 0.000661 | 0.98 | 1.5 | 0.231 | | | | | |
| 634 | 1100 | Nucleus of the posterior commissure | 83.32 ± 56.59 | 9 | 10.91 ± 5.09 | 7 | 27.19 ± 10.37 | 8 | 46.40 ± 16.32 | 7 | 0.181 | 0.674 | 0.665 | 0.422 | 1.75 | 0.198 | | | | | |
| 706 | 1100 | Olivary pretectal nucleus | 51.72 ± 46.14 | 6 | 21.61 ± 13.07 | 7 | 23.63 ± 9.81 | 6 | 42.37 ± 21.85 | 7 | 0.00485 | 0.945 | 0.0488 | 0.827 | 0.902 | 0.353 | | | | | |
| 1061 | 1100 | Posterior pretectal nucleus | 10.80 ± 4.26 | 8 | 6.82 ± 3.66 | 7 | 22.34 ± 11.49 | 8 | 41.74 ± 22.01 | 7 | 3.35 | 0.0786 | 0.394 | 0.536 | 0.904 | 0.35 | | | | | |
| 549009203 | 1100 | Retroparafascicular nucleus | 0.00 ± 0.00 | 6 | 0.00 ± 0.00 | 7 | 124.14 ± 79.67 | 5 | 70.20 ± 36.21 | 5 | 6.49 | 0.0197 | 0.526 | 0.477 | 0.509 | 0.484 | | | | | |

| | | | | | | | | | | | | | | | | | | | | |
|---|---|---|---|---|---|---|---|---|---|---|---|---|---|---|---|---|---|---|---|---|
| 348 | 313 | Midbrain, behavioral state related | 31.17 ± 31.17 | 5 | 11.60 ± 9.20 | 6 | 4.56 ± 1.76 | 6 | 25.41 ± 13.10 | 5 | 0.158 | 0.696 | 0.00583 | 0.94 | 1.57 | 0.226 | | | | |
| 374 | 348 | Substantia nigra, compact part | 62.34 ± 62.34 | 5 | 0.00 ± 0.00 | 4 | 9.17 ± 3.51 | 6 | 27.33 ± 23.15 | 5 | 0.258 | 0.618 | 0.294 | 0.595 | 1.43 | 0.249 | | | | |
| 967 | 1009 | cranial nerves | 18.92 ± 8.80 | 15 | 8.96 ± 3.61 | 15 | 13.68 ± 4.13 | 12 | 86.71 ± 39.32 | 10 | 4.4 | 0.0412 | 2.14 | 0.15 | 6.51 | 0.014 | | * | ** | * |
| 1009 | 997 | fiber tracts | 12.93 ± 4.34 | 15 | 11.90 ± 2.84 | 15 | 24.10 ± 5.18 | 12 | 52.17 ± 9.51 | 10 | 21.9 | 0.0000242 | 3.68 | 0.0612 | 7.41 | 0.00902 | | **** | **** | ** |
| 21 | 840 | lateral olfactory tract, general | 18.61 ± 7.10 | 15 | 16.48 ± 6.18 | 15 | 7.58 ± 3.95 | 12 | 13.55 ± 6.23 | 10 | 1.3 | 0.259 | 0.0661 | 0.798 | 0.411 | 0.524 | | | | |
| 665 | 21 | lateral olfactory tract, body | 37.23 ± 14.20 | 15 | 32.97 ± 12.36 | 15 | 15.15 ± 7.91 | 12 | 27.09 ± 12.47 | 10 | 1.3 | 0.259 | 0.0661 | 0.798 | 0.411 | 0.524 | | | | |
| 840 | 967 | olfactory nerve | 11.90 ± 4.32 | 15 | 9.64 ± 2.28 | 15 | 7.23 ± 2.47 | 12 | 25.95 ± 11.60 | 10 | 0.94 | 0.337 | 1.4 | 0.243 | 3.73 | 0.0595 | | | | |
| 848 | 967 | optic nerve | 47.68 ± 26.63 | 15 | 21.23 ± 9.93 | 15 | 29.74 ± 10.32 | 12 | 203.66 ± 96.87 | 10 | 3.5 | 0.0673 | 1.8 | 0.186 | 5.92 | 0.0188 | | | * | * |
| 900 | 840 | anterior commissure, olfactory limb | 21.04 ± 8.80 | 15 | 19.06 ± 8.53 | 14 | 22.75 ± 8.82 | 12 | 118.79 ± 56.55 | 9 | 4.36 | 0.0423 | 2.79 | 0.102 | 4.92 | 0.0315 | | * | * | * |
| 117 | 848 | optic chiasm | 6.81 ± 6.81 | 10 | 1.84 ± 1.20 | 8 | 10.02 ± 5.28 | 9 | 67.37 ± 67.37 | 4 | 2.17 | 0.153 | 0.77 | 0.388 | 2.95 | 0.0975 | | | | |
| 125 | 848 | optic tract | 112.59 ± 64.93 | 15 | 48.15 ± 29.04 | 15 | 61.62 ± 30.12 | 12 | 525.56 ± 297.51 | 10 | 2.64 | 0.111 | 1.52 | 0.224 | 4.63 | 0.0366 | | | * | |
| 158 | 832 | posterior commissure | 24.88 ± 22.05 | 9 | 4.62 ± 3.10 | 7 | 7.57 ± 7.57 | 9 | 35.54 ± 31.11 | 7 | 0.0402 | 0.843 | 0.0409 | 0.841 | 1.6 | 0.216 | | | | |
| 832 | 967 | oculomotor nerve | 12.44 ± 11.02 | 9 | 8.38 ± 6.47 | 9 | 3.78 ± 3.78 | 9 | 17.77 ± 15.56 | 7 | 0.000606 | 0.981 | 0.212 | 0.649 | 0.893 | 0.352 | | | | |
| 916 | 848 | brachium of the superior colliculus | 66.80 ± 43.16 | 11 | 62.85 ± 31.42 | 9 | 125.08 ± 82.93 | 10 | 154.96 ± 58.14 | 7 | 1.55 | 0.222 | 0.0213 | 0.885 | 0.0819 | 0.777 | | | | |
| 792 | 967 | dorsal roots | 4.94 ± 2.79 | 10 | 1.68 ± 0.83 | 11 | 13.44 ± 5.01 | 10 | 24.10 ± 7.17 | 8 | 13.1 | 0.000907 | 0.214 | 0.647 | 2.76 | 0.106 | | * | ** | |
| 932 | 792 | cervicothalamic tract | 7.42 ± 4.18 | 10 | 2.51 ± 1.24 | 11 | 20.15 ± 7.52 | 10 | 36.15 ± 10.76 | 8 | 13.1 | 0.000907 | 0.214 | 0.647 | 2.76 | 0.106 | | * | ** | |
| 697 | 932 | medial lemniscus | 14.83 ± 8.37 | 10 | 5.03 ± 2.48 | 11 | 40.31 ± 15.03 | 10 | 72.29 ± 21.52 | 8 | 13.1 | 0.000907 | 0.214 | 0.647 | 2.76 | 0.106 | | * | ** | |
| 579 | 956 | external capsule | 31.70 ± 14.00 | 15 | 17.61 ± 3.97 | 15 | 58.10 ± 9.37 | 12 | 89.02 ± 18.35 | 10 | 16 | 0.000215 | 0.0542 | 0.817 | 3.56 | 0.0654 | | ** | *** | |
| 776 | 983 | corpus callosum | 21.15 ± 7.45 | 15 | 26.97 ± 7.25 | 15 | 43.56 ± 7.93 | 12 | 98.42 ± 24.90 | 10 | 14.6 | 0.000383 | 4.29 | 0.0438 | 4.2 | 0.0459 | | *** | *** | * |
| 956 | 776 | corpus callosum, anterior forceps | 15.06 ± 6.73 | 15 | 14.81 ± 5.89 | 15 | 25.98 ± 4.83 | 12 | 54.57 ± 16.23 | 10 | 8.43 | 0.00556 | 1.67 | 0.203 | 2.89 | 0.0954 | | * | * | |
| 983 | 1009 | lateral forebrain bundle system | 13.41 ± 5.08 | 15 | 12.36 ± 2.96 | 15 | 19.98 ± 3.62 | 12 | 51.01 ± 12.53 | 10 | 12.5 | 0.000932 | 3.56 | 0.0653 | 6.74 | 0.0124 | | *** | *** | ** |
| 484682512 | 1009 | supra-callosal cerebral white matter | 27.23 ± 12.53 | 15 | 38.47 ± 12.02 | 15 | 32.88 ± 9.56 | 12 | 129.40 ± 65.28 | 10 | 2.81 | 0.1 | 2.76 | 0.103 | 2.4 | 0.128 | | | | |
| 6 | 784 | internal capsule | 20.14 ± 9.44 | 15 | 9.56 ± 3.17 | 15 | 14.12 ± 3.55 | 12 | 76.21 ± 33.40 | 10 | 3.99 | 0.0514 | 1.78 | 0.188 | 6.52 | 0.0139 | | * | * | * |
| 784 | 983 | corticospinal tract | 11.90 ± 6.49 | 15 | 5.10 ± 1.65 | 15 | 6.88 ± 1.87 | 12 | 36.20 ± 12.74 | 10 | 3.63 | 0.0628 | 1.58 | 0.215 | 8.07 | 0.00659 | | | ** | * |
| 924 | 784 | cerebal peduncle | 60.91 ± 52.75 | 9 | 29.21 ± 24.12 | 9 | 11.75 ± 7.14 | 9 | 66.50 ± 15.26 | 7 | 0.077 | 0.783 | 0.084 | 0.774 | 1.84 | 0.185 | | | | |
| 964 | 776 | corpus callosum, extreme capsule | 14.66 ± 9.68 | 15 | 15.82 ± 6.94 | 15 | 51.23 ± 15.66 | 12 | 59.83 ± 16.44 | 10 | 11.3 | 0.00153 | 0.0453 | 0.832 | 0.097 | 0.757 | | | | |
| 971 | 776 | corpus callosum, posterior forceps | 87.24 ± 36.94 | 12 | 70.89 ± 24.71 | 10 | 138.38 ± 35.45 | 10 | 372.80 ± 125.50 | 8 | 7.77 | 0.00844 | 2.59 | 0.116 | 4.51 | 0.0407 | | ** | ** | |
| 986 | 776 | corpus callosum, splenium | 86.26 ± 33.95 | 13 | 94.78 ± 35.24 | 15 | 207.78 ± 49.78 | 12 | 356.71 ± 118.77 | 10 | 10 | 0.00275 | 0.853 | 0.361 | 1.36 | 0.249 | | * | * | |
| 1108 | 776 | genu of corpus callosum | 18.60 ± 11.16 | 14 | 27.92 ± 10.22 | 10 | 30.33 ± 13.05 | 10 | 92.22 ± 47.55 | 9 | 2.44 | 0.126 | 2.4 | 0.13 | 1.34 | 0.254 | | | | |
| 484682516 | 776 | corpus callosum, body | 46.99 ± 16.65 | 15 | 68.39 ± 31.10 | 13 | 80.13 ± 22.82 | 12 | 304.41 ± 125.95 | 10 | 5.29 | 0.026 | 4 | 0.0514 | 3.4 | 0.0718 | | * | * | * |
| 896 | 983 | thalamus related | 25.07 ± 11.40 | 15 | 13.88 ± 3.67 | 15 | 17.98 ± 3.75 | 12 | 55.80 ± 16.35 | 10 | 2.82 | 0.0995 | 0.848 | 0.362 | 6.5 | 0.0141 | | | * | |
| 1092 | 896 | external medullary lamina of the thalamus | 28.89 ± 14.01 | 15 | 21.00 ± 15.18 | 13 | 26.46 ± 5.41 | 12 | 55.76 ± 14.99 | 10 | 1.2 | 0.279 | 0.388 | 0.536 | 1.92 | 0.172 | | | | |
| 102 | 760 | nigrostriatal tract | 22.97 ± 12.64 | 15 | 28.33 ± 9.91 | 15 | 79.72 ± 19.90 | 12 | 87.62 ± 24.98 | 10 | 12.6 | 0.00089 | 0.057 | 0.812 | 0.00604 | 0.938 | | * | | |
| 760 | 1000 | cerebral nuclei related | 11.49 ± 6.32 | 15 | 14.17 ± 4.95 | 15 | 39.86 ± 9.95 | 12 | 43.81 ± 12.49 | 10 | 12.6 | 0.00089 | 0.057 | 0.812 | 0.00604 | 0.938 | | * | | |
| 1000 | 1009 | extrapyramidal fiber systems | 7.66 ± 4.21 | 15 | 8.01 ± 2.78 | 15 | 26.57 ± 6.63 | 12 | 28.58 ± 8.46 | 10 | 13.4 | 0.000625 | 0.00104 | 0.974 | 0.0236 | 0.879 | | * | | |
| 484682520 | 896 | optic radiation | 58.17 ± 27.36 | 14 | 31.72 ± 8.64 | 15 | 36.20 ± 9.82 | 12 | 128.42 ± 40.85 | 10 | 2.3 | 0.136 | 0.952 | 0.334 | 6.54 | 0.0138 | | | * | |
| 484682524 | 896 | auditory radiation | 44.18 ± 23.30 | 14 | 11.58 ± 5.39 | 15 | 37.57 ± 8.32 | 12 | 66.87 ± 16.29 | 10 | 2.28 | 0.137 | 0.24 | 0.626 | 4.06 | 0.0496 | | | | |
| 768 | 991 | cerebrum related | 21.26 ± 6.17 | 15 | 27.71 ± 8.87 | 15 | 67.15 ± 16.18 | 12 | 114.04 ± 20.53 | 10 | 26.5 | 4.83E-06 | 2.72 | 0.105 | 2.56 | 0.116 | | **** | *** | |
| 884 | 768 | amygdalar capsule | 22.97 ± 16.16 | 15 | 17.95 ± 7.37 | 15 | 50.42 ± 8.88 | 12 | 52.42 ± 14.74 | 10 | 5.99 | 0.0181 | 0.0765 | 0.783 | 0.0776 | 0.782 | | | | |
| 908 | 768 | anterior commissure, temporal limb | 6.59 ± 5.04 | 10 | 4.44 ± 4.44 | 4 | 9.29 ± 4.96 | 7 | 155.35 ± 114.27 | 4 | 2.1 | 0.162 | 3.72 | 0.0675 | 3.87 | 0.0624 | | * | | |
| 940 | 768 | cingulum bundle | 44.69 ± 17.01 | 15 | 32.57 ± 11.61 | 15 | 89.45 ± 35.80 | 12 | 192.93 ± 73.04 | 10 | 8.16 | 0.00633 | 0.871 | 0.355 | 2.74 | 0.104 | | * | * | |
| 991 | 1009 | medial forebrain bundle system | 15.44 ± 4.66 | 15 | 19.01 ± 5.58 | 15 | 46.67 ± 11.15 | 12 | 78.22 ± 14.20 | 10 | 26.3 | 5.14E-06 | 2.44 | 0.125 | 2.6 | 0.113 | | **** | *** | |
| 1099 | 768 | fornix system | 29.15 ± 8.47 | 15 | 44.57 ± 15.10 | 15 | 108.91 ± 26.25 | 12 | 178.56 ± 33.94 | 10 | 26.2 | 5.29E-06 | 2.73 | 0.105 | 1.73 | 0.195 | * | **** | *** | |
| 436 | 737 | columns of the fornix | 25.98 ± 14.09 | 15 | 29.16 ± 10.37 | 14 | 64.92 ± 11.99 | 12 | 86.63 ± 16.91 | 10 | 12.6 | 0.000905 | 0.544 | 0.465 | 0.474 | 0.495 | | * | * | |
| 466 | 1099 | alveus | 42.32 ± 7.57 | 15 | 99.47 ± 27.97 | 15 | 225.63 ± 41.40 | 12 | 391.64 ± 71.87 | 10 | 38.7 | 1.16E-07 | 6.15 | 0.0167 | 2.07 | 0.157 | ** | **** | **** | * |
| 603 | 1099 | fimbria | 73.25 ± 26.47 | 15 | 99.33 ± 59.27 | 15 | 228.80 ± 70.79 | 12 | 357.27 ± 95.99 | 10 | 10.7 | 0.00203 | 0.948 | 0.335 | 0.669 | 0.417 | | * | * | |
| 618 | 1099 | hippocampal commissures | 46.11 ± 16.09 | 15 | 59.70 ± 15.35 | 15 | 195.81 ± 48.76 | 12 | 339.58 ± 111.27 | 10 | 17.7 | 0.000111 | 1.42 | 0.24 | 1.68 | 0.202 | | ** | ** | |
| 737 | 1099 | postcommissural fornix | 12.99 ± 7.05 | 15 | 14.58 ± 5.18 | 14 | 32.46 ± 6.00 | 12 | 42.60 ± 8.47 | 10 | 12.2 | 0.00105 | 0.479 | 0.492 | 0.403 | 0.529 | | * | * | |
| 54 | 824 | medial forebrain bundle | 87.09 ± 59.44 | 9 | 107.97 ± 69.07 | 10 | 144.28 ± 43.15 | 8 | 84.58 ± 25.11 | 8 | 0.0829 | 0.775 | 0.0888 | 0.768 | 0.53 | 0.472 | | | | |
| 301 | 768 | stria terminalis | 107.60 ± 58.08 | 15 | 21.81 ± 14.12 | 15 | 61.58 ± 29.67 | 12 | 120.46 ± 36.42 | 10 | 0.312 | 0.579 | 0.423 | 0.518 | 3.22 | 0.0791 | | | | |
| 443 | 618 | dorsal hippocampal commissure | 92.15 ± 32.21 | 15 | 118.47 ± 30.95 | 15 | 391.90 ± 97.45 | 12 | 679.15 ± 222.55 | 10 | 17.8 | 0.000109 | 1.4 | 0.243 | 1.68 | 0.201 | | ** | ** | |
| 824 | 991 | hypothalamus related | 19.37 ± 8.94 | 15 | 14.95 ± 3.21 | 14 | 31.66 ± 7.10 | 12 | 50.29 ± 18.98 | 10 | 5.4 | 0.0244 | 0.243 | 0.625 | 1.35 | 0.252 | | | | |
| 484682528 | 301 | commissural branch of stria terminalis | 219.71 ± 202.36 | 14 | 5.67 ± 4.01 | 13 | 67.28 ± 28.84 | 12 | 106.33 ± 39.64 | 9 | 0.0983 | 0.755 | 0.718 | 0.402 | 1.07 | 0.306 | | | | |
| 349 | 824 | supraoptic commissures | 50.45 ± 36.05 | 14 | 28.14 ± 15.77 | 12 | 33.28 ± 22.92 | 12 | 8.01 ± 5.34 | 10 | 0.528 | 0.471 | 0.851 | 0.361 | 0.00335 | 0.954 | | | | |
| 46 | 824 | mammillary related | 18.49 ± 10.50 | 14 | 15.97 ± 4.82 | 15 | 41.00 ± 9.79 | 12 | 46.52 ± 15.87 | 10 | 6.77 | 0.0124 | 0.00473 | 0.945 | 0.157 | 0.694 | | | | |
| 681 | 46 | mammillotegmental tract | 72.63 ± 43.47 | 9 | 81.62 ± 38.40 | 10 | 37.08 ± 24.78 | 9 | 158.73 ± 99.79 | 7 | 0.122 | 0.729 | 1.27 | 0.269 | 1.16 | 0.29 | | | | |
| 690 | 46 | mammillothalamic tract | 31.97 ± 21.49 | 14 | 7.91 ± 3.26 | 14 | 45.85 ± 13.98 | 12 | 62.31 ± 14.13 | 10 | 4.78 | 0.0339 | 0.269 | 0.606 | 1.78 | 0.188 | | | | |
| 753 | 46 | principal mammillary tract | 74.67 ± 36.86 | 10 | 171.35 ± 65.76 | 9 | 258.27 ± 94.48 | 9 | 278.72 ± 136.47 | 8 | 2.95 | 0.0954 | 0.492 | 0.488 | 0.196 | 0.661 | | | | |
| 1083 | 824 | epithalamus related | 30.32 ± 14.68 | 15 | 16.47 ± 4.22 | 15 | 44.81 ± 15.84 | 12 | 83.95 ± 39.89 | 10 | 4.23 | 0.0452 | 0.124 | 0.726 | 1.89 | 0.176 | | | | |
| 595 | 1083 | fasciculus retroflexus | 24.38 ± 17.63 | 14 | 11.13 ± 3.92 | 15 | 29.97 ± 14.24 | 12 | 37.38 ± 13.66 | 10 | 1.37 | 0.248 | 0.165 | 0.687 | 0.604 | 0.441 | | | | |
| 611 | 1083 | habenular commissure | 61.83 ± 42.92 | 13 | 40.92 ± 23.96 | 15 | 208.57 ± 126.02 | 11 | 91.46 ± 38.69 | 9 | 2.25 | 0.14 | 1.12 | 0.296 | 0.521 | 0.474 | | | | |
| 802 | 1083 | stria medullaris | 90.06 ± 63.96 | 15 | 52.67 ± 18.18 | 13 | 130.89 ± 53.44 | 12 | 272.04 ± 149.28 | 10 | 2.59 | 0.114 | 0.275 | 0.602 | 1.36 | 0.249 | | | | |
