# Supplementary material for: Hyperactive neuronal networks facilitate tau spread in an Alzheimer’s disease mouse model
Source: bioRxiv. 2024 Dec 3:2024.12.01.625514. Preprint. [Version 2] doi: 10.1101/2024.12.01.625514 (PMC11642807; doi:10.1101/2024.12.01.625514)
Supplement: Supplementary file 3 [file media-3.pdf]

Region.ID	Parent ID	Region	WT.Sal	n	WT.PTZ	n	5X.Sal	n	5X.PTZ	n	Genotype F	Genotype p	Treatment F	Treatment p	Interaction F	Interaction p	p1	p2	p3	p4	p5	p6
8	997	Basic cell groups and regions	2.26 ± 0.51	15	5.17 ± 1.11	16	2.58 ± 0.44	12	9.32 ± 1.45	12	5.4	0.0241	22.2	0.0000192	3.86	0.0548			****		*	***
567	8	Cerebrum	2.49 ± 0.55	15	6.68 ± 1.56	16	2.71 ± 0.42	12	12.24 ± 2.00	12	4.91	0.0312	24.5	0.0000085	4.09	0.0484			****		*	****
688	567	Cerebral cortex	3.03 ± 0.68	15	8.54 ± 2.10	16	3.31 ± 0.53	12	15.97 ± 2.56	12	5.1	0.0282	25	7.13E-06	4.27	0.0439			****		*	****
695	688	Cortical plate	3.63 ± 0.82	15	10.38 ± 2.57	16	3.95 ± 0.64	12	19.10 ± 3.03	12	4.84	0.0324	25.1	6.88E-06	4.07	0.0489			****		*	****
315	695	Isocortex	5.04 ± 1.12	15	14.57 ± 3.57	16	6.16 ± 0.89	11	26.77 ± 4.49	12	5.14	0.0278	22.6	0.0000171	3.33	0.0739			****		*	***
500	315	Somatomotor areas	3.71 ± 1.00	15	18.48 ± 4.95	16	6.04 ± 0.87	11	37.68 ± 7.56	12	5.91	0.0187	23.8	0.0000113	3.42	0.0705			****		*	***
985	500	Primary motor area	5.74 ± 1.30	13	28.86 ± 8.51	16	8.09 ± 1.35	11	63.27 ± 11.88	11	6.41	0.0147	23.3	0.0000152	4.51	0.0391			****		**	****
993	500	Secondary motor area	8.40 ± 2.76	11	27.21 ± 6.25	16	11.40 ± 1.65	11	62.35 ± 11.15	11	9.32	0.00379	22.5	0.0000212	5.73	0.0209			****		**	****
453	315	Somatosensory areas	5.73 ± 1.41	15	20.09 ± 5.36	16	7.81 ± 1.09	11	33.35 ± 6.03	12	3.35	0.0731	20.2	0.0000413	1.68	0.201			***			**
322	453	Primary somatosensory area	8.57 ± 1.99	14	26.65 ± 7.19	16	10.42 ± 1.58	10	44.78 ± 8.12	11	3.07	0.0863	17.7	0.000117	1.86	0.179			***			**
353	322	Primary somatosensory area, nose	12.22 ± 3.12	10	28.02 ± 11.77	10	11.46 ± 3.12	8	46.69 ± 13.05	11	0.987	0.327	6.99	0.0122	0.974	0.33						
329	322	Primary somatosensory area, barrel field	15.56 ± 3.64	14	45.92 ± 12.98	16	19.13 ± 2.63	11	70.87 ± 13.14	11	2.06	0.158	15.1	0.000313	1.11	0.297			**			**
337	322	Primary somatosensory area, lower limb	13.30 ± 4.49	13	47.05 ± 18.71	12	14.62 ± 2.37	11	67.35 ± 13.58	11	0.809	0.373	13.2	0.000742	0.645	0.426			*			*
345	322	Primary somatosensory area, mouth	7.18 ± 1.45	13	33.31 ± 9.18	14	11.11 ± 1.94	10	66.08 ± 11.69	11	6.02	0.0181	25.6	7.86E-06	3.47	0.069			****		*	***
369	322	Primary somatosensory area, upper limb	9.66 ± 2.71	14	37.98 ± 12.85	14	13.75 ± 3.06	11	65.58 ± 12.62	11	2.85	0.0982	17.2	0.000143	1.57	0.217			***			**
361	322	Primary somatosensory area, trunk	14.14 ± 3.24	14	45.63 ± 15.40	13	17.15 ± 2.49	11	71.97 ± 14.77	11	1.8	0.187	15.5	0.000287	1.17	0.286			**			**
182305689	322	Primary somatosensory area, unassigned	14.46 ± 4.12	14	45.79 ± 22.24	12	17.82 ± 1.97	11	67.34 ± 15.21	11	0.817	0.371	9.02	0.00439	0.459	0.502			*			
378	453	Supplemental somatosensory area	9.47 ± 2.42	12	32.43 ± 8.57	16	12.91 ± 1.91	10	59.62 ± 10.69	11	4.65	0.0363	18.3	0.0000943	2.33	0.134			***			**
1057	315	Gustatory areas	7.49 ± 2.30	15	24.01 ± 5.81	14	5.31 ± 1.04	11	53.26 ± 9.53	12	5.94	0.0186	30.9	1.18E-06	7.84	0.00733			****		**	****
677	315	Visceral area	4.79 ± 0.99	14	20.61 ± 6.73	14	6.58 ± 1.11	10	38.16 ± 9.44	12	2.83	0.0994	15.2	0.000314	1.73	0.195			**			**
247	315	Auditory areas	9.40 ± 1.98	14	20.44 ± 5.19	16	11.01 ± 1.91	11	39.70 ± 7.94	11	4.79	0.0336	14.2	0.000454	3.27	0.077			***		*	**
1011	247	Dorsal auditory area	16.71 ± 4.34	12	32.89 ± 9.09	16	17.00 ± 3.10	11	62.03 ± 13.74	10	3.07	0.0865	10.1	0.00268	2.75	0.104			**			**
1002	247	Primary auditory area	16.72 ± 2.80	13	35.50 ± 9.00	12	18.33 ± 3.79	11	56.58 ± 20.14	9	1.2	0.279	7.88	0.00761	1	0.322			*			
1027	247	Posterior auditory area	14.02 ± 4.30	6	30.85 ± 16.99	7	13.43 ± 4.18	10	95.27 ± 21.89	6	5.31	0.0298	13.8	0.00103	6.4	0.018			**		*	***
1018	247	Ventral auditory area	11.28 ± 3.13	14	28.60 ± 7.11	16	15.39 ± 2.52	11	50.72 ± 13.45	10	3.22	0.079	11.2	0.00162	1.52	0.224			**			*
669	315	Visual areas	15.86 ± 3.60	14	19.17 ± 5.72	16	13.57 ± 3.11	11	44.94 ± 8.20	11	4.8	0.0333	7.33	0.00938	6.41	0.0146			**		**	**
402	669	Anterolateral visual area	23.40 ± 8.47	7	37.45 ± 13.13	8	23.00 ± 7.31	9	87.20 ± 17.94	6	3.85	0.0604	9.4	0.00501	4.58	0.0419			**		*	**
394	669	Anteromedial visual area	16.65 ± 5.36	12	30.98 ± 8.55	15	17.60 ± 2.26	11	65.10 ± 10.90	11	5.6	0.0224	13.4	0.000651	4.55	0.0383			***		*	***
385	669	Primary visual area	34.16 ± 8.77	11	47.47 ± 14.41	10	25.30 ± 8.76	9	88.51 ± 17.72	7	1.24	0.273	7.73	0.00889	4.03	0.053			*			*
533	669	posteromedial visual area	21.31 ± 6.70	10	47.17 ± 14.83	8	15.39 ± 4.15	10	60.61 ± 11.41	7	0.0642	0.802	14	0.000745	1.06	0.31			*			*
31	315	Anterior cingulate area	9.34 ± 2.83	15	20.34 ± 3.87	16	11.85 ± 2.07	11	41.89 ± 5.69	12	10.1	0.00251	24.7	8.16E-06	5.96	0.0182			****		**	****
39	31	Anterior cingulate area, dorsal part	15.65 ± 4.83	13	32.65 ± 7.35	15	19.02 ± 3.47	11	68.64 ± 10.38	12	8.24	0.00611	19.4	0.0000619	5.16	0.0278			****		**	***
48	31	Anterior cingulate area, ventral part	14.80 ± 4.61	15	30.02 ± 5.04	15	17.81 ± 3.18	11	61.48 ± 8.25	12	9.91	0.00279	25.5	6.62E-06	6.48	0.0141			****		**	****
972	315	Prelimbic area	6.52 ± 2.51	10	33.54 ± 11.10	10	23.03 ± 8.75	6	49.99 ± 12.92	8	2.87	0.101	8.64	0.00627	0.00000984	0.998			*			
44	315	Infralimbic area	16.10 ± 10.92	9	31.77 ± 13.21	10	18.90 ± 13.99	4	44.68 ± 12.26	8	0.452	0.507	2.45	0.129	0.134	0.717						
714	315	Orbital area	5.20 ± 1.30	6	16.86 ± 7.38	7	14.23 ± 9.29	4	24.32 ± 6.21	9	1.47	0.238	3.3	0.0828	0.0132	0.91						
723	714	Orbital area, lateral part	8.38 ± 1.86	6	25.70 ± 11.03	7	21.35 ± 13.93	4	36.47 ± 9.31	9	1.36	0.256	3.25	0.0852	0.0115	0.915						
95	315	Agranular insular area	4.11 ± 0.80	14	11.83 ± 3.38	16	4.27 ± 0.61	10	23.24 ± 3.91	12	4.82	0.033	19.8	0.0000507	3.89	0.0542			****		*	***
104	95	Agranular insular area, dorsal part	6.94 ± 1.59	11	20.72 ± 5.96	15	6.04 ± 0.97	10	38.58 ± 7.27	12	3.27	0.0776	17.2	0.00015	3.07	0.0868			***			***
111	95	Agranular insular area, posterior part	5.02 ± 1.12	13	13.42 ± 4.28	13	5.43 ± 1.20	10	30.83 ± 5.64	12	6.16	0.0169	19.6	0.0000623	5.11	0.0288			****		**	***
119	95	Agranular insular area, ventral part	9.07 ± 1.89	14	22.11 ± 5.87	15	7.12 ± 1.78	10	53.62 ± 14.97	12	3.93	0.0533	12.3	0.00102	4.33	0.0428			**		*	**
254	315	Retrosplenial area	8.07 ± 2.09	14	18.72 ± 4.35	16	8.69 ± 1.53	11	33.62 ± 6.24	11	3.91	0.0538	17.1	0.000144	3.14	0.0829			***			***
894	254	Retrosplenial area, lateral agranular part	14.96 ± 3.04	14	34.34 ± 9.33	16	16.52 ± 2.28	11	61.34 ± 10.97	11	3.59	0.0643	15.1	0.000312	2.7	0.107			***			**
879	254	Retrosplenial area, dorsal part	13.43 ± 3.44	15	33.68 ± 8.98	16	17.39 ± 3.20	10	69.85 ± 11.82	11	6.78	0.0123	18.2	0.0000919	4.08	0.049			****		*	***
886	254	Retrosplenial area, ventral part	11.35 ± 2.91	15	23.47 ± 5.05	16	10.53 ± 2.29	11	40.57 ± 7.16	12	3.12	0.0836	17.3	0.000125	3.47	0.0685			***			***
22	315	Posterior parietal association areas	11.53 ± 2.78	15	27.38 ± 7.29	16	14.09 ± 2.28	11	48.52 ± 7.76	11	4.2	0.0459	16.5	0.000179	2.52	0.119			***			**
312782546	22	Anterior area	14.76 ± 3.79	15	39.69 ± 11.04	16	17.06 ± 3.32	11	65.65 ± 11.15	11	2.73	0.105	16.4	0.000181	1.87	0.177			***			**
417	22	Rostrolateral visual area	21.82 ± 5.89	14	47.23 ± 11.78	16	24.51 ± 5.11	11	85.05 ± 13.90	11	4.11	0.0481	15.4	0.000281	2.95	0.0926			***			**
541	315	Temporal association areas	8.46 ± 2.76	13	20.62 ± 6.01	13	8.58 ± 1.88	11	44.76 ± 12.66	9	3.31	0.076	12.4	0.00104	3.63	0.0635			**			**
922	315	Perirhinal area	3.17 ± 1.00	14	8.07 ± 2.25	14	4.22 ± 1.46	11	26.00 ± 4.61	9	14.5	0.000434	24.1	0.0000128	12.7	0.000897			****		****	****
895	315	Ectorhinal area	7.15 ± 1.98	14	15.48 ± 4.02	14	6.91 ± 2.11	11	36.78 ± 8.29	9	5.52	0.0234	16.3	0.000213	6.52	0.0142			****		**	***
698	695	Olfactory areas	7.98 ± 2.04	14	17.33 ± 5.03	15	7.27 ± 1.29	12	38.37 ± 7.38	12	5.09	0.0285	17.4	0.000122	5.64	0.0215			***		*	***
159	698	Anterior olfactory nucleus	14.72 ± 4.84	8	22.95 ± 7.05	5	25.04 ± 2.56	6	92.07 ± 21.77	7	8.69	0.00744	12.3	0.00196	5.35	0.0305			***		**	**
589	698	Taenia tecta	8.64 ± 1.99	12	24.39 ± 9.70	10	7.23 ± 2.12	8	25.80 ± 4.75	10	0.0000221	0.996	9.25	0.00438	0.0622	0.804						
597	589	Taenia tecta, dorsal part	17.39 ± 3.87	12	54.08 ± 20.90	10	14.53 ± 4.23	8	51.25 ± 9.82	10	0.0627	0.804	10.4	0.00268	0.00000158	0.999						
814	698	Dorsal peduncular area	18.58 ± 6.56	14	49.87 ± 17.89	9	20.14 ± 4.32	8	64.39 ± 11.32	10	0.51	0.48	12.8	0.000997	0.351	0.557			*			*
961	698	Piriform area	21.15 ± 5.31	10	65.41 ± 18.77	10	21.92 ± 3.36	8	94.77 ± 17.95	12	1.26	0.269	16.5	0.000248	0.931	0.341			**			**
619	698	Nucleus of the lateral olfactory tract	15.24 ± 5.00	13	28.54 ± 11.13	6	12.29 ± 3.44	10	30.33 ± 8.99	6	0.0413	0.84	5.28	0.0285	0.123	0.728						
260	619	Nucleus of the lateral olfactory tract, molecular layer	51.39 ± 23.28	13	44.57 ± 23.43	6	20.90 ± 7.25	9	41.71 ± 21.73	6	0.884	0.355	0.035	0.853	0.369	0.548						
268	619	Nucleus of the lateral olfactory tract, pyramidal layer	27.31 ± 10.44	13	78.51 ± 32.74	6	25.07 ± 8.84	10	73.88 ± 24.95	6	0.0338	0.855	8.12	0.00772	0.00459	0.946						
1139	619	Nucleus of the lateral olfactory tract, layer 3	19.95 ± 6.97	13	40.97 ± 20.19	6	19.55 ± 7.76	10	57.88 ± 24.48	6	0.195	0.662	4.97	0.0331	0.421	0.521						
631	698	Cortical amygdalar area	11.47 ± 2.67	14	15.75 ± 3.61	11	8.81 ± 1.51	10	37.68 ± 11.97	12	2.07	0.157	6.55	0.0141	3.44	0.0704			*			*
639	631	Cortical amygdalar area, anterior part	49.04 ± 10.48	15	75.23 ± 15.14	10	48.66 ± 8.13	10	112.09 ± 23.51	12	1.21	0.278	8.85	0.00479	1.39	0.245			*			*
647	631	Cortical amygdalar area, posterior part	7.52 ± 2.84	13	11.28 ± 3.23	12	4.93 ± 1.70	10	37.48 ± 11.79	9	4.14	0.0487	8.83	0.005	6.92	0.0121			**		*	**
655	647	Cortical amygdalar area, posterior part, lateral zone	15.03 ± 5.08	13	19.09 ± 5.67	12	10.28 ± 4.09	10	74.75 ± 21.75	9	5.69	0.0219	9	0.00463	8.96	0.00471			***		**	***
663	647	Cortical amygdalar area, posterior part, medial zone	17.98 ± 8.08	11	26.92 ± 9.35	12	10.40 ± 3.13	9	65.03 ± 27.72	8	1.25	0.271	4.39	0.0433	2.93	0.0955						*
788	698	Piriform-amygdalar area	13.50 ± 2.82	15	35.75 ± 12.88	11	15.95 ± 2.13	10	90.37 ± 15.91	11	7.71	0.00812	24.7	0.0000114	6.97	0.0115			****		**	****
1089	695	Hippocampal formation	1.21 ± 0.27	15	7.66 ± 2.32	16	1.81 ± 0.39	12	13.40 ± 3.20	12	2.65	0.11	19.5	0.0000526	1.69	0.199			***			**
1080	1089	Hippocampal region	1.53 ± 0.34	15	10.13 ± 3.15	16	2.27 ± 0.48	12	17.46 ± 4.33	12	2.33	0.133	18.6	0.0000749	1.52	0.223			***			**
375	1080	Ammon's horn	1.67 ± 0.39	15	7.60 ± 2.04	16	2.43 ± 0.45	12	15.87 ± 4.25	12	4.18	0.0461	17	0.000137	2.82	0.0991			***			**
382	375	Field CA1	2.50 ± 0.55	15	10.49 ± 2.91	16	3.79 ± 0.83	12	31.40 ± 10.41	12	5.28	0.0257	11.4	0.00141	4.02	0.0502			***		*	**
423	375	Field CA2	1.17 ± 0.59	15	11.48 ± 4.05	16	2.39 ± 1.18	12	25.80 ± 6.74	12	4.19	0.0458	17.4	0.000117	2.9	0.0944			***			***
463	375	Field CA3	4.80 ± 1.33	14	21.51 ± 6.03	16	6.21 ± 1.04	12	35.27 ± 9.84	12	1.83	0.182	14.8	0.00034	1.15	0.288			**			**
726	1080	Dentate gyrus	4.04 ± 0.87	15	36.43 ± 12.86	16	6.23 ± 1.55	12	55.24 ± 12.89	12	1.25	0.268	17.6	0.000109	0.767	0.385			**			**
10703	726	Dentate gyrus, molecular layer	5.73 ± 1.19	15	42.80 ± 13.80	16	9.46 ± 2.88	12	66.77 ± 14.13	12	1.84	0.181	19.9	0.0000449	0.961	0.332	*		***			**
10704	726	Dentate gyrus, polymorph layer	8.36 ± 2.79	15	120.11 ± 50.37	16	23.96 ± 9.26	12	157.82 ± 43.21	12	0.571	0.453	11.8	0.00118	0.097	0.757			*			
632	726	Dentate gyrus, granule cell layer	13.80 ± 3.95	15	131.62 ± 48.34	16	16.46 ± 3.96	12	193.94 ± 48.17	12	0.856	0.359	16.5	0.000167	0.703	0.406			**			**
982	1080	Fasciola cinerea	7.75 ± 4.41	13	27.78 ± 10.36	13	7.65 ± 5.56	12	28.14 ± 12.89	10	0.000212	0.988	5.12	0.0284	0.000627	0.98						
19	1080	Induseum griseum	0.46 ± 0.46	15	14.41 ± 8.49	13	7.99 ± 4.24	12	50.33 ± 11.89	12	8.65	0.00501	14.9	0.000333	3.86	0.0554			****		**	**
822	1089	Retrohippocampal region	2.34 ± 0.69	14	5.58 ± 2.14	14	2.92 ± 1.07	12	14.98 ± 3.14	9	6.44	0.0147	13.6	0.000608	5.82	0.02			***		**	***
909	822	Entorhinal area	2.66 ± 0.88	14	5.33 ± 1.68	13	2.10 ± 0.77	11	18.22 ± 4.64	9	7.25	0.0101	15.4	0.00031	10.1	0.00272			****		***	****
918	909	Entorhinal area, lateral part	4.11 ± 1.36	14	8.27 ± 2.61	13	3.24 ± 1.18	11	28.09 ± 7.15	9	7.16	0.0105	15.4	0.000311	10	0.00282			****		***	****

502	822	Subiculum	7.24 ± 2.88	12	21.06 ± 9.08	11	20.49 ± 10.80	10	31.91 ± 6.98	7	2.3	0.138	2.27	0.141	0.0218	0.883					
484682470	822	Prosubiculum	1.51 ± 1.14	10	19.98 ± 8.23	10	22.49 ± 6.78	10	53.19 ± 13.12	7	12	0.00148	8.09	0.0076	0.634	0.432		***		*	
703	688	Cortical subplate	5.74 ± 1.15	15	12.77 ± 3.41	16	6.13 ± 1.12	12	29.73 ± 5.83	12	6.91	0.0113	18.1	0.0000894	6.12	0.0167		****		**	***
583	703	Claustrum	18.66 ± 5.42	15	41.93 ± 14.47	15	31.45 ± 8.47	12	106.07 ± 15.90	12	10.6	0.00202	15.4	0.000263	4.73	0.0344		****		**	***
942	703	Endopiriform nucleus	6.55 ± 1.50	13	16.32 ± 4.66	14	7.14 ± 1.39	12	28.77 ± 5.41	12	3.11	0.0845	16.5	0.000183	2.49	0.121		***			**
952	942	Endopiriform nucleus, dorsal part	13.00 ± 3.30	13	36.72 ± 10.53	15	12.48 ± 2.04	12	55.72 ± 9.41	12	1.52	0.223	17.6	0.000117	1.58	0.214		**			**
966	942	Endopiriform nucleus, ventral part	13.07 ± 3.56	13	19.91 ± 5.96	15	17.62 ± 4.84	12	60.30 ± 13.57	12	8.95	0.00437	8.64	0.00504	5.39	0.0245		***		**	**
131	703	Lateral amygdalar nucleus	11.85 ± 3.78	14	33.91 ± 9.13	15	11.11 ± 2.27	12	69.13 ± 15.59	12	3.73	0.0591	17.8	0.000106	3.94	0.0529		***		*	***
295	703	Basolateral amygdalar nucleus	7.80 ± 1.80	15	19.23 ± 5.51	14	9.71 ± 2.01	9	44.45 ± 9.72	12	6.05	0.0177	15.1	0.000322	3.96	0.0525		***		*	**
303	295	Basolateral amygdalar nucleus, anterior part	23.11 ± 5.94	15	41.34 ± 10.69	13	31.69 ± 6.81	9	111.23 ± 25.99	12	7.56	0.00857	11	0.00178	4.25	0.045		***		**	**
311	295	Basolateral amygdalar nucleus, posterior part	9.43 ± 3.52	14	27.76 ± 10.45	13	8.08 ± 2.77	9	83.37 ± 22.15	9	5.72	0.0214	14.5	0.000468	6.51	0.0146		***		**	***
451	295	Basolateral amygdalar nucleus, ventral part	10.31 ± 3.25	14	14.17 ± 4.06	12	10.49 ± 2.65	9	64.97 ± 12.25	10	16	0.000257	18.4	0.000106	15.9	0.000274		****		****	****
319	703	Basomedial amygdalar nucleus	10.85 ± 2.29	14	20.10 ± 4.75	14	10.71 ± 3.12	9	51.90 ± 11.72	12	6.61	0.0135	13.2	0.000709	5.7	0.0213		***		**	**
327	319	Basomedial amygdalar nucleus, anterior part	29.35 ± 5.44	14	55.53 ± 13.90	13	28.59 ± 7.83	9	119.13 ± 24.17	12	4.94	0.0314	14.6	0.00041	4.54	0.0387		***		*	**
334	319	Basomedial amygdalar nucleus, posterior part	12.33 ± 5.17	13	20.80 ± 8.41	12	13.47 ± 4.90	9	68.79 ± 18.46	9	5.99	0.019	8.6	0.00559	5.63	0.0227		**		**	**
780	703	Posterior amygdalar nucleus	11.44 ± 3.52	12	30.84 ± 10.69	12	9.94 ± 2.57	11	73.68 ± 28.57	8	2.27	0.14	9.16	0.00436	3.17	0.0827		**			**
623	567	Cerebral nuclei	2.74 ± 0.56	15	5.84 ± 1.11	16	2.82 ± 0.40	12	8.47 ± 1.42	12	2.01	0.162	19	0.0000638	1.73	0.194		***			**
477	623	Striatum	3.90 ± 0.79	15	8.32 ± 1.57	16	3.98 ± 0.56	12	12.29 ± 2.05	12	2.21	0.143	19.7	0.0000483	1.98	0.165		***			**
485	477	Striatum dorsal region	2.65 ± 0.50	15	8.68 ± 2.08	16	3.28 ± 0.49	12	10.09 ± 1.79	12	0.468	0.497	18.4	0.0000814	0.068	0.795	*	**			*
672	485	Caudoputamen	5.68 ± 1.00	14	18.53 ± 4.27	15	6.55 ± 0.98	12	22.02 ± 3.37	11	0.54	0.466	22.5	0.0000194	0.196	0.66	*	**	*		**
493	477	Striatum ventral region	5.72 ± 1.23	15	7.32 ± 1.39	15	4.60 ± 0.70	12	14.77 ± 3.25	12	3.07	0.0859	9.08	0.00406	5.6	0.0218		**		*	**
56	493	Nucleus accumbens	9.77 ± 1.91	12	16.98 ± 3.92	13	9.70 ± 1.49	12	36.39 ± 8.05	10	4.9	0.0322	13.8	0.000581	5.28	0.0265		***		*	***
998	493	Fundus of striatum	14.54 ± 5.66	9	25.99 ± 10.97	6	14.37 ± 5.09	6	34.86 ± 9.76	5	0.224	0.64	3.93	0.0601	0.324	0.575					
754	493	Olfactory tubercle	17.84 ± 5.20	13	16.84 ± 2.66	12	8.86 ± 1.94	10	27.80 ± 7.05	11	0.0501	0.824	2.93	0.0945	4.34	0.0433					*
275	477	Lateral septal complex	12.94 ± 3.68	14	21.87 ± 5.28	13	13.45 ± 1.87	12	28.37 ± 5.05	12	0.668	0.418	7.86	0.00734	0.502	0.482					
242	275	Lateral septal nucleus	19.97 ± 5.55	14	33.33 ± 8.11	13	20.45 ± 2.67	12	44.13 ± 8.68	12	0.688	0.411	7.52	0.00862	0.595	0.444					
258	242	Lateral septal nucleus, rostral (rostroventral) part	35.85 ± 9.38	14	68.00 ± 16.64	12	38.69 ± 5.30	12	79.48 ± 18.22	12	0.28	0.599	7.67	0.00805	0.107	0.745					
266	242	Lateral septal nucleus, ventral part	68.77 ± 28.74	11	75.81 ± 26.88	7	63.34 ± 11.90	10	116.88 ± 24.77	10	0.38	0.542	1.93	0.174	0.896	0.351					
333	275	Septohippocampal nucleus	13.96 ± 6.89	14	17.06 ± 8.99	8	19.58 ± 12.76	10	6.21 ± 4.56	7	0.0076	0.931	0.208	0.651	0.754	0.391					
278	477	Striatum-like amygdalar nuclei	9.22 ± 2.09	15	16.64 ± 3.09	15	9.69 ± 2.11	12	35.13 ± 7.03	12	5.97	0.0181	16	0.000206	5.4	0.0242		***		**	***
23	278	Anterior amygdalar area	12.99 ± 4.25	13	18.16 ± 6.36	9	13.37 ± 4.13	9	32.16 ± 5.54	8	1.59	0.216	5.21	0.0286	1.76	0.193		*			
292	278	Bed nucleus of the accessory olfactory tract	45.25 ± 26.49	5	47.98 ± 16.00	9	32.95 ± 18.64	4	64.83 ± 21.73	4	0.0318	0.86	0.405	0.532	0.446	0.513					
536	278	Central amygdalar nucleus	6.43 ± 1.26	15	15.87 ± 3.50	15	9.12 ± 2.33	11	27.61 ± 5.56	12	4.51	0.0387	15.6	0.000246	1.72	0.196		***			**
544	536	Central amygdalar nucleus, capsular part	9.29 ± 2.31	14	40.44 ± 11.74	14	23.77 ± 8.71	11	74.55 ± 14.22	12	5.78	0.0202	15.3	0.000287	0.912	0.344		***			**
551	536	Central amygdalar nucleus, lateral part	13.69 ± 4.08	14	40.88 ± 7.38	15	14.10 ± 3.96	11	73.62 ± 17.83	12	3.14	0.083	18.5	0.000083	2.76	0.103		***			***
559	536	Central amygdalar nucleus, medial part	14.63 ± 2.87	15	21.14 ± 5.89	14	18.21 ± 4.40	11	42.00 ± 8.89	12	4.51	0.0388	6.47	0.0142	2.23	0.142		**			*
1105	278	Intercalated amygdalar nucleus	21.10 ± 5.77	15	36.40 ± 12.31	14	30.68 ± 8.46	12	90.37 ± 39.04	12	2.59	0.114	3.42	0.0704	1.29	0.261					
403	278	Medial amygdalar nucleus	29.77 ± 7.14	15	47.16 ± 9.39	15	27.44 ± 6.10	12	106.46 ± 19.59	12	6.38	0.0147	16	0.000212	7.47	0.00865		****		**	***
803	623	Pallidum	2.37 ± 0.54	15	3.96 ± 1.08	15	2.62 ± 0.57	11	5.47 ± 1.05	12	1.04	0.312	6.2	0.0162	0.524	0.473					
818	803	Pallidum, dorsal region	1.85 ± 0.48	15	2.67 ± 0.77	14	2.02 ± 0.56	11	4.63 ± 0.80	12	2.52	0.119	6.21	0.0161	1.81	0.185		*			
1022	818	Globus pallidus, external segment	4.07 ± 1.02	15	6.94 ± 1.94	14	5.64 ± 1.41	11	13.23 ± 2.57	12	4.79	0.0335	8.13	0.00639	1.71	0.197		**			*
1031	818	Globus pallidus, internal segment	3.73 ± 2.32	14	3.12 ± 1.18	13	1.47 ± 0.71	11	2.20 ± 1.09	12	1.02	0.317	0.00134	0.971	0.179	0.674					
835	803	Pallidum, ventral region	3.73 ± 0.90	15	5.28 ± 1.46	15	3.04 ± 0.84	12	9.73 ± 3.03	12	1.22	0.274	5.16	0.0274	2.28	0.138					
342	835	Substantia innominata	7.29 ± 1.90	15	10.69 ± 2.93	15	6.66 ± 1.77	11	21.87 ± 6.41	11	2.28	0.138	5.93	0.0187	2.86	0.0971		*			*
826	803	Pallidum, medial region	4.45 ± 1.87	14	7.85 ± 2.83	12	4.13 ± 1.32	11	5.12 ± 1.25	11	0.559	0.459	1.28	0.264	0.372	0.545					
904	826	Medial septal complex	6.67 ± 2.81	14	10.27 ± 4.10	12	5.84 ± 1.95	11	5.71 ± 1.39	11	0.843	0.364	0.392	0.535	0.424	0.518					
564	904	Medial septal nucleus	0.00 ± 0.00	9	9.07 ± 2.48	6	2.85 ± 1.46	8	22.38 ± 7.90	8	3.09	0.0904	12.8	0.00132	1.47	0.235		**			*
596	904	Diagonal band nucleus	14.21 ± 5.68	14	20.28 ± 8.27	12	12.99 ± 4.01	11	7.69 ± 2.65	11	1.35	0.252	0.0105	0.919	0.97	0.33					
343	8	Brain stem	3.25 ± 0.78	15	3.74 ± 0.76	16	4.27 ± 0.94	12	5.89 ± 0.80	12	3.74	0.0587	1.39	0.244	0.467	0.498					
1129	343	Interbrain	3.64 ± 0.87	15	4.11 ± 0.74	16	5.06 ± 1.10	12	7.31 ± 1.02	12	6.35	0.0149	1.75	0.192	0.937	0.338		*			
549	1129	Thalamus	2.84 ± 0.73	15	3.20 ± 0.60	16	4.49 ± 1.05	12	7.52 ± 1.26	12	11.2	0.00152	2.74	0.104	2.22	0.143		**		**	
864	549	Thalamus, sensory-motor cortex related	2.26 ± 0.51	15	2.96 ± 0.75	16	5.72 ± 1.42	12	9.68 ± 1.91	12	19.3	0.000057	3.13	0.0831	1.98	0.166		***		***	
637	864	Ventral group of the dorsal thalamus	2.48 ± 0.55	15	3.19 ± 0.72	16	6.52 ± 1.52	12	13.07 ± 2.64	12	24	0.0000101	4.92	0.031	4.17	0.0465		****		****	*
629	637	Ventral anterior-lateral complex of the thalamus	2.80 ± 0.81	15	2.11 ± 0.87	14	11.25 ± 2.71	12	30.56 ± 7.90	12	22.7	0.0000173	5.16	0.0275	6.78	0.0122		****		****	**
685	637	Ventral medial nucleus of the thalamus	6.40 ± 2.88	15	6.00 ± 1.37	15	9.16 ± 3.45	12	13.05 ± 3.01	12	3.28	0.0761	0.312	0.579	0.626	0.433					
709	637	Ventral posterior complex of the thalamus	3.80 ± 0.75	15	5.26 ± 1.23	16	10.82 ± 2.53	12	21.24 ± 4.27	12	24.6	0.0000081	5.05	0.029	3.7	0.0601		****		****	*
718	709	Ventral posterolateral nucleus of the thalamus	2.83 ± 0.57	15	5.78 ± 1.58	16	13.50 ± 3.51	12	28.98 ± 6.36	12	26.2	4.71E-06	6.1	0.0169	3.55	0.0652		****		****	*
725	709	Ventral posterolateral nucleus of the thalamus, parvicellular part	0.00 ± 0.00	11	3.93 ± 1.98	13	5.49 ± 2.80	8	31.11 ± 11.81	7	12.3	0.00128	6.12	0.0184	5.47	0.0252		***		**	**
733	709	Ventral posteromedial nucleus of the thalamus	12.83 ± 2.68	14	14.15 ± 3.52	16	26.58 ± 6.03	12	52.29 ± 11.45	12	17.5	0.000118	3.27	0.0765	3.75	0.0585		***		***	*
741	709	Ventral posteromedial nucleus of the thalamus, parvicellular part	1.81 ± 1.81	9	4.52 ± 2.25	12	7.90 ± 3.37	10	13.43 ± 4.18	9	6.38	0.0161	1.25	0.272	0.221	0.641					
563807435	637	Posterior triangular thalamic nucleus	11.86 ± 8.67	9	2.38 ± 1.74	10	12.81 ± 4.87	10	22.75 ± 8.19	7	2.65	0.113	0.0689	0.795	2.5	0.124					
406	864	Subparafascicular nucleus	12.59 ± 4.03	11	5.51 ± 2.41	14	28.21 ± 9.99	11	18.92 ± 3.57	8	6.2	0.0171	3.04	0.0887	0.0357	0.851			*		
414	406	Subparafascicular nucleus, magnocellular part	23.41 ± 9.43	6	20.15 ± 10.21	9	81.56 ± 37.59	7	69.18 ± 15.14	5	5.75	0.025	0.609	0.443	0.0416	0.84					
422	406	Subparafascicular nucleus, parvicellular part	21.07 ± 9.39	9	8.42 ± 4.36	10	35.70 ± 19.07	9	18.48 ± 9.30	7	1.09	0.304	1.79	0.191	0.0368	0.849					
1008	864	Geniculate group, dorsal thalamus	5.05 ± 1.82	14	5.70 ± 1.92	16	11.58 ± 3.38	12	14.85 ± 3.57	11	8.9	0.00445	0.275	0.602	0.247	0.622					
475	1008	Medial geniculate complex	1.34 ± 0.64	8	0.96 ± 0.64	9	4.29 ± 1.54	10	15.94 ± 4.58	7	16.2	0.000358	4.55	0.0413	7.9	0.00863		***		***	**
1072	475	Medial geniculate complex, dorsal part	0.00 ± 0.00	6	4.26 ± 4.26	8	10.30 ± 7.29	9	41.68 ± 13.32	6	9.57	0.00482	3.55	0.0713	3.18	0.0868		**		*	*
1079	475	Medial geniculate complex, ventral part	0.00 ± 0.00	6	4.51 ± 3.18	7	7.70 ± 3.49	9	36.96 ± 15.52	6	7.24	0.0128	4.33	0.0482	2.94	0.0994		*		*	*
1088	475	Medial geniculate complex, medial part	4.76 ± 2.02	8	0.00 ± 0.00	9	9.48 ± 3.92	10	27.83 ± 6.34	7	18.5	0.000168	1.77	0.194	10.1	0.00339		**		****	**
170	1008	Dorsal part of the lateral geniculate complex	8.40 ± 3.21	14	9.79 ± 3.20	16	21.62 ± 6.26	12	27.31 ± 6.97	11	10.1	0.00251	0.257	0.615	0.198	0.658		*			
496345664	170	Dorsal part of the lateral geniculate complex, shell	7.16 ± 4.58	13	23.59 ± 8.53	15	31.79 ± 11.54	12	35.23 ± 10.70	11	3.97	0.0521	1.12	0.296	0.512	0.478					
496345668	170	Dorsal part of the lateral geniculate complex, core	21.04 ± 8.53	14	18.07 ± 5.92	16	48.53 ± 18.07	12	60.55 ± 15.11	11	8.68	0.00491	0.0197	0.889	0.397	0.532					
496345672	170	Dorsal part of the lateral geniculate complex, ipsilateral zone	16.65 ± 8.15	12	21.86 ± 8.26	15	42.81 ± 14.00	12	64.98 ± 24.98	11	5.87	0.0194	0.525	0.472	0.347	0.559					
856	549	Thalamus, polymodal association cortex related	4.33 ± 1.14	15	4.67 ± 0.83	16	5.83 ± 1.34	12	9.64 ± 1.52	12	7.5	0.00846	2.33	0.133	2.14	0.15		*		*	
138	856	Lateral group of the dorsal thalamus	1.91 ± 0.44	14	2.70 ± 0.72	16	6.32 ± 1.77	12	15.60 ± 2.89	12	30.7	1.11E-06	7.39	0.00897	7.18	0.00995		****		****	**
218	138	Lateral posterior nucleus of the thalamus	4.77 ± 1.37	14	5.76 ± 1.77	16	12.85 ± 3.02	12	22.53 ± 4.21	12	22.6	0.0000174	2.87	0.0965	2.7	0.106		***		***	
1020	138	Posterior complex of the thalamus	2.33 ± 0.69	14	5.69 ± 2.87	16	13.38 ± 4.98	12	40.32 ± 8.78	12	23.1	0.0000146	7.45	0.00874	5.96	0.0182		****		****	**
1029	138	Posterior limiting nucleus of the thalamus	20.44 ± 8.29	10	7.34 ± 3.74	10	12.48 ± 5.15	10	14.38 ± 5.68	10	0.0225	0.882	0.993	0.326	1.55	0.222					
325	138	Suprageniculate nucleus	7.16 ± 4.64	6	0.00 ± 0.00	7	0.84 ± 0.84	9	27.18 ± 15.73	6	1.83	0.188	1.73	0.2	5.65	0.0258					
560581551	138	Ethmoid nucleus of the thalamus	5.04 ± 2.59	13	4.60 ± 1.93	16	8.95 ± 3.43	12	11.13 ± 4.30	12	3.02	0.0887	0.0256	0.874	0.187	0.668					
239	856	Anterior group of the dorsal thalamus	4.14 ± 1.26	15	4.15 ± 1.08	14	4.71 ± 1.02	12	8.40 ± 1.27	12	4.07	0.0492	2.14	0.15	2.42	0.126					
255	239	Anteroventral nucleus of thalamus	11.55 ± 3.89	13	8.49 ± 4.41	10	7.41 ± 2.22	12	10.15 ± 3.53	10	0.189	0.666	0.00446	0.947	0.651	0.424					
127	239	Anteromedial nucleus	3.31 ± 1.75	13	2.19 ± 0.82	9	3.75 ± 1.11	12	2.57 ± 1.34	10	0.0899	0.766	0.648	0.426	0.000504	0.982					
1096	127	Anteromedial nucleus, dorsal part	6.83 ± 4.66	13	4.41 ± 2.37	9	4.20 ± 1.70	12	5.42 ± 3.10	10	0.0966	0.758	0.0332	0.856	0.283	0.598					
1104	127	Anteromedial nucleus, ventral part	6.14 ± 2.36	13	4.16 ± 2.65	9	12.98 ± 4.40	12	4.68 ± 2.41	10	1.66	0.204	2.41	0.128	0.967	0.331					

64	239	Anterodorsal nucleus	15.70 ± 8.13	12	11.60 ± 2.75	10	5.27 ± 2.17	11	11.75 ± 5.85	10	0.977	0.329	0.0296	0.864	0.898	0.349			
1120	239	Interanteromedial nucleus of the thalamus	29.66 ± 14.46	12	18.62 ± 5.17	9	30.89 ± 24.71	10	16.69 ± 9.48	9	0.000149	0.99	0.618	0.437	0.00975	0.922			
1113	239	Interanterodorsal nucleus of the thalamus	37.85 ± 12.13	12	12.36 ± 8.18	9	23.33 ± 6.38	12	34.43 ± 14.13	10	0.0232	0.88	0.38	0.541	2.9	0.0963			
155	239	Lateral dorsal nucleus of thalamus	3.97 ± 1.01	15	10.51 ± 4.64	14	11.37 ± 3.04	12	23.01 ± 3.56	12	9	0.00425	7.52	0.00849	0.599	0.443	***		
444	856	Medial group of the dorsal thalamus	5.91 ± 1.46	15	6.57 ± 1.47	15	8.56 ± 2.26	12	12.70 ± 2.41	12	5.48	0.0233	1.4	0.242	0.862	0.358			
59	444	Intermediodorsal nucleus of the thalamus	24.04 ± 6.07	14	31.81 ± 6.36	15	40.41 ± 8.20	12	31.86 ± 7.55	12	1.33	0.255	0.00118	0.973	1.36	0.25			
362	444	Mediodorsal nucleus of thalamus	10.70 ± 2.83	15	8.92 ± 2.11	15	14.33 ± 4.81	12	21.80 ± 4.63	12	5.35	0.0248	0.431	0.514	1.68	0.201			
366	444	Submedial nucleus of the thalamus	2.94 ± 0.79	14	6.51 ± 1.53	14	17.93 ± 5.81	12	37.27 ± 8.24	11	24.5	0.0000101	4.98	0.0304	2.95	0.0924	****	***	*
1077	444	Perireunensis nucleus	30.30 ± 9.24	14	14.38 ± 4.29	14	15.03 ± 5.20	12	28.50 ± 5.11	12	0.0147	0.904	0.157	0.694	4.91	0.0315			
571	856	Midline group of the dorsal thalamus	20.60 ± 5.93	15	19.61 ± 5.68	16	18.55 ± 3.12	12	28.07 ± 4.26	12	0.395	0.533	0.469	0.497	1.01	0.319			
149	571	Paraventricular nucleus of the thalamus	80.87 ± 18.53	15	72.73 ± 21.61	16	81.97 ± 14.86	12	98.93 ± 16.90	12	0.525	0.472	0.0188	0.892	0.433	0.514			
15	571	Parataenial nucleus	64.81 ± 26.99	10	35.41 ± 22.71	6	48.57 ± 17.58	7	24.53 ± 18.02	6	0.335	0.568	1.34	0.257	0.012	0.914			
181	571	Nucleus of reuniens	14.67 ± 6.28	15	20.38 ± 5.75	14	12.19 ± 3.48	12	29.37 ± 7.72	12	0.271	0.605	3.28	0.0764	0.89	0.35			
560581559	571	Xiphoid thalamic nucleus	27.49 ± 7.45	15	31.08 ± 7.51	14	22.39 ± 6.77	12	47.78 ± 12.73	12	0.413	0.523	2.42	0.126	1.55	0.219			
51	856	Intralaminar nuclei of the dorsal thalamus	7.28 ± 1.84	15	6.51 ± 1.45	16	8.27 ± 2.06	12	14.10 ± 2.70	12	4.73	0.0343	1.07	0.305	2.75	0.104		*	
189	51	Rhomboid nucleus	17.21 ± 5.97	15	21.71 ± 8.29	14	22.79 ± 8.31	12	40.50 ± 13.81	12	1.73	0.195	1.37	0.248	0.517	0.476			
599	51	Central medial nucleus of the thalamus	32.59 ± 7.54	15	27.98 ± 6.71	15	32.01 ± 10.18	12	41.33 ± 10.64	12	0.544	0.464	0.0336	0.855	0.647	0.425			
907	51	Paracentral nucleus	4.62 ± 2.02	14	8.84 ± 4.18	14	7.58 ± 2.96	12	32.53 ± 7.10	12	9.4	0.00356	10.1	0.00257	5.68	0.0211	***	**	**
575	51	Central lateral nucleus of the thalamus	10.24 ± 4.95	14	14.48 ± 4.75	15	8.33 ± 1.53	12	21.85 ± 4.31	12	0.422	0.519	3.83	0.0561	1.16	0.287			
930	51	Parafascicular nucleus	6.97 ± 3.47	11	5.36 ± 2.22	11	19.65 ± 6.76	10	15.05 ± 6.17	7	5.65	0.0231	0.674	0.417	0.0976	0.757			
560581563	51	Posterior intralaminar thalamic nucleus	14.14 ± 8.45	4	3.52 ± 3.52	5	4.21 ± 2.72	6	18.35 ± 7.97	6	0.284	0.601	0.324	0.577	4.12	0.0584			
262	856	Reticular nucleus of the thalamus	2.46 ± 0.56	15	7.98 ± 1.89	15	10.40 ± 3.75	12	24.72 ± 5.19	12	16.3	0.000186	9.64	0.00313	2.07	0.157	****	**	*
1014	856	Geniculate group, ventral thalamus	22.45 ± 9.08	13	18.61 ± 4.93	16	29.20 ± 7.39	11	21.30 ± 6.72	11	0.433	0.514	0.723	0.399	0.0818	0.776			
27	1014	Intergeniculate leaflet of the lateral geniculate complex	60.84 ± 24.16	9	43.19 ± 15.11	13	109.07 ± 30.72	10	61.17 ± 23.29	8	1.94	0.172	2.44	0.127	0.414	0.524			
563807439	1014	Intermediate geniculate nucleus	20.52 ± 12.00	4	22.77 ± 18.09	7	90.68 ± 58.48	7	23.25 ± 7.87	4	0.715	0.409	1.1	0.307	0.696	0.415			
178	1014	Ventral part of the lateral geniculate complex	47.75 ± 20.66	13	40.05 ± 11.19	16	57.04 ± 15.39	12	45.07 ± 14.45	11	0.205	0.652	0.423	0.519	0.0185	0.892			
321	1014	Subgeniculate nucleus	4.48 ± 4.48	7	13.17 ± 7.91	11	40.00 ± 40.00	5	13.17 ± 8.48	6	0.942	0.341	0.157	0.696	1.25	0.274			
958	856	Epithalamus	8.47 ± 1.63	15	14.20 ± 3.32	16	7.78 ± 3.47	12	14.43 ± 2.40	12	0.0062	0.938	4.75	0.0339	0.0257	0.873			
483	958	Medial habenula	21.01 ± 5.46	15	36.92 ± 9.78	16	13.66 ± 4.30	12	41.79 ± 8.02	12	0.0227	0.881	7.89	0.00702	0.642	0.427			
186	958	Lateral habenula	11.89 ± 3.02	15	17.42 ± 5.83	15	17.73 ± 11.75	12	15.47 ± 4.94	12	0.083	0.775	0.0949	0.759	0.332	0.567			
1097	1129	Hypothalamus	9.67 ± 2.14	14	10.14 ± 2.00	15	11.38 ± 2.35	12	13.66 ± 1.86	12	1.56	0.218	0.35	0.557	0.185	0.669			
157	1097	Periventricular zone	16.58 ± 5.26	14	21.85 ± 6.78	15	23.54 ± 5.66	12	31.46 ± 5.42	12	1.95	0.169	1.14	0.29	0.0497	0.824			
390	157	Supraoptic nucleus	83.65 ± 36.42	10	72.34 ± 21.83	7	79.37 ± 36.24	10	17.92 ± 9.22	8	0.683	0.415	1.48	0.233	0.635	0.432			
38	157	Paraventricular hypothalamic nucleus	61.56 ± 22.81	13	140.29 ± 32.59	9	73.92 ± 19.24	10	162.74 ± 30.03	10	0.423	0.519	10.7	0.00228	0.0374	0.848	*		
30	157	Periventricular hypothalamic nucleus, anterior part	9.16 ± 4.68	8	93.78 ± 54.91	6	58.19 ± 28.05	7	67.79 ± 21.76	8	0.204	0.656	2.63	0.117	1.66	0.209			
118	157	Periventricular hypothalamic nucleus, intermediate part	16.91 ± 8.68	13	24.28 ± 8.43	15	16.23 ± 6.61	12	37.83 ± 10.30	12	0.593	0.445	2.52	0.119	0.676	0.415			
223	157	Arcuate hypothalamic nucleus	30.70 ± 9.90	13	29.02 ± 11.45	14	31.36 ± 11.41	11	30.42 ± 8.68	11	0.00947	0.923	0.0166	0.898	0.00119	0.973			
141	1097	Periventricular region	13.47 ± 2.79	15	17.95 ± 3.13	16	23.72 ± 4.85	12	28.61 ± 4.24	12	7.97	0.00676	1.5	0.227	0.00306	0.956	*		
830	141	Dorsomedial nucleus of the hypothalamus	38.31 ± 9.00	12	39.45 ± 11.08	14	50.79 ± 11.53	11	69.68 ± 14.14	11	3.53	0.0669	0.543	0.465	0.591	0.446			
126	141	Periventricular hypothalamic nucleus, posterior part	8.07 ± 3.99	9	19.93 ± 7.10	10	32.22 ± 9.79	7	27.37 ± 11.16	8	3.52	0.0704	0.322	0.575	1.05	0.314			
347	141	Subparaventricular zone	18.00 ± 7.27	13	59.50 ± 18.50	9	51.13 ± 15.84	11	85.62 ± 22.13	10	3.6	0.0653	6.35	0.0159	0.0483	0.827	*		
286	141	Suprachiasmatic nucleus	37.05 ± 12.49	10	39.65 ± 12.95	6	132.14 ± 79.88	8	51.64 ± 37.11	7	1.72	0.2	0.518	0.478	0.803	0.378			
467	1097	Hypothalamic medial zone	16.01 ± 3.87	15	15.95 ± 3.57	16	16.12 ± 3.26	12	17.82 ± 2.60	12	0.0824	0.775	0.0393	0.844	0.0625	0.804			
88	467	Anterior hypothalamic nucleus	26.22 ± 5.25	15	42.47 ± 11.33	12	41.77 ± 14.50	12	41.18 ± 6.93	10	0.632	0.431	0.762	0.387	0.697	0.408			
331	467	Mammillary body	11.81 ± 4.85	9	7.57 ± 2.55	13	7.98 ± 2.58	11	9.47 ± 1.37	7	0.115	0.737	0.22	0.642	0.794	0.379			
210	331	Lateral mammillary nucleus	1.98 ± 1.98	6	23.78 ± 21.68	8	11.70 ± 6.28	9	10.12 ± 7.19	7	0.0287	0.867	0.57	0.457	0.828	0.371			
491	331	Medial mammillary nucleus	29.37 ± 22.66	7	12.32 ± 7.14	10	4.77 ± 1.92	8	10.85 ± 2.79	7	1.28	0.267	0.172	0.681	1.1	0.304			
732	491	Medial mammillary nucleus, median part	91.09 ± 69.77	7	11.61 ± 6.72	9	5.98 ± 4.30	7	15.38 ± 4.99	7	1.34	0.258	1.15	0.293	1.82	0.189			
606826647	491	Medial mammillary nucleus, lateral part	52.94 ± 45.72	7	28.16 ± 15.74	10	11.67 ± 3.63	8	13.29 ± 5.65	7	1.47	0.235	0.157	0.695	0.341	0.564			
606826651	491	Medial mammillary nucleus, medial part	10.02 ± 6.87	7	6.95 ± 6.12	10	4.93 ± 3.23	8	35.54 ± 11.13	6	2.91	0.0996	2.33	0.139	5.94	0.0217		*	*
525	331	Supramammillary nucleus	29.33 ± 10.20	8	23.01 ± 7.44	10	34.60 ± 12.25	10	41.62 ± 6.09	7	1.4	0.246	0.0264	0.872	0.455	0.505			
557	331	Tuberomammillary nucleus	8.77 ± 3.86	8	13.58 ± 4.28	12	15.27 ± 5.85	9	12.36 ± 4.08	6	0.312	0.58	0.0402	0.842	0.611	0.44			
1126	557	Tuberomammillary nucleus, dorsal part	22.93 ± 12.45	8	31.20 ± 10.68	8	64.51 ± 33.02	6	11.59 ± 7.33	5	0.489	0.491	0.927	0.346	2.86	0.104			
1	557	Tuberomammillary nucleus, ventral part	17.91 ± 8.58	7	23.32 ± 9.49	10	16.18 ± 7.19	9	30.73 ± 12.62	6	0.0814	0.778	0.955	0.337	0.228	0.637			
980	467	Dorsal premammillary nucleus	64.21 ± 26.94	7	33.02 ± 13.58	8	24.54 ± 7.59	7	34.63 ± 10.17	6	1.33	0.26	0.413	0.527	1.53	0.228			
1004	467	Ventral premammillary nucleus	28.38 ± 12.38	8	32.63 ± 10.52	11	22.40 ± 8.21	9	39.38 ± 12.17	7	0.00144	0.97	0.86	0.361	0.336	0.566			
63	467	Paraventricular hypothalamic nucleus, descending division	68.25 ± 16.55	13	40.81 ± 7.64	12	78.80 ± 19.75	9	63.70 ± 8.70	11	1.5	0.228	2.3	0.137	0.198	0.658			
693	467	Ventromedial hypothalamic nucleus	17.70 ± 3.93	14	23.87 ± 6.12	13	17.78 ± 7.02	12	45.46 ± 8.38	11	2.7	0.107	6.45	0.0146	2.88	0.0962	*		*
946	467	Posterior hypothalamic nucleus	57.59 ± 15.87	14	47.73 ± 12.78	15	66.80 ± 15.09	11	45.16 ± 8.84	9	0.0615	0.805	1.1	0.3	0.166	0.686			
290	1097	Hypothalamic lateral zone	8.60 ± 1.98	15	9.30 ± 2.01	16	9.94 ± 2.26	12	13.04 ± 1.91	12	1.53	0.222	0.693	0.409	0.338	0.563			
194	290	Lateral hypothalamic area	19.30 ± 4.49	15	24.86 ± 6.70	16	23.45 ± 5.60	10	23.41 ± 3.79	12	0.0436	0.836	0.352	0.556	0.25	0.619			
226	290	Lateral preoptic area	11.74 ± 5.56	6	5.27 ± 4.38	6	10.83 ± 3.78	5	17.19 ± 8.46	4	0.855	0.368	0.0554	0.817	1.35	0.261			
364	290	Parasubthalamic nucleus	31.29 ± 8.07	12	51.81 ± 12.95	13	40.23 ± 12.24	11	85.00 ± 15.14	8	2.72	0.107	5.72	0.0216	0.976	0.329	*		
576073704	290	Perifornical nucleus	24.78 ± 5.39	15	23.21 ± 6.17	14	35.65 ± 12.36	12	31.24 ± 7.87	11	1.39	0.244	0.128	0.722	0.0309	0.861			
173	290	Retrochiasmatic area	7.26 ± 2.87	13	1.70 ± 1.70	5	19.35 ± 12.72	9	11.59 ± 7.61	8	2.04	0.163	0.305	0.585	0.0181	0.894			
470	290	Subthalamic nucleus	5.86 ± 2.30	12	6.59 ± 3.03	13	11.35 ± 6.63	11	43.86 ± 23.55	9	4.17	0.0475	1.75	0.193	2.44	0.126			
614	290	Tuberal nucleus	17.77 ± 5.06	14	16.74 ± 3.76	14	18.02 ± 4.43	11	36.96 ± 7.86	11	3.67	0.0616	2.14	0.151	3.49	0.0681		*	
797	290	Zona incerta	14.20 ± 3.56	15	14.92 ± 4.15	16	17.70 ± 3.68	12	21.95 ± 3.63	12	1.87	0.178	0.322	0.573	0.208	0.65			
804	797	Fields of Forel	19.98 ± 9.38	10	18.41 ± 8.89	11	18.24 ± 8.34	9	24.85 ± 7.61	8	0.0676	0.796	0.0501	0.824	0.214	0.646			
10671	1097	Median eminence	27.35 ± 15.86	12	11.59 ± 5.45	14	10.28 ± 6.11	10	18.75 ± 8.98	10	0.2	0.657	0.246	0.622	1.41	0.241			
313	343	Midbrain	13.92 ± 3.61	13	10.76 ± 4.06	13	10.85 ± 3.13	12	11.33 ± 2.57	10	0.145	0.705	0.169	0.683	0.266	0.609			
339	313	Midbrain, sensory related	1.56 ± 1.05	11	16.73 ± 8.28	11	18.99 ± 15.77	10	28.05 ± 6.58	7	2.25	0.142	1.35	0.253	0.0962	0.758			
599626923	339	Subcommissural organ	0.00 ± 0.00	11	19.75 ± 19.75	9	32.00 ± 32.00	10	112.82 ± 61.17	7	3.7	0.0631	2.21	0.147	0.997	0.325			
323	313	Midbrain, motor related	12.80 ± 3.77	13	9.93 ± 3.72	14	11.31 ± 3.46	12	11.02 ± 2.26	10	0.00485	0.945	0.235	0.63	0.133	0.717			
381	323	Substantia nigra, reticular part	0.77 ± 0.77	5	5.13 ± 5.13	9	2.11 ± 0.91	9	3.42 ± 1.38	7	0.0104	0.92	0.718	0.405	0.208	0.652			
749	323	Ventral tegmental area	3.60 ± 3.60	4	1.14 ± 1.14	5	10.71 ± 4.11	8	2.74 ± 1.88	5	1.53	0.232	3.36	0.0835	0.588	0.453			
128	323	Midbrain reticular nucleus	11.21 ± 3.94	8	9.30 ± 3.98	10	12.88 ± 5.13	10	19.43 ± 3.14	6	1.52	0.227	0.0474	0.829	0.882	0.355			
795	323	Periaqueductal gray	37.03 ± 14.58	13	24.15 ± 7.42	14	24.91 ± 6.73	12	20.97 ± 4.74	10	0.647	0.426	0.757	0.389	0.212	0.648			
50	795	Precommissural nucleus	82.12 ± 31.21	10	54.36 ± 15.87	13	66.33 ± 14.97	12	42.45 ± 11.48	10	0.516	0.476	1.62	0.21	0.0101	0.92			
67	795	Interstitial nucleus of Cajal	13.14 ± 8.48	4	0.00 ± 0.00	4	2.69 ± 2.69	7	3.10 ± 3.10	6	0.868	0.364	1.31	0.268	2.65	0.122			
587	795	Nucleus of Darkschewitsch	12.42 ± 8.58	4	16.00 ± 16.00	4	3.36 ± 3.36	7	3.51 ± 3.51	6	2.02	0.174	0.0634	0.804	0.0514	0.823			
1100	323	Pretectal region	9.03 ± 2.68	11	15.24 ± 7.46	12	10.46 ± 3.16	11	10.98 ± 2.12	8	0.0617	0.805	0.604	0.442	0.333	0.567			
215	1100	Anterior pretectal nucleus	12.71 ± 3.87	11	25.42 ± 14.17	12	20.25 ± 6.15	11	21.81 ± 5.23	8	0.0635	0.802	0.66	0.421	0.358	0.553			
531	1100	Medial pretectal area	25.10 ± 10.64	6	21.98 ± 15.29	6	39.08 ± 39.08	5	43.43 ± 7.73	4	0.639	0.435	0.00172	0.967	0.029	0.867			
628	1100	Nucleus of the optic tract	15.98 ± 6.28	8	11.08 ± 5.65	8	17.33 ± 6.37	8	21.68 ± 7.47	7	0.822	0.373	0.00956	0.923	0.518	0.478			

634	1100	Nucleus of the posterior commissure	38.89 ± 15.69	9	57.23 ± 35.39	8	16.90 ± 8.36	8	22.61 ± 8.16	7	1.84	0.186	0.371	0.548	0.0937	0.762
706	1100	Olivary pretectal nucleus	16.68 ± 7.28	8	10.62 ± 4.76	8	13.39 ± 5.28	7	22.15 ± 8.52	7	0.389	0.538	0.0168	0.898	1.26	0.272
1061	1100	Posterior pretectal nucleus	33.81 ± 19.98	6	48.73 ± 28.74	7	42.69 ± 20.45	7	18.11 ± 8.25	6	0.253	0.62	0.0344	0.855	0.837	0.37
549009203	1100	Retroparafascicular nucleus	10.16 ± 10.16	6	1.40 ± 1.40	8	14.28 ± 8.01	6	14.69 ± 8.61	6	1.56	0.225	0.508	0.484	0.402	0.532
348	313	Midbrain, behavioral state related	1.05 ± 1.05	5	1.12 ± 0.61	7	7.60 ± 4.07	8	2.87 ± 1.54	5	2.21	0.152	1.35	0.258	0.716	0.407
374	348	Substantia nigra, compact part	2.09 ± 2.09	5	1.64 ± 1.64	5	12.38 ± 8.46	8	3.52 ± 3.52	5	1.02	0.325	0.833	0.373	0.421	0.524
