# Supplementary material for: Hyperactive neuronal networks facilitate tau spread in an Alzheimer’s disease mouse model
Source: bioRxiv. 2024 Dec 3:2024.12.01.625514. Preprint. [Version 2] doi: 10.1101/2024.12.01.625514 (PMC11642807; doi:10.1101/2024.12.01.625514)
Supplement: Supplementary file 4 [file media-4.pdf]

Region.ID	Parent ID	Region	WT.Sal	n	WT.PTZ	n	5X.Sal	n	5X.PTZ	n	Genotype F	Genotype p	Treatment F	Treatment p	Interaction F	Interaction p	p1	p2	p3	p4	p5	p6
8	997	Basic cell groups and regions	2.12 ± 0.36	15	5.21 ± 1.09	16	2.78 ± 0.44	12	9.31 ± 1.50	12	6.29	0.0154	23	0.0000146	3.22	0.0787			****		*	***
567	8	Cerebrum	2.38 ± 0.40	15	6.75 ± 1.52	16	2.92 ± 0.42	12	12.23 ± 2.08	12	5.44	0.0237	25	0.00000717	3.58	0.0643			****		*	****
688	567	Cerebral cortex	2.93 ± 0.49	15	8.58 ± 2.00	16	3.51 ± 0.55	12	15.87 ± 2.68	12	5.47	0.0233	25.4	0.00000619	3.87	0.0545			****		*	****
695	688	Cortical plate	3.52 ± 0.59	15	10.42 ± 2.45	16	4.15 ± 0.67	12	19.02 ± 3.20	12	5.13	0.0278	25.4	0.00000623	3.73	0.0589			****		*	****
315	695	Isocortex	4.95 ± 0.88	15	14.91 ± 3.41	16	6.65 ± 0.97	11	27.62 ± 5.00	12	5.71	0.0207	22.9	0.0000158	3.14	0.0825			****		*	***
500	315	Somatomotor areas	3.69 ± 0.69	15	17.85 ± 4.81	16	6.42 ± 0.95	11	33.58 ± 7.00	12	4.86	0.0321	21.5	0.0000255	2.28	0.137			****			***
985	500	Primary motor area	4.95 ± 0.85	15	27.45 ± 7.77	16	7.95 ± 1.28	11	56.97 ± 10.37	11	6.4	0.0147	26.9	0.00000401	4.16	0.0468	*		****		*	****
993	500	Secondary motor area	7.66 ± 1.62	13	27.83 ± 7.77	14	13.26 ± 2.13	11	54.46 ± 11.00	11	5.74	0.0208	18.8	0.0000808	2.39	0.129			****		*	**
453	315	Somatosensory areas	5.48 ± 1.07	15	19.32 ± 4.66	16	7.83 ± 1.04	11	34.68 ± 7.16	11	4.5	0.039	21.2	0.00003	2.38	0.129			****			***
322	453	Primary somatosensory area	7.20 ± 1.39	15	25.53 ± 6.13	16	10.42 ± 1.37	11	45.48 ± 8.94	11	4.68	0.0353	22.2	0.0000207	2.39	0.128			****			***
353	322	Primary somatosensory area, nose	12.72 ± 2.94	9	15.42 ± 8.08	8	12.32 ± 3.30	9	115.37 ± 76.99	10	1.3	0.263	1.83	0.186	1.34	0.256						
329	322	Primary somatosensory area, barrel field	14.96 ± 2.83	14	42.41 ± 9.72	16	18.73 ± 2.21	11	70.64 ± 14.03	11	3.51	0.0671	18.7	0.0000762	1.97	0.167			***			**
337	322	Primary somatosensory area, lower limb	12.63 ± 2.71	13	35.55 ± 11.17	11	16.21 ± 2.84	11	68.79 ± 14.96	11	3.78	0.0585	17.1	0.000163	2.62	0.113			***			**
345	322	Primary somatosensory area, mouth	6.53 ± 1.21	13	36.43 ± 11.13	13	9.95 ± 1.64	11	71.73 ± 14.97	10	4.47	0.0403	24.1	0.0000138	3.17	0.0822			****		*	***
369	322	Primary somatosensory area, upper limb	8.48 ± 2.17	14	35.45 ± 12.31	14	13.05 ± 2.34	11	62.32 ± 12.52	11	3.03	0.0885	16.8	0.000166	1.52	0.223			***			**
361	322	Primary somatosensory area, trunk	10.41 ± 2.03	13	41.51 ± 12.37	14	18.44 ± 3.96	11	72.25 ± 15.06	11	3.79	0.0579	16.7	0.000175	1.27	0.265			***			**
182305689	322	Primary somatosensory area, ussigned	14.27 ± 4.33	14	39.66 ± 14.25	12	18.93 ± 2.60	11	66.71 ± 16.11	11	2.1	0.154	11.7	0.00138	1.1	0.3			**			*
378	453	Supplemental somatosensory area	9.73 ± 1.87	14	32.44 ± 8.20	16	12.62 ± 1.78	11	67.50 ± 13.81	10	6.07	0.0174	21.3	0.0000304	4.37	0.0421			****		*	***
1057	315	Gustatory areas	7.49 ± 1.86	15	29.04 ± 8.91	14	4.95 ± 1.10	11	61.45 ± 12.76	12	3.81	0.0569	23.9	0.0000116	5.1	0.0285			****		*	****
677	315	Visceral area	5.66 ± 1.40	14	25.20 ± 7.86	14	6.18 ± 1.26	11	54.27 ± 10.63	10	5.1	0.0288	24.8	0.00000987	5.01	0.0302			****		*	****
247	315	Auditory areas	8.55 ± 1.86	14	22.16 ± 5.06	15	12.46 ± 2.16	11	49.35 ± 11.63	12	6.48	0.0142	14.7	0.000367	3.42	0.0707			***		*	**
1011	247	Dorsal auditory area	11.87 ± 3.46	12	38.01 ± 9.52	15	17.05 ± 2.61	11	77.59 ± 17.50	12	5.17	0.0276	15.7	0.000252	2.72	0.106			***		*	**
1002	247	Primary auditory area	14.94 ± 3.26	13	36.62 ± 7.95	14	22.03 ± 4.04	11	82.65 ± 22.99	11	5.4	0.0248	11.3	0.0016	2.83	0.0995			***		*	**
1027	247	Posterior auditory area	18.86 ± 4.26	7	39.66 ± 16.56	7	17.83 ± 7.22	8	86.33 ± 28.91	7	1.75	0.198	7.18	0.0128	2.04	0.165			*			*
1018	247	Ventral auditory area	11.47 ± 2.65	14	32.13 ± 7.77	15	16.43 ± 3.09	11	63.53 ± 15.22	12	4.72	0.0347	14.4	0.000418	2.36	0.131			***			**
669	315	Visual areas	15.15 ± 3.23	14	23.35 ± 6.16	15	17.20 ± 3.77	11	51.66 ± 10.33	10	5.99	0.0183	9.48	0.00349	4.58	0.0376			***		*	**
402	669	Anterolateral visual area	24.28 ± 6.23	8	43.41 ± 12.87	8	20.47 ± 9.24	8	103.15 ± 21.45	8	4.17	0.0506	13.8	0.000891	5.39	0.0278			**		*	**
394	669	Anteromedial visual area	15.82 ± 3.33	13	41.29 ± 10.53	14	24.33 ± 5.35	11	64.18 ± 13.72	10	3.02	0.0895	12.2	0.00112	0.639	0.428			**			*
385	669	Primary visual area	41.30 ± 7.94	11	48.38 ± 14.51	11	34.11 ± 9.21	9	87.50 ± 19.41	9	1.43	0.239	4.43	0.0423	3.02	0.0909						*
533	669	posteromedial visual area	16.93 ± 3.76	10	36.99 ± 11.74	10	22.56 ± 6.05	10	62.11 ± 13.03	9	2.67	0.111	9.92	0.00334	1.11	0.3			**			*
31	315	Anterior cingulate area	8.45 ± 1.34	15	20.36 ± 3.87	15	12.22 ± 2.01	11	38.11 ± 5.63	12	9.35	0.0036	26.4	0.00000482	3.82	0.0564			****		**	****
39	31	Anterior cingulate area, dorsal part	13.99 ± 2.40	15	34.05 ± 7.14	15	18.60 ± 3.57	11	58.46 ± 8.97	12	5.8	0.0199	22.7	0.000017	2.61	0.113			****		*	***
48	31	Anterior cingulate area, ventral part	11.41 ± 2.17	15	28.69 ± 4.83	15	19.41 ± 3.04	11	59.06 ± 8.76	12	14	0.000493	29.7	0.00000172	4.7	0.0351			****		***	****
972	315	Prelimbic area	15.87 ± 2.35	9	34.74 ± 14.44	8	15.72 ± 8.68	6	48.82 ± 17.46	7	0.354	0.557	4.67	0.04	0.356	0.556						
44	315	Infralimbic area	12.21 ± 3.93	8	35.13 ± 17.49	8	18.74 ± 9.72	4	31.73 ± 13.33	5	0.0078	0.93	2.18	0.154	0.13	0.722						
714	315	Orbital area	6.34 ± 1.19	7	7.98 ± 2.84	8	3.34 ± 2.17	5	21.92 ± 5.92	5	3.13	0.0915	6.82	0.0163	6.9	0.0158			*		*	**
723	714	Orbital area, lateral part	10.77 ± 2.29	7	11.16 ± 3.80	8	5.01 ± 3.25	5	32.94 ± 8.91	5	3.12	0.0918	5.77	0.0256	8.42	0.00854			*		*	**
95	315	Agranular insular area	3.64 ± 0.52	15	12.75 ± 3.56	16	4.46 ± 0.81	11	26.90 ± 4.31	11	6.94	0.0112	26.3	0.000005	5.38	0.0246			****		**	****
104	95	Agranular insular area, dorsal part	5.99 ± 0.62	14	19.66 ± 5.90	16	6.27 ± 1.28	11	43.61 ± 7.90	10	5.96	0.0184	21.8	0.0000255	5.69	0.0211			****		**	****
111	95	Agranular insular area, posterior part	3.64 ± 0.65	15	13.43 ± 3.77	14	5.34 ± 1.00	11	35.13 ± 5.96	11	11.7	0.00129	31.1	0.00000119	8.78	0.00476			****		***	****
119	95	Agranular insular area, ventral part	9.33 ± 1.29	14	21.88 ± 6.38	16	9.48 ± 2.44	11	49.42 ± 7.51	10	7.03	0.0109	20.2	0.0000453	6.88	0.0117			****		**	****
254	315	Retrosplenial area	8.94 ± 1.54	14	18.96 ± 4.67	16	10.39 ± 2.21	11	31.38 ± 6.32	12	2.9	0.095	12.2	0.00103	1.67	0.203			**			**
894	254	Retrosplenial area, lateral agranular part	15.69 ± 2.53	13	36.33 ± 8.78	16	17.56 ± 2.99	11	58.68 ± 11.60	11	2.88	0.0959	15.2	0.000295	1.83	0.183			**			**
879	254	Retrosplenial area, dorsal part	14.89 ± 2.66	14	34.03 ± 8.70	16	20.55 ± 4.50	11	62.34 ± 14.08	11	4.19	0.0461	11.4	0.00148	1.79	0.187			**			*
886	254	Retrosplenial area, ventral part	12.76 ± 2.38	14	22.66 ± 5.51	16	12.31 ± 3.00	11	36.93 ± 7.36	12	2.07	0.156	10.3	0.0023	2.1	0.154			**			*
22	315	Posterior parietal association areas	13.49 ± 2.82	14	30.90 ± 7.12	16	18.35 ± 3.07	11	49.98 ± 9.58	11	3.63	0.0625	13.3	0.000644	1.2	0.278			**			*
312782546	22	Anterior area	17.74 ± 4.47	15	47.39 ± 11.73	16	24.59 ± 5.71	11	70.61 ± 15.47	11	2.15	0.149	12.7	0.000833	0.629	0.432			**			*
417	22	Rostrolateral visual area	20.64 ± 5.79	14	47.69 ± 9.88	15	33.77 ± 6.07	11	98.49 ± 16.90	10	9.98	0.0028	17.6	0.000121	3.52	0.067			****		**	***
541	315	Temporal association areas	8.36 ± 1.77	13	20.77 ± 5.81	16	12.16 ± 2.09	11	45.41 ± 10.25	12	6	0.018	12.2	0.00104	2.9	0.0951			***		*	**
922	315	Perirhil area	1.61 ± 0.68	14	8.17 ± 2.23	16	3.21 ± 1.16	11	16.66 ± 6.16	12	2.71	0.106	9.07	0.00411	1.17	0.284			**			*
895	315	Ectorhil area	6.68 ± 1.54	14	17.96 ± 5.05	16	7.18 ± 1.77	11	30.29 ± 6.83	12	2.22	0.143	13.3	0.000654	1.71	0.198			**			**
698	695	Olfactory areas	7.85 ± 1.38	14	14.38 ± 3.93	15	7.86 ± 1.39	12	33.13 ± 5.98	12	6.83	0.0119	16.7	0.000159	6.59	0.0133			****		**	***
159	698	Anterior olfactory nucleus	14.53 ± 3.78	8	21.33 ± 6.60	7	13.43 ± 4.80	8	132.21 ± 58.22	6	4.13	0.0538	5.04	0.0346	4.57	0.0434			*		*	*
589	698	Taenia tecta	7.57 ± 1.68	12	17.36 ± 6.61	10	6.25 ± 0.92	9	23.18 ± 3.90	10	0.317	0.577	11.6	0.00159	0.835	0.367			*			*
597	589	Taenia tecta, dorsal part	15.30 ± 3.43	12	39.39 ± 13.96	9	11.96 ± 1.85	9	46.45 ± 8.09	10	0.0445	0.834	14.1	0.000606	0.441	0.511			*			*
814	698	Dorsal peduncular area	16.60 ± 4.15	13	50.30 ± 17.35	9	27.19 ± 9.69	8	69.07 ± 10.31	10	1.89	0.177	14.3	0.000561	0.149	0.702			**			
961	698	Piriform area	20.18 ± 2.77	12	32.56 ± 12.33	11	22.35 ± 3.81	9	95.32 ± 15.51	11	10.3	0.00268	17	0.00019	8.41	0.00609			****		***	***
619	698	Nucleus of the lateral olfactory tract	15.47 ± 6.13	10	40.34 ± 22.24	7	23.31 ± 8.47	8	41.71 ± 12.07	6	0.167	0.686	3.04	0.0928	0.0651	0.801						
260	619	Nucleus of the lateral olfactory tract, molecular layer	36.09 ± 14.87	10	31.02 ± 14.72	6	28.21 ± 10.92	8	43.82 ± 21.87	6	0.000853	0.977	0.0924	0.764	0.42	0.523						
268	619	Nucleus of the lateral olfactory tract, pyramidal layer	34.58 ± 14.31	10	110.60 ± 62.91	7	61.90 ± 21.29	8	104.47 ± 25.15	6	0.159	0.694	3.32	0.0797	0.247	0.623						
1139	619	Nucleus of the lateral olfactory tract, layer 3	23.78 ± 11.81	10	114.79 ± 88.86	7	43.27 ± 18.66	8	206.06 ± 173.41	6	0.404	0.53	2.49	0.126	0.206	0.654						
631	698	Cortical amygdalar area	11.12 ± 2.75	14	20.61 ± 6.10	13	8.48 ± 2.27	10	26.91 ± 5.97	12	0.173	0.68	8.27	0.00615	0.878	0.354						
639	631	Cortical amygdalar area, anterior part	48.15 ± 10.58	14	91.56 ± 17.99	12	40.61 ± 9.81	10	89.02 ± 15.67	12	0.125	0.725	10.4	0.00239	0.0311	0.861						
647	631	Cortical amygdalar area, posterior part	5.78 ± 1.43	13	14.89 ± 3.43	13	4.45 ± 1.18	9	29.35 ± 7.48	11	2.77	0.104	15.2	0.000343	3.5	0.0685			**			**
655	647	Cortical amygdalar area, posterior part, lateral zone	10.20 ± 3.12	13	25.85 ± 8.75	13	10.61 ± 3.31	9	58.92 ± 14.25	11	4.04	0.0509	12.4	0.00106	3.44	0.0706			**		*	**
663	647	Cortical amygdalar area, posterior part, medial zone	13.45 ± 4.01	11	32.59 ± 8.45	13	7.02 ± 1.80	9	53.96 ± 14.78	9	0.858	0.36	13.1	0.000872	2.61	0.115			*			**
788	698	Piriform-amygdalar area	12.94 ± 2.71	15	38.14 ± 15.13	15	16.46 ± 4.25	11	78.13 ± 12.67	11	4.16	0.0468	14.9	0.000343	2.92	0.0937			***			**
1089	695	Hippocampal formation	0.98 ± 0.17	15	7.42 ± 2.28	16	1.38 ± 0.25	12	11.56 ± 2.31	12	1.84	0.181	23	0.0000143	1.23	0.274	*		***			**
1080	1089	Hippocampal region	1.23 ± 0.20	15	10.25 ± 3.16	16	1.80 ± 0.33	12	15.48 ± 3.19	12	1.58	0.215	22.6	0.0000169	0.998	0.323	*		***			**
375	1080	Ammon's horn	1.28 ± 0.25	15	8.00 ± 2.30	16	2.23 ± 0.41	12	11.54 ± 2.26	12	1.78	0.188	21.6	0.0000238	0.586	0.448	*		***			**
382	375	Field CA1	1.69 ± 0.43	15	11.19 ± 3.70	16	3.82 ± 1.02	12	24.27 ± 4.65	12	6.45	0.0142	22.3	0.0000189	3.28	0.0761			****		*	***
423	375	Field CA2	2.06 ± 0.68	15	15.77 ± 5.94	16	2.98 ± 1.21	12	20.51 ± 6.74	12	0.378	0.542	11.4	0.00144	0.174	0.678			*			
463	375	Field CA3	3.57 ± 0.76	15	21.43 ± 6.24	16	5.17 ± 0.74	12	22.80 ± 5.27	11	0.116	0.735	16.8	0.000153	0.000687	0.979	*		*	*		*
726	1080	Dentate gyrus	3.51 ± 0.60	15	36.54 ± 12.74	16	4.13 ± 0.90	11	56.09 ± 12.35	12	1.22	0.275	19.3	0.0000588	0.997	0.323	*		**			**
10703	726	Dentate gyrus, molecular layer	5.54 ± 1.14	15	40.57 ± 14.01	16	5.66 ± 1.54	11	78.01 ± 19.84	12	2.52	0.119	17.6	0.000111	2.3	0.135			***			**
10704	726	Dentate gyrus, polymorph layer	7.13 ± 2.96	15	145.22 ± 50.71	16	7.85 ± 2.64	11	115.20 ± 29.64	12	0.208	0.65	14.3	0.000418	0.212	0.647	*			*		
632	726	Dentate gyrus, granule cell layer	10.85 ± 2.02	15	131.25 ± 48.03	16	14.92 ± 3.23	11	198.30 ± 45.79	12	1.07	0.305	17.6	0.000112	0.785	0.38			**			**
982	1080	Fasciola cinerea	8.20 ± 6.37	13	15.61 ± 6.04	16	6.97 ± 3.79	12	22.34 ± 9.92	11	0.177	0.676	2.59	0.114	0.349	0.557						
19	1080	Induseum griseum	0.51 ± 0.51	15	16.37 ± 12.13	13	11.87 ± 9.26	12	54.85 ± 19.84	12	4.3	0.0435	6.19	0.0164	1.32	0.256			**			
822	1089	Retrohippocampal region	1.78 ± 0.60	14	4.99 ± 1.86	16	1.74 ± 0.50	12	9.33 ± 2.84	11	1.63	0.208	8.95	0.00434	1.69	0.2			*			*
909	822	Entorhil area	2.08 ± 0.69	14	5.68 ± 1.80	16	1.95 ± 0.59	11	11.93 ± 3.69	11	2.52	0.119	10	0.00271	2.58	0.115			**			**
918	909	Entorhil area, lateral part	3.21 ± 1.07	14	9.29 ± 3.23	16	3.01 ± 0.91	11	18.42 ± 5.69	11	2.02	0.161	9.58	0.00328	2.08	0.155			*			*

1037	822	Postsubiculum	0.00 ± 0.00	5	67.12 ± 37.68	6	5.60 ± 5.60	5	31.90 ± 8.06	6	0.609	0.445	4.74	0.043	0.906	0.354						
502	822	Subiculum	7.28 ± 2.58	12	19.18 ± 12.82	12	6.74 ± 2.08	10	24.45 ± 8.55	7	0.0598	0.808	2.88	0.0982	0.117	0.734						
484682470	822	Prosubiculum	4.00 ± 2.67	10	14.14 ± 7.89	11	5.97 ± 2.93	10	31.01 ± 13.21	7	1.58	0.217	5.1	0.0304	1.12	0.298						
703	688	Cortical subplate	4.78 ± 0.76	15	12.95 ± 3.38	16	6.81 ± 1.22	12	28.76 ± 5.36	12	8.17	0.00614	20.1	0.0000413	4.77	0.0335			****		**	***
583	703	Claustrum	18.06 ± 4.12	15	41.60 ± 13.14	15	27.92 ± 4.68	12	108.11 ± 19.85	12	10.3	0.00236	16.9	0.000145	5.65	0.0214			****		**	***
942	703	Endopiriform nucleus	5.07 ± 0.83	14	13.44 ± 3.77	16	8.24 ± 1.53	12	34.08 ± 6.71	11	10.3	0.00235	17.3	0.000129	5.52	0.0229			****		**	***
952	942	Endopiriform nucleus, dorsal part	10.88 ± 1.85	14	27.75 ± 7.25	16	16.92 ± 3.05	12	62.27 ± 12.59	12	8.07	0.0065	15.9	0.000219	3.81	0.0565			****		**	***
966	942	Endopiriform nucleus, ventral part	8.68 ± 1.81	14	25.82 ± 8.54	16	15.78 ± 3.45	12	63.55 ± 15.57	12	6.74	0.0124	11.8	0.00122	3.02	0.0885			***		*	**
131	703	Lateral amygdalar nucleus	6.60 ± 1.94	14	37.24 ± 10.12	16	12.61 ± 3.04	12	56.53 ± 13.12	12	2.23	0.141	17.9	0.0000997	0.596	0.444	*		**			**
295	703	Basolateral amygdalar nucleus	6.04 ± 1.23	14	19.49 ± 5.65	14	9.68 ± 2.38	10	48.97 ± 8.65	11	10.4	0.00234	23	0.0000179	6.07	0.0177			****		**	****
303	295	Basolateral amygdalar nucleus, anterior part	16.63 ± 3.38	14	48.80 ± 11.50	14	28.29 ± 7.46	10	132.88 ± 22.42	11	14.9	0.000359	26.4	0.00000587	8.18	0.0064			****		***	****
311	295	Basolateral amygdalar nucleus, posterior part	8.28 ± 2.63	13	33.23 ± 14.68	14	8.01 ± 2.91	10	69.34 ± 14.70	10	2.71	0.107	13.4	0.000673	2.7	0.108			**			**
451	295	Basolateral amygdalar nucleus, ventral part	7.05 ± 2.08	12	16.31 ± 4.96	13	12.63 ± 5.91	10	66.23 ± 17.60	10	10.2	0.00272	10.5	0.00235	6.32	0.016			***		**	**
319	703	Basomedial amygdalar nucleus	11.38 ± 2.37	15	23.07 ± 7.24	14	12.68 ± 3.10	11	50.19 ± 9.85	12	5.13	0.028	14.2	0.000449	4.16	0.0468			***		*	**
327	319	Basomedial amygdalar nucleus, anterior part	29.25 ± 6.31	15	65.09 ± 22.99	12	37.64 ± 11.02	11	111.96 ± 22.29	12	2.65	0.11	11.3	0.00154	1.34	0.254			**			*
334	319	Basomedial amygdalar nucleus, posterior part	12.23 ± 3.28	14	32.57 ± 14.32	14	10.42 ± 3.11	10	71.97 ± 15.41	11	3.05	0.0876	12.3	0.00105	3.46	0.0696			**			**
780	703	Posterior amygdalar nucleus	9.90 ± 3.56	12	19.16 ± 6.28	14	11.00 ± 4.82	11	46.09 ± 17.83	9	2.58	0.115	5.23	0.0273	2.29	0.138			*			*
623	567	Cerebral nuclei	2.45 ± 0.42	15	5.85 ± 1.09	16	3.21 ± 0.49	12	8.61 ± 1.38	12	3.58	0.0641	20.9	0.0000316	1.13	0.292	*		***			**
477	623	Striatum	3.45 ± 0.60	15	8.44 ± 1.57	16	4.53 ± 0.72	12	13.19 ± 1.87	11	4.95	0.0306	25.1	0.00000724	1.99	0.164	*		****			***
485	477	Striatum dorsal region	2.24 ± 0.42	15	7.53 ± 1.59	16	3.05 ± 0.49	12	9.56 ± 1.97	12	1.19	0.281	19.9	0.0000454	0.219	0.642	*		**			**
672	485	Caudoputamen	5.17 ± 0.82	13	15.06 ± 3.18	16	6.09 ± 0.98	12	22.94 ± 3.61	10	2.93	0.0938	24.4	0.0000103	1.85	0.18	*		***			***
493	477	Striatum ventral region	4.46 ± 0.85	15	8.57 ± 1.50	15	6.01 ± 1.05	12	14.42 ± 2.91	12	4.85	0.0323	13	0.000721	1.64	0.206			***			**
56	493	Nucleus accumbens	6.96 ± 1.19	13	17.07 ± 3.95	14	11.88 ± 2.70	12	37.05 ± 8.24	10	7.97	0.00706	14	0.000507	3.03	0.0884			***		*	**
998	493	Fundus of striatum	20.09 ± 12.00	10	15.02 ± 4.99	4	18.77 ± 9.05	6	34.52 ± 11.43	5	0.282	0.601	0.259	0.616	0.693	0.414						
754	493	Olfactory tubercle	13.76 ± 3.44	13	18.63 ± 3.87	13	11.84 ± 2.05	13	22.29 ± 3.61	9	0.037	0.848	4.71	0.0356	0.677	0.415						
275	477	Lateral septal complex	11.84 ± 2.38	14	24.52 ± 5.16	13	11.39 ± 1.63	12	27.39 ± 6.30	12	0.0773	0.782	11.3	0.00153	0.152	0.699						
242	275	Lateral septal nucleus	18.88 ± 3.84	14	38.14 ± 7.65	13	17.90 ± 2.61	12	43.11 ± 10.51	12	0.0821	0.776	10.7	0.002	0.194	0.662						
258	242	Lateral septal nucleus, rostral (rostroventral) part	35.22 ± 7.40	14	75.02 ± 16.20	12	33.98 ± 4.59	12	78.72 ± 20.94	12	0.0071	0.933	9.72	0.00314	0.0331	0.856						
266	242	Lateral septal nucleus, ventral part	46.13 ± 15.73	10	79.99 ± 24.55	9	52.91 ± 19.22	11	139.14 ± 31.81	9	1.83	0.185	6.79	0.0134	1.3	0.262			*			
333	275	Septohippocampal nucleus	3.28 ± 2.62	13	22.64 ± 10.95	8	2.73 ± 1.84	10	19.55 ± 11.11	7	0.058	0.811	7.67	0.00904	0.0371	0.848						
278	477	Striatum-like amygdalar nuclei	8.81 ± 1.63	15	20.04 ± 4.80	15	12.96 ± 2.61	12	35.56 ± 5.61	12	6.17	0.0164	17.1	0.000135	2.06	0.158			****		*	**
23	278	Anterior amygdalar area	9.25 ± 4.31	10	51.94 ± 42.40	5	16.22 ± 1.95	7	18.98 ± 5.20	7	0.415	0.525	1.92	0.179	1.75	0.198						
292	278	Bed nucleus of the accessory olfactory tract	38.30 ± 15.47	12	24.81 ± 14.26	9	16.60 ± 9.79	4	63.24 ± 41.05	5	0.176	0.678	0.0767	0.784	1.8	0.192						
536	278	Central amygdalar nucleus	6.81 ± 1.33	14	16.85 ± 4.25	14	10.95 ± 2.52	12	33.78 ± 5.93	12	7.58	0.00832	17.5	0.000124	2.79	0.101			****		*	***
544	536	Central amygdalar nucleus, capsular part	12.53 ± 2.94	13	34.52 ± 7.58	14	34.44 ± 12.31	12	84.46 ± 17.21	12	11	0.00175	10.1	0.00266	1.65	0.205			***		*	*
551	536	Central amygdalar nucleus, lateral part	11.88 ± 3.31	13	39.91 ± 10.47	14	19.32 ± 4.71	12	83.61 ± 18.71	12	5.65	0.0216	16.9	0.00016	2.77	0.102			***		*	**
559	536	Central amygdalar nucleus, medial part	15.76 ± 3.68	14	31.16 ± 9.31	14	18.71 ± 4.73	12	54.77 ± 7.78	12	3.75	0.0587	13.3	0.000654	2.27	0.139			**			**
1105	278	Intercalated amygdalar nucleus	17.67 ± 4.93	15	42.19 ± 10.44	14	33.67 ± 5.81	12	78.20 ± 16.76	12	6.47	0.0142	11.3	0.0015	0.969	0.33			***			*
403	278	Medial amygdalar nucleus	28.63 ± 5.64	15	60.02 ± 16.42	15	40.35 ± 9.28	12	106.53 ± 18.44	12	4.72	0.0345	12.4	0.00093	1.69	0.2			***			**
803	623	Pallidum	2.22 ± 0.41	15	2.88 ± 0.54	15	2.72 ± 0.34	12	6.70 ± 1.54	12	7.34	0.00922	7.28	0.00948	4.35	0.0421			**		**	**
818	803	Pallidum, dorsal region	2.10 ± 0.44	15	2.49 ± 0.57	15	1.88 ± 0.43	12	4.10 ± 0.94	12	1.23	0.273	4.08	0.0489	2.2	0.145						
1022	818	Globus pallidus, exterl segment	4.04 ± 0.95	14	7.01 ± 1.65	14	4.53 ± 1.53	12	9.86 ± 2.12	12	1.11	0.298	6.57	0.0135	0.554	0.461						
1031	818	Globus pallidus, interl segment	5.93 ± 1.57	13	1.71 ± 0.74	14	2.90 ± 1.18	12	4.29 ± 1.57	11	0.035	0.852	1.62	0.209	4.79	0.0338						
835	803	Pallidum, ventral region	3.82 ± 0.86	15	5.14 ± 1.59	15	4.55 ± 0.79	12	11.38 ± 3.20	12	3.81	0.0565	4.53	0.0384	2.38	0.129			*			
342	835	Substantia innomita	7.75 ± 1.78	15	10.80 ± 3.38	14	9.40 ± 1.57	12	25.70 ± 7.04	11	4.64	0.0363	5.76	0.0203	3.17	0.0814			**		*	*
826	803	Pallidum, medial region	2.59 ± 1.01	14	3.62 ± 1.07	11	6.12 ± 2.18	11	7.46 ± 1.69	11	5.89	0.0195	0.854	0.361	0.0109	0.917						
904	826	Medial septal complex	3.89 ± 1.52	14	5.43 ± 1.60	11	7.90 ± 3.26	11	10.40 ± 2.17	11	4.18	0.047	1.08	0.306	0.0483	0.827						
564	904	Medial septal nucleus	0.00 ± 0.00	8	12.46 ± 5.86	6	7.26 ± 3.70	8	26.15 ± 12.72	8	1.86	0.184	4.96	0.0349	0.183	0.673						
596	904	Diagol band nucleus	8.83 ± 3.37	14	9.98 ± 3.28	8	15.12 ± 6.62	11	20.50 ± 4.05	11	3.49	0.0687	0.678	0.415	0.227	0.636						
343	8	Brain stem	2.88 ± 0.56	15	3.76 ± 0.77	16	4.56 ± 0.96	12	5.94 ± 0.79	12	6.24	0.0157	1.93	0.171	0.0997	0.753			*			
1129	343	Interbrain	3.28 ± 0.64	15	4.13 ± 0.73	16	5.44 ± 1.09	12	7.29 ± 0.98	12	9.82	0.00286	2.16	0.147	0.344	0.56			**			
549	1129	Thalamus	2.67 ± 0.55	14	3.03 ± 0.56	16	5.07 ± 1.15	12	7.56 ± 1.14	12	17	0.00014	2.01	0.162	1.58	0.214			**		**	
864	549	Thalamus, sensory-motor cortex related	2.40 ± 0.60	14	2.80 ± 0.59	16	6.25 ± 1.52	12	9.21 ± 1.51	12	23.7	0.0000118	1.69	0.199	1.46	0.233			***		***	
637	864	Ventral group of the dorsal thalamus	2.47 ± 0.49	14	3.26 ± 0.62	16	7.26 ± 1.86	12	11.95 ± 2.05	12	26.8	4.04E-06	3.13	0.0831	2.21	0.144			****		***	
629	637	Ventral anterior-lateral complex of the thalamus	3.02 ± 1.04	14	4.92 ± 1.32	14	11.96 ± 2.48	12	26.00 ± 8.50	12	12.9	0.000779	3.24	0.0782	2.11	0.153			**		**	
685	637	Ventral medial nucleus of the thalamus	5.75 ± 2.19	14	5.87 ± 1.38	15	14.30 ± 5.40	12	12.47 ± 2.51	12	6.19	0.0163	0.0873	0.769	0.102	0.75						
709	637	Ventral posterior complex of the thalamus	4.24 ± 0.78	13	4.95 ± 1.07	16	11.39 ± 3.00	12	19.35 ± 3.19	12	26.5	4.72E-06	2.65	0.11	2.9	0.0947			****		****	
718	709	Ventral posterolateral nucleus of the thalamus	4.68 ± 1.51	13	6.86 ± 1.64	16	12.00 ± 2.17	12	29.05 ± 4.85	12	31	1.08E-06	9.16	0.00394	7.5	0.00859			****		****	***
725	709	Ventral posterolateral nucleus of the thalamus, parvicellular part	1.22 ± 1.22	11	5.75 ± 3.38	12	23.15 ± 9.71	10	10.19 ± 4.66	8	6.01	0.019	0.571	0.455	2.49	0.123		*				
733	709	Ventral posteromedial nucleus of the thalamus	11.35 ± 1.98	13	11.51 ± 2.65	16	30.21 ± 9.60	12	46.32 ± 7.79	12	21.1	0.0000303	1.05	0.311	1.81	0.185			***		***	
741	709	Ventral posteromedial nucleus of the thalamus, parvicellular part	6.37 ± 2.90	8	5.80 ± 2.60	14	8.67 ± 4.39	10	10.89 ± 3.67	9	1.23	0.275	0.00176	0.967	0.16	0.691						
563807435	637	Posterior triangular thalamic nucleus	4.14 ± 1.71	9	11.10 ± 5.69	8	10.23 ± 5.49	10	19.08 ± 9.12	7	1.48	0.233	1.71	0.201	0.0271	0.87						
406	864	Subparafascicular nucleus	11.22 ± 3.51	11	10.21 ± 3.64	13	8.89 ± 3.13	11	14.14 ± 2.09	8	0.0348	0.853	0.242	0.625	0.837	0.366						
414	406	Subparafascicular nucleus, magnocellular part	36.42 ± 9.75	6	40.28 ± 13.70	8	21.66 ± 9.59	7	28.08 ± 11.27	4	1.22	0.282	0.419	0.524	0.0106	0.919						
422	406	Subparafascicular nucleus, parvicellular part	17.85 ± 9.94	9	10.97 ± 5.16	9	9.39 ± 4.65	8	25.45 ± 4.49	7	0.144	0.707	0.254	0.618	2.81	0.104						
1008	864	Geniculate group, dorsal thalamus	6.10 ± 3.08	14	4.43 ± 1.32	16	13.85 ± 3.28	12	16.86 ± 3.23	12	13.9	0.000496	0.00083	0.977	0.733	0.396			*		*	
475	1008	Medial geniculate complex	5.35 ± 3.32	8	2.34 ± 0.88	9	6.03 ± 2.30	10	17.27 ± 4.69	7	6.6	0.0154	1.21	0.28	6.13	0.0192			*		**	*
1072	475	Medial geniculate complex, dorsal part	14.98 ± 12.66	7	3.88 ± 2.67	7	11.77 ± 8.44	8	14.52 ± 6.65	7	0.171	0.683	0.232	0.634	0.673	0.42						
1079	475	Medial geniculate complex, ventral part	21.61 ± 12.70	6	7.03 ± 5.93	7	11.78 ± 6.94	8	40.79 ± 16.25	7	1.23	0.279	0.524	0.476	3.93	0.059						
1088	475	Medial geniculate complex, medial part	2.93 ± 2.03	8	4.02 ± 1.93	9	7.21 ± 3.52	10	36.22 ± 7.04	7	20.6	0.0000854	11.3	0.00215	13.2	0.00103			****		****	****
170	1008	Dorsal part of the lateral geniculate complex	9.03 ± 4.67	14	8.05 ± 2.63	16	26.75 ± 6.24	12	30.01 ± 6.23	12	16.6	0.000165	0.00273	0.959	0.188	0.666			*	*	*	
496345664	170	Dorsal part of the lateral geniculate complex, shell	17.45 ± 13.38	14	15.57 ± 6.56	15	37.03 ± 14.93	11	58.73 ± 19.99	11	5.31	0.0256	0.328	0.57	0.742	0.393						
496345668	170	Dorsal part of the lateral geniculate complex, core	19.42 ± 8.71	14	18.44 ± 5.48	16	58.75 ± 15.40	12	68.44 ± 13.65	12	17.4	0.000121	0.0462	0.831	0.246	0.622			*	*	**	
496345672	170	Dorsal part of the lateral geniculate complex, ipsilateral zone	18.23 ± 9.86	12	11.84 ± 5.57	16	51.56 ± 15.07	11	48.48 ± 13.97	11	10.4	0.00232	0.47	0.497	0.0231	0.88						
856	549	Thalamus, polymodal association cortex related	3.82 ± 0.75	15	4.49 ± 0.83	16	6.55 ± 1.42	12	10.01 ± 1.55	12	13.7	0.000521	2.69	0.107	1.55	0.218			**		**	
138	856	Lateral group of the dorsal thalamus	1.53 ± 0.36	14	2.62 ± 0.68	16	6.35 ± 1.46	12	14.76 ± 2.26	12	45.1	1.66E-08	10.2	0.00239	8.17	0.00618			****		****	***
218	138	Lateral posterior nucleus of the thalamus	3.64 ± 0.88	14	7.45 ± 2.19	16	14.30 ± 3.64	12	24.49 ± 3.91	12	25.7	5.93E-06	5.12	0.0281	1.34	0.252		*	****		***	
1020	138	Posterior complex of the thalamus	1.71 ± 0.68	14	3.71 ± 1.03	16	10.88 ± 1.92	12	37.87 ± 6.97	12	44.7	1.86E-08	14.4	0.000408	14.4	0.000405			****		****	****
1029	138	Posterior limiting nucleus of the thalamus	4.61 ± 2.37	11	1.65 ± 1.08	8	16.04 ± 7.65	10	20.63 ± 9.37	7	6.33	0.0171	0.00609	0.938	0.412	0.525						
325	138	Suprageniculate nucleus	11.73 ± 7.45	7	2.90 ± 2.90	6	7.00 ± 5.67	8	12.08 ± 5.03	7	0.091	0.766	0.0569	0.813	1.48	0.236						
560581551	138	Ethmoid nucleus of the thalamus	4.24 ± 1.68	13	3.64 ± 1.81	16	13.11 ± 7.06	12	15.87 ± 5.39	12	6.22	0.0161	0.00805	0.929	0.155	0.696						
239	856	Anterior group of the dorsal thalamus	3.48 ± 0.86	15	2.84 ± 0.52	14	6.35 ± 1.43	12	9.19 ± 1.57	12	16.9	0.000148	0.84	0.364	2.45	0.124			**		**	
255	239	Anteroventral nucleus of thalamus	18.61 ± 6.69	12	17.17 ± 11.25	10	5.72 ± 1.52	12	14.95 ± 9.01	10	1.14	0.291	0.266	0.609	0.5	0.484						
127	239	Anteromedial nucleus	2.65 ± 0.96	13	1.23 ± 0.57	10	2.47 ± 0.79	12	4.01 ± 1.73	10	1.13	0.293	0.00308	0.956	1.9	0.176						
1096	127	Anteromedial nucleus, dorsal part	7.06 ± 2.75	13	2.18 ± 1.16	10	6.29 ± 2.66	12	7.36 ± 3.96	10	0.443	0.509	0.461	0.501	1.1	0.299						

1104	127	Anteromedial nucleus, ventral part	2.77 ± 1.31	13	2.81 ± 1.57	10	2.56 ± 1.35	12	10.62 ± 5.43	9	1.63	0.209	2.23	0.143	2.43	0.127						
64	239	Anterodorsal nucleus	11.58 ± 3.18	11	4.07 ± 2.52	10	4.66 ± 1.87	11	13.38 ± 4.30	9	0.0388	0.845	0.0143	0.906	7.3	0.0103						
1120	239	Interanteromedial nucleus of the thalamus	19.96 ± 8.03	11	14.30 ± 7.61	9	29.34 ± 16.90	10	31.85 ± 9.77	9	1.35	0.254	0.0141	0.906	0.129	0.722						
1113	239	Interanterodorsal nucleus of the thalamus	26.86 ± 7.53	12	10.11 ± 4.04	10	44.01 ± 13.80	12	23.73 ± 9.49	9	2.51	0.121	3.63	0.0642	0.0316	0.86						
155	239	Lateral dorsal nucleus of thalamus	3.83 ± 1.44	15	5.77 ± 1.45	14	16.65 ± 4.23	12	22.12 ± 4.55	12	23	0.0000153	1.58	0.215	0.338	0.563		*	***		**	
444	856	Medial group of the dorsal thalamus	5.96 ± 1.29	15	6.53 ± 1.22	14	11.16 ± 2.74	12	13.02 ± 2.40	12	9.45	0.00341	0.369	0.546	0.113	0.738						
59	444	Intermediodorsal nucleus of the thalamus	17.53 ± 4.95	14	28.35 ± 7.05	15	46.41 ± 14.84	12	35.06 ± 8.03	12	3.81	0.0567	0.00276	0.958	1.51	0.226						
362	444	Mediodorsal nucleus of thalamus	12.01 ± 2.64	15	8.94 ± 1.51	14	21.58 ± 5.95	12	23.56 ± 4.89	12	9.64	0.00317	0.022	0.883	0.422	0.519						
366	444	Submedial nucleus of the thalamus	4.73 ± 1.47	14	9.34 ± 2.28	14	16.37 ± 5.22	11	33.61 ± 8.17	12	14.8	0.000359	5.21	0.0271	1.8	0.186			***		**	
1077	444	Perireunensis nucleus	33.24 ± 13.90	15	21.16 ± 6.68	14	20.68 ± 7.71	12	23.80 ± 8.54	12	0.252	0.618	0.274	0.603	0.562	0.457						
571	856	Midline group of the dorsal thalamus	16.71 ± 3.47	15	20.96 ± 5.75	16	22.16 ± 3.66	12	29.80 ± 5.67	12	2.15	0.149	1.34	0.253	0.121	0.73						
149	571	Paraventricular nucleus of the thalamus	70.48 ± 15.30	15	83.54 ± 23.49	16	104.84 ± 15.81	12	103.12 ± 20.20	12	1.85	0.179	0.0997	0.753	0.14	0.709						
15	571	Parataenial nucleus	32.56 ± 9.97	10	43.41 ± 28.05	7	24.20 ± 12.26	8	34.10 ± 22.72	7	0.238	0.63	0.3	0.588	0.000693	0.979						
181	571	Nucleus of reuniens	12.58 ± 3.94	15	17.13 ± 4.44	14	17.01 ± 5.00	12	27.06 ± 9.05	12	1.56	0.217	1.59	0.213	0.232	0.632						
560581559	571	Xiphoid thalamic nucleus	18.05 ± 5.14	15	44.65 ± 14.15	14	28.33 ± 8.83	12	50.01 ± 14.14	12	0.505	0.481	4.96	0.0306	0.0496	0.825						
51	856	Intralamir nuclei of the dorsal thalamus	6.72 ± 1.51	15	6.54 ± 2.17	16	9.63 ± 2.55	12	14.24 ± 2.25	12	6.3	0.0153	0.75	0.39	1.27	0.265						
189	51	Rhomboid nucleus	15.23 ± 4.47	15	14.24 ± 8.74	14	28.18 ± 9.49	12	40.04 ± 10.79	12	5.27	0.0261	0.381	0.54	0.586	0.448						
599	51	Central medial nucleus of the thalamus	26.49 ± 7.30	15	24.98 ± 8.35	15	40.46 ± 14.42	12	29.16 ± 5.45	12	0.961	0.332	0.406	0.527	0.279	0.599						
907	51	Paracentral nucleus	11.24 ± 4.29	14	8.11 ± 5.68	14	12.83 ± 3.18	12	43.34 ± 14.09	12	5.72	0.0208	2.61	0.113	4.77	0.0338			*		*	*
575	51	Central lateral nucleus of the thalamus	8.98 ± 2.14	14	15.37 ± 5.82	15	12.86 ± 3.90	12	23.07 ± 5.08	12	1.63	0.208	3.13	0.0833	0.176	0.677						
930	51	Parafascicular nucleus	7.84 ± 3.03	10	5.04 ± 1.86	11	15.59 ± 5.87	9	21.45 ± 6.41	7	7.72	0.00893	0.000454	0.983	1.03	0.318						
262	856	Reticular nucleus of the thalamus	2.86 ± 0.88	15	6.17 ± 1.88	15	12.59 ± 4.08	12	24.32 ± 4.81	12	21	0.0000305	5.45	0.0237	1.92	0.172			****		***	
1014	856	Geniculate group, ventral thalamus	17.71 ± 4.99	14	15.31 ± 4.82	16	24.37 ± 7.00	12	27.64 ± 7.49	12	2.55	0.117	0.000954	0.975	0.223	0.639						
27	1014	Intergeniculate leaflet of the lateral geniculate complex	45.24 ± 18.11	12	43.99 ± 17.84	11	85.14 ± 26.39	10	63.76 ± 28.60	8	1.88	0.179	0.247	0.622	0.201	0.657						
178	1014	Ventral part of the lateral geniculate complex	37.14 ± 10.87	14	34.44 ± 10.85	16	49.97 ± 14.77	12	59.16 ± 15.34	12	2.18	0.146	0.0239	0.878	0.215	0.645						
321	1014	Subgeniculate nucleus	0.00 ± 0.00	8	2.17 ± 2.17	11	3.08 ± 3.08	10	0.00 ± 0.00	7	0.0516	0.822	0.0308	0.862	1.4	0.245						
958	856	Epithalamus	6.10 ± 1.58	15	13.65 ± 3.24	16	7.64 ± 1.84	12	12.98 ± 1.92	12	0.0301	0.863	7.62	0.008	0.212	0.647						
483	958	Medial habenula	15.09 ± 5.40	15	37.64 ± 9.04	16	18.42 ± 4.55	12	39.76 ± 6.31	12	0.151	0.699	10	0.00258	0.00753	0.931						
186	958	Lateral habenula	8.92 ± 2.50	15	12.63 ± 4.44	15	11.94 ± 3.48	12	12.31 ± 2.77	12	0.15	0.7	0.417	0.521	0.23	0.634						
1097	1129	Hypothalamus	8.57 ± 1.57	14	11.14 ± 2.28	14	11.64 ± 2.17	12	13.68 ± 1.95	12	1.94	0.17	1.34	0.254	0.0171	0.897						
157	1097	Periventricular zone	14.32 ± 3.24	15	27.32 ± 7.97	15	26.68 ± 5.89	12	29.95 ± 5.43	12	1.56	0.218	2.12	0.152	0.659	0.421						
390	157	Supraoptic nucleus	39.56 ± 19.55	9	73.99 ± 35.32	6	69.59 ± 32.01	9	24.75 ± 11.90	7	0.0139	0.907	0.071	0.792	2.21	0.149						
38	157	Paraventricular hypothalamic nucleus	64.58 ± 16.26	13	146.01 ± 32.11	9	114.74 ± 26.80	11	143.40 ± 20.36	10	1.29	0.263	5.81	0.0207	1.24	0.273						
30	157	Periventricular hypothalamic nucleus, anterior part	6.15 ± 6.12	8	123.40 ± 50.10	6	32.05 ± 11.41	7	74.64 ± 24.67	8	0.148	0.703	9.25	0.00546	2.13	0.157	*					
118	157	Periventricular hypothalamic nucleus, intermediate part	13.61 ± 6.53	13	30.71 ± 12.87	15	21.70 ± 8.08	12	40.78 ± 12.21	12	0.738	0.395	2.8	0.101	0.00873	0.926						
223	157	Arcuate hypothalamic nucleus	27.88 ± 10.08	13	48.79 ± 15.14	15	20.26 ± 3.18	11	28.59 ± 7.21	11	1.59	0.214	2.04	0.16	0.316	0.577						
141	1097	Periventricular region	12.68 ± 2.64	14	15.90 ± 4.74	16	23.57 ± 7.24	12	26.53 ± 4.53	12	4.72	0.0345	0.313	0.579	0.000734	0.978						
830	141	Dorsomedial nucleus of the hypothalamus	26.31 ± 9.22	12	34.14 ± 12.81	14	57.21 ± 17.63	11	58.78 ± 11.62	11	4.44	0.0408	0.0889	0.767	0.057	0.812						
126	141	Periventricular hypothalamic nucleus, posterior part	9.65 ± 3.33	9	7.91 ± 2.83	10	27.75 ± 10.73	7	30.91 ± 9.94	8	9.06	0.00525	0.00679	0.935	0.127	0.724						
347	141	Subparaventricular zone	28.63 ± 12.54	13	61.05 ± 18.12	9	61.93 ± 25.98	10	67.29 ± 24.74	9	1.11	0.298	1.08	0.305	0.444	0.509						
286	141	Suprachiasmatic nucleus	27.10 ± 11.76	9	32.98 ± 16.34	6	127.07 ± 66.33	8	56.97 ± 40.23	7	2.63	0.117	0.46	0.504	0.827	0.371						
467	1097	Hypothalamic medial zone	13.95 ± 3.08	15	18.37 ± 4.04	16	15.77 ± 3.19	12	19.27 ± 2.85	12	0.152	0.698	1.35	0.251	0.0179	0.894						
88	467	Anterior hypothalamic nucleus	29.34 ± 6.99	14	47.55 ± 12.63	12	44.18 ± 11.01	12	37.89 ± 8.89	11	0.11	0.741	0.457	0.503	1.5	0.227						
331	467	Mammillary body	7.43 ± 1.73	10	7.76 ± 2.41	12	5.51 ± 1.33	11	11.43 ± 1.79	8	0.147	0.704	2.11	0.155	2.05	0.16						
210	331	Lateral mammillary nucleus	10.63 ± 10.63	7	11.75 ± 6.45	9	10.91 ± 9.01	8	19.10 ± 5.25	8	0.263	0.612	0.316	0.578	0.201	0.657						
491	331	Medial mammillary nucleus	6.62 ± 2.72	7	10.61 ± 4.53	9	4.45 ± 1.32	8	12.54 ± 2.57	7	0.000488	0.983	3.46	0.0736	0.401	0.532						
732	491	Medial mammillary nucleus, median part	24.04 ± 9.30	7	11.33 ± 5.68	9	11.22 ± 7.87	7	17.87 ± 9.93	7	0.101	0.753	0.181	0.674	1.42	0.244						
606826647	491	Medial mammillary nucleus, lateral part	9.39 ± 5.74	7	32.56 ± 13.80	9	5.34 ± 2.24	8	21.18 ± 7.23	7	0.719	0.404	4.92	0.0351	0.158	0.694						
606826651	491	Medial mammillary nucleus, medial part	10.68 ± 4.36	7	11.94 ± 9.02	9	2.83 ± 2.11	8	50.26 ± 6.94	6	5.01	0.034	10.1	0.00384	12.2	0.00176			**		**	***
525	331	Supramammillary nucleus	31.64 ± 9.01	9	25.51 ± 8.41	10	30.39 ± 9.72	10	56.99 ± 17.11	8	1.74	0.196	0.596	0.445	2.18	0.149						
557	331	Tuberomammillary nucleus	6.55 ± 2.91	10	8.52 ± 3.29	10	7.24 ± 2.01	9	3.66 ± 3.05	7	0.421	0.521	0.0152	0.903	0.897	0.351						
1126	557	Tuberomammillary nucleus, dorsal part	19.79 ± 14.23	8	37.19 ± 16.34	8	31.94 ± 11.87	6	10.26 ± 10.26	6	0.267	0.61	0.00209	0.964	1.87	0.185						
1	557	Tuberomammillary nucleus, ventral part	11.93 ± 4.90	10	8.34 ± 5.76	8	8.79 ± 2.96	9	1.77 ± 1.77	7	1.15	0.292	1.4	0.245	0.155	0.696						
980	467	Dorsal premammillary nucleus	43.19 ± 13.06	10	34.97 ± 16.02	9	26.05 ± 12.81	7	30.63 ± 5.05	8	0.675	0.418	0.0619	0.805	0.243	0.625						
1004	467	Ventral premammillary nucleus	26.98 ± 11.20	10	53.74 ± 19.02	10	32.81 ± 8.63	10	34.54 ± 9.46	8	0.205	0.654	1.36	0.252	0.903	0.349						
63	467	Paraventricular hypothalamic nucleus, descending division	58.38 ± 9.90	12	63.71 ± 13.47	12	110.43 ± 30.13	9	51.19 ± 11.03	10	1.29	0.263	1.9	0.176	3.8	0.0584						
693	467	Ventromedial hypothalamic nucleus	20.90 ± 4.19	14	28.53 ± 9.54	13	16.73 ± 4.27	11	48.49 ± 14.74	11	0.745	0.393	4.41	0.0414	1.83	0.183						
946	467	Posterior hypothalamic nucleus	48.44 ± 13.08	14	49.74 ± 11.82	15	62.74 ± 17.50	11	43.28 ± 7.54	9	0.104	0.749	0.309	0.581	0.58	0.45						
290	1097	Hypothalamic lateral zone	6.93 ± 1.31	15	9.35 ± 1.86	16	10.27 ± 1.86	12	12.79 ± 1.97	12	3.66	0.0614	1.89	0.176	0.0008	0.978						
194	290	Lateral hypothalamic area	16.10 ± 3.25	15	19.93 ± 5.00	16	25.93 ± 5.40	11	25.47 ± 5.20	12	2.54	0.117	0.187	0.667	0.202	0.655						
226	290	Lateral preoptic area	14.53 ± 12.15	4	12.58 ± 5.92	5	8.53 ± 4.51	5	31.69 ± 8.88	5	0.845	0.373	1.91	0.188	2.5	0.135						
364	290	Parasubthalamic nucleus	26.34 ± 10.25	12	55.39 ± 19.23	13	26.40 ± 6.64	11	63.91 ± 12.08	9	0.089	0.767	5.61	0.0227	0.0933	0.762						
576073704	290	Perifornical nucleus	12.82 ± 3.93	15	32.87 ± 12.64	14	32.68 ± 7.02	12	47.44 ± 9.73	10	3.75	0.0587	3.77	0.058	0.0863	0.77			*			
173	290	Retrochiasmatic area	5.61 ± 3.08	12	16.88 ± 12.58	7	9.31 ± 3.49	8	12.81 ± 8.15	7	0.00395	0.95	1.31	0.262	0.323	0.574						
470	290	Subthalamic nucleus	9.24 ± 3.82	10	6.24 ± 2.37	16	7.13 ± 2.98	11	24.28 ± 11.33	11	2.39	0.129	0.872	0.356	2.92	0.0946						
614	290	Tuberal nucleus	9.68 ± 2.13	14	29.43 ± 8.89	14	24.78 ± 5.34	11	29.23 ± 6.80	11	1.34	0.253	4.16	0.0472	1.42	0.24						
797	290	Zo incerta	12.57 ± 2.36	15	10.50 ± 3.03	16	16.06 ± 3.43	12	19.38 ± 2.99	12	4.37	0.0415	0.004	0.95	0.821	0.369						
804	797	Fields of Forel	9.00 ± 3.30	10	21.36 ± 9.59	10	19.94 ± 6.87	10	18.68 ± 7.87	8	0.388	0.537	0.616	0.438	0.874	0.356						
10671	1097	Median eminence	13.07 ± 13.07	12	8.60 ± 4.74	14	7.11 ± 5.83	11	18.56 ± 11.18	10	0.0509	0.823	0.0777	0.782	0.758	0.389						
313	343	Midbrain	10.46 ± 2.52	13	11.28 ± 3.78	15	12.52 ± 3.99	12	11.91 ± 3.27	11	0.145	0.706	0.00076	0.978	0.0412	0.84						
339	313	Midbrain, sensory related	13.92 ± 8.73	11	10.29 ± 5.43	11	4.57 ± 2.91	10	19.71 ± 4.02	7	0.0213	0.885	0.523	0.475	2.23	0.145						
302	339	Superior colliculus, sensory related	3.62 ± 2.45	6	25.89 ± 12.65	7	9.45 ± 6.26	7	34.78 ± 4.42	6	0.843	0.368	8.45	0.00817	0.0365	0.85						
851	302	Superior colliculus, optic layer	9.56 ± 7.75	4	44.10 ± 21.20	7	14.31 ± 9.21	7	52.15 ± 9.13	6	0.196	0.663	5.77	0.0261	0.0121	0.914						
834	302	Superior colliculus, zol layer	11.00 ± 8.22	5	57.27 ± 34.45	7	20.57 ± 12.61	7	46.00 ± 11.89	6	0.00395	0.95	2.66	0.118	0.225	0.64						
599626923	339	Subcommissural organ	25.25 ± 17.73	11	0.00 ± 0.00	9	6.40 ± 6.40	10	8.47 ± 8.47	7	0.373	0.546	1.15	0.292	1.34	0.255						
323	313	Midbrain, motor related	8.10 ± 1.78	13	8.19 ± 3.03	15	17.16 ± 7.01	12	12.37 ± 4.02	10	2.42	0.127	0.371	0.545	0.323	0.573						
381	323	Substantia nigra, reticular part	0.00 ± 0.00	6	2.46 ± 2.08	8	1.32 ± 1.32	8	3.87 ± 1.78	7	0.708	0.408	2.15	0.155	0.000899	0.976						
128	323	Midbrain reticular nucleus	8.21 ± 2.11	8	6.40 ± 2.12	10	9.82 ± 4.01	10	18.74 ± 4.60	7	4.08	0.0522	0.513	0.479	2.55	0.12						
795	323	Periaqueductal gray	21.49 ± 4.62	13	20.37 ± 7.09	14	35.34 ± 11.26	12	21.77 ± 6.05	10	1.03	0.317	0.875	0.354	0.646	0.426						
50	795	Precommissural nucleus	50.80 ± 12.39	10	42.81 ± 15.30	13	64.84 ± 20.75	12	99.93 ± 36.10	10	2.62	0.113	0.173	0.679	0.941	0.338						
67	795	Interstitial nucleus of Cajal	0.00 ± 0.00	4	40.00 ± 40.00	4	18.18 ± 18.18	8	2.56 ± 2.56	6	0.16	0.694	0.076	0.786	1.86	0.19						
587	795	Nucleus of Darkschewitsch	11.59 ± 11.59	4	2.92 ± 2.92	4	7.56 ± 5.48	8	28.33 ± 9.83	6	1.42	0.248	1.34	0.262	3.13	0.0937						
1100	323	Pretectal region	8.00 ± 1.87	12	15.56 ± 6.90	11	10.30 ± 4.35	10	13.15 ± 2.48	7	0.00237	0.961	1.45	0.237	0.251	0.62						
215	1100	Anterior pretectal nucleus	11.74 ± 3.08	12	24.09 ± 13.07	11	19.79 ± 6.68	10	26.81 ± 6.84	7	0.439	0.512	1.3	0.261	0.0946	0.76						
531	1100	Medial pretectal area	17.98 ± 13.20	6	28.38 ± 20.39	5	49.44 ± 49.44	4	30.68 ± 11.87	4	0.473	0.502	0.00215	0.964	0.33	0.574						

628	1100	Nucleus of the optic tract	16.47 ± 4.81	8	23.91 ± 8.96	8	10.44 ± 6.98	9	24.39 ± 3.43	7	0.202	0.656	2.68	0.112	0.24	0.628
634	1100	Nucleus of the posterior commissure	24.99 ± 10.14	9	52.78 ± 33.71	8	26.19 ± 12.23	8	25.68 ± 7.98	7	0.395	0.535	0.578	0.453	0.542	0.468
706	1100	Olivary pretectal nucleus	18.43 ± 9.41	6	8.94 ± 4.73	8	3.63 ± 2.33	6	25.77 ± 7.78	7	0.176	0.679	0.768	0.39	5.78	0.0247
1061	1100	Posterior pretectal nucleus	36.68 ± 15.04	8	30.34 ± 15.06	8	23.00 ± 17.77	9	22.33 ± 8.36	7	0.532	0.472	0.0352	0.853	0.035	0.853
549009203	1100	Retroparafascicular nucleus	14.66 ± 11.88	6	21.66 ± 12.46	8	4.17 ± 4.17	6	2.19 ± 2.19	5	2.13	0.159	0.219	0.644	0.19	0.667
348	313	Midbrain, behavioral state related	0.00 ± 0.00	5	3.98 ± 2.75	7	2.18 ± 1.66	7	5.40 ± 2.40	6	0.674	0.421	2.45	0.132	0.0305	0.863
374	348	Substantia nigra, compact part	0.00 ± 0.00	5	7.80 ± 7.80	5	1.01 ± 1.01	7	19.51 ± 16.19	6	0.448	0.511	2.21	0.154	0.335	0.569
