# Supplementary material for: Hyperactive neuronal networks facilitate tau spread in an Alzheimer’s disease mouse model
Source: bioRxiv. 2024 Dec 3:2024.12.01.625514. Preprint. [Version 2] doi: 10.1101/2024.12.01.625514 (PMC11642807; doi:10.1101/2024.12.01.625514)
Supplement: Supplementary file 5 [file media-5.pdf]

Supplemental material

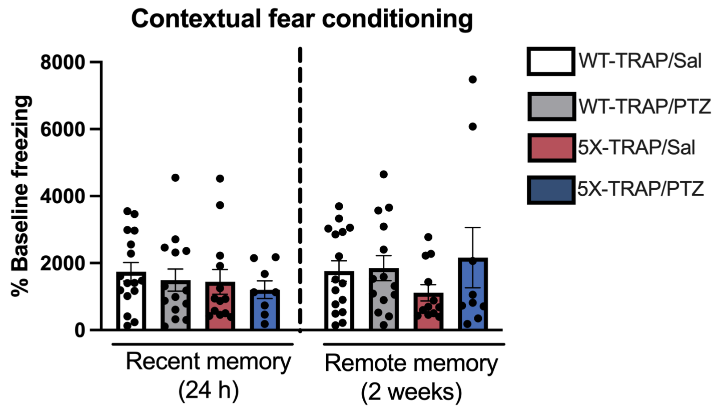

Figure S1. Results from contextual fear conditioning. Results are displayed as % change from baseline freezing levels to control for baseline differences in motor activity. Recent (1 day after conditioning) and remote (2 weeks after conditioning) recall were analyzed separately by 2-way ANOVA with genotype (WT-TRAP vs 5X-TRAP) and kindling (PTZ vs Sal) as independent variables. No significant effects were found. n=9-16/group. Histograms display group mean ± SEM.

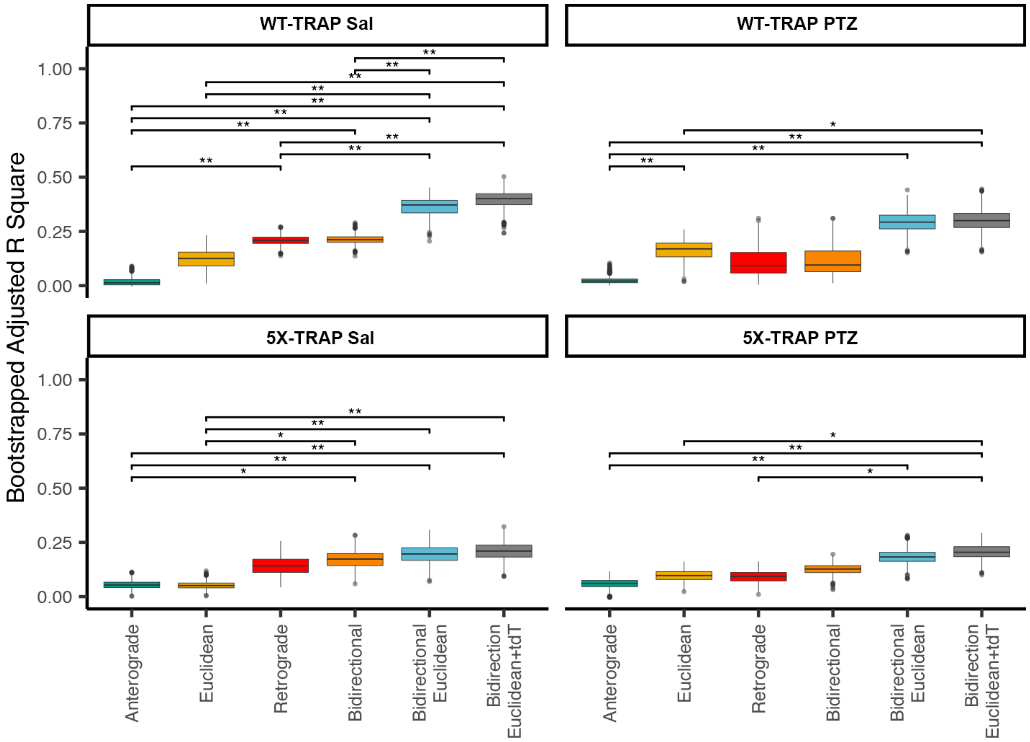

Figure S2. Between model comparisons of tau spread model fit for each experimental group. We fit spread models using a network defined by either anterograde connectivity, retrograde connectivity or Euclidean distance between regions. We also generated "bidirectional" models that combined anterograde and retrograde connections ("Bidirectional"), with the addition of Euclidean distance ("Bidirectional Euclidean") and a vector of tdT positive cell counts ("Bidirectional Euclidean + tdT"). We bootstrapped adjusted R square values for each of these model architectures to compare the model fit while accounting for the fact that increased model complexity will always improve training set performance. We compared bootstrapped adjusted R square models between groups for each model architecture. p-values were adjusted for multiple comparisons in each panel using FDR correction. *, $p<0.05$; **, $p<0.01$.

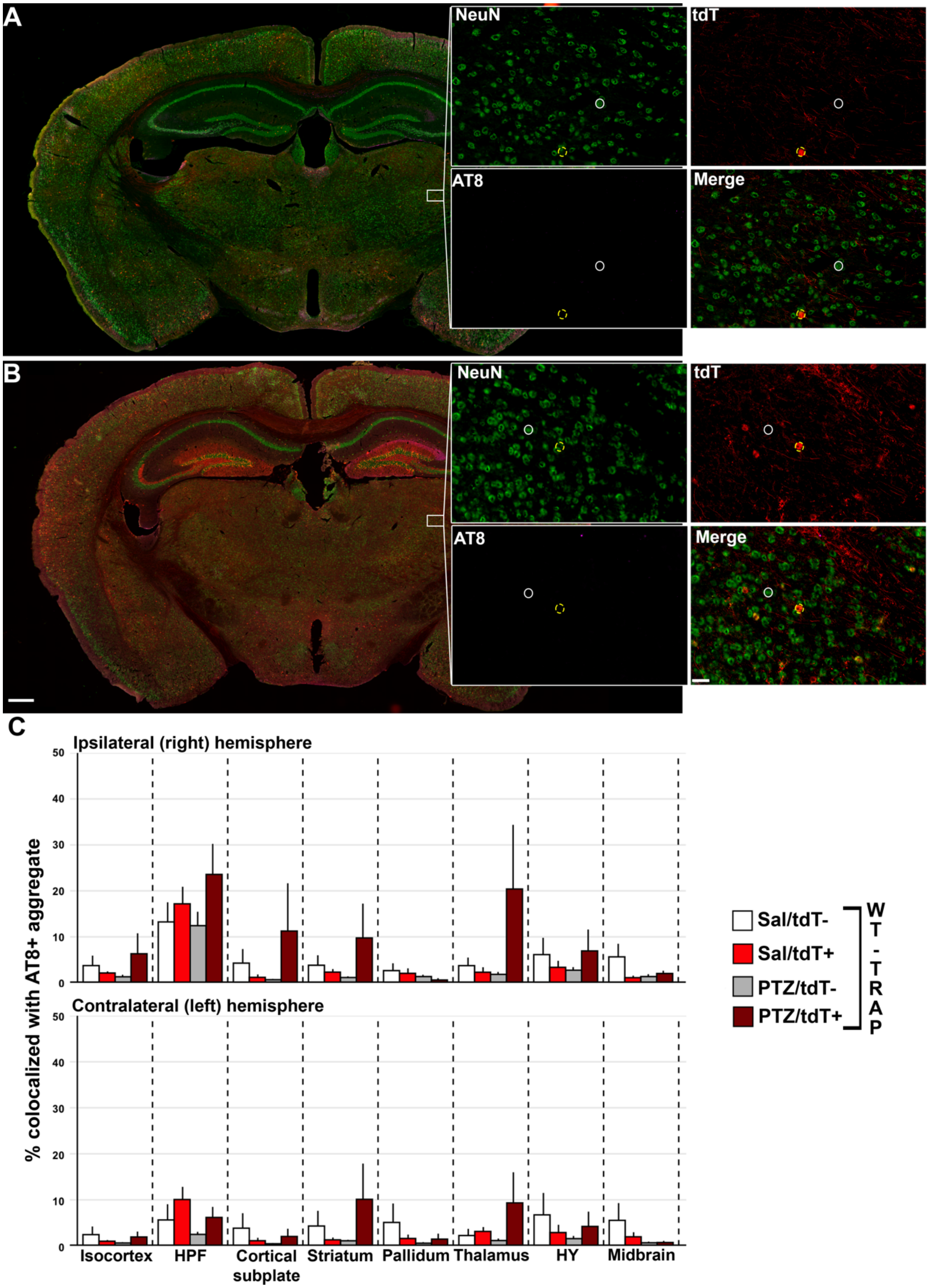

Figure S3. tdT+ and tdT- neurons colocalization with tau pathology in WT-TRAP mice.

Representative slide scanned coronal sections from (**A**) saline and (**B**) PTZ treated WT-TRAP

mice. Inset displays thalamic tdT+ (white circles) and tdT- (NeuN+) (yellow dashed circles) neurons colocalization with AT8+ tau aggregates. Scale bars = 500 μm, 20 μm (inset). (**C**) tdT+, tdT-, and colocalized AT8+ aggregates were mapped to the Allen Brain Atlas and % of detected tdT+/tdT- cells were quantified. Two-way ANOVA with treatment (Sal vs PTZ) and tdT labelling (tdT+ vs tdT-) as independent variables were performed. No significant differences were found. Histograms display group mean ± SEM.

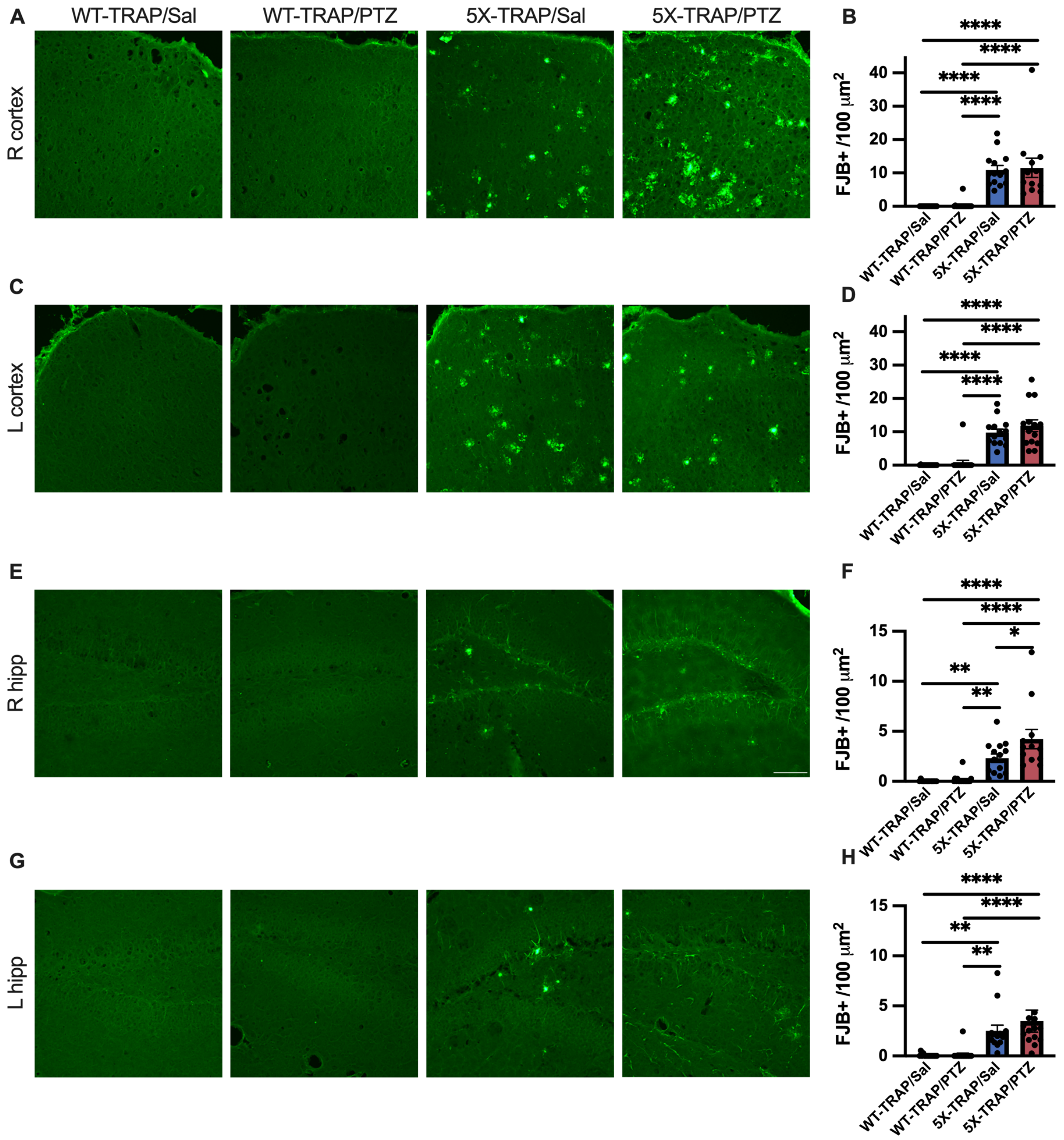

Figure S4. Neurodegeneration in 5X-TRAP mice is exacerbated by PTZ kindling.

Neurodegeneration was assessed by Fluorojade-B (FJB) stain. Representative images of FJB stain from the (**A**) ipsilateral to injection site (right, R) cortex and (**C**) contralateral (left, L) cortex, (**E**) ipsilateral hippocampus, and (**G**) contralateral hippocampus. Scale bar = 50 μm. FJB+

counts were compared by two-way ANOVA with genotype (WT-TRAP vs 5X-TRAP) and kindling (Sal vs PTZ) as independent variables. Increased FJB+ counts were found in Sal and PTZ treated 5X-TRAP mice compared to Sal and PTZ treated WT-TRAP mice in all sampled brain regions, and in PTZ kindled 5X-TRAP mice compared to Sal treated 5X-TRAP mice in the R hippocampus. Tukey’s post hoc: *,**, ****=$p<0.05$, 0.01, 0.0001. n=13-17/group. Histograms display group mean ± SEM.

Table S1. Results from AT8+ aggregate mapping in the hemisphere ipsilateral (right) to tau injection. Mean AT8+ aggregates/mm^2 +/- standard error of the mean and sample size for each experimental group (n) for each sampled brain region. Parent ID indicates the hierarchical "parent" area for each region. Results from two-way ANOVAs are displayed with F an p values for the main effects for each independent variable (genotype: WT-TRAP vs 5X-TRAP, kindling: Sal vs PTZ) and interaction. Results from Tukey's post hoc to compare individual groups are displayed where main effects or interactions were found: p1= WT-TRAP/Sal vs WT-TRAP/PTZ, p2=WT-TRAP/Sal vs 5X-TRAP/Sal, p3=WT-TRAP/Sal vs 5X-TRAP/PTZ, p4=WT-TRAP/PTZ vs 5X-TRAP/Sal, p5=WT-TRAP/PTZ vs 5X-TRAP/PTZ, p6=5X-TRAP/Sal vs 5X-TRAP/PTZ. *,**,***,****=p<0.05, 0.01, 0.001, 0.0001.

Table S2. Results from AT8+ aggregate mapping in the hemisphere contralateral (left) to tau injection. Mean AT8+ aggregates/mm^2 +/- standard error of the mean and sample size for each experimental group (n) for each sampled brain region. Parent ID indicates the hierarchical "parent" area for each region. Results from two-way ANOVAs are displayed with F an p values for the main effects for each independent variable (genotype: WT-TRAP vs 5X-TRAP, kindling: Sal vs PTZ) and interaction. Results from Tukey's post hoc to compare individual groups are displayed where main effects or interactions were found: p1= WT-TRAP/Sal vs WT-TRAP/PTZ, p2=WT-TRAP/Sal vs 5X-TRAP/Sal, p3=WT-TRAP/Sal vs 5X-TRAP/PTZ, p4=WT-TRAP/PTZ vs 5X-TRAP/Sal, p5=WT-TRAP/PTZ vs 5X-TRAP/PTZ, p6=5X-TRAP/Sal vs 5X-TRAP/PTZ. *,**,***,****=p<0.05, 0.01, 0.001, 0.0001.

Table S3. Results from tdTomato (tdT)+ count mapping in the hemisphere ipsilateral (right) to tau injection. Mean tdT+ cells/mm^2 +/- standard error of the mean and sample size for each experimental group (n) for each sampled brain region. Parent ID indicates the hierarchical "parent" area for each region. Results from two-way ANOVAs are displayed with F an p values for the main effects for each independent variable (genotype: WT-TRAP vs 5X-TRAP, kindling: Sal vs PTZ) and interaction. Results from Tukey's post hoc to compare individual groups are displayed where main effects or interactions were found: p1= WT-TRAP/Sal vs WT-TRAP/PTZ, p2=WT-TRAP/Sal vs 5X-TRAP/Sal, p3=WT-TRAP/Sal vs 5X-TRAP/PTZ, p4=WT-TRAP/PTZ vs 5X-TRAP/Sal, p5=WT-TRAP/PTZ vs 5X-TRAP/PTZ, p6=5X-TRAP/Sal vs 5X-TRAP/PTZ. *,**,***,****=p<0.05, 0.01, 0.001, 0.0001.

Table S4. Results from tdTomato (tdT)+ count mapping in the hemisphere contralateral (left) to tau injection. Mean tdT+ cells/mm^2 +/- standard error of the mean and sample size for each experimental group (n) for each sampled brain region. Parent ID indicates the hierarchical "parent" area for each region. Results from two-way ANOVAs are displayed with F an p values for the main effects for each independent variable (genotype: WT-TRAP vs 5X-TRAP, kindling: Sal vs PTZ) and interaction. Results from Tukey's post hoc to compare individual groups are displayed where main effects or interactions were found: p1= WT-TRAP/Sal vs WT-TRAP/PTZ, p2=WT-TRAP/Sal vs 5X-TRAP/Sal, p3=WT-TRAP/Sal vs 5X-TRAP/PTZ, p4=WT-TRAP/PTZ vs 5X-TRAP/Sal, p5=WT-TRAP/PTZ vs 5X-TRAP/PTZ, p6=5X-TRAP/Sal vs 5X-TRAP/PTZ. *,**,***,****=p<0.05, 0.01, 0.001, 0.0001.

Brain region	Dependent variable	Independent variable					
		Sex		Genotype		Kindling	
		t statistic (M)	p value	t statistic (WT)	p value	t statistic (Sal)	p value
R isocortex	AT8+/mm^2	-0.743	0.46	-4.58	<0.0001	-1.838	0.073
R olfactory areas		-0.3	0.77	-2.55	0.014	-0.143	0.89
R hippocampus		-2.52	0.015	-5.014	<0.0001	-0.355	0.72
R cortical subplate		-1.162	0.25	-5.533	<0.0001	-0.437	0.66
R striatum		-1.582	0.12	-8.122	<0.0001	-0.49	0.63
R thalamus		-1.43	0.16	-1.55	0.13	-0.766	0.48
R hypothalamus		-0.456	0.65	-1.187	0.24	-0.381	0.71
R midbrain		-1.948	0.0583	-0.395	0.7	0.144	0.88
R fiber tracts		-2.249	0.016	-6.894	<0.0001	-0.783	0.44
L isocortex		-1.558	0.13	-5.103	<0.0001	-4.089	0.073
L olfactory areas		-0.194	0.85	-3.213	0.0024	-0.757	0.453
L hippocampus		-3.002	0.0043	-5.559	<0.0001	-0.468	0.64
L cortical subplate		-0.28	0.79	-4.773	<0.0001	-1.026	0.3
L striatum		0.353	0.73	-5.746	<0.0001	-1.756	0.085
L Thalamus		-0.463	0.65	-4.681	<0.0001	-1.825	0.074
L hypothalamus		-0.0781	0.94	-0.52	0.6	0.271	0.79
L midbrain		-1.464	0.15	-1.171	0.25	-0.781	0.44
L fiber tracts		-1.285	0.2	-4.275	<0.0001	-2.012	0.05
R isocortex	tdT+/mm^2	1.844	0.071	-2.573	0.013	-4.872	<0.0001
R olfactory areas		2.164	0.035	-2.585	0.013	-4.296	<0.0001
R hippocampus		1.091	0.2803	-1.809	0.076	-4.476	<0.0001
R cortical subplate		1.824	0.074	-2.883	0.0057	-4.29	<0.0001
R striatum		1.937	0.058	-1.877	0.066	-4.644	<0.0001
R thalamus		0.118	0.91	-3.235	0.0021	-1.676	0.01
R hypothalamus		1.306	0.17	-1.576	0.12	-0.712	0.48
R midbrain		0.156	0.88	0.336	0.74	0.391	0.7
L isocortex		1.61	0.11	-2.66	0.011	-4.848	<0.0001
L olfactory areas		1.762	0.084	-2.802	0.0073	-4.103	0.00015
L hippocampus		0.887	0.38	-1.504	0.14	-4.844	<0.0001
L cortical subplate		1.555	0.13	-3.057	0.0035	-4.519	<0.0001
L striatum		1.142	0.16	-2.455	0.018	-5.148	<0.0001
L Thalamus		-0.0743	0.94	-3.939	0.00025	-1.519	0.14
L hypothalamus		1.753	0.086	-1.805	0.077	-1.326	0.19
L midbrain		0.322	0.75	-0.453	0.65	-0.099	0.92
	OF distance	-1.24	0.22	2.425	0.019	1.03	0.31
	OF center time	-0.181	0.86	-0.408	0.68	0.0131	0.99
	OF rearing	3.165	0.0025	4.585	<0.0001	-0.143	0.89
	CFC recent	-0.948	0.35	-1.309	0.2	-1.36	0.18
	CFC remote	-0.441	0.66	-0.887	0.38	-1.469	0.15
	NOR	-0.138	0.89	1.925	0.06	1.142	0.26
R hippocampus	SYNGR3/SB2	0.199	0.84	0.26	0.8	1.574	0.124
L hippocampus		-0.835	0.41	0.875	0.39	-1.019	0.31
R hippocampus	LRP1	-0.769	0.45	0.556	0.58	0.612	0.54
L hippocampus		-0.975	0.34	-1.494	0.14	0.561	0.578
L CA1	SYNGR (IHC)	1.201	0.25			1.693	0.11
R cortex	Fluorojade-B	-1.282	0.21	-7.895	<0.0001	-0.162	0.87
R hippocampus		-1.303	0.198	-6.412	<0.0001	-1.741	0.087
L cortex		-1.092	0.389	-9.865	<0.0001	-0.869	0.29
L hippocampus		-1.654	0.1	-4.957	<0.0001	-0.857	0.39

Table S5. Multiple linear regressions for sex effects in mouse studies. Multiple linear regression was performed including sex, genotype, and kindling as independent variables for

AT8+ aggregate and tdTomato (tdT)+ levels (Figure 1). We found that female sex (reference variable) was associated with increased AT8+ aggregate levels in the hippocampus ipsilateral (right, R) and contralateral (left, L) to AD-tau injection compared to male mice and that male sex was associated with increased tdT labelling in the R olfactory areas. Females displayed less rearing behavior in the open field assay.

Brain Region	Dependent variable	Independent variable					
		Sex		Seizure history		AD	
		t statistic (M)	p value	t statistic (+Sz)	p value	t statistic (AD)	p value
Amygdala	Tau pathology score	0.376	0.71	NA	NA		
Dentate gyrus		-0.434	0.66	-0.514	0.61		
CA/Subiculum		-7.851	0.432	NA	NA		
Entorhinal cortex		-0.152	0.88	NA	NA		
Middle frontal gyrus		0.0168	0.99	1.978	0.48		
Angular gyrus		0.0703	0.94	2.104	0.035		
SMT gyrus		0.41	0.68	1.351	0.18		
Cingulate gyrus		0.164	0.87	1.69	0.091		
Occipital cortex		-0.674	0.92	0.0997	0.5		
Striatum		0.926	0.35	0.775	0.44		
Globus pallidus		-0.392	0.7	0.0865	0.93		
Thalamus		-0.41	0.682	1.318	0.19		
Midbrain		1.441	0.15	1.251	0.21		
Substantia nigra		1.725	0.085	1.635	0.1		
Pons		0.398	0.25	1.146	0.25		
Locus Coeruleus		1.722	0.085	0.704	0.48		
Medulla		1.673	0.094	0.101	0.92		
Cerebellum		-0.555	0.58	NA	NA		
Temporal lobe cortex	SYNGR3/SYP	-2.11	0.042	2.715	0.01	-0.186	0.85
Temporal lobe cortex	LRP1	-0.94	0.36	1.224	0.24	0.0566	0.96

Table S6. Statistical analyses for sex effects in human studies. Ordinal linear regressions for regional tau pathology scores with seizure history and sex as independent variables. NA indicates that all values were the same in the AD+Sz group. Multiple linear regression was performed for western blot data with sex and seizure history as independent variables. Male sex was associated with reduced SYNGR3/SYP.

Antibody	Ref (Distributor)	Isotype	Size (kDa)
β-actin	A5441 (Sigma A.)	Mouse monoclonal IgG	43
LRP1	ab92544 (Abcam)	Rabbit polyclonal IgG	85
NeuN	ABN78 (EMD Millipore)	Rabbit polyclonal IgG	48/42
phospho-tau AT8 [Ser202; Thr205]	MN1020 (Millipore)	Mouse monoclonal IgG1κ	40-80
Synaptobrevin-2	104221 (Synaptic Systems)	Mouse monoclonal IgG1	15
Synaptogyrin-3	NBP2-30475 (Novus Bio)	Rabbit polyclonal IgG	25
Synaptophysin	5467S (Cell Signaling)	Rabbit monoclonal IgG	38

Table S7. Primary antibody information.

ID	Seizures	Age at death (years)	PMI	Braak stage	Sex	Age of onset	Brain weight (g)	Ventricular enlargement	Brain atrophy
**Con 1**	Unknown	57	24	0	Male		1297	no	mild
**Con 2**	Unknown	46	12	0	Female		1228	No	No
**Con 3**	Unknown	67	15	1	Male		1545	No	No
**Con 4**	Unknown	59	17	0	Male		1218	No	No
**Con 5**	Unknown	57	14	0	Male		1360	mild	No
**Con 6**	Unknown	68	17		Male		1371	None/Normal	None/Normal
**Con 7**	Unknown	72	17		Male		1216	None/Normal	Mild
**Con 8**	Unknown	68	14		Male		1512	None/Normal	None/Normal
**Con 9**	Unknown	65	19		Female		1207	None/Normal	None/Normal
AD-Sz1	Unknown	84	6	6	Female	78	1204	Mild	None/Normal
AD-Sz2	Unknown	90+	3	5	Male	76	1309	Severe	Mild
AD-Sz3	Unknown	78	20	5	Male	70	1190	Severe	Severe
AD-Sz4	Unknown	70	17	6	Male	61	1256	Moderate	Moderate
AD-Sz5	Unknown	90+	5	3	Female	86	1032	Severe	Severe
AD-Sz6	Unknown	79	19	6	Male	72	1345	Moderate	Mild
AD-Sz7	Unknown	88	10	6	Female	76	1094	Mild	Moderate
AD-Sz8	Unknown	90+	17		Female	89	1123	Mild	Mild
AD-Sz9	Unknown	87	4	6	Male	73	1198	Mild	Mild
AD-Sz10	Unknown	86	4	6	Male	72	1278	Moderate	Mild
AD-Sz11	Unknown	62	15	6	Female	56	988	None/Normal	Mild
AD-Sz12	Unknown	90+	9	5	Female	83	1124	None/Normal	Mild
AD-Sz13	Unknown	79	44	6	Female	69	909	Mild	Moderate
AD-Sz14	Unknown	83	14	5	Male	69	1155	Severe	Severe
AD-Sz15	Unknown	85	4	6	Female	76	1014	Moderate	Moderate
AD-Sz16	Unknown	44	3	6	Male	42	1510	None/Normal	None/Normal
AD-Sz17	Unknown	85	11	6	Female	78	1138	None/Normal	Moderate
AD-Sz18	Unknown	63	6		Male	54	1243	None/Normal	Mild
AD-Sz19	Unknown	74	4	6	Female	62	989	Moderate	Moderate
AD-Sz20	Unknown	84	20		Female	69	1151	Mild	Mild
AD-Sz21	Unknown	83	18.5	6	Female	76	1130	Mild	Mild
AD-Sz22	Unknown	70	12	6	Female	57	978	Severe	Severe
AD-Sz23	Unknown	71	6	6	Male	59	1330	Moderate	Moderate
AD-Sz24	Unknown	63	16	6	Female	53	1137	None/Normal	None/Normal
AD-Sz25	Unknown	84	16.5	6	Male	77	1281	Moderate	None/Normal
AD-Sz26	Unknown	78	6.5	6	Male	68	1100	None/Normal	Moderate
AD-Sz27	Unknown	65	17	6	Female	55	997	Severe	Severe
AD-Sz28	Unknown	84	39	5	Male	68	1185	Moderate	Severe
AD-Sz29	Unknown	90+	20	3	Female	86	938	Mild	Mild
AD-Sz30	Unknown	74	7	6	Female	64	1149	Mild	Mild

AD-Sz31	Unknown	70	12	6	Female	51	845	Severe	Severe
AD-Sz32	Unknown	64	16	6	Male	55	1320	None/Normal	None/Normal
AD-Sz33	Unknown	84	12	6	Male	69	1309	Moderate	Mild
AD-Sz34	Unknown	78	5		Male	64	926	Severe	Severe
AD-Sz35	Unknown	65	12	6	Male	59	960	Moderate	Severe
AD-Sz36	Unknown	64	11	6	Female	59	1098	Moderate	Severe
AD-Sz37	Unknown	72	4.5	6	Male	63	1167	Moderate	Moderate
AD-Sz38	Unknown	68	4	6	Male	54	1058	Moderate	Moderate
AD-Sz39	Unknown	88	14	6	Female	78	1145	Mild	None/Normal
AD-Sz40	Unknown	89	18.5	6	Female	72	1080	Severe	Moderate
AD-Sz41	Unknown	90+	18	6	Female	76	1283	Moderate	Moderate
AD-Sz42	Unknown	89	4.5	6	Female	76	1060	Severe	Severe
AD-Sz43	Unknown	85	20	5	Female	70	1069	Moderate	Mild
AD-Sz44	Unknown	90+	3	6	Male	82	1459		Moderate
AD-Sz45	Unknown	87	8	5	Female	82	1269	Mild	Moderate
AD-Sz46	Unknown	90+	18	6	Male	84	1105	Moderate	Moderate
AD-Sz47	Unknown	75	4.5	6	Male	61	1210	Severe	Mild
AD-Sz48	Unknown	84	6	5	Female	74	1053	Severe	Severe
AD-Sz49	Unknown	81	9	3	Male	73	1427	Moderate	Mild
AD-Sz50	Unknown	83	8	6	Male	66	1013	Moderate	Severe
AD-Sz51	Unknown	84	20	6	Female	78	1043	Moderate	Moderate
AD-Sz52	Unknown	90+	16	5	Male	83	1237	Moderate	Moderate
AD-Sz53	Unknown	61	4		Male	53	1203	Severe	Severe
AD-Sz54	Unknown	86	8	6	Male	75	1147	Severe	Moderate
AD-Sz55	Unknown	71	10	6	Male	62	1234	Mild	Moderate
AD-Sz56	Unknown	85	4	6	Female	66	969	Severe	Severe
AD-Sz57	Unknown	82	13	6	Female	71	1113	Moderate	Moderate
AD-Sz58	Unknown	86	27		Female	76	889	Mild	Moderate
AD-Sz59	Unknown	90+	22	6	Male	86	1232	Mild	Moderate
AD-Sz60	Unknown	79	7		Male	72	1147	Moderate	Mild
AD-Sz61	Unknown	85	15	3	Male	65	1404	Severe	Moderate
AD-Sz62	Unknown	83	20	6	Female	66	845	Severe	Severe
AD-Sz63	Unknown	68	14.5	4	Male	50	1140	Mild	Mild
AD-Sz64	Unknown	75	5	6	Female	65	1207	Moderate	Severe
AD-Sz65	Unknown	70	21	6	Male	56	1201	Severe	Severe
AD-Sz66	Unknown	74	22	6	Male	59	1173	Moderate	Mild
AD-Sz67	Unknown	90+	73	6	Female	83	1214	Mild	Mild
AD-Sz68	Unknown	85	14	6	Female	61	967	Severe	Severe
AD-Sz69	Unknown	88	20	6	Female	76	1205	Moderate	Mild
AD-Sz70	Unknown	63	15	6	Female	51	999	Severe	Moderate
AD-Sz71	Unknown	83	12	6	Male	73	1336	Moderate	Mild

AD-Sz72	Unknown	89	24	5	Female	73	1208	Moderate	Severe
AD-Sz73	Unknown	88	12	5	Female	72	1016	Severe	Severe
AD-Sz74	Unknown	88	15	4	Male	79	1177	Mild	Mild
AD-Sz75	Unknown	83	6	5	Male	71	1190	Moderate	Moderate
AD-Sz76	Unknown	85	48	4	Female	79	1202	Moderate	Moderate
AD-Sz77	Unknown	65	13	2	Female	56	1085	Severe	Mild
AD-Sz78	Unknown	66	4	3	Male	59	1135	None/Normal	Mild
AD-Sz79	Unknown	75	14	2	Male	69	1258	Moderate	Severe
AD-Sz80	Unknown	72	8	3	Female	64	1164	Mild	Mild
AD-Sz81	Unknown	73	27	5	Female	63	1022	Moderate	Moderate
AD-Sz82	Unknown	77	15	3	Female	70	1102	Moderate	Mild
AD-Sz83	Unknown	68	19	4	Male	62	1134	Mild	Mild
AD-Sz84	Unknown	75	9	6	Male	72	1257	None/Normal	Mild
AD-Sz85	Unknown	55	14	3	Female	49	956	Moderate	Moderate
AD-Sz86	Unknown	72	41	2	Male	68	1176	Moderate	Moderate
AD-Sz87	Unknown	63	21	3	Male	57	1424	Severe	Mild
AD-Sz88	Unknown	75	12	3	Female	70	1140	Mild	Moderate
AD-Sz89	Unknown	65	6.5	4	Male	60	1486	Mild	Mild
**AD-Sz91**	Unknown	79	5		Male	72	1426	Moderate	Mild
**AD-Sz92**	Unknown	72	11	6	Female	58	1092	Moderate	Moderate
AD-Sz93	Unknown	77	17		Male	68	1265	Severe	Moderate
**AD+Sz1**	Y	76	11	6	Female	59	953	severe	severe
**AD+Sz2**	Y	85	19	5-6	Female	73	1109	severe	mild/moderate
**AD+Sz3**	Y	90+	16	6	Female	68	980	severe	moderate
**AD+Sz4**	Y	68	5	5-6	Male	58	929	severe	severe
**AD+Sz5**	Y	79	8	6	Male	62	1005	severe	severe
**AD+Sz6**	Y	84	19	2	Male	71	1150	severe	moderate/severe
AD+Sz7	Y	77	10	6	Female	67	922	Moderate	Severe
AD+Sz8	Y	88	15		Male	77	1125	Mild	Mild
AD+Sz9	Y	89	3	6	Male	77	1034	Mild	Mild
AD+Sz10	Y	65	11	6	Female	55	1125	Moderate	Moderate
AD+Sz11	Y	83	11	6	Male	71	1077	Severe	Severe
AD+Sz12	Y	68	9	6	Female	60	1000	Mild	Moderate
AD+Sz13	Y	80	12	6	Female	67	1063	Moderate	Moderate
AD+Sz14	Y	68	12	6	Female	56	942	Severe	Severe
AD+Sz15	Y	71	20	6	Male	63	1164	Moderate	Moderate
AD+Sz16	Y	83	5	6	Female	59	783	Moderate	Severe
AD+Sz17	Y	81	45	6	Female	71	847	Severe	Moderate
AD+Sz18	Y	77	24	6	Female	71	1010	Mild	Moderate
AD+Sz19	Y	76	19	6	Female	66	1139	Mild	Mild

Table S8. Clinical information for human subjects. Bold indicates subjects included in western blot and italics indicates subjects included in tau pathology scores. PMI=postmortem interval.
